# MS-H: A Novel Proteomic Approach to Isolate and Type the *E. coli* H Antigen Using Membrane Filtration and Liquid Chromatography-Tandem Mass Spectrometry (LC-MS/MS)

**DOI:** 10.1371/journal.pone.0057339

**Published:** 2013-02-21

**Authors:** Keding Cheng, Mike Drebot, Joanne McCrea, Lorea Peterson, David Lee, Stuart McCorrister, Richard Nickel, Alyssia Gerbasi, Angela Sloan, Debra Janella, Gary Van Domselaar, Daniel Beniac, Tim Booth, Linda Chui, Helen Tabor, Garrett Westmacott, Matthew Gilmour, Gehua Wang

**Affiliations:** 1 National Microbiology Laboratory, Public Health Agency of Canada, Winnipeg, Manitoba, Canada; 2 Department of Human Anatomy and Cell Sciences, Faculty of Medicine, University of Manitoba, Winnipeg, Canada; 3 Alberta Provincial Laboratory for Public Health, Calgary, Canada; INRA Clermont-Ferrand Research Center, France

## Abstract

Serotyping is the long-standing gold standard method to determine *E. coli* H antigens; however, this method requires a panel of H-antigen specific antibodies and often culture-based induction of the H-antigen flagellar motility. In this study, a rapid and accurate method to isolate and identify the *Escherichia coli* (*E. coli*) H flagellar antigen was developed using membrane filtration and liquid chromatography-tandem mass spectrometry (LC-MS/MS). Flagella were isolated from pure culture, digested with trypsin, and then subjected to LC-MS/MS using one of two systems (Agilent-nano-LC-QSTAR XL or Proxeon-nano-LC-LTQ-Orbitrap XL). The resulting peptide sequence data were searched against a custom *E. coli* flagella/H antigen database. This approach was evaluated using flagella isolated from reference *E. coli* strains representing all 53 known H antigen types and 41 clinical *E. coli* strains. The resulting LC-MS/MS classifications of H antigen types (MS-H) were concordant with the known H serogroup for all 53 reference types, and of 41 clinical isolates tested, 38 (92.7%) were concordant with the known H serogroup. MS-H clearly also identified two clinical isolates (4.9%) that were untypeable by serotyping. Notably, successful detection and classification of flagellar antigens with MS-H did not generally require induction of motility, establishing this proteomic approach as more rapid and cost-effective than traditional methods, while providing equitable specificity for typing *E. coli* H antigens.

## Introduction

Traditional typing methods of *E. coli* bacteria involve biochemical tests and serotyping of O antigens (lipopolysaccharides) on the bacterial surface, K antigens from the capsule, and H antigens on the extracellular flagella [1]. Serotyping of the H antigen involves the examination of 53 distinct types of flagella (H1 to H56; designations H13, H22, and H50 no longer exist [2]), and is commonly used to identify and classify clinical and food-borne isolates of *E. coli,* with notable classifications including the most commonly seen O157:H7 group and the “non-O157” group representing other toxigenic strains [3]. However, conventional serotyping methodology based on antisera can be costly and laborious to perform due to varying quality of antibody preparations and the number of antibody agglutination reactions needed to assign a final classification [4], [5]. When bacterial cells do not generate lipopolysaccharide on the surface, the cultured colonies become “rough strains”, and both O and H antigen identification by antibody-based agglutination may be problematic despite the retention of cellular motility and presence of the H antigen flagellar structure [1], [5].

Molecular typing methods using polymerase chain reaction (PCR)-based amplification and targeted genetic sequencing are gaining popularity as a means for serotype classification due to their potential for higher throughput [4]–[6]. Other recent technologies for bacterial classification and identification include the application of mass spectrometry (MS) for bacterial nucleic acid detection, mass pattern analysis [7], and the quantification of bacterial proteins [8]. Matrix-associated laser-desorption/ionization time-of-flight (MALDI-TOF) MS usage for whole bacterial protein profiling to classify and type bacteria has also shown some promising results due to the ease of use and high throughput potential [9], [10].

Flagella are homopolymeric filaments comprising 40–60 kDa flagellin subunits, with *E. coli* flagellum filaments apporoximately15–20 µm long and 20 nm in diameter [11], [12]. Flagella have roles in bacterial motility, adhesion to substrates, biofilm formation, and virulence processes [12]. When studied in vitro, flagella are easily sheared off the bacterial surface by physical forces such as vortexing or thin-needle shearing, and can be purified by ultracentrifugation [11]. They are also heat-liable and easily digested into peptides at 37°C [12].

In this study, a method to rapidly determine *E. coli* H antigen types was developed, which combines the isolation of flagella on a filter membrane followed by enzyme digestion and online LC-MS/MS of the flagellin peptides using one of two LC-MS/MS platforms: Agilent-nano-LC-QSTAR XL (QSTAR in brief) or Proxeon-nano-LC-LTQ-Orbitrap XL (Orbitrap in brief). Comparing the resulting peptide sequence data to a custom reference H antigen protein database allowed for classification of H antigen types. When compared to traditional serotyping, this proteomic approach for *E. coli* flagellar H typing through MS, described here as MS-H, was found to be equally specific, but also a more rapid and reproducible means of obtaining H antigen type information without the requirement of antisera and motility induction.

## Results

### Method development: Proof-of-principle using H7 isolates, establishing a curated database of reference flagellar peptides, and determining the specificity of the method

Detailed procedures describing flagella purification, enzymatic digestion, and sample preparation for LC-MS/MS are described in the Materials and Methods. In brief, flagella were detached from their bacterial walls by vortexing a liquid *E. coli* cell suspension after overnight culture on agar. High speed centrifugation was then used to separate the flagella (in the supernatant) from the cellular pellet [12], [13]. Flagella were isolated on a membrane syringe filter which additionally provided an optimum substrate for rapid buffer exchange, minimal contamination, and efficient on-membrane trypsin digestion [14]. The digest was flushed out of the syringe filter, vacuum dried, and applied onto QSTAR for MS-H.

A curated database of reference flagellin proteins was established to enable the final classification of H types from peptide sequences deduced by LC-MS/MS and a Mascot search engine. This database included all available *E. coli* flagellin protein sequences from NCBI, with each sequence denoted by its known H antigen serogroup (Figure S1). Using this custom database and the Mascot search engine, the identity and classification of H antigen serogroups from flagellin peptide data was determined by using a minimum of two serogroup-specific peptide sequences [15]. MS-H types were assigned as the top scoring hit in the identified protein list possessing the highest confidence score. If more than one H type represented the top scoring hit, the result would be considered ambiguous.

To determine the method’s ability to rapidly isolate flagella and classify H antigen serogroups after LC-MS/MS, 11 *E. coli* reference isolates known to express the H7 antigen and one known non-motile *E. coli* reference isolate (E32511) were tested. Duplicate experiments were performed on *E. coli* strains cultured from frozen stocks without any induction of motility. For each of the H7 isolates, a minimum of 60% peptide sequence coverage was obtained based on the reference H7 protein sequences included in the curated database for all of the *E. coli* flagellin protein sequences, and in all instances H7 was the top-scoring hit, indicating 100% specificity. For the known non-motile strain E32511, there were no matches to flagellin peptides (Table 1).

**Table 1:** H7 identification of reference strains by MS-H with the QSTAR platform^a^.

Strain number	MS-H type	Sequence coverage (%)	
		Test 1	Test 2
EDL-933	H7	72	68
E175	H7	72	86
06-1139	H7	79	76
06-3122	H7	80	90
07-0097	H7	67	61
07-0918	H7	61	74
07-1591	H7	78	80
07-1756	H7	75	88
07-1946	H7	75	88
87-1215	H7	82	87
90-2380	H7	71	78
E32511 (non-motile)	No flagellin detected	0	0

a 11 known H7 positive *E. coli* strains and one non-motile strain (E32511) were twice tested for MS-H, and the sequence coverage were obtained by Mascot database search.

Analytical sensitivity [16], [17] was determined by diluting the flagellin digest (dilution factor ranged from 2 to 100) of one reference strain (87-1215). Since a major component of the syringe filter digests was the added trypsin, a parallel experiment was done to purify the intact flagella by ultracentrifugation of the flagella-containing supernatant [18], after which protein quantitation was carried out on the intact flagellin. This flagellin identification method was found to be very sensitive, as good sequence coverage and an accurate identification of H7 antigen was achieved with the QSTAR using a sub-microgram detection level of flagella. In general, a higher flagella concentration yielded higher protein sequence coverage (Table S1).

### Advanced evaluation of specificity by testing the full panel of H antigen types

MS-H typing of all *E. coli* H antigens was completed using bacterial stocks of reference strains. All 53 types were successfully identified from overnight cultures of frozen stocks without motility induction (Table 2). For strains that lost flagellar motility, confirmed by subsequent electron microscopy (EM) observations and motility tests, alternate reference strains having the same H types were selected for analyses. This examination also confirmed that the curated database was suitable for specific identification of all H types. Detection of flagella by EM (Figure S2) and characterization of intact flagellin by SDS-PAGE (Figure S3 **)** showed that the production of flagella and the expression of flagellin were quite heterogeneous.

**Table 2:** MS-H identification of all references strains with the QSTAR platform^a^.

Recorded H serotype	Representative strain	MS-H types with no motility induced	MS-H type sequence coverage (%)	Number of strains tested by MS-H
H1	E169	H1	61	1
H2	E170	H2	81	2 (1 UI)
H3	E171	H3	49	1
H4	E172	H4	88	1
H5	E173	H5	45	1
H6	E174	H6	65	1
H7	E175	H7	68	1
H8	E176	H8	78	1
H9	E177	H9	74	1
H10	E659	H10	62	2 (1 UI)
H11	07-6285	H11	70	2 (1 UI)
H12	E241	H12	47	1
H14	E182	H14	89	1
H15	E183	H15	93	1
H16	E184	H16	81	1
H17	E185	H17	87	1
H18	E186	H18	30	3 (1 UI)
H19	09-0523	H19	69	2
H20	E188	H20	94	1
H21	E189	H21	71	1
H23	E191	H23	69	1
H24	E192	H24	88	1
H25	E193	H25	86	1
H26	E194	H26	95	1
H27	E195	H27	76	1
H28	E196	H28	49	1
H29	E197	H29	98	1
H30	E198	H30	58	1
H31	E199	H31	49	1
H32	E200	H32	70	3 (1 UI)
H33	E201	H33	70	1
H34	E589	H34	62	1
H35	E203	H35	58	2
H36	E204	H36	89	1
H37	E205	H37	81	1
H38	E206	H38	99	1
H39	E207	H39	81	1
H40	E208	H40	95	1
H41	E209	H41	79	1
H42	E210	H42	29	2
H43	E211	H43	83	1
H44	E212	H44	87	1
H45	E213	H45	47	1
H46	E214	H46	37	1
H47	E346	H47	58	1
H48	E247	H48	70	1
H49	E248	H49	79	1
H51	E372	H51	43	1
H52	E373	H52	47	1
H53	E374	H53	74	1
H54	E377	H54	76	1
H55	E375	H55	74	1
H56	E376	H56	46	1

UI, unidentified strain.

a Known reference strains encompassing all 53 H types were tested by MS-H. If a primary strain could not be identified after three consecutive MS-H analyses, an alternate strain was selected for MS-H typing.

A comparison of MS-H and serotyping was then performed on 41 clinical isolates randomly chosen over a three-month period from incoming *E. coli* samples for routine serotyping. 38 samples gave identical results for both MS-H and the traditional serotyping method (Table 3). However, strain 09-0417, which was H7 by serotyping and then became untypeable, and strain 09-1760, which was also untypeable, were confirmed to be H21 by MS-H and DNA sequencing (Table S2). Strain 09-1775 (serotype H25), an unstable strain that became rough during the serotyping process, exhibited low sequence coverage for MS-H (H4, coverage 8%, Table 3). This isolate was confirmed to be MS-H 25 later by the more sensitive Orbitrap system for side-by-side comparison of MS-H typing and serotyping. In summary, 92.7% (i.e. 38 of 41 isolates) of the MS-H results matched the corresponding serotyping result, with two untypeable strains (i.e. 2 of 41 isolates, 4.9%) by serotyping being clearly assigned to unique H types by MS-H. Notably, the traditional serotyping analysis may have been inconsistent due to the possibility of isolates changing their phenotypes from smooth to rough during the cell culture and motility induction processes.

**Table 3:** Comparison of H serotyping and MS-H typing results for clinical isolates with the QSTAR platform^a^.

Strain number	Motility	Serotypes with motility induced	MS-H (without motility induction) / sequence coverage	
			H types	Sequence coverage (%)
09-0409	M	H28	H28	50
09-0410	M	H28	H28	40
09-0411	M	H11	H11	58
09-0412	M	H49	H49	42
09-0413	M	H16	H16	53
09-0414	M	H7	H7	51
09-0415	M	H8	H8	38
09-0416	M	H28	H28	44
09-0417	M	H7, then untypeable	H21^b^	80
09-1340	M	H11	H11	52
09-1341	M	H11	H11	63
09-1342	M	H11	H11	57
09-1343	M	H11	H11	50
09-1344	M	H11	H11	52
09-1347	M	H19	H19	56
09-1348	M	H19	H19	41
09-1349	M	H7	H7	39
09-1350	M	H7	H7	45
09-1351	M	H7	H7	41
09-1352	M	H7	H7	39
09-1353	M	H25	H25	42
09-1354	M	H25	H25	28
09-1760	M	untypeable	H21^b^	71
09-1764	M	H19	H19	41
09-1765	M	H11	H11	77
09-1766	M	H34	H34	14
09-1767	M	H11	H11	72
09-1768	M	H14	H14	40
09-1769	M	H14	H14	65
09-1770	M	H19	H19	45
09-1774	M	H19	H19	49
09-1775	M	H25, then rough	H4	8
09-2554	M	H21	H21	73
09-2555	M	H21	H21	72
09-2560	M	H21	H21	83
09-1336	NM		UI	
09-1337	NM		UI	
09-1338	NM		UI	
09-1339	NM		UI	
09-1345	NM		UI	
09-1346	NM		UI	

M, motile; NM, non-motile; UI, unidentifiable.

a Incoming clinical *E. coli* samples, collected over a three-month period for routine serotyping, were selected and MS-H was performed independently without motility induction; ^b^PCR-based DNA sequencing carried out for confirmation of H21.

### A side-by-side comparison of MS-H typing and serotyping

During the initial method development and advanced evaluation stages, MS-H identification of *E. coli* H types was compared to previous independent serotyping results (“gold standards”) with stock isolates. However, *E. coli* strains can be quite heterogeneous and dynamic in terms of flagella growth and flagellin production as shown above. For example, previously identified motile reference strains may not produce flagella from their frozen stocks, and some clinical strains may become rough during the subculture and motility induction processes. A side-by-side comparison was therefore required to further evaluate MS-H typing with serotyping. Four reference strains of known H type were randomly selected for side-by-side testing of motility, serotyping, and MS-H on the QSTAR consecutively for 16 days. After 24 hours of culturing, only one strain could be typed by traditional serotyping, but MS-H was able to identify three. On day 7, both serotyping and MS-H could identify three of the four strains, while strain E-375 (H55) turned rough and was no longer typeable by the traditional method. On day 16 of motility induction, E-375 remained rough, but could be identified as MS-H 55 using flagellin extracted from the motility-induced culture (Table S3). This indicates that MS-H can be successfully performed on rough strains after motility induction.

A side-by-side comparison between serotyping and MS-H for *E. coli* flagella identification was then expanded to 12 previously-typed H7 strains and other reference strains encompassing all 53 H types using the Orbitrap platform. The repeatability of MS-H identification was also tested by performing three tests on each strain. Serotyping was arranged to be done in parallel with MS-H on the first culture from a single colony (or the second day culture from a frozen stock) on plates without motility induction. 65 of 66 strains were correctly identified by MS-H from the primary culture of a single colony, while only 31 strains showed expected serotyping results without motility induction (Table 4). Three strains with unstable MS-H results (i.e. poor repeatability), including E375 tested above, were re-tested for MS-H and serotyping after motility induction over 7 days. Among them, E241 (H12), a reference strain that gave a solid identification result from MS-H two years earlier with the QSTAR (Table 2 **)**, turned non-motile and was unidentifiable by both methods here, and E210 and E375, were unidentifiable on day 1 by serotyping, and on day 2 (E210 and E375) and day 3 (E375) by MS-H. After motility induction, these three non-motile or “sluggish” strains were successfully identified by MS-H repeatedly, while serotyping was able to identify all but rough strain E375 (Table 4). Strain E204, another rough strain, was successfully identified as MS-H 36 even without motility induction. The serotyping-untypable strain 09-1760 (MS-H 21, confirmed by PCR-based sequencing; Table 3), was reconfirmed by MS-H as H21 on the Orbitrap platform, which matched the correct serotype titrated by the designated antiserum of H21. Table 4 also shows that MS-H can achieve good sequence coverage (50% or higher) with the Orbitrap platform using very stringent database search parameters [19] from a much smaller fraction of prepared sample (1/120 of the total digest) and that vacuum-drying of the sample is not a necessity.

**Table 4:** Side-by-side comparison of H serotyping and MS-H typing of *E. coli* H types with the Orbitrap platform^a^.

Strain Number	Previously recorded H-types with induced motility	Serotyping without induced motility	MS-H	Sequence coverage (%)		
				Day 1	Day 2	Day 3
06-4319	H7	H7	H7	54	62	87
06-1139	H7	–	H7	87	84	87
07-1591	H7	–	H7	93	89	81
07-1756	H7	–	H7	85	87	87
EDL933	H7	H7	H7	90	71	88
90-2380	H7	–	H7	85	83	90
05-0958	H7	–	H7	81	62	88
09-0414	H7	–	H7	94	79	89
09-1349	H7	–	H7	94	88	90
09-1350	H7	–	H7	91	73	90
09-1351	H7	–	H7	77	72	88
09-1352	H7	–	H7	90	83	88
E169	H1	H1	H1	98	83	83
E170	H2	H2	H2	67	71	68
E171	H3	–	H3	92	90	86
E172	H4	H4	H4	89	99	88
E173	H5	–	H5	81	76	58
E174	H6	H6	H6	90	80	63
E176	H8	–	H8	90	90	77
E177	H9	H9	H9	80	77	80
E659	H10	–	H10	99	79	85
07-6285	H11	–	H11	85	84	80
E241	H12	–	–	–	–	–
E241M^b^	H12	H12	H12	98	97	97
E752	H12	–	H12	98	91	97
E182	H14	H14	H14	47	81	55
E183	H15	H15	H15	73	81	79
E184	H16	–	H16	64	81	77
E185	H17	–	H17	74	85	84
E186	H18	H18	H18	58	68	65
09-0523	H19	H19	H19	66	89	88
E188	H20	H20	H20	81	73	81
E189	H21	H21	H21	95	98	95
E191	H23	H23	H23	65	67	79
E192	H24	H24	H24	72	65	74
E193	H25	H25	H25	66	73	68
E194	H26	H26	H26	98	75	85
E195	H27	H27	H27	71	63	69
E196	H28	–	H28	70	73	79
E197	H29	H29	H29	83	87	87
E198	H30	–	H30	78	78	79
E199	H31	–	H31	59	40	76
E200	H32	H32	H32	72	42	67
E201	H33	–	H33	70	65	75
E589	H34	H34	H34	65	66	67
E203	H35	–	H35	51	56	45
E204	H36	rough	H36	90	90	92
E205	H37	–	H37	96	91	85
E206	H38	H38	H38	97	98	86
E207	H39	H39	H39	83	68	61
E208	H40	H40	H40	97	92	91
E209	H41	–	H41	72	47	50
E210	H42	–	H42	48	–	36
E210M^b^	H42	H42	H42	72	70	74
E211	H43	–	H43	90	89	89
E212	H44	H44	H44	78	74	76
E213	H45	–	H45	61	60	53
E214	H46	H46	H46	73	68	59
E346	H47	H47	H47	99	83	99
E247	H48	H48	H48	89	91	82
E248	H49	H49	H49	87	87	87
E372	H51	–	H51	84	63	56
E373	H52	–	H52	85	67	59
E374	H53	H53	H53	72	58	74
E377	H54	–	H54	81	68	70
E375	H55	–	H55	63	–	–
E375M^b^	H55	rough	H55	71	71	65
E376	H56	–	H56	65	40	53
09-1760^c^	Untypeable	H21^d^	H21	97	95	96

-, serotyping titration or MS identification was not reached.

a Serotyping and MS-H were performed concurrently from subcultures of single bacterial colonies. MS-H was repeated on two consecutive days; **^b^**motility induction was performed for these inconsistent strains after initial MS-H; ^c^untypeable by serotyping although previous MS and PCR-based sequencing showed type H21; ^d^using designated antisera by MS-H.

Diagnostic sensitivity and specificity, run-to-run repeatability and instrument-to-instrument reproducibility were also tested on the Orbitrap platform using the earlier clinical strains and the residual flagellin digests from earlier method evaluation on the QSTAR. MS-H reaches 100% diagnostic specificity and 100% diagnostic sensitivity with the Orbtrap platform. In addition, the Orbitrap instrument gave consistent results when runs were performed in triplicate on the same sample for each strain. Repeated runs on residual digests, which were frozen at -80°C for two years prior to testing, showed excellent sample stability and reproducibility, and the current instrumentation gave much better sequence coverage for MS-H with less sample loading (Table 5).

**Table 5:** Diagnostic specificity, sample stability and run-to-run repeatability test results for MS-H with the Orbitrap platform^a^.

Strain number	Previously recorded serotypes with motility induction	MS-H types without motility induction	Sequence coverage (%)		
			Run 1	Run 2	Run 3
09-1336	NM	N/A	N/A	N/A	N/A
09-1337	NM	N/A	N/A	N/A	N/A
09-1338	NM	N/A	N/A	N/A	N/A
09-1339	NM	N/A	N/A	N/A	N/A
09-1345	NM	N/A	N/A	N/A	N/A
09-1346	NM	N/A	N/A	N/A	N/A
09-0411^b^	H11	H11	81	81	81
09-1342^b^	H11	H11	83	82	83
09-1344^b^	H11	H11	76	74	78
09-1765^b^	H11	H11	98	98	91
09-1767^b^	H11	H11	93	93	93
09-0409	H28	H28	70	79	75
09-0410	H28	H28	68	73	74
09-0416	H28	H28	76	77	78
09-0416	H28	H28	76	77	78
09-0412	H49	H49	88	89	89
09-0413	H16	H16	74	77	78
09-0415	H8	H8	98	91	90
09-1347	H19	H19	70	71	75
09-1348	H19	H19	67	69	68
09-1353	H25	H25	67	70	72
09-1354	H25	H25	64	74	78
09-1770	H19	H19	81	88	78
09-1774	H19	H19	88	88	88
09-1764	H19	H19	86	88	82
09-1766	H34	H34	59	59	59
09-1768	H14	H14	98	98	98
09-1769	H14	H14	98	98	98
09-1775	H25, then rough	H25	76	81	80

NM, non-motile; N/A, not attainable.

a The same flagella digest was tested by LC-MS/MS three times within a one-week period; ^b^a two-year old residual digest was re-used.

Detection limit tests were designed to determine the smallest amount of culture needed for flagella extraction, and the highest dilution of digested flagellin needed for MS-H. Reference strain 87-1215 (O157:H7) was used for this experiment. Colonies from the subculture of a single colony were counted after serial dilutions and the colony count average was used to calculate cell numbers within the single colony. The culture collection size of 2.16×10^14^ cells from 500 colonies was very similar to a full 10 µl loop size routinely used for flagella extraction. However, since the absolute amount of flagellin could not be quantified due to trypsin contamination during digestion, the fraction of the total digest was then used as the amount of sample loaded on to the nano-LC column. The test shows that 1/100 loopful of cell culture collection (i.e. 2×10^12^ cells from 5 colonies) could still give accurate identification for MS-H, and the use of 500 colonies gave the best sequence coverage for MS-H using only 1/160 of the flagellin digest (Table S4).

## Discussion

This study of LC-MS/MS-based method development and evaluation of *E. coli* H typing (MS-H) was based on international analytical method validation guidelines as they pertain to the characteristics of current *E. coli* serotyping for clinical diagnosis [16], [20]. The flagella purification assay was modified from traditional flagella purification procedures [13], but omitted tedious ultracentrifugation and gradient separation of the large volume of cell culture. Further, the process was specific for flagella due to their unique polymerized structure, size, and length [11]–[13]. The methodology not only made sample preparation faster and easier, but also minimized the presence of MS intolerable residues [14].

A 10 µl loopful of culture grown on TSA agar was sufficient to extract flagella on a 13 mm diameter filter for MS-H. Since flagella extraction and tryptic digestion were limited to a tiny, fixed space (roughly 80 µl) of syringe filter, more flagellin products relatively reduced the ratio of trypsin used in the digest, giving a much stronger flagellin to trypsin MS signal. Consequently, it is recommended to use an almost-full 10 µl loopful of fresh bacterial culture in order to achieve less noise-interference from trypsin auto-digestion. The QSTAR system gave valid results after loading half (i.e. 10 µl of 20 µl) of the re-dissolved flagellin preparation following vacuum drying of the digest. With the Orbitrap platform, accurate results were obtained with only 1/120 (i.e. 5 µl of 600 µl) of the digest without the need for vacuum drying. Additionally, the quantity of digested flagella was far beyond the need [15] for protein identification using this system, with more than 50% protein sequence coverage routinely obtained from a small fraction of the flagella digest on Orbitrap platform. This may be attributed to the purity of the flagella through such unique extraction and digestion methods, which also enabled the differentiation of H types with close sequence similarity. Sample analyses of LC-MS/MS with the two instrumentation platforms (QSTAR, Orbitrap) used in this study have proven that MS-H is reproducible and robust.

While embarking on database searches at the onset of this project, it was discovered that public databases such as NCBInr or Swiss-prot do not always display the necessary information needed for H antigen type investigation, and in some cases, there is no H type specified for the flagellin protein. Thus, a custom flagellin database was generated with the H type listed in the flagellin protein description. This curated database proved useful in obtaining correct MS-H types, and is available in the supporting information (Protein Database S1).

Table 6 summarizes the features of both MS-H and traditional serotyping. From this study, it can be concluded that the two methods possess similar diagnostic sensitivity and specificity [16], [20]. However, the peptide sequence-based MS-H method does appear to show some marked improvements over antisera-based serotyping. Serotyping must withstand many stringent conditions relative to MS-H, such as motility induction which can be time-consuming, and the quality of serological reagents. For instance, antisera characteristics play an important role in serotyping, and ultimately affect the overall capacity of the assay. The MS-H method does not routinely require motility induction of *E. coli*, and uses far fewer reagents besides not using antisera, both of which make MS-H more straightforward to perform and less time-consuming to finish. In addition, based on the observations through EM and SDS-PAGE that flagella production by *E. coli* may vary and the quantity of extracted flagella may differ between strains. Although this heterogeneity of flagella production and dynamics of motility were considered major factors affecting serotyping, the MS-H method proved to be more tolerant to these changes, albeit with a lower detection limit and higher sensitivity. MS-H can also be used for “sluggish” or inactive growing cultures, rough strains, and small volumes of culture as long as enough amounts of flagella can be extracted from the bacteria.

**Table 6:** Comparison of H serotyping and MS-H of *E. coli*.

Parameter	H Serotyping	MS-H
Diagnostic sensitivity	Could reach 100%	Could reach 100%
Diagnostic specificity	Could reach 100%	Could reach 100%
Analytical sensitivity	Loop size of culture	5 colonies
Analytical specificity	Antigenic epitope dependent	Ionization dependent
Read-out	Agglutination titer observation by eyes; process may require several steps	Protein and peptide sequences analyzed by software; one step identification
Motility induction	Routinely required	Not routinely required
Rapidity to get result	3 to 5 days	4 hr for a single sample
Ease of identifying rough strains	Impossible	Possible; motility induction can be used to obtain result
Result consistency	Motility induction and antisera dependent	Instrumentation and software dependent
Robustness and ruggedness	Limited; largely performed manually; may require optimal antigen/antisera reaction conditions	Good; largely performed by machine; can tolerate wide range of sample amounts and different instruments
Throughput	Limited, largely performed manually; not easily repeated	Good; LC-MS/MS can run day and night; easy to repeat and obtain a better result
Sample stability	Variation in bacteria growth	Protein digests are stable
Consumables and labor used	Antisera, culture media,Craigie tubes; Half day of labor	Trypsin, lysozyme, nano-LC columns; instrument service contract; MS routine runs, half hour of labor
System suitability/accessibility	Reference labs/institutions with antiserum or antibody production	Institutions or service labs with MS capability

In light of the many advantages in using the MS-H approach, factors influencing this method and the result should also be mentioned. These include differences between protein sequences of *E. coli* flagellin [21], genetic polymorphisms amongst the same type of H antigens [22]–[24], ionization differences amongst different peptides, and some unique technical features during LC-MS/MS (e.g. a millisecond level scanning speed for ion selection and down-stream fragmentation of ions on Orbitrap for peptides eluted by the nano-LC at the front end of the mass spectrometer). With these factors considered, the run-to-run peptide numbers detected and the related sequence coverage for protein identification may vary slightly, but H type identification would ultimately remain unaffected.

In conclusion, advantages of the MS-H method described in this study are primarily high specificity, sensitivity, accuracy, and reproducibility. The approach is rapid, simple, and reliable. MS-H can be used independently to type *E. coli* flagella without motility induction. In addition, by avoiding the traditional methods of motility induction and multi-step agglutination reactions, results are generated much faster with greater simplicity than antibody-based agglutination and/or primer-based PCR. Lastly, the MS-H method should be particularly useful during *E. coli* outbreak situations to provide presumptive H type classifications.

## Materials and Methods

### Bacterial strains and isolates

All the bacterial strains and isolates were from the ISO-certified national enteric reference center of National Microbiology Laboratory, Public Health Agency of Canada. The clinical isolates were originally from Alberta Provincial Laboratory for Public Health.

### Flagella purification and on-filter digestion

*E. coli* bacteria were grown at 37°C overnight on TSA plates with 5% sheep blood. A full loopful culture on a 10 micro-liter loop was diluted in 1 ml of water containing 2 mg of lysozyme and gently suspended using a pipette tip. The suspension was incubated at room temperature for 10 min. Then the sample was vortexed at a maximum speed on a vortex mixer (Vortex-Genie 2, VWR) for 20 sec each time with 1 min break after vortexing for a total of 3 cycles of vortexing. After centrifugation for 20 min at 16,000xg on a bench-top centrifuge (*eppendorf* 5417C), the supernatant was gently collected using a 1 ml syringe and passed through a 13 mm diameter filter with a 0.20 µm pore size (Acrodisc, PALL). The filter was washed with 3 ml of water and then flushed with air using a 1 ml syringe. 100 µl of trypsin (Promega mass spec grade, 100 µg per ml in 100 mM ammonium bicarbonate) was applied to the filter for digestion at 37°C for 2 hrs. The filter was flushed with 600 µl of water followed by air to collect the digest. For QSTAR MS detection, digests were dried down in a vacuum dryer and were reconstituted in 20 µl buffer A solution (0.1% formic acid used in nano-LC). For Orbitrap MS detection, 5 µl digests were directly mixed with 5 µl of 2x buffer A (0.2% formic acid) before loading.

Intact flagella were prepared by ultracentrifugation of the above supernatant at 50,000xg for 1 hr at 4°C [13] after lysozyme treatment and vortexing step shown above. The flagella pellet was then washed with 1 ml of cold PBS, spun down at 50,000xg for 1 hr at 4°C, and finally dissolved in 100 µl of 100 mM ammonium bicarbonate for protein quantification with a BCA kit (ThermoFisher). Trypsin was added to the purified flagella at a 1∶10 enzyme to protein ratio (in micrograms) for overnight digestion at 37°C and the digest was diluted with 2x buffer A for MS-H.

For side-by-side comparison of serotyping and MS-H, *E. coli* bacteria were grown from a single colony of culture from frozen stocks into two plates. One plate will be used for serotyping, and the other will be used for MS-H. For detection limit test of MS-H, cells from the single colony will be diluted in series with LB broth in triplicate, and the dilutions will be sub-cultured on TSA plates overnight at 37°C. The colonies will counted next day to convert to the cell numbers contained in the single colony used a day earlier. Certain numbers of colonies (5 to 500) will be picked for in-filter flagella extraction and tryptic digestion.

### LC-MS/MS

For the QSTAR LC-MS/MS system, the 600 µl tryptic digest was vacuum-dried and 20 µl of buffer A was added to the digest. After 15 min equilibration with buffer A, 10 µl of the sample was loaded on to a 0.3×5 mm C18 pre-column (Agilent) for pre-binding and the pre-column was washed with buffer A for 5 min. The pre-column was then automatically switched to connect to a nano-LC-column. Nano-LC (Agilent) separation was run at 300 nl/min on a 0.075×15 mm C18 nano-column (Agilent) with a 55 min acetonitrile gradient from 5 to 35 percent, followed by a 10 min flush with 95% acetonitrile before equilibration with buffer A. MS data was collected from a triple-quadrupole-time-of-flight mass spectrometer (QSTAR-XL, ABSciex) with an information-dependent acquisition (IDA) method. A one-second parent ion scan followed by three 3-second product ion scans (i.e. a scanning cycle) were used to collect the tandem mass spectra of the 3 strongest ions from each scanning cycle [25]. For the Orbitrap system, 5 µl of the 600 µl flagellin digest was mixed with 5 µl 0.2% formic acid and then loaded on to a 0.1×2 mm C18 pre-column (ThermoFisher) for binding after a 15 min equilibration time with buffer A. The pre-column was washed for 5 min with buffer A and switched to connect to a nano-LC column (ThermoFisher). Nano-LC (Proxeon EASY-nano-LC, ThermoFisher) separation was run at 300 nl/min on a 0.075×10 mm C18 nano-column with a 55 min acetonitrile gradient from 5 to 35 percent, followed by a 10 min flush with 95% acetonitrile before equilibration with buffer A. MS data was collected from an LTQ-Orbitrap XL system (ThermoFisher) with an IDA. One profile ion scan followed by 5 product ion scans (i.e. a scanning cycle) were used to collect the tandem mass spectra of the 5 strong ions from each scanning cycle [19].

### *E. coli* flagellin custom database creation and database search

A FASTA-formatted database for *E. coli* H types was created using the sequences and serotype information found in the NCBI protein database. Redundant sequences were collapsed into a single entry. The H type was listed in the sequence description. If no H type was specified in the NCBI database, the sequence was compared by BLASTp analysis against the sequences for which the H type was known, and the H type for the top blast result was used. In some cases the H-type was manually assigned (based on literature search) to sequences with missing H-type in NCBI, or assigned to sequences with incorrect H-type listed in the NCBI entry. Incorrect H-types were also discovered by finding outliers in a phylogenetic analysis of all *E. coli* flagellin sequences in the database. The final flagellin database had 196 protein sequence entries, and each entry contains a flagellin protein sequence of a specific H type (Fig. S1). The more common types, such as H7 and H11, have more entries (slightly different in amino acid composition due to some mutations) based on more studies on these types. Each entry has many theoretical tryptic peptides for protein identification and variable unique peptides to differentiate H types. This database was used to search the raw data in parallel with NCBInr using Mascot (Matrix Science) search engine. The search parameters of 0.3 Dalton mass error tolerance for parent ions and 0.8 Dalton mass error tolerance for product ions were chosen for QSTAR data [25]. For Orbitrap data, 30 ppm mass error tolerance for parent ions and 0.5 Dalton mass error tolerance for product ions were chosen [19]. In all cases, two missed cleavages of trypsin digestion were used. Oxidation on methionine and deamidation on glutamine and asparagines were chosen as possible modifications. The top Mascot scoring hit was used to decide the H type. If more than one H type was present in the top scoring hits, the result would be considered ambiguous. The protein database and all peptide data are available in the supporting information (Protein Database S1 and Representative Peptide Data S1, respectively).

### Electron microscopy

*E. coli* culture was gently mixed with fixative containing buffered 2% glutaraldehyde and 1% paraformaldehyde. The sample was then adsorbed to a glow discharged carbon-coated formvar film on a 400-mesh copper grid for 1 min, and negatively contrasted with 2% methylamine tungstate (Nano-W; Nanoprobes, Yaphank, NY, USA). Specimens were imaged in a FEI Tecnai 20 transmission electron microscope operating at 200 kV. Digital images of the specimens were acquired by an AMT Advantage XR 12 CCD camera (AMT, Danvers, MA, USA)

### *E. coli* H Serotyping

The *E. coli* H antigen was serotyped based on the methods of several publications [22], [26]-[28] summarized for our standard operation procedure. Basically, for motility induction, the bacteria were plated on MacConkey agar to check for purity and a single colony was selected. This colony was subcultured to a 0.25% Craigie tube and incubated overnight at 35°C ± 2°C. Motile *E. coli* bacteria should travel through the Craigie tube and up through the media using their flagella, while developing their H antigen. *E. coli* was then selected from the top of the media and transferred to a 0.3% Craigie tube to further develop motility after incubation overnight at 35°C ± 2°C. To prepare the H antigen, Ewing’s broth was added to the top of the 0.3% Craigie tube and gently drawn up and down so that the most motile bacteria originally at the surface of the Craigie tube became suspended fully into the Ewing’s broth. The suspension was incubated at 35°C ± 2°C for approximately 4 hours and treated with formalin to kill the live bacteria and preserve the H antigen. The H antigen was diluted and screened first in antisera pools prepared with 5 to 8 individual monovalent antisera. For any pool with a positive reaction, individual monovalent antisera were tested. Absorbed antisera were used for final confirmation of the H serotype for any occasional strains that cross-reacted with more than one monovalent antisera. All antisera had been previously titred with reference *E. coli* strains. A positive H serotype was obtained when the H antigen had an agglutination equivalent to or better than the reference titre for that antiserum. Serotyping synchronized with MS-H for comparison was done without motility induction, and was proceeded directly from a single colony subculture from frozen stocks with targeted antisera based on known H types through earlier serotyping and primary MS-H.

### Sequencing of *fliC* for H21

Oligonucleotides used for PCR based sequencing of *fliC* for H21 are listed in Table S2. DNA amplification was performed using Platinum High Fidelity *Taq* (Invitrogen) kit as per manufacturer’s instructions. The reaction mix included deoxynucleotide triphosphates at a final concentration of 200 mM and the oligos JHF2 and JHR2 at a concentration of 200 nM for H21 DNA amplification, together with the reaction buffer supplied from the kit. PCR conditions were: initial denaturation at 94 °C for 5 min, 30 cycles of denaturation at 94 °C for 30 sec, annealing at 55 °C for 30 sec and extension at 68 °C for 2 min, with a final extension at 68 °C for 5 min. PCR products were purified using Montage PCR spin columns (Millipore) and sequenced on an ABI 3730 (Applied Biosystems) using PCR primers (JHF2 and JFR2) and sequence specific primers (H21F3 and H21R3). Sequence data were analyzed using DNAStar Lasergene 7 (DNASTAR). The resulting consensus sequences were subjected to BLAST search to determine similarity to published sequences.

## Supporting Information

Figure S1 **Database of reference flagellin protein sequences and their known H antigen serogroups.** The X-axis represents the number of unique protein sequences obtained. The Y-axis represents all 53 known H type serogroups. The final flagellin database contained 196 sequences.
(DOCX)

Figure S2 **Electron microscopy images of** ***E. coli*** **flagella.** a. Reference *E. coli* strain E179 (H11) that lost flagella growth after long-time storage. b. Reference *E. coli* strain E 170 (H2) flagella. c. Clinical *E. coli* non-motil*e* isolate (09-1339) with no flagella. d. Clinical *E. coli* motil*e* isolate 09-1353 (H25) flagella.
(DOCX)

Figure S3 **SDS-PAGE of intact flagellin.** Coomassie blue staining of a 4-12% gradient SDS-PAGE gel showing the variable amounts of flagellin and purity of flagellin produced from a 10 µl loopful cell culture and extracted by ultracentrifugation from four *E. coli* strains representing different H types. 10 µl of the 100 µl extracted protein were loaded onto the SDS-PAGE gel. Strains used were: H7, 87-1215; H17: E185; H37: E205; H56: E376. A BCA kit was used to determine the total amount of flagellin extracted from each strain, which is labeled on the X-axis under each H type.
(DOCX)

Table S1 **Analytical sensitivity test for MS-H of purified flagellin tryptic digests on QSTAR platform.** Reference strain 87-1215 (O157:H7) was cultured overnight at 37°C and intact flagella were purified by ultracentrifugation as shown in Materials and Methods. The flagella were dissolved in 100 µl of 100 mM ammonium bicarbonate for protein quantitation with a BCA kit. Trypsin was added at a 1∶10 enzyme to protein ratio for overnight digestion at 37°C. The digest was diluted with 2x buffer A and designated amounts of the protein digest were loaded onto the LC-MS/MS system for MS-H.
(DOCX)

Table S2 **Primers used for H21 sequencing.**
(DOCX)

Table S3 **Real-time comparison of H serotyping and MS-H of flagella extracted from four selected** ***E.*** *coli* strains. Flagella were extracted from *E. coli* for LC-MS/MS in parallel with motility induction and serotyping independently on day 2, 7 and 16. MS-H was performed on the QSTAR system. Motility induced culture was used on day 16, but non-induced culture was used on day 2 and day 7.
(DOCX)

Table S4 **Analytical sensitivity check for** ***E. coli*** **strain 87-1215 (O157:H7) by MS-H with Orbitrap platform.** Cells from different numbers of colonies were used for flagella extraction. Note that more flagellar products relatively reduced the ratio of trypsin used for digestion, resulting in a relatively stronger flagellin peptide MS signal and a better chance for ion selection and fragmentation to obtain flagellin sequences.
(DOCX)

Protein Database S1 **The database comprises 196 flagellar protein sequences representing all 53 known serogroups.** The sequence data are presented in FASTA format with unique gi numbers. The database is updated annually with any new entries.
(DOCX)

Representative Peptide Data S1 **Peptide data are represented as the Mascot search results from all 53 serotypes, obtained under the Orbitrap platform in** **Table 4** **with related** ***E. coli*** **reference strains.** “U” denotes a unique peptide specific for each of the proteins 1.1, 1.2, and beyond. The number 1.1 (shown as 1 in the peptide list and phylogenetic tree) represents the protein which obtained the highest score and confidence value after a Mascot search. This protein, known as the first hit, was used to designate the MS-H type of the unknown flagellin. Related peptides 1.2 (2), 1.3 (3), etc. represented the second, third, etc. hits for MS-H typing analysis.
(DOCX)
