# Supplementary material for: MS-H: A Novel Proteomic Approach to Isolate and Type the E. coli H Antigen Using Membrane Filtration and Liquid Chromatography-Tandem Mass Spectrometry (LC-MS/MS)
Source: PLoS One. 2013 Feb 21;8(2):e57339. doi: 10.1371/journal.pone.0057339 (PMC3578835; doi:10.1371/journal.pone.0057339)
Supplement: Figure S1 — Database of reference flagellin protein sequences and their known H antigen serogroups. The X-axis represents the number of unique protein sequences obtained. The Y-axis represents all 53 known H type serogroups. The final flagellin database contained 196 sequences. (DOCX) [file pone.0057339.s001.docx]

**Figure S1.** Database of reference flagellin protein sequences and their known H antigen serogroups^a^

^a^The X-axis represents the number of unique protein sequences obtained. The Y-axis represents all 53 known H type serogroups. The final flagellin database contained 196 sequences.
