# Supplementary material for: MS-H: A Novel Proteomic Approach to Isolate and Type the E. coli H Antigen Using Membrane Filtration and Liquid Chromatography-Tandem Mass Spectrometry (LC-MS/MS)
Source: PLoS One. 2013 Feb 21;8(2):e57339. doi: 10.1371/journal.pone.0057339 (PMC3578835; doi:10.1371/journal.pone.0057339)
Supplement: Figure S2 — Electron microscopy images of E. coli flagella. a. Reference E. coli strain E179 (H11) that lost flagella growth after long-time storage. b. Reference E. coli strain E 170 (H2) flagella. c. Clinical E. coli non-motile isolate (09-1339) with no flagella. d. Clinical E. coli motile isolate 09-1353 (H25) flagella. (DOCX) [file pone.0057339.s002.docx]

**Figure S2.** Electron microscopy images of *E. coli* flagella
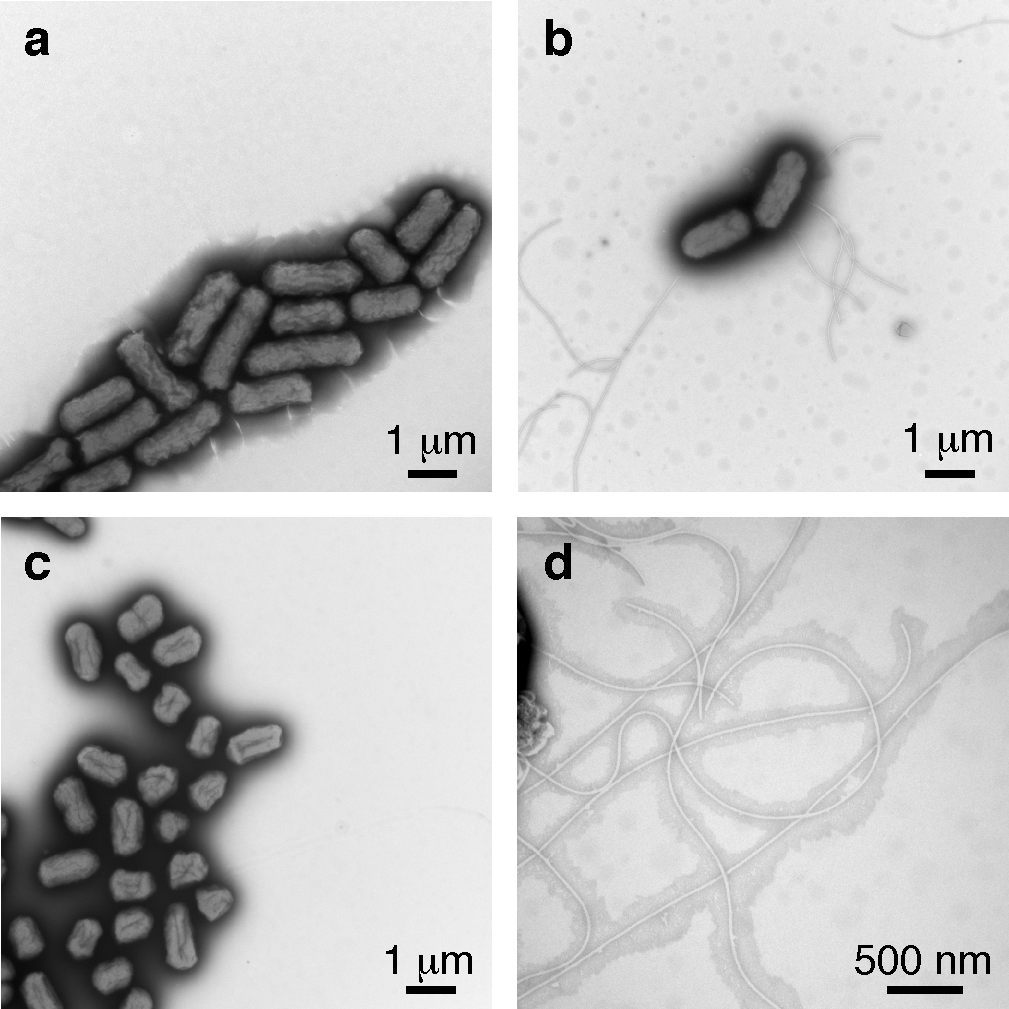


a. Reference *E. coli* strain E179 (H11) that lost flagella growth after long-time storage.

b. Reference *E. coli* strain E 170 (H2) flagella.

c. Clinical *E. coli* non-motil*e* isolate (09-1339) with no flagella.

d. Clinical *E. coli* motil*e* isolate 09-1353 (H25) flagella.
