# Supplementary material for: MS-H: A Novel Proteomic Approach to Isolate and Type the E. coli H Antigen Using Membrane Filtration and Liquid Chromatography-Tandem Mass Spectrometry (LC-MS/MS)
Source: PLoS One. 2013 Feb 21;8(2):e57339. doi: 10.1371/journal.pone.0057339 (PMC3578835; doi:10.1371/journal.pone.0057339)
Supplement: Figure S3 — SDS-PAGE of intact flagellin. Coomassie blue staining of a 4-12% gradient SDS-PAGE gel showing the variable amounts of flagellin and purity of flagellin produced from a 10 µl loopful cell culture and extracted by ultracentrifugation from four E. coli strains representing different H types. 10 µl of the 100 µl extracted protein were loaded onto the SDS-PAGE gel. Strains used were: H7, 87-1215; H17: E185; H37: E205; H56: E376. A BCA kit was used to determine the total amount of flagellin extracted from each strain, which is labeled on the X-axis under each H type. (DOCX) [file pone.0057339.s003.docx]

**Figure S3.** SDS-PAGE of intact flagellin

M, molecular weight marker (Invitrogen).

Coomassie blue staining of a 4-12% gradient SDS-PAGE gel showing the variable amounts of flagellin and purity of flagellin produced from a 10 µl loopful cell culture and extracted by ultracentrifugation from four *E. coli* strains representing different H types. 10 µl of the 100 µl extracted protein were loaded onto the SDS-PAGE gel. Strains used were: H7, 87-1215; H17: E185; H37: E205; H56: E376. A BCA kit was used to determine the total amount of flagellin extracted from each strain, which is labeled on the X-axis under each H type.
