# Supplementary material for: MS-H: A Novel Proteomic Approach to Isolate and Type the E. coli H Antigen Using Membrane Filtration and Liquid Chromatography-Tandem Mass Spectrometry (LC-MS/MS)
Source: PLoS One. 2013 Feb 21;8(2):e57339. doi: 10.1371/journal.pone.0057339 (PMC3578835; doi:10.1371/journal.pone.0057339)
Supplement: Table S1 — Analytical sensitivity test for MS-H of purified flagellin tryptic digests on QSTAR platform. Reference strain 87-1215 (O157:H7) was cultured overnight at 37°C and intact flagella were purified by ultracentrifugation as shown in Materials and Methods. The flagella were dissolved in 100 µl of 100 mM ammonium bicarbonate for protein quantitation with a BCA kit. Trypsin was added at a 1∶10 enzyme to protein ratio for overnight digestion at 37°C. The digest was diluted with 2x buffer A and designated amounts of the protein digest were loaded onto the LC-MS/MS system for MS-H. (DOCX) [file pone.0057339.s004.docx]

**Table S1.** Analytical sensitivity test for MS-H of purified flagellin tryptic digests on QSTAR platform^a^

| Amount of flagellin digest used for MS-H | Percentage of sequence  coverage (%) |
| --- | --- |
| 0.15 µg | 60 |
| 1.5 µg | 68 |
| 3.0 µg | 72 |
| 7.5 µg | 88 |

^a^Reference strain 87-1215 (O157:H7) was cultured overnight at 37^o^C and intact flagella were purified by ultracentrifugation as shown in Materials and Methods. The flagella were dissolved in 100 µl of 100 mM ammonium bicarbonate for protein quantitation with a BCA kit. Trypsin was added at a 1:10 enzyme to protein ratio for overnight digestion at 37^o^C. The digest was diluted with 2x buffer A and designated amounts of the protein digest were loaded onto the LC-MS/MS system for MS-H.
