# Supplementary material for: MS-H: A Novel Proteomic Approach to Isolate and Type the E. coli H Antigen Using Membrane Filtration and Liquid Chromatography-Tandem Mass Spectrometry (LC-MS/MS)
Source: PLoS One. 2013 Feb 21;8(2):e57339. doi: 10.1371/journal.pone.0057339 (PMC3578835; doi:10.1371/journal.pone.0057339)
Supplement: Table S2 — Primers used for H21 sequencing. (DOCX) [file pone.0057339.s005.docx]

**Table S2.** Primers used for H21 Sequencing

| **Oligonucleotide** | **Sequence (5’-3’)** | **Application** |
| --- | --- | --- |
| JHF2 | CGGGCCAGGCGATTGCTAACCGCT | PCR/sequencing |
| JHR2 | GCATGATTATCCGTTTCTGCAG | PCR/sequencing |
| H21F3 | acaggttgtttacggtgttg | Sequencing for H21 |
| H21R3 | tccaggctgaaattactcaa | sequencing for H21 |
