# Supplementary material for: MS-H: A Novel Proteomic Approach to Isolate and Type the E. coli H Antigen Using Membrane Filtration and Liquid Chromatography-Tandem Mass Spectrometry (LC-MS/MS)
Source: PLoS One. 2013 Feb 21;8(2):e57339. doi: 10.1371/journal.pone.0057339 (PMC3578835; doi:10.1371/journal.pone.0057339)
Supplement: Table S3 — Real-time comparison of H serotyping and MS-H of flagella extracted from four selected E. coli strains. Flagella were extracted from E. coli for LC-MS/MS in parallel with motility induction and serotyping independently on day 2, 7 and 16. MS-H was performed on the QSTAR system. Motility induced culture was used on day 16, but non-induced culture was used on day 2 and day 7. (DOCX) [file pone.0057339.s006.docx]

**Table S3.** Real-time comparison of H serotyping and MS-H of flagellar extracted from four selected *E. coli* strains^a^

| Strains with recorded  serotype | Motility induction | | | Serotyping | | | MS-H | | |
| --- | --- | --- | --- | --- | --- | --- | --- | --- | --- |
|  | Days | | | Days | | | Days | | |
|  | 2 | 7 | 16 | 2 | 7 | 16 | 2 | 7 | 16* |
|  |  |  |  |  |  |  |  |  |  |
| EDL933 (H7) | M | M | M | H7 | H7 | H7 | H7 | H7 | H7 |
| 87-1215 (H7) | M | M | M | - | H7 | H7 | H7 | H7 | H7 |
| 90-2380 (H7) | M | M | M | - | H7 | H7 | H7 | H7 | H7 |
| E-375 (H55) | NM | M | M | - | rough | rough | UI | UI | H55 |

-, serotyping titration not reached. UI,unidentifiable; M, motile; NM, non-motile.

^a^Flagella were extracted from *E. coli* for LC-MS/MS in parallel with motility induction and serotyping independently on day 2,7, and 16. MS-H was performed on the QSTAR system. *Motility induced culture was used on day 16, but non-induced culture was used on day 2 and day 7.
