# Supplementary material for: MS-H: A Novel Proteomic Approach to Isolate and Type the E. coli H Antigen Using Membrane Filtration and Liquid Chromatography-Tandem Mass Spectrometry (LC-MS/MS)
Source: PLoS One. 2013 Feb 21;8(2):e57339. doi: 10.1371/journal.pone.0057339 (PMC3578835; doi:10.1371/journal.pone.0057339)
Supplement: Table S4 — Analytical sensitivity check for E. coli strain 87-1215 (O157:H7) by MS-H with Orbitrap platform. Cells from different numbers of colonies were used for flagella extraction. Note that more flagellar products relatively reduced the ratio of trypsin used for digestion, resulting in a relatively stronger flagellin peptide MS signal and a better chance for ion selection and fragmentation to obtain flagellin sequences. (DOCX) [file pone.0057339.s007.docx]

**Table S4.** Analytical sensitivity check for *E. coli* strain 87-1215 (O157:H7) by MS-H with Orbitrap platform^a^

| **Cultured cells used for flagella extraction and the estimate of related cell numbers** | **Loaded amount of flagella digests (all 10 µl in buffer A)** | **Peptide number / sequence coverage (%) of H7 by Mascot search** |
| --- | --- | --- |
| 5 colonies  (2.16x10^12^cells) | Whole digest after vacuum drying | 10 / 20% |
| 50 colonies  (2.16x10^13^ cells) | 1/5 of whole digest after vacuum drying | 15 / 38% |
| 500 colonies*  (2.16x10^14^ cells) | 1/160 of 600 µl whole digest without vacuum drying | 43 / 80% |

^a^Cells from different numbers of colonies were used for flagella extraction.

*Note that more flagellar products relatively reduced the ratio of trypsin used for digestion, resulting in a relatively stronger flagellin peptide MS signal and a better chance for ion selection and fragmentation to obtain flagellin sequences.
