# Supplementary material for: MS-H: A Novel Proteomic Approach to Isolate and Type the E. coli H Antigen Using Membrane Filtration and Liquid Chromatography-Tandem Mass Spectrometry (LC-MS/MS)
Source: PLoS One. 2013 Feb 21;8(2):e57339. doi: 10.1371/journal.pone.0057339 (PMC3578835; doi:10.1371/journal.pone.0057339)
Supplement: Representative Peptide Data S1 — Peptide data are represented as the Mascot search results from all 53 serotypes, obtained under the Orbitrap platform in Table 4 with related E. coli reference strains. “U” denotes a unique peptide specific for each of the proteins 1.1, 1.2, and beyond. The number 1.1 (shown as 1 in the peptide list and phylogenetic tree) represents the protein which obtained the highest score and confidence value after a Mascot search. This protein, known as the first hit, was used to designate the MS-H type of the unknown flagellin. Related peptides 1.2 (2), 1.3 (3), etc. represented the second, third, etc. hits for MS-H typing analysis. (DOCX) [file pone.0057339.s009.docx › H44-E212.pdf]

MATRIX SCIENCE MASCOT Search Results

User :
E-mail :
Search title : Submitted from 20110822-0608 by Mascot Daemon on VARIABLE
MS data file : C:\Documents and Settings\keding\Desktop\Raw data\20110822-002-0031-00608\20110822-003-EC212MS3.RAW
Database : Flagellin_v2 (192 sequences; 89,845 residues)
Taxonomy : Bacteria (Eubacteria) (192 sequences)
Timestamp : 24 Aug 2011 at 17:06:49 GMT

Not what you expected? Try **the select summary**.

▶ **Search parameters**

▶ **Score distribution**

▶ **Legend**

Protein Family Summary

Significance threshold p< 0.05 Max. number of families AUTO
Ions score or expect cut-off 0 Dendrograms cut at 0

Protein family 1 (out of 1)

10 per page 1

▼1

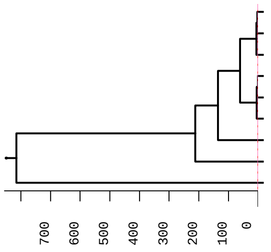

1	gi|125659369	3325	H44 0|H44|781-55
6	gi|30059920	1144	H46 0|H46|5306-56
9	gi|16901494	423	H27 0|H27|0
3	gi|190901652	1241	H28 0|O148:H28|B7A [gi|191168280|ref|ZP_03030073| H28 0|O148:H28|B7A] [gi|209919345|r...
4	gi|30059912	1190	H41 0|H41|RVC 1787
8	gi|330911731	798	Hxx(H4 99.4%) 0|0|AA86
5	gi|192955539	1154	H9 0|O111:H9|E110019 [gi|193071144|ref|ZP_03052068| H9 0|O111:H9|E110019]
7	gi|121144335	1040	H55 0|O75:H55|E2987-73
2	gi|6941886	2315	H43 0|O157:H43|O157:H43 [gi|30059916|ref|AAP13330| H43 0|H43|149-51]

Threshold (**0**): 0

		Score	Mass	Matches	Sequences	emPAI
1.1	gi|125659369	3325	58912	77 (63)	40 (37)	14.11
	H44 0|H44|781-55					
1.2	gi|6941886	2315	51071	46 (36)	25 (20)	3.78
	H43 0|O157:H43|O157:H43 [gi|30059916|ref|AAP13330| H43 0|H43|149-51]					
1.3	gi|190901652	1241	59358	41 (28)	23 (18)	2.65
	H28 0|O148:H28|B7A [gi|191168280|ref|ZP_03030073| H28 0|O148:H28|B7A] [gi|209919345|ref|YP_002293429| Hxx 0|0|SE11] [gi|209912604|ref|BAG77678| Hxx 0|0|SE11]					
	▶2 samesets of gi|190901652					
1.4	gi|30059912	1190	57346	38 (27)	20 (17)	2.61
	H41 0|H41|RVC 1787					
	▶1 sameset of gi|30059912					
1.5	gi|192955539	1154	68106	36 (23)	21 (15)	1.69
	H9 0|O111:H9|E110019 [gi|193071144|ref|ZP_03052068| H9 0|O111:H9|E110019]					
	▶5 samesets of gi|192955539					
1.6	gi|30059920	1144	57918	35 (24)	19 (16)	2.19
	H46 0|H46|5306-56					
	▶1 sameset of gi|30059920					
1.7	gi|121144335	1040	62285	33 (23)	18 (15)	1.80
	H55 0|O75:H55|E2987-73					
1.8	gi|330911731	798	36283	26 (18)	15 (13)	3.42
	Hxx(H4 99.4%) 0|0|AA86					
	▶2 samesets of gi|330911731					
1.9	gi|16901494	423	50823	15 (8)	10 (5)	0.55
	H27 0|H27|0					
	▶2 samesets of gi|16901494					

▼**128 peptide matches (91 non-duplicate, 37 duplicate)**

Query	Dupes	Observed	Mr(expt)	Mr(calc)	Delta	M	Score	Expect	Rank	U	1	2	3	4	5	6	7	8	9	Peptide
26	▶1	316.6906	631.3666	631.3653	0.0013	0	29	0.013	▶1		■	■	■	■	■	■	■	■	■	R.LSSGLR.I
52	▶1	338.6935	675.3724	674.3963	0.9761	0	19	0.013	▶1	U									■	K.TVTGLGK.T
86		355.1985	708.3824	708.3806	0.0018	0	18	0.094	▶1		■	■	■	■	■	■	■	■		R.FTSNIK.G
90	▶1	358.7072	715.3998	715.3977	0.0022	0	31	0.0062	▶1		■	■	■	■	■	■	■	■		K.GLTQAAR.N
126	▶1	380.6963	759.3780	759.3763	0.0018	0	29	0.0079	▶1		■		■	■	■	■	■	■		R.LDEIDR.V
142		386.7327	771.4508	771.4490	0.0018	0	15	0.033	▶1	U								■		K.ALDAAIAK.V
258		427.7169	853.4192	853.4182	0.0011	0	31	0.00079	▶1		■					■				K.FTTDAATK.A
301		444.2461	886.4776	886.4760	0.0017	0	47	2.1e-05	▶1		■		■							K.AATTADPLK.A
314	▶1	450.2816	898.5486	898.5488	-0.0001	0	54	3.7e-06	▶1	U	■									K.LQSILTPK.A
324		452.7038	903.3930	902.5073	0.8858	0	0	1.4	▶1	U			■							K.AATLDALTK.N
370	▶1	466.2519	930.4892	930.4883	0.0010	0	75	1.3e-07	▶1		■	■	■	■	■		■	■		R.SSLGAVQNR.L
506		496.7362	991.4578	991.4570	0.0008	0	26	0.0024	▶1	U	■									K.DGSITNNSGK.A

Query	Dupes	Observed	Mr(expt)	Mr(calc)	Delta	M	Score	Expect	Rank	U	1	2	3	4	5	6	7	8	9	Peptide
525	1	502.2619	1002.5092	1002.5094	-0.0002	1	39	0.00074	1		■		■	■	■	■	■	■		K.SRLDEIDR.V
526		335.1781	1002.5125	1002.5094	0.0031	1	34	0.0021	1		■		■	■	■	■	■	■		K.SRLDEIDR.V
538		504.7541	1007.4936	1007.4924	0.0013	0	56	2.8e-06	1	U	■									K.AVYVQEDGK.F
538		504.7541	1007.4936	1007.4771	0.0165	0	2	0.58	2	U							■			K.DGTITTTDGK.S
577		513.2719	1024.5292	1024.5302	-0.0009	0	81	7.6e-09	1	U	■									K.AGDTAHLNVK.I
578		342.5182	1024.5328	1024.5302	0.0026	0	30	0.00099	1	U	■									K.AGDTAHLNVK.I
639		350.8743	1049.6011	1048.5036	1.0974	0	13	0.049	1	U				■						K.AADGSLTSEAK.G
679	1	537.7983	1073.5820	1073.5717	0.0104	0	15	0.032	1	U						■				R.ISADALQSAAK.G
738		551.2686	1100.5226	1100.5210	0.0016	0	67	1.7e-06	1		■	■	■	■	■	■	■	■	■	K.DDAAGQAIANR.F
774		561.2965	1120.5784	1120.5764	0.0020	0	54	4.3e-06	1	U	■									K.NETEIFLQK.D
806	1	568.7850	1135.5554	1135.5543	0.0012	0	91	1.2e-09	1	U	■									R.ISAEAMQSATK.T
824		574.2965	1146.5784	1146.5768	0.0016	0	74	4.3e-08	1	U	■									K.ALDDAISSIDK.F
836		576.7823	1151.5500	1151.5492	0.0008	0	83	5.6e-09	1	U	■									R.ISAEAMQSATK.T + Oxidation (M)
836		576.7823	1151.5500	1151.5492	0.0008	0	31	0.00081	2	U			■							R.MSAESLQSATK.S
868		390.1813	1167.5221	1166.5819	0.9401	0	2	0.66	1	U									■	K.DVTFTIDATGK.D
876		587.2974	1172.5802	1172.6765	-0.0962	1	3	0.54	1	U									■	K.AAVSLAKDASIK.Y
968	1	612.2886	1222.5626	1222.5612	0.0015	0	63	5.5e-07	1		■						■			K.NQSSMSTAIER.L
980		615.8203	1229.6260	1228.6412	0.9849	0	8	0.18	1	U		■								K.QAGAAPGTALTSGK.V
1000		620.2858	1238.5570	1238.5561	0.0010	0	63	5e-07	1		■						■			K.NQSSMSTAIER.L + Oxidation (M)
1017		415.8528	1244.5366	1244.6361	-0.0995	1	3	0.48	1	U					■					K.EQKVNISQDGK.I
1019		623.8353	1245.6560	1245.6565	-0.0004	0	86	2.8e-09	1	U		■								K.TALAAAGADTSGLK.L
1045		631.8101	1261.6056	1261.6038	0.0019	0	65	3.2e-07	1	U	■									K.SDLDSIQDEIK.S
1118		651.8625	1301.7104	1302.6415	-0.9311	0	5	0.69	1	U									■	K.AATASDLDLNNAK.K
1180		672.8793	1343.7440	1343.7408	0.0032	0	69	1.3e-07	1	U							■			-.SLSLITQNNINK.N
1189	1	676.3396	1350.6646	1350.6627	0.0020	0	82	6.2e-09	1	U	■									R.ELTVQSSTGTNSK.S
1296		716.3572	1430.6998	1430.7365	-0.0366	1	1	1.4	1	U									■	K.AATASDLDLNNAKK.V
1307	1	720.9132	1439.8118	1439.8096	0.0022	0	104	2e-10	1		■		■	■	■	■	■			K.AQIIQQAGNSVLAK.A
1319	4	724.3867	1446.7588	1446.7566	0.0022	0	123	1.1e-12	1	U	■									K.IGATSVDVVLSSDGK.I
1326		484.2568	1449.7486	1449.7463	0.0022	1	35	0.00029	1	U	■									K.ALDDAISSIDKFR.S
1327		725.8817	1449.7488	1449.7463	0.0025	1	78	1.5e-08	1	U	■									K.ALDDAISSIDKFR.S
1341		729.5184	1457.0222	1455.8045	1.2177	0	18	0.027	1			■						■		K.AQIIQQAGNSVLSK.A
1394		747.9189	1493.8232	1493.8202	0.0031	0	40	0.00058	1		■	■	■	■	■	■		■		K.ANQVPQQVLSLLQG.-
1484	2	781.4214	1560.8282	1560.8260	0.0022	0	75	1.4e-07	1		■	■	■	■		■	■			R.VSGQTQFNGVNVLAK.D
1552		538.9450	1613.8132	1613.8121	0.0011	1	38	0.0012	1		■	■	■	■	■	■	■	■	■	R.INSAKDDAAGQAIANR.F
1553		807.9143	1613.8140	1613.8121	0.0019	1	86	2.2e-08	1		■	■	■	■	■	■	■	■	■	R.INSAKDDAAGQAIANR.F
1563		540.9573	1619.8501	1619.8479	0.0022	1	33	0.00053	1	U	■									R.IRELTVQSSTGTNSK.S
1571		814.9124	1627.8102	1627.8054	0.0049	0	108	1.4e-11	1	U	■									K.TTGFTTGTTTVAANTGK.V
1591		820.9243	1639.8340	1639.8305	0.0035	0	97	1.9e-10	1	U		■								K.IDSSTLGLTGFDVSTK.A
1634	1	836.3815	1670.7484	1670.7457	0.0027	0	125	1.8e-12	1		■	■	■	■	■	■	■	■		R.IQDADYATEVSNMSK.A
1636	3	836.4511	1670.8876	1670.8839	0.0037	0	123	3.1e-12	1	U		■								K.IQVGANDGQTISIDLK.K
1661	1	843.4611	1684.9076	1684.8996	0.0081	0	91	3e-09	1		■		■	■		■				K.IQVGANDGQTITIDLK.K
1661		843.4611	1684.9076	1685.8836	-0.9759	0	33	0.0022	2						■		■			K.IQVGANDGETITIDLK.K
1679	1	849.9796	1697.9446	1697.9424	0.0022	0	100	1e-10	1	U	■									K.LAGATVAGQSGAIVVTGAR.I
1680		566.9889	1697.9449	1697.9424	0.0024	0	46	2.5e-05	1	U	■									K.LAGATVAGQSGAIVVTGAR.I
1694	1	854.9048	1707.7950	1707.7912	0.0039	0	137	2e-14	1	U		■								K.DASGNSTTAAVTLGGSDGK.T
1695		570.5724	1708.6954	1707.7912	0.9042	0	2	0.69	1	U		■								K.DASGNSTTAAVTLGGSDGK.T
1719		574.9578	1721.8516	1721.8836	-0.0320	1	1	0.82	2	U	■									K.FTTDAATKAATTADPLK.A
1721		431.4724	1721.8605	1721.8836	-0.0231	1	4	0.39	1	U	■									K.FTTDAATKAATTADPLK.A
1784		884.9711	1767.9276	1767.9255	0.0022	1	117	1.9e-12	1	U		■								K.KIDSSTLGLTGFDVSTK.A
1785		590.3165	1767.9277	1767.9255	0.0022	1	43	5e-05	1	U		■								K.KIDSSTLGLTGFDVSTK.A
1826		359.9980	1794.9536	1795.8589	-0.9052	0	3	0.51	2	U			■							K.STGFTVDVGATGNSAGDIK.V
1842		902.9438	1803.8730	1803.9438	-0.0708	1	2	3.6	1			■	■	■	■	■				K.NQSALSSSIERLSSGLR.I
1964		954.9873	1907.9600	1907.9589	0.0012	0	113	5.1e-12	1	U	■									K.VTIGGNQAYTQTDGTLAAK.N
1965		636.9947	1907.9623	1907.9589	0.0034	0	39	0.00011	1	U	■									K.VTIGGNQAYTQTDGTLAAK.N
2069		672.6907	2015.0503	2015.0422	0.0080	1	33	0.0005	1	U	■									K.AATTADPLKALDDAISSIDK.F
2107	1	1043.0700	2084.1254	2084.1225	0.0029	0	134	2.7e-13	1		■	■	■	■	■	■		■	■	M.AQVINTNSLSLiTQNNiNK.N
2108		695.7158	2084.1256	2084.1225	0.0030	0	65	2.1e-06	1		■	■	■	■	■	■		■	■	M.AQVINTNSLSLiTQNNiNK.N
2185		739.7271	2216.1595	2215.1630	0.9964	0	0	6.2	2		■	■	■	■	■	■		■	■	-.MAQVINTNSLSLiTQNNiNK.N
2187		1110.7910	2219.5674	2220.0944	-0.5269	1	1	4.2	1						■		■			K.DGSMKIQVGANDGETITIDLK.K + Oxidation (M)
2203	1	1119.0580	2236.1014	2236.0972	0.0043	0	103	1.2e-10	1	U	■									K.IDSSTLNLTGFNVNGEGSVANK.A
2214		1125.0570	2248.0994	2248.0931	0.0063	0	124	2.3e-12	1		■	■	■	■	■	■	■	■		R.LDSAVTNLNNTTTNLSEAQSR.I
2215		750.3741	2248.1005	2248.0931	0.0074	0	60	6.6e-06	1		■	■	■	■	■	■	■	■		R.LDSAVTNLNNTTTNLSEAQSR.I
2254		773.0783	2316.2131	2316.2074	0.0057	1	56	2.7e-06	1	U		■								R.LEEIDRVSGQTQFNGVNVLAK.D
2265		789.0731	2364.1975	2365.0744	-0.8769	0	2	0.67	2	U					■					K.SNFTIDMGGTGSVTYTVSNGDVK.A + Oxidation (M)
2266		1183.1070	2364.1994	2364.1921	0.0073	1	107	1.8e-11	1	U	■									K.KIDSSTLNLTGFNVNGEGSVANK.A
2267	1	789.4016	2365.1830	2364.1921	0.9909	1	64	4.4e-07	1	U	■									K.KIDSSTLNLTGFNVNGEGSVANK.A
2306		856.0864	2565.2374	2565.2293	0.0080	0	57	6e-06	1	U		■								R.ELTVQATTGTNSESDLSSIQDEIK.S
2306		856.0864	2565.2374	2565.2294	0.0080	0	33	0.0015	2	U					■					R.ELTVQATTGTNSTSDLDSIQDEIK.S
2306		856.0864	2565.2374	2565.1930	0.0444	0	22	0.02	3				■	■						R.ELTVQASTGTNSDSDLDSIQDEIK.S
2307	1	1283.6260	2565.2374	2565.1930	0.0445	0	19	0.035	3				■	■						R.ELTVQASTGTNSDSDLDSIQDEIK.S
2308	1	1283.6290	2565.2434	2565.2293	0.0141	0	153	1.5e-15	1	U		■								R.ELTVQATTGTNSESDLSSIQDEIK.S
2308	1	1283.6290	2565.2434	2565.2294	0.0141	0	75	9.3e-08	2	U					■					R.ELTVQATTGTNSTSDLDSIQDEIK.S
2319		877.1000	2628.2782	2628.2739	0.0043	0	50	4.9e-05	1		■		■	■	■	■	■			R.NANDGISVAQTTEGALSEINNNLQR
2320		1315.1500	2628.2854	2628.2739	0.0115	0	131	3.7e-13	1		■		■	■	■	■	■			R.NANDGISVAQTTEGALSEINNNLQR
2325	1	881.4609	2641.3609	2642.2896	-0.9287	0	3	0.78	2	U		■								R.NANDGISLAQTTEGALSEINNNLQR.V
2326	1	881.4612	2641.3618	2641.3559	0.0059	0	120	1.5e-12	1	U	■									K.ADLTAAQLTTTAAGGTIAAPAADANGVTK.Y
2327		1321.6890	2641.3634	2641.3559	0.0076	0	127	3.2e-13	1	U	■									K.ADLTAAQLTTTAAGGTIAAPAADANGVTK.Y
2461	2	1211.2370	3630.6892	3630.6750	0.0141	0	101	8.3e-11	1	U		■								K.YYAQITSAANPGLDGAYEIHVNDADGSFTVAASDK.Q
2468	1	1252.9360	3755.7862	3755.7691	0.0171	0	71	8.2e-08	1	U		■								K.VTNVGYGLQNDSGTIFATDYDGTTVTTPGAETVTYK.D

57 subsets and intersections (149 subset proteins in total)

10 per page 1

Mascot: http://www.matrixscience.com/
