# Supplementary material for: MS-H: A Novel Proteomic Approach to Isolate and Type the E. coli H Antigen Using Membrane Filtration and Liquid Chromatography-Tandem Mass Spectrometry (LC-MS/MS)
Source: PLoS One. 2013 Feb 21;8(2):e57339. doi: 10.1371/journal.pone.0057339 (PMC3578835; doi:10.1371/journal.pone.0057339)
Supplement: Representative Peptide Data S1 — Peptide data are represented as the Mascot search results from all 53 serotypes, obtained under the Orbitrap platform in Table 4 with related E. coli reference strains. “U” denotes a unique peptide specific for each of the proteins 1.1, 1.2, and beyond. The number 1.1 (shown as 1 in the peptide list and phylogenetic tree) represents the protein which obtained the highest score and confidence value after a Mascot search. This protein, known as the first hit, was used to designate the MS-H type of the unknown flagellin. Related peptides 1.2 (2), 1.3 (3), etc. represented the second, third, etc. hits for MS-H typing analysis. (DOCX) [file pone.0057339.s009.docx › H45-E213.pdf]

MATRIX SCIENCE MASCOT Search Results

User :
E-mail :
Search title : Submitted from 20110822-0611 by Mascot Daemon on VARIABLE
MS data file : C:\Documents and Settings\keding\Desktop\Raw data\20110823-001-0031-00611\20110823-006-EC213MS2-RP.RAW
Database : Flagellin_v2 (192 sequences; 89,845 residues)
Taxonomy : Bacteria (Eubacteria) (192 sequences)
Timestamp : 24 Aug 2011 at 18:07:08 GMT

Not what you expected? Try **the select summary**.

▶ **Search parameters**
▶ **Score distribution**
▶ **Legend**

Protein Family Summary

Significance threshold p<	0.05	Max. number of families	AUTO
Ions score or expect cut-off	0	Dendrograms cut at	0

Protein families 1–2 (out of 2)

10 per page 1

▼**1**

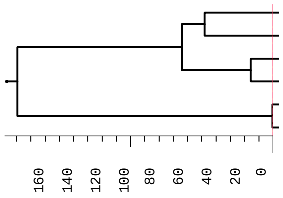

1	gi|6009843	1410	H45 0|0|4106-54
6	gi|112820171	249	H21 0|EHEC serogroup: O113:H21|0 [gi|291323135|ref|EFE62563| Hxx 0|0|B088] [gi...
2	gi|125659376	958	H55 0|H55|E2987-73
5	gi|33590243	483	H16 NM|O157:NM(H16)|PC02
3	gi|125659364	600	H44 0|H44|E2987-73
4	gi|30059910	572	H39 0|H39|E3a

Threshold (**0**): 0

		Score	Mass	Matches	Sequences	emPAI
☑ **1.1**	**gi|6009843**<br>H45 0|0|4106-54<br>▶**1 sameset of gi|6009843**	1410	57810	31 (24)	24 (21)	2.77
☑ **1.2**	**gi|125659376**<br>H55 0|H55|E2987-73	958	63681	26 (18)	19 (15)	1.47
☑ **1.3**	**gi|125659364**<br>H44 0|H44|E2987-73<br>▶**2 samesets of gi|125659364**	600	46286	21 (13)	16 (12)	1.45
☑ **1.4**	**gi|30059910**<br>H39 0|H39|E3a	572	44239	22 (13)	16 (12)	1.55
☑ **1.5**	**gi|33590243**<br>H16 NM|O157:NM(H16)|PC02<br>▶**2 samesets of gi|33590243**	483	55093	21 (9)	15 (7)	0.69
☑ **1.6**	**gi|112820171**<br>H21 0|EHEC serogroup: O113:H21|0 [gi|291323135|ref|EFE62563| Hxx 0|0|B088] [gi|293446305|ref|ZP_06662727| Hxx 0|0|B088]<br>▶**8 samesets of gi|112820171**	249	51472	9 (5)	7 (4)	0.36

▼**52 peptide matches (44 non-duplicate, 8 duplicate)**

Query	Dupes	Observed	Mr(expt)	Mr(calc)	Delta	M	Score	Expect	Rank	U	1	2	3	4	5	6	Peptide
33	▶1	**316.6911**	**631.3676**	**631.3653**	**0.0023**	**0**	**32**	**0.0066**	▶1		■	■	■	■	■	■	**R.LSSGLR.I**
113		355.1987	708.3828	708.3806	0.0022	0	13	0.36	▶1		■	■	■	■			R.FTSNIK.G
116	▶1	**358.7074**	**715.4002**	**715.3977**	**0.0026**	**0**	**28**	**0.011**	▶1		■	■	■	■			**K.GLTQAAR.N**
127		366.2272	730.4398	731.3813	-0.9415	0	1	2.9	▶3	U					■		R.LSEIDR.V
151	▶2	**380.2045**	**758.3944**	**758.4174**	**-0.0229**	**0**	**36**	**0.0016**	▶1	U					■		**K.LDEALAK.V**
155	▶1	**380.6960**	**759.3774**	**759.3763**	**0.0012**	**0**	**31**	**0.0041**	▶1		■	■	■	■			**R.LDEIDR.V**
282		**418.2382**	**834.4618**	**834.4600**	**0.0019**	**0**	**41**	**8.4e-05**	▶1	U			■				**K.AFVSVQGK.S**
440	▶1	**466.2518**	**930.4890**	**930.4883**	**0.0008**	**0**	**67**	**8.5e-07**	▶1			■	■	■	■		**R.SSLGAVQNR**
477		**473.2600**	**944.5054**	**944.5039**	**0.0015**	**0**	**52**	**1.9e-05**	▶1	U	■						**R.SSLGAIQNR.L**
605		500.3205	998.6264	998.5873	0.0392	1	4	0.65	▶1	U						■	K.ALAKVDNLR.S
615		**502.2625**	**1002.5104**	**1002.5094**	**0.0010**	**1**	**31**	**0.0049**	▶1		■	■	■	■			**K.SRLDEIDR.V**
645		508.7749	1015.5352	1014.5709	0.9643	0	5	0.34	▶1	U					■		K.ALATTNPLSK.L
647	▶1	**508.7910**	**1015.5674**	**1016.5502**	**-0.9828**	**0**	**13**	**0.045**	▶1	U				■			**K.NVDLSAVATK.L**
801		**551.2684**	**1100.5222**	**1100.5210**	**0.0012**	**0**	**74**	**3.5e-07**	▶1		■	■	■	■	■	■	**K.DDAAGQAIANR.F**
889		382.5606	1144.6600	1144.6564	0.0036	1	10	0.8	▶1		■	■	■	■	■	■	R.LSSGLRINSAK.D
975		397.1871	1188.5395	1187.6034	0.9361	0	4	0.42	▶1	U		■					K.ALDDAISQIDK.F
980		**596.3027**	**1190.5908**	**1190.5891**	**0.0018**	**0**	**81**	**4.7e-08**	▶1	U	■						**K.NQSALSSSIER.L**
1000		600.8543	1199.6940	1199.6734	0.0206	1	13	0.051	▶1	U					■		K.LRSSLGAVQNR.F
1038		407.5516	1219.6330	1220.6150	-0.9820	0	6	0.22	▶1	U					■		R.VSNQTQFNGVK.V

Query	Dupes	Observed	Mr(expt)	Mr(calc)	Delta	M	Score	Expect	Rank	U	1	2	3	4	5	6	Peptide
1158	▶1	641.3318	1280.6490	1280.6460	0.0031	0	72	1.1e-07	▶1	U	■						K.LTTETTSAGTATK.D
1366		712.8865	1423.7584	1423.7671	-0.0086	1	4	0.4	▶1	U					■		K.VYTANITNKTATK.G
1390		720.9124	1439.8102	1439.8096	0.0006	0	94	1.7e-09	▶1		■	■					K.AQIIQQAGNSVLAK.A
1448		743.8734	1485.7322	1485.7311	0.0012	0	62	9.4e-07	▶1	U						■	K.SEGGSPILVNEDAAK.S
1459		747.9197	1493.8248	1493.8202	0.0047	0	60	5.5e-06	▶1		■	■	■	■			K.ANQVPQQVLSLLQG.-
1481		753.8748	1505.7350	1505.7322	0.0029	0	98	1.7e-10	▶1	U	■						K.DTGTVTVASNGAGATGK.F
1542		779.4067	1556.7988	1556.7933	0.0055	0	104	6.2e-11	▶1	U	■						K.DPLAALDEAISSIDK.F
1548		781.4211	1560.8276	1560.8260	0.0016	0	63	2.4e-06	▶1		■	■	■	■			R.VSGQTQFNGVNVLAK
1678		836.3809	1670.7472	1670.7457	0.0015	0	126	1.6e-12	▶1		■	■	■	■			R.IQDADYATEVSNMSK.A
1704		843.4595	1684.9044	1685.8836	-0.9791	0	41	0.0003	▶1	U		■					K.IQVGANDGETITIDLK.K
1704		843.4595	1684.9044	1684.8996	0.0049	0	39	0.00046	▶2	U	■						K.IQVGANDGQTITIDLK.K
1745		860.3595	1718.7044	1718.7974	-0.0929	0	1	0.77	▶1	U					■		K.ALAYNDAPMSVYFGGK.N + Oxidation (M)
1995		647.6526	1939.9360	1938.9898	0.9461	0	1	0.72	▶1	U				■			K.IDSSTLGLSGFSVSQNSLK.L
2106		1043.0720	2084.1294	2084.1225	0.0069	0	124	2.5e-12	▶1		■	■	■	■	■	■	M.AQVINTNSLSLiTQNNiNK.N
2107		695.7171	2084.1295	2084.1225	0.0069	0	37	0.0012	▶1		■	■	■	■	■	■	M.AQVINTNSLSLiTQNNiNK.N
2114		1047.4980	2092.9814	2092.9801	0.0014	0	115	2.9e-12	▶1	U	■						K.TGDTVTTTGSSAAIYTYDAAK.G
2178		739.7242	2216.1508	2215.1630	0.9877	0	0	6.3	▶2		■	■	■	■	■	■	-.MAQVINTNSLSLiTQNNiNK.N
2204		750.3729	2248.0969	2248.0931	0.0038	0	82	3.6e-08	▶1		■	■					R.LDSAVTNLNNTTTNLSEAQSR.I
2205		1125.0560	2248.0974	2248.0931	0.0043	0	130	6.1e-13	▶1		■	■					R.LDSAVTNLNNTTTNLSEAQSR.I
2296		860.1271	2577.3595	2577.3498	0.0097	0	88	1.6e-09	▶1	U	■						K.TVSADTVLSTVQSAATANTAVTGATIK.Y
2303		877.1009	2628.2809	2628.2739	0.0070	0	74	1.8e-07	▶1		■	■			■		R.NANDGISVAQTTEGALSEINNNLQR.I
2304		1315.1480	2628.2814	2628.2739	0.0075	0	144	2e-14	▶1		■	■			■		R.NANDGISVAQTTEGALSEINNNLQR.I
2339		966.8297	2897.4673	2897.4591	0.0082	1	58	7.9e-06	▶1		■	■			■		R.NANDGISVAQTTEGALSEINNNLQRIR.E
2339		966.8297	2897.4673	2897.4591	0.0082	1	11	0.4	▶2	U			■				R.NANDGISLAQTTEGALSEINNNLQRVR.E
2383		1091.2490	3270.7252	3270.7167	0.0084	1	118	2.6e-12	▶1				■	■			M.AQVINTNSLSLITQNNINKNQSALSTSIER.L

▶61 subsets and intersections (154 subset proteins in total)

▶2 gi|307553085 14 Hxx(H54 27.9%) 0|0|ABU 83972

10 per page 1

Not what you expected? Try the select summary.

Mascot: http://www.matrixscience.com/
