# Supplementary material for: MS-H: A Novel Proteomic Approach to Isolate and Type the E. coli H Antigen Using Membrane Filtration and Liquid Chromatography-Tandem Mass Spectrometry (LC-MS/MS)
Source: PLoS One. 2013 Feb 21;8(2):e57339. doi: 10.1371/journal.pone.0057339 (PMC3578835; doi:10.1371/journal.pone.0057339)
Supplement: Representative Peptide Data S1 — Peptide data are represented as the Mascot search results from all 53 serotypes, obtained under the Orbitrap platform in Table 4 with related E. coli reference strains. “U” denotes a unique peptide specific for each of the proteins 1.1, 1.2, and beyond. The number 1.1 (shown as 1 in the peptide list and phylogenetic tree) represents the protein which obtained the highest score and confidence value after a Mascot search. This protein, known as the first hit, was used to designate the MS-H type of the unknown flagellin. Related peptides 1.2 (2), 1.3 (3), etc. represented the second, third, etc. hits for MS-H typing analysis. (DOCX) [file pone.0057339.s009.docx › H46-E214.pdf]

MATRIX SCIENCE MASCOT Search Results

User :
E-mail :
Search title : Submitted from 20110819-604 by Mascot Daemon on VARIABLE
MS data file : C:\Documents and Settings\keding\Desktop\Raw data\20110817-001-0079-00604\20110817-005-EC214MS1.RAW
Database : Flagellin_v2 (192 sequences; 89,845 residues)
Taxonomy : Bacteria (Eubacteria) (192 sequences)
Timestamp : 19 Aug 2011 at 17:54:09 GMT

Not what you expected? Try **the select summary**.

▸ **Search parameters**

▸ **Score distribution**

▸ **Legend**

Protein Family Summary

Significance threshold p< 0.05 Max. number of families AUTO
Ions score or expect cut-off 0 Dendrograms cut at 0

Protein family 1 (out of 1)

10 per page 1

▾**1**

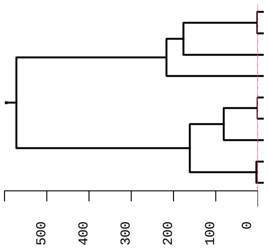

1	gi|30059920	2386	H46 0|H46|5306-56
9	gi|99645039	297	H27 0|H27|0
7	gi|37651050	931	H28 0|O145:H28|0917/99 [gi|37651052|ref|CAD97427| Hxx 0|O145:H-|2032/99]
6	gi|121144335	977	H55 0|O75:H55|E2987-73
2	gi|125659369	1785	H44 0|H44|781-55
4	gi|88683097	1122	H44 0|H44|0
8	gi|323174561	580	Hxx(H25 99.7%) 0|0|LT-68
3	gi|157081065	1268	Hxx(H28 99.7%) 0|0|E24377A [gi|157159035|ref|YP_001463228| Hxx 0|0|E24377A]
5	gi|30059928	988	H52 0|H52|C2187-69

Threshold (**0**): 0

	Accession	Score	Mass	Matches	Sequences	emPAI
1.1	gi|30059920<br>H46 0|H46|5306-56	2386	57918	48 (42)	36 (33)	7.62
1.2	gi|125659369<br>H44 0|H44|781-55	1785	58912	35 (30)	25 (23)	3.58
1.3	gi|157081065<br>Hxx(H28 99.7%) 0|0|E24377A [gi|157159035|ref|YP_001463228| Hxx 0|0|E24377A]<br>▸ **3 samesets of gi|157081065**	1268	59373	31 (24)	22 (18)	2.27
1.4	gi|88683097<br>H44 0|H44|0	1122	55289	28 (22)	20 (17)	2.18
1.5	gi|30059928<br>H52 0|H52|C2187-69	988	46003	27 (21)	19 (16)	3.00
1.6	gi|121144335<br>H55 0|O75:H55|E2987-73	977	62285	28 (21)	20 (16)	1.66
1.7	gi|37651050<br>H28 0|O145:H28|0917/99 [gi|37651052|ref|CAD97427| Hxx 0|O145:H-|2032/99]	931	55672	27 (21)	19 (16)	1.98
1.8	gi|323174561<br>Hxx(H25 99.7%) 0|0|LT-68<br>▸ **1 sameset of gi|323174561**	580	46392	21 (13)	16 (11)	1.44
1.9	gi|99645039<br>H27 0|H27|0	297	50847	16 (7)	12 (6)	0.55

▾**86 peptide matches (76 non-duplicate, 10 duplicate)**

Query	Dupes	Observed	Mr(expt)	Mr(calc)	Delta	M	Score	Expect	Rank	U	1	2	3	4	5	6	7	8	9	Peptide
25	▸1	**316.6894**	**631.3642**	**631.3653**	**-0.0011**	**0**	**33**	**0.0046**	▸1		■	■	■	■	■	■	■	■	■	**R.LSSGLR.I**
47		330.2076	658.4006	658.4014	-0.0007	0	5	0.33	▸2	U									■	K.AAVSLAK.D
55		337.2155	672.4164	673.3759	-0.9594	0	8	0.15	▸1	U								■		K.NGATALK.L
56		338.2050	674.3954	674.3963	-0.0009	0	10	0.12	▸1	U									■	K.TVTGLGK.T
89		355.1975	708.3804	708.3806	-0.0002	0	12	0.43	▸1		■	■	■	■	■	■	■	■		R.FTSNIK.G
94		**358.7059**	**715.3972**	**715.3977**	**-0.0004**	**0**	**28**	**0.012**	▸1		■	■	■	■	■	■	■	■		**K.GLTQAAR.N**
123		**380.6950**	**759.3754**	**759.3763**	**-0.0008**	**0**	**36**	**0.0014**	▸1		■	■	■	■	■	■	■	■		**R.LDEIDR.V**
130		**382.2130**	**762.4114**	**762.4123**	**-0.0009**	**0**	**19**	**0.013**	▸1	**U**	■									**K.IDSSTLK.L**
147		394.7136	787.4126	786.4599	0.9527	0	1	0.85	▸1	U								■		K.QTGQLIK.V
254		**427.7155**	**853.4164**	**853.4182**	**-0.0017**	**0**	**31**	**0.0008**	▸1		■	■								**K.FTTDAATK.A**
304		**441.2208**	**880.4270**	**880.4290**	**-0.0020**	**0**	**40**	**9.9e-05**	▸1	**U**	■									**K.GVYTDAAGK.F**
311		**446.2601**	**890.5056**	**890.5073**	**-0.0016**	**1**	**28**	**0.0052**	▸1	**U**	■									**K.KIDSSTLK.L**
329		452.2422	902.4698	902.5073	-0.0374	0	0	0.94	▸4	U			■							K.AATTADLLK.A
332		452.7137	903.4128	902.5073	0.9056	0	5	0.47	▸1				■				■			K.AATLDALTK.N
362		**459.2501**	**916.4856**	**916.4866**	**-0.0009**	**0**	**51**	**8.9e-06**	▸1	**U**	■									**K.AATTTDPLK.A**
377	▸1	**466.2507**	**930.4868**	**930.4883**	**-0.0014**	**0**	**67**	**8.4e-07**	▸1			■	■	■	■	■	■	■		**R.SSLGAVQNR.L**

Query	Dupes	Observed	Mr(expt)	Mr(calc)	Delta	M	Score	Expect	Rank	U	1	2	3	4	5	6	7	8	9	Peptide
392		468.2554	934.4962	934.4872	0.0090	0	1	0.88	1	U				■						K.LSGFNVNGK.G
413	1	473.2588	944.5030	944.5039	-0.0009	0	73	1.4e-07	1	U	■									R.SSLGAIQNR.L
478		488.2582	974.5018	974.5033	-0.0014	0	83	5.9e-09	1		■						■			K.AGDTANLSVK.I
487		489.6761	977.3376	978.5134	-1.1758	0	17	0.019	1	U					■					K.GFSVSGNALK.V
493		490.7559	979.4972	979.4975	-0.0002	0	26	0.0038	1		■						■			K.YTVDAGLNK.A
537	1	502.2612	1002.5078	1002.5094	-0.0016	1	37	0.0013	1		■	■	■	■	■	■	■	■		K.SRLDEIDR.V
538		335.1769	1002.5089	1002.5094	-0.0005	1	22	0.036	1		■	■	■	■	■	■	■	■		K.SRLDEIDR.V
690		537.7928	1073.5710	1073.5717	-0.0006	0	70	8.9e-08	1	U	■									R.ISADALQSAAK.G
752		551.2675	1100.5204	1100.5210	-0.0006	0	67	1.9e-06	1		■	■	■	■	■	■	■	■	■	K.DDAAGQAIANR.F
812		568.7949	1135.5752	1135.5543	0.0210	0	1	1	1	U		■								R.ISAEAMQSATK.T
899		594.8087	1187.6028	1187.6034	-0.0005	0	72	6.2e-08	1		■					■				K.ALDDAISQIDK.F
908		596.3016	1190.5886	1190.5891	-0.0004	0	60	5.4e-06	1		■		■		■		■		■	K.NQSALSSSIER.L
1029		627.8033	1253.5920	1253.5710	0.0210	0	2	0.64	2	U					■					K.FGANDTAAAAMAK.T + Oxidation (M)
1112	1	651.8622	1301.7098	1302.6415	-0.9317	0	7	0.45	1	U									■	K.AATASDLDLNNAK.K
1168		672.8772	1343.7398	1343.7408	-0.0010	0	62	6.8e-07	1	U						■				-.SLSLITQNNINK.N
1176		676.3384	1350.6622	1350.6627	-0.0004	0	78	1.8e-08	1	U		■								R.ELTVQSSTGTNSK.S
1279		720.9116	1439.8086	1439.8096	-0.0010	0	106	1e-10	1		■	■	■	■		■				K.AQIIQQAGNSVLAK.A
1288		724.3848	1446.7550	1446.7566	-0.0016	0	119	3.2e-12	1	U		■								K.IGATSVDVVLSSDGK.I
1307		730.9003	1459.7860	1459.7882	-0.0022	0	120	9.3e-13	1	U	■									K.IGTTSVNVVLASDGK.I
1307		730.9003	1459.7860	1460.7723	-0.9862	0	21	0.0086	2	U							■			K.IGTTSVDVVLASDGK.I
1307		730.9003	1459.7860	1460.7722	-0.9862	0	20	0.011	3	U			■							K.IGSTSIDVVLASDGK.I
1326		491.3515	1471.0327	1471.8246	-0.7919	1	0	0.9	1	U				■						K.QDVIIAKDGTLTAK.D
1350		497.9313	1490.7721	1490.7729	-0.0008	1	12	0.061	1		■					■				K.ALDDAISQIDKFR.S
1354		747.9190	1493.8234	1493.8202	0.0033	0	62	4.2e-06	1	U	■	■	■	■	■			■		K.ANQVPQQVLSLxQG.-
1395		506.9334	1517.7784	1517.7950	-0.0167	0	14	0.043	1	U				■						K.ANQVPQQVLSLHQG.-
1455	1	781.4195	1560.8244	1560.8260	-0.0016	0	65	1.4e-06	1		■	■	■	■	■	■	■			R.VSGQTQFNGVNVLAK.D
1526		538.9442	1613.8108	1613.8121	-0.0013	1	24	0.036	1		■	■	■	■	■	■	■	■	■	R.INSAKDDAAGQAIANR.F
1599		836.3797	1670.7448	1670.7457	-0.0009	0	132	4.1e-13	1		■	■	■	■	■	■		■		R.IQDADYATEVSNMSK.A
1600		836.4489	1670.8832	1670.8839	-0.0007	0	101	4.4e-10	1	U								■		K.IQVGANDGQTISIDLK.K
1625	1	843.4565	1684.8984	1684.8996	-0.0011	0	123	1.8e-12	1		■	■	■	■			■			K.IQVGANDGQTITIDLK
1626	1	843.4581	1684.9016	1685.8836	-0.9819	0	58	6.2e-06	2	U						■				K.IQVGANDGETITIDLK.K
1640		849.9779	1697.9412	1697.9424	-0.0012	0	114	4.2e-12	1	U		■								K.LAGATVAGQSGAIVVTGAR.I
1641		566.9877	1697.9413	1697.9424	-0.0012	0	65	3e-07	1	U		■								K.LAGATVAGQSGAIVVTGAR.I
1646		852.4256	1702.8366	1702.8374	-0.0007	0	106	2.3e-11	1	U	■									K.GTVINVDNGADDISVSK.T
1658		855.9126	1709.8106	1709.8109	-0.0002	0	108	1.4e-11	1	U	■									K.TGVVTTGGAPTYTDADGK.L
1686	1	575.7634	1724.2684	1724.9019	-0.6335	1	0	0.93	1	U									■	K.TMYLSKSEGGSPILVK.E + Oxidation (M)
1799		902.4515	1802.8884	1803.9438	-1.0554	1	1	4	1		■		■		■		■		■	K.NQSALSSSIERLSSGLR.I
1806	1	904.9603	1807.9060	1807.9064	-0.0004	0	106	3.5e-11	1	U	■									K.LTGFNVNGSGSVANTAATK.D
1918		954.9855	1907.9564	1907.9589	-0.0024	0	93	5.5e-10	1	U		■								K.VTIGGNQAYTQTDGTLAAK.N
1940		643.6630	1927.9672	1929.0418	-1.0747	1	7	0.36	1	U									■	K.SLQSTTNPLETIDKALAK.V
2001		499.4686	1993.8453	1992.9865	0.8588	0	18	0.043	1	U									■	R.FDSAITNLGNTVNNLSSAR.S
2039		686.6822	2057.0248	2057.0277	-0.0029	0	27	0.0018	1	U	■									K.DELAAAAAAAGTTPAVGTDGVTK.Y
2040		1029.5200	2057.0254	2057.0277	-0.0022	0	107	2e-11	1	U	■									K.DELAAAAAAAGTTPAVGTDGVTK.Y
2060		1043.0680	2084.1214	2084.1225	-0.0011	0	141	4.8e-14	1		■	■	■	■	■			■	■	M.AQVINTNSLSLITQNNINK.N
2061		695.7150	2084.1232	2084.1225	0.0006	0	77	1.2e-07	1		■	■	■	■	■			■	■	M.AQVINTNSLSLITQNNINK.N
2158		1125.0530	2248.0914	2248.0931	-0.0017	0	137	1.1e-13	1		■	■	■		■	■	■			R.LDSAVTNLNNTTTNLSEAQSR.I
2159		750.3715	2248.0927	2248.0931	-0.0004	0	88	9.2e-09	1		■	■	■		■	■	■			R.LDSAVTNLNNTTTNLSEAQSR.I
2198		577.0193	2304.0481	2304.2550	-0.2069	1	7	0.22	1	U						■				K.AQIIQQAGNSVLAKANQVPQQV.-
2212		785.0098	2352.0076	2353.1471	-1.1396	0	9	0.11	1	U						■				K.DSLLSMLAPNAGDSFTASVSIGGK.A + Oxidation (M
2221		1210.0840	2418.1534	2418.1551	-0.0017	0	101	8.3e-11	1	U	■									K.LTTTNTVDYFLQTDGSVTNGSGK.G
2222		807.0588	2418.1546	2418.1551	-0.0005	0	52	6e-06	1	U	■									K.LTTTNTVDYFLQTDGSVTNGSGK.G
2253		1297.1280	2592.2414	2592.2402	0.0012	0	101	8.4e-11	1	U	■									R.ELTVQATTGTNSQSDLDSIQDEIK.S
2258		877.0997	2628.2773	2628.2739	0.0034	0	67	8.3e-07	1		■	■	■	■	■	■	■			R.NANDGISVAQTTEGALSEINNNLQR
2259		1315.1460	2628.2774	2628.2739	0.0035	0	120	4.5e-12	1		■	■	■	■	■	■	■			R.NANDGISVAQTTEGALSEINNNLQR
2263		881.4593	2641.3561	2641.3559	0.0002	0	75	4.5e-08	1	U		■								K.ADLTAAQLTTTAAGGTIAAPAADANGVTK.Y
2263		881.4593	2641.3561	2642.2896	-0.9335	0	0	1.4	2	U								■		R.NANDGISLAQTTEGALSEINNNLQR.V
2299		966.8293	2897.4661	2897.4591	0.0070	1	49	6.5e-05	1			■	■	■	■	■	■			R.NANDGISVAQTTEGALSEINNNLQRIR.E
2299		966.8293	2897.4661	2897.4591	0.0070	1	5	1.5	2	U								■		R.NANDGISLAQTTEGALSEINNNLQRVR.E
2336		1097.2310	3288.6712	3288.6732	-0.0020	1	99	1.2e-10	1			■		■						M.AQVINTNSLSLITQNNINKNQSSMSTAIER.L
2395		1283.3170	3846.9292	3846.9236	0.0056	1	79	1.1e-08	1	U	■									K.LTGFNVNGSGSVANTAATKDELAAAAAAAGTTPAVGTDGV

59 subsets and intersections (159 subset proteins in total)

10 per page 1

Not what you expected? Try **the select summary**.

Mascot: http://www.matrixscience.com/
