# Supplementary material for: MS-H: A Novel Proteomic Approach to Isolate and Type the E. coli H Antigen Using Membrane Filtration and Liquid Chromatography-Tandem Mass Spectrometry (LC-MS/MS)
Source: PLoS One. 2013 Feb 21;8(2):e57339. doi: 10.1371/journal.pone.0057339 (PMC3578835; doi:10.1371/journal.pone.0057339)
Supplement: Representative Peptide Data S1 — Peptide data are represented as the Mascot search results from all 53 serotypes, obtained under the Orbitrap platform in Table 4 with related E. coli reference strains. “U” denotes a unique peptide specific for each of the proteins 1.1, 1.2, and beyond. The number 1.1 (shown as 1 in the peptide list and phylogenetic tree) represents the protein which obtained the highest score and confidence value after a Mascot search. This protein, known as the first hit, was used to designate the MS-H type of the unknown flagellin. Related peptides 1.2 (2), 1.3 (3), etc. represented the second, third, etc. hits for MS-H typing analysis. (DOCX) [file pone.0057339.s009.docx › H47-E346.pdf]

MATRIX SCIENCE MASCOT Search Results

User :
E-mail :
Search title : Submitted from 20110825-0614-02 by Mascot Daemon on VARIABLE
MS data file : C:\Documents and Settings\keding\Desktop\Raw data\20110824-001-0031-00614\20110824-004-EC346-MS1.RAW
Database : Flagellin_v2 (192 sequences; 89,845 residues)
Taxonomy : Bacteria (Eubacteria) (192 sequences)
Timestamp : 25 Aug 2011 at 16:45:28 GMT

Not what you expected? Try **the select summary**.

▶ **Search parameters**

▶ **Score distribution**

▶ **Legend**

Protein Family Summary

Significance threshold p< 0.05 Max. number of families AUTO
Ions score or expect cut-off 0 Dendrograms cut at 0

Protein families 1–3 (out of 3)

10 per page 1

▼**1**

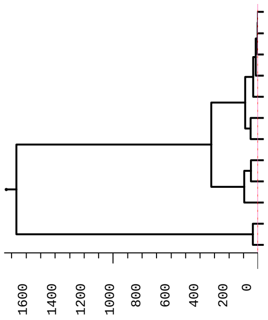

1	gi|154710458	25220	H47 0|H47|0
7	gi|6941882	2119	H14 0|O138:H14|O138:H14
6	gi|16901498	2121	H34 0|H34|0
5	gi|12516024	2135	H7 0|O157:H7|EDL933 [gi|15802358|ref|NP_288384| H7 0|O157:H7|EDL933]
3	gi|46093545	7219	H3 0|0|Bi7327-41
12	gi|37651050	713	H28 0|O145:H28|0917/99 [gi|37651052|ref|CAD97427| Hxx 0|O145:H-|2032/99]
11	gi|121144335	731	H55 0|O75:H55|E2987-73
9	gi|30577587	1527	H17 0|O15:H17|P12b
10	gi|33590227	1472	H42 NM|O157:NM(H42)|25
4	gi|30059872	2775	H16 0|H16|F 8316-41
2	gi|112820171	10287	H21 0|EHEC serogroup: O113:H21|0 [gi|291323135|ref|EFE62563| Hxx 0|0|B088] [gi|29344630...
8	gi|62532942	1918	H11 0|O26:H11|0

Threshold (**0**): 0

		Score	Mass	Matches	Sequences	emPAI
1.1	gi|154710458<br>H47 0|H47|0	25220	39177	572 (511)	48 (47)	603.75
1.2	gi|112820171<br>H21 0|EHEC serogroup: O113:H21|0 [gi|291323135|ref|EFE62563| Hxx 0|0|B088] [gi|293446305|ref|ZP_06662727| Hxx 0|0|B088]	10287	51472	244 (194)	40 (39)	29.29
1.3	gi|46093545<br>H3 0|0|Bi7327-41	7219	55534	158 (137)	15 (13)	3.22
1.4	gi|30059872<br>H16 0|H16|F 8316-41	2775	52639	103 (62)	16 (11)	1.81
1.5	gi|12516024<br>H7 0|O157:H7|EDL933 [gi|15802358|ref|NP_288384| H7 0|O157:H7|EDL933]	2135	59916	84 (54)	13 (8)	0.71
1.6	gi|16901498<br>H34 0|H34|0<br>▶ 3 samesets of gi|16901498	2121	56006	79 (54)	10 (8)	0.77
1.7	gi|6941882<br>H14 0|O138:H14|O138:H14	2119	56492	84 (54)	15 (8)	0.76
1.8	gi|62532942<br>H11 0|O26:H11|0	1918	37909	56 (46)	17 (16)	4.32
1.9	gi|30577587<br>H17 0|O15:H17|P12b	1527	36285	77 (52)	9 (7)	1.20
1.10	gi|33590227<br>H42 NM|O157:NM(H42)|25	1472	44094	74 (54)	9 (7)	0.92
1.11	gi|121144335<br>H55 0|O75:H55|E2987-73	731	62285	56 (32)	9 (7)	0.51
1.12	gi|37651050<br>H28 0|O145:H28|0917/99 [gi|37651052|ref|CAD97427| Hxx 0|O145:H-|2032/99]<br>▶ 1 sameset of gi|37651050	713	55672	62 (32)	13 (7)	0.58

▼**821 peptide matches (163 non-duplicate, 658 duplicate)**

Query	Dupes	Observed	Mr(expt)	Mr(calc)	Delta	M	Score	Expect	Rank	U	1	2	3	4	5	6	7	8	9	10	11	12	Peptide
19	▶11	316.6910	631.3674	631.3653	0.0021	0	31	0.0081	▶1		■	■	■	■	■	■	■		■	■	■	■	R.LSSGLR.I
59		347.2012	692.3878	692.3857	0.0021	0	28	0.0042	▶1			■		■									R.FTANIK.G
64		353.1935	704.3724	704.3705	0.0020	0	47	2.1e-05	▶1	U	■												K.DGATTLK.G
67	▶4	355.1985	708.3824	708.3806	0.0018	0	23	0.031	▶1		■		■		■	■	■		■	■	■	■	R.FTSNIK.G
76		358.7022	715.3898	715.3864	0.0034	0	6	1.8	▶1	U										■			R.LAEIDR.V
83	▶1	366.7050	731.3954	731.3926	0.0029	0	34	0.0014	▶1		■	■		■									K.GLTQASR.N
89	▶9	373.7066	745.3986	745.3970	0.0016	0	45	3.3e-05	▶1	U	■												K.TSNPLSK.I

Query	Dupes	Observed	Mr(expt)	Mr(calc)	Delta	M	Score	Expect	Rank	U	1	2	3	4	5	6	7	8	9	10	11	12	Peptide
104	▶13	380.6961	759.3776	759.3763	0.0014	0	39	0.00074	▶1		■				■	■	■		■		■	■	R.LDEIDR.V
106		380.6963	759.3780	758.4174	0.9607	0	3	2.6	▶1	U				■									K.LDEALAK.V
125		387.7036	773.3926	773.3919	0.0007	0	22	0.015	▶1			■						■					R.LEEIDR.V
129	▶3	388.2327	774.4508	774.4487	0.0021	0	51	2e-05	▶1	U	■												K.IDSTVLK.L
163	▶1	409.2249	816.4352	816.4341	0.0011	0	26	0.0026	▶1	U		■											K.LTANVDGK.A
167		411.2047	820.3948	820.3927	0.0022	0	33	0.00051	▶1	U	■												K.DSTSVQGK.F
174	▶12	415.2260	828.4374	828.4381	-0.0007	0	54	4.4e-06	▶1	U	■												K.YYAVVSK.S
224		428.7618	855.5090	855.3974	0.1116	0	1	0.73	▶2						■								K.SGDFTTTK.S
232		431.2653	860.5160	860.4603	0.0557	0	1	0.87	▶1	U							■						K.AATTADALK.A
269		444.2635	886.5124	886.5124	0.0001	0	2	0.74	▶2	U						■							K.GSDILAALK.T
269		444.2635	886.5124	886.4760	0.0365	0	0	0.94	▶3	U							■					■	K.AATTADpLK.A
278	▶1	446.2292	890.4438	890.4531	-0.0093	1	15	0.1	▶1	U					■								K.ADMKALDK.A
279		446.2435	890.4724	890.4709	0.0015	0	42	0.00019	▶1			■						■					K.ATGSDLISK.F
292	▶6	452.2797	902.5448	902.5437	0.0012	1	44	4.4e-05	▶1	U	■												K.KIDSTVLK.L
298	▶1	301.8560	902.5462	902.5437	0.0025	1	17	0.02	▶1	U	■												K.KIDSTVLK.L
298		301.8560	902.5462	903.4661	-0.9200	0	5	0.31	▶2	U							■						K.AATTADNLK.A
301		454.2269	906.4392	906.4480	-0.0088	1	29	0.0012	▶1	U					■								K.ADMKALDK.A + Oxidation (M)
321		308.1684	921.4834	922.4616	-0.9782	1	2	0.68	▶1	U					■								K.ADMKALMK.A + Oxidation (M)
331		466.7435	931.4724	930.4883	0.9842	0	4	1.6	▶1				■	■			■		■		■	■	R.SSLGAVQNR
342		315.5109	943.5109	944.5039	-0.9930	0	11	0.18	▶1						■	■							R.SSLGAIQNR.L
342		315.5109	943.5109	944.5179	-1.0070	0	3	1.3	▶2	U			■										K.IDADTLGLK.D
355	▶13	480.2487	958.4828	958.4832	-0.0004	0	76	4.9e-08	▶1		■	■						■					R.SDLGAVQNR.F
356	▶10	480.2493	958.4840	958.5196	-0.0355	0	39	0.00023	▶2	U										■			R.SSLGVVQNR.L
373		481.8287	961.6428	961.5055	0.1374	1	5	0.32	▶1	U					■								K.ADMKALWK.A
417	▶2	495.7645	989.5144	989.5142	0.0003	0	70	1.2e-07	▶1	U		■											K.NSAGQLTATK.V
464		511.3664	1020.7182	1019.5400	1.1783	0	4	0.65	▶1	U										■			K.AIAQVDTFR.S
499	▶2	524.2608	1046.5070	1046.5066	0.0004	0	20	0.018	▶1			■						■					K.VLAENNEMK.I
519		532.7952	1063.5758	1062.5015	1.0743	0	3	0.86	▶1			■						■					K.VLAENNEMK.I + Oxidation (M)
565	▶14	551.2681	1100.5280	1100.5210	0.0006	0	76	2.2e-07	▶1		■	■	■	■	■	■	■		■	■	■	■	K.DDAAGQAIANR.F
605	▶1	559.8071	1117.5996	1117.5979	0.0017	1	39	0.00013	▶1	U	■												K.DGATTLKGDIK.D
607	▶1	373.5411	1117.6015	1117.5979	0.0036	1	22	0.0061	▶1	U	■												K.DGATTLKGDIK.D
610	▶20	560.2803	1118.5460	1118.5455	0.0005	0	76	2.7e-08	▶1	U	■												K.VVDEDATAATK.T
645		565.2983	1128.5820	1128.5815	0.0005	1	44	4.2e-05	▶1	U	■												K.DGKYYAVVSK.S
646		377.2021	1128.5845	1128.5815	0.0030	1	25	0.0029	▶1	U	■												K.DGKYYAVVSK.S
655	▶1	568.2834	1134.5522	1134.5517	0.0006	0	61	1.4e-06	▶1			■						■					K.TITETASGNNK.V
657		569.4087	1136.8028	1137.5150	-0.7121	0	5	0.35	▶1	U				■									K.DTTDATGTAGTK.V
683		573.3351	1144.6556	1144.6564	-0.0007	1	62	6e-06	▶1		■	■	■	■	■	■	■		■	■	■	■	R.LSSGLRINSAK.D
684		382.5596	1144.6570	1144.6564	0.0006	1	4	3.7	▶1		■	■	■	■	■	■	■		■	■	■	■	R.LSSGLRINSAK.D
727	▶12	582.7974	1163.5802	1163.5782	0.0020	0	74	1.3e-07	▶1		■	■	■	■									K.SQSSLSSAIER.L
738		390.1810	1167.5212	1167.5441	-0.0230	0	13	0.056	▶1	U												■	R.MSAESLQSATK.S + Oxidation (M)
815	▶1	407.5527	1219.6363	1220.6150	-0.9787	0	0	0.96	▶1	U				■									R.VSNQTQFNGVK.V
841	▶1	617.8182	1233.6218	1233.6201	0.0017	1	46	9.3e-05	▶1	U	■												K.GDIKDSTSVQGK.F
842		412.2150	1233.6232	1233.6201	0.0031	1	16	0.1	▶1	U	■												K.GDIKDSTSVQGK.F
845	▶4	618.8153	1235.6160	1235.6146	0.0014	0	60	2.4e-06	▶1			■						■					R.VSEQTQFNGVK.V
846	▶3	618.8159	1235.6172	1234.6306	0.9866	0	40	0.0002	▶2	U			■										R.VSQQTQFNGVK.V
924	▶19	648.8212	1295.6278	1295.6245	0.0033	0	75	3.3e-08	▶1	U	■												R.DLDVVSETQYK.D
947		651.8510	1301.6874	1301.6827	0.0048	0	69	2.6e-07	▶1			■						■					K.AATLSDLDLNAAK.K
971	▶15	658.8247	1315.6348	1315.6296	0.0053	0	73	4.6e-08	▶1	U	■												K.FDTTSEAAISFK.D
976	▶3	439.5525	1315.6357	1315.6296	0.0061	0	36	0.00024	▶1	U	■												K.FDTTSEAAISFK.D
981	▶2	659.8293	1317.6440	1317.6412	0.0028	0	96	2.3e-10	▶1	U	■												K.IDDAISDVDSLR.S
984	▶1	440.2222	1317.6448	1317.6412	0.0036	0	31	0.00075	▶1	U	■												K.IDDAISDVDSLR.S
1019		672.8796	1343.7446	1343.7408	0.0038	0	84	4e-09	▶1	U											■		-.SLSLITQNNINK.N
1025	▶1	450.2369	1347.6889	1347.6882	0.0007	1	57	1.9e-06	▶1	U	■												K.TKVVDEDATAATK.T
1027	▶3	674.8521	1347.6896	1347.6882	0.0015	1	79	1.3e-08	▶1	U	■												K.TKVVDEDATAATK.T
1052		683.3371	1364.6596	1364.6572	0.0024	0	48	1.5e-05	▶1	U								■					R.FDSAITNLGNTVN.-
1081	▶5	694.8694	1387.7242	1387.7195	0.0048	0	98	1.5e-10	▶1	U	■												K.VTVDLADAAGDLTK.T
1082	▶1	463.9126	1388.7160	1387.7195	0.9965	0	2	0.57	▶1	U	■												K.VTVDLADAAGDLTK.T
1111		474.9285	1421.7637	1421.7627	0.0010	1	31	0.00075	▶1	U	■												R.FTSNIKGLTQASR.N
1129		715.8971	1429.7796	1429.7776	0.0020	1	94	5.8e-10	▶1			■						■					K.AATLSDLDLNAAKK.T
1150	▶1	724.8750	1447.7354	1447.7307	0.0047	0	58	3.1e-06	▶1			■						■					K.TLGLDGFNIDGAQK.A
1171	▶1	487.5945	1459.7617	1459.7630	-0.0014	1	29	0.0012	▶1	U		■											K.NSAGQLTATKVENK.A
1171		487.5945	1459.7617	1460.7723	-1.0106	0	6	0.26	▶2	U												■	K.IGTTSVDVVLASDGK.I
1202	▶2	743.8738	1485.7330	1485.7311	0.0020	0	84	6.6e-09	▶1			■						■					K.SEGGSPILVNEDAAK.S
1238	▶15	758.9155	1515.8164	1515.8144	0.0020	1	138	1.7e-14	▶1	U	■												K.KVTVDLADAAGDLTK.T
1248	▶15	506.2803	1515.8191	1515.8144	0.0046	1	47	2.1e-05	▶1	U	■												K.KVTVDLADAAGDLTK.T
1301	▶1	774.5765	1547.1384	1545.7886	1.3498	0	5	0.57	▶1			■						■					K.SLQSTTNPLETIDK.A
1337	▶20	783.4141	1564.8136	1564.8097	0.0040	1	88	1.6e-09	▶1	U	■												K.LRDLDVVSETQYK.D
1343	▶14	522.6125	1564.8157	1564.8097	0.0060	1	36	0.00026	▶1	U	■												K.LRDLDVVSETQYK.D
1417	▶1	807.9136	1613.8126	1613.8121	0.0005	1	91	7.7e-09	▶1		■	■	■	■	■	■	■		■	■	■	■	R.INSAKDDAAGQAIANR.F
1420	▶2	538.9460	1613.8162	1613.8121	0.0041	1	48	0.00013	▶1		■	■	■	■	■	■	■		■	■	■	■	R.INSAKDDAAGQAIANR.F
1430	▶4	539.6002	1615.7788	1615.7730	0.0058	1	35	0.00035	▶1	U	■												K.FDTTSEAAISFKDGK.Y
1434	▶2	809.4411	1616.8676	1615.7730	1.0947	1	31	0.00082	▶1	U	■												K.FDTTSEAAISFKDGK.Y
1466		823.9116	1645.8086	1644.9046	0.9040	1	15	0.033	▶1	U						■							K.TTANTAAKGSDILAALK.T
1505		422.2193	1684.8481	1685.8836	-1.0355	0	1	3.2	▶1						■						■		K.IQVGANDGETITIDLK.K
1510		564.6055	1690.7947	1690.7686	0.0261	0	0	0.9	▶1	U							■						K.SYSFDTTTASAADVQK.Y
1522		850.8785	1699.7424	1699.7359	0.0065	0	123	8.5e-13	▶1			■		■									R.IEDADYATEVSNMSR.A
1537		572.9188	1715.7346	1715.7308	0.0038	0	66	5.3e-07	▶1		■		■										R.IEDSDYATEVSNMSR.A
1544	▶17	858.8751	1715.7356	1715.7308	0.0048	0	146	5.6e-15	▶1		■		■										R.IEDSDYATEVSNMSR.A
1546	▶7	858.8754	1715.7362	1715.7308	0.0054	0	17	0.04	▶2			■		■									R.IEDADYATEVSNMSR.A + Oxidation (M)
1573		578.2492	1731.7258	1731.7257	0.0000	0	35	0.00034	▶1		■		■										R.IEDSDYATEVSNMSR.A + Oxidation (M)
1574		866.8713	1731.7280	1731.7257	0.0023	0	121	7.7e-13	▶1		■		■										R.IEDSDYATEVSNMSR.A + Oxidation (M)
1595		581.3096	1740.9070	1740.8530	0.0540	0	6	0.24	▶1	U									■				K.QVNLLSYTDTASNSTK.Y
1598	▶12	871.4611	1740.9076	1740.9006	0.0070	0	130	1.1e-13	▶1	U	■												K.IQVGANDNQSIDINLK.K

Query	Dupes	Observed	Mr(expt)	Mr(calc)	Delta	M	Score	Expect	Rank	U	1	2	3	4	5	6	7	8	9	10	11	12	Peptide
1602	4	581.3100	1740.9082	1740.9006	0.0076	0	55	3.1e-06	1	U	■												K.IQVGANDNQSIDINLK.K
1639	7	878.9780	1755.9414	1755.9367	0.0048	0	94	7.1e-10	1			■						■					K.IQVGANDGETITINLAK.I
1642	1	586.3218	1755.9436	1755.9367	0.0069	0	30	0.002	1			■						■					K.IQVGANDGETITINLAK.I
1655		589.2959	1764.8659	1763.8789	0.9870	1	13	0.047	1	U							■						K.LTTDAETKAATTADDLK.A
1745	17	918.9539	1835.8932	1835.8901	0.0031	0	108	1.7e-11	1	U	■												R.VSQQTEFNGVSVLGEDK.T
1752	11	612.9723	1835.8951	1835.8901	0.0049	0	62	5.8e-07	1	U	■												R.VSQQTEFNGVSVLGEDK.T
1793	1	927.9609	1853.9072	1853.9007	0.0065	0	115	5.8e-12	1			■						■					K.TGSTLVVNGATYDVSADGK.T
1796		619.6531	1855.9375	1855.9349	0.0025	1	39	0.00016	1			■						■					K.TITETASGNNKVMYLSK.S
1797		928.9767	1855.9388	1855.9349	0.0039	1	94	5e-10	1			■						■					K.TITETASGNNKVMYLSK.S
1818	9	935.5076	1869.0006	1868.9956	0.0051	1	63	5.4e-07	1	U	■												K.IQVGANDNQSIDINLKK.I
1824	14	624.0082	1869.0028	1868.9956	0.0072	1	52	6.4e-06	1	U	■												K.IQVGANDNQSIDINLKK.I
1844	1	937.9274	1873.8402	1874.0361	-0.1958	1	1	1.7	3	U												■	K.IGTTSVDVVLASDGKITAK.D
1845		469.4677	1873.8417	1874.0361	-0.1944	1	3	1	1	U												■	K.IGTTSVDVVLASDGKITAK.D
1902		972.4443	1942.8740	1942.8690	0.0050	1	83	8.9e-09	1			■		■									R.SRIEDADYATEVSNMSR.A
1913	14	653.9627	1958.8663	1958.8640	0.0023	1	63	1.1e-06	1		■		■										R.SRIEDSDYATEVSNMSR.A
1915	12	980.4409	1958.8672	1958.8640	0.0033	1	88	4.1e-09	1		■		■										R.SRIEDSDYATEVSNMSR.A
1918		490.7243	1958.8681	1958.8640	0.0041	1	21	0.018	1		■		■										R.SRIEDSDYATEVSNMSR.A
1928	11	980.4434	1958.8722	1958.8640	0.0083	1	17	0.051	2			■		■									R.SRIEDADYATEVSNMSR.A + Oxidation (M)
1935	5	653.9653	1958.8741	1958.8640	0.0101	1	22	0.015	2			■		■									R.SRIEDADYATEVSNMSR.A + Oxidation (M)
1949		659.2951	1974.8635	1974.8589	0.0046	1	58	1.7e-06	1		■		■										R.SRIEDSDYATEVSNMSR.A + Oxidation (M)
1950		988.4395	1974.8644	1974.8589	0.0056	1	78	1.6e-08	1		■		■										R.SRIEDSDYATEVSNMSR.A + Oxidation (M)
1954	1	661.6696	1981.9870	1981.9844	0.0026	1	44	9.2e-05	1	U	■												R.DLDVVSETQYKDGATTLK.G
1955		992.0011	1981.9876	1981.9844	0.0032	1	87	4e-09	1	U	■												R.DLDVVSETQYKDGATTLK.G
1958	1	661.6741	1982.0005	1981.9957	0.0048	1	61	1.7e-06	1			■						■					K.KTGSTLVVNGATYDVSADGK.T
1959		992.0077	1982.0008	1981.9957	0.0052	1	116	5.3e-12	1			■						■					K.KTGSTLVVNGATYDVSADGK.T
1964		664.6728	1990.9966	1990.9960	0.0006	1	44	7.5e-05	1			■						■					R.LEEIDRVSEQTQFNGVK.V
1965		996.5073	1991.0000	1990.9960	0.0041	1	74	7.2e-08	1			■						■					R.LEEIDRVSEQTQFNGVK.V
1969	12	997.5035	1992.9924	1992.9865	0.0060	0	156	6.2e-16	1		■	■	■	■									R.FDSAITNLGNTVNNLSSAR.S
1974	3	665.3391	1992.9955	1992.9865	0.0090	0	80	2.5e-08	1		■	■	■	■									R.FDSAITNLGNTVNNLSSAR.S
1995		1002.5410	2003.0674	2003.0422	0.0252	1	2	0.58	1	U							■						K.AATTADALKALDEAISSIDK.F
2004		1007.5550	2013.0954	2013.0854	0.0100	0	65	2.9e-07	1	U												■	-.QVINTNSLSLITQNNINK.N
2028	2	1021.0320	2040.0494	2040.0415	0.0079	0	112	6.8e-12	1	U		■											K.IVYEGIEFTNTGTVAIDAK.G
2031	5	681.0239	2040.0499	2040.0415	0.0083	0	42	6.9e-05	1	U		■											K.IVYEGIEFTNTGTVAIDAK.G
2040		512.7350	2046.9109	2046.0480	0.8629	1	0	0.91	1	U							■						K.AATTADNLKALDEAISSIDK.F
2041		514.0254	2052.0725	2050.9492	1.1233	1	1	0.83	1	U				■									K.MGKALAYNDAPMSVYFGGK.N + 2 Oxidatio
2055	11	1043.0710	2084.1274	2084.1225	0.0049	0	141	5.1e-14	1		■	■	■		■	■	■						M.AQVINTNSLSLiTQNNiNK.N
2059	10	695.7172	2084.1298	2084.1225	0.0072	0	71	5.2e-07	1		■	■	■		■	■	■						M.AQVINTNSLSLiTQNNiNK.N
2059	9	695.7172	2084.1298	2085.1066	-0.9768	0	60	6.6e-06	4	U										■			M.AQVINTNSLSLITQNNIDK.N
2059	9	695.7172	2084.1298	2085.0814	-0.9516	0	56	1.7e-05	5	U									■				M.AQVINTNSLSLNTQNNINK.N
2065	11	1043.0730	2084.1314	2085.1066	-0.9751	0	90	6.9e-09	4	U										■			M.AQVINTNSLSLITQNNIDK.N
2072	11	1043.5640	2085.1134	2085.0814	0.0320	0	96	1.6e-09	1	U									■				M.AQVINTNSLSLNTQNNINK.N
2116	2	712.3444	2134.0114	2134.0066	0.0047	0	30	0.0011	1	U		■											K.QVYVSTADGSLTTSSDTQFK.I
2130	17	1068.0150	2134.0154	2134.0066	0.0088	0	154	4.1e-16	1	U		■											K.QVYVSTADGSLTTSSDTQFK.I
2157	1	727.0475	2178.1207	2178.1168	0.0038	1	60	1.1e-06	1	U	■												R.VSQQTEFNGVSVLGEDKTLK.I
2196		753.7150	2258.1232	2258.1139	0.0093	1	64	4.4e-07	1	U	■												K.IDDAISDVDSLRSDLGAVQNR.F
2248		1199.1440	2396.2734	2396.2659	0.0075	0	112	6.7e-12	1	U				■									R.AQILQQAGTSVLAQANQTTQNVL.-
2249		799.7674	2396.2804	2396.2659	0.0144	0	54	3.7e-06	1	U				■									R.AQILQQAGTSVLAQANQTTQNVL.-
2277		860.0955	2577.2647	2577.2558	0.0088	1	52	6.4e-06	1	U	■												R.LDEIDRVSQQTEFNGVSVLGEDK.T
2278		1289.6410	2577.2674	2577.2558	0.0116	1	72	6e-08	1	U	■												R.LDEIDRVSQQTEFNGVSVLGEDK.T
2278		1289.6410	2577.2674	2577.3035	-0.0360	1	5	0.33	2	U												■	K.KIDSDTLGLSGFNVNGGGAVANTAATK.D
2292	18	1300.1420	2598.2694	2598.2634	0.0061	0	118	1.8e-12	1	U	■												R.NANDGISVAQTTEGALGEINNNLQR.I
2319	16	867.1010	2598.2812	2598.2634	0.0178	0	89	1.4e-09	1	U	■												R.NANDGISVAQTTEGALGEINNNLQR.I
2327	10	1308.6040	2615.1934	2615.1835	0.0100	0	116	2.3e-12	1	U	■												K.STTTDNNGIYAASVDSDGNVTIDASK.K
2352		886.1049	2655.2929	2655.2848	0.0081	0	34	0.00093	1	U		■											R.NANDGISVAQTTEGALNEINNNLQR.I
2353		1328.6540	2655.2934	2655.2848	0.0086	0	125	8.5e-13	1	U		■											R.NANDGISVAQTTEGALNEINNNLQR.I
2360		1338.6730	2675.3314	2675.3178	0.0137	0	23	0.0048	1	U		■											K.AVEFTISGSTDTSGTSATVAPTTALYK.N
2375	5	1372.6510	2743.2874	2743.2784	0.0090	1	89	1.4e-09	1	U	■												K.STTTDNNGIYAASVDSDGNVTIDASKK.V
2384	14	915.4374	2743.2904	2743.2784	0.0119	1	84	4e-09	1	U	■												K.STTTDNNGIYAASVDSDGNVTIDASKK.V
2400	9	922.1161	2763.3265	2763.3159	0.0106	0	79	1.5e-08	1	U		■											R.ELSVQATNGTNSDSDLSSIQAEITQR.L
2418	7	951.1215	2850.3427	2850.3301	0.0125	0	96	2.4e-10	1	U	■												R.ELTVQATNGTNSQSDMESIQAEITQR.L
2431	10	1426.1800	2850.3454	2850.3301	0.0153	0	143	5e-15	1	U	■												R.ELTVQATNGTNSQSDMESIQAEITQR.L
2434	1	717.4006	2865.5733	2865.5672	0.0061	0	47	1.8e-05	1		■	■	■										R.AQILQQAGTSVLAQANQTTQNVLSLLR.-
2437	1	956.2030	2865.5872	2865.5672	0.0200	0	112	5.7e-12	1		■	■	■										R.AQILQQAGTSVLAQANQTTQNVLSLLR.-
2439	10	956.7975	2867.3707	2866.3250	1.0456	0	35	0.00031	1	U	■												R.ELTVQATNGTNSQSDMESIQAEITQR.L + Oxi
2452		956.7995	2867.3767	2867.4485	-0.0718	1	10	0.091	1	U	■												R.NANDGISVAQTTEGALGEINNNLQRIR.E
2496		975.8353	2924.4841	2924.4700	0.0141	1	72	1.1e-07	1	U		■											R.NANDGISVAQTTEGALNEINNNLQRIR.E
2498		734.3747	2933.4697	2933.4591	0.0106	1	36	0.00041	1		■	■											R.SDLGAVQNRFDSAITNLGNTVNNLSSAR.S
2499		978.8316	2933.4730	2933.4591	0.0139	1	78	2.3e-08	1		■	■											R.SDLGAVQNRFDSAITNLGNTVNNLSSAR.S
2514	1	780.8889	3119.5265	3119.5153	0.0112	1	38	0.00017	1	U	■												R.IRELTVQATNGTNSQSDMESIQAEITQR.L
2515	1	1040.8500	3119.5282	3119.5153	0.0129	1	101	8.4e-11	1	U	■												R.IRELTVQATNGTNSQSDMESIQAEITQR.L
2535	9	1063.4970	3187.4692	3187.4542	0.0150	1	120	1.1e-12	1	U		■											K.ATGTDNYQINGTDNYTVNVDSGVVQDKDGK.Q
2538		808.4333	3229.7041	3229.6902	0.0139	1	28	0.0023	1		■	■	■										M.AQVINTNSLSLLTQNNLNKSQSSLSSAIER.L
2540	2	1077.5760	3229.7062	3229.6902	0.0160	1	146	3.6e-15	1		■	■	■										M.AQVINTNSLSLLTQNNLNKSQSSLSSAIER.L

42 subsets and intersections (156 subset proteins in total)

2	gi|112820172	2190	H21 0|EHEC serogroup: O113:H21|0
3	gi|307553085	974	Hxx(H54 27.9%) 0|0|ABU 83972

10 per page 1

Not what you expected? Try **the select summary**.

Mascot: http://www.matrixscience.com/
