# Supplementary material for: MS-H: A Novel Proteomic Approach to Isolate and Type the E. coli H Antigen Using Membrane Filtration and Liquid Chromatography-Tandem Mass Spectrometry (LC-MS/MS)
Source: PLoS One. 2013 Feb 21;8(2):e57339. doi: 10.1371/journal.pone.0057339 (PMC3578835; doi:10.1371/journal.pone.0057339)
Supplement: Representative Peptide Data S1 — Peptide data are represented as the Mascot search results from all 53 serotypes, obtained under the Orbitrap platform in Table 4 with related E. coli reference strains. “U” denotes a unique peptide specific for each of the proteins 1.1, 1.2, and beyond. The number 1.1 (shown as 1 in the peptide list and phylogenetic tree) represents the protein which obtained the highest score and confidence value after a Mascot search. This protein, known as the first hit, was used to designate the MS-H type of the unknown flagellin. Related peptides 1.2 (2), 1.3 (3), etc. represented the second, third, etc. hits for MS-H typing analysis. (DOCX) [file pone.0057339.s009.docx › H48-E247.pdf]

MASCOT Search Results

User :
E-mail :
Search title : Submitted from 20110824-0616 by Mascot Daemon on VARIABLE
MS data file : C:\Documents and Settings\keding\Desktop\Raw data\20110825-001-0013-00616\20110825-003-EC247-MS2.RAW
Database : Flagellin_v2 (192 sequences; 89,845 residues)
Taxonomy : Bacteria (Eubacteria) (192 sequences)
Timestamp : 26 Aug 2011 at 13:56:35 GMT

Not what you expected? Try **the select summary**.

▸ **Search parameters**
▸ **Score distribution**
▸ **Legend**

Protein Family Summary

Significance threshold p< 0.05 Max. number of families AUTO
Ions score or expect cut-off 0 Dendrograms cut at 0

Protein families 1–3 (out of 3)

10 per page 1

▾**1**

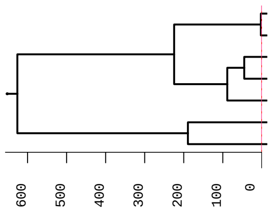

1	**gi|120312**	3275	Hxx(H48 100.0%) 0|0|0 [gi|6009847|ref|BAA85088| H48 0|H48|P4] [gi|30059922|ref|AAP133...
6	**gi|33590260**	879	H29 0|O157:H29|Y63
2	**gi|6941882**	1447	H14 0|O138:H14|O138:H14
5	**gi|5107847**	968	H12 0|O111:H12|DEC 6a
4	**gi|121144335**	1158	H55 0|O75:H55|E2987-73
3	**gi|154710458**	1336	H47 0|H47|0
7	**gi|46093563**	655	H54 0|0|E223-69

Threshold (**0**): 0

		Score	Mass	Matches	Sequences	emPAI
☑ 1.1	**gi|120312**	3275	51265	78 (63)	38 (32)	15.49
	Hxx(H48 100.0%) 0|0|0 [gi|6009847|ref|BAA85088| H48 0|H48|P4] [gi|30059922|ref|AAP13333| H48 0|H48|P4] [gi|1788232|ref|AAC74990| H48 0|0|K-12] [gi|89108758|ref|AP_002538| Hxx 0|0|...					
☑ 1.2	**gi|6941882**	1447	56492	50 (31)	30 (19)	3.12
	H14 0|O138:H14|O138:H14					
☑ 1.3	**gi|154710458**	1336	39177	30 (21)	15 (12)	2.11
	H47 0|H47|0					
☑ 1.4	**gi|121144335**	1158	62285	36 (25)	19 (14)	1.66
	H55 0|O75:H55|E2987-73					
☑ 1.5	**gi|5107847**	968	57823	40 (23)	23 (14)	1.86
	H12 0|O111:H12|DEC 6a					
☑ 1.6	**gi|33590260**	879	45720	32 (21)	17 (13)	2.27
	H29 0|O157:H29|Y63					
☑ 1.7	**gi|46093563**	655	54419	21 (10)	13 (7)	0.80
	H54 0|0|E223-69					

▾**128 peptide matches (88 non-duplicate, 40 duplicate)**

Query	Dupes	Observed	Mr(expt)	Mr(calc)	Delta	M	Score	Expect	Rank	U	1	2	3	4	5	6	7	Peptide
16		306.1470	610.2794	609.2758	1.0036	0	4	0.44	▸2	U				■				K.AYNDK.Y
24		309.1851	616.3556	616.3180	0.0376	0	1	1.5	▸1	U							■	K.IGGADGK.T
31	▸1	**316.6906**	**631.3666**	**631.3653**	**0.0013**	**0**	**33**	**0.0045**	▸1		■	■	■	■	■	■	■	**R.LSSGLR.I**
65		337.6900	673.3654	673.3647	0.0008	0	4	0.4	▸1	U	■							K.TTDPLK.A
94		351.7066	701.3986	702.3912	-0.9925	0	9	0.2	▸1	U						■		K.AIASVDK.F
104		355.1986	708.3826	708.3806	0.0020	0	14	0.29	▸1		■	■	■	■	■	■		R.FTSNIK.G
110		**358.7068**	**715.3990**	**715.3977**	**0.0014**	**0**	**31**	**0.0061**	▸1		■	■		■	■	■		**K.GLTQAAR.N**
112		359.2114	716.4082	715.4228	0.9854	0	0	1	▸1	U					■			K.ITIGGQK.A
144	▸4	**380.6963**	**759.3780**	**759.3763**	**0.0018**	**0**	**22**	**0.035**	▸1		■	■	■	■	■	■		**R.LDEIDR.V**
144	▸4	380.6963	759.3780	758.3922	0.9858	0	12	0.37	▸2	U							■	R.LNEIDR.V
167		387.7187	773.4228	774.4487	-1.0259	0	3	1.2	▸1	U			■					K.IDSTVLK.L
200	▸1	401.7163	801.4180	801.4708	-0.0528	1	3	0.51	▸1	U					■			K.VATAKQGK.A
252		419.2057	836.3968	835.4440	0.9529	0	2	0.66	▸1	U							■	K.VEGGYALK.V
323		438.7073	875.4000	875.3985	0.0016	0	8	0.15	▸1	U	■							K.ITGGDNDGK.Y
332		444.7375	887.4604	888.4916	-1.0312	0	10	0.2	▸1	U		■						K.AATTADVLK.A
358		452.7352	903.4558	904.4502	-0.9943	0	0	1.4	▸2	U		■						K.AATTADDLK.A
404	▸2	**466.2516**	**930.4886**	**930.4883**	**0.0004**	**0**	**82**	**2.6e-08**	▸1		■	■		■		■		**R.SSLGAVQNR.L**
501		489.3153	976.6160	976.5077	0.1084	0	1	0.72	▸1	U							■	K.TETVTIGEK.T
548	▸1	**502.2619**	**1002.5092**	**1002.5094**	**-0.0002**	**1**	**38**	**0.001**	▸1		■	■		■	■	■		**K.SRLDEIDR.V**
549		**335.1772**	**1002.5098**	**1002.5094**	**0.0004**	**1**	**39**	**0.00075**	▸1		■	■		■	■	■		**K.SRLDEIDR.V**
612		**518.7878**	**1035.5610**	**1035.5601**	**0.0010**	**0**	**51**	**8.3e-06**	▸1	U	■							**K.TLGLDGFSVK.N**
711		**549.2592**	**1096.5038**	**1096.5037**	**0.0002**	**0**	**49**	**1.3e-05**	▸1	U	■							**K.GNDTDTYALK.D**
724		**551.2682**	**1100.5218**	**1100.5210**	**0.0008**	**0**	**70**	**9.4e-07**	▸1		■	■	■	■	■	■	■	**K.DDAAGQAIANR.F**

Query	Dupes	Observed	Mr(expt)	Mr(calc)	Delta	M	Score	Expect	Rank	U	1	2	3	4	5	6	7	Peptide
752	1	559.2908	1116.5670	1116.5663	0.0008	0	84	4e-09	1	U	■							K.ALDDAIASVDK.F
835		581.2999	1160.5852	1159.5179	1.0673	1	6	0.33	1	U							■	K.MTYTDSNGKK.V + Oxidation (M)
890	1	596.3027	1190.5908	1190.5891	0.0018	0	58	8.6e-06	1		■	■			■			K.NQSALSSSIER.L
943		406.8587	1217.5543	1217.5888	-0.0345	0	2	0.64	1	U						■		R.VTIDGDGTNQAK.I
944		609.7863	1217.5580	1217.5888	-0.0308	0	15	0.033	1	U						■		R.VTIDGDGTNQAK.I
1006		624.3737	1246.7328	1247.6734	-0.9406	1	3	1.2	1	U					■			K.FRSSLGAIQNR.L
1055		427.5636	1279.6690	1279.7136	-0.0446	1	2	0.97	2	U		■						K.LYIDTTGRLTK.N
1118		656.8651	1311.7156	1311.7146	0.0010	0	58	1.6e-06	1	U					■			K.AQIIQQAGNSVLA.-
1129		658.8250	1315.6354	1315.6296	0.0059	0	55	3.5e-06	1	U			■					K.FDTTSEAAISFK.D
1173		672.8787	1343.7428	1343.7408	0.0020	0	71	7.2e-08	1	U				■				-.SLSLITQNNINK.N
1246		703.3699	1404.7252	1405.7677	-1.0425	1	2	4.7	1		■	■		■	■	■		R.FTSNIKGLTQAAR.N
1261		710.8757	1419.7368	1419.7358	0.0011	1	92	6.1e-10	1	U	■							K.ALDDAIASVDKFR.S
1262		474.2535	1419.7387	1419.7358	0.0029	1	45	3.2e-05	1	U	■							K.ALDDAIASVDKFR.S
1298	1	720.9133	1439.8120	1439.8096	0.0024	0	105	1.4e-10	1		■	■		■				K.AQIIQQAGNSVLAK.A
1377		747.9209	1493.8272	1493.8202	0.0071	0	58	9.6e-06	1		■	■				■		K.ANQVPQQVLSLLQG.-
1476	2	781.4212	1560.8278	1560.8260	0.0018	0	70	4.7e-07	1		■	■		■	■	■		R.VSGQTQFNGVNVLAK
1531		538.9442	1613.8108	1613.8121	-0.0013	1	42	0.00053	1		■	■	■	■	■	■	■	R.INSAKDDAAGQAIANR.F
1532		807.9132	1613.8118	1613.8121	-0.0003	1	100	8.3e-10	1		■	■	■	■	■	■	■	R.INSAKDDAAGQAIANR.F
1537		539.9365	1616.7877	1616.7894	-0.0017	1	45	3.3e-05	1	U	■							K.LGGDDGKTEVVDIDGK.T
1538		809.4016	1616.7886	1616.7894	-0.0007	1	88	1.6e-09	1	U	■							K.LGGDDGKTEVVDIDGK.T
1544		811.4181	1620.8216	1620.7818	0.0399	1	0	0.99	1	U					■			K.KTASVTMGGTTYNFK.T + Oxidation (M)
1550		544.0636	1629.1690	1627.8529	1.3161	1	7	0.21	1	U				■				K.DVDNAKAAASNVLAAAK.N
1605		556.9415	1667.8027	1667.8002	0.0024	1	38	0.00016	1	U	■							K.LQDSKGNDTDTYALK.D
1608		836.3815	1670.7484	1670.7457	0.0027	0	132	4.3e-13	1		■	■		■	■	■		R.IQDADYATEVSNMSK.A
1633		844.3798	1686.7450	1686.7407	0.0044	0	120	6.9e-12	1		■	■		■	■	■		R.IQDADYATEVSNMSK.A + Oxidation (M)
1653		570.0898	1707.2476	1705.8734	1.3742	1	1	0.88	2	U		■						K.LTTDAETKAATTADGLK.A
1673		573.4146	1717.2220	1715.7308	1.4912	0	2	1.5	1	U							■	R.IEDADYATEVSNMSR.A + Oxidation (M)
1708	8	871.4590	1740.9034	1740.9006	0.0028	0	99	1.2e-10	1	U			■					K.IQVGANDNQSIDINLK.K
1709	8	871.4590	1740.9034	1741.9210	-1.0176	0	62	5.8e-07	2	U	■							K.IQVGANDNQTITIDLK.Q
1727		584.9471	1751.8195	1751.8611	-0.0416	1	8	0.18	1	U		■						K.LTTDAETKAATTADCLK.A
1755	1	878.9335	1755.8524	1755.8527	-0.0002	0	115	6.7e-12	1	U	■							K.TITYTDSSGAASSPTAVK.L
1774		591.6493	1771.9261	1771.9203	0.0057	1	53	6.9e-06	1	U	■							K.TTDPLKALDDAIASVDK.F
1775		886.9714	1771.9282	1771.9203	0.0079	1	48	2.1e-05	1	U	■							K.TTDPLKALDDAIASVDK.F
1785		594.2772	1779.8098	1779.8924	-0.0826	1	16	0.027	1	U		■						K.LTTDAETKAATTADMLK.A
1804		597.9348	1790.7826	1790.8911	-0.1085	1	0	7.8	1		■	■	■	■	■	■		K.DDAAGQAIANRFTSNIK.G
1833		902.4535	1802.8924	1803.9438	-1.0514	1	1	3.8	1		■	■			■			K.NQSALSSSIERLSSGLR.I
1924		624.0086	1869.0040	1868.9956	0.0084	1	26	0.0024	1	U			■					K.IQVGANDNQSIDINLKK.I
1949		631.4564	1891.3474	1890.9171	0.4303	0	7	0.19	1	U		■						K.GTITIDGSAQDVQISSDGK.I
2044		997.5035	1992.9924	1992.9865	0.0060	0	148	3.7e-15	1	U			■					R.FDSAITNLGNTVNNLSSAR.S
2051		502.0259	2004.0745	2003.0422	1.0323	1	1	0.77	1	U		■						K.AATTADALKALDEAISSIDK.F
2071		1021.4720	2040.9294	2040.9236	0.0058	0	1	0.88	1	U					■			K.TGADADAATANAGVSFTDTASK.E
2096		1043.0700	2084.1254	2084.1225	0.0029	0	142	4.6e-14	1		■	■	■			■	■	M.AQVINTNSLSLiTQNNiNK.N
2097		695.7164	2084.1274	2084.1225	0.0048	0	80	6.1e-08	1		■	■	■			■	■	M.AQVINTNSLSLiTQNNiNK.N
2134	1	1070.0160	2138.0174	2138.0128	0.0047	0	108	1.7e-11	1	U	■							K.DTNGNLYAADVNETTGAVSVK.T
2196	1	1125.0560	2248.0974	2248.0931	0.0043	0	138	1.1e-13	1		■	■		■	■			R.LDSAVTNLNNTTTNLSEAQSR.I
2197		750.3732	2248.0978	2248.0931	0.0047	0	82	4e-08	1		■	■		■	■			R.LDSAVTNLNNTTTNLSEAQSR.I
2227		1150.0950	2298.1754	2298.1703	0.0051	0	85	3.2e-09	1	U							■	K.AALATDVNNASSIGVSDAIPGDIK.F
2230		768.4050	2302.1932	2302.1917	0.0014	1	50	4.4e-05	1		■	■		■	■	■		R.LDEIDRVSGQTQFNGVNVLAK
2247		780.0565	2337.1477	2337.1449	0.0028	0	75	3.3e-08	1	U	■							K.NNDTVTTSAPVTAFGATTTNNIK.L
2248		1169.5820	2337.1494	2337.1449	0.0046	0	98	1.4e-10	1	U	■							K.NNDTVTTSAPVTAFGATTTNNIK.L
2295		856.0827	2565.2263	2565.2293	-0.0031	0	39	0.00036	1	U	■							R.ELTVQATTGTNSESDLSSIQDEIK.S
2295		856.0827	2565.2263	2565.1930	0.0333	0	16	0.078	3			■			■			R.ELTVQASTGTNSDSDLDSIQDEIK.S
2296		1283.6250	2565.2354	2565.2293	0.0061	0	148	5.4e-15	1	U	■							R.ELTVQATTGTNSESDLSSIQDEIK.S
2296		1283.6250	2565.2354	2565.1930	0.0425	0	22	0.02	3			■			■			R.ELTVQASTGTNSDSDLDSIQDEIK.S
2302		1308.6050	2615.1954	2615.1835	0.0120	0	84	4.3e-09	1	U			■					K.STTTDNNGIYAASVDSDGNVTIDASK.K
2304		877.1006	2628.2800	2628.2739	0.0061	0	25	0.015	1		■	■		■	■		■	R.NANDGISVAQTTEGALSEINNNLQR
2305		1315.1480	2628.2814	2628.2739	0.0075	0	136	1.1e-13	1		■	■		■	■		■	R.NANDGISVAQTTEGALSEINNNLQR
2321		915.4384	2743.2934	2743.2784	0.0149	1	72	6.9e-08	1	U			■					K.STTTDNNGIYAASVDSDGNVTIDASKK.V
2331	2	960.4745	2878.4017	2878.3945	0.0072	0	85	3e-09	1	U	■							K.TYDSADLNGGNLQTGLTAGGEALTAVANGK.T
2332	1	1440.2090	2878.4034	2878.3945	0.0090	0	136	2.3e-14	1	U	■							K.TYDSADLNGGNLQTGLTAGGEALTAVANGK.T
2342		972.5184	2914.5334	2914.5248	0.0086	0	78	6.1e-08	1	U	■							K.ATTITSGGTPVQIDNTAGSATANLGAVSLVK.L
2343		1458.2740	2914.5334	2914.5248	0.0087	0	105	1.4e-10	1	U	■							K.ATTITSGGTPVQIDNTAGSATANLGAVSLVK.L
2385		1077.5720	3229.6942	3229.6902	0.0040	1	117	2.9e-12	1				■				■	M.AQVINTNSLSLLTQNNLNKSQSSLSSAIER.L
2388		1086.5770	3256.7092	3256.7011	0.0081	1	104	1.7e-10	1		■	■						M.AQVINTNSLSLITQNNINKNQSALSSSIER.L
2458		1227.5790	3679.7152	3679.7013	0.0138	0	87	2.2e-09	1	U	■							K.LTGITLSTEAATDTGGTNPASIEGVYTDNGNDYYAK.I

61 subsets and intersections (165 subset proteins in total)

2	gi|307553085	21	Hxx(H54 27.9%) 0|0|ABU 83972
3	gi|112820172	14	H21 0|EHEC serogroup: O113:H21|0

10 per page 1

Not what you expected? Try the select summary.

Mascot: http://www.matrixscience.com/
