# Supplementary material for: MS-H: A Novel Proteomic Approach to Isolate and Type the E. coli H Antigen Using Membrane Filtration and Liquid Chromatography-Tandem Mass Spectrometry (LC-MS/MS)
Source: PLoS One. 2013 Feb 21;8(2):e57339. doi: 10.1371/journal.pone.0057339 (PMC3578835; doi:10.1371/journal.pone.0057339)
Supplement: Representative Peptide Data S1 — Peptide data are represented as the Mascot search results from all 53 serotypes, obtained under the Orbitrap platform in Table 4 with related E. coli reference strains. “U” denotes a unique peptide specific for each of the proteins 1.1, 1.2, and beyond. The number 1.1 (shown as 1 in the peptide list and phylogenetic tree) represents the protein which obtained the highest score and confidence value after a Mascot search. This protein, known as the first hit, was used to designate the MS-H type of the unknown flagellin. Related peptides 1.2 (2), 1.3 (3), etc. represented the second, third, etc. hits for MS-H typing analysis. (DOCX) [file pone.0057339.s009.docx › H49-E248.pdf]

MATRIX SCIENCE MASCOT Search Results

User :
E-mail :
Search title : Submitted from 20110824-0616 by Mascot Daemon on VARIABLE
MS data file : C:\Documents and Settings\keding\Desktop\Raw data\20110825-001-0013-00616\20110825-007-EC248-MS2.RAW
Database : Flagellin_v2 (192 sequences; 89,845 residues)
Taxonomy : Bacteria (Eubacteria) (192 sequences)
Timestamp : 26 Aug 2011 at 14:03:33 GMT

Not what you expected? Try **the select summary**.

▸**Search parameters**

▸**Score distribution**

▸**Legend**

Protein Family Summary

Significance threshold p< 0.05 Max. number of families AUTO
Ions score or expect cut-off 0 Dendrograms cut at 0

Protein families 1–3 (out of 3)

10 per page 1

▾1

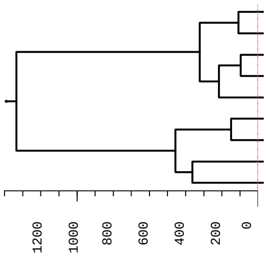

1	gi|30059924	4259	H49 0|H49|2147-59 [gi|315061223|ref|ADT75550| Hxx 0|0|W] [gi|323186300|ref|EFZ71650| H...
7	gi|5107837	994	H7 0|O128:H7|DEC 13a
4	gi|120312	1846	Hxx(H48 100.0%) 0|0|0 [gi|6009847|ref|BAA85088| H48 0|H48|P4] [gi|30059922|ref|AAP13333...
6	gi|121144335	1090	H55 0|O75:H55|E2987-73
5	gi|300399814	1740	Hxx(H6 98.7%) 0|0|MS 69-1 [gi|301018227|ref|ZP_07182742| Hxx 0|0|MS 69-1]
2	gi|112820171	2565	H21 0|EHEC serogroup: O113:H21|0 [gi|291323135|ref|EFE62563| Hxx 0|0|B088] [gi|29344630...
9	gi|40353143	939	H2 0|O111:H2|5721/96
3	gi|154710458	1964	H47 0|H47|0
8	gi|46093563	987	H54 0|0|E223-69

Threshold (**0**): 0

		Score	Mass	Matches	Sequences	emPAI
☑ 1.1	gi|30059924	4259	57964	94 (83)	43 (41)	22.30
	H49 0|H49|2147-59 [gi|315061223|ref|ADT75550| Hxx 0|0|W] [gi|323186300|ref|EFZ71650| Hxx 0|0|1357] [gi|307314170|ref|ZP_07593780| Hxx 0|0|W] [gi|323378200|ref|ADX50468| Hxx 0|0|KO...					
☑ 1.2	gi|112820171	2565	51472	55 (48)	26 (23)	5.43
	H21 0|EHEC serogroup: O113:H21|0 [gi|291323135|ref|EFE62563| Hxx 0|0|B088] [gi|293446305|ref|ZP_06662727| Hxx 0|0|B088]					
☑ 1.3	gi|154710458	1964	39177	43 (37)	16 (15)	2.97
	H47 0|H47|0					
☑ 1.4	gi|120312	1846	51265	51 (45)	21 (19)	4.05
	Hxx(H48 100.0%) 0|0|0 [gi|6009847|ref|BAA85088| H48 0|H48|P4] [gi|30059922|ref|AAP13333| H48 0|H48|P4] [gi|1788232|ref|AAC74990| H48 0|0|K-12] [gi|89108758|ref|AP_002538| Hxx 0|0|K-...					
	▸1 sameset of gi|120312					
☑ 1.5	gi|300399814	1740	56260	44 (37)	23 (18)	3.38
	Hxx(H6 98.7%) 0|0|MS 69-1 [gi|301018227|ref|ZP_07182742| Hxx 0|0|MS 69-1]					
☑ 1.6	gi|121144335	1090	62285	29 (25)	18 (14)	1.80
	H55 0|O75:H55|E2987-73					
☑ 1.7	gi|5107837	994	56230	28 (23)	15 (13)	1.78
	H7 0|O128:H7|DEC 13a					
☑ 1.8	gi|46093563	987	54419	22 (19)	15 (12)	1.42
	H54 0|0|E223-69					
☑ 1.9	gi|40353143	939	47290	30 (26)	19 (16)	2.85
	H2 0|O111:H2|5721/96					

▾209 peptide matches (118 non-duplicate, 91 duplicate)

Query	Dupes	Observed	Mr(expt)	Mr(calc)	Delta	M	Score	Expect	Rank	U	1	2	3	4	5	6	7	8	9	Peptide
19		316.6908	631.3670	631.3653	0.0017	0	24	0.042	▸1		■	■	■	■	■	■	■	■	■	R.LSSGLR.I
54		355.1989	708.3832	708.3806	0.0026	0	15	0.2	▸1		■		■	■	■	■	■			R.FTSNIK.G
56		358.7072	715.3998	715.3977	0.0022	0	23	0.033	▸1		■			■	■	■	■			K.GLTQAAR.N
75		380.6961	759.3776	759.3763	0.0014	0	36	0.0015	▸1		■		■	■	■	■	■			R.LDEIDR.V
75		380.6961	759.3776	758.3922	0.9854	0	18	0.1	▸2	U								■		R.LNEIDR.V
222		445.3270	888.6394	888.5102	0.1292	1	2	1.1	▸1	U							■			K.ADMKALLK.A
270		466.7439	931.4732	930.4883	0.9850	0	12	0.29	▸1					■	■	■				R.SSLGAVQNR.L
290	▸2	473.2598	944.5050	944.5039	0.0011	0	74	1.1e-07	▸1		■						■			R.SSLGAIQNR.L
322		480.2491	958.4836	958.4832	0.0004	0	40	0.00019	▸1			■	■					■	■	R.SDLGAVQNR.F
388		502.2626	1002.5106	1002.5094	0.0012	1	49	7.6e-05	▸1		■			■	■	■	■			K.SRLDEIDR.V
389		335.1783	1002.5131	1002.5094	0.0037	1	29	0.0068	▸1		■			■	■	■	■			K.SRLDEIDR.V
460		529.3040	1056.5934	1056.5927	0.0007	0	34	0.00043	▸1	U									■	-.LLTQNNLNK.S
467	▸1	530.7675	1059.5204	1059.5197	0.0008	0	80	1.1e-08	▸1	U	■									K.DGDSVAVAAQK.Y
529		551.2683	1100.5220	1100.5210	0.0010	0	72	6e-07	▸1		■	■	■	■	■	■		■	■	K.DDAAGQAIANR.F
595		380.4978	1138.4716	1139.5459	-1.0743	0	2	0.65	▸2	U					■					K.NDTYTATVGAK.T
648		582.7964	1163.5782	1163.5782	0.0000	0	42	0.00021	▸1			■	■					■	■	K.SQSSLSSAIER.L
685		397.2044	1188.5914	1187.6034	0.9880	0	2	0.7	▸1	U						■				K.ALDDAISQIDK.F

Query	Dupes	Observed	Mr(expt)	Mr(calc)	Delta	M	Score	Expect	Rank	U	1	2	3	4	5	6	7	8	9	Peptide
690		596.3028	1190.5910	1190.5891	0.0020	0	60	5.4e-06	▶1		■			■	■					K.NQSALSSSIER.L
694		598.2830	1194.5514	1194.5517	-0.0002	0	71	7.2e-08	▶1	U	■									K.DAAQSSIDFGGK.K
762		618.8159	1235.6172	1235.6146	0.0026	0	46	6.1e-05	▶1			■							■	R.VSEQTQFNGVK.V
795	▶1	628.3210	1254.6274	1254.6244	0.0030	0	77	2.2e-08	▶1	U	■									K.FNALDAATAFSK.L
804		630.3647	1258.7148	1259.6106	-0.8957	0	2	0.7	▶1	U					■					K.NNAGGDTQATLAK.L
843		641.8284	1281.6422	1281.6412	0.0010	0	68	2.3e-07	▶1	U	■									K.LTTNTTSTGSATK.D
856		647.3192	1292.6238	1292.6209	0.0030	0	102	5.7e-11	▶1	U	■									K.DTGAVSVTGGSGTGK.Y
868		651.3574	1300.7002	1300.6987	0.0016	1	72	1.3e-07	▶1	U	■									K.LKDGDSVAVAAQK.Y
869	▶1	434.5742	1300.7008	1300.6987	0.0021	1	36	0.00057	▶1	U	■									K.LKDGDSVAVAAQK.Y
874		651.8638	1301.7130	1301.6827	0.0304	0	0	2.1	▶2			■							■	K.AATLSDLDLNAAK.K
889		656.8651	1311.7156	1311.7146	0.0010	0	84	3.9e-09	▶1	U							■			K.AQIIQQAGNSVLA.-
904		658.8254	1315.6362	1315.6296	0.0067	0	34	0.00043	▶1	U			■							K.FDTTSEAAISFK.D
940		672.8790	1343.7434	1343.7408	0.0026	0	72	5.8e-08	▶1	U						■				-.SLSLITQNNINK.N
943		673.8633	1345.7120	1345.7089	0.0031	0	66	2.6e-07	▶1	U	■									K.DTVSSDALLAQVK.A
1066	▶2	720.9131	1439.8116	1439.8096	0.0020	0	107	9.9e-11	▶1		■			■	■	■				K.AQIIQQAGNSVLAK.A
1091	▶1	729.3368	1456.6590	1456.6583	0.0008	0	90	1.7e-09	▶1	U	■									K.YEFAGGNSTNGGGVK.F
1118		491.3531	1471.0375	1471.6943	-0.6569	1	14	0.042	▶1	U									■	K.FKATGTDNYDVGGK.T
1137		743.8740	1485.7334	1485.7311	0.0024	0	61	1.3e-06	▶1			■							■	K.SEGGSPILVNEDAAK.S
1150	▶2	747.9203	1493.8260	1493.8202	0.0059	0	66	1.5e-06	▶1		■			■	■					K.ANQVPQQVLSLLQG.-
1246	▶4	777.8858	1553.7570	1553.7474	0.0096	0	77	2.2e-08	▶1	U	■									K.YAANVGAQAYVGADGK.L
1254	▶2	781.4216	1560.8286	1560.8260	0.0026	0	69	5.6e-07	▶1		■			■		■	■			R.VSGQTQFNGVNVLAK
1285		792.9145	1583.8144	1583.8042	0.0102	0	110	9.5e-12	▶1	U	■									K.DPLNALDEAIASIDK.F
1286	▶2	529.2587	1584.7543	1584.7532	0.0010	1	25	0.0033	▶1	U	■									K.KYEFAGGNSTNGGGVK.F
1287	▶1	793.3856	1584.7566	1584.7532	0.0034	1	80	9.2e-09	▶1	U	■									K.KYEFAGGNSTNGGGVK.F
1319		538.9449	1613.8129	1613.8121	0.0008	1	25	0.028	▶1		■	■	■	■	■	■		■	■	R.INSAKDDAAGQAIANR.F
1320		807.9152	1613.8158	1613.8121	0.0037	1	56	2.2e-05	▶1		■	■	■	■	■	■		■	■	R.INSAKDDAAGQAIANR.F
1331	▶1	541.2983	1620.8731	1620.8723	0.0008	1	30	0.00089	▶1	U	■									K.FKDTVSSDALLAQVK.A
1332		811.4442	1620.8738	1620.8723	0.0016	1	105	3.3e-11	▶1	U	■									K.FKDTVSSDALLAQVK.A
1380	▶2	829.8940	1657.7770	1657.7696	0.0038	0	102	6.5e-11	▶1	U	■									K.AAAGQSQSGTYTFANGK.V
1381		553.5989	1657.7749	1657.7696	0.0053	0	10	0.096	▶1	U	■									K.AAAGQSQSGTYTFANGK.V
1390	▶1	832.4199	1662.8252	1662.8213	0.0039	0	90	1.7e-09	▶1		■				■		■			K.IDSDTLGLNGFNVNGK.G
1402		557.9236	1670.7490	1670.7457	0.0032	0	36	0.0014	▶1		■			■	■	■	■			R.IQDADYATEVSNMSK.A
1403		836.3826	1670.7506	1670.7457	0.0049	0	130	6.4e-13	▶1		■			■	■	■	■			R.IQDADYATEVSNMSK.A
1431		843.4607	1684.9068	1684.8996	0.0073	0	93	1.8e-09	▶1	U	■									K.IQVGANDGQTITIDLK.K
1431		843.4607	1684.9068	1685.8836	-0.9767	0	55	1.1e-05	▶2						■	■	■			K.IQVGANDGETITIDLK.K
1434		844.3802	1686.7458	1686.7407	0.0052	0	106	1.7e-10	▶1		■			■	■	■	■			R.IQDADYATEVSNMSK.A + Oxidation (M)
1451	▶1	565.9333	1694.7781	1694.7748	0.0033	1	45	2.9e-05	▶1	U	■									K.AADDKDAAQSSIDFGGK.K
1452		848.3967	1694.7788	1694.7748	0.0041	1	99	1.4e-10	▶1	U	■									K.AADDKDAAQSSIDFGGK.K
1458		850.8768	1699.7390	1699.7359	0.0031	0	93	7.6e-10	▶1			■						■		R.IEDADYATEVSNMSR.A
1513		579.4050	1735.1932	1734.8425	0.3507	0	3	0.5	▶1	U	■									K.VNFDVDASGNITIGGEK.A
1531	▶12	871.4607	1740.9068	1740.9006	0.0062	0	101	7.8e-11	▶1	U			■							K.IQVGANDNQSIDINLK.K
1531	▶12	871.4607	1740.9068	1741.9210	-1.0142	0	55	3.5e-06	▶2	U				■						K.IQVGANDNQTITIDLK.Q
1567	▶1	878.9772	1755.9398	1755.9367	0.0032	0	74	8.9e-08	▶1			■							■	K.IQVGANDGETITINLAK.I
1586		592.3050	1773.8932	1774.8962	-1.0030	1	3	1.5	▶1			■						■	■	K.DDAAGQAIANRFTANIK.G
1600		596.9639	1787.8699	1786.7679	1.1019	1	10	0.1	▶1	U									■	R.SRIEDADYATEVSNMS.-
1611	▶2	896.4670	1790.9194	1790.9163	0.0032	1	104	3e-10	▶1		■				■		■			K.KIDSDTLGLNGFNVNGK.G
1612	▶1	597.9814	1790.9224	1790.9163	0.0061	1	52	5.5e-05	▶1		■				■		■			K.KIDSDTLGLNGFNVNGK.G
1693	▶1	927.9589	1853.9032	1853.9007	0.0025	0	95	5.1e-10	▶1	U		■								K.TGSTLVVNGATYDVSADGK.T
1693		927.9589	1853.9032	1852.9167	0.9866	0	62	1.1e-06	▶2	U									■	K.TGSTLVVNGATYNVSADGK.T
1694		619.6525	1855.9357	1855.9349	0.0007	1	33	0.00066	▶1	U		■								K.TITETASGNNKVMYLSK.S
1695		928.9770	1855.9394	1855.9349	0.0045	1	40	0.00014	▶1	U		■								K.TITETASGNNKVMYLSK.S
1697		465.2161	1856.8353	1855.9349	0.9004	1	2	0.97	▶1	U		■								K.TITETASGNNKVMYLSK.S
1716	▶3	624.0083	1869.0031	1868.9956	0.0075	1	37	0.00021	▶1	U			■							K.IQVGANDNQSIDINLKK.I
1739		944.4994	1886.9842	1886.9738	0.0105	1	86	2.8e-09	▶1	U	■									K.DPLNALDEAIASIDKFR.S
1740		630.0023	1886.9851	1886.9738	0.0113	1	79	1.2e-08	▶1	U	■									K.DPLNALDEAIASIDKFR.S
1790		648.6323	1942.8751	1942.8690	0.0060	1	53	8.7e-06	▶1			■						■		R.SRIEDADYATEVSNMSR.A
1811		992.0063	1981.9980	1981.9957	0.0024	1	117	4.2e-12	▶1	U		■								K.KTGSTLVVNGATYDVSADGK.T
1811		992.0063	1981.9980	1981.0116	0.9864	1	23	0.012	▶2	U									■	K.KTGSTLVVNGATYNVSADGK.T
1812		661.6736	1981.9990	1981.9957	0.0033	1	56	5.4e-06	▶1	U		■								K.KTGSTLVVNGATYDVSADGK.T
1812		661.6736	1981.9990	1981.0116	0.9873	1	39	0.00024	▶2	U									■	K.KTGSTLVVNGATYNVSADGK.T
1820		664.6746	1991.0020	1990.9960	0.0060	1	43	9e-05	▶1			■							■	R.LEEIDRVSEQTQFNGVK.V
1821		996.5083	1991.0020	1990.9960	0.0061	1	76	4.4e-08	▶1			■							■	R.LEEIDRVSEQTQFNGVK.V
1822		997.5040	1992.9934	1992.9865	0.0070	0	108	3.6e-11	▶1			■	■						■	R.FDSAITNLGNTVNNLSSAR.S
1860	▶4	1021.0340	2040.0534	2040.0415	0.0119	0	135	3.2e-14	▶1	U		■								K.IVYEGIEFTNTGTVAIDAK.G
1865		1030.5070	2058.9994	2058.9957	0.0038	0	3	0.58	▶1	U					■					K.AESTSDPLAALDDAISQIDK.F
1878	▶1	695.7167	2084.1283	2084.1225	0.0057	0	82	4e-08	▶1		■	■	■	■	■			■		M.AQVINTNSLSLiTQNNiNK.N
1880	▶2	1043.0730	2084.1314	2084.1225	0.0089	0	106	1.7e-10	▶1		■	■	■	■	■			■		M.AQVINTNSLSLiTQNNiNK.N
1918	▶1	1068.0150	2134.0154	2134.0066	0.0088	0	154	4.3e-16	▶1	U		■								K.QVYVSTADGSLTTSSDTQFK.I
1968	▶1	1125.0570	2248.0994	2248.0931	0.0063	0	117	1.3e-11	▶1		■			■	■	■				R.LDSAVTNLNNTTTNLSEAQSR.I
1969		750.3743	2248.1011	2248.0931	0.0080	0	79	8.2e-08	▶1		■			■	■	■				R.LDSAVTNLNNTTTNLSEAQSR.I
2000		1150.0960	2298.1774	2298.1703	0.0071	0	74	4.3e-08	▶1	U								■		K.AALATDVNNASSIGVSDAIPGDIK.F
2002		768.4057	2302.1953	2302.1917	0.0035	1	57	8.5e-06	▶1		■			■		■	■			R.LDEIDRVSGQTQFNGVNVLAK
2030	▶1	1210.1400	2418.2654	2418.2602	0.0053	0	128	1.5e-13	▶1	U	■									K.AATISDLAATGANVTNSSNIVVTTK.F
2036		509.2531	2541.2291	2540.2395	0.9896	1	9	0.12	▶1	U						■				K.LAGFTAGATPAADGTVTYSKDVDNAK.A
2044	▶1	1283.6080	2565.2014	2565.1930	0.0085	0	132	2e-13	▶1	U	■									R.ELTVQASTGTNSDSDLDSIQDEIK.S
2045	▶1	1283.6270	2565.2394	2565.2293	0.0101	0	150	2.8e-15	▶1	U				■						R.ELTVQATTGTNSESDLSSIQDEIK.S
2045	▶1	1283.6270	2565.2394	2565.2294	0.0101	0	80	3.4e-08	▶2	U					■					R.ELTVQATTGTNSTSDLDSIQDEIK.S
2047		1292.6560	2583.2974	2583.2891	0.0084	0	59	1.2e-06	▶1	U	■									K.AFLVGGALTTNDPTGSTPATMSSLFK.A
2048		862.1076	2583.3010	2583.2891	0.0119	0	71	7.3e-08	▶1	U	■									K.AFLVGGALTTNDPTGSTPATMSSLFK.A
2050		1300.1440	2598.2734	2598.2634	0.0101	0	73	4.7e-08	▶1	U			■							R.NANDGISVAQTTEGALGEINNNLQR.I
2051		1300.6520	2599.2894	2599.2840	0.0055	0	92	6.7e-10	▶1	U	■									K.AFLVGGALTTNDPTGSTPATMSSLFK.A + Oxidation
2052	▶1	867.4379	2599.2919	2599.2840	0.0079	0	81	7.7e-09	▶1	U	■									K.AFLVGGALTTNDPTGSTPATMSSLFK.A + Oxidation
2053		867.4606	2599.3600	2598.2634	1.0966	0	2	0.65	▶2	U			■							R.NANDGISVAQTTEGALGEINNNLQR.I

Query	Dupes	Observed	Mr(expt)	Mr(calc)	Delta	M	Score	Expect	Rank	U	1	2	3	4	5	6	7	8	9	Peptide
2055	▶3	1308.6010	2615.1874	2615.1835	0.0040	0	85	3e-09	▶1	U			■							K.STTTDNNGIYAASVDSDGNVTIDASK.K
2061		877.1005	2628.2797	2628.2739	0.0058	0	70	4.3e-07	▶1		■			■	■	■	■	■		R.NANDGISVAQTTEGALSEINNNLQR
2062		1315.1480	2628.2814	2628.2739	0.0075	0	131	3.6e-13	▶1		■			■	■	■	■	■		R.NANDGISVAQTTEGALSEINNNLQR
2071		888.1725	2661.4957	2662.2974	-0.8017	1	5	0.33	▶1	U		■								K.QVYVSTADGSLTTSSDTQFKIDATK.L
2079	▶1	892.7820	2675.3242	2675.3178	0.0064	0	30	0.0011	▶1	U		■								K.AVEFTISGSTDTSGTSATVAPTTALYK.N
2081	▶2	1338.6710	2675.3274	2675.3178	0.0097	0	80	1e-08	▶1	U		■								K.AVEFTISGSTDTSGTSATVAPTTALYK.N
2093	▶3	915.4391	2743.2955	2743.2784	0.0170	1	75	3.1e-08	▶1	U			■							K.STTTDNNGIYAASVDSDGNVTIDASKK.V
2099	▶1	1382.6710	2763.3274	2763.3159	0.0116	0	105	4.4e-11	▶1			■							■	R.ELSVQATNGTNSDSDLSSIQAEITQR.L
2102	▶4	922.1171	2763.3295	2763.3159	0.0136	0	62	8e-07	▶1			■							■	R.ELSVQATNGTNSDSDLSSIQAEITQR.L
2105		950.1552	2847.4438	2847.4349	0.0089	1	64	6.9e-07	▶1	U	■									K.LTTNTTSTGSATKDPLNALDEAIASIDK.F
2116		972.5178	2914.5316	2914.5248	0.0068	0	33	0.0019	▶1	U				■						K.ATTITSGGTPVQIDNTAGSATANLGAVSLVK.L
2125	▶1	1011.8440	3032.5102	3032.5010	0.0092	1	90	1.9e-09	▶1			■						■	■	R.iRELsVQATNGTNSDSDLSSIQAEITQR.L
2135		1048.1650	3141.4732	3141.4626	0.0106	0	68	1.6e-07	▶1	U	■									K.YTYNASTNDFTTENTVATGTATTDLGATLK.A
2135		1048.1650	3141.4732	3140.6201	0.8531	1	2	0.61	▶2	U								■		K.QATLGEEATTTTVNPLDAIDKALAQVDSLR.S
2144		1059.2060	3174.5962	3174.5865	0.0097	1	103	1.1e-10	▶1	U	■									R.SSLGAIQNRLDSAVTNLNNTTTNLSEAQSR.I
2147	▶2	1063.4930	3187.4572	3187.4542	0.0030	1	120	1e-12	▶1	U		■								K.ATGTDNYQINGTDNYTVNVDSGVVQDKDGK.Q
2151		1077.5740	3229.7002	3229.6902	0.0100	1	88	2.4e-09	▶1			■	■					■		M.AQVINTNSLSLLTQNNLNKSQSSLSSAIER.L
2155		1086.5780	3256.7122	3256.7011	0.0111	1	94	1.4e-09	▶1		■			■	■					M.AQVINTNSLSLITQNNINKNQSALSSSIER.L

▶91 subsets and intersections (163 subset proteins in total)

▶2	gi|307553085	118	Hxx(H54 27.9%) 0|0|ABU 83972
▶3	gi|112820172	45	H21 0|EHEC serogroup: O113:H21|0

10 per page 1

Not what you expected? Try **the select summary**.

Mascot: http://www.matrixscience.com/
