# Supplementary material for: MS-H: A Novel Proteomic Approach to Isolate and Type the E. coli H Antigen Using Membrane Filtration and Liquid Chromatography-Tandem Mass Spectrometry (LC-MS/MS)
Source: PLoS One. 2013 Feb 21;8(2):e57339. doi: 10.1371/journal.pone.0057339 (PMC3578835; doi:10.1371/journal.pone.0057339)
Supplement: Representative Peptide Data S1 — Peptide data are represented as the Mascot search results from all 53 serotypes, obtained under the Orbitrap platform in Table 4 with related E. coli reference strains. “U” denotes a unique peptide specific for each of the proteins 1.1, 1.2, and beyond. The number 1.1 (shown as 1 in the peptide list and phylogenetic tree) represents the protein which obtained the highest score and confidence value after a Mascot search. This protein, known as the first hit, was used to designate the MS-H type of the unknown flagellin. Related peptides 1.2 (2), 1.3 (3), etc. represented the second, third, etc. hits for MS-H typing analysis. (DOCX) [file pone.0057339.s009.docx › H51-E372.pdf]

MASCOT Search Results

User :
E-mail :
Search title : Submitted from 20110901-0628 by Mascot Daemon on VARIABLE
MS data file : C:\Documents and Settings\keding\Desktop\Raw data\20110901-001-0031-00628\20110901-003-EC372-MS3rp.RAW
Database : Flagellin_v2 (192 sequences; 89,845 residues)
Taxonomy : Bacteria (Eubacteria) (192 sequences)
Timestamp : 3 Sep 2011 at 15:37:58 GMT

Not what you expected? Try **the select summary**.

▸ **Search parameters**
▸ **Score distribution**
▸ **Legend**

Protein Family Summary

Significance threshold p< 0.05 Max. number of families AUTO
Ions score or expect cut-off 0 Dendrograms cut at 0

Protein family 1 (out of 1)

10 per page 1

▾**1**

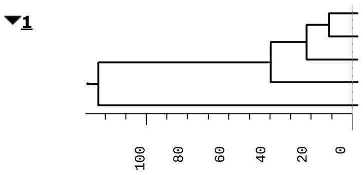

1	gi|6009851	2297	H51 0|H51|C218-70 [gi|30059926|ref|AAP13335| H51 0|H51|C218-70]
5	gi|33590243	325	H16 NM|O157:NM(H16)|PC02
4	gi|16901492	337	H8 0|H8|0
2	gi|30059924	959	H49 0|H49|2147-59 [gi|315061223|ref|ADT75550| Hxx 0|0|W] [gi|323186300|ref|EFZ...
3	gi|121144335	679	H55 0|O75:H55|E2987-73

Threshold (**0**): 0

		Score	Mass	Matches	Sequences	emPAI
1.1	**gi|6009851**	2297	61407	48 (43)	27 (26)	5.21

H51 0|H51|C218-70 [gi|30059926|ref|AAP13335| H51 0|H51|C218-70]

		Score	Mass	Matches	Sequences	emPAI
1.2	**gi|30059924**	959	57964	24 (18)	14 (12)	1.42

H49 0|H49|2147-59 [gi|315061223|ref|ADT75550| Hxx 0|0|W] [gi|323186300|ref|EFZ71650| Hxx 0|0|1357] [gi|307314170|ref|ZP_07593780| Hxx 0|0|W] [gi|323378200|ref|ADX50468| H...

▸ **2 samesets of gi|30059924**

		Score	Mass	Matches	Sequences	emPAI
1.3	**gi|121144335**	679	62285	18 (12)	13 (8)	0.76

H55 0|O75:H55|E2987-73

		Score	Mass	Matches	Sequences	emPAI
1.4	**gi|16901492**	337	52025	13 (9)	9 (6)	0.63

H8 0|H8|0

▸ **3 samesets of gi|16901492**

		Score	Mass	Matches	Sequences	emPAI
1.5	**gi|33590243**	325	55093	18 (10)	12 (7)	0.69

H16 NM|O157:NM(H16)|PC02

▸ **3 samesets of gi|33590243**

▾**67 peptide matches (55 non-duplicate, 12 duplicate)**

Query	Dupes	Observed	Mr(expt)	Mr(calc)	Delta	M	Score	Expect	Rank	U	1	2	3	4	5	Peptide
31		315.7008	629.3870	629.3860	0.0010	1	1	0.75	▸1	U					■	K.VDKLR.S
36	▸1	**316.6906**	**631.3666**	**631.3653**	**0.0013**	**0**	**27**	**0.02**	▸1		■	■	■	■	■	**R.LSSGLR.I**
64		331.6812	661.3478	660.3806	0.9672	0	0	0.97	▸1	U				■		K.TGAVSVK.T
100		351.2136	700.4126	700.4847	-0.0720	1	0	0.99	▸1	U			■			K.TIIKVK.D
113		355.1981	708.3816	708.3806	0.0010	0	7	1	▸1		■	■	■			R.FTSNIK.G
120		**358.7067**	**715.3988**	**715.3977**	**0.0012**	**0**	**31**	**0.0056**	▸1		■	■	■			**K.GLTQAAR.N**
135		**366.6982**	**731.3818**	**731.3813**	**0.0005**	**0**	**35**	**0.001**	▸1		■			■	■	**R.LSEIDR.V**
162		379.7426	757.4706	758.4174	-0.9467	0	3	0.52	▸2	U					■	K.LDEALAK.V
299		430.2479	858.4812	859.4399	-0.9587	0	0	0.9	▸1	U			■			K.AQDVNVSK.D
301		430.7089	859.4032	860.4240	-1.0207	0	1	0.84	▸1	U				■		K.VELGGSDGK.T
437	▸2	**473.2592**	**944.5038**	**944.5039**	**-0.0001**	**0**	**59**	**3.9e-06**	▸1		■	■				**R.SSLGAIQNR.L**
491		**486.2668**	**970.5190**	**971.5148**	**-0.9958**	**0**	**35**	**0.0003**	▸1	**U**				■		**R.SNLGAIQNR.F**
566		502.7936	1003.5726	1002.5094	1.0632	1	2	0.95	▸1			■	■			K.SRLDEIDR.V
628	▸1	**519.7459**	**1037.4772**	**1037.4778**	**-0.0005**	**0**	**47**	**2.1e-05**	▸1	**U**	■					**K.AVDNGNGTYK.V**
657		**525.7767**	**1049.5388**	**1049.5393**	**-0.0005**	**0**	**62**	**6.4e-07**	▸1	**U**	■					**K.ELFTLAGDGK.S**
680		531.8186	1061.6226	1061.4924	0.1303	0	4	0.96	▸1	U					■	K.NDGSAQAIMR.E
703	▸1	**539.2696**	**1076.5246**	**1077.4873**	**-0.9626**	**0**	**21**	**0.011**	▸1	**U**					■	**K.NDGSAQAIMR.E + Oxidation (M)**
769	▸1	**551.2674**	**1100.5202**	**1100.5210**	**-0.0008**	**0**	**68**	**1.4e-06**	▸1		■	■	■	■	■	**K.DDAAGQAIANR.F**
800		**559.8059**	**1117.5972**	**1117.5979**	**-0.0006**	**0**	**40**	**9.7e-05**	▸1	**U**	■					**K.AATISDLTAQK.A**
828		567.8111	1133.6076	1133.6080	-0.0004	1	2	1.1	▸1	U				■		K.LFKLEDANGK.D
835		379.8428	1136.5066	1137.5150	-1.0084	0	1	0.74	▸2	U					■	K.DTTDATGTAGTK.V
942		397.2037	1188.5893	1187.6034	0.9859	0	2	0.67	▸1	U			■			K.ALDDAISQIDK.F
947	▸1	**596.3018**	**1190.5890**	**1190.5891**	**-0.0000**	**0**	**72**	**3.5e-07**	▸1		■	■				**K.NQSALSSSIER.L**
948		397.8891	1190.6455	1190.5891	0.0564	0	6	1.5	▸1		■	■				K.NQSALSSSIER.L

Query	Dupes	Observed	Mr(expt)	Mr(calc)	Delta	M	Score	Expect	Rank	U	1	2	3	4	5	Peptide
965		600.8538	1199.6930	1199.6734	0.0196	1	17	0.019	▶1	U					■	K.LRSSLGAVQNR.F
1066		628.3198	1254.6250	1254.6244	0.0006	0	34	0.00043	▶1	U		■				K.FNALDAATAFSK.L
1117	▶1	641.3301	1280.6456	1280.6460	-0.0003	0	79	1.9e-08	▶1	U	■					K.LTTETTSAGTATK.D
1186	▶1	664.3292	1326.6438	1326.6456	-0.0017	0	83	4.5e-09	▶1	U	■					K.FEAVAGADAYVSK.D
1216		672.8773	1343.7400	1343.7408	-0.0008	0	56	2.5e-06	▶1	U			■			-.SLSLITQNNINK.N
1240	▶1	685.3422	1368.6698	1368.6708	-0.0009	0	84	3.8e-09	▶1	U	■					K.SGVMIGSATFTNGK.G
1261		693.3401	1384.6656	1384.6657	-0.0000	0	81	8.8e-09	▶1	U	■					K.SGVMIGSATFTNGK.G + Oxidation (M)
1321		720.9124	1439.8102	1439.8096	0.0006	0	112	2.8e-11	▶1		■	■	■			K.AQIIQQAGNSVLAK.A
1375		747.9174	1493.8202	1493.8202	0.0001	0	58	1.1e-05	▶1		■	■				K.ANQVPQQVLSLLQG.-
1381		750.4026	1498.7906	1498.7879	0.0028	0	133	5.4e-14	▶1	U	■					K.DPLAALDAAISSIDK.F
1435		521.6169	1561.8289	1561.8311	-0.0023	0	31	0.0038	▶1	U	■					K.VTTSNAALTASQALSK.L
1436		781.9230	1561.8314	1561.8311	0.0003	0	96	1e-09	▶1	U	■					K.VTTSNAALTASQALSK.L
1450	▶1	789.4178	1576.8210	1576.8209	0.0001	0	94	4.5e-10	▶1	U	■					R.VSGQTQFNGVNVLSK.D
1480		538.9440	1613.8102	1613.8121	-0.0019	1	45	0.0003	▶1		■	■	■	■	■	R.INSAKDDAAGQAIANR.F
1481		807.9126	1613.8106	1613.8121	-0.0015	1	86	2.1e-08	▶1		■	■	■	■	■	R.INSAKDDAAGQAIANR.F
1491		543.2695	1626.7867	1626.7889	-0.0023	1	19	0.013	▶1	U	■					K.FEAVAGADAYVSKDGK.L
1517		836.3802	1670.7458	1670.7457	0.0001	0	118	9.5e-12	▶1		■	■	■			R.IQDADYATEVSNMSK.A
1533		844.3768	1686.7390	1686.7407	-0.0016	0	103	3.9e-10	▶1		■	■	■			R.IQDADYATEVSNMSK.A + Oxidation (M)
1561		860.3570	1718.6994	1718.7974	-0.0979	0	8	0.15	▶1	U					■	K.ALAYNDAPMSVYFGGK.N + Oxidation (M)
1563		861.4225	1720.8304	1720.8302	0.0002	0	86	2.7e-09	▶1	U	■					K.GTADGMTSGTTPVVATGAK.A
1579		869.4201	1736.8256	1736.8251	0.0005	0	95	3.1e-10	▶1	U	■					K.GTADGMTSGTTPVVATGAK.A + Oxidation (M)
1605		588.9194	1763.7364	1763.7387	-0.0023	0	28	0.0017	▶1	U	■					K.YDADAGNFSYNNTANK.T
1606		882.8759	1763.7372	1763.7387	-0.0014	0	161	8.6e-17	▶1	U	■					K.YDADAGNFSYNNTANK.T
1702		620.0022	1856.9848	1856.9843	0.0004	0	37	0.00031	▶1	U	■					K.TSAAAGTLADTLLPAAGQTK.T
1703		929.5002	1856.9858	1856.9843	0.0015	0	123	6.7e-13	▶1	U	■					K.TSAAAGTLADTLLPAAGQTK.T
1791		663.1566	1986.4480	1986.0633	0.3847	1	0	0.98	▶1	U					■	K.GAELSASDLKALATTNPLSK.L
1850		695.7147	2084.1223	2084.1225	-0.0003	0	68	1.1e-06	▶1		■	■		■	■	M.AQVINTNSLSLiTQNNiNK.N
1851		1043.0690	2084.1234	2084.1225	0.0009	0	105	2.2e-10	▶1		■	■		■	■	M.AQVINTNSLSLiTQNNiNK.N
1910		750.3715	2248.0927	2248.0931	-0.0004	0	88	8.5e-09	▶1		■	■	■			R.LDSAVTNLNNTTTNLSEAQSR.I
1911		1125.0540	2248.0934	2248.0931	0.0003	0	137	1.1e-13	▶1		■	■	■			R.LDSAVTNLNNTTTNLSEAQSR.I
1938	▶1	779.0688	2334.1846	2334.1815	0.0030	1	55	2.9e-06	▶1	U	■					K.KIDSDTLGLNGFNVNGSGTIANK.A

▶45 subsets and intersections (158 subset proteins in total)

10 per page 1

Not what you expected? Try the select summary.

Mascot: http://www.matrixscience.com/
