# Supplementary material for: MS-H: A Novel Proteomic Approach to Isolate and Type the E. coli H Antigen Using Membrane Filtration and Liquid Chromatography-Tandem Mass Spectrometry (LC-MS/MS)
Source: PLoS One. 2013 Feb 21;8(2):e57339. doi: 10.1371/journal.pone.0057339 (PMC3578835; doi:10.1371/journal.pone.0057339)
Supplement: Representative Peptide Data S1 — Peptide data are represented as the Mascot search results from all 53 serotypes, obtained under the Orbitrap platform in Table 4 with related E. coli reference strains. “U” denotes a unique peptide specific for each of the proteins 1.1, 1.2, and beyond. The number 1.1 (shown as 1 in the peptide list and phylogenetic tree) represents the protein which obtained the highest score and confidence value after a Mascot search. This protein, known as the first hit, was used to designate the MS-H type of the unknown flagellin. Related peptides 1.2 (2), 1.3 (3), etc. represented the second, third, etc. hits for MS-H typing analysis. (DOCX) [file pone.0057339.s009.docx › H52-E373.pdf]

MASCOT Search Results

User :
E-mail :
Search title : Submitted from 20110901-0623 by Mascot Daemon on VARIABLE
MS data file : C:\Documents and Settings\keding\Desktop\Raw data\20110901-002-0031-00623\20110901-007-EC373-MS2-RP-r.RAW
Database : Flagellin_v2 (192 sequences; 89,845 residues)
Taxonomy : Bacteria (Eubacteria) (192 sequences)
Timestamp : 4 Sep 2011 at 17:45:11 GMT

Not what you expected? Try **the select summary**.

Search parameters

Score distribution

Legend

Protein Family Summary

Significance threshold p< 0.05 Max. number of families AUTO
Ions score or expect cut-off 0 Dendrograms cut at 0

Protein family 1 (out of 1)

10 per page 1

1

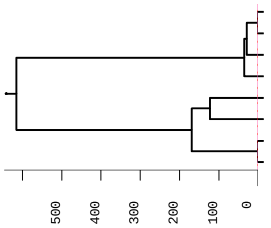

1	**gi|30059928**	1601	H52 0|H52|C2187-69
8	**gi|46093545**	230	H3 0|0|Bi7327-41
7	**gi|154710444**	258	H35 0|H35|0
5	**gi|323174561**	647	Hxx(H25 99.7%) 0|0|LT-68
2	**gi|30059924**	1381	H49 0|H49|2147-59 [gi|315061223|ref|ADT75550| Hxx 0|0|W] [gi|323186300|ref|EFZ71650| H...
4	**gi|6009851**	734	H51 0|H51|C218-70 [gi|30059926|ref|AAP13335| H51 0|H51|C218-70]
3	**gi|120312**	746	Hxx(H48 100.0%) 0|0|0 [gi|6009847|ref|BAA85088| H48 0|H48|P4] [gi|30059922|ref|AAP13333...
6	**gi|291432882**	515	Hxx(H10 99.8%) 0|0|B185 [gi|293415234|ref|ZP_06657877| Hxx 0|0|B185]

Threshold (**0**): 0

		Score	Mass	Matches	Sequences	emPAI
**1.1**	**gi|30059928**<br>H52 0|H52|C2187-69	1601	46003	41 (32)	29 (27)	8.20
**1.2**	**gi|30059924**<br>H49 0|H49|2147-59 [gi|315061223|ref|ADT75550| Hxx 0|0|W] [gi|323186300|ref|EFZ71650| Hxx 0|0|1357] [gi|307314170|ref|ZP_07593780| Hxx 0|0|W] [gi|323378200|ref|ADX50468| Hxx 0|0|KO...	1381	57964	37 (29)	28 (24)	3.96
**1.3**	**gi|120312**<br>Hxx(H48 100.0%) 0|0|0 [gi|6009847|ref|BAA85088| H48 0|H48|P4] [gi|30059922|ref|AAP13333| H48 0|H48|P4] [gi|1788232|ref|AAC74990| H48 0|0|K-12] [gi|89108758|ref|AP_002538| Hxx 0|0|K-...<br>**2 samesets of gi|120312**	746	51265	28 (18)	19 (15)	2.07
**1.4**	**gi|6009851**<br>H51 0|H51|C218-70 [gi|30059926|ref|AAP13335| H51 0|H51|C218-70]	734	61407	20 (17)	17 (14)	1.43
**1.5**	**gi|323174561**<br>Hxx(H25 99.7%) 0|0|LT-68<br>**1 sameset of gi|323174561**	647	46392	27 (14)	19 (12)	1.62
**1.6**	**gi|291432882**<br>Hxx(H10 99.8%) 0|0|B185 [gi|293415234|ref|ZP_06657877| Hxx 0|0|B185]<br>**5 samesets of gi|291432882**	515	44250	18 (14)	13 (12)	1.74
**1.7**	**gi|154710444**<br>H35 0|H35|0	258	52714	14 (8)	11 (7)	0.62
**1.8**	**gi|46093545**<br>H3 0|0|Bi7327-41	230	55534	16 (7)	13 (6)	0.50

82 peptide matches (74 non-duplicate, 8 duplicate)

Query	Dupes	Observed	Mr(expt)	Mr(calc)	Delta	M	Score	Expect	Rank	U	1	2	3	4	5	6	7	8	Peptide
6		302.1713	602.3280	601.3911	0.9369	1	7	0.3	1	U								■	K.TVVRK.D
23		**316.6901**	**631.3656**	**631.3653**	**0.0003**	**0**	**29**	**0.013**	1		■	■	■	■	■	■	■	■	**R.LSSGLR.I**
68		337.2161	672.4176	673.3759	-0.9582	0	8	0.16	1	U					■				K.NGATALK.L
104		352.2031	702.3916	702.3912	0.0005	0	3	0.74	1	U					■				K.AIASVDK.F
111		355.1979	708.3812	708.3806	0.0006	0	17	0.14	1		■	■	■	■	■	■	■	■	R.FTSNIK.G
114		**358.7066**	**715.3986**	**715.3977**	**0.0010**	**0**	**35**	**0.0021**	1		■	■	■	■	■	■			**K.GLTQAAR.N**
126		366.2189	730.4232	730.4225	0.0008	0	7	0.64	1	U								■	K.LDTALAK.V
156	1	**380.4469**	**758.8792**	**759.3763**	**-0.4970**	**0**	**23**	**0.027**	1		■	■	■		■				**R.LDEIDR.V**
369		**455.2187**	**908.4228**	**908.4239**	**-0.0011**	**0**	**42**	**7e-05**	1	**U**	■								**K.DYLAGADGK.D**
398	2	**466.2509**	**930.4872**	**930.4883**	**-0.0010**	**0**	**58**	**7.9e-06**	1		■		■		■	■	■	■	**R.SSLGAVQNR**
402		**467.2655**	**932.5164**	**932.5179**	**-0.0014**	**0**	**34**	**0.00039**	1	**U**	■								**K.IDSSTLGLK.G**
430		**473.2532**	**944.4918**	**944.5039**	**-0.0121**	**0**	**18**	**0.046**	1			■		■					**R.SSLGAIQNR.L**
477		484.3056	966.5966	965.5182	1.0785	1	5	0.34	1	U							■		K.KDGSGYVIK.G
497		488.7757	975.5368	974.5145	1.0224	1	7	0.18	1	U				■					K.SRLSEIDR.V
503		**490.2630**	**978.5114**	**978.5134**	**-0.0020**	**0**	**45**	**3.6e-05**	1	**U**	■								**K.GFSVSGNALK.V**
549	2	502.2612	1002.5078	1002.5094	-0.0016	1	17	0.12	1		■	■	■		■				K.SRLDEIDR.V
591		**511.8809**	**1021.7472**	**1020.5604**	**1.1869**	**0**	**13**	**0.048**	1	**U**						■			**K.ANQSLVVYK.D**
610		344.5262	1030.5568	1031.5512	-0.9945	0	0	0.89	1	U								■	K.AVQIANFGGR.V

Query	Dupes	Observed	Mr(expt)	Mr(calc)	Delta	M	Score	Expect	Rank	U	1	2	3	4	5	6	7	8	Peptide
708		551.2672	1100.5198	1100.5210	-0.0012	0	65	2.9e-06	▶1		■	■	■	■	■	■	■	■	K.DDAAGQAIANR.F
721		554.2739	1106.5332	1106.5356	-0.0024	0	13	0.047	▶1	U								■	K.AGAFAAQTADGK.S
776		570.2815	1138.5484	1137.5666	0.9818	0	0	0.92	▶1	U					■				K.ATADYVVQSGK.D
864		596.3009	1190.5872	1190.5891	-0.0018	0	55	1.7e-05	▶1		■	■	■	■		■			K.NQSALSSSIER.L
870		598.8000	1195.5854	1194.5517	1.0338	0	4	0.37	▶1	U		■							K.DAAQSSIDFGGK.K
879		600.8522	1199.6898	1199.6734	0.0164	1	0	0.91	▶1	U								■	K.LRSSLGAVQNR.F
909		406.2035	1215.5887	1216.6663	-1.0776	1	22	0.0068	▶1	U							■		K.EINSKTLGLDK.L
935		619.7941	1237.5736	1237.5761	-0.0024	0	109	1.2e-11	▶1	U	■								K.FGANDTAAAAMAK.T
954	▶1	627.7924	1253.5702	1253.5710	-0.0008	0	87	1.8e-09	▶1	U	■								K.FGANDTAAAAMAK.T + Oxidation (M)
958	▶1	628.3187	1254.6228	1254.6244	-0.0016	0	61	8e-07	▶1	U		■							K.FNALDAATAFSK.L
1052		441.8926	1322.6560	1322.6466	0.0093	1	11	0.076	▶1	U		■							K.DAAQSSIDFGGKK.Y
1121		459.8791	1376.6155	1375.7347	0.8808	1	7	0.19	▶1	U							■		K.EVLFEAKVAADGK.V
1188		720.9108	1439.8070	1439.8096	-0.0026	0	67	9.7e-07	▶1			■	■	■		■			K.AQIIQQAGNSVLAK.A
1202		728.9066	1455.7986	1455.8045	-0.0059	0	115	4.7e-12	▶1		■				■				K.AQIIQQAGNSVLSK.A
1233		747.9177	1493.8208	1493.8202	0.0007	0	40	0.00066	▶1		■	■	■	■	■	■			K.ANQVPQQVLSLLQG.-
1259		766.3949	1530.7752	1530.7777	-0.0025	0	58	1.7e-06	▶1	U	■								K.IQLTDELDVDGSVK.T
1278		777.8801	1553.7456	1553.7474	-0.0018	0	92	5.7e-10	▶1	U		■							K.YAANVGAQAYVGADGK.L
1279		781.4199	1560.8252	1560.8260	-0.0008	0	75	1.4e-07	▶1		■	■	■						R.VSGQTQFNGVNVLAK
1318		538.9436	1613.8090	1613.8121	-0.0031	1	27	0.018	▶1		■	■	■	■	■	■	■	■	R.INSAKDDAAGQAIANR.F
1322		810.8522	1619.6898	1619.6911	-0.0012	0	106	2.7e-11	▶1	U	■								K.DAIENGGDAATNEDTK.I
1334		547.2904	1638.8494	1638.7771	0.0723	0	1	0.8	▶1	U								■	K.SEMSPTLTSVNATTGK.N + Oxidation (M)
1344	▶1	827.4221	1652.8296	1651.8781	0.9516	1	1	0.84	▶1	U					■				K.KIDSSTLGLNGFSVSK.N
1349		829.8922	1657.7698	1657.7696	0.0002	0	38	0.00014	▶1	U		■							K.AAAGQSQSGTYTFANGK.V
1356		836.3781	1670.7416	1670.7457	-0.0041	0	114	2.5e-11	▶1		■	■	■	■	■	■			R.IQDADYATEVSNMSK.A
1365		843.4569	1684.8992	1684.8996	-0.0003	0	122	2.7e-12	▶1	U		■							K.IQVGANDGQTITIDLK.K
1365		843.4569	1684.8992	1685.8836	-0.9843	0	38	0.00066	▶2	U							■		K.IQVGANDGETITIDLK.E
1366		844.3768	1686.7390	1686.7407	-0.0016	0	90	8e-09	▶1		■	■	■	■	■	■			R.IQDADYATEVSNMSK.A + Oxidation (M)
1373		565.9316	1694.7730	1694.7748	-0.0018	1	21	0.0078	▶1	U		■							K.AADDKDAAQSSIDFGGK.K
1397		868.4326	1734.8506	1734.8425	0.0082	0	113	4.9e-12	▶1	U		■							K.VNFDVDASGNITIGGEK.A
1402		868.9619	1735.9092	1735.9105	-0.0012	0	75	3.1e-08	▶1	U	■								K.VTTNSAGAAVGYVTIQGK.D
1445		900.4777	1798.9408	1798.9789	-0.0380	1	96	4.6e-10	▶1		■			■	■				K.IQVGANDGQTISIDLkK.I
1525		992.0229	1982.0312	1982.0321	-0.0008	0	68	3e-07	▶1	U	■								K.VSDAITTVPGANAGDAPVTVK.F
1533		670.6525	2008.9357	2008.9953	-0.0596	0	2	0.68	▶1	U							■		R.NTFSTSDLAATATTELAPAK.T
1544		693.6910	2078.0512	2078.0532	-0.0020	0	14	0.17	▶1	U	■								K.TAATATFSGTATNDPLALLDK.A
1545		1040.0340	2078.0534	2078.0532	0.0003	0	110	4.1e-11	▶1	U	■								K.TAATATFSGTATNDPLALLDK.A
1547		695.7141	2084.1205	2084.1225	-0.0021	0	74	2.7e-07	▶1		■	■	■	■	■	■	■	■	M.AQVINTNSLSLiTQNNiNK.N
1548		1043.0680	2084.1214	2084.1225	-0.0011	0	85	2.2e-08	▶1		■	■	■	■	■	■	■	■	M.AQVINTNSLSLiTQNNiNK.N
1552		700.6894	2099.0464	2099.0495	-0.0031	0	40	0.00011	▶1	U	■								K.TLGISDTSGLSLHNVQSADGK.A
1561		1070.0130	2138.0114	2138.0128	-0.0013	0	90	9.6e-10	▶1	U			■						K.DTNGNLYAADVNETTGAVSVK.T
1585		750.3710	2248.0912	2248.0931	-0.0019	0	34	0.0025	▶1		■	■	■	■					R.LDSAVTNLNNTTTNLSEAQSR.I
1586		1125.0530	2248.0914	2248.0931	-0.0017	0	101	4.9e-10	▶1		■	■	■	■					R.LDSAVTNLNNTTTNLSEAQSR.I
1589		760.4007	2278.1803	2278.1805	-0.0002	0	54	4.7e-06	▶1	U				■					K.AAANVTDGAGVIAAAGVTYTATVSK.D
1592		767.0620	2298.1642	2297.2227	0.9415	1	4	0.39	▶2	U			■						K.IQVGANDNQTITIDLKQIDAK.T
1599		1210.1360	2418.2574	2418.2602	-0.0027	0	133	4.9e-14	▶1	U		■							K.AATISDLAATGANVTNSSNIVVTTK.F
1600		807.0933	2418.2581	2418.2602	-0.0021	0	88	1.9e-09	▶1	U		■							K.AATISDLAATGANVTNSSNIVVTTK.F
1603		837.7086	2510.1040	2510.1045	-0.0005	1	80	9.6e-09	▶1	U	■								K.DYLAGADGKDAIENGGDAATNEDTK.I
1604		856.0711	2565.1915	2565.1930	-0.0015	0	41	0.00025	▶1		■	■							R.ELTVQASTGTNSDSDLDSIQDEIK.S
1604		856.0711	2565.1915	2565.2293	-0.0379	0	23	0.015	▶3				■			■			R.ELTVQATTGTNSESDLSSIQDEIK.S
1605		1283.6040	2565.1934	2565.1930	0.0005	0	76	7.5e-08	▶1		■	■							R.ELTVQASTGTNSDSDLDSIQDEIK.S
1605		1283.6040	2565.1934	2565.2293	-0.0359	0	4	1.2	▶3				■			■			R.ELTVQATTGTNSESDLSSIQDEIK.S
1614		966.8277	2897.4613	2897.4591	0.0022	1	53	2.6e-05	▶1		■	■							R.NANDGISVAQTTEGALSEINNNLQRIR.E
1614		966.8277	2897.4613	2897.4591	0.0022	1	15	0.18	▶2					■	■			■	R.NANDGISLAQTTEGALSEINNNLQRVR.E
1616		1037.5460	3109.6162	3108.4558	1.1604	0	0	5.3	▶1	U			■						K.YYAVTVANDGTVTMATGATANATVTDANTTK.A + Oxidati
1617		1048.1610	3141.4612	3141.4626	-0.0014	0	49	1.2e-05	▶1	U		■							K.YTYNASTNDFTTENTVATGTATTDLGATLK.A
1618		1059.2040	3174.5902	3174.5865	0.0037	1	62	1.6e-06	▶1			■		■					R.SSLGAIQNRLDSAVTNLNNTTTNLSEAQSR.I
1618		1059.2040	3174.5902	3174.5865	0.0037	1	51	1.8e-05	▶2	U					■				R.SSLGAVQNRLDSAITNLNNTTTNLSEAQSR.I

▶57 subsets and intersections (155 subset proteins in total)

10 per page 1

Not what you expected? Try **the select summary**.

Mascot: http://www.matrixscience.com/
