# Supplementary material for: MS-H: A Novel Proteomic Approach to Isolate and Type the E. coli H Antigen Using Membrane Filtration and Liquid Chromatography-Tandem Mass Spectrometry (LC-MS/MS)
Source: PLoS One. 2013 Feb 21;8(2):e57339. doi: 10.1371/journal.pone.0057339 (PMC3578835; doi:10.1371/journal.pone.0057339)
Supplement: Representative Peptide Data S1 — Peptide data are represented as the Mascot search results from all 53 serotypes, obtained under the Orbitrap platform in Table 4 with related E. coli reference strains. “U” denotes a unique peptide specific for each of the proteins 1.1, 1.2, and beyond. The number 1.1 (shown as 1 in the peptide list and phylogenetic tree) represents the protein which obtained the highest score and confidence value after a Mascot search. This protein, known as the first hit, was used to designate the MS-H type of the unknown flagellin. Related peptides 1.2 (2), 1.3 (3), etc. represented the second, third, etc. hits for MS-H typing analysis. (DOCX) [file pone.0057339.s009.docx › H53-E374.pdf]

MASCOT Search Results

User :
E-mail :
Search title : Submitted from 20110901-0623 by Mascot Daemon on VARIABLE
MS data file : C:\Documents and Settings\keding\Desktop\Raw data\20110901-002-0031-00623\20110901-008-EC374-MS1-RP-r.RAW
Database : Flagellin_v2 (192 sequences; 89,845 residues)
Taxonomy : Bacteria (Eubacteria) (192 sequences)
Timestamp : 4 Sep 2011 at 17:49:42 GMT

Not what you expected? Try **the select summary**.

▶ **Search parameters**

▶ **Score distribution**

▶ **Legend**

Protein Family Summary

Significance threshold p< 0.05 Max. number of families AUTO
Ions score or expect cut-off 0 Dendrograms cut at 0

Protein families 1–3 (out of 3)

10 per page 1

▼**1**

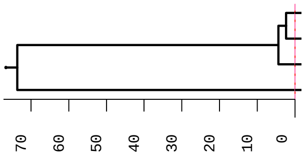

1	gi|46093552	1356	H53 0|0|E480-68
3	gi|16901498	214	H34 0|H34|0
4	gi|30059910	173	H39 0|H39|E3a
2	gi|16901500	285	H49 0|H49|0

Threshold (**0**): 0

		Score	Mass	Matches	Sequences	emPAI
1.1	**gi|46093552** H53 0|0|E480-68	1356	44861	32 (26)	26 (22)	4.50
1.2	**gi|16901500** H49 0|H49|0 ▶ 2 samesets of gi|16901500	285	57940	12 (5)	9 (5)	0.32
1.3	**gi|16901498** H34 0|H34|0 ▶ 3 samesets of gi|16901498	214	56006	10 (5)	8 (5)	0.33
1.4	**gi|30059910** H39 0|H39|E3a	173	44239	8 (4)	8 (4)	0.33

▼**44 peptide matches (39 non-duplicate, 5 duplicate)**

Query	Dupes	Observed	Mr(expt)	Mr(calc)	Delta	M	Score	Expect	Rank	U	1	2	3	4	Peptide
26		316.6895	631.3644	631.3653	-0.0009	0	34	0.0036	1		■	■	■	■	R.LSSGLR.I
99		355.1976	708.3806	708.3806	0.0000	0	7	1.3	1		■	■	■	■	R.FTSNIK.G
103		358.7004	715.3862	715.3864	-0.0002	0	44	0.00027	1	U	■				R.LAEIDR.V
104		358.7130	715.4114	715.4116	-0.0001	0	33	0.004	1	U	■				K.IDIDLK.K
112		364.7131	727.4116	727.3613	0.0504	0	1	0.78	2	U			■		K.NQAGNPK.K
119		366.7035	731.3924	731.3926	-0.0001	0	34	0.0015	1	U	■				K.GLTQASR.N
195		405.7036	809.3926	809.3919	0.0007	0	36	0.00026	1	U	■				K.DGTGYAVK.D
265		424.2472	846.4798	846.4811	-0.0013	0	37	0.00025	1	U	■				K.TGVVSVGTK.I
366		461.7475	921.4804	920.4967	0.9837	0	0	0.9	2	U	■				K.AADGLYALK.D
376		466.2546	930.4946	930.4559	0.0387	0	3	2.1	1	U	■				K.DGAYHAAVK.N
378		466.7426	931.4706	930.4883	0.9824	0	3	2.2	1	U				■	R.SSLGAVQNR.L
393	1	473.2525	944.4904	944.5039	-0.0135	0	60	2.9e-06	1		■	■	■		R.SSLGAIQNR
498		508.7887	1015.5628	1016.5502	-0.9874	0	17	0.018	1	U				■	K.NVDLSAVATK.L
629		551.2669	1100.5192	1100.5210	-0.0018	0	74	3.4e-07	1		■	■	■	■	K.DDAAGQAIANR.F
720		582.7950	1163.5754	1163.5782	-0.0028	0	58	4.3e-06	1	U	■				K.SQSSLSSAIER.L
721	1	582.8031	1163.5916	1163.5935	-0.0019	0	56	7.3e-06	1	U	■				R.VSGQTQFNGVK.V
738		587.7950	1173.5754	1173.5778	-0.0024	1	61	7.7e-07	1	U	■				K.DKDGAYHAAVK.N
739		392.1995	1173.5767	1173.5778	-0.0011	1	35	0.00034	1	U	■				K.DKDGAYHAAVK.N
766		598.8005	1195.5864	1194.5517	1.0348	0	6	0.27	1	U		■			K.DAAQSSIDFGGK.K
815		615.8181	1229.6216	1229.6252	-0.0035	0	68	1.6e-07	1	U	■				K.LSIQVGANDGEK.I
841		627.8034	1253.5922	1254.6244	-1.0322	0	3	0.56	1	U		■			K.FNALDAATAFSK.L

Query	Dupes	Observed	Mr(expt)	Mr(calc)	Delta	M	Score	Expect	Rank	U	1	2	3	4	Peptide
850		420.8853	1259.6341	1259.6721	-0.0381	0	11	0.084	▶1	U	■				K.TVANGGDIVLSSK.T
851	▶1	630.8413	1259.6680	1259.6721	-0.0041	0	95	3.1e-10	▶1	U	■				K.TVANGGDIVLSSK.T
954		671.4111	1340.8076	1340.7300	0.0777	0	0	0.93	▶1	U				■	K.GTATQNPLALLDK.A
1006		705.8463	1409.6780	1409.6787	-0.0006	0	68	1.7e-07	▶1	U	■				K.AEIQIDSHSADPK.A
1007		470.9000	1409.6782	1409.6787	-0.0005	0	36	0.00024	▶1	U	■				K.AEIQIDSHSADPK.A
1095		746.8889	1491.7632	1491.7681	-0.0049	0	129	1.8e-13	▶1	U	■				K.VAANTSGLAANQTFK.S
1207		823.9073	1645.8000	1644.9046	0.8954	1	15	0.031	▶1	U			■		K.TTANTAAKGSDILAALK.T
1216		829.4545	1656.8944	1656.8934	0.0010	0	105	3e-11	▶1	U	■				K.ISTSPDVLATIDNALK.I
1285	▶1	868.4308	1734.8470	1734.8425	0.0046	0	87	2.2e-09	▶1	U		■			K.VNFDVDASGNITIGGEK.A
1303		584.9459	1751.8159	1751.9053	-0.0895	1	1	0.76	▶1	U	■				K.TIKAEIQIDSHSADPK.A
1325		589.3145	1764.9217	1764.9258	-0.0041	1	24	0.0043	▶1	U	■				K.KIDTGTLGLANFSVDSK.F
1359	▶1	902.9415	1803.8684	1803.9438	-0.0754	1	5	1.6	▶1			■	■		K.NQSALSSSIERLSSGLR.I
1454		985.4476	1968.8806	1968.8813	-0.0007	0	116	2.8e-12	▶1	U	■				K.DNGELGYVVENADGTFNR.A
1469		997.4996	1992.9846	1992.9865	-0.0018	0	171	1.9e-17	▶1	U	■				R.FDSAITNLGNTVNNLSSAR.S
1504		1043.0720	2084.1294	2084.1225	0.0069	0	106	1.8e-10	▶1		■	■	■	■	M.AQVINTNSLSLiTQNNiNK.N
1564		565.7657	2259.0337	2259.1165	-0.0828	0	0	0.91	▶2	U				■	K.NGSMAIQVGANDGQTISIDLQK.I
1640		1335.6580	2669.3014	2669.3004	0.0010	0	115	3e-12	▶1	U	■				R.NANDGISIAQTTEGALNEINNNLQR.V
1656		968.4586	2902.3540	2902.3540	-0.0001	0	83	5e-09	▶1	U	■				R.ELAVQSQNGTNSDSDVQSIQEEIQQR.L

▶**20 subsets and intersections (163 subset proteins in total)**

▶**2**	**gi|112820172**	1264	H21 0|EHEC serogroup: O113:H21|0
▶**3**	**gi|307553085**	224	Hxx(H54 27.9%) 0|0|ABU 83972

10 per page 1

Not what you expected? Try **the select summary**.

Mascot: http://www.matrixscience.com/
