# Supplementary material for: MS-H: A Novel Proteomic Approach to Isolate and Type the E. coli H Antigen Using Membrane Filtration and Liquid Chromatography-Tandem Mass Spectrometry (LC-MS/MS)
Source: PLoS One. 2013 Feb 21;8(2):e57339. doi: 10.1371/journal.pone.0057339 (PMC3578835; doi:10.1371/journal.pone.0057339)
Supplement: Representative Peptide Data S1 — Peptide data are represented as the Mascot search results from all 53 serotypes, obtained under the Orbitrap platform in Table 4 with related E. coli reference strains. “U” denotes a unique peptide specific for each of the proteins 1.1, 1.2, and beyond. The number 1.1 (shown as 1 in the peptide list and phylogenetic tree) represents the protein which obtained the highest score and confidence value after a Mascot search. This protein, known as the first hit, was used to designate the MS-H type of the unknown flagellin. Related peptides 1.2 (2), 1.3 (3), etc. represented the second, third, etc. hits for MS-H typing analysis. (DOCX) [file pone.0057339.s009.docx › H54-E377.pdf]

MASCOT Search Results

User :
E-mail :
Search title : Submitted from 20110901-0623 by Mascot Daemon on VARIABLE
MS data file : C:\Documents and Settings\keding\Desktop\Raw data\20110901-002-0031-00623\20110901-009-EC377-MS1-RP-r.RAW
Database : Flagellin_v2 (192 sequences; 89,845 residues)
Taxonomy : Bacteria (Eubacteria) (192 sequences)
Timestamp : 4 Sep 2011 at 17:52:57 GMT

Not what you expected? Try **the select summary**.

Search parameters
Score distribution
Legend

Protein Family Summary

Significance threshold p< 0.05 Max. number of families AUTO
Ions score or expect cut-off 0 Dendrograms cut at 0

Protein family 1 (out of 1)

10 per page 1

1

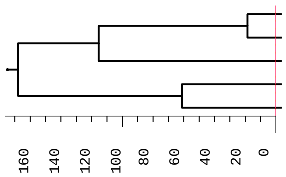

**1 gi|46093563**	2839	H54 0|0|E223-69
**2 gi|33590243**	957	H16 NM|O157:NM(H16)|PC02
**3 gi|121144335**	505	H55 0|O75:H55|E2987-73
**5 gi|33590227**	345	H42 NM|O157:NM(H42)|25
**4 gi|33590260**	388	H29 0|O157:H29|Y63

Threshold (**0**): 0

		Score	Mass	Matches	Sequences	emPAI
**1.1**	**gi|46093563**<br>H54 0|0|E223-69	2839	54419	81 (64)	43 (41)	31.07
**1.2**	**gi|33590243**<br>H16 NM|O157:NM(H16)|PC02<br>**2 samesets of gi|33590243**	957	55093	30 (20)	17 (12)	1.84
**1.3**	**gi|121144335**<br>H55 0|O75:H55|E2987-73	505	62285	15 (11)	8 (7)	0.59
**1.4**	**gi|33590260**<br>H29 0|O157:H29|Y63	388	45720	18 (10)	9 (7)	0.87
**1.5**	**gi|33590227**<br>H42 NM|O157:NM(H42)|25	345	44094	15 (11)	9 (8)	1.06

102 peptide matches (81 non-duplicate, 21 duplicate)

Query	Dupes	Observed	Mr(expt)	Mr(calc)	Delta	M	Score	Expect	Rank	U	1	2	3	4	5	Peptide
3		301.1872	600.3598	600.3595	0.0004	0	28	0.0014	1	U	■					K.LNVQK.A
10		304.6737	607.3328	607.2636	0.0693	0	6	0.23	1	U				■		K.DNTMK.I
33	2	316.6898	631.3650	631.3653	-0.0003	0	36	0.0022	1		■	■	■	■	■	R.LSSGLR.I
110	1	347.2002	692.3858	692.3857	0.0001	0	24	0.0097	1		■	■				R.FTANIK.G
160		366.7037	731.3928	731.3926	0.0003	0	21	0.025	1		■	■				K.GLTQASR.N
170		374.6665	747.3184	747.3188	-0.0003	0	6	0.24	1	U	■					K.DHDFSK.Q
179		380.2033	758.3920	758.4174	-0.0253	0	33	0.0029	1	U		■				K.LDEALAK.V
179		380.2033	758.3920	758.3922	-0.0002	0	1	5.1	3	U	■					R.LNEIDR.V
203		391.6870	781.3594	781.3606	-0.0012	0	16	0.027	1	U	■					K.FNETSGK.Y
283	2	418.7289	835.4432	835.4440	-0.0007	0	57	2.1e-06	1	U	■					K.VEGGYALK.V
334		429.2208	856.4270	856.4291	-0.0020	0	27	0.0021	1	U	■					K.DVDGVPQK.G
419		455.2550	908.4954	908.4967	-0.0013	1	36	0.00026	1	U	■					K.TYAADKLK.D
420		303.8398	908.4976	908.4967	0.0009	1	17	0.022	1	U	■					K.TYAADKLK.D
437		460.2396	918.4646	918.4658	-0.0012	0	78	1.6e-08	1	U	■					K.SLEDVGATK.N
455		466.7422	931.4698	930.4883	0.9816	0	3	2.2	1			■	■	■		R.SSLGAVQNR
503		480.2478	958.4810	958.4832	-0.0022	0	62	1.2e-06	1	U	■					R.SDLGAVQNR.F
503		480.2478	958.4810	958.5196	-0.0385	0	26	0.0042	2	U					■	R.SSLGVVQNR.L
515		482.7752	963.5358	963.5389	-0.0031	1	47	2.2e-05	1	U	■					K.KVEGGYALK.V
517		322.1869	963.5389	963.5389	-0.0000	1	42	7e-05	1	U	■					K.KVEGGYALK.V
529		486.7753	971.5360	971.5400	-0.0039	0	62	7.7e-07	1	U	■					K.ALAQVDSLR.S
538	2	489.2601	976.5056	976.5077	-0.0020	0	40	9.4e-05	1	U	■					K.TETVTIGEK.T
560		495.2569	988.4992	988.4978	0.0015	1	33	0.00053	1	U	■					K.LKDHDFSK.Q
561		330.5078	988.5016	988.4978	0.0038	1	43	4.8e-05	1	U	■					K.LKDHDFSK.Q
561		330.5078	988.5016	989.5029	-1.0013	0	4	0.4	2	U		■				K.GAELSASDLK.A

Query	Dupes	Observed	Mr(expt)	Mr(calc)	Delta	M	Score	Expect	Rank	U	1	2	3	4	5	Peptide
616	4	508.7200	1015.4254	1015.4281	-0.0026	0	39	0.00012	1	U	■					K.MTYTDSNGK.K
627		340.8618	1019.5636	1019.5400	0.0236	0	2	1	1	U					■	K.AIAQVDTFR.S
687		533.2627	1064.5108	1064.5138	-0.0030	0	45	3.1e-05	1	U	■					K.AYTPSGENVK.V
701		539.2690	1076.5234	1077.4873	-0.9638	0	19	0.016	1	U		■				K.NDGSAQAIMR.E + Oxidation (M)
702		360.1894	1077.5464	1077.4873	0.0591	0	5	0.35	1	U		■				K.NDGSAQAIMR.E + Oxidation (M)
747	2	551.2666	1100.5186	1100.5210	-0.0024	0	64	3.4e-06	1		■	■	■	■	■	K.DDAAGQAIANR.F
806		572.7678	1143.5210	1143.5230	-0.0020	1	32	0.005	1	U	■					K.MTYTDSNGKK.V
807	1	382.1815	1143.5227	1143.5230	-0.0003	1	28	0.013	1	U	■					K.MTYTDSNGKK.V
827	1	577.2710	1152.5274	1152.5299	-0.0024	0	57	1.8e-06	1	U	■					K.NGDYEITVDK.D
841		581.2982	1160.5818	1159.5179	1.0639	1	4	0.5	1	U	■					K.MTYTDSNGKK.V + Oxidation (M)
847		582.7954	1163.5762	1163.5782	-0.0020	0	59	4.2e-06	1		■	■				K.SQSSLSSAIER.L
848	1	582.8027	1163.5908	1163.5935	-0.0027	0	65	1e-06	1	U	■					R.VSGQTQFNGVK.V
862	2	588.2849	1174.5552	1174.5578	-0.0026	0	103	5e-11	1	U	■					K.TGGVDNTAAGNAK.L
895		600.8530	1199.6914	1199.6734	0.0180	1	9	0.14	1	U		■				K.LRSSLGAVQNR.F
920		406.5210	1216.5412	1217.5888	-1.0476	0	12	0.068	1	U				■		R.VTIDGDGTNQAK.I
921	2	609.2780	1216.5414	1217.5888	-1.0474	0	14	0.04	1	U				■		R.VTIDGDGTNQAK.I
923		609.3207	1216.6268	1216.6299	-0.0031	0	29	0.0013	1	U	■					K.EINSQTLGLDK.L
1013		644.3294	1286.6442	1286.6466	-0.0024	0	102	6.7e-11	1	U	■					K.DGSAALVAGQSSPK.S
1084		672.8769	1343.7392	1343.7408	-0.0016	0	94	4.2e-10	1	U			■			-.SLSLITQNNINK.N
1165		475.5923	1423.7551	1423.7671	-0.0120	1	1	0.92	1	U		■				K.VYTANITNKTATK.G
1186		719.3633	1436.7120	1436.7147	-0.0027	1	43	6.5e-05	1	U	■					R.TTSYKDVDGVPQK.G
1188		720.9106	1439.8066	1439.8096	-0.0030	0	30	0.0046	1				■		■	K.AQIIQQAGNSVLAK.A
1229		747.9180	1493.8214	1493.8202	0.0013	0	58	1e-05	1					■	■	K.ANQVPQQVLSLLQG.-
1260		770.8658	1539.7170	1539.7205	-0.0035	0	74	3.9e-08	1	U	■					K.YYVAITSTEHNDK.N
1261		514.2465	1539.7177	1539.7205	-0.0028	0	37	0.00022	1	U	■					K.YYVAITSTEHNDK.N
1285		788.4165	1574.8184	1574.8152	0.0033	1	104	4e-11	1	U	■					K.IGGADGKTETVTIGEK.T
1286		525.9470	1574.8192	1574.8152	0.0040	1	55	3.4e-06	1	U	■					K.IGGADGKTETVTIGEK.T
1310		538.9438	1613.8096	1613.8121	-0.0025	1	44	0.00036	1		■	■	■	■	■	R.INSAKDDAAGQAIANR.F
1311		807.9127	1613.8108	1613.8121	-0.0013	1	85	2.6e-08	1		■	■	■	■	■	R.INSAKDDAAGQAIANR.F
1339		836.3790	1670.7434	1670.7457	-0.0023	0	44	0.00026	1				■	■	■	R.IQDADYATEVSNMSK.A
1348		842.3524	1682.6902	1682.6948	-0.0045	0	101	7.9e-11	1	U	■					K.VGDDYYAADYESTSK.T
1358		850.8738	1699.7330	1699.7359	-0.0029	0	133	7.2e-14	1		■	■				R.IEDADYATEVSNMSR.A
1365		858.8710	1715.7274	1715.7308	-0.0034	0	110	2e-11	1		■	■				R.IEDADYATEVSNMSR.A + Oxidation (M)
1370		860.3577	1718.7008	1718.7974	-0.0965	0	1	0.75	1	U		■				K.ALAYNDAPMSVYFGGK.N + Oxidation (M)
1440		600.6660	1798.9762	1798.9789	-0.0027	1	29	0.0027	1	U	■					K.EINSQTLGLDKLNVQK.A
1441		900.4958	1798.9770	1798.9789	-0.0018	1	60	1.8e-06	1	U	■					K.EINSQTLGLDKLNVQK.A
1475		926.4446	1850.8746	1850.8786	-0.0039	0	111	7.3e-12	1	U	■					K.VDTTTYTTTYTDGTAIK.N
1535		972.4410	1942.8674	1942.8690	-0.0016	1	70	1.6e-07	1		■	■				R.SRIEDADYATEVSNMSR.A
1536		648.6299	1942.8679	1942.8690	-0.0012	1	57	3.1e-06	1		■	■				R.SRIEDADYATEVSNMSR.A
1539		653.9606	1958.8600	1958.8640	-0.0040	1	46	5.4e-05	1		■	■				R.SRIEDADYATEVSNMSR.A + Oxidation (M)
1577		1019.5130	2037.0114	2037.0127	-0.0012	0	97	2.1e-10	1	U	■					R.FDSTITNLGNTLNNLSSAR.S
1578		680.0112	2037.0118	2037.0127	-0.0009	0	15	0.031	1	U	■					R.FDSTITNLGNTLNNLSSAR.S
1603		1043.0670	2084.1194	2084.1225	-0.0031	0	127	1.3e-12	1	U	■	■		■		M.AQVINTNSLSLiTQNNiNK.N
1603		1043.0670	2084.1194	2085.1066	-0.9871	0	67	1.4e-06	5	U					■	M.AQVINTNSLSLITQNNIDK.N
1604		695.7150	2084.1232	2084.1225	0.0006	0	71	4.8e-07	1	U	■	■		■		M.AQVINTNSLSLiTQNNiNK.N
1604		695.7150	2084.1232	2085.1066	-0.9834	0	67	1.2e-06	4						■	M.AQVINTNSLSLITQNNIDK.N
1635		1094.5520	2187.0894	2187.0907	-0.0012	0	99	1.3e-10	1	U	■					K.QATLGEEATTTTVNPLDAIDK.A
1636		730.0375	2187.0907	2187.0907	0.0000	0	48	1.6e-05	1	U	■					K.QATLGEEATTTTVNPLDAIDK.A
1681		1150.0920	2298.1694	2298.1703	-0.0009	0	136	2.5e-14	1	U	■					K.AALATDVNNASSIGVSDAIPGDIK.F
1682		767.0646	2298.1720	2298.1703	0.0017	0	77	1.8e-08	1	U	■					K.AALATDVNNASSIGVSDAIPGDIK.F
1712		808.0598	2421.1576	2421.1660	-0.0084	1	53	4.8e-06	1	U	■					K.NGDYEITVDKDGSAALVAGQSSPK.S
1748	1	1315.1450	2628.2754	2628.2739	0.0015	0	119	6e-12	1		■	■	■			R.NANDGISVAQTTEGALSEINNNLQR
1749		877.0998	2628.2776	2628.2739	0.0037	0	76	1.1e-07	1		■	■	■			R.NANDGISVAQTTEGALSEINNNLQR
1761		669.5662	2674.2357	2674.2398	-0.0041	1	28	0.0016	1	U	■					K.YYVAITSTEHNDKNGDYEITVDK.D
1762		892.4198	2674.2376	2674.2398	-0.0023	1	60	1e-06	1	U	■					K.YYVAITSTEHNDKNGDYEITVDK.D
1784		1023.1770	3066.5092	3066.5105	-0.0014	0	70	1.1e-07	1	U	■					K.NVTAYQVANTQSNTQSVDATVSAGAISELK.T
1785		1534.2620	3066.5094	3066.5105	-0.0011	0	53	4.6e-06	1	U	■					K.NVTAYQVANTQSNTQSVDATVSAGAISELK.T

36 subsets and intersections (166 subset proteins in total)

10 per page 1

Not what you expected? Try **the select summary**.

Mascot: http://www.matrixscience.com/
