# Supplementary material for: MS-H: A Novel Proteomic Approach to Isolate and Type the E. coli H Antigen Using Membrane Filtration and Liquid Chromatography-Tandem Mass Spectrometry (LC-MS/MS)
Source: PLoS One. 2013 Feb 21;8(2):e57339. doi: 10.1371/journal.pone.0057339 (PMC3578835; doi:10.1371/journal.pone.0057339)
Supplement: Representative Peptide Data S1 — Peptide data are represented as the Mascot search results from all 53 serotypes, obtained under the Orbitrap platform in Table 4 with related E. coli reference strains. “U” denotes a unique peptide specific for each of the proteins 1.1, 1.2, and beyond. The number 1.1 (shown as 1 in the peptide list and phylogenetic tree) represents the protein which obtained the highest score and confidence value after a Mascot search. This protein, known as the first hit, was used to designate the MS-H type of the unknown flagellin. Related peptides 1.2 (2), 1.3 (3), etc. represented the second, third, etc. hits for MS-H typing analysis. (DOCX) [file pone.0057339.s009.docx › H55-E375M.pdf]

MATRIX SCIENCE MASCOT Search Results

User :
E-mail :
Search title : Submitted from 20110901-0623 by Mascot Daemon on VARIABLE
MS data file : C:\Documents and Settings\keding\Desktop\Raw data\20110901-002-0031-00623\20110901-010-EC375-MS1-RP-r.RAW
Database : Flagellin_v2 (192 sequences; 89,845 residues)
Taxonomy : Bacteria (Eubacteria) (192 sequences)
Timestamp : 4 Sep 2011 at 17:55:40 GMT

Not what you expected? Try **the select summary**.

Search parameters
Score distribution
Legend

Protein Family Summary

Significance threshold p< 0.05 Max. number of families AUTO
Ions score or expect cut-off 0 Dendrograms cut at 0

Protein families 1–2 (out of 2)

10 per page 1

1

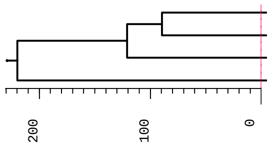

1	**gi|125659376**	2042	H55 0|H55|E2987-73
3	**gi|6941882**	948	H14 0|O138:H14|O138:H14
4	**gi|46093563**	401	H54 0|0|E223-69
2	**gi|30059924**	988	H49 0|H49|2147-59 [gi|315061223|ref|ADT75550| Hxx 0|0|W] [gi|323186300|ref|EFZ71650| ...

Threshold (**0**): 0

		Score	Mass	Matches	Sequences	emPAI
1.1	gi|125659376<br>H55 0|H55|E2987-73	2042	63681	42 (34)	33 (30)	4.26
1.2	gi|30059924<br>H49 0|H49|2147-59 [gi|315061223|ref|ADT75550| Hxx 0|0|W] [gi|323186300|ref|EFZ71650| Hxx 0|0|1357] [gi|307314170|ref|ZP_07593780| Hxx 0|0|W] [gi|323378200|ref|ADX50468| Hxx 0|0|K...<br>**1 sameset of gi|30059924**	988	57964	24 (16)	20 (15)	1.42
1.3	gi|6941882<br>H14 0|O138:H14|O138:H14	948	56492	26 (17)	23 (16)	1.62
1.4	gi|46093563<br>H54 0|0|E223-69	401	54419	18 (8)	14 (6)	0.51

68 peptide matches (61 non-duplicate, 7 duplicate)

Query	Dupes	Observed	Mr(expt)	Mr(calc)	Delta	M	Score	Expect	Rank	U	1	2	3	4	Peptide
39		316.6901	631.3656	631.3653	0.0003	0	31	0.0071	1		■	■	■	■	R.LSSGLR.I
122		347.2004	692.3862	692.3857	0.0005	0	5	0.74	2	U				■	R.FTANIK.G
147		355.1976	708.3806	708.3806	0.0000	0	7	1.1	1		■	■	■		R.FTSNIK.G
153		358.7061	715.3976	715.3977	-0.0000	0	31	0.0063	1		■	■	■		K.GLTQAAR.N
194	1	380.6953	759.3760	759.3763	-0.0002	0	35	0.0016	1		■	■	■		R.LDEIDR.V
194		380.6953	759.3760	758.3922	0.9838	0	17	0.11	2	U				■	R.LNEIDR.V
375	1	430.7267	859.4388	859.4399	-0.0011	0	27	0.0019	1	U	■				K.AQDVNVSK.D
435		447.7187	893.4228	892.4290	0.9938	0	1	0.72	1	U			■		K.VDQAAFDK.A
454		452.3256	902.6366	902.5073	0.1294	0	8	0.15	1	U			■		K.AATTADILK.A
455		453.3237	904.6328	904.4502	0.1827	0	2	0.64	1	U			■		K.AATTADDLK.A
504		466.2526	930.4906	930.4883	0.0024	0	82	2.6e-08	1		■		■		R.SSLGAVQNR.L
531		473.2532	944.4918	944.5039	-0.0121	0	46	7.1e-05	1	U		■			R.SSLGAIQNR.L
587	1	489.2239	976.4332	976.5077	-0.0744	0	4	0.38	1	U				■	K.TETVTIGEK.T
598		493.7845	985.5544	985.5556	-0.0012	0	96	2.7e-10	1	U	■				K.AAASNVLAAAK.N
635		504.7447	1007.4748	1007.4771	-0.0023	0	30	0.00098	1	U	■				K.DGTITTTDGK.S
759		551.2667	1100.5188	1100.5210	-0.0022	0	71	7.2e-07	1		■	■	■	■	K.DDAAGQAIANR.F
856		386.2155	1155.6247	1154.6295	0.9951	1	2	0.69	1	U				■	K.TVTVRTTSYK.D
863		581.2986	1160.5826	1159.5179	1.0647	1	2	0.92	1	U				■	K.MTYTDSNGKK.V + Oxidation (M)
869		582.8214	1163.6282	1163.5782	0.0500	0	4	1.1	1	U				■	K.SQSSLSSAIER.L
886	2	588.2853	1174.5560	1174.5578	-0.0018	0	44	4.1e-05	1	U				■	K.TGGVDNTAAGNAK.L
906		396.8716	1187.5930	1187.6034	-0.0104	0	4	0.43	1	U	■				K.ALDDAISQIDK.F
907	1	594.8064	1187.5982	1187.6034	-0.0051	0	81	7.2e-09	1	U	■				K.ALDDAISQIDK.F
915		598.8000	1195.5854	1194.5517	1.0338	0	3	0.55	1	U		■			K.DAAQSSIDFGGK.K
962		612.2874	1222.5602	1222.5612	-0.0009	0	62	5.8e-07	1	U	■				K.NQSSMSTAIER.L
979		620.2839	1238.5532	1238.5561	-0.0028	0	66	2.3e-07	1	U	■				K.NQSSMSTAIER.L + Oxidation (M)
998		627.8040	1253.5934	1254.6244	-1.0310	0	5	0.34	1	U		■			K.FNALDAATAFSK.L
1108		448.2479	1341.7219	1341.7252	-0.0033	0	3	0.48	1	U			■		K.ADLVAANATVVGNK.Y
1127		683.3458	1364.6770	1364.6783	-0.0013	0	86	2.6e-09	1	U	■				K.GSVSNTAATTDTLK.L
1154		696.8161	1391.6176	1391.6205	-0.0028	0	116	2.8e-12	1	U	■				K.ASDSYSFSATAASK.D
1197		720.9106	1439.8066	1439.8096	-0.0030	0	111	3.2e-11	1		■	■	■		K.AQIIQQAGNSVLAK.A
1236		497.9313	1490.7721	1490.7729	-0.0008	1	38	0.00014	1	U	■				K.ALDDAISQIDKFR.S
1239		747.9175	1493.8204	1493.8202	0.0003	0	54	2.7e-05	1		■	■	■		K.ANQVPQQVLSLLQG.-

Query	Dupes	Observed	Mr(expt)	Mr(calc)	Delta	M	Score	Expect	Rank	U	1	2	3	4	Peptide
1295		781.4198	1560.8250	1560.8260	-0.0010	0	88	7.5e-09	1		■	■	■		R.VSGQTQFNGVNVLAK.D
1330		538.9438	1613.8096	1613.8121	-0.0025	1	18	0.14	1		■	■	■	■	R.INSAKDDAAGQAIANR.F
1356		836.3778	1670.7410	1670.7457	-0.0047	0	118	9.4e-12	1		■	■	■		R.IQDADYATEVSNMSK.A
1362		840.4338	1678.8530	1678.8526	0.0004	0	72	6.2e-08	1	U	■				K.IDSSTLNLTGFNVNGK.G
1368		844.4356	1686.8566	1685.8836	0.9731	0	12	0.21	1	U	■				K.IQVGANDGETITIDLK.K
1370		844.9832	1687.9518	1686.7407	1.2112	0	2	1.3	1		■	■	■		R.IQDADYATEVSNMSK.A + Oxidation (M)
1387		859.9427	1717.8708	1717.8734	-0.0025	0	110	1e-11	1	U	■				K.LTTEASTAAETTANPLK.A
1458		599.6505	1795.9297	1795.8873	0.0423	1	3	0.56	1	U			■		K.LTTDAETKAATTADMLK.A + Oxidation (M)
1465		603.3223	1806.9451	1806.9476	-0.0025	1	58	1.6e-06	1	U	■				K.KIDSSTLNLTGFNVNGK.G
1466		904.4800	1806.9454	1806.9476	-0.0021	1	110	1e-11	1	U	■				K.KIDSSTLNLTGFNVNGK.G
1523		949.9813	1897.9480	1897.9422	0.0059	0	60	1e-06	1	U	■				K.LAGFTAGATPAADGTVTYSK.D
1527		636.3226	1905.9460	1905.9466	-0.0006	1	95	2.9e-10	1	U	■				K.VKDMTITSAGGNAQVATDK.A
1531		956.6984	1911.3822	1912.0126	-0.6304	1	3	0.48	1	U				■	K.ALAQVDSLRSDLGAVQNR.F
1617		1043.0700	2084.1254	2084.1225	0.0029	0	120	6.3e-12	1		■	■	■	■	M.AQVINTNSLSLiTQNNiNK.N
1707		750.3707	2248.0903	2248.0931	-0.0028	0	71	4.9e-07	1		■	■	■		R.LDSAVTNLNNTTTNLSEAQSR.I
1708		1125.0530	2248.0914	2248.0931	-0.0017	0	124	2.4e-12	1		■	■	■		R.LDSAVTNLNNTTTNLSEAQSR.I
1732		767.0621	2298.1645	2298.1703	-0.0058	0	18	0.017	1	U				■	K.AALATDVNNASSIGVSDAIPGDIK.F
1735		1150.0920	2298.1694	2298.1703	-0.0009	0	103	5e-11	1	U				■	K.AALATDVNNASSIGVSDAIPGDIK.F
1762		1210.1370	2418.2594	2418.2602	-0.0007	0	116	2.4e-12	1	U		■			K.AATISDLAATGANVTNSSNIVVTTK.F
1775	1	826.3774	2476.1104	2476.1142	-0.0039	0	94	4e-10	1	U	■				K.YYAHTNGSVTNDSGSAIYATEAGK.L
1790		862.0937	2583.2593	2583.2891	-0.0298	0	3	0.52	1	U		■			K.AFLVGGALTTNDPTGSTPATMSSLFK.A
1793		1298.1170	2594.2194	2594.2195	-0.0001	0	110	9.1e-12	1	U	■				R.ELTVQSSTGTNSESDLNSIQDEIK.S
1796		867.4567	2599.3483	2599.2840	0.0643	0	2	0.63	1	U		■			K.AFLVGGALTTNDPTGSTPATMSSLFK.A + Oxidation (M)
1797		1315.1450	2628.2754	2628.2739	0.0015	0	117	8.5e-12	1		■	■	■	■	R.NANDGISVAQTTEGALSEINNNLQR
1810		893.4659	2677.3759	2677.3043	0.0716	1	15	0.03	1	U			■		K.GTITIDGSAQDVQISSDGKITASNGDK.L
1825		955.4760	2863.4062	2863.4047	0.0015	1	69	1.4e-07	1	U	■				R.IRELTVQSSTGTNSESDLNSIQDEIK.S
1842		1031.8560	3092.5462	3092.5448	0.0014	1	39	0.00055	1		■	■	■		R.IQDADYATEVSNMSKAQIIQQAGNSVLAK.A
1854		1054.5320	3160.5742	3160.5708	0.0033	1	44	0.00013	1		■		■		R.SSLGAVQNRLDSAVTNLNNTTTNLSEAQSR.I
1858		1086.5750	3256.7032	3256.7011	0.0021	1	106	9.5e-11	1			■	■		M.AQVINTNSLSLITQNNINKNQSALSSSIER.L

53 subsets and intersections (166 subset proteins in total)

---

2 gi|112820172 16 H21 0|EHEC serogroup: O113:H21|0

10 per page 1

Not what you expected? Try the select summary.

Mascot: http://www.matrixscience.com/
