# Supplementary material for: MS-H: A Novel Proteomic Approach to Isolate and Type the E. coli H Antigen Using Membrane Filtration and Liquid Chromatography-Tandem Mass Spectrometry (LC-MS/MS)
Source: PLoS One. 2013 Feb 21;8(2):e57339. doi: 10.1371/journal.pone.0057339 (PMC3578835; doi:10.1371/journal.pone.0057339)
Supplement: Representative Peptide Data S1 — Peptide data are represented as the Mascot search results from all 53 serotypes, obtained under the Orbitrap platform in Table 4 with related E. coli reference strains. “U” denotes a unique peptide specific for each of the proteins 1.1, 1.2, and beyond. The number 1.1 (shown as 1 in the peptide list and phylogenetic tree) represents the protein which obtained the highest score and confidence value after a Mascot search. This protein, known as the first hit, was used to designate the MS-H type of the unknown flagellin. Related peptides 1.2 (2), 1.3 (3), etc. represented the second, third, etc. hits for MS-H typing analysis. (DOCX) [file pone.0057339.s009.docx › H56-E376.pdf]

MASCOT Search Results

User :
E-mail :
Search title : Submitted from 20110824-0616-02 by Mascot Daemon on VARIABLE
MS data file : C:\Documents and Settings\keding\Desktop\Raw data\20110825-001-0013-00616\20110825-012-EC376-MS2.RAW
Database : Flagellin_v2 (192 sequences; 89,845 residues)
Taxonomy : Bacteria (Eubacteria) (192 sequences)
Timestamp : 26 Aug 2011 at 20:04:29 GMT

Not what you expected? Try **the select summary**.

Search parameters

Score distribution

Legend

Protein Family Summary

Significance threshold p< 0.05 Max. number of families AUTO
Ions score or expect cut-off 0 Dendrograms cut at 0

Protein family 1 (out of 1)

10 per page 1

1

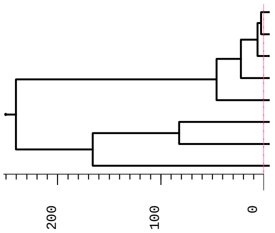

1	**gi|30059930**	860	H56 0|H56|SN3N/1
7	**gi|169754667**	424	Hxx(H3306 97.9%) 0|0|ATCC 8739 [gi|170019739|ref|YP_001724693| Hxx 0|0|ATCC 8739]
4	**gi|33590260**	651	H29 0|O157:H29|Y63
5	**gi|120312**	569	Hxx(H48 100.0%) 0|0|0 [gi|6009847|ref|BAA85088| H48 0|H48|P4] [gi|30059922|ref|AAP...
6	**gi|16901500**	467	H49 0|H49|0
2	**gi|154710458**	816	H47 0|H47|0
8	**gi|33590243**	422	H16 NM|O157:NM(H16)|PC02
3	**gi|112820171**	651	H21 0|EHEC serogroup: O113:H21|0 [gi|291323135|ref|EFE62563| Hxx 0|0|B088] [gi|2934...

Threshold (**0**): 0

		Score	Mass	Matches	Sequences	emPAI
1.1	gi|30059930	860	45219	30 (19)	18 (15)	2.32
	H56 0|H56|SN3N/1					
1.2	gi|154710458	816	39177	28 (20)	18 (11)	1.87
	H47 0|H47|0					
1.3	gi|112820171	651	51472	22 (18)	12 (10)	1.24
	H21 0|EHEC serogroup: O113:H21|0 [gi|291323135|ref|EFE62563| Hxx 0|0|B088] [gi|293446305|ref|ZP_06662727| Hxx 0|0|B088]					
1.4	gi|33590260	651	45720	24 (16)	14 (12)	1.65
	H29 0|O157:H29|Y63					
1.5	gi|120312	569	51265	27 (19)	13 (10)	1.11
	Hxx(H48 100.0%) 0|0|0 [gi|6009847|ref|BAA85088| H48 0|H48|P4] [gi|30059922|ref|AAP13333| H48 0|H48|P4] [gi|1788232|ref|AAC74990| H48 0|0|K-12] [gi|89108758|ref|AP_002538| Hxx 0...					
	**2 samesets of gi|120312**					
1.6	gi|16901500	467	57940	18 (11)	13 (9)	0.74
	H49 0|H49|0					
	**3 samesets of gi|16901500**					
1.7	gi|169754667	424	58285	18 (11)	13 (9)	0.73
	Hxx(H3306 97.9%) 0|0|ATCC 8739 [gi|170019739|ref|YP_001724693| Hxx 0|0|ATCC 8739]					
	**2 samesets of gi|169754667**					
1.8	gi|33590243	422	55093	16 (10)	10 (7)	0.59
	H16 NM|O157:NM(H16)|PC02					
	**2 samesets of gi|33590243**					

82 peptide matches (56 non-duplicate, 26 duplicate)

Query	Dupes	Observed	Mr(expt)	Mr(calc)	Delta	M	Score	Expect	Rank	U	1	2	3	4	5	6	7	8	Peptide
13		308.2118	614.4090	615.3592	-0.9501	0	1	1.4	1	U	■								K.DQLIK.V
22		316.6913	631.3680	631.3653	0.0027	0	24	0.034	1		■	■	■	■	■	■	■	■	R.LSSGLR.I
108		355.1988	708.3830	708.3806	0.0024	0	17	0.12	1		■	■		■	■	■	■		R.FTSNIK.G
114		358.7075	715.4004	715.3977	0.0028	0	28	0.011	1		■			■	■	■	■		K.GLTQAAR.N
145		380.2047	758.3948	758.4174	-0.0225	0	30	0.0055	1	U								■	K.LDEALAK.V
146	1	380.6963	759.3780	759.3763	0.0018	0	33	0.0027	1		■	■		■	■	■	■		R.LDEIDR.V
215	1	403.7175	805.4204	805.4182	0.0023	0	18	0.014	1	U	■								K.DLTTATGK.T
397		452.2493	902.4840	902.5437	-0.0596	1	4	0.43	1	U		■							K.KIDSTVLK.L
435		308.1688	921.4846	920.4200	1.0646	0	16	0.025	1	U							■		K.DGSVTNGSGK.A
452	3	466.2522	930.4898	930.4883	0.0016	0	91	3.7e-09	1		■			■	■			■	R.SSLGAVQNR
657	1	502.2411	1002.4676	1002.5094	-0.0418	1	8	0.74	1		■			■	■	■	■		K.SRLDEIDR.V
659		335.1783	1002.5131	1002.5094	0.0037	1	19	0.078	1		■			■	■	■	■		K.SRLDEIDR.V
691	1	510.7778	1019.5410	1019.5400	0.0011	0	54	6.2e-06	1	U	■								K.AIAQVDTFR.S
737		522.2841	1042.5536	1043.6339	-1.0802	1	9	0.14	1	U		■							K.IDSTVLKLR.D
863		551.2688	1100.5230	1100.5210	0.0020	0	74	3.4e-07	1		■	■	■	■	■	■	■	■	K.DDAAGQAIANR.F

Query	Dupes	Observed	Mr(expt)	Mr(calc)	Delta	M	Score	Expect	Rank	U	1	2	3	4	5	6	7	8	Peptide
1097		598.8027	1195.5908	1194.5517	1.0392	0	10	0.11	1	U						■			K.DAAQSSIDFGGK.K
1123		603.3109	1204.6072	1204.6048	0.0025	0	58	3e-06	1		■			■					K.NQSALSTSIER.L
1148		406.5233	1216.5481	1217.5888	-1.0407	0	15	0.033	1	U				■					R.VTIDGDGTNQAK.I
1155		609.7866	1217.5586	1217.5888	-0.0302	0	19	0.012	1	U				■					R.VTIDGDGTNQAK.I
1190		617.7870	1233.5594	1233.6201	-0.0607	1	1	3.1	1	U		■							K.GDIKDSTSVQGK.F
1257		424.5656	1270.6750	1270.5942	0.0808	0	2	0.59	1	U							■		K.NGFAAGATSNAYK.L
1370		668.3247	1334.6348	1334.6314	0.0035	0	99	1.3e-10	1	U	■								K.DTDNGLTTAATQK.D
1392		675.3400	1348.6654	1347.6882	0.9773	1	2	0.6	1	U		■							K.TKVVDEDATAATK.T
1445		463.9131	1388.7175	1387.7195	0.9980	0	2	0.62	1	U		■							K.VTVDLADAAGDLTK.T
1547		728.9109	1455.8072	1455.8045	0.0027	0	99	1.9e-10	1		■			■					K.AQIIQQAGNSVLSK.A
1548		486.6817	1457.0233	1455.8045	1.2188	0	1	1.3	1		■			■					K.AQIIQQAGNSVLSK.A
1667		777.8845	1553.7544	1553.7474	0.0070	0	59	1.2e-06	1	U						■			K.YAANVGAQAYVGADGK.L
1671		781.4227	1560.8308	1560.8260	0.0048	0	70	4.5e-07	1		■			■	■	■	■		R.VSGQTQFNGVNVLAK
1736		538.9455	1613.8147	1613.8121	0.0026	1	32	0.0061	1		■	■	■	■	■	■	■	■	R.INSAKDDAAGQAIANR.F
1810		836.3824	1670.7502	1670.7457	0.0045	0	108	8.7e-11	1		■			■	■	■	■		R.IQDADYATEVSNMSK.A
1829		843.4590	1684.9034	1684.8996	0.0039	0	9	0.54	1							■	■		K.IQVGANDGQTITIDLK.K
1861		571.9736	1712.8990	1712.8945	0.0045	0	33	0.00056	1	U	■								K.TVDVSSLTLHNTLDAK.G
1862		857.4578	1712.9010	1712.8945	0.0066	0	80	9.9e-09	1	U	■								K.TVDVSSLTLHNTLDAK.G
1868		860.3600	1718.7054	1718.7974	-0.0919	0	2	0.69	1	U								■	K.ALAYNDAPMSVYFGGK.N + Oxidation (M)
1869		574.0743	1719.2011	1718.7974	0.4037	0	4	0.44	1	U								■	K.ALAYNDAPMSVYFGGK.N + Oxidation (M)
1888		581.3087	1740.9043	1740.9006	0.0037	0	38	0.00017	1	U		■							K.IQVGANDNQSIDINLK.K
1888		581.3087	1740.9043	1741.9210	-1.0167	0	13	0.045	2	U					■				K.IQVGANDNQTITIDLK.Q
1893	6	871.4608	1740.9070	1740.9006	0.0064	0	80	9.7e-09	1	U		■							K.IQVGANDNQSIDINLK.K
1895	5	871.4612	1740.9078	1741.9210	-1.0132	0	35	0.00029	2	U					■				K.IQVGANDNQTITIDLK.Q
1914		586.3214	1755.9424	1755.9367	0.0057	0	33	0.0011	1	U			■						K.IQVGANDGETITINLAK.I
1915	3	878.9785	1755.9424	1755.9367	0.0058	0	49	2.4e-05	1	U			■						K.IQVGANDGETITINLAK.I
1933		592.9547	1775.8423	1776.9330	-1.0907	1	1	3.9	1			■	■					■	K.SQSSLSSAIERLSSGLR.I
2039		624.0076	1869.0010	1868.9956	0.0054	1	15	0.031	1	U		■							K.IQVGANDNQSIDINLKK.I
2071		639.1391	1914.3955	1914.0132	0.3823	1	3	0.49	1	U								■	K.VPMSSAVALKSEAAPDLTK.V
2139	2	1021.0310	2040.0474	2040.0415	0.0059	0	73	5.2e-08	1	U			■						K.IVYEGIEFTNTGTVAIDAK.G
2141		681.0237	2040.0493	2040.0415	0.0077	0	56	2.5e-06	1	U			■						K.IVYEGIEFTNTGTVAIDAK.G
2158		695.7160	2084.1262	2084.1225	0.0036	0	61	5.5e-06	1		■	■	■	■	■	■	■	■	M.AQVINTNSLSLiTQNNiNK.N
2159	1	1043.0710	2084.1274	2084.1225	0.0049	0	99	9e-10	1		■	■	■	■	■	■	■	■	M.AQVINTNSLSLiTQNNiNK.N
2169		712.3446	2134.0120	2134.0066	0.0053	0	19	0.012	1	U			■						K.QVYVSTADGSLTTSSDTQFK.I
2200		565.7682	2259.0437	2259.1529	-0.1092	1	2	0.67	1	U					■				K.NGSMKIQVGANDNQTITIDLK.Q
2220		1308.6050	2615.1954	2615.1835	0.0120	0	66	2.2e-07	1	U		■							K.STTTDNNGIYAASVDSDGNVTIDASK.K
2223	1	892.7812	2675.3218	2675.3178	0.0040	0	11	0.087	1	U			■						K.AVEFTISGSTDTSGTSATVAPTTALYK.N
2226		915.4374	2743.2904	2743.2784	0.0119	1	60	9.2e-07	1	U		■							K.STTTDNNGIYAASVDSDGNVTIDASKK.V
2227		956.2012	2865.5818	2865.5672	0.0146	0	28	0.0015	1			■	■						R.AQILQQAGTSVLAQANQTTQNVLSLLR.-
2230		1063.4980	3187.4722	3187.4542	0.0180	1	80	1.1e-08	1	U			■						K.ATGTDNYQINGTDNYTVNVDSGVVQDKDGK.Q
2231		1077.5730	3229.6972	3229.6902	0.0070	1	69	1.7e-07	1			■	■					■	M.AQVINTNSLSLLTQNNLNKSQSSLSSAIER.L

40 subsets and intersections (156 subset proteins in total)

10 per page 1

Not what you expected? Try **the select summary**.

Mascot: http://www.matrixscience.com/
