# Supplementary material for: MS-H: A Novel Proteomic Approach to Isolate and Type the E. coli H Antigen Using Membrane Filtration and Liquid Chromatography-Tandem Mass Spectrometry (LC-MS/MS)
Source: PLoS One. 2013 Feb 21;8(2):e57339. doi: 10.1371/journal.pone.0057339 (PMC3578835; doi:10.1371/journal.pone.0057339)
Supplement: Representative Peptide Data S1 — Peptide data are represented as the Mascot search results from all 53 serotypes, obtained under the Orbitrap platform in Table 4 with related E. coli reference strains. “U” denotes a unique peptide specific for each of the proteins 1.1, 1.2, and beyond. The number 1.1 (shown as 1 in the peptide list and phylogenetic tree) represents the protein which obtained the highest score and confidence value after a Mascot search. This protein, known as the first hit, was used to designate the MS-H type of the unknown flagellin. Related peptides 1.2 (2), 1.3 (3), etc. represented the second, third, etc. hits for MS-H typing analysis. (DOCX) [file pone.0057339.s009.docx › H1-E169.pdf]

MASCOT Search Results

User :
E-mail :
Search title : Submitted from 20110714-H1-H11 by Mascot Daemon on VARIABLE
MS data file : C:\Documents and Settings\keding\Desktop\Raw data\20110714-H1-H11\20110714-003-E169MS1.RAW
Database : Flagellin_v2 (192 sequences; 89,845 residues)
Taxonomy : Bacteria (Eubacteria) (192 sequences)
Timestamp : 15 Jul 2011 at 17:18:35 GMT

Not what you expected? Try **the select summary**.

Search parameters

Score distribution

Legend

Protein Family Summary

Significance threshold p< 0.05 Max. number of families AUTO
Ions score or expect cut-off 0 Dendrograms cut at 0

Protein family 1 (out of 1)

10 per page 1

1

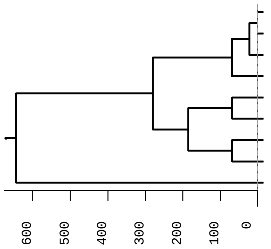

1	gi|6009831	14309	Hxx(H1 100.0%) 0|0|Su 1242 [gi|312946515|ref|ADR27342| H1 0|O83:H1|NRG 857C] [gi|3075...
9	gi|33590252	977	Hxx(H8 89.5%) 0|0|PC41
4	gi|88683097	3178	H44 0|H44|0
8	gi|323174561	1555	Hxx(H25 99.7%) 0|0|LT-68
3	gi|192955539	3462	H9 0|O111:H9|E110019 [gi|193071144|ref|ZP_03052068| H9 0|O111:H9|E110019]
7	gi|30577587	1673	H17 0|O15:H17|P12b
5	gi|121144335	2481	H55 0|O75:H55|E2987-73
6	gi|33590227	1890	H42 NM|O157:NM(H42)|25
2	gi|5107847	9528	H12 0|O111:H12|DEC 6a

Threshold (**0**): 0

		Score	Mass	Matches	Sequences	emPAI
1.1	gi|6009831	14309	60901	282 (254)	57 (55)	65.99
	Hxx(H1 100.0%) 0|0|Su 1242 [gi|312946515|ref|ADR27342| H1 0|O83:H1|NRG 857C] [gi|307553937|ref|ADN46712| Hxx 0|0|ABU 83972] [gi|301050721|ref|ZP_07197581| Hxx 0|0|MS 185-1] [gi|30...					
1.2	gi|5107847	9528	57823	214 (188)	46 (44)	28.24
	H12 0|O111:H12|DEC 6a					
1.3	gi|192955539	3462	68106	109 (87)	28 (24)	4.19
	H9 0|O111:H9|E110019 [gi|193071144|ref|ZP_03052068| H9 0|O111:H9|E110019]					
	**3 samesets of gi|192955539**					
1.4	gi|88683097	3178	55289	89 (70)	24 (18)	3.25
	H44 0|H44|0					
1.5	gi|121144335	2481	62285	81 (61)	23 (18)	2.62
	H55 0|O75:H55|E2987-73					
1.6	gi|33590227	1890	44094	61 (48)	16 (13)	2.67
	H42 NM|O157:NM(H42)|25					
1.7	gi|30577587	1673	36285	61 (47)	16 (12)	3.82
	H17 0|O15:H17|P12b					
1.8	gi|323174561	1555	46392	60 (43)	18 (12)	2.22
	Hxx(H25 99.7%) 0|0|LT-68					
	**1 sameset of gi|323174561**					
1.9	gi|33590252	977	52373	55 (25)	10 (5)	0.53
	Hxx(H8 89.5%) 0|0|PC41					

403 peptide matches (120 non-duplicate, 283 duplicate)

Query	Dupes	Observed	Mr(expt)	Mr(calc)	Delta	M	Score	Expect	Rank	U	1	2	3	4	5	6	7	8	9	Peptide
9		303.1740	604.3334	603.3228	1.0106	0	2	0.63	1	U									■	K.VVGSDK.T
35	7	316.6913	631.3680	631.3653	0.0027	0	33	0.0045	1		■	■	■	■	■	■	■	■	■	R.LSSGLR.I
53		330.7003	659.3860	659.3602	0.0258	0	6	0.25	1	U			■							K.QGNLTK.T
56	1	337.2155	672.4164	673.3759	-0.9594	0	9	0.14	1	U								■		K.NGATALK.L
72	1	344.2059	686.3972	686.3963	0.0010	0	37	0.00021	1		■	■								K.ALDQLK.D
87	1	352.2028	702.3910	702.3912	-0.0001	0	21	0.011	1		■	■								K.ETVLNK.V
92		352.7009	703.3872	704.3705	-0.9832	0	14	0.055	1	U				■						K.DGTLTAK.D
92		352.7009	703.3872	702.3912	0.9961	0	3	0.69	3	U								■		K.AIASVDK.F
98	1	355.1977	708.3808	708.3806	0.0002	0	21	0.047	1		■	■	■	■	■	■	■	■		R.FTSNIK.G
104	2	358.7076	715.4006	715.3977	0.0030	0	32	0.0042	1		■	■	■	■	■	■	■	■		K.GLTQAAR.N
130	4	380.6946	759.3746	759.3763	-0.0016	0	35	0.0016	1		■	■	■	■	■		■	■		R.LDEIDR.V
142		386.7194	771.4242	771.4490	-0.0248	0	1	0.77	1	U							■			K.ALDAAIAK.V
241	20	421.7588	841.5030	841.4658	0.0373	0	14	0.044	1	U									■	K.AVTQPQAK.D
259	2	424.7214	847.4282	847.4287	-0.0005	0	60	9.9e-07	1		■	■								K.DGDTVTIK.A
359	1	466.7448	931.4750	930.4883	0.9868	0	4	1.7	1				■	■	■		■	■		R.SSLGAVQNR.L
370		470.7607	939.5068	938.4934	1.0135	1	0	1	2	U				■						K.VAHKDANGK.I

Query	Dupes	Observed	Mr(expt)	Mr(calc)	Delta	M	Score	Expect	Rank	U	1	2	3	4	5	6	7	8	9	Peptide
379	4	473.2588	944.5030	944.5039	-0.0009	0	80	3.2e-08	1		■	■								R.SSLGAIQNR.L
429	1	491.2347	980.4548	980.4563	-0.0015	0	42	7e-05	1		■	■								K.YSIDANNGK.V
437		493.7534	985.4922	985.5556	-0.0634	0	6	0.26	1	U					■					K.AAASNVLAAAK.N
448		496.7970	991.5794	990.5234	1.0561	0	5	0.35	1	U									■	K.DLTDVTTVK.G
463	1	502.2610	1002.5074	1002.5094	-0.0020	1	36	0.0014	1		■	■	■	■	■		■	■		K.SRLDEIDR.V
466	1	335.1774	1002.5104	1002.5094	0.0010	1	37	0.001	1		■	■	■	■	■		■	■		K.SRLDEIDR.V
488	2	511.2606	1020.5066	1020.5088	-0.0021	0	72	9.8e-08	1		■	■								K.VTVDSGTGTGK.Y
552	1	531.2946	1060.5746	1060.5764	-0.0018	0	68	1.5e-07	1		■	■								K.AATISDLTAAK.M
598	3	547.2665	1092.5184	1092.5200	-0.0015	0	86	2.8e-09	1	U	■									K.AGDGQSIGFNK.T
617	6	551.2664	1100.5182	1100.5210	-0.0028	0	78	1.4e-07	1		■	■	■	■	■	■	■	■	■	K.DDAAGQAIANR.F
701		382.5594	1144.6564	1144.6564	-0.0000	1	6	2.2	1		■	■	■	■	■	■	■	■	■	R.LSSGLRINSAK.D
737	2	581.3035	1160.5924	1160.5925	-0.0000	0	95	3.9e-10	1		■	■	■							K.ALDEAISSIDK.F
778		396.8718	1187.5936	1187.6034	-0.0098	0	1	0.85	1	U					■					K.ALDDAISQIDK.F
785	2	596.3014	1190.5882	1190.5891	-0.0008	0	66	1.3e-06	1		■	■	■							K.NQSALSSSIER.L
834	1	407.8784	1220.6134	1220.6149	-0.0016	1	7	0.21	1	U	■									K.KAGDGQSIGFNK.T
836	2	611.3143	1220.6140	1220.6149	-0.0009	1	67	1.9e-07	1	U	■									K.KAGDGQSIGFNK.T
968		656.8646	1311.7146	1311.7146	0.0000	0	81	7.4e-09	1	U		■								K.AQIIQQAGNSVLA.-
1008		672.8770	1343.7394	1343.7408	-0.0014	0	83	4.7e-09	1	U					■					-.SLSLITQNNINK.N
1054		469.5957	1405.7653	1405.7677	-0.0025	1	11	0.67	1	U									■	R.FTANIKGLTQASR.N
1054		469.5957	1405.7653	1405.7677	-0.0025	1	6	2.5	2		■	■	■	■	■	■	■	■		R.FTSNIKGLTQAAR.N
1089	1	480.9430	1439.8072	1439.8096	-0.0024	0	5	1.3	1		■		■	■	■	■				K.AQIIQQAGNSVLAK.A
1094	4	720.9125	1439.8104	1439.8096	0.0008	0	103	2.3e-10	1		■		■	■	■	■				K.AQIIQQAGNSVLAK.A
1121	1	732.8880	1463.7614	1463.7620	-0.0005	1	86	3.4e-09	1		■	■	■							K.ALDEAISSIDKFR.S
1122	1	488.9278	1463.7616	1463.7620	-0.0004	1	57	2.4e-06	1		■	■	■							K.ALDEAISSIDKFR.S
1141	1	739.3537	1476.6928	1476.6919	0.0010	0	84	3.9e-09	1		■	■								K.TASVTMGGTTYNFK.T
1147	1	494.2580	1479.7522	1479.7569	-0.0048	1	21	0.0081	1		■	■								K.VTVDSGTGTGKYAPK.V
1149		740.8842	1479.7538	1479.7569	-0.0031	1	88	1.7e-09	1		■	■								K.VTVDSGTGTGKYAPK.V
1164	14	747.3465	1492.6784	1492.6794	-0.0009	0	107	1.3e-10	1		■	■								K.AASGEVNFDVDANGK.I
1173		747.3491	1492.6836	1492.6868	-0.0032	0	106	1.5e-10	1		■	■								K.TASVTMGGTTYNFK.T + Oxidation (M)
1176	1	747.9172	1493.8198	1493.8202	-0.0003	0	29	0.0077	1	U	■		■	■		■	■	■		K.ANQVPQQVLSLxQG.-
1206	2	506.2780	1515.8122	1515.8144	-0.0023	1	52	6e-06	1		■	■								K.ALDQLKDGDTVTIK.A
1207	2	758.9135	1515.8124	1515.8144	-0.0020	1	65	2.9e-07	1		■	■								K.ALDQLKDGDTVTIK.A
1213		506.9334	1517.7784	1517.7950	-0.0167	0	12	0.063	1	U				■						K.ANQVPQQVLSLHQG.-
1241		516.0306	1545.0700	1543.7995	1.2705	0	5	0.7	3	U				■						K.ANQVPQQVLSLYQG.-
1259	2	781.4207	1560.8268	1560.8260	0.0008	0	62	2.9e-06	1		■	■		■	■	■				R.VSGQTQFNGVNVLAK
1307	1	807.9122	1613.8098	1613.8121	-0.0023	1	99	1.2e-09	1		■	■	■	■	■	■	■	■	■	R.INSAKDDAAGQAIANR.F
1308	1	538.9440	1613.8102	1613.8121	-0.0019	1	42	0.00056	1		■	■	■	■	■	■	■	■	■	R.INSAKDDAAGQAIANR.F
1349	2	836.3809	1670.7472	1670.7457	0.0015	0	126	1.6e-12	1		■	■	■	■	■	■	■	■		R.IQDADYATEVSNMSK.A
1363	8	843.4572	1684.8998	1685.8836	-0.9837	0	68	5.6e-07	2				■		■					K.IQVGANDGETITIDLK.K
1365	10	843.4573	1684.9000	1684.8996	0.0005	0	118	6.6e-12	1		■	■		■						K.IQVGANDGQTITIDLK
1369		844.3776	1686.7406	1686.7407	-0.0000	0	111	5.5e-11	1		■	■	■	■	■	■	■	■		R.IQDADYATEVSNMSK.A + Oxidation (M)
1429	21	884.9388	1767.8630	1767.8639	-0.0008	1	58	1.7e-06	2	U		■								K.QGKAAAADGDTSATITYK.S
1441	20	884.9399	1767.8652	1767.8639	0.0013	0	125	3.2e-13	1	U	■									K.QGTAVAANGDTSATITYK.S
1460		896.4525	1790.8904	1790.8911	-0.0006	1	51	6e-05	1		■	■	■	■	■	■	■	■		K.DDAAGQAIANRFTSNIK.G
1461		597.9709	1790.8909	1790.8911	-0.0002	1	38	0.0012	1		■	■	■	■	■	■	■	■		K.DDAAGQAIANRFTSNIK.G
1479		605.3391	1812.9955	1812.9945	0.0009	1	54	1.6e-05	1		■	■								K.IQVGANDGQTITIDLKK.I
1479		605.3391	1812.9955	1813.9785	-0.9831	1	46	9.8e-05	2				■		■					K.IQVGANDGETITIDLKK.I
1545	1	643.6498	1927.9276	1927.9276	-0.0000	0	79	2.4e-08	1		■	■								K.SGVQTYQAVFAAGDGTASAK.Y
1547	9	964.9711	1927.9276	1927.9276	0.0001	0	149	2.3e-15	1		■	■								K.SGVQTYQAVFAAGDGTASAK.Y
1556	7	969.9737	1937.9328	1937.9364	-0.0036	0	145	3e-15	1		■	■								K.MDAATNTITTTNNALTASK.A
1558		646.9859	1937.9359	1937.9364	-0.0005	0	65	3.2e-07	1		■	■								K.MDAATNTITTTNNALTASK.A
1574		977.9726	1953.9306	1953.9313	-0.0007	0	156	2.6e-16	1		■	■								K.MDAATNTITTTNNALTASK.A + Oxidation (M)
1585	1	658.3488	1972.0246	1972.0266	-0.0020	0	32	0.00064	1	U	■									K.AGDVAASLLPPAGQTASGVYK.A
1586	1	987.0206	1972.0266	1972.0266	0.0001	0	78	1.7e-08	1	U	■									K.AGDVAASLLPPAGQTASGVYK.A
1595	12	992.4646	1982.9146	1982.9181	-0.0035	0	139	2e-14	1	U	■									K.TGADAGAATANAGVSFTDTASK.E
1597	1	661.9792	1982.9158	1982.9181	-0.0024	0	35	0.00048	1	U	■									K.TGADAGAATANAGVSFTDTASK.E
1610		661.9899	1982.9479	1982.9545	-0.0067	1	59	1.8e-06	1		■	■								K.YSIDANNGKVTVDSGTGTGK.Y
1660	1	695.7145	2084.1217	2084.1225	-0.0009	0	64	2.4e-06	1		■		■	■				■	■	M.AQVINTNSLSLiTQNNiNK.N
1660	1	695.7145	2084.1217	2085.0814	-0.9597	0	57	1.4e-05	4	U							■			M.AQVINTNSLSLNTQNNINK.N
1660	1	695.7145	2084.1217	2085.1066	-0.9849	0	47	0.00012	5	U						■				M.AQVINTNSLSLITQNNIDK.N
1663	7	1043.0690	2084.1234	2084.1225	0.0009	0	120	6.5e-12	1		■		■	■				■	■	M.AQVINTNSLSLiTQNNiNK.N
1663	6	1043.0690	2084.1234	2085.0814	-0.9580	0	87	1.5e-08	4	U							■			M.AQVINTNSLSLNTQNNINK.N
1663	6	1043.0690	2084.1234	2085.1066	-0.9831	0	80	6.4e-08	5	U						■				M.AQVINTNSLSLITQNNIDK.N
1699		1104.0490	2206.0834	2206.0866	-0.0031	0	135	5.8e-14	1		■	■								K.IDSDTLGLNGFNVNGSGTIANK.A
1716	3	1125.0550	2248.0954	2248.0931	0.0023	0	138	1e-13	1		■	■	■		■	■	■			R.LDSAVTNLNNTTTNLSEAQSR.I
1717	1	750.3727	2248.0963	2248.0931	0.0032	0	86	1.6e-08	1		■	■	■		■	■	■			R.LDSAVTNLNNTTTNLSEAQSR.I
1747		1152.1040	2302.1934	2302.1917	0.0017	1	91	3.8e-09	1		■	■		■	■					R.LDEIDRVSGQTQFNGVNVLAK.D
1748	2	768.4053	2302.1941	2302.1917	0.0023	1	59	5.9e-06	1		■	■		■	■					R.LDEIDRVSGQTQFNGVNVLAK.D
1775	1	779.0673	2334.1801	2334.1815	-0.0015	1	54	4.1e-06	1		■	■								K.KIDSDTLGLNGFNVNGSGTIANK.A
1777		1168.0990	2334.1834	2334.1815	0.0019	1	142	6.9e-15	1		■	■								K.KIDSDTLGLNGFNVNGSGTIANK.A
1808	3	1236.1130	2470.2114	2470.2075	0.0039	0	111	8e-12	1		■	■								K.VGAEVYVSANGTLTTDATSEGTVTK.D
1824	1	856.0711	2565.1915	2565.2294	-0.0379	0	40	0.00028	1	U			■							R.ELTVQATTGTNSTSDLDSIQDEIK.S
1824	1	856.0711	2565.1915	2565.1930	-0.0015	0	39	0.00037	2		■	■								R.ELTVQASTGTNSDSDLDSIQDEIK.S
1827	4	1283.6050	2565.1954	2565.1930	0.0025	0	145	9.1e-15	1		■	■								R.ELTVQASTGTNSDSDLDSIQDEIK.S
1827	4	1283.6050	2565.1954	2565.2294	-0.0339	0	58	4.6e-06	2	U			■							R.ELTVQATTGTNSTSDLDSIQDEIK.S
1837	1	877.0988	2628.2746	2628.2739	0.0007	0	81	4e-08	1		■	■	■	■	■					R.NANDGISVAQTTEGALSEINNNLQR.I
1838	1	1315.1460	2628.2774	2628.2739	0.0035	0	146	1.1e-14	1		■	■	■	■	■					R.NANDGISVAQTTEGALSEINNNLQR.I
1856	1	890.1072	2667.2998	2667.2988	0.0010	1	86	2.6e-09	1	U	■									K.TGADAGAATANAGVSFTDTASKETVLNK.V
1894		562.2688	2806.3076	2806.3832	-0.0756	1	0	1.7	1							■		■		R.VRELTVQATTGTNSDSDLSSIQDEIK.S
1912	1	936.7774	2807.3104	2808.3625	-1.0521	1	19	0.053	2	U			■							R.ELTVQATTGTNSTSDLDSIQDEIKSR.L
1913	1	937.1154	2808.3244	2808.3261	-0.0017	1	41	0.00025	1		■	■								R.ELTVQASTGTNSDSDLDSIQDEIKSR.L
1918	2	945.8010	2834.3812	2834.3781	0.0030	1	111	2.9e-11	1		■	■								R.IRELTVQASTGTNSDSDLDSIQDEIK.S
1918	2	945.8010	2834.3812	2834.4145	-0.0333	1	53	1.7e-05	2	U			■							R.IRELTVQATTGTNSTSDLDSIQDEIK.S

Query	Dupes	Observed	Mr(expt)	Mr(calc)	Delta	M	Score	Expect	Rank	U	1	2	3	4	5	6	7	8	9	Peptide
1919		1418.1990	2834.3834	2834.3781	0.0053	1	61	2.4e-06	▶1		■	■								R.IRELTVQASTGTNSDSDLDSIQDEIK.S
1919		1418.1990	2834.3834	2834.4145	-0.0311	1	27	0.0068	▶2	U			■							R.IRELTVQATTGTNSTSDLDSIQDEIK.S
1944	▶14	975.4984	2923.4734	2923.4662	0.0071	1	76	4.4e-08	▶1		■	■								K.VGAEVYVSANGTLTTDATSEGTVTKDPLK.A
1966		1031.8560	3092.5462	3092.5448	0.0014	1	102	2.5e-10	▶1		■		■	■	■	■				R.IQDADYATEVSNMSKAQIIQQAGNSVLAK.A
1976	▶5	1058.1520	3171.4342	3171.4381	-0.0039	0	119	1.3e-12	▶1	U	■									K.ADAAQTATVYTYNASAGNFSFSNVSNNTSAK.A
1983	▶2	1586.7290	3171.4434	3171.4381	0.0054	0	74	3.7e-08	▶1	U	■									K.ADAAQTATVYTYNASAGNFSFSNVSNNTSAK.A
1984		1059.2030	3174.5872	3174.5865	0.0007	1	86	6.4e-09	▶1	U								■		R.SSLGAVQNRLDSAITNLNNTTTNLSEAQSR.I
1984		1059.2030	3174.5872	3174.5865	0.0007	1	84	8.7e-09	▶2		■	■								R.SSLGAIQNRLDSAVTNLNNTTTNLSEAQSR.I
1990		1059.7950	3176.3632	3176.3616	0.0016	0	88	1.8e-09	▶1	U	■									K.YADNTDVSNATATYTDADGEMTTIGSYTTK.Y
1990		1059.7950	3176.3632	3176.3979	-0.0348	1	29	0.0013	▶2	U		■								K.YADKADVSNATATYTDADGEMTTIGSYTTK.Y + Oxidat
1992	▶4	1589.1890	3176.3634	3176.3616	0.0019	0	81	7.4e-09	▶1	U	■									K.YADNTDVSNATATYTDADGEMTTIGSYTTK.Y
1992		1589.1890	3176.3634	3176.3979	-0.0345	1	1	0.75	▶2	U		■								K.YADKADVSNATATYTDADGEMTTIGSYTTK.Y + Oxidat
1993	▶2	1065.1260	3192.3562	3192.3565	-0.0003	0	52	5.9e-06	▶1	U	■									K.YADNTDVSNATATYTDADGEMTTIGSYTTK.Y + Oxidat
1995		1597.1880	3192.3614	3192.3565	0.0050	0	31	0.00077	▶1	U	■									K.YADNTDVSNATATYTDADGEMTTIGSYTTK.Y + Oxidat
2013		1086.5730	3256.6972	3256.7011	-0.0039	1	117	7.5e-12	▶1		■		■							M.AQVINTNSLSLITQNNINKNQSALSSSIER.L
2014		815.1843	3256.7081	3256.7011	0.0070	1	34	0.0014	▶1		■		■							M.AQVINTNSLSLITQNNINKNQSALSSSIER.L
2052	▶2	1119.5550	3355.6432	3355.6420	0.0012	0	111	7.7e-12	▶1	U	■									K.ITIGGQEAYLTSDGNLTTNDAGGATAATLDGLFK.K
2052	▶2	1119.5550	3355.6432	3354.6943	0.9488	1	96	2.4e-10	▶2	U		■								K.ITIGGQKAYLTSDGNLTTNDAGGATAATLDGLFK.K
2056		1126.8820	3377.6242	3376.7256	0.8986	1	1	1.2	▶1	U									■	-.MAQVINTNSLSLLTQNNLNKSQSSLSSAIER.L + Oxida
2068		871.9421	3483.7393	3483.7369	0.0024	1	8	0.16	▶1	U	■									K.ITIGGQEAYLTSDGNLTTNDAGGATAATLDGLFKK.A
2069	▶1	1162.2540	3483.7402	3483.7369	0.0032	1	182	6.8e-19	▶1	U	■									K.ITIGGQEAYLTSDGNLTTNDAGGATAATLDGLFKK.A

▶ **61 subsets and intersections (158 subset proteins in total)**

---

10 per page 1

Not what you expected? Try **the select summary**.

Mascot: http://www.matrixscience.com/
