# Supplementary material for: MS-H: A Novel Proteomic Approach to Isolate and Type the E. coli H Antigen Using Membrane Filtration and Liquid Chromatography-Tandem Mass Spectrometry (LC-MS/MS)
Source: PLoS One. 2013 Feb 21;8(2):e57339. doi: 10.1371/journal.pone.0057339 (PMC3578835; doi:10.1371/journal.pone.0057339)
Supplement: Representative Peptide Data S1 — Peptide data are represented as the Mascot search results from all 53 serotypes, obtained under the Orbitrap platform in Table 4 with related E. coli reference strains. “U” denotes a unique peptide specific for each of the proteins 1.1, 1.2, and beyond. The number 1.1 (shown as 1 in the peptide list and phylogenetic tree) represents the protein which obtained the highest score and confidence value after a Mascot search. This protein, known as the first hit, was used to designate the MS-H type of the unknown flagellin. Related peptides 1.2 (2), 1.3 (3), etc. represented the second, third, etc. hits for MS-H typing analysis. (DOCX) [file pone.0057339.s009.docx › H2-E170.pdf]

MATRIX SCIENCE MASCOT Search Results

User :
E-mail :
Search title : Submitted from 20110809-0586 by Mascot Daemon on VARIABLE
MS data file : C:\Documents and Settings\keding\Desktop\Raw data\20110809-001-0031-00586\20110809-003-E170MS1_110810164058.RAW
Database : Flagellin_v2 (192 sequences; 89,845 residues)
Taxonomy : Bacteria (Eubacteria) (192 sequences)
Timestamp : 11 Aug 2011 at 17:12:48 GMT

Not what you expected? Try **the select summary**.

▸ **Search parameters**
▸ **Score distribution**
▸ **Legend**

Protein Family Summary

Significance threshold p< 0.05 Max. number of families AUTO
Ions score or expect cut-off 0 Dendrograms cut at 0

Protein families 1–2 (out of 2)

10 per page 1

▾**1**

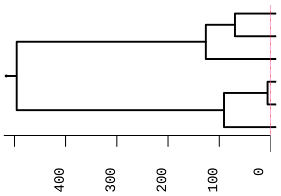

1	**gi|23477612**	1646	H2 0|O128:H2|0
3	**gi|33590243**	627	H16 NM|O157:NM(H16)|PC02
6	**gi|46093552**	411	H53 0|0|E480-68
2	**gi|33590235**	649	H12 0|O157:H12|90
5	**gi|6941882**	525	H14 0|O138:H14|O138:H14
4	**gi|192955539**	569	H9 0|O111:H9|E110019 [gi|193071144|ref|ZP_03052068| H9 0|O111:H9|E110019]

Threshold (**0**): 0

		Score	Mass	Matches	Sequences	emPAI
**1.1**	**gi|23477612**<br>H2 0|O128:H2|0	1646	51966	40 (35)	31 (29)	7.61
**1.2**	**gi|33590235**<br>H12 0|O157:H12|90<br>▸**3 samesets of gi|33590235**	649	61014	21 (16)	19 (16)	1.31
**1.3**	**gi|33590243**<br>H16 NM|O157:NM(H16)|PC02<br>▸**2 samesets of gi|33590243**	627	55093	24 (17)	18 (14)	1.68
**1.4**	**gi|192955539**<br>H9 0|O111:H9|E110019 [gi|193071144|ref|ZP_03052068| H9 0|O111:H9|E110019]<br>▸**2 samesets of gi|192955539**	569	68106	19 (13)	16 (12)	0.84
**1.5**	**gi|6941882**<br>H14 0|O138:H14|O138:H14	525	56492	25 (13)	23 (13)	1.09
**1.6**	**gi|46093552**<br>H53 0|0|E480-68	411	44861	15 (10)	13 (9)	1.03

▾**80 peptide matches (75 non-duplicate, 5 duplicate)**

Query	Dupes	Observed	Mr(expt)	Mr(calc)	Delta	M	Score	Expect	Rank	U	1	2	3	4	5	6	Peptide
22		315.7002	629.3858	629.3860	-0.0002	1	1	0.81	▸1	U			■				K.VDKLR.S
29	▸1	**316.6894**	**631.3642**	**631.3653**	**-0.0011**	**0**	**27**	**0.018**	▸1		■	■	■	■	■	■	**R.LSSGLR.I**
73		**347.2001**	**692.3856**	**692.3857**	**-0.0001**	**0**	**20**	**0.023**	▸1		■		■				**R.FTANIK.G**
93		355.1975	708.3804	708.3806	-0.0002	0	8	1	▸2			■		■	■	■	R.FTSNIK.G
98		358.7129	715.4112	715.4116	-0.0003	0	9	0.96	▸2	U						■	K.IDIDLK.K
99		358.7188	715.4230	715.3864	0.0366	0	5	2.1	▸1	U	■						K.LVDANGK.D
102		359.2073	716.4000	715.3977	1.0024	0	2	3.2	▸1			■		■	■		K.GLTQAAR.N
115		**366.6978**	**731.3810**	**731.3813**	**-0.0003**	**0**	**26**	**0.0082**	▸1		■		■				**R.LSEIDR.V**
132		**380.2032**	**758.3918**	**758.4174**	**-0.0255**	**0**	**35**	**0.0017**	▸1	**U**			■				**K.LDEALAK.V**
133	▸1	**380.6953**	**759.3760**	**759.3763**	**-0.0002**	**0**	**28**	**0.0095**	▸1			■		■	■		**R.LDEIDR.V**
193		403.6950	805.3754	804.3977	0.9777	0	4	0.38	▸1	U					■		K.ITASNGDK.L
213	▸1	409.2119	816.4092	816.4090	0.0003	0	8	0.2	▸1	U	■						K.GTTTPGQR.D
307	▸1	**431.2194**	**860.4242**	**860.4240**	**0.0003**	**0**	**34**	**0.00036**	▸1	**U**	■						**K.VELGGSDGK.T**
337		438.7375	875.4604	876.4552	-0.9948	0	8	0.17	▸1	U					■		K.AATTADSLK.A
354		444.2687	886.5228	886.4760	0.0469	0	6	0.27	▸1	U					■		K.AATTADPLK.A
415		466.3114	930.6082	930.4559	0.1523	0	0	4.4	▸1	U						■	K.DGAYHAAVK.N
416		466.7433	931.4720	930.4883	0.9838	0	3	2.2	▸1				■	■	■		R.SSLGAVQNR
440		**473.2589**	**944.5032**	**944.5039**	**-0.0007**	**0**	**59**	**3.9e-06**	▸1			■				■	**R.SSLGAIQNR**
513		**486.7645**	**971.5144**	**971.5148**	**-0.0004**	**0**	**52**	**7.4e-06**	▸1	**U**	■						**R.SNLGAIQNR.F**
530		**491.2350**	**980.4554**	**980.4563**	**-0.0009**	**0**	**22**	**0.0069**	▸1	**U**		■					**K.YSIDANNGK.V**
548		**494.7746**	**987.5346**	**987.5349**	**-0.0002**	**0**	**52**	**6.2e-06**	▸1	**U**	■						**K.ALSQVDSLR.S**

Query	Dupes	Observed	Mr(expt)	Mr(calc)	Delta	M	Score	Expect	Rank	U	1	2	3	4	5	6	Peptide
550		495.2666	988.5186	989.5029	-0.9843	0	19	0.012	1	U			■				K.GAELSASDLK.A
577		502.2396	1002.4646	1002.5094	-0.0448	1	5	0.58	1			■		■	■		K.SRLDEIDR.V
606		508.2621	1014.5096	1014.5709	-0.0613	0	11	0.088	1	U			■				K.ALATTNPLSK.L
616		510.7631	1019.5116	1019.5135	-0.0019	0	54	6.6e-06	1	U	■						K.TEVVTATDGK.T
619		511.2617	1020.5088	1020.5088	0.0001	0	75	4.6e-08	1	U		■					K.VTVDSGTGTGK.Y
712		531.2946	1060.5746	1060.5764	-0.0018	0	32	0.00069	1	U		■					K.AATISDLTAAK.M
713		532.2980	1062.5814	1061.4924	1.0891	0	9	0.27	1	U			■				K.NDGSAQAIMR.E
730		536.7795	1071.5444	1071.5448	-0.0003	0	45	3.5e-05	1	U	■						K.TEDPLAAIDK.A
740	1	539.2701	1076.5256	1077.4873	-0.9616	0	20	0.013	1	U			■				K.NDGSAQAIMR.E + Oxidation (M)
797		549.2757	1096.5368	1096.5401	-0.0032	0	61	7.3e-07	1	U	■						K.TYSVSDLQGK.S
811		551.2675	1100.5204	1100.5210	-0.0006	0	70	9.6e-07	1		■	■	■	■	■	■	K.DDAAGQAIANR.F
847		561.2478	1120.4810	1120.4819	-0.0008	0	59	1.3e-06	1	U	■						R.DGNIMSDANGK.L
872		567.8035	1133.5924	1133.5928	-0.0004	0	63	7.6e-07	1	U	■						K.TDSIAAISTQK.T
877		569.2463	1136.4780	1136.4768	0.0013	0	30	0.001	1	U	■						R.DGNIMSDANGK.L + Oxidation (M)
936		581.3033	1160.5920	1160.5925	-0.0004	0	69	1.6e-07	1			■		■	■		K.ALDEAISSIDK.F
942		582.7960	1163.5774	1163.5782	-0.0008	0	57	6.2e-06	1				■			■	K.SQSSLSSAIER.L
944		582.8038	1163.5930	1163.5935	-0.0005	0	46	8.1e-05	1		■					■	R.VSGQTQFNGVK.V
986		594.2659	1186.5172	1186.5176	-0.0004	0	64	4e-07	1	U	■						K.TMSYTDADGVK.H
995		596.3015	1190.5884	1190.5891	-0.0006	0	65	1.7e-06	1			■		■	■		K.NQSALSSSIER.L
1016		600.8536	1199.6926	1199.6734	0.0192	1	8	0.15	1	U			■				K.LRSSLGAVQNR.F
1200		648.3090	1294.6034	1294.6041	-0.0007	0	104	4.4e-11	1	U	■						K.DTGSFALIGDDGK.Q
1385		719.9036	1437.7926	1437.7940	-0.0013	0	64	4.7e-07	1	U	■						K.LGALAGTQPQAGNLK.E
1387		720.9125	1439.8104	1439.8096	0.0008	0	99	6.1e-10	1			■		■	■		K.AQIIQQAGNSVLAK.A
1394		723.8585	1445.7024	1445.7038	-0.0014	0	93	5e-10	1	U	■						K.AATAETTYFGSTVK.L
1440		747.3706	1492.7266	1491.7681	0.9585	0	1	3.2	1	U						■	K.VAANTSGLAANQTFK.S
1442		747.9182	1493.8218	1493.8202	0.0017	0	52	4.2e-05	1			■		■	■		K.ANQVPQQVLSLLQG.-
1518		781.4209	1560.8272	1560.8260	0.0012	0	67	1e-06	1			■			■		R.VSGQTQFNGVNVLAK.D
1573		538.9445	1613.8117	1613.8121	-0.0004	1	23	0.047	1		■	■	■	■	■	■	R.INSAKDDAAGQAIANR.F
1621		836.3806	1670.7466	1670.7457	0.0009	0	106	1.5e-10	1			■		■	■		R.IQDADYATEVSNMSK.A
1652		850.8750	1699.7354	1699.7359	-0.0005	0	140	1.5e-14	1		■		■				R.IEDADYATEVSNMSR.A
1672		858.8714	1715.7282	1715.7308	-0.0026	0	86	5.5e-09	1		■		■				R.IEDADYATEVSNMSR.A + Oxidation (M)
1677		860.3573	1718.7000	1718.7974	-0.0973	0	14	0.039	1	U			■				K.ALAYNDAPMSVYFGGK.N + Oxidation (M)
1702		579.6243	1735.8511	1735.8840	-0.0329	1	8	0.16	1	U					■		K.LTTDAETKAATTADSLK.A
1741		445.9593	1779.8081	1779.8098	-0.0017	1	19	0.014	1	U	■						K.TMSYTDADGVKHDNVK.V
1742		594.2770	1779.8092	1779.8098	-0.0006	1	28	0.0017	1	U	■						K.TMSYTDADGVKHDNVK.V
1759		896.4789	1790.9432	1790.9414	0.0018	0	106	1.9e-10	1	U	■						K.LQEINSDTLGLSGFGIK.D
1771		599.6516	1795.9330	1795.8873	0.0456	1	2	0.61	1	U					■		K.LTTDAETKAATTADMLK.A + Oxidation (M)
1835		621.6495	1861.9267	1861.9269	-0.0003	1	55	3.4e-06	1	U	■						K.VELGGSDGKTEVVTATDGK.T
1912		648.6299	1942.8679	1942.8690	-0.0012	1	48	2.4e-05	1		■		■				R.SRIEDADYATEVSNMSR.A
1913		972.4420	1942.8694	1942.8690	0.0004	1	76	4e-08	1		■		■				R.SRIEDADYATEVSNMSR.A
1950		665.3366	1992.9880	1992.9865	0.0015	0	58	3.8e-06	1		■		■			■	R.FDSAITNLGNTVNNLSSAR.S
1951		997.5016	1992.9886	1992.9865	0.0022	0	123	1.1e-12	1		■		■			■	R.FDSAITNLGNTVNNLSSAR.S
1981		679.3546	2035.0420	2035.0143	0.0277	1	19	0.014	1	U					■		K.AATTADCLKALDEAISSIDK.F
1994		695.7155	2084.1247	2084.1225	0.0021	0	56	1.7e-05	1		■	■	■	■	■	■	M.AQVINTNSLSLiTQNNiNK.N
2062		745.0631	2232.1675	2232.1638	0.0037	1	78	1.2e-07	1	U	■						K.LQEINSDTLGLSGFGIKDPTK.L
2133		1245.6460	2489.2774	2489.2762	0.0013	0	104	4.1e-11	1	U				■			K.ASDLLANITDGSVITGGGANAFGVAAK.N
2134		830.7672	2489.2798	2489.2762	0.0036	0	20	0.011	1	U				■			K.ASDLLANITDGSVITGGGANAFGVAAK.N
2158		1328.6520	2655.2894	2655.2848	0.0046	0	130	2.4e-13	1	U	■						R.NANDGISVAQTTEGALNEINNNLQR.I
2159		886.1039	2655.2899	2655.2848	0.0051	0	69	3.1e-07	1	U	■						R.NANDGISVAQTTEGALNEINNNLQR.I
2167		893.4659	2677.3759	2677.3043	0.0716	1	4	0.42	1	U					■		K.GTITIDGSAQDVQISSDGKITASNGDK.L
2194		956.1985	2865.5737	2865.5672	0.0065	0	57	1.9e-06	1	U						■	R.AQILQQAGTSVLAQANQTTQNVLSLLR.-
2197		971.1091	2910.3055	2910.3043	0.0012	0	54	4.6e-06	1	U	■						K.VAGSDKPAENGYYEVTVEDDPTSPDAGK.L
2238		1086.5760	3256.7062	3256.7011	0.0051	1	78	6.4e-08	1			■		■	■		M.AQVINTNSLSLITQNNINKNQSALSSSIER.L
2238		1086.5760	3256.7062	3256.7011	0.0051	1	66	1.1e-06	3	U	■						M.AQVINTNSLSLLTQNNLNKSQSSLNSAIER.L

51 subsets and intersections (159 subset proteins in total)

2	gi|307553085	16	Hxx(H54 27.9%) 0|0|ABU 83972

10 per page 1

Not what you expected? Try **the select summary**.

Mascot: http://www.matrixscience.com/
