# Supplementary material for: MS-H: A Novel Proteomic Approach to Isolate and Type the E. coli H Antigen Using Membrane Filtration and Liquid Chromatography-Tandem Mass Spectrometry (LC-MS/MS)
Source: PLoS One. 2013 Feb 21;8(2):e57339. doi: 10.1371/journal.pone.0057339 (PMC3578835; doi:10.1371/journal.pone.0057339)
Supplement: Representative Peptide Data S1 — Peptide data are represented as the Mascot search results from all 53 serotypes, obtained under the Orbitrap platform in Table 4 with related E. coli reference strains. “U” denotes a unique peptide specific for each of the proteins 1.1, 1.2, and beyond. The number 1.1 (shown as 1 in the peptide list and phylogenetic tree) represents the protein which obtained the highest score and confidence value after a Mascot search. This protein, known as the first hit, was used to designate the MS-H type of the unknown flagellin. Related peptides 1.2 (2), 1.3 (3), etc. represented the second, third, etc. hits for MS-H typing analysis. (DOCX) [file pone.0057339.s009.docx › H3-E171.pdf]

MASCOT Search Results

User :
E-mail :
Search title : Submitted from 20110714-H1-H11 by Mascot Daemon on VARIABLE
MS data file : C:\Documents and Settings\keding\Desktop\Raw data\20110714-H1-H11\20110714-006-E171MS1.RAW
Database : Flagellin_v2 (192 sequences; 89,845 residues)
Taxonomy : Bacteria (Eubacteria) (192 sequences)
Timestamp : 15 Jul 2011 at 17:26:36 GMT

Not what you expected? Try **the select summary**.

Search parameters

Score distribution

Legend

Protein Family Summary

Significance threshold p< 0.05 Max. number of families AUTO
Ions score or expect cut-off 0 Dendrograms cut at 0

Protein families 1–3 (out of 3)

10 per page 1

1

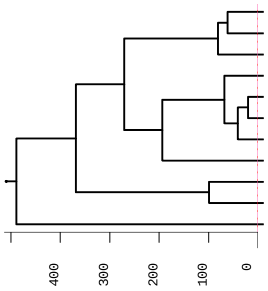

1	gi|46093545	7713	H3 0|0|Bi7327-41
2	gi|154710444	3331	H35 0|H35|0
10	gi|121144335	642	H55 0|O75:H55|E2987-73
3	gi|192925955	2148	H2 0|O103:H2|E22 [gi|193066370|ref|ZP_03047418| H2 0|O103:H2|E22] [gi|260844327|ref|YP...
8	gi|59044663	1183	H11 0|O78:H11|ATCC 35401 [gi|59044673|ref|AAW84030| H11 0|O26:H11|88-353] [gi|590446...
9	gi|40353143	1153	H2 0|O111:H2|5721/96
11	gi|62532942	90	H11 0|O26:H11|0
4	gi|154710451	1844	H36 0|H36|0
7	gi|290437	1238	H1 0|0|Su 1242 (Orskov)
6	gi|145997	1452	Hxx(H12 99.7%) 0|0|Bi 316-42 (Orskov)
5	gi|30059872	1779	H16 0|H16|F 8316-41

Threshold (**0**): 0

		Score	Mass	Matches	Sequences	emPAI
1.1	**gi|46093545**<br>H3 0|0|Bi7327-41	7713	55534	181 (157)	51 (46)	55.47
1.2	**gi|154710444**<br>H35 0|H35|0	3331	52714	80 (65)	18 (15)	3.55
1.3	**gi|192925955**<br>H2 0|O103:H2|E22 [gi|193066370|ref|ZP_03047418| H2 0|O103:H2|E22] [gi|260844327|ref|YP_003222105| H2 0|O103:H2|12009] [gi|257759474|ref|BAI30971| H2 0|O103:H2|12009] [gi|3231585...	2148	51996	65 (46)	22 (16)	2.64
1.4	**gi|154710451**<br>H36 0|H36|0	1844	57784	59 (34)	16 (11)	1.29
1.5	**gi|30059872**<br>H16 0|H16|F 8316-41	1779	52639	63 (44)	21 (15)	1.98
1.6	**gi|145997**<br>Hxx(H12 99.7%) 0|0|Bi 316-42 (Orskov)	1452	61008	38 (25)	14 (9)	0.88
1.7	**gi|290437**<br>H1 0|0|Su 1242 (Orskov)<br>**1 sameset of gi|290437**	1238	60887	30 (21)	13 (9)	0.78
1.8	**gi|59044663**<br>H11 0|O78:H11|ATCC 35401 [gi|59044673|ref|AAW84030| H11 0|O26:H11|88-353] [gi|59044679|ref|AAW84033| H11 0|O26:H11|DEC9E] [gi|59044695|ref|AAW84041| H11 0|O111:H11|88-4110] [g...<br>**4 samesets of gi|59044663**	1183	46411	38 (30)	9 (7)	0.99
1.9	**gi|40353143**<br>H2 0|O111:H2|5721/96	1153	47290	39 (30)	10 (7)	0.96
1.10	**gi|121144335**<br>H55 0|O75:H55|E2987-73	642	62285	27 (16)	12 (6)	0.43
1.11	**gi|62532942**<br>H11 0|O26:H11|0	90	37909	7 (4)	3 (2)	0.18

271 peptide matches (134 non-duplicate, 137 duplicate)

Query	Dupes	Observed	Mr(expt)	Mr(calc)	Delta	M	Score	Expect	Rank	U	1	2	3	4	5	6	7	8	9	10	11	Peptide
13		305.1660	608.3174	609.2758	-0.9584	0	3	0.54	1	U										■		K.AYNDK.Y
18	1	308.6865	615.3584	615.3591	-0.0007	0	20	0.02	1	U	■											K.NLEIK.Q
34	2	315.7004	629.3862	629.3860	0.0002	1	27	0.0019	1		■				■							K.VDKLR.S
39	3	316.6897	631.3648	631.3653	-0.0005	0	29	0.012	1		■	■	■	■	■	■	■	■	■	■		R.LSSGLR.I
53		324.6821	647.3496	647.3490	0.0006	0	33	0.00047	1	U	■											K.VTVDSK.A
61		330.7004	659.3862	660.3806	-0.9944	0	0	0.95	3	U			■									K.TGAVSVK.T
96		347.1998	692.3850	692.3857	-0.0007	0	12	0.15	1				■		■			■	■			R.FTANIK.G
113		352.6826	703.3506	703.3501	0.0006	0	17	0.032	1	U	■											K.AVNTGDK.T
120	1	355.1973	708.3800	708.3806	-0.0006	0	17	0.14	1		■	■		■		■	■			■		R.FTSNIK.G
126		358.7135	715.4124	715.3977	0.0148	0	4	0.59	1							■	■			■		K.GLTQAAR.N

Query	Dupes	Observed	Mr(expt)	Mr(calc)	Delta	M	Score	Expect	Rank	U	1	2	3	4	5	6	7	8	9	10	11	Peptide
130		359.2112	716.4078	715.4228	0.9850	0	9	0.99	1	U						■						K.ITIGGQK.A
134	1	359.6958	717.3770	717.3769	0.0001	0	32	0.00073	1	U	■											K.GLSQASR.N
141	2	366.2183	730.4220	730.4225	-0.0004	0	58	6.2e-06	1	U	■											K.LDTALAK.V
143	2	366.6975	731.3804	731.3813	-0.0009	0	33	0.0019	1		■		■		■							R.LSEIDR.V
144	2	366.7204	731.4262	731.4177	0.0085	0	10	0.35	3	U				■								K.ISATNVK.I
226		409.7238	817.4330	816.4090	1.0241	0	5	0.47	2	U			■									K.GTTTPGQR.D
235	2	412.7112	823.4078	823.4076	0.0003	0	40	9.7e-05	1	U	■											K.DFSVASAK.V
309		428.1958	854.3770	854.3770	0.0000	0	44	4.2e-05	1	U	■											K.QSGSGYEK.V
404		458.7768	915.5390	915.5138	0.0253	1	3	0.5	2	U	■											K.DGAAKTVVR.K
423	5	466.2504	930.4862	930.4883	-0.0020	0	75	1.3e-07	1		■	■			■					■		R.SSLGAVQNR
447	2	471.7842	941.5538	941.5546	-0.0007	0	53	5e-06	1	U	■											K.VPTSGAVALK.S
456	1	473.2661	944.5176	944.5179	-0.0002	0	69	4.2e-07	1	U	■											K.IDADTLGLK.D
502		486.7943	971.5740	971.5148	0.0592	0	34	0.00043	1	U			■									R.SNLGAIQNR.F
502		486.7943	971.5740	972.4988	-0.9248	0	21	0.0087	2	U				■								R.SDLGAIQNR.F
505		488.2827	974.5508	973.5444	1.0065	1	4	0.38	1	U				■								K.ISAEDLKAK.A
525		494.7742	987.5338	987.5349	-0.0010	0	21	0.0071	1	U			■									K.ALSQVDSLR.S
529		496.7570	991.4994	991.5008	-0.0014	0	30	0.00092	1	U					■							K.VLASDQTMK.I
551	4	503.2719	1004.5292	1004.5325	-0.0032	0	39	0.00012	1	U	■											K.VLATNQTMK.I
551	3	503.2719	1004.5292	1004.5325	-0.0032	0	28	0.0016	2	U		■										K.VLASQQTMK.I
581	3	511.2701	1020.5256	1020.5274	-0.0017	0	67	3e-07	1	U	■											K.VLATNQTMK.I + Oxidation (M)
581	3	511.2701	1020.5256	1020.5274	-0.0017	0	50	1.5e-05	2	U		■										K.VLASQQTMK.I + Oxidation (M)
598	1	516.7830	1031.5514	1031.5512	0.0002	0	62	5.7e-07	1	U	■											K.AVQIANFGGR.V
640		529.3028	1056.5910	1056.5927	-0.0017	0	33	0.00048	1	U									■			-.LLTQNNLNK.S
660		535.7946	1069.5746	1069.5768	-0.0021	0	56	2.7e-06	1	U	■											K.QVLDADGKPK.A
661		357.5328	1069.5766	1069.5768	-0.0002	0	46	2.5e-05	1	U	■											K.QVLDADGKPK.A
665		537.2470	1072.4794	1071.5448	0.9347	0	16	0.023	1	U			■									K.TEDPLAAIDK.A
668	1	538.2927	1074.5708	1074.5743	-0.0035	0	35	0.0005	1	U	■											K.GMTTAKPLEK.L
671	1	359.1982	1074.5728	1074.5743	-0.0015	0	23	0.0077	1	U	■											K.GMTTAKPLEK.L
671		359.1982	1074.5728	1074.5306	0.0422	0	1	1.2	2	U					■							K.VNATDGSVGGAK.A
672		539.2694	1076.5242	1077.4873	-0.9630	0	13	0.068	1	U					■							K.NDGSAQAIMR.E + Oxidation (M)
713	1	364.5294	1090.5664	1090.5692	-0.0029	0	32	0.00065	1	U	■											K.GMTTAKPLEK.L + Oxidation (M)
715	3	546.2908	1090.5670	1090.5692	-0.0022	0	41	8.5e-05	1	U	■											K.GMTTAKPLEK.L + Oxidation (M)
734	3	551.2663	1100.5180	1100.5210	-0.0030	0	76	2.2e-07	1		■	■	■	■	■	■	■	■	■	■		K.DDAAGQAIANR.F
741	2	554.2733	1106.5320	1106.5356	-0.0036	0	104	4.1e-11	1	U	■											K.AGAFAAQTADGK.S
763	1	559.8271	1117.6396	1118.5641	-0.9245	1	10	0.099	1	U				■								K.TTDPMAKLDK.A
849		582.2975	1162.5804	1163.5935	-1.0131	0	2	2	1				■	■								R.VSGQTQFNGVK.V
852	8	582.7953	1163.5760	1163.5782	-0.0022	0	61	2.3e-06	1		■	■	■	■	■			■	■			K.SQSSLSSAIER.L
887		594.8035	1187.5924	1187.6034	-0.0109	0	3	0.5	1	U										■		K.ALDDAISQIDK.F
887		594.8035	1187.5924	1186.5176	1.0748	0	3	0.5	2	U			■									K.TMSYTDADGVK.H
900		599.3116	1196.6086	1196.5310	0.0777	0	9	0.13	1	U									■			K.ATGTDNYDVGGK.T
908		600.8531	1199.6916	1199.6734	0.0182	1	11	0.084	1		■				■							K.LRSSLGAVQNR.F
934	1	608.7779	1215.5412	1215.5408	0.0004	0	75	3e-08	1	U	■											R.TDDVNTYFGGK.A
934		608.7779	1215.5412	1216.6663	-1.1250	1	8	0.17	2	U		■										K.EINSKTLGLDK.L
937		406.5465	1216.6177	1216.6663	-0.0486	1	1	0.88	1	U		■										K.EINSKTLGLDK.L
944		611.3141	1220.6136	1220.6150	-0.0013	0	45	3.3e-05	1			■			■							R.VSNQTQFNGVK.V
962	2	618.3216	1234.6286	1234.6306	-0.0020	0	53	3e-05	1	U	■											R.VSQQTQFNGVK.V
962	3	618.3216	1234.6286	1235.6146	-0.9860	0	34	0.0025	2									■	■		■	R.VSEQTQFNGVK.V
1082		651.8625	1301.7104	1301.6827	0.0278	0	7	0.48	1									■	■		■	K.AATLSDLDLNAAK.K
1113	2	659.8126	1317.6106	1317.6102	0.0004	0	55	3.2e-06	1	U	■											K.NGTNYAFGATFR.T
1114		660.3041	1318.5936	1317.6929	0.9008	0	7	0.18	2	U					■							K.TLGLDNFSVAPGK.V
1147		672.8777	1343.7408	1343.7408	0.0000	0	81	7.8e-09	1	U										■		-.SLSLITQNNINK.N
1165		683.3223	1364.6300	1364.6783	-0.0483	0	2	0.7	1	U										■		K.GSVSNTAATTDTLK.L
1166	1	683.3361	1364.6576	1364.6572	0.0004	0	54	3.9e-06	1	U											■	R.FDSAITNLGNTVN.-
1199		464.9151	1391.7235	1390.7303	0.9931	1	5	0.34	1	U					■							K.TATKGAELSASDLK.A
1255		719.9032	1437.7918	1437.7940	-0.0021	0	41	8.9e-05	1	U			■									K.LGALAGTQPQAGNLK.E
1256		721.5192	1441.0238	1439.8096	1.2142	0	2	3	1							■	■			■		K.AQIIQQAGNSVLAK.A
1277		729.8991	1457.7836	1457.7838	-0.0001	0	64	4.2e-07	1	U								■				-.NSLSLLTQNNLNK.S
1280		730.8362	1459.6578	1459.6579	-0.0001	0	96	2.4e-10	1	U	■											K.DADGNNVEWVVDK.D
1320		747.3467	1492.6788	1492.6794	-0.0006	0	77	1.2e-07	1							■	■					K.AASGEVNFDVDANGK.I
1432	1	794.9099	1587.8052	1586.8264	0.9788	0	88	1.4e-09	1	U	■											K.SLAVNIDGNGNTSVVK.D
1454	1	807.9118	1613.8090	1613.8121	-0.0031	1	97	1.6e-09	1		■	■	■	■	■	■	■	■	■	■		R.INSAKDDAAGQAIANR.F
1455	1	538.9446	1613.8120	1613.8121	-0.0001	1	43	0.00044	1		■	■	■	■	■	■	■	■	■	■		R.INSAKDDAAGQAIANR.F
1466		812.3978	1622.7810	1622.7822	-0.0011	0	116	2.8e-12	1	U	■											K.SEMSPTLTSVNATTGK.N
1472		816.4277	1630.8408	1630.8414	-0.0005	0	15	0.028	1	U			■									K.LADANTLDADITATVK.G
1484		820.3951	1638.7756	1638.7771	-0.0014	0	100	1.1e-10	1	U	■											K.SEMSPTLTSVNATTGK.N + Oxidation (M)
1485		547.2899	1638.8479	1638.7771	0.0708	0	7	0.18	1	U	■											K.SEMSPTLTSVNATTGK.N + Oxidation (M)
1485	1	547.2899	1638.8479	1637.8308	1.0171	1	6	0.26	2	U		■										K.DVHVGGGRVMAANDIK.G
1528		840.4583	1678.9020	1678.9730	-0.0709	1	1	0.82	1	U			■									K.LKLGALAGTQPQAGNLK.E
1533		843.4581	1684.9016	1684.8996	0.0021	0	79	4.7e-08	1							■	■					K.IQVGANDGQTITIDLK.K
1533		843.4581	1684.9016	1685.8836	-0.9819	0	31	0.0032	2			■								■		K.IQVGANDGETITIDLK
1550		850.8760	1699.7374	1699.7359	0.0015	0	117	3.5e-12	1				■	■	■							R.IEDADYATEVSNMSR.A
1567	2	858.8721	1715.7296	1715.7308	-0.0012	0	144	8.9e-15	1		■	■										R.IEDSDYATEVSNMSR.A
1568	2	858.8727	1715.7308	1715.7308	0.0000	0	12	0.12	2				■	■	■							R.IEDADYATEVSNMSR.A + Oxidation (M)
1594	2	866.8663	1731.7180	1731.7257	-0.0077	0	140	1.1e-14	1		■	■										R.IEDSDYATEVSNMSR.A + Oxidation (M)
1615		584.3115	1749.9127	1749.9149	-0.0022	1	9	0.13	1	U	■											K.IDADTLGLKDFSVASAK.V
1632		880.9120	1759.8094	1759.8087	0.0007	0	87	2.1e-09	1	U	■											K.ALSFNDSQMSVYVDGK.N
1641	11	885.9641	1769.9136	1769.9159	-0.0023	0	131	7.2e-14	1	U	■											K.IQVGANDGQTIEIGLDK.I
1652		888.9088	1775.8030	1775.8036	-0.0006	0	95	1.5e-09	1	U	■											K.ALSFNDSQMSVYVDGK.N + Oxidation (M)
1668		596.6694	1786.9864	1786.9862	0.0001	1	40	0.00013	1	U	■											K.GMTTAKPLEKLDTALAK.V
1692	1	902.4525	1802.8904	1803.9438	-1.0534	1	1	3.9	1							■	■					K.NQSALSSSIERLSSGLR.I
1781		634.9656	1901.8750	1901.8755	-0.0006	1	45	3e-05	1	U	■											K.DADGNNVEWVVDKDGAAK.T
1782		951.9450	1901.8754	1901.8755	-0.0001	1	69	1.1e-07	1	U	■											K.DADGNNVEWVVDKDGAAK.T
1804		964.9709	1927.9272	1927.9276	-0.0003	0	77	3.6e-08	1							■	■					K.SGVQTYQAVFAAGDGTASAK.Y

Query	Dupes	Observed	Mr(expt)	Mr(calc)	Delta	M	Score	Expect	Rank	U	1	2	3	4	5	6	7	8	9	10	11	Peptide
1816		648.6302	1942.8688	1942.8690	-0.0003	1	41	0.00014	1				■	■	■							R.SRIEDADYATEVSNMSR.A
1819		975.0078	1948.0010	1948.0014	-0.0004	1	66	2.3e-07	1	U	■											R.LSEIDRVSQQTQFNGVK.V
1820		650.3417	1948.0033	1948.0014	0.0019	1	39	0.00013	1	U	■											R.LSEIDRVSQQTQFNGVK.V
1828	2	653.9618	1958.8636	1958.8640	-0.0004	1	67	4.2e-07	1		■	■										R.SRIEDSDYATEVSNMSR.A
1829	1	980.4398	1958.8650	1958.8640	0.0011	1	75	7.5e-08	1		■	■										R.SRIEDSDYATEVSNMSR.A
1829	1	980.4398	1958.8650	1958.8640	0.0011	1	12	0.16	2				■	■	■							R.SRIEDADYATEVSNMSR.A + Oxidation (M)
1831		654.2902	1959.8488	1958.8640	0.9848	1	4	0.84	2				■	■	■							R.SRIEDADYATEVSNMSR.A + Oxidation (M)
1845		988.4351	1974.8556	1974.8589	-0.0032	1	74	4.1e-08	1		■	■										R.SRIEDSDYATEVSNMSR.A + Oxidation (M)
1846	1	659.2928	1974.8566	1974.8589	-0.0023	1	60	1.1e-06	1		■	■										R.SRIEDSDYATEVSNMSR.A + Oxidation (M)
1851		992.4665	1982.9184	1982.9181	0.0003	0	113	6.6e-12	1	U							■					K.TGADAGAATANAGVSFTDTASK.E
1864	1	665.3361	1992.9865	1992.9865	0.0000	0	84	1.1e-08	1		■	■	■		■			■	■			R.FDSAITNLGNTVNNLSSAR.S
1866	5	997.5010	1992.9874	1992.9865	0.0010	0	141	1.9e-14	1		■	■	■		■			■	■			R.FDSAITNLGNTVNNLSSAR.S
1870	1	499.4689	1993.8465	1992.9865	0.8600	0	23	0.014	1		■	■	■		■			■	■			R.FDSAITNLGNTVNNLSSAR.S
1926	3	1043.0680	2084.1214	2084.1225	-0.0011	0	142	4.6e-14	1		■	■	■	■		■	■					M.AQVINTNSLSLiTQNNiNK.N
1929	2	695.7153	2084.1241	2084.1225	0.0015	0	81	4.9e-08	1		■	■	■	■		■	■					M.AQVINTNSLSLiTQNNiNK.N
1974	1	724.7028	2171.0866	2171.0859	0.0007	0	65	3.3e-07	1	U	■											R.VTAFVDDGTAAHNALSVDLQK.G
1975		1086.5520	2171.0894	2171.0859	0.0036	0	136	2.3e-14	1	U	■											R.VTAFVDDGTAAHNALSVDLQK.G
1984		734.6638	2200.9696	2202.0627	-1.0931	1	3	0.55	1	U	■											K.GTVGKALSFNDSQMSVYVDGK.N
1992		738.3937	2212.1593	2212.1587	0.0006	1	24	0.0044	1	U					■							K.IQVGANDGETIEIALDKIDAK.T
2046	1	1199.1420	2396.2694	2396.2659	0.0035	0	123	4.9e-13	1	U					■							R.AQILQQAGTSVLAQANQTTQNVL.-
2067		849.7823	2546.3251	2546.3262	-0.0011	1	73	5.3e-08	1	U	■											K.VPTSGAVALKSEMSPTLTSVNATTGK.N
2071		1283.6050	2565.1954	2565.1930	0.0025	0	91	2.3e-09	1							■	■					R.ELTVQASTGTNSDSDLDSIQDEIK.S
2089	3	1322.1560	2642.2974	2642.2896	0.0079	0	150	1.6e-15	1	U	■			■								R.NANDGISiAQTTEGALSEINNNLQR.V
2089	3	1322.1560	2642.2974	2642.2896	0.0079	0	130	1.9e-13	3	U						■						R.NANDAISVAQTTEGALSEINNNLQR.I
2090	2	881.7731	2642.2975	2642.2896	0.0079	0	84	7.1e-09	1	U	■			■								R.NANDGISiAQTTEGALSEINNNLQR.V
2090	2	881.7731	2642.2975	2642.2896	0.0079	0	80	1.8e-08	3	U						■						R.NANDAISVAQTTEGALSEINNNLQR.I
2091		661.5819	2642.2985	2642.2896	0.0089	0	6	0.41	1	U	■			■								R.NANDGISiAQTTEGALSEINNNLQR.V
2091		661.5819	2642.2985	2642.2896	0.0089	0	5	0.59	3	U						■						R.NANDAISVAQTTEGALSEINNNLQR.I
2122		675.1135	2696.4249	2696.4232	0.0017	1	32	0.00063	1	U	■											K.IQVGANDGQTIEIGLDKIDADTLGLK.D
2124	1	1349.2200	2696.4254	2696.4232	0.0022	1	112	7e-12	1	U	■											K.IQVGANDGQTIEIGLDKIDADTLGLK.D
2127	5	899.8173	2696.4301	2696.4232	0.0068	1	81	7.8e-09	1	U	■											K.IQVGANDGQTIEIGLDKIDADTLGLK.D
2144		1392.7010	2783.3874	2783.3866	0.0009	0	104	7.6e-11	1	U	■											K.IYGASVTGFGGTPTVNVDTTAIDASELK.G
2145		928.8035	2783.3887	2783.3866	0.0021	0	60	1.8e-06	1	U	■											K.IYGASVTGFGGTPTVNVDTTAIDASELK.G
2158	1	1416.6830	2831.3514	2831.3533	-0.0019	0	121	8e-13	1	U	■											R.ELAVQATNGTNSQSDLNSIQDEITQR.L
2159		944.7913	2831.3521	2831.3533	-0.0012	0	108	1.6e-11	1	U	■											R.ELAVQATNGTNSQSDLNSIQDEITQR.L
2166		1433.7950	2865.5754	2865.5672	0.0082	0	138	1.6e-14	1		■	■	■	■								R.AQILQQAGTSVLAQANQTTQNVLSLLR.-
2167		717.4012	2865.5757	2865.5672	0.0085	0	6	0.22	1		■	■	■	■								R.AQILQQAGTSVLAQANQTTQNVLSLLR.-
2168	1	956.1998	2865.5776	2865.5672	0.0104	0	71	8.4e-08	1		■	■	■	■								R.AQILQQAGTSVLAQANQTTQNVLSLLR.-
2179		969.4960	2905.4662	2905.4642	0.0020	1	16	0.025	1		■	■			■							R.SSLGAVQNRFDSAITNLGNTVNNLSSAR.S
2191	1	1029.8490	3086.5252	3086.5228	0.0023	1	138	1.6e-14	1	U	■											R.VRELAVQATNGTNSQSDLNSIQDEITQR.L
2198		1077.5730	3229.6972	3229.6902	0.0070	1	81	1e-08	1		■	■	■	■								M.AQVINTNSLSLLTQNNLNKSQSSLSSAIER.L

45 subsets and intersections (157 subset proteins in total)

2	gi|112820172	61	H21 0|EHEC serogroup: O113:H21|0
3	gi|307553085	22	Hxx(H54 27.9%) 0|0|ABU 83972

10 per page 1

Not what you expected? Try **the select summary**.

Mascot: http://www.matrixscience.com/
