# Supplementary material for: MS-H: A Novel Proteomic Approach to Isolate and Type the E. coli H Antigen Using Membrane Filtration and Liquid Chromatography-Tandem Mass Spectrometry (LC-MS/MS)
Source: PLoS One. 2013 Feb 21;8(2):e57339. doi: 10.1371/journal.pone.0057339 (PMC3578835; doi:10.1371/journal.pone.0057339)
Supplement: Representative Peptide Data S1 — Peptide data are represented as the Mascot search results from all 53 serotypes, obtained under the Orbitrap platform in Table 4 with related E. coli reference strains. “U” denotes a unique peptide specific for each of the proteins 1.1, 1.2, and beyond. The number 1.1 (shown as 1 in the peptide list and phylogenetic tree) represents the protein which obtained the highest score and confidence value after a Mascot search. This protein, known as the first hit, was used to designate the MS-H type of the unknown flagellin. Related peptides 1.2 (2), 1.3 (3), etc. represented the second, third, etc. hits for MS-H typing analysis. (DOCX) [file pone.0057339.s009.docx › H4-E172.pdf]

MASCOT Search Results

User :
E-mail :
Search title : Submitted from 20110714-H1-H11 by Mascot Daemon on VARIABLE
MS data file : C:\Documents and Settings\keding\Desktop\Raw data\20110714-H1-H11\20110714-007-E172MS1.RAW
Database : Flagellin_v2 (192 sequences; 89,845 residues)
Taxonomy : Bacteria (Eubacteria) (192 sequences)
Timestamp : 15 Jul 2011 at 17:29:43 GMT

Not what you expected? Try **the select summary**.

Search parameters
Score distribution
Legend

Protein Family Summary

Significance threshold p< 0.05 Max. number of families AUTO
Ions score or expect cut-off 0 Dendrograms cut at 0

Protein family 1 (out of 1)

10 per page 1

1

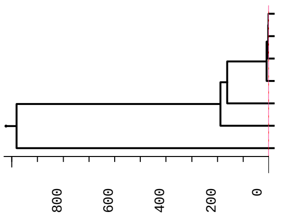

1	gi|40994711	3567	H4 0|O154:H4|0
4	gi|192955539	1446	H9 0|O111:H9|E110019 [gi|193071144|ref|ZP_03052068| H9 0|O111:H9|E110019]
5	gi|6941882	1213	H14 0|O138:H14|O138:H14
7	gi|88683097	915	H44 0|H44|0
6	gi|121144335	967	H55 0|O75:H55|E2987-73
3	gi|323174561	1712	Hxx(H25 99.7%) 0|0|LT-68
2	gi|46093545	1730	H3 0|0|Bi7327-41

Threshold (**0**): 0

		Score	Mass	Matches	Sequences	emPAI
1.1	gi|40994711<br>H4 0|O154:H4|0	3567	36224	81 (63)	28 (27)	22.44
1.2	gi|46093545<br>H3 0|0|Bi7327-41	1730	55534	56 (41)	31 (26)	5.70
1.3	gi|323174561<br>Hxx(H25 99.7%) 0|0|LT-68<br>1 sameset of gi|323174561	1712	46392	51 (34)	19 (17)	3.53
1.4	gi|192955539<br>H9 0|O111:H9|E110019 [gi|193071144|ref|ZP_03052068| H9 0|O111:H9|E110019]<br>2 samesets of gi|192955539	1446	68106	44 (29)	17 (15)	1.56
1.5	gi|6941882<br>H14 0|O138:H14|O138:H14	1213	56492	41 (25)	16 (13)	1.77
1.6	gi|121144335<br>H55 0|O75:H55|E2987-73	967	62285	36 (21)	14 (11)	1.16
1.7	gi|88683097<br>H44 0|H44|0	915	55289	35 (21)	13 (12)	1.52

136 peptide matches (79 non-duplicate, 57 duplicate)

Query	Dupes	Observed	Mr(expt)	Mr(calc)	Delta	M	Score	Expect	Rank	U	1	2	3	4	5	6	7	Peptide
7		302.1714	602.3282	601.3911	0.9371	1	6	0.33	1	U		■						K.TVVRK.D
26	1	308.7056	615.3966	615.3591	0.0375	0	3	1	1	U		■						K.NLEIK.Q
42	3	316.6903	631.3660	631.3653	0.0007	0	23	0.053	1		■	■	■	■	■	■	■	R.LSSGLR.I
72	2	332.6928	663.3710	663.3704	0.0006	1	17	0.019	1	U	■							K.VDKFR.S
82		337.7081	673.4016	673.3759	0.0258	0	5	0.3	1	U			■					K.NGATALK.L
134	1	355.1979	708.3812	708.3806	0.0006	0	20	0.067	1		■	■	■	■	■	■	■	R.FTSNIK.G
139	3	358.7060	715.3974	715.3977	-0.0002	0	31	0.0061	1		■		■	■	■	■	■	K.GLTQAAR.N
146	1	359.6963	717.3780	717.3769	0.0011	0	21	0.0082	1	U		■						K.GLSQASR.N
152	1	366.2191	730.4236	730.4225	0.0012	0	30	0.0035	1	U		■						K.LDTALAK.V
155		366.6981	731.3816	731.3813	0.0003	0	20	0.038	1	U		■						R.LSEIDR.V
178	4	380.6953	759.3760	759.3763	-0.0002	0	30	0.0057	1		■		■	■	■	■	■	R.LDEIDR.V
192	2	386.7319	771.4492	771.4490	0.0002	0	67	2.2e-07	1	U	■							K.ALDAAIAK.V
264		412.7115	823.4084	823.4076	0.0009	0	18	0.016	1	U		■						K.DFSVASAK.V
332		424.2569	846.4992	846.4447	0.0546	0	16	0.03	1	U					■			K.AATTADGLK.A
358		430.2477	858.4808	859.4399	-0.9591	0	6	0.26	1	U						■		K.AQDVNVSK.D
448	2	466.2506	930.4866	930.4883	-0.0016	0	61	3.3e-06	1		■	■	■	■	■	■	■	R.SSLGAVQNR
470		471.7843	941.5540	941.5546	-0.0005	0	24	0.0037	1	U		■						K.VPTSGAVALK.S
477		473.2664	944.5182	944.5179	0.0004	0	35	0.001	1	U		■						K.IDADTLGLK.D
478		473.2818	944.5490	945.5243	-0.9753	1	3	1.7	1	U					■			K.AATTADRLK.A

Query	Dupes	Observed	Mr(expt)	Mr(calc)	Delta	M	Score	Expect	Rank	U	1	2	3	4	5	6	7	Peptide
575		502.2618	1002.5090	1002.5094	-0.0004	1	31	0.0043	▶1		■		■	■	■	■	■	K.SRLDEIDR.V
576		335.1778	1002.5116	1002.5094	0.0022	1	37	0.0012	▶1		■		■	■	■	■	■	K.SRLDEIDR.V
582		503.2722	1004.5298	1004.5325	-0.0026	0	32	0.00065	▶1	U		■						K.VLATNQTMK.I
592	▶2	505.7609	1009.5072	1009.5080	-0.0008	0	67	2e-07	▶1	U	■							K.YAVVDSATGK.Y
626		516.7829	1031.5512	1031.5512	0.0000	0	39	0.00013	▶1	U		■						K.AVQIANFGGR.V
685		538.2934	1074.5722	1074.5743	-0.0021	0	28	0.0027	▶1	U		■						K.GMTTAKPLEK.L
686		359.1989	1074.5749	1074.5743	0.0006	0	9	0.18	▶1	U		■						K.GMTTAKPLEK.L
735	▶2	551.2671	1100.5196	1100.5210	-0.0014	0	65	2.9e-06	▶1		■	■	■	■	■	■	■	K.DDAAGQAIANR.F
832	▶1	582.7962	1163.5778	1163.5782	-0.0004	0	58	5.1e-06	▶1	U		■						K.SQSSLSSAIER.L
890		600.8535	1199.6924	1199.6734	0.0190	1	3	0.52	▶1	U		■						K.LRSSLGAVQNR.F
900	▶3	603.3098	1204.6050	1204.6048	0.0003	0	58	3e-06	▶1		■		■					K.NQSALSTSIER.L
916		608.7778	1215.5410	1215.5408	0.0002	0	68	1.4e-07	▶1	U		■						R.TDDVNTYFGGK.A
947		618.3222	1234.6298	1234.6306	-0.0008	0	54	2.4e-05	▶1	U		■						R.VSQQTQFNGVK.V
1115		672.8776	1343.7406	1343.7408	-0.0002	0	61	8.9e-07	▶1	U						■		-.SLSLITQNNINK.N
1120		675.3386	1348.6626	1348.6470	0.0156	0	14	0.041	▶1	U				■				K.GSVANTAATSDDLK.L
1167		463.2525	1386.7357	1386.7354	0.0002	0	39	0.00016	▶1	U	■							K.LTAADGTAIAAADVK.D
1169	▶1	694.3785	1386.7424	1386.7354	0.0070	0	117	2.2e-12	▶1	U	■							K.LTAADGTAIAAADVK.D
1173	▶6	694.8293	1387.6440	1387.6442	-0.0002	0	110	1.1e-11	▶1	U	■							K.GFTVSGMADFSAAK.L
1242		721.3343	1440.6540	1439.8096	0.8444	0	34	0.0016	▶1		■			■	■	■	■	K.AQIIQQAGNSVLAK.A
1264	▶3	728.9094	1455.8042	1455.8045	-0.0003	0	112	1e-11	▶1	U			■					K.AQIIQQAGNSVLSK.A
1319	▶1	747.9182	1493.8218	1493.8202	0.0017	0	68	9.6e-07	▶1	U	■		■	■	■		■	K.ANQVPQQVLSLxQG.-
1348		506.9349	1517.7829	1517.7950	-0.0122	0	21	0.0075	▶1	U							■	K.ANQVPQQVLSLHQG.-
1419	▶2	789.4178	1576.8210	1576.8209	0.0001	0	94	4.6e-10	▶1		■		■	■				R.VSGQTQFNGVNVLSK
1430		794.4202	1586.8258	1586.8264	-0.0006	0	75	3.5e-08	▶1	U		■						K.SLAVNIDGNGNTSVVK.D
1456		807.9129	1613.8112	1613.8121	-0.0009	1	87	2e-08	▶1		■	■	■	■	■	■	■	R.INSAKDDAAGQAIANR.F
1457		538.9448	1613.8126	1613.8121	0.0005	1	33	0.0041	▶1		■	■	■	■	■	■	■	R.INSAKDDAAGQAIANR.F
1508	▶1	836.3835	1670.7524	1670.7457	0.0067	0	126	1.6e-12	▶1		■		■	■	■	■	■	R.IQDADYATEVSNMSK.A
1524		844.3778	1686.7410	1686.7407	0.0004	0	118	1.3e-11	▶1		■		■	■	■	■	■	R.IQDADYATEVSNMSK.A + Oxidation (M)
1551	▶1	858.8721	1715.7296	1715.7308	-0.0012	0	117	4.2e-12	▶1	U		■						R.IEDSDYATEVSNMSR.A
1585	▶1	871.4382	1740.8618	1740.8530	0.0089	0	102	6.2e-11	▶1	U	■							K.QVNLLSYTDTASNSTK.Y
1609	▶2	885.9641	1769.9136	1769.9159	-0.0023	0	76	2.4e-08	▶1	U		■						K.IQVGANDGQTIEIGLDK.I
1652		601.9721	1802.8945	1803.9438	-1.0494	1	0	4.2	▶1					■	■			K.NQSALSSSIERLSSGLR.I
1658		605.9867	1814.9383	1814.9374	0.0009	1	42	0.0001	▶1	U	■							K.LTAADGTAIAAADVKDAGGK.Q
1724		953.4635	1904.9124	1905.9466	-1.0341	1	5	0.34	▶1	U						■		K.VKDMTITSAGGNAQVATDK.A
1753		980.4387	1958.8628	1958.8640	-0.0011	1	57	4.2e-06	▶1	U		■						R.SRIEDSDYATEVSNMSR.A
1754		653.9618	1958.8636	1958.8640	-0.0004	1	30	0.0025	▶1	U		■						R.SRIEDSDYATEVSNMSR.A
1774		997.5038	1992.9930	1992.9865	0.0066	0	134	9.3e-14	▶1	U		■						R.FDSAITNLGNTVNNLSSAR.S
1777		499.4690	1993.8469	1992.9865	0.8604	0	26	0.0058	▶1	U		■						R.FDSAITNLGNTVNNLSSAR.S
1813		695.7141	2084.1205	2084.1225	-0.0021	0	51	5.6e-05	▶1		■	■	■	■	■		■	M.AQVINTNSLSLiTQNNiNK.N
1814	▶1	1043.0690	2084.1234	2084.1225	0.0009	0	127	1.2e-12	▶1		■	■	■	■	■		■	M.AQVINTNSLSLiTQNNiNK.N
1845		724.7039	2171.0899	2171.0859	0.0040	0	69	1.2e-07	▶1	U		■						R.VTAFVDDGTAAHNALSVDLQK.G
1846		1086.5590	2171.1034	2171.0859	0.0176	0	114	4.3e-12	▶1	U		■						R.VTAFVDDGTAAHNALSVDLQK.G
1887	▶2	1125.0530	2248.0914	2248.0931	-0.0017	0	138	1.1e-13	▶1		■			■	■	■		R.LDSAVTNLNNTTTNLSEAQSR.I
1888		750.3719	2248.0939	2248.0931	0.0008	0	84	2.6e-08	▶1		■			■	■	■		R.LDSAVTNLNNTTTNLSEAQSR.I
1928		773.7362	2318.1868	2318.1866	0.0001	1	62	1.3e-06	▶1		■		■	■				R.LDEIDRVSGQTQFNGVNVLSK
1970	▶2	1269.6030	2537.1914	2537.1980	-0.0066	0	165	2.9e-17	▶1	U	■							R.ELTVQASTGTNSDSDLSSIQDEIK.S
1971	▶1	846.7397	2537.1973	2537.1980	-0.0008	0	37	0.00018	▶1	U	■							R.ELTVQASTGTNSDSDLSSIQDEIK.S
1992	▶1	871.7690	2612.2852	2612.2790	0.0062	0	84	3.8e-09	▶1	U	■							R.NANDGISLAQTAEGALSEINNNLQR.I
1993		1307.1500	2612.2854	2612.2790	0.0065	0	143	4.5e-15	▶1	U	■							R.NANDGISLAQTAEGALSEINNNLQR.I
1998		881.7714	2642.2924	2642.2896	0.0028	0	53	9.3e-06	▶1			■	■					R.NANDGISLAQTTEGALSEINNNLQR.V
1999		1322.1560	2642.2974	2642.2896	0.0079	0	104	6.3e-11	▶1			■	■					R.NANDGISLAQTTEGALSEINNNLQR.V
2022	▶1	899.8187	2696.4343	2696.4232	0.0110	1	77	1.9e-08	▶1	U		■						K.IQVGANDGQTIEIGLDKIDADTLGLK.D
2041		933.5004	2797.4794	2797.4821	-0.0028	0	57	2.1e-06	▶1	U	■							K.IQIGANDNQTISIGLQQIDSTTLNLK.G
2042		1399.7490	2797.4834	2797.4821	0.0013	0	110	9.2e-12	▶1	U	■							K.IQIGANDNQTISIGLQQIDSTTLNLK.G
2048		936.4433	2806.3081	2806.3832	-0.0751	1	18	0.031	▶1	U	■							R.IRELTVQASTGTNSDSDLSSIQDEIK.S
2048		936.4433	2806.3081	2806.3832	-0.0752	1	17	0.039	▶2	U			■					R.VRELTVQATTGTNSDSDLSSIQDEIK.S
2056		1416.6830	2831.3514	2831.3533	-0.0019	0	111	7.1e-12	▶1	U		■						R.ELAVQATNGTNSQSDLNSIQDEITQR.L
2057		944.7932	2831.3578	2831.3533	0.0045	0	89	1.4e-09	▶1	U		■						R.ELAVQATNGTNSQSDLNSIQDEITQR.L
2118	▶1	1091.2470	3270.7192	3270.7167	0.0024	1	116	3.7e-12	▶1		■		■					M.AQVINTNSLSLITQNNINKNQSALSTSIER.L
2131		1114.8380	3341.4922	3341.6559	-0.1638	1	1	0.87	▶1	U		■						K.GLSQASRNANDGISLAQTTEGALSEINNNLQR.V

▶59 subsets and intersections (163 subset proteins in total)

10 per page 1

Not what you expected? Try the select summary.

Mascot: http://www.matrixscience.com/
