# Supplementary material for: MS-H: A Novel Proteomic Approach to Isolate and Type the E. coli H Antigen Using Membrane Filtration and Liquid Chromatography-Tandem Mass Spectrometry (LC-MS/MS)
Source: PLoS One. 2013 Feb 21;8(2):e57339. doi: 10.1371/journal.pone.0057339 (PMC3578835; doi:10.1371/journal.pone.0057339)
Supplement: Representative Peptide Data S1 — Peptide data are represented as the Mascot search results from all 53 serotypes, obtained under the Orbitrap platform in Table 4 with related E. coli reference strains. “U” denotes a unique peptide specific for each of the proteins 1.1, 1.2, and beyond. The number 1.1 (shown as 1 in the peptide list and phylogenetic tree) represents the protein which obtained the highest score and confidence value after a Mascot search. This protein, known as the first hit, was used to designate the MS-H type of the unknown flagellin. Related peptides 1.2 (2), 1.3 (3), etc. represented the second, third, etc. hits for MS-H typing analysis. (DOCX) [file pone.0057339.s009.docx › H5-E173.pdf]

# MASCOT Search Results

**User** :
**E-mail** :
**Search title** : Submitted from 20110714-H1-H11 by Mascot Daemon on VARIABLE
**MS data file** : C:\Documents and Settings\keding\Desktop\Raw data\20110714-H1-H11\20110714-008-E173MS1.RAW
**Database** : Flagellin_v2 (192 sequences; 89,845 residues)
**Taxonomy** : Bacteria (Eubacteria) (192 sequences)
**Timestamp** : 15 Jul 2011 at 17:32:25 GMT

Not what you expected? Try **the select summary**.

**Search parameters**

**Score distribution**

**Legend**

## Protein Family Summary

Significance threshold p< 0.05 Max. number of families AUTO
Ions score or expect cut-off 0 Dendrograms cut at 0

## Protein family 1 (out of 1)

10 per page 1

**1**

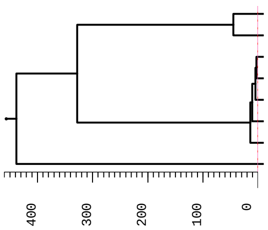


| | | | |
|---|---|---|---|
| 1 | gi\|6009835 | 1562 | H5 0\|0\|U4-41 [gi\|30059852\|ref\|AAP13298\| H5 0\|H5\|U4-41] |
| 4 | gi\|121144335 | 586 | H55 0\|O75:H55\|E2987-73 |
| 3 | gi\|46093545 | 736 | H3 0\|0\|Bi7327-41 |
| 8 | gi\|46093563 | 153 | H54 0\|0\|E223-69 |
| 7 | gi\|16901492 | 266 | H8 0\|H8\|0 |
| 5 | gi\|33590243 | 349 | H16 NM\|O157:NM(H16)\|PC02 |
| 6 | gi\|99645039 | 276 | H27 0\|H27\|0 |
| 2 | gi\|6009833 | 1236 | H4 0\|0\|U9-41 [gi\|56383039\|ref\|CAE53941\| H4 0\|O5:K4:H4\|U1-41] [gi\|30059850\|ref\|AAP13297\|... |

Threshold (**0**): 0

| | | Score | Mass | Matches | Sequences | emPAI |
|---|---|---|---|---|---|---|
| 1.1 | gi\|6009835<br>H5 0\|0\|U4-41 [gi\|30059852\|ref\|AAP13298\| H5 0\|H5\|U4-41]<br>**1 sameset of gi\|6009835** | 1562 | 45439 | 41 (28) | 26 (22) | 5.20 |
| 1.2 | gi\|6009833<br>H4 0\|0\|U9-41 [gi\|56383039\|ref\|CAE53941\| H4 0\|O5:K4:H4\|U1-41] [gi\|30059850\|ref\|AAP13297\| H4 0\|H4\|U9-41] [gi\|218695530\|ref\|YP_002403197\| Hxx 0\|0\|55989] [gi\|218352262\|ref\|CAU98018\| Hx... | 1236 | 36268 | 35 (21) | 23 (16) | 4.26 |
| 1.3 | gi\|46093545<br>H3 0\|0\|Bi7327-41 | 736 | 55534 | 28 (12) | 17 (9) | 0.88 |
| 1.4 | gi\|121144335<br>H55 0\|O75:H55\|E2987-73 | 586 | 62285 | 24 (13) | 16 (10) | 0.85 |
| 1.5 | gi\|33590243<br>H16 NM\|O157:NM(H16)\|PC02<br>**2 samesets of gi\|33590243** | 349 | 55093 | 18 (7) | 11 (6) | 0.42 |
| 1.6 | gi\|99645039<br>H27 0\|H27\|0 | 276 | 50847 | 13 (5) | 8 (5) | 0.37 |
| 1.7 | gi\|16901492<br>H8 0\|H8\|0<br>**3 samesets of gi\|16901492** | 266 | 52025 | 15 (6) | 10 (5) | 0.36 |
| 1.8 | gi\|46093563<br>H54 0\|0\|E223-69 | 153 | 54419 | 17 (5) | 11 (5) | 0.34 |

**98 peptide matches (78 non-duplicate, 20 duplicate)**

| Query | Dupes | Observed | Mr(expt) | Mr(calc) | Delta | M | Score | Expect | Rank | U | 1 | 2 | 3 | 4 | 5 | 6 | 7 | 8 | Peptide |
|---|---|---|---|---|---|---|---|---|---|---|---|---|---|---|---|---|---|---|---|
| 5 | 1 | 301.1999 | 600.3852 | 600.3595 | 0.0258 | 0 | 4 | 0.37 | 3 | U | | | | | | | | ■ | K.LNVQK.A |
| 15 | | 308.1953 | 614.3760 | 615.3591 | -0.9831 | 0 | 4 | 0.75 | 2 | U | | | ■ | | | | | | K.NLEIK.Q |
| 16 | 1 | 308.6873 | 615.3600 | 616.3180 | -0.9580 | 0 | 16 | 0.05 | 1 | U | | | | | | | | ■ | K.IGGADGK.T |
| 17 | 1 | 308.6873 | 615.3600 | 615.3592 | 0.0009 | 0 | 18 | 0.034 | 1 | U | ■ | | | | | | | | K.DQLIK.V |
| 28 | 1 | 315.6857 | 629.3568 | 629.3860 | -0.0292 | 1 | 3 | 0.52 | 1 | | | | ■ | | ■ | | | | K.VDKLR.S |
| 34 | 1 | 316.6898 | 631.3650 | 631.3653 | -0.0003 | 0 | 25 | 0.033 | 1 | | ■ | ■ | ■ | ■ | ■ | ■ | ■ | ■ | R.LSSGLR.I |
| 106 | | 351.7057 | 701.3968 | 700.4119 | 0.9849 | 1 | 2 | 0.91 | 2 | U | | | | | | | ■ | | K.DPTKLK.A |
| 118 | | 355.1978 | 708.3810 | 708.3806 | 0.0004 | 0 | 6 | 0.78 | 1 | | ■ | ■ | ■ | ■ | | | | | R.FTSNIK.G |
| 126 | 1 | 358.7056 | 715.3966 | 715.3977 | -0.0010 | 0 | 32 | 0.0046 | 1 | | ■ | ■ | | ■ | | | | | K.GLTQAAR.N |
| 137 | 1 | 366.2266 | 730.4386 | 730.4225 | 0.0162 | 0 | 2 | 2.1 | 1 | U | | | ■ | | | | | | K.LDTALAK.V |
| 138 | 1 | 366.6979 | 731.3812 | 731.3813 | -0.0001 | 0 | 4 | 1.3 | 1 | | | | ■ | | ■ | | ■ | | R.LSEIDR.V |
| 159 | | 380.2033 | 758.3920 | 758.4174 | -0.0253 | 0 | 30 | 0.0054 | 1 | U | | | | | ■ | | | | K.LDEALAK.V |
| 162 | 1 | 380.6957 | 759.3768 | 759.3763 | 0.0006 | 0 | 30 | 0.0062 | 1 | | ■ | ■ | | ■ | | | | | R.LDEIDR.V |
| 162 | 1 | 380.6957 | 759.3768 | 758.3922 | 0.9846 | 0 | 22 | 0.033 | 2 | U | | | | | | | | ■ | R.LNEIDR.V |
| 171 | | 386.7316 | 771.4486 | 771.4490 | -0.0004 | 0 | 12 | 0.057 | 1 | U | | ■ | | | | | | | K.ALDAAIAK.V |
| 217 | 1 | 403.7159 | 805.4172 | 805.4182 | -0.0009 | 0 | 24 | 0.0041 | 1 | U | ■ | | | | | | | | K.DLTTATGK.T |
| 324 | 1 | 430.7083 | 859.4020 | 860.4240 | -1.0219 | 0 | 18 | 0.016 | 1 | U | | | | | | | ■ | | K.VELGGSDGK.T |
| 423 | 2 | 466.2507 | 930.4868 | 930.4883 | -0.0014 | 0 | 65 | 1.4e-06 | 1 | | ■ | ■ | ■ | ■ | ■ | | | | R.SSLGAVQNR |

| Query | Dupes | Observed | Mr(expt) | Mr(calc) | Delta | M | Score | Expect | Rank | U | 1 | 2 | 3 | 4 | 5 | 6 | 7 | 8 | Peptide |
|---|---|---|---|---|---|---|---|---|---|---|---|---|---|---|---|---|---|---|---|
| 499 | | 487.2745 | 972.5344 | 971.5400 | 0.9945 | 0 | 6 | 0.31 | ▶1 | U | | | | | | | | ■ | K.ALAQVDSLR.S |
| 504 | ▶1 | 489.2248 | 976.4350 | 976.5077 | -0.0726 | 0 | 5 | 0.3 | ▶1 | U | | | | | | | | ■ | K.TETVTIGEK.T |
| 523 | | 495.2114 | 988.4082 | 987.5349 | 0.8734 | 0 | 7 | 0.22 | ▶1 | U | | | | | | | ■ | | K.ALSQVDSLR.S |
| 547 | | **502.2507** | **1002.4868** | **1002.5094** | **-0.0226** | **1** | **21** | **0.046** | ▶1 | | ■ | ■ | | ■ | | | | | **K.SRLDEIDR.V** |
| 548 | | 335.1776 | 1002.5110 | 1002.5094 | 0.0016 | 1 | 15 | 0.2 | ▶1 | | ■ | ■ | | ■ | | | | | K.SRLDEIDR.V |
| 562 | | **505.7608** | **1009.5070** | **1009.5080** | **-0.0010** | **0** | **26** | **0.0027** | ▶1 | **U** | | ■ | | | | | | | **K.YAVVDSATGK.Y** |
| 585 | ▶1 | **510.7762** | **1019.5378** | **1019.5400** | **-0.0021** | **0** | **50** | **1.4e-05** | ▶1 | **U** | ■ | | | | | | | | **K.AIAQVDTFR.S** |
| 668 | | 536.3241 | 1070.6336 | 1069.5768 | 1.0569 | 0 | 0 | 0.91 | ▶1 | U | | | ■ | | | | | | K.QVLDADGKPK.A |
| 673 | | 539.2703 | 1076.5260 | 1077.4873 | -0.9612 | 0 | 10 | 0.15 | ▶1 | U | | | | | ■ | | | | K.NDGSAQAIMR.E + Oxidation (M) |
| 730 | | **551.2673** | **1100.5200** | **1100.5210** | **-0.0010** | **0** | **63** | **4.9e-06** | ▶1 | | ■ | ■ | ■ | ■ | ■ | ■ | ■ | ■ | **K.DDAAGQAIANR.F** |
| 892 | | **596.3013** | **1190.5880** | **1190.5891** | **-0.0010** | **0** | **34** | **0.0021** | ▶1 | **U** | | | | | | ■ | | | **K.NQSALSSSIER.L** |
| 893 | | 397.8892 | 1190.6458 | 1190.5891 | 0.0567 | 0 | 12 | 0.38 | ▶1 | U | | | | | | ■ | | | K.NQSALSSSIER.L |
| 913 | | **603.3091** | **1204.6036** | **1204.6048** | **-0.0011** | **0** | **73** | **8.9e-08** | ▶1 | | ■ | ■ | | | | | | | **K.NQSALSTSIER.L** |
| 933 | | 406.5468 | 1216.6186 | 1216.6299 | -0.0113 | 0 | 3 | 0.51 | ▶1 | U | | | | | | | | ■ | K.EINSQTLGLDK.L |
| 1025 | | 423.5067 | 1267.4983 | 1268.6725 | -1.1742 | 1 | 0 | 0.98 | ▶1 | U | | | | | | | | ■ | K.DVDGVPQKGLNK.I |
| 1092 | ▶1 | 652.3961 | 1302.7776 | 1302.6415 | 0.1361 | 0 | 9 | 0.28 | ▶1 | U | | | | | | ■ | | | K.AATASDLDLNNAK.K |
| 1160 | | **672.8784** | **1343.7422** | **1343.7408** | **0.0014** | **0** | **58** | **1.7e-06** | ▶1 | **U** | | | | ■ | | | | | **-.SLSLITQNNINK.N** |
| 1180 | | 683.3226 | 1364.6306 | 1364.6783 | -0.0477 | 0 | 5 | 0.31 | ▶1 | U | | | | ■ | | | | | K.GSVSNTAATTDTLK.L |
| 1204 | | **694.3748** | **1386.7350** | **1386.7354** | **-0.0004** | **0** | **112** | **7.8e-12** | ▶1 | **U** | | ■ | | | | | | | **K.LTAADGTAIAAADVK.D** |
| 1206 | ▶1 | **694.8300** | **1387.6454** | **1387.6442** | **0.0012** | **0** | **92** | **5.7e-10** | ▶1 | **U** | | ■ | | | | | | | **K.GFTVSGMADFSAAK.L** |
| 1277 | | 480.9437 | 1439.8093 | 1439.8096 | -0.0003 | 0 | 2 | 2.9 | ▶1 | U | | | | ■ | | | | | K.AQIIQQAGNSVLAK.A |
| 1285 | | 723.8675 | 1445.7204 | 1445.7038 | 0.0166 | 0 | 13 | 0.052 | ▶1 | U | | | | | | | ■ | | K.AATAETTYFGSTVK.L |
| 1304 | | **728.9092** | **1455.8038** | **1455.8045** | **-0.0007** | **0** | **115** | **4.3e-12** | ▶1 | | ■ | ■ | | | | | | | **K.AQIIQQAGNSVLSK.A** |
| 1351 | | 747.9185 | 1493.8224 | 1493.8202 | 0.0023 | 0 | 13 | 0.3 | ▶1 | | ■ | ■ | | | | | | | K.ANQVPQQVLSLLQG.- |
| 1421 | | **781.4195** | **1560.8244** | **1560.8260** | **-0.0016** | **0** | **70** | **4.4e-07** | ▶1 | | ■ | | | ■ | | | | | **R.VSGQTQFNGVNVLAK.D** |
| 1443 | | 789.0634 | 1576.1122 | 1576.8209 | -0.7087 | 0 | 2 | 0.81 | ▶1 | U | | ■ | | | | | | | R.VSGQTQFNGVNVLSK.N |
| 1471 | | 538.9446 | 1613.8120 | 1613.8121 | -0.0001 | 1 | 21 | 0.071 | ▶1 | | ■ | ■ | ■ | ■ | ■ | ■ | ■ | ■ | R.INSAKDDAAGQAIANR.F |
| 1519 | | **836.3800** | **1670.7454** | **1670.7457** | **-0.0003** | **0** | **113** | **2.8e-11** | ▶1 | | ■ | ■ | | ■ | | | | | **R.IQDADYATEVSNMSK.A** |
| 1537 | | **844.3773** | **1686.7400** | **1686.7407** | **-0.0006** | **0** | **100** | **6.9e-10** | ▶1 | | ■ | ■ | | ■ | | | | | **R.IQDADYATEVSNMSK.A + Oxidation (M)** |
| 1560 | | **571.9716** | **1712.8930** | **1712.8945** | **-0.0015** | **0** | **36** | **0.00028** | ▶1 | **U** | ■ | | | | | | | | **K.TVDVSSLTLHNTLDAK.G** |
| 1561 | | **857.4551** | **1712.8956** | **1712.8945** | **0.0012** | **0** | **87** | **2.2e-09** | ▶1 | **U** | ■ | | | | | | | | **K.TVDVSSLTLHNTLDAK.G** |
| 1568 | | 860.3567 | 1718.6988 | 1718.7974 | -0.0985 | 0 | 1 | 0.84 | ▶1 | U | | | | | ■ | | | | K.ALAYNDAPMSVYFGGK.N + Oxidation (M) |
| 1591 | | 581.6310 | 1741.8712 | 1742.8112 | -0.9400 | 0 | 3 | 0.49 | ▶1 | U | ■ | | | | | | | | K.VAADSDGSAAGYVTFQGK.N |
| 1593 | | **872.4138** | **1742.8130** | **1742.8112** | **0.0019** | **0** | **74** | **3.7e-08** | ▶1 | **U** | ■ | | | | | | | | **K.VAADSDGSAAGYVTFQGK.N** |
| 1609 | | **878.4236** | **1754.8326** | **1754.8323** | **0.0004** | **0** | **103** | **1e-10** | ▶1 | **U** | ■ | | | | | | | | **K.NYATTVSTALDDNTAAK.A** |
| 1621 | ▶1 | **885.9644** | **1769.9142** | **1769.9159** | **-0.0017** | **0** | **91** | **8.1e-10** | ▶1 | **U** | | | ■ | | | | | | **K.IQVGANDGQTIEIGLDK.I** |
| 1661 | | 601.9707 | 1802.8903 | 1803.9438 | -1.0536 | 1 | 0 | 4.3 | ▶2 | U | | | | | | ■ | | | K.NQSALSSSIERLSSGLR.I |
| 1669 | | 606.4342 | 1816.2808 | 1814.9374 | 1.3434 | 1 | 0 | 0.97 | ▶1 | U | | ■ | | | | | | | K.LTAADGTAIAAADVKDAGGK.Q |
| 1704 | | 616.9984 | 1847.9734 | 1848.9065 | -0.9332 | 1 | 1 | 0.76 | ▶1 | U | | | | ■ | | | | | K.AQDVNVSKDGTITTTDGK.S |
| 1792 | | 653.8126 | 1958.4160 | 1958.8640 | -0.4480 | 1 | 2 | 1.2 | ▶1 | U | | | ■ | | | | | | R.SRIEDSDYATEVSNMSR.A |
| 1815 | | **997.5024** | **1992.9902** | **1992.9865** | **0.0038** | **0** | **128** | **4e-13** | ▶1 | | | | ■ | | ■ | ■ | ■ | | **R.FDSAITNLGNTVNNLSSAR.S** |
| 1816 | | 499.4688 | 1993.8461 | 1992.9865 | 0.8596 | 0 | 4 | 1 | ▶1 | | | | ■ | | ■ | ■ | ■ | | R.FDSAITNLGNTVNNLSSAR.S |
| 1827 | | **1007.4670** | **2012.9194** | **2012.9175** | **0.0020** | **0** | **93** | **4.7e-10** | ▶1 | **U** | ■ | | | | | | | | **K.ADVEYTDTDNGLTTAATQK.D** |
| 1854 | | **1043.0690** | **2084.1234** | **2084.1225** | **0.0009** | **0** | **107** | **1.3e-10** | ▶1 | | ■ | ■ | ■ | | ■ | ■ | ■ | ■ | **M.AQVINTNSLSLiTQNNiNK.N** |
| 1855 | | 695.7157 | 2084.1253 | 2084.1225 | 0.0027 | 0 | 11 | 0.23 | ▶1 | | ■ | ■ | ■ | | ■ | ■ | ■ | ■ | M.AQVINTNSLSLiTQNNiNK.N |
| 1890 | | **724.7023** | **2171.0851** | **2171.0859** | **-0.0008** | **0** | **51** | **8.6e-06** | ▶1 | **U** | | | ■ | | | | | | **R.VTAFVDDGTAAHNALSVDLQK.G** |
| 1891 | | **1086.5510** | **2171.0874** | **2171.0859** | **0.0016** | **0** | **82** | **6.8e-09** | ▶1 | **U** | | | ■ | | | | | | **R.VTAFVDDGTAAHNALSVDLQK.G** |
| 1926 | | **1125.0550** | **2248.0954** | **2248.0931** | **0.0023** | **0** | **137** | **1.1e-13** | ▶1 | | ■ | ■ | | ■ | | | | | **R.LDSAVTNLNNTTTNLSEAQSR.I** |
| 1927 | | **750.3725** | **2248.0957** | **2248.0931** | **0.0026** | **0** | **58** | **1.1e-05** | ▶1 | | ■ | ■ | | ■ | | | | | **R.LDSAVTNLNNTTTNLSEAQSR.I** |
| 1987 | ▶1 | **1269.6080** | **2537.2014** | **2537.1980** | **0.0034** | **0** | **145** | **3.2e-15** | ▶1 | **U** | | ■ | | | | | | | **R.ELTVQASTGTNSDSDLSSIQDEIK.S** |
| 1993 | | **1276.6130** | **2551.2114** | **2551.2137** | **-0.0023** | **0** | **157** | **2.9e-16** | ▶1 | **U** | ■ | | | | | | | | **R.ELTVQATTGTNSDSDLSSIQDEIK.S** |
| 1996 | | 855.3967 | 2563.1683 | 2564.2864 | -1.1181 | 1 | 5 | 0.35 | ▶1 | U | | | | ■ | | | | | -.SLSLITQNNINKNQSSMSTAIER.L + Oxidation (M) |
| 2009 | | **881.7728** | **2642.2966** | **2642.2896** | **0.0070** | **0** | **52** | **1.2e-05** | ▶1 | | ■ | | ■ | | | | | | **R.NANDGISLAQTTEGALSEINNNLQR.V** |
| 2010 | | **1322.1560** | **2642.2974** | **2642.2896** | **0.0079** | **0** | **154** | **6.5e-16** | ▶1 | | ■ | | ■ | | | | | | **R.NANDGISLAQTTEGALSEINNNLQR.V** |
| 2026 | | **899.8173** | **2696.4301** | **2696.4232** | **0.0068** | **1** | **54** | **4.4e-06** | ▶1 | **U** | | | ■ | | | | | | **K.IQVGANDGQTIEIGLDKIDADTLGLK.D** |
| 2027 | | **901.0882** | **2700.2428** | **2700.2416** | **0.0012** | **0** | **74** | **4.2e-08** | ▶1 | **U** | ■ | | | | | | | | **K.GAATSQFVVQSGNDFYSASINHTDGK.V** |
| 2039 | | **933.5031** | **2797.4875** | **2797.4821** | **0.0053** | **0** | **39** | **0.00014** | ▶1 | **U** | | ■ | | | | | | | **K.IQIGANDNQTISIGLQQIDSTTLNLK.G** |
| 2040 | | **1399.7530** | **2797.4914** | **2797.4821** | **0.0093** | **0** | **54** | **3.7e-06** | ▶1 | **U** | | ■ | | | | | | | **K.IQIGANDNQTISIGLQQIDSTTLNLK.G** |
| 2042 | | 936.4431 | 2806.3075 | 2806.3832 | -0.0757 | 1 | 3 | 0.87 | ▶1 | U | | ■ | | | | | | | R.IRELTVQASTGTNSDSDLSSIQDEIK.S |
| 2049 | | 939.1757 | 2814.5053 | 2814.5015 | 0.0038 | 0 | 8 | 0.17 | ▶1 | U | ■ | | | | | | | | K.VVVELSTAKPTAQFSGASSADPLALLDK.A |

▶ **51 subsets and intersections (158 subset proteins in total)**

10 per page 1

Not what you expected? Try **the select summary**.

Mascot: http://www.matrixscience.com/
