# Supplementary material for: MS-H: A Novel Proteomic Approach to Isolate and Type the E. coli H Antigen Using Membrane Filtration and Liquid Chromatography-Tandem Mass Spectrometry (LC-MS/MS)
Source: PLoS One. 2013 Feb 21;8(2):e57339. doi: 10.1371/journal.pone.0057339 (PMC3578835; doi:10.1371/journal.pone.0057339)
Supplement: Representative Peptide Data S1 — Peptide data are represented as the Mascot search results from all 53 serotypes, obtained under the Orbitrap platform in Table 4 with related E. coli reference strains. “U” denotes a unique peptide specific for each of the proteins 1.1, 1.2, and beyond. The number 1.1 (shown as 1 in the peptide list and phylogenetic tree) represents the protein which obtained the highest score and confidence value after a Mascot search. This protein, known as the first hit, was used to designate the MS-H type of the unknown flagellin. Related peptides 1.2 (2), 1.3 (3), etc. represented the second, third, etc. hits for MS-H typing analysis. (DOCX) [file pone.0057339.s009.docx › H6-E174.pdf]

MASCOT Search Results

User :
E-mail :
Search title : Submitted from 20110714-H1-H11 by Mascot Daemon on VARIABLE
MS data file : C:\Documents and Settings\keding\Desktop\Raw data\20110714-H1-H11\20110714-009-E174MS1.RAW
Database : Flagellin_v2 (192 sequences; 89,845 residues)
Taxonomy : Bacteria (Eubacteria) (192 sequences)
Timestamp : 15 Jul 2011 at 17:34:44 GMT

Not what you expected? Try **the select summary**.

Search parameters

Score distribution

Legend

Protein Family Summary

Significance threshold p< 0.05 Max. number of families AUTO
Ions score or expect cut-off 0 Dendrograms cut at 0

Protein family 1 (out of 1)

10 per page 1

1

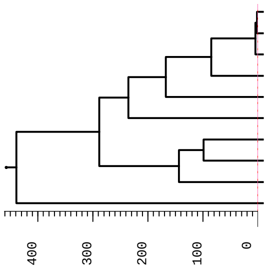

1	gi|30059854	5934	H6 0|H6|A20a
10	gi|46093552	508	H53 0|0|E480-68
5	gi|88683097	2381	H44 0|H44|0
9	gi|46093545	709	H3 0|0|Bi7327-41
4	gi|3377710	2606	H9 0|0|O78:K80; EC341 95 [gi|30059860|ref|AAP13302| H9 0|H9|Bi 7575-41]
3	gi|290439	2676	Hxx(H7 98.6%) 0|0|U5-41 (Orskov)
2	gi|300399814	4471	Hxx(H6 98.7%) 0|0|MS 69-1 [gi|301018227|ref|ZP_07182742| Hxx 0|0|MS 69-1]
6	gi|5107845	2053	H7 0|O55:H7|DEC 5d [gi|5107857|ref|AAD28529| Hxx 0|0|ECOR 37]
7	gi|121144335	1973	H55 0|O75:H55|E2987-73
8	gi|40994711	1886	H4 0|O154:H4|0

Threshold (**0**): 0

		Score	Mass	Matches	Sequences	emPAI
1.1	gi|30059854<br>H6 0|H6|A20a	5934	56213	135 (117)	47 (44)	35.16
1.2	gi|300399814<br>Hxx(H6 98.7%) 0|0|MS 69-1 [gi|301018227|ref|ZP_07182742| Hxx 0|0|MS 69-1]	4471	56260	119 (102)	43 (40)	20.53
1.3	gi|290439<br>Hxx(H7 98.6%) 0|0|U5-41 (Orskov)	2676	59752	76 (61)	25 (22)	4.27
1.4	gi|3377710<br>H9 0|0|O78:K80; EC341 95 [gi|30059860|ref|AAP13302| H9 0|H9|Bi 7575-41]	2606	68093	73 (58)	26 (22)	2.92
1.5	gi|88683097<br>H44 0|H44|0	2381	55289	63 (48)	18 (16)	2.57
1.6	gi|5107845<br>H7 0|O55:H7|DEC 5d [gi|5107857|ref|AAD28529| Hxx 0|0|ECOR 37]	2053	56638	60 (50)	18 (17)	2.88
1.7	gi|121144335<br>H55 0|O75:H55|E2987-73	1973	62285	61 (49)	19 (17)	2.27
1.8	gi|40994711<br>H4 0|O154:H4|0	1886	36224	51 (38)	21 (17)	5.87
1.9	gi|46093545<br>H3 0|0|Bi7327-41	709	55534	31 (16)	13 (7)	0.68
1.10	gi|46093552<br>H53 0|0|E480-68	508	44861	19 (12)	9 (5)	0.64

194 peptide matches (105 non-duplicate, 89 duplicate)

Query	Dupes	Observed	Mr(expt)	Mr(calc)	Delta	M	Score	Expect	Rank	U	1	2	3	4	5	6	7	8	9	10	Peptide
12		305.1663	608.3180	609.2428	-0.9248	0	6	0.26	1	U								■			K.NDSMK.I + Oxidation (M)
18	2	308.7055	615.3964	615.3591	0.0373	0	7	0.4	1	U									■		K.NLEIK.Q
35	3	**316.6901**	**631.3656**	**631.3653**	**0.0003**	**0**	**30**	**0.0089**	1		■	■	■	■	■	■	■	■	■	■	**R.LSSGLR.I**
62		330.7005	659.3864	659.3602	0.0262	0	7	0.2	1	U				■							K.QGNLTK.T
120	1	355.1978	708.3810	708.3806	0.0004	0	19	0.075	1		■	■	■	■	■	■	■	■	■	■	R.FTSNIK.G
125	3	**358.7060**	**715.3974**	**715.3977**	**-0.0002**	**0**	**32**	**0.0043**	1		■	■	■	■	■	■	■	■			**K.GLTQAAR.N**
127		358.7135	715.4124	715.4116	0.0009	0	10	0.57	1	U										■	K.IDIDLK.K
137		366.6984	731.3822	731.3813	0.0009	0	14	0.14	1	U									■		R.LSEIDR.V
139	1	**366.7166**	**731.4186**	**731.4177**	**0.0009**	**0**	**32**	**0.0021**	1		■	■									**K.LATATGAK.A**
140	2	366.7215	731.4284	730.4225	1.0060	0	5	1	2	U									■		K.LDTALAK.V
147	1	**373.7245**	**745.4344**	**745.4334**	**0.0010**	**0**	**29**	**0.0013**	1		■	■									**K.ATVTIGGK.D**
159	3	**380.6952**	**759.3758**	**759.3763**	**-0.0004**	**0**	**38**	**0.00095**	1		■	■	■	■	■	■	■	■			**R.LDEIDR.V**
175		386.7322	771.4498	771.4490	0.0008	0	11	0.072	1	U								■			K.ALDAAIAK.V
308	1	**424.2297**	**846.4448**	**846.4447**	**0.0002**	**0**	**54**	**5.1e-06**	1		■	■									**K.AATIQTDK.G**
328		428.7789	855.5432	854.3770	1.1662	0	1	0.73	1	U									■		K.QSGSGYEK.V

Query	Dupes	Observed	Mr(expt)	Mr(calc)	Delta	M	Score	Expect	Rank	U	1	2	3	4	5	6	7	8	9	10	Peptide
334		431.2267	860.4388	859.4399	0.9989	0	13	0.047	▶1	U				■							K.VNISQDGK.I
343	▶1	433.7270	865.4394	865.4393	0.0002	0	24	0.0045	▶1		■	■									K.LTTDTTSK.A
429	▶3	466.2508	930.4870	930.4883	-0.0012	0	79	5.8e-08	▶1		■	■		■	■		■	■	■		R.SSLGAVQNR
429	▶3	466.2508	930.4870	930.4883	-0.0012	0	28	0.0079	▶2	U			■								R.SSVGAIQNR.L
442		468.2557	934.4968	934.4872	0.0096	0	0	0.98	▶1	U					■						K.LSGFNVNGK.G
459	▶2	473.7263	945.4380	945.4403	-0.0023	0	39	0.0004	▶1		■	■									K.DQDVNIDK.S
493		322.8469	965.5189	965.5182	0.0007	1	19	0.014	▶1		■	■									K.LVYKGSDGK.L
494		484.2621	966.5096	965.5182	0.9915	1	13	0.051	▶1		■	■									K.LVYKGSDGK.L
550		502.2620	1002.5094	1002.5094	0.0000	1	33	0.0027	▶1		■	■	■	■	■	■	■	■			K.SRLDEIDR.V
551		335.1779	1002.5119	1002.5094	0.0025	1	37	0.001	▶1		■	■	■	■	■	■	■	■			K.SRLDEIDR.V
660	▶1	538.7819	1075.5492	1075.5509	-0.0017	0	55	4.2e-06	▶1		■	■									K.GETANTAATLK.D
664		539.2800	1076.5454	1076.5462	-0.0007	0	28	0.0022	▶1	U						■					-.QSALSSSIER.L
718	▶1	551.2674	1100.5202	1100.5210	-0.0008	0	65	2.9e-06	▶1		■	■	■	■	■	■	■	■	■	■	K.DDAAGQAIANR.F
779	▶1	570.7800	1139.5454	1139.5459	-0.0004	0	70	1.1e-07	▶1		■	■									K.NDTYTATVGAK.T
862	▶1	596.3013	1190.5880	1190.5891	-0.0010	0	61	4.4e-06	▶1		■	■	■	■							K.NQSALSSSIER.L
865		397.8896	1190.6470	1190.5891	0.0579	0	2	3.6	▶1		■	■	■	■							K.NQSALSSSIER.L
878		600.8538	1199.6930	1199.6734	0.0196	1	11	0.087	▶1	U									■		K.LRSSLGAVQNR.F
885		603.3096	1204.6046	1204.6048	-0.0001	0	19	0.023	▶1	U								■			K.NQSALSTSIER.L
980	▶3	630.8115	1259.6084	1259.6106	-0.0021	0	71	8.1e-08	▶1		■	■									K.NNAGGDTQATLAK.L
983		630.8248	1259.6350	1259.6721	-0.0371	0	15	0.034	▶1	U										■	K.TVANGGDIVLSSK.T
1069		655.8253	1309.6360	1309.6362	-0.0001	1	46	2.7e-05	▶1		■	■									K.GSDGKLTTDTTSK.A
1115		672.8781	1343.7416	1343.7408	0.0008	0	72	6.2e-08	▶1	U							■				-.SLSLITQNNINK.N
1119		675.3391	1348.6636	1348.6470	0.0166	0	30	0.0011	▶1	U				■							K.GSVANTAATSDDLK.L
1154		694.8301	1387.6456	1387.6442	0.0014	0	95	3.3e-10	▶1	U								■			K.GFTVSGMADFSAAK.L
1214	▶2	720.9119	1439.8092	1439.8096	-0.0004	0	117	8.9e-12	▶1		■	■		■	■	■	■	■			K.AQIIQQAGNSVLAK.A
1284		747.9186	1493.8226	1493.8202	0.0025	0	37	0.0013	▶1	U	■	■	■	■	■			■			K.ANQVPQQVLSLxQG.-
1314		759.4262	1516.8378	1517.7950	-0.9572	0	0	0.94	▶1	U					■						K.ANQVPQQVLSLHQG.-
1316	▶1	506.9342	1517.7808	1517.7950	-0.0143	0	18	0.017	▶1	U					■						K.ANQVPQQVLSLHQG.-
1354	▶2	781.4203	1560.8260	1560.8260	0.0000	0	70	4.4e-07	▶1		■		■		■	■	■				R.VSGQTQFNGVNVLAK.D
1400		535.3787	1603.1143	1601.8988	1.2154	1	6	0.27	▶1	U										■	K.TVANGGDIVLSSKTIK.A
1408		807.9133	1613.8120	1613.8121	-0.0001	1	99	1e-09	▶1		■	■	■	■	■	■	■	■	■	■	R.INSAKDDAAGQAIANR.F
1409	▶1	538.9450	1613.8132	1613.8121	0.0011	1	33	0.0043	▶1		■	■	■	■	■	■	■	■	■	■	R.INSAKDDAAGQAIANR.F
1417		811.4187	1620.8228	1619.8631	0.9597	1	0	0.93	▶1	U										■	K.KVAANTSGLAANQTFK.S
1446	▶1	832.4180	1662.8214	1662.8213	0.0001	0	93	8.4e-10	▶1		■	■	■			■					K.IDSDTLGLNGFNVNGK.G
1454	▶2	836.3821	1670.7496	1670.7457	0.0039	0	133	3.3e-13	▶1		■	■	■	■	■	■	■	■			R.IQDADYATEVSNMSK.A
1456		837.4388	1672.8630	1672.8632	-0.0001	1	77	1.9e-08	▶1		■	■									K.ATVTIGGKDQDVNIDK.S
1457		558.6290	1672.8652	1672.8632	0.0020	1	27	0.0018	▶1		■	■									K.ATVTIGGKDQDVNIDK.S
1478	▶12	843.4574	1684.9002	1684.8996	0.0007	0	120	4.2e-12	▶1		■				■						K.IQVGANDGQTITIDLK
1483	▶9	843.4602	1684.9058	1685.8836	-0.9777	0	61	3.1e-06	▶2			■	■	■		■	■				K.IQVGANDGETITIDLK.K
1485		844.3773	1686.7400	1686.7407	-0.0006	0	105	2.3e-10	▶1		■	■	■	■	■	■	■	■			R.IQDADYATEVSNMSK.A + Oxidation (M)
1489		847.3688	1692.7230	1692.8418	-0.1187	0	0	0.91	▶1	U				■							K.LTTEATTASSSTADPLK.A
1554	▶1	885.9638	1769.9130	1769.9159	-0.0029	0	88	1.7e-09	▶1	U									■		K.IQVGANDGQTIEIGLDK.I
1573	▶1	597.9793	1790.9161	1790.9163	-0.0002	1	52	5.6e-05	▶1		■	■	■			■					K.KIDSDTLGLNGFNVNGK.G
1575		896.4667	1790.9188	1790.9163	0.0026	1	103	4.4e-10	▶1		■	■	■			■					K.KIDSDTLGLNGFNVNGK.G
1595		601.9691	1802.8855	1803.9438	-1.0584	1	1	4.1	▶1		■	■	■	■							K.NQSALSSSIERLSSGLR.I
1602		605.3380	1812.9922	1813.9785	-0.9864	1	34	0.0015	▶1			■	■	■		■	■				K.IQVGANDGETITIDLKK.I
1602		605.3380	1812.9922	1812.9945	-0.0024	1	29	0.0047	▶2	U	■										K.IQVGANDGQTITIDLKK.I
1605		607.3022	1818.8848	1817.9595	0.9253	1	1	1	▶1	U								■			K.NQSALSTSIERLSSGLR.I
1633	▶1	620.3217	1857.9433	1857.9432	0.0001	0	73	5.5e-08	▶1	U	■										K.SAVASSVDILNAVAGADGNK.V
1634	▶1	929.9803	1857.9460	1857.9432	0.0028	0	108	1.5e-11	▶1	U	■										K.SAVASSVDILNAVAGADGNK.V
1736	▶1	1030.5090	2059.0034	2058.9957	0.0078	0	132	7.5e-14	▶1		■	■									K.AESTSDPLAALDDAISQIDK.F
1751	▶1	695.7144	2084.1214	2084.1225	-0.0012	0	73	3e-07	▶1		■	■	■	■	■			■	■	■	M.AQVINTNSLSLiTQNNiNK.N
1752	▶1	1043.0680	2084.1214	2084.1225	-0.0011	0	141	5e-14	▶1		■	■	■	■	■			■	■	■	M.AQVINTNSLSLiTQNNiNK.N
1812	▶4	1117.5550	2233.0954	2233.0903	0.0051	0	128	2e-13	▶1		■	■									K.VTTSADVGFGTPAAAVTYTYNK.D
1821		1123.0270	2244.0394	2244.0369	0.0026	0	86	2.5e-09	▶1		■	■									K.DMSGFTAAAAPGGTVGVTQYTDK.S
1822		749.0209	2244.0409	2244.0369	0.0040	0	62	7e-07	▶1		■	■									K.DMSGFTAAAAPGGTVGVTQYTDK.S
1827	▶3	1125.0530	2248.0914	2248.0931	-0.0017	0	130	6e-13	▶1		■	■	■	■		■	■	■			R.LDSAVTNLNNTTTNLSEAQSR.I
1830	▶1	750.3727	2248.0963	2248.0931	0.0032	0	81	4.4e-08	▶1		■	■	■	■		■	■	■			R.LDSAVTNLNNTTTNLSEAQSR.I
1834		1131.0230	2260.0314	2260.0318	-0.0004	0	111	7.3e-12	▶1		■	■									K.DMSGFTAAAAPGGTVGVTQYTDK.S + Oxidation (
1854		1149.5500	2297.0854	2297.0845	0.0009	0	105	3.5e-11	▶1		■	■									K.SGNLTAADDGAVLYMDATGNLTK.N
1855		766.7321	2297.1745	2297.0845	0.0899	0	25	0.0029	▶1		■	■									K.SGNLTAADDGAVLYMDATGNLTK.N
1858		768.4037	2302.1893	2302.1917	-0.0025	1	53	2.6e-05	▶1		■		■		■	■	■				R.LDEIDRVSGQTQFNGVNVLAK.D
1859		1152.1040	2302.1934	2302.1917	0.0017	1	38	0.00079	▶1		■		■		■	■	■				R.LDEIDRVSGQTQFNGVNVLAK.D
1865		1157.5480	2313.0814	2313.0795	0.0020	0	110	1e-11	▶1		■	■									K.SGNLTAADDGAVLYMDATGNLTK.N + Oxidation (
1885		788.3981	2362.1725	2362.1652	0.0073	1	46	2.3e-05	▶1		■	■									K.AESTSDPLAALDDAISQIDKFR.S
1908		1269.6080	2537.2014	2537.1980	0.0034	0	151	8.5e-16	▶1	U								■			R.ELTVQASTGTNSDSDLSSIQDEIK.S
1917		855.7370	2564.1892	2564.2864	-0.0972	1	4	1.4	▶1	U							■				-.SLSLITQNNINKNQSSMSTAIER.L + Oxidation (
1919	▶1	1283.6040	2565.1934	2565.1930	0.0005	0	131	2.4e-13	▶1		■		■	■							R.ELTVQASTGTNSDSDLDSIQDEIK.S
1919	▶1	1283.6040	2565.1934	2565.2294	-0.0359	0	52	2.1e-05	▶2	U		■									R.ELTVQATTGTNSTSDLDSIQDEIK.S
1920		856.0720	2565.1942	2565.1930	0.0012	0	60	3.1e-06	▶1		■		■	■							R.ELTVQASTGTNSDSDLDSIQDEIK.S
1920		856.0720	2565.1942	2565.2294	-0.0352	0	36	0.00079	▶2	U		■									R.ELTVQATTGTNSTSDLDSIQDEIK.S
1936	▶1	877.1017	2628.2833	2628.2739	0.0094	0	55	1.4e-05	▶1		■	■	■	■	■	■	■	■			R.NANDGISVAQTTEGALSEINNNLQR.I
1937	▶1	1315.1630	2628.3114	2628.2739	0.0375	0	142	2.8e-14	▶1		■	■	■	■	■	■	■	■			R.NANDGISVAQTTEGALSEINNNLQR.I
1955		899.8195	2696.4367	2696.4232	0.0134	1	23	0.0048	▶1	U									■		K.IQVGANDGQTIEIGLDKIDADTLGLK.D
1964		1363.6160	2725.2174	2725.2178	-0.0003	0	124	3.7e-13	▶1	U	■										K.GTFTSDGTAFDGASMSIDTNTFANAVK.N
1966	▶1	1371.6150	2741.2154	2741.2127	0.0028	0	96	2.3e-10	▶1	U	■										K.GTFTSDGTAFDGASMSIDTNTFANAVK.N + Oxidati
1967		914.7460	2741.2162	2741.2127	0.0035	0	28	0.0015	▶1	U	■										K.GTFTSDGTAFDGASMSIDTNTFANAVK.N + Oxidati
1976		933.5027	2797.4863	2797.4821	0.0041	0	35	0.00033	▶1	U								■			K.IQIGANDNQTISIGLQQIDSTTLNLK.G
1990	▶1	945.7990	2834.3752	2834.3781	-0.0030	1	107	6.1e-11	▶1		■		■	■							R.IRELTVQASTGTNSDSDLDSIQDEIK.S
1990	▶1	945.7990	2834.3752	2834.4145	-0.0393	1	43	0.00017	▶2	U		■									R.IRELTVQATTGTNSTSDLDSIQDEIK.S
1996		969.8159	2906.4259	2906.4244	0.0015	1	10	0.11	▶1		■	■									K.LTTDTTSKAESTSDPLAALDDAISQIDK.F
2014	▶1	1015.8050	3044.3932	3044.3847	0.0085	0	54	4.4e-06	▶1		■	■									K.DTNSYSAASDDISSANLAAFLNPQAGDTTK.A
2016	▶1	1523.2050	3044.3954	3044.3847	0.0108	0	100	1.1e-10	▶1		■	■									K.DTNSYSAASDDISSANLAAFLNPQAGDTTK.A

Query	Dupes	Observed	Mr(expt)	Mr(calc)	Delta	M	Score	Expect	Rank	U	1	2	3	4	5	6	7	8	9	10	Peptide
2039		1054.5100	3160.5082	3160.5708	-0.0627	1	17	0.057	1		■	■		■			■	■			R.SSLGAVQNRLDSAVTNLNNTTTNLSEAQSR.I
2039		1054.5100	3160.5082	3160.5708	-0.0627	1	16	0.07	2	U			■								R.SSVGAIQNRLDSAVTNLNNTTTNLSEAQSR.I
2048		1086.5760	3256.7062	3256.7011	0.0051	1	119	5.1e-12	1		■	■	■	■							M.AQVINTNSLSLITQNNINKNQSALSSSIER.L
2094		1185.5610	3553.6612	3553.6519	0.0093	1	93	4.9e-10	1	U	■										K.AATIQTDKGTFTSDGTAFDGASMSIDTNTFANAVK.N
2094		1185.5610	3553.6612	3553.6519	0.0093	1	31	0.00077	2	U		■									K.AATIQTDKGTFTSDGTTFDGASMSIDANTFANAVK.N
2100		1190.8880	3569.6422	3569.6468	-0.0046	1	40	9e-05	1	U	■										K.AATIQTDKGTFTSDGTAFDGASMSIDTNTFANAVK.N
2100		1190.8880	3569.6422	3569.6468	-0.0046	1	13	0.054	2	U		■									K.AATIQTDKGTFTSDGTTFDGASMSIDANTFANAVK.N

63 subsets and intersections (161 subset proteins in total)

10 per page 1

Not what you expected? Try **the select summary**.

Mascot: http://www.matrixscience.com/
