# Supplementary material for: MS-H: A Novel Proteomic Approach to Isolate and Type the E. coli H Antigen Using Membrane Filtration and Liquid Chromatography-Tandem Mass Spectrometry (LC-MS/MS)
Source: PLoS One. 2013 Feb 21;8(2):e57339. doi: 10.1371/journal.pone.0057339 (PMC3578835; doi:10.1371/journal.pone.0057339)
Supplement: Representative Peptide Data S1 — Peptide data are represented as the Mascot search results from all 53 serotypes, obtained under the Orbitrap platform in Table 4 with related E. coli reference strains. “U” denotes a unique peptide specific for each of the proteins 1.1, 1.2, and beyond. The number 1.1 (shown as 1 in the peptide list and phylogenetic tree) represents the protein which obtained the highest score and confidence value after a Mascot search. This protein, known as the first hit, was used to designate the MS-H type of the unknown flagellin. Related peptides 1.2 (2), 1.3 (3), etc. represented the second, third, etc. hits for MS-H typing analysis. (DOCX) [file pone.0057339.s009.docx › H7-EDL933.pdf]

MATRIX SCIENCE MASCOT Search Results

User :
E-mail :
Search title : Submitted from 20100706-H7-MS1 by Mascot Daemon on VARIABLE
MS data file : C:\Documents and Settings\keding\Desktop\Raw data\20110706-H7\20110706-001-EDL933MS1.RAW
Database : Flagellin_v2 (192 sequences; 89,845 residues)
Taxonomy : Bacteria (Eubacteria) (192 sequences)
Timestamp : 8 Jul 2011 at 15:46:16 GMT

Not what you expected? Try **the select summary**.

▸ **Search parameters**
▸ **Score distribution**
▸ **Legend**

Protein Family Summary

Significance threshold p< 0.05 Max. number of families AUTO
Ions score or expect cut-off 0 Dendrograms cut at 0

Protein families 1–2 (out of 2)

10 per page 1

▾**1**

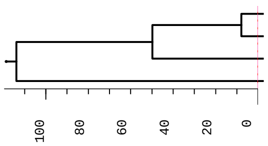

1	**gi|1655807**	5773	Hxx(H7 99.8%) 0|0|E32511
4	**gi|118403359**	438	H40 0|H40|H511
3	**gi|30577587**	1186	H17 0|O15:H17|P12b
2	**gi|121144335**	1934	H55 0|O75:H55|E2987-73

Threshold (**0**): 0

		Score	Mass	Matches	Sequences	emPAI
**1.1**	**gi|1655807** Hxx(H7 99.8%) 0|0|E32511 ▸ **2 samesets of gi|1655807**	5773	59918	138 (118)	56 (53)	53.95
**1.2**	**gi|121144335** H55 0|O75:H55|E2987-73	1934	62285	60 (44)	22 (17)	2.81
**1.3**	**gi|30577587** H17 0|O15:H17|P12b	1186	36285	38 (29)	15 (12)	4.74
**1.4**	**gi|118403359** H40 0|H40|H511 ▸ **1 sameset of gi|118403359**	438	51630	16 (11)	9 (6)	0.64

▾**152 peptide matches (91 non-duplicate, 61 duplicate)**

Query	Dupes	Observed	Mr(expt)	Mr(calc)	Delta	M	Score	Expect	Rank	U	1	2	3	4	Peptide
14	▸2	316.6893	631.3640	631.3653	-0.0013	0	33	0.0045	▸1		■	■	■	■	R.LSSGLR.I
61	▸1	355.1973	708.3800	708.3806	-0.0006	0	14	0.25	▸1		■	■	■		R.FTSNIK.G
62		355.1975	708.3804	708.3806	-0.0002	0	2	4.1	▸2	U				■	K.FTINSK.A
64		358.7059	715.3972	715.3977	-0.0004	0	29	0.0086	▸1		■	■	■		K.GLTQAAR.N
71		363.6686	725.3226	725.3232	-0.0005	0	22	0.0067	▸1	U	■				K.SYVDDK.G
89	▸3	380.6950	759.3754	759.3763	-0.0008	0	32	0.0038	▸1		■	■	■		R.LDEIDR.V
222	▸1	428.7050	855.3954	855.3974	-0.0020	0	64	4.3e-07	▸1	U	■				K.SGDFTTTK.S
230		430.2472	858.4798	859.4399	-0.9601	0	1	0.83	▸1	U		■			K.AQDVNVSK.D
275	▸1	445.7444	889.4742	889.4757	-0.0014	0	34	0.0015	▸1	U	■				K.DVVLSETK.A
354	▸2	473.2581	944.5016	944.5039	-0.0023	0	73	1.4e-07	▸1	U	■				R.SSLGAIQNR.L
359		474.2545	946.4944	946.4971	-0.0027	0	21	0.0083	▸1	U				■	K.SIDATELAK.L
374	▸1	478.7362	955.4578	955.4611	-0.0032	0	47	2.2e-05	▸1	U	■				K.SVNGSYTTK.D
379		480.2234	958.4322	958.4832	-0.0510	0	6	0.49	▸1	U				■	R.SDLGAVQNR.F
473	▸1	502.2609	1002.5072	1002.5094	-0.0022	1	35	0.0019	▸1		■	■	■		K.SRLDEIDR.V
474		335.1772	1002.5098	1002.5094	0.0004	1	30	0.0053	▸1		■	■	■		K.SRLDEIDR.V
666	▸1	551.2663	1100.5180	1100.5210	-0.0030	0	65	3e-06	▸1		■	■	■	■	K.DDAAGQAIANR.F
748		573.3344	1144.6542	1144.6564	-0.0021	1	34	0.0031	▸1		■	■	■	■	R.LSSGLRINSAK.D
834		596.3008	1190.5870	1190.5891	-0.0020	0	53	2.6e-05	▸1	U	■				K.NQSALSSSIER.L
836	▸1	596.3133	1190.6120	1190.6143	-0.0022	0	77	1.2e-07	▸1	U	■				K.AATVSDLTSAGAK.L
914		619.8342	1237.6538	1237.6554	-0.0016	0	62	5.9e-07	▸1	U	■				K.LNTTTGLYDLK.T
914		619.8342	1237.6538	1238.5561	-0.9022	0	4	0.44	▸2	U		■			K.NQSSMSTAIER.L + Oxidation (M)
1003	▸5	641.3433	1280.6720	1280.6725	-0.0004	0	77	3.5e-08	▸1	U	■				R.DALAATLHADVGK.S
1005	▸1	427.8983	1280.6731	1280.6725	0.0006	0	23	0.0084	▸1	U	■				R.DALAATLHADVGK.S
1109		672.8766	1343.7386	1343.7408	-0.0022	0	74	3.9e-08	▸1	U		■			-.SLSLITQNNINK.N
1120	▸3	677.8253	1353.6360	1353.6412	-0.0052	0	91	8.6e-10	▸1	U	■				K.TIGFTAGESSDAAK.S
1131		683.3217	1364.6288	1364.6783	-0.0495	0	0	0.94	▸1	U		■			K.GSVSNTAATTDTLK.L
1158		693.8482	1385.6818	1385.6827	-0.0009	0	82	7.5e-09	▸1	U	■				K.VTVGGVDYTYNAK.S
1180		469.5950	1405.7632	1405.7677	-0.0046	1	53	4.3e-05	▸1		■	■	■		R.FTSNIKGLTQAAR.N
1180		469.5950	1405.7632	1405.7677	-0.0046	1	1	7.3	▸2	U				■	R.FTANIKGLTQASR.N
1181	▸1	703.8890	1405.7634	1405.7677	-0.0043	1	82	5.5e-08	▸1		■	■	■		R.FTSNIKGLTQAAR.N
1224		479.9304	1436.7694	1436.7736	-0.0042	1	20	0.012	▸1	U	■				K.RDALAATLHADVGK.S
1229		480.9430	1439.8072	1439.8096	-0.0024	0	45	0.00014	▸1		■	■			K.AQIIQQAGNSVLAK.A

Query	Dupes	Observed	Mr(expt)	Mr(calc)	Delta	M	Score	Expect	Rank	U	1	2	3	4	Peptide
1230	▶4	720.9111	1439.8076	1439.8096	-0.0020	0	106	1.1e-10	▶1		■	■			K.AQIIQQAGNSVLAK.A
1284		747.9173	1493.8200	1493.8202	-0.0001	0	30	0.0064	▶1		■		■		K.ANQVPQQVLSLLQG.-
1285		498.9475	1493.8207	1493.8202	0.0005	0	39	0.00072	▶1		■		■		K.ANQVPQQVLSLLQG.-
1338		770.3796	1538.7446	1538.7464	-0.0018	0	85	4.2e-09	▶1	U	■				K.TENTLLTTDAAFDK.L
1360	▶3	781.4188	1560.8230	1560.8260	-0.0030	0	70	4.5e-07	▶1		■	■			R.VSGQTQFNGVNVLAK.D
1404		807.9125	1613.8104	1613.8121	-0.0017	1	104	3.9e-10	▶1		■	■	■	■	R.INSAKDDAAGQAIANR.F
1405	▶1	538.9442	1613.8108	1613.8121	-0.0013	1	49	0.00012	▶1		■	■	■	■	R.INSAKDDAAGQAIANR.F
1414		542.2883	1623.8431	1623.8468	-0.0037	0	21	0.0095	▶1	U	■				K.AAAATSSITFNSGVLSK.T
1415	▶1	812.9297	1623.8448	1623.8468	-0.0019	0	91	8.9e-10	▶1	U	■				K.AAAATSSITFNSGVLSK.T
1434		824.4199	1646.8252	1646.8264	-0.0011	0	109	1.2e-11	▶1	U	■				K.DYAPAIGTAVNVNSAGK.I
1447	▶1	832.4166	1662.8186	1662.8213	-0.0027	0	72	1.1e-07	▶1	U	■				K.IDSDTLGLNGFNVNGK.G
1455	▶1	836.3796	1670.7446	1670.7457	-0.0011	0	124	2.2e-12	▶1		■	■	■		R.IQDADYATEVSNMSK.A
1456		557.9225	1670.7457	1670.7457	-0.0001	0	47	0.00011	▶1		■	■	■		R.IQDADYATEVSNMSK.A
1465	▶1	839.8961	1677.7776	1677.7806	-0.0029	0	116	3.5e-12	▶1	U	■				K.STAGTGVDAAAQAADSASK.R
1466		560.2668	1677.7786	1677.7806	-0.0020	0	59	1.9e-06	▶1	U	■				K.STAGTGVDAAAQAADSASK.R
1478	▶8	843.9485	1685.8824	1685.8836	-0.0011	0	105	3.4e-10	▶1		■	■			K.IQVGANDGETITIDLK.K
1482	▶1	844.3751	1686.7356	1686.7407	-0.0050	0	90	8.1e-09	▶1		■	■	■		R.IQDADYATEVSNMSK.A + Oxidation (M)
1576	▶1	885.9088	1769.8030	1769.8068	-0.0037	0	84	3.6e-09	▶1	U	■				K.DNGSVTVAGYASATDTNK.D
1598	▶2	894.4431	1786.8716	1786.8737	-0.0021	0	118	2e-12	▶1	U	■				K.GGITNVADYTVSYSVNK.D
1603	▶1	896.4630	1790.9114	1790.9163	-0.0048	1	80	8.2e-08	▶1	U	■				K.KIDSDTLGLNGFNVNGK.G
1604	▶1	597.9793	1790.9161	1790.9163	-0.0002	1	51	6.5e-05	▶1	U	■				K.KIDSDTLGLNGFNVNGK.G
1619		902.4483	1802.8820	1803.9438	-1.0618	1	3	2.5	▶1	U	■				K.NQSALSSSIERLSSGLR.I
1624		602.6567	1804.9483	1804.9530	-0.0048	1	49	6.2e-05	▶1	U	■				K.GTITNKAATVSDLTSAGAK.L
1630		605.6676	1813.9810	1813.9785	0.0024	1	4	1.8	▶1		■	■			K.IQVGANDGETITIDLKK.I
1651		612.3002	1833.8788	1833.8817	-0.0029	1	70	1e-07	▶1	U	■				K.STAGTGVDAAAQAADSASKR.D
1652		917.9468	1833.8790	1833.8817	-0.0026	1	136	2.3e-14	▶1	U	■				K.STAGTGVDAAAQAADSASKR.D
1729		985.9929	1969.9712	1969.9745	-0.0033	1	100	1.1e-10	▶1	U	■				K.LGNGDKVTVGGVDYTYNAK.S
1731	▶1	657.9926	1970.9560	1969.9745	0.9814	1	50	9.9e-06	▶1	U	■				K.LGNGDKVTVGGVDYTYNAK.S
1769		695.7141	2084.1205	2085.0814	-0.9609	0	63	3e-06	▶1	U			■		M.AQVINTNSLSLNTQNNINK.N
1769		695.7141	2084.1205	2084.1225	-0.0021	0	62	3.9e-06	▶2	U	■			■	M.AQVINTNSLSLiTQNNLNK.N
1770	▶1	1043.0680	2084.1214	2084.1225	-0.0011	0	127	1.3e-12	▶1	U	■			■	M.AQVINTNSLSLiTQNNLNK.N
1770	▶1	1043.0680	2084.1214	2085.0814	-0.9600	0	85	2e-08	▶4	U			■		M.AQVINTNSLSLNTQNNINK.N
1779		1062.5250	2123.0354	2123.0382	-0.0028	1	83	5.1e-09	▶1	U	■				K.TENTLLTTDAAFDKLGNGDK.V
1780	▶1	708.6862	2123.0368	2123.0382	-0.0015	1	54	4.2e-06	▶1	U	■				K.TENTLLTTDAAFDKLGNGDK.V
1799	▶1	1095.5120	2189.0094	2189.0124	-0.0030	0	117	2.2e-12	▶1	U	■				K.AASEGSDGASLTFNGTEYTIAK.A
1835	▶2	1125.0530	2248.0914	2248.0931	-0.0017	0	124	2.2e-12	▶1		■	■	■		R.LDSAVTNLNNTTTNLSEAQSR.I
1836		750.3716	2248.0930	2248.0931	-0.0001	0	81	5.1e-08	▶1		■	■	■		R.LDSAVTNLNNTTTNLSEAQSR.I
1862		1152.1020	2302.1894	2302.1917	-0.0023	1	76	1.2e-07	▶1		■	■			R.LDEIDRVSGQTQFNGVNVLAK.D
1863		768.4039	2302.1899	2302.1917	-0.0019	1	62	2.9e-06	▶1		■	■			R.LDEIDRVSGQTQFNGVNVLAK.D
1898		819.1190	2454.3352	2454.3370	-0.0018	0	38	0.00015	▶1	U	■				K.ATPATTTPVAPLIPGGITYQATVSK.D
1899		1228.1750	2454.3354	2454.3370	-0.0016	0	62	6.7e-07	▶1	U	■				K.ATPATTTPVAPLIPGGITYQATVSK.D
1904		832.4022	2494.1848	2494.1864	-0.0016	1	41	0.00011	▶1	U	■				K.SYVDDKGGITNVADYTVSYSVNK.D
1905		1248.1000	2494.1854	2494.1864	-0.0009	1	90	1.5e-09	▶1	U	■				K.SYVDDKGGITNVADYTVSYSVNK.D
1922		860.7426	2579.2060	2579.2086	-0.0026	0	37	0.0002	▶1	U	■				R.ELTVQATTGTNSDSDLDSIQDEIK.S
1924	▶2	1290.6110	2579.2074	2579.2086	-0.0012	0	141	8e-15	▶1	U	■				R.ELTVQATTGTNSDSDLDSIQDEIK.S
1932		1315.1450	2628.2754	2628.2739	0.0015	0	146	1.1e-14	▶1		■	■			R.NANDGISVAQTTEGALSEINNNLQR.I
1933		877.0992	2628.2758	2628.2739	0.0019	0	69	5.6e-07	▶1		■	■			R.NANDGISVAQTTEGALSEINNNLQR.I
1976		1382.6910	2763.3674	2763.3662	0.0013	0	114	5.5e-12	▶1	U	■				K.ITTETTSAGSATTNPLAALDDAISSIDK.F
1978		925.1425	2772.4057	2772.3640	0.0417	1	0	0.93	▶1	U			■		K.GFTVSGMADFSAAKLTAADGTAIAAADVK.D + Oxidation (M)
1991		941.7867	2822.3383	2822.3418	-0.0035	1	71	7.6e-08	▶1	U	■				R.ELTVQATTGTNSDSDLDSIQDEIKSR.L
1994		950.4708	2848.3906	2848.3938	-0.0032	1	74	5.7e-08	▶1	U	■				R.IRELTVQATTGTNSDSDLDSIQDEIK.S
2019		1031.8540	3092.5402	3092.5448	-0.0046	1	87	9.9e-09	▶1		■	■			R.IQDADYATEVSNMSKAQIIQQAGNSVLAK.A
2025		1037.5440	3109.6102	3108.5397	1.0705	1	3	0.92	▶1	U			■		R.IQDADYATEVSNMSKAQIIQQAGNSVLSK.A
2030		1059.2020	3174.5842	3174.5865	-0.0023	1	105	7e-11	▶1	U	■				R.SSLGAIQNRLDSAVTNLNNTTTNLSEAQSR.I
2031		794.6547	3174.5897	3174.5865	0.0032	1	6	0.56	▶1	U	■				R.SSLGAIQNRLDSAVTNLNNTTTNLSEAQSR.I
2037		815.1816	3256.6973	3256.7011	-0.0038	1	33	0.0019	▶1	U	■				M.AQVINTNSLSLITQNNLNKNQSALSSSIER.L
2039	▶1	1086.5740	3256.7002	3256.7011	-0.0009	1	140	3.6e-14	▶1	U	■				M.AQVINTNSLSLITQNNLNKNQSALSSSIER.L
2056		1109.6080	3325.8022	3325.8021	0.0001	1	52	3.4e-05	▶1	U	■				K.ATPATTTPVAPLIPGGITYQATVSKDVVLSETK.A
2092		1133.8820	3398.6242	3398.6226	0.0016	1	93	5e-10	▶1	U	■				K.DNGSVTVAGYASATDTNKDYAPAIGTAVNVNSAGK.I

▶ 63 subsets and intersections (165 subset proteins in total)

▶2 gi|112820172 13 H21 0|EHEC serogroup: O113:H21|0

10 per page 1

Not what you expected? Try **the select summary**.

Mascot: http://www.matrixscience.com/
