# Supplementary material for: MS-H: A Novel Proteomic Approach to Isolate and Type the E. coli H Antigen Using Membrane Filtration and Liquid Chromatography-Tandem Mass Spectrometry (LC-MS/MS)
Source: PLoS One. 2013 Feb 21;8(2):e57339. doi: 10.1371/journal.pone.0057339 (PMC3578835; doi:10.1371/journal.pone.0057339)
Supplement: Representative Peptide Data S1 — Peptide data are represented as the Mascot search results from all 53 serotypes, obtained under the Orbitrap platform in Table 4 with related E. coli reference strains. “U” denotes a unique peptide specific for each of the proteins 1.1, 1.2, and beyond. The number 1.1 (shown as 1 in the peptide list and phylogenetic tree) represents the protein which obtained the highest score and confidence value after a Mascot search. This protein, known as the first hit, was used to designate the MS-H type of the unknown flagellin. Related peptides 1.2 (2), 1.3 (3), etc. represented the second, third, etc. hits for MS-H typing analysis. (DOCX) [file pone.0057339.s009.docx › H8-E176.pdf]

MATRIX SCIENCE MASCOT Search Results

User :
E-mail :
Search title : Submitted from 20110714-H1-H11 by Mascot Daemon on VARIABLE
MS data file : C:\Documents and Settings\keding\Desktop\Raw data\20110714-H1-H11\20110714-010-E176MS1.RAW
Database : Flagellin_v2 (192 sequences; 89,845 residues)
Taxonomy : Bacteria (Eubacteria) (192 sequences)
Timestamp : 15 Jul 2011 at 17:37:14 GMT

Not what you expected? Try **the select summary**.

Search parameters
Score distribution
Legend

Protein Family Summary

Significance threshold p< 0.05 Max. number of families AUTO
Ions score or expect cut-off 0 Dendrograms cut at 0

Protein family 1 (out of 1)

10 per page 1

1

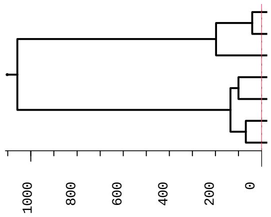

1	**gi|118403361**	5524	H8 0|O2:H8|AP 320c [gi|124516985|ref|CAI56315| H8 0|O111:H8|CB3466] [gi|124516987|ref...
2	**gi|99645039**	2956	H27 0|H27|0
4	**gi|46093545**	1099	H3 0|0|Bi7327-41
7	**gi|121144335**	632	H55 0|O75:H55|E2987-73
5	**gi|40994711**	892	H4 0|O154:H4|0
3	**gi|30059854**	1967	H6 0|H6|A20a
6	**gi|33590227**	653	H42 NM|O157:NM(H42)|25

Threshold (**0**): 0

		Score	Mass	Matches	Sequences	emPAI
1.1	**gi|118403361**	5524	51452	119 (107)	44 (43)	42.95
	H8 0|O2:H8|AP 320c [gi|124516985|ref|CAI56315| H8 0|O111:H8|CB3466] [gi|124516987|ref|CAI56316| H8 0|O111:H8|CB5778] [gi|323180826|ref|EFZ66366| Hxx 0|0|1180] [gi|257764871|ref|B...					
	**1 sameset of gi|118403361**					
1.2	**gi|99645039**	2956	50847	75 (60)	31 (26)	9.88
	H27 0|H27|0					
1.3	**gi|30059854**	1967	56213	48 (39)	27 (23)	3.15
	H6 0|H6|A20a					
1.4	**gi|46093545**	1099	55534	34 (20)	19 (10)	1.12
	H3 0|0|Bi7327-41					
1.5	**gi|40994711**	892	36224	23 (18)	14 (12)	2.41
	H4 0|O154:H4|0					
1.6	**gi|33590227**	653	44094	22 (17)	14 (10)	1.38
	H42 NM|O157:NM(H42)|25					
1.7	**gi|121144335**	632	62285	25 (18)	12 (10)	0.76
	H55 0|O75:H55|E2987-73					

194 peptide matches (111 non-duplicate, 83 duplicate)

Query	Dupes	Observed	Mr(expt)	Mr(calc)	Delta	M	Score	Expect	Rank	U	1	2	3	4	5	6	7	Peptide
3	1	301.1999	600.3852	601.3911	-1.0059	1	4	0.44	5	U				■				K.TVVRK.D
11	1	308.6745	615.3344	615.3340	0.0004	0	10	0.18	1		■	■						K.VDNLR.S
13		308.7057	615.3968	615.3591	0.0377	0	10	0.17	1	U				■				K.NLEIK.Q
23	2	316.6904	631.3662	631.3653	0.0009	0	27	0.018	1		■	■	■	■	■	■	■	R.LSSGLR.I
43		330.2082	658.4018	658.4014	0.0005	0	3	0.52	1	U		■						K.AAVSLAK.D
70	1	347.2006	692.3866	692.3857	0.0009	0	24	0.011	1		■	■						R.FTANIK.G
76		348.6818	695.3490	695.3490	0.0001	0	44	3.7e-05	1	U	■							K.GSIEYK.G
85		352.2030	702.3914	703.3752	-0.9838	0	5	0.51	1	U						■		K.DDTLIK.V
93		355.1978	708.3810	708.3806	0.0004	0	0	1.5	1				■	■	■	■	■	R.FTSNIK.G
97		358.7062	715.3978	715.3977	0.0002	0	11	0.52	1				■		■	■	■	K.GLTQAAR.N
110		366.6986	731.3826	731.3813	0.0013	0	9	0.48	1	U				■				R.LSEIDR.V
112	2	366.7040	731.3934	731.3926	0.0009	0	34	0.0014	1		■	■						K.GLTQASR.N
118		370.7047	739.3948	739.3938	0.0010	0	33	0.00075	1	U	■							K.VMYLSK.S
124	2	378.7020	755.3894	755.3887	0.0007	0	37	0.00025	1	U	■							K.VMYLSK.S + Oxidation (M)
137		387.7036	773.3926	773.3919	0.0007	0	33	0.0013	1		■	■						R.LEEIDR.V
320	1	446.2423	890.4700	890.4709	-0.0009	0	48	5.4e-05	1		■	■						K.ATGSDLISK.F
371	2	466.2510	930.4874	930.4883	-0.0008	0	58	7.3e-06	1				■	■	■		■	R.SSLGAVQNR
395	1	473.2794	944.5442	945.4403	-0.8961	0	3	1.5	1	U			■					K.DQDVNIDK.S
427	1	480.2485	958.4824	958.4832	-0.0008	0	68	2.8e-07	1		■	■						R.SDLGAVQNR.F
427	1	480.2485	958.4824	958.5196	-0.0371	0	30	0.0019	2	U						■		R.SSLGVVQNR.L
538		524.2601	1046.5056	1046.5066	-0.0010	0	35	0.00059	1		■	■						K.VLAENNEMK.I
549	1	527.7617	1053.5088	1053.5091	-0.0002	0	51	1.3e-05	1		■	■						K.NSAGQFTTTK.V

Query	Dupes	Observed	Mr(expt)	Mr(calc)	Delta	M	Score	Expect	Rank	U	1	2	3	4	5	6	7	Peptide
559		532.2578	1062.5010	1062.5015	-0.0005	0	39	0.00027	▶1		■	■						K.VLAENNEMK.I + Oxidation (M)
575		538.7818	1075.5490	1075.5509	-0.0019	0	29	0.0017	▶1	U			■					K.GETANTAATLK.D
597	▶1	544.2843	1086.5540	1086.5557	-0.0017	0	65	3e-07	▶1	U	■							K.TVTDTTPGAPK.V
623	▶1	551.2673	1100.5200	1100.5210	-0.0010	0	72	6e-07	▶1		■	■	■	■	■	■	■	K.DDAAGQAIANR.F
650		562.3024	1122.5902	1122.5921	-0.0018	1	42	6.4e-05	▶1	U	■							K.LADKGSIEYK.G
651		375.2050	1122.5932	1122.5921	0.0011	1	32	0.00071	▶1	U	■							K.LADKGSIEYK.G
693		570.7796	1139.5446	1139.5459	-0.0012	0	29	0.0013	▶1	U			■					K.NDTYTATVGAK.T
731	▶1	582.7958	1163.5770	1163.5782	-0.0012	0	66	7.6e-07	▶1		■			■				K.SQSSLSSAIER.L
737		390.1807	1167.5203	1166.5819	0.9383	0	4	0.4	▶1	U		■						K.DVTFTIDATGK.D
773		596.3020	1190.5894	1190.5891	0.0004	0	49	6.5e-05	▶1			■	■					K.NQSALSSSIER.L
778	▶1	599.2724	1196.5302	1196.5310	-0.0007	0	69	1.1e-07	▶1	U	■							K.ATGTDNYDVGGK.T
782		600.8536	1199.6926	1199.6734	0.0192	1	4	0.43	▶1	U				■				K.LRSSLGAVQNR.F
836	▶2	618.8144	1235.6142	1235.6146	-0.0004	0	70	2.4e-07	▶1		■	■						R.VSEQTQFNGVK.V
836		618.8144	1235.6142	1234.6306	0.9836	0	45	7.6e-05	▶2	U				■				R.VSQQTQFNGVK.V
891		630.8121	1259.6096	1259.6106	-0.0009	0	44	3.8e-05	▶1	U			■					K.NNAGGDTQATLAK.L
899	▶1	634.3287	1266.6428	1266.6456	-0.0027	0	62	6.6e-07	▶1	U	■							K.TYTVNVESGAVK.N
964	▶3	651.8489	1301.6832	1301.6827	0.0006	0	89	3.1e-09	▶1	U	■							K.AATLSDLDLNAAK.K
966	▶1	652.3965	1302.7784	1302.6415	0.1369	0	9	0.23	▶1	U		■						K.AATASDLDLNNAK.K
996		660.3378	1318.6610	1318.6616	-0.0006	0	115	3.5e-12	▶1	U	■							K.VSGESIDATELAK.L
1025		672.8776	1343.7406	1343.7408	-0.0002	0	64	4e-07	▶1	U							■	-.SLSLITQNNINK.N
1073		694.3751	1386.7356	1386.7354	0.0002	0	49	1.6e-05	▶1	U					■			K.LTAADGTAIAAADVK.D
1075		694.8303	1387.6460	1387.6442	0.0018	0	97	2e-10	▶1	U					■			K.GFTVSGMADFSAAK.L
1119		715.8964	1429.7782	1429.7776	0.0006	1	51	1.3e-05	▶1	U	■							K.AATLSDLDLNAAKK.T
1120		477.6001	1429.7785	1429.7776	0.0009	1	15	0.046	▶1	U	■							K.AATLSDLDLNAAKK.T
1130		720.9125	1439.8104	1439.8096	0.0008	0	60	4.2e-06	▶1				■		■	■	■	K.AQIIQQAGNSVLAK.A
1137		724.8735	1447.7324	1447.7307	0.0017	0	87	4.1e-09	▶1		■	■						K.TLGLDGFNIDGAQK.A
1170	▶1	491.5716	1471.6930	1471.6943	-0.0014	1	29	0.0012	▶1	U	■							K.FKATGTDNYDVGGK.T
1171		736.8543	1471.6940	1471.6943	-0.0003	1	76	2.7e-08	▶1	U	■							K.FKATGTDNYDVGGK.T
1189	▶1	743.8721	1485.7296	1485.7311	-0.0014	0	84	6.6e-09	▶1	U	■							K.SEGGSPILVNEDAAK.S
1201		747.9191	1493.8236	1493.8202	0.0035	0	26	0.015	▶1				■		■	■		K.ANQVPQQVLSLLQG.-
1244		762.8862	1523.7578	1523.7580	-0.0001	1	76	4.8e-08	▶1		■	■						K.NSAGQFTTTKVENK.A
1245		508.9270	1523.7592	1523.7580	0.0012	1	27	0.0032	▶1		■	■						K.NSAGQFTTTKVENK.A
1265	▶1	773.9019	1545.7892	1545.7886	0.0006	0	78	2.9e-08	▶1		■	■						K.SLQSTTNPLETIDK.A
1282		781.4209	1560.8272	1560.8260	0.0012	0	42	0.00027	▶1				■			■	■	R.VSGQTQFNGVNVLAK
1346		538.9437	1613.8093	1613.8121	-0.0028	1	31	0.0064	▶1		■	■	■	■	■	■	■	R.INSAKDDAAGQAIANR.F
1347		807.9136	1613.8126	1613.8121	0.0005	1	99	1.2e-09	▶1		■	■	■	■	■	■	■	R.INSAKDDAAGQAIANR.F
1401		836.3813	1670.7480	1670.7457	0.0023	0	101	5.3e-10	▶1				■		■	■	■	R.IQDADYATEVSNMSK.A
1424	▶9	843.4571	1684.8996	1684.8996	0.0001	0	116	9.8e-12	▶1	U			■					K.IQVGANDGQTITIDLK.K
1429	▶7	843.4587	1684.9028	1685.8836	-0.9807	0	44	0.00017	▶2	U							■	K.IQVGANDGETITIDLK.K
1445	▶1	850.8763	1699.7380	1699.7359	0.0021	0	134	7.1e-14	▶1		■	■						R.IEDADYATEVSNMSR.A
1465	▶2	858.8707	1715.7268	1715.7308	-0.0040	0	121	1.6e-12	▶1		■	■						R.IEDADYATEVSNMSR.A + Oxidation (M)
1465		858.8707	1715.7268	1715.7308	-0.0040	0	8	0.33	▶2	U				■				R.IEDSDYATEVSNMSR.A
1480	▶9	861.9186	1721.8226	1721.8220	0.0006	0	126	2.3e-13	▶1	U	■							K.GITFTNNTGAELDANGK.G
1530	▶5	878.9759	1755.9372	1755.9367	0.0006	0	91	1.8e-09	▶1		■	■						K.IQVGANDGETITINLAK.I
1548		885.9649	1769.9152	1769.9159	-0.0007	0	98	1.8e-10	▶1	U				■				K.IQVGANDGQTIEIGLDK.I
1589	▶2	900.9286	1799.8426	1799.8425	0.0001	0	137	3.8e-14	▶1	U	■							K.DVFVSAADGSLTTSSDTK.V
1596		601.9706	1802.8900	1803.9438	-1.0539	1	0	4.4	▶1			■	■					K.NQSALSSSIERLSSGLR.I
1645	▶3	927.4673	1852.9200	1852.9167	0.0034	0	103	1e-10	▶1	U	■							K.TGSTLVVNGATYNVSADGK.T
1655		929.9804	1857.9462	1857.9432	0.0030	0	95	3.1e-10	▶1	U			■					K.SAVASSVDILNAVAGADGNK.V
1721		644.0220	1929.0442	1929.0418	0.0023	1	44	9.2e-05	▶1		■	■						K.SLQSTTNPLETIDKALAK.V
1722		965.5301	1929.0456	1929.0418	0.0038	1	34	0.00091	▶1		■	■						K.SLQSTTNPLETIDKALAK.V
1733	▶1	648.6310	1942.8712	1942.8690	0.0021	1	58	2.5e-06	▶1		■	■						R.SRIEDADYATEVSNMSR.A
1734		972.4429	1942.8712	1942.8690	0.0022	1	65	5.1e-07	▶1		■	■						R.SRIEDADYATEVSNMSR.A
1742	▶2	653.9619	1958.8639	1958.8640	-0.0001	1	55	7.2e-06	▶1		■	■						R.SRIEDADYATEVSNMSR.A + Oxidation (M)
1743		980.4399	1958.8652	1958.8640	0.0013	1	62	1.4e-06	▶1		■	■						R.SRIEDADYATEVSNMSR.A + Oxidation (M)
1743		980.4399	1958.8652	1958.8640	0.0013	1	3	1.1	▶2	U				■				R.SRIEDSDYATEVSNMSR.A
1758		991.5128	1981.0110	1981.0116	-0.0006	1	120	1.7e-12	▶1	U	■							K.KTGSTLVVNGATYNVSADGK.T
1765	▶1	664.6727	1990.9963	1990.9960	0.0003	1	47	3.1e-05	▶1		■	■						R.LEEIDRVSEQTQFNGVK.V
1766		996.5057	1990.9968	1990.9960	0.0009	1	82	1e-08	▶1		■	■						R.LEEIDRVSEQTQFNGVK.V
1772	▶2	997.5021	1992.9896	1992.9865	0.0032	0	149	3.4e-15	▶1		■	■		■				R.FDSAITNLGNTVNNLSSAR.S
1773		665.3374	1992.9904	1992.9865	0.0039	0	56	6.8e-06	▶1		■	■		■				R.FDSAITNLGNTVNNLSSAR.S
1775		499.4695	1993.8489	1992.9865	0.8624	0	11	0.18	▶1		■	■		■				R.FDSAITNLGNTVNNLSSAR.S
1826	▶1	1043.0700	2084.1254	2084.1225	0.0029	0	127	1.3e-12	▶1		■	■	■	■	■			M.AQVINTNSLSLiTQNNiNK.N
1826	▶1	1043.0700	2084.1254	2085.1066	-0.9811	0	81	5.4e-08	▶4	U						■		M.AQVINTNSLSLITQNNIDK.N
1827	▶1	695.7162	2084.1268	2084.1225	0.0042	0	71	4.8e-07	▶1		■	■	■	■	■			M.AQVINTNSLSLiTQNNiNK.N
1827	▶1	695.7162	2084.1268	2085.1066	-0.9798	0	67	1.3e-06	▶5	U						■		M.AQVINTNSLSLITQNNIDK.N
1875		734.6655	2200.9747	2202.0627	-1.0880	1	3	0.49	▶1	U				■				K.GTVGKALSFNDSQMSVYVDGK.N
1897		1117.5550	2233.0954	2233.0903	0.0051	0	87	2.8e-09	▶1	U			■					K.VTTSADVGFGTPAAAVTYTYNK.D
1906		1123.0280	2244.0414	2244.0369	0.0046	0	71	8.8e-08	▶1	U			■					K.DMSGFTAAAAPGGTVGVTQYTDK.S
1909		1125.0560	2248.0974	2248.0931	0.0043	0	130	6.3e-13	▶1				■		■	■	■	R.LDSAVTNLNNTTTNLSEAQSR.I
1927		1149.5520	2297.0894	2297.0845	0.0049	0	99	1.3e-10	▶1	U			■					K.SGNLTAADDGAVLYMDATGNLTK.N
1928		766.7326	2297.1760	2297.0845	0.0914	0	4	0.38	▶1	U			■					K.SGNLTAADDGAVLYMDATGNLTK.N
1942		781.7037	2342.0893	2342.0874	0.0019	1	93	5.6e-10	▶1	U	■							K.NDANKDVFVSAADGSLTTSSDTK.V
1944	▶1	1172.0530	2342.0914	2342.0874	0.0041	1	80	1e-08	▶1	U	■							K.NDANKDVFVSAADGSLTTSSDTK.V
1987	▶1	1283.6050	2565.1954	2565.1930	0.0025	0	103	1.5e-10	▶1	U			■					R.ELTVQASTGTNSDSDLDSIQDEIK.S
1997	▶1	1328.6520	2655.2894	2655.2848	0.0046	0	142	1.7e-14	▶1		■	■						R.NANDGISVAQTTEGALNEINNNLQR
1998		886.1039	2655.2899	2655.2848	0.0051	0	76	6.8e-08	▶1		■	■						R.NANDGISVAQTTEGALNEINNNLQR
2012		899.8161	2696.4265	2696.4232	0.0032	1	1	0.85	▶1	U				■				K.IQVGANDGQTIEIGLDKIDADTLGLK.D
2022		1363.6190	2725.2234	2725.2178	0.0057	0	122	6.5e-13	▶1	U			■					K.GTFTSDGTAFDGASMSIDTNTFANAVK.N
2028		922.1136	2763.3190	2763.3159	0.0031	0	67	3e-07	▶1	U	■							R.ELSVQATNGTNSDSDLSSIQAEITQR.L
2029		1382.6670	2763.3194	2763.3159	0.0036	0	123	6.7e-13	▶1	U	■							R.ELSVQATNGTNSDSDLSSIQAEITQR.L
2032		933.5038	2797.4896	2797.4821	0.0074	0	22	0.007	▶1	U					■			K.IQIGANDNQTISIGLQQIDSTTLNLK.G

Query	Dupes	Observed	Mr(expt)	Mr(calc)	Delta	M	Score	Expect	Rank	U	1	2	3	4	5	6	7	Peptide
2042		945.8035	2834.3887	2834.3781	0.0105	1	75	1.1e-07	1	U			■					R.IRELTVQASTGTNSDSDLDSIQDEIK.S
2045		956.2011	2865.5815	2865.5672	0.0143	0	55	3.3e-06	1		■	■		■				R.AQILQQAGTSVLAQANQTTQNVLSLLR.-
2058		978.8298	2933.4676	2933.4591	0.0085	1	54	6e-06	1		■	■						R.SDLGAVQNRFDSAITNLGNTVNNLSSAR.S
2064		1011.8430	3032.5072	3032.5010	0.0062	1	71	1.3e-07	1	U	■							R.IRELSVQATNGTNSDSDLSSIQAEITQR.L
2064		1011.8430	3032.5072	3032.5010	0.0061	1	59	2.1e-06	2	U		■						R.VRELTVQATNGTNSDSDLSSIQAEITQR.L
2113		1125.9430	3374.8072	3374.7570	0.0502	1	1	0.72	1	U						■		K.IDSSALGLSGFSVAGGALKLSDTVTQVGDGSAAPVK.V
2133		1185.5610	3553.6612	3553.6519	0.0093	1	32	0.00066	1	U			■					K.AATIQTDKGTFTSDGTAFDGASMSIDTNTFANAVK.N

89 subsets and intersections (165 subset proteins in total)

10 per page 1

Not what you expected? Try **the select summary**.

Mascot: http://www.matrixscience.com/
