# Supplementary material for: MS-H: A Novel Proteomic Approach to Isolate and Type the E. coli H Antigen Using Membrane Filtration and Liquid Chromatography-Tandem Mass Spectrometry (LC-MS/MS)
Source: PLoS One. 2013 Feb 21;8(2):e57339. doi: 10.1371/journal.pone.0057339 (PMC3578835; doi:10.1371/journal.pone.0057339)
Supplement: Representative Peptide Data S1 — Peptide data are represented as the Mascot search results from all 53 serotypes, obtained under the Orbitrap platform in Table 4 with related E. coli reference strains. “U” denotes a unique peptide specific for each of the proteins 1.1, 1.2, and beyond. The number 1.1 (shown as 1 in the peptide list and phylogenetic tree) represents the protein which obtained the highest score and confidence value after a Mascot search. This protein, known as the first hit, was used to designate the MS-H type of the unknown flagellin. Related peptides 1.2 (2), 1.3 (3), etc. represented the second, third, etc. hits for MS-H typing analysis. (DOCX) [file pone.0057339.s009.docx › H9-E177.pdf]

MATRIX SCIENCE MASCOT Search Results

User :
E-mail :
Search title : Submitted from 20110714-H1-H11 by Mascot Daemon on VARIABLE
MS data file : C:\Documents and Settings\keding\Desktop\Raw data\20110714-H1-H11\20110714-011-E177MS1.RAW
Database : Flagellin_v2 (192 sequences; 89,845 residues)
Taxonomy : Bacteria (Eubacteria) (192 sequences)
Timestamp : 15 Jul 2011 at 17:40:21 GMT

Not what you expected? Try **the select summary**.

▸ **Search parameters**
▸ **Score distribution**
▸ **Legend**

Protein Family Summary

Significance threshold p< 0.05 Max. number of families AUTO
Ions score or expect cut-off 0 Dendrograms cut at 0

Protein families 1–3 (out of 3)

10 per page 1

▾**1**

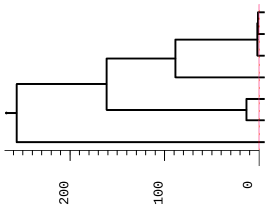

1	gi|3377710	10501	H9 0|0|O78:K80; EC341 95 [gi|30059860|ref|AAP13302| H9 0|H9|Bi 7575-41]
4	gi|40994711	1831	H4 0|O154:H4|0
5	gi|33590260	889	H29 0|O157:H29|Y63
7	gi|118403359	475	H40 0|H40|H511
2	gi|157081065	3507	Hxx(H28 99.7%) 0|0|E24377A [gi|157159035|ref|YP_001463228| Hxx 0|0|E24377A]
6	gi|99645039	649	H27 0|H27|0
3	gi|121144335	2348	H55 0|O75:H55|E2987-73

Threshold (**0**): 0

		Score	Mass	Matches	Sequences	emPAI
**1.1**	**gi|3377710**	10501	68093	176 (163)	52 (51)	23.67
	H9 0|0|O78:K80; EC341 95 [gi|30059860|ref|AAP13302| H9 0|H9|Bi 7575-41]					
**1.2**	**gi|157081065**	3507	59373	79 (67)	25 (23)	4.32
	Hxx(H28 99.7%) 0|0|E24377A [gi|157159035|ref|YP_001463228| Hxx 0|0|E24377A]					
	▸ **2 samesets of gi|157081065**					
**1.3**	**gi|121144335**	2348	62285	54 (43)	19 (16)	1.95
	H55 0|O75:H55|E2987-73					
**1.4**	**gi|40994711**	1831	36224	47 (37)	20 (16)	5.87
	H4 0|O154:H4|0					
	▸ **1 sameset of gi|40994711**					
**1.5**	**gi|33590260**	889	45720	28 (22)	12 (11)	1.84
	H29 0|O157:H29|Y63					
**1.6**	**gi|99645039**	649	50847	24 (15)	16 (8)	0.87
	H27 0|H27|0					
**1.7**	**gi|118403359**	475	51630	21 (11)	14 (6)	0.64
	H40 0|H40|H511					
	▸ **3 samesets of gi|118403359**					

▾**218 peptide matches (97 non-duplicate, 121 duplicate)**

Query	Dupes	Observed	Mr(expt)	Mr(calc)	Delta	M	Score	Expect	Rank	U	1	2	3	4	5	6	7	Peptide
13		305.1657	608.3168	609.2428	-0.9260	0	7	0.21	▸1	U				■				K.NDSMK.I + Oxidation (M)
28		310.1505	618.2864	618.2973	-0.0108	1	2	0.66	▸2	U							■	K.DKDNK.D
47	▸2	316.6898	631.3650	631.3653	-0.0003	0	35	0.0032	▸1		■	■	■	■	■	■	■	R.LSSGLR.I
54	▸1	318.6895	635.3644	635.3643	0.0002	0	32	0.00071	▸1	U	■							K.LAGFTK.G
81		330.2080	658.4014	658.4014	0.0001	0	3	0.54	▸2	U						■		K.AAVSLAK.D
84		330.6876	659.3606	660.3079	-0.9472	0	2	0.69	▸2	U			■					K.DVDNAK.A
85	▸1	330.6878	659.3610	659.3602	0.0008	0	16	0.025	▸1	U	■							K.QGNLTK.T
89		332.6758	663.3370	663.3704	-0.0334	1	5	0.33	▸1	U				■				K.VDKFR.S
100		338.6927	675.3708	674.3963	0.9745	0	11	0.085	▸1	U						■		K.TVTGLGK.T
155	▸1	355.1971	708.3796	708.3806	-0.0010	0	14	0.28	▸1		■	■	■	■	■			R.FTSNIK.G
156	▸1	355.1979	708.3812	708.3806	0.0006	0	6	1.5	▸2	U							■	K.FTINSK.A
163	▸2	358.7060	715.3974	715.3977	-0.0002	0	35	0.0023	▸1		■	■	■	■	■			K.GLTQAAR.N
207	▸2	380.6950	759.3754	759.3763	-0.0008	0	32	0.004	▸1		■	■	■	■	■			R.LDEIDR.V
225		386.7315	771.4484	771.4490	-0.0006	0	14	0.038	▸1	U				■				K.ALDAAIAK.V
376	▸2	430.7268	859.4390	859.4399	-0.0009	0	60	1e-06	▸1	U	■							K.VNISQDGK.I
493	▸1	466.2507	930.4868	930.4883	-0.0014	0	86	1e-08	▸1		■	■	■	■	■			R.SSLGAVQNR.L
589		493.3003	984.5860	984.5604	0.0257	0	8	0.15	▸1	U							■	K.LAINLADQK.S
622		502.2610	1002.5074	1002.5094	-0.0020	1	40	0.00054	▸1		■	■	■	■	■			K.SRLDEIDR.V
623		335.1772	1002.5098	1002.5094	0.0004	1	31	0.005	▸1		■	■	■	■	■			K.SRLDEIDR.V
664	▸1	515.2480	1028.4814	1028.4815	-0.0000	0	37	0.00022	▸1	U	■							K.DVTYFEQK.N
761		544.2930	1086.5714	1086.5557	0.0157	0	0	0.95	▸1	U							■	K.TVTDTTPGAPK.V

Query	Dupes	Observed	Mr(expt)	Mr(calc)	Delta	M	Score	Expect	Rank	U	1	2	3	4	5	6	7	Peptide
769	▶ 1	545.2455	1088.4764	1088.4774	-0.0010	0	53	4.9e-06	▶ 1	U	■							K.NGYTYDAASK.S
794	▶ 1	551.2666	1100.5186	1100.5210	-0.0024	0	70	1e-06	▶ 1		■	■	■	■	■	■	■	K.DDAAGQAIANR.F
872	▶ 1	581.3032	1160.5918	1160.5925	-0.0006	0	86	3.5e-09	▶ 1		■	■						K.ALDEAISSIDK.F
881		583.4245	1164.8344	1165.6343	-0.7998	1	6	1.2	▶ 1							■	■	K.ATGSDLISKFK.A
884		390.1805	1167.5197	1166.5819	0.9377	0	4	0.38	▶ 1	U						■		K.DVTFTIDATGK.D
913	▶ 1	596.3017	1190.5888	1190.5891	-0.0002	0	59	6.6e-06	▶ 1		■	■				■		K.NQSALSSSIER.L
953		609.7853	1217.5560	1217.5888	-0.0328	0	15	0.033	▶ 1	U					■			R.VTIDGDGTNQAK.I
1069	▶ 2	645.2852	1288.5558	1288.5571	-0.0013	0	73	5.1e-08	▶ 1	U	■							K.SYSFAADGADSAK.T
1150	▶ 1	672.8774	1343.7402	1343.7408	-0.0006	0	64	4.2e-07	▶ 1	U			■					-.SLSLITQNNINK.N
1155	▶ 1	675.3301	1348.6456	1348.6470	-0.0014	0	98	1.5e-10	▶ 1	U	■							K.GSVANTAATSDDLK.L
1204		694.3027	1386.5908	1387.6442	-1.0534	0	2	0.79	▶ 1	U				■				K.GFTVSGMADFSAAK.L
1279		717.0056	1431.9966	1430.7365	1.2602	1	2	0.6	▶ 1	U						■		K.AATASDLDLNNAKK.V
1287	▶ 3	720.9124	1439.8102	1439.8096	0.0006	0	103	2.5e-10	▶ 1		■	■	■	■				K.AQIIQQAGNSVLAK.A
1316	▶ 2	732.8882	1463.7618	1463.7620	-0.0001	1	80	1.5e-08	▶ 1		■	■						K.ALDEAISSIDKFR.S
1318	▶ 1	488.9283	1463.7631	1463.7620	0.0011	1	53	6.8e-06	▶ 1		■	■						K.ALDEAISSIDKFR.S
1354		746.3367	1490.6588	1490.7729	-0.1140	1	2	0.65	▶ 1	U			■					K.ALDDAISQIDKFR.S
1358	▶ 1	747.9187	1493.8228	1493.8202	0.0027	0	35	0.002	▶ 1		■	■		■	■			K.ANQVPQQVLSLLQG.-
1401	▶ 1	762.3831	1522.7516	1522.7515	0.0002	0	107	3.3e-11	▶ 1	U	■							K.ITAADDNATLYLDK.Q
1480	▶ 3	789.4170	1576.8194	1576.8209	-0.0015	0	94	4.8e-10	▶ 1		■			■				R.VSGQTQFNGVNVLSK
1518		807.9122	1613.8098	1613.8121	-0.0023	1	92	6.1e-09	▶ 1		■	■	■	■	■	■	■	R.INSAKDDAAGQAIANR.F
1519		538.9445	1613.8117	1613.8121	-0.0004	1	34	0.0037	▶ 1		■	■	■	■	■	■	■	R.INSAKDDAAGQAIANR.F
1595	▶ 1	836.3800	1670.7454	1670.7457	-0.0003	0	130	6.6e-13	▶ 1		■	■	■	■	■			R.IQDADYATEVSNMSK.A
1601	▶ 2	839.4275	1676.8404	1676.8370	0.0035	0	91	8.9e-10	▶ 1	U	■							K.IDSDTLNLAGFNVNGK.G
1618	▶ 10	843.9484	1685.8822	1684.8996	0.9827	0	44	0.00038	▶ 2	U		■						K.IQVGANDGQTITIDLK.K
1622	▶ 12	843.9499	1685.8852	1685.8836	0.0017	0	126	2.3e-12	▶ 1		■		■					K.IQVGANDGETITIDLK.K
1628		844.3772	1686.7398	1686.7407	-0.0008	0	111	6.3e-11	▶ 1		■	■	■	■	■			R.IQDADYATEVSNMSK.A + Oxidation (M)
1635	▶ 1	847.4264	1692.8382	1692.8418	-0.0035	0	126	2.7e-13	▶ 1	U	■							K.LTTEATTASSSTADPLK.A
1671		858.8907	1715.7668	1715.7308	0.0360	0	1	1.4	▶ 1							■	■	R.IEDADYATEVSNMSR.A + Oxidation (M)
1689		576.3044	1725.8914	1724.9019	0.9895	1	26	0.0023	▶ 1	U						■		K.TMYLSKSEGGSPILVK.E + Oxidation (M)
1729		878.8983	1755.7820	1755.9367	-0.1546	0	1	1.5	▶ 1							■	■	K.IQVGANDGETITINLAK.I
1773		359.9968	1794.9476	1795.8589	-0.9112	0	16	0.023	▶ 1	U		■						K.STGFTVDVGATGNSAGDIK.V
1780	▶ 1	899.6440	1797.2734	1795.8589	1.4146	0	7	0.22	▶ 1	U		■						K.STGFTVDVGATGNSAGDIK.V
1785		900.9287	1799.8428	1799.8425	0.0003	0	103	8.3e-11	▶ 1	U							■	K.DVFVSAADGSLTTSSDTK.V
1790	▶ 2	903.4723	1804.9300	1804.9319	-0.0019	1	121	4.6e-12	▶ 1	U	■							K.KIDSDTLNLAGFNVNGK.G
1791	▶ 2	602.6509	1804.9309	1804.9319	-0.0010	1	47	0.00012	▶ 1	U	■							K.KIDSDTLNLAGFNVNGK.G
1795		903.9775	1805.9404	1806.9476	-1.0071	1	14	0.046	▶ 2	U			■					K.KIDSSTLNLTGFNVNGK.G
1807		605.6668	1813.9786	1813.9785	0.0000	1	28	0.007	▶ 1		■		■					K.IQVGANDGETITIDLKK.I
1807		605.6668	1813.9786	1812.9945	0.9840	1	10	0.4	▶ 2	U		■						K.IQVGANDGQTITIDLKK.I
1882	▶ 14	935.4447	1868.8748	1868.8752	-0.0003	0	141	8.2e-15	▶ 1	U	■							K.NGAITNSTGGTIYETADGK.L
1954		643.6628	1927.9666	1929.0418	-1.0753	1	10	0.18	▶ 1							■	■	K.SLQSTTNPLETIDKALAK.V
1975	▶ 5	975.9741	1949.9336	1949.9330	0.0006	0	126	2.7e-13	▶ 1	U	■							K.AAANADVYVEDGALSANATK.D
2005		656.3401	1965.9985	1966.0007	-0.0023	1	35	0.00034	▶ 1	U	■							K.GSVANTAATSDDLKLAGFTK.G
2006		984.0072	1965.9998	1966.0007	-0.0009	1	107	2.1e-11	▶ 1	U	■							K.GSVANTAATSDDLKLAGFTK.G
2014	▶ 1	986.9584	1971.9022	1971.9022	0.0001	0	128	1.5e-13	▶ 1	U	■							K.GTTDTNGVTAYTNTISNDK.A
2027		499.4692	1993.8477	1992.9865	0.8612	0	16	0.06	▶ 1							■	■	R.FDSAITNLGNTVNNLSSAR.S
2091		695.7156	2084.1250	2084.1225	0.0024	0	62	3.9e-06	▶ 1		■	■		■	■	■	■	M.AQVINTNSLSLiTQNNiNK.N
2092	▶ 1	1043.0700	2084.1254	2084.1225	0.0029	0	114	2.6e-11	▶ 1		■	■		■	■	■	■	M.AQVINTNSLSLiTQNNiNK.N
2140		722.3742	2164.1008	2164.1012	-0.0004	1	61	7.6e-07	▶ 1	U	■							K.ITAADDNATLYLDKQGNLTK.T
2147	▶ 1	1086.0420	2170.0694	2170.0688	0.0006	0	98	1.8e-10	▶ 1	U	■							K.VDTVNVTNAHVSAEGMANLTK.S
2148	▶ 3	724.3641	2170.0705	2170.0688	0.0016	0	66	2.7e-07	▶ 1	U	■							K.VDTVNVTNAHVSAEGMANLTK.S
2151		724.6837	2171.0293	2171.0342	-0.0050	1	28	0.0015	▶ 1	U	■							K.GTTDTNGVTAYTNTISNDKAK.A
2152		1086.5230	2171.0314	2171.0342	-0.0028	1	100	9.4e-11	▶ 1	U	■							K.GTTDTNGVTAYTNTISNDKAK.A
2154	▶ 2	1087.5680	2173.1214	2173.1226	-0.0012	0	119	1.3e-12	▶ 1	U	■							K.TLSIINPNTGDSSQATVTIGGK.E
2162		729.6933	2186.0581	2186.0638	-0.0057	0	33	0.00051	▶ 1	U	■							K.VDTVNVTNAHVSAEGMANLTK.S + Oxidation (M)
2197	▶ 1	1125.0540	2248.0934	2248.0931	0.0003	0	138	9.9e-14	▶ 1		■	■	■	■				R.LDSAVTNLNNTTTNLSEAQSR.I
2198	▶ 1	750.3727	2248.0963	2248.0931	0.0032	0	66	1.5e-06	▶ 1		■	■	■	■				R.LDSAVTNLNNTTTNLSEAQSR.I
2228		773.7358	2318.1856	2318.1866	-0.0011	1	50	2e-05	▶ 1		■			■				R.LDEIDRVSGQTQFNGVNVLSK
2229		1160.1010	2318.1874	2318.1866	0.0008	1	82	1.2e-08	▶ 1		■			■				R.LDEIDRVSGQTQFNGVNVLSK
2243	▶ 7	1182.5570	2363.0994	2363.0952	0.0043	0	124	3.9e-13	▶ 1	U	■							K.SNFTIDMGGTGTVTYTVSNGDVK.A
2287	▶ 5	1245.6470	2489.2794	2489.2762	0.0033	0	152	6.4e-16	▶ 1	U	■							K.ASDLLANITDGSVITGGGANAFGVAAK.N
2288	▶ 1	830.7672	2489.2798	2489.2762	0.0036	0	91	7.4e-10	▶ 1	U	■							K.ASDLLANITDGSVITGGGANAFGVAAK.N
2309		853.7804	2558.3194	2558.3188	0.0006	1	28	0.0016	▶ 1	U	■							K.TLSIINPNTGDSSQATVTIGGKEQK.V
2315		856.0717	2565.1933	2565.1930	0.0003	0	57	5.6e-06	▶ 1		■	■						R.ELTVQASTGTNSDSDLDSIQDEIK.S
2321	▶ 7	1283.6050	2565.1954	2565.1930	0.0025	0	135	9.3e-14	▶ 1		■	■						R.ELTVQASTGTNSDSDLDSIQDEIK.S
2338	▶ 1	1315.1470	2628.2794	2628.2739	0.0055	0	146	1.1e-14	▶ 1		■	■	■					R.NANDGISVAQTTEGALSEINNNLQR.I
2339		877.1006	2628.2800	2628.2739	0.0061	0	72	3.1e-07	▶ 1		■	■	■					R.NANDGISVAQTTEGALSEINNNLQR.I
2371	▶ 1	937.1163	2808.3271	2808.3261	0.0010	1	64	1.2e-06	▶ 1		■	■						R.ELTVQASTGTNSDSDLDSIQDEIKSR.L
2379	▶ 1	945.8002	2834.3788	2834.3781	0.0006	1	118	4.7e-12	▶ 1		■	■						R.IRELTVQASTGTNSDSDLDSIQDEIK.S
2381		946.1495	2835.4267	2835.4237	0.0030	1	34	0.0012	▶ 1	U	■							K.LTTEATTASSSTADPLKALDEAISSIDK.F
2396		987.8094	2960.4064	2960.4039	0.0024	1	85	3.3e-09	▶ 1	U	■							K.AAANADVYVEDGALSANATKDVTYFEQK.N
2397		1481.2140	2960.4134	2960.4039	0.0095	1	82	7e-09	▶ 1	U	■							K.AAANADVYVEDGALSANATKDVTYFEQK.N
2398	▶ 1	988.8193	2963.4361	2963.4360	0.0000	0	76	2.8e-08	▶ 1	U	■							K.DATLTVTSGTGQNTVYSTTGSGAQFTSLAK.V
2400		1482.7270	2963.4394	2963.4360	0.0034	0	125	3.5e-13	▶ 1	U	■							K.DATLTVTSGTGQNTVYSTTGSGAQFTSLAK.V
2433	▶ 1	1054.5330	3160.5772	3160.5708	0.0063	1	99	3.4e-10	▶ 1		■	■	■	■				R.SSLGAVQNRLDSAVTNLNNTTTNLSEAQSR.I
2442	▶ 1	1086.5750	3256.7032	3256.7011	0.0021	1	128	6.9e-13	▶ 1		■	■				■		M.AQVINTNSLSLITQNNINKNQSALSSSIER.L
2486	▶ 1	1182.2430	3543.7072	3543.7064	0.0008	1	140	1.1e-14	▶ 1	U	■							K.NGAITNSTGGTIYETADGKLTTEATTASSSTADPLK.A

▶ 71 subsets and intersections (159 subset proteins in total)

▶ 2 gi\|112820172 16 H21 0\|EHEC serogroup: O113:H21\|0

▶ 3 gi\|307553085 15 Hxx(H54 27.9%) 0\|0\|ABU 83972

---

10 per page 1

Mascot: http://www.matrixscience.com/
