# Supplementary material for: MS-H: A Novel Proteomic Approach to Isolate and Type the E. coli H Antigen Using Membrane Filtration and Liquid Chromatography-Tandem Mass Spectrometry (LC-MS/MS)
Source: PLoS One. 2013 Feb 21;8(2):e57339. doi: 10.1371/journal.pone.0057339 (PMC3578835; doi:10.1371/journal.pone.0057339)
Supplement: Representative Peptide Data S1 — Peptide data are represented as the Mascot search results from all 53 serotypes, obtained under the Orbitrap platform in Table 4 with related E. coli reference strains. “U” denotes a unique peptide specific for each of the proteins 1.1, 1.2, and beyond. The number 1.1 (shown as 1 in the peptide list and phylogenetic tree) represents the protein which obtained the highest score and confidence value after a Mascot search. This protein, known as the first hit, was used to designate the MS-H type of the unknown flagellin. Related peptides 1.2 (2), 1.3 (3), etc. represented the second, third, etc. hits for MS-H typing analysis. (DOCX) [file pone.0057339.s009.docx › H10-E659.pdf]

MATRIX SCIENCE MASCOT Search Results

User :
E-mail :
Search title : Submitted from 20110714-H1-H11 by Mascot Daemon on VARIABLE
MS data file : C:\Documents and Settings\keding\Desktop\Raw data\20110714-H1-H11\20110714-013-EC659MS1.RAW
Database : Flagellin_v2 (192 sequences; 89,845 residues)
Taxonomy : Bacteria (Eubacteria) (192 sequences)
Timestamp : 15 Jul 2011 at 17:15:57 GMT

Not what you expected? Try **the select summary**.

Search parameters

Score distribution

Legend

Protein Family Summary

Significance threshold p< 0.05 Max. number of families AUTO
Ions score or expect cut-off 0 Dendrograms cut at 0

Protein family 1 (out of 1)

10 per page 1

1

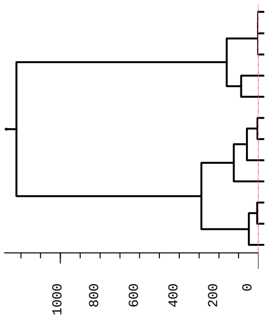

1	gi|30059862	6889	H10 0|H10|Bi 623-42
11	gi|112820171	675	H21 0|EHEC serogroup: O113:H21|0 [gi|291323135|ref|EFE62563| Hxx 0|0|B088] [gi|29344630...
12	gi|33590252	640	Hxx(H8 89.5%) 0|0|PC41
7	gi|33590227	1376	H42 NM|O157:NM(H42)|25
8	gi|30577587	1273	H17 0|O15:H17|P12b
2	gi|3377710	3940	H9 0|0|O78:K80; EC341 95 [gi|30059860|ref|AAP13302| H9 0|H9|Bi 7575-41]
9	gi|33590243	1247	H16 NM|O157:NM(H16)|PC02
4	gi|16901500	2364	H49 0|H49|0
6	gi|5107855	1815	Hxx(H6 99.6%) 0|0|E2348/69
3	gi|30059894	3425	H30 0|H30|HW32 [gi|188494683|ref|ZP_03001953| Hxx 0|0|53638] [gi|188489882|ref|EDU649...
10	gi|5107837	1205	H7 0|O128:H7|DEC 13a
5	gi|121144335	1996	H55 0|O75:H55|E2987-73

Threshold (**0**): 0

		Score	Mass	Matches	Sequences	emPAI
1.1	gi|30059862<br>H10 0|H10|Bi 623-42<br>**1 sameset of gi|30059862**	6889	44249	155 (136)	50 (46)	85.38
1.2	gi|3377710<br>H9 0|0|O78:K80; EC341 95 [gi|30059860|ref|AAP13302| H9 0|H9|Bi 7575-41]	3940	68093	96 (72)	33 (28)	4.46
1.3	gi|30059894<br>H30 0|H30|HW32 [gi|188494683|ref|ZP_03001953| Hxx 0|0|53638] [gi|188489882|ref|EDU64985| Hxx 0|0|53638]	3425	58102	85 (67)	24 (19)	2.97
1.4	gi|16901500<br>H49 0|H49|0	2364	57940	71 (49)	22 (15)	2.02
1.5	gi|121144335<br>H55 0|O75:H55|E2987-73	1996	62285	53 (37)	16 (11)	1.06
1.6	gi|5107855<br>Hxx(H6 99.6%) 0|0|E2348/69	1815	52951	65 (41)	20 (12)	1.63
1.7	gi|33590227<br>H42 NM|O157:NM(H42)|25	1376	44094	37 (26)	13 (9)	1.38
1.8	gi|30577587<br>H17 0|O15:H17|P12b	1273	36285	36 (24)	12 (9)	1.85
1.9	gi|33590243<br>H16 NM|O157:NM(H16)|PC02<br>**3 samesets of gi|33590243**	1247	55093	31 (24)	9 (7)	0.79
1.10	gi|5107837<br>H7 0|O128:H7|DEC 13a	1205	56230	33 (24)	8 (6)	0.58
1.11	gi|112820171<br>H21 0|EHEC serogroup: O113:H21|0 [gi|291323135|ref|EFE62563| Hxx 0|0|B088] [gi|293446305|ref|ZP_06662727| Hxx 0|0|B088]<br>**8 samesets of gi|112820171**	675	51472	17 (15)	7 (6)	0.64
1.12	gi|33590252<br>Hxx(H8 89.5%) 0|0|PC41	640	52373	40 (13)	8 (5)	0.53

286 peptide matches (124 non-duplicate, 162 duplicate)

Query	Dupes	Observed	Mr(expt)	Mr(calc)	Delta	M	Score	Expect	Rank	U	1	2	3	4	5	6	7	8	9	10	11	12	Peptide
6	1	301.1998	600.3850	601.3799	-0.9949	1	4	0.38	2	U			■										K.KVDIK.G
21	2	316.6906	631.3666	631.3653	0.0013	0	27	0.02	1		■	■	■	■	■	■	■	■	■	■	■	■	R.LSSGLR.I
73		351.7054	701.3962	700.4119	0.9843	1	7	0.33	1	U												■	K.DPTKLK.A
85	1	355.1977	708.3808	708.3806	0.0002	0	21	0.053	1		■	■	■	■	■	■	■	■		■			R.FTSNIK.G
88	3	358.7067	715.3988	715.3977	0.0012	0	45	0.00021	1		■	■	■	■	■	■	■	■		■			K.GLTQAAR.N
91	1	359.2111	716.4076	716.4068	0.0008	0	55	2.4e-05	1	U	■												K.LDASALK.A

Query	Dupes	Observed	Mr(expt)	Mr(calc)	Delta	M	Score	Expect	Rank	U	1	2	3	4	5	6	7	8	9	10	11	12	Peptide
93		359.6909	717.3672	717.3657	0.0015	0	48	1.6e-05	1	U	■												K.DGATINK.Q
115	1	380.6953	759.3760	759.3763	-0.0002	0	2	1.2	2			■	■	■	■	■		■		■			R.LDEIDR.V
126	1	387.7034	773.3922	773.3919	0.0003	0	36	0.00063	1		■										■		R.LEEIDR.V
133	1	394.7294	787.4442	787.4440	0.0003	1	34	0.00036	1	U	■												K.LVEKDGK.Y
178		409.7221	817.4296	816.4341	0.9955	0	4	0.58	2	U											■		K.LTANVDGK.A
202	2	417.2403	832.4660	832.4654	0.0006	0	44	4.3e-05	1	U	■												K.ASGSLGTLK.L
228	23	421.7587	841.5028	841.4658	0.0371	0	15	0.03	1	U												■	K.AVTQPQAK.D
246		423.7379	845.4612	846.4447	-0.9834	0	6	0.23	2	U						■							K.AATIQTDK.G
272		431.2271	860.4396	859.4399	0.9997	0	23	0.0047	1	U		■											K.VNISQDGK.I
272		431.2271	860.4396	860.4240	0.0157	0	10	0.1	2	U												■	K.VELGGSDGK.T
312	2	451.7168	901.4190	901.4181	0.0009	0	42	7e-05	1	U	■												K.YYVNDTK.S
341	5	466.2507	930.4868	930.4883	-0.0014	0	74	2e-07	1		■	■	■		■	■		■	■				R.SSLGAVQNR
410		486.2694	970.5242	971.5148	-0.9906	0	0	0.95	1	U												■	R.SNLGAIQNR.F
439	1	496.2625	990.5104	990.5135	-0.0030	0	62	1e-06	1	U	■												K.QVAVGAGDFK.D
488		509.2691	1016.5236	1016.5250	-0.0014	1	44	4.4e-05	1	U	■												K.SRLEEIDR.V
489		339.8493	1016.5261	1016.5250	0.0010	1	27	0.0018	1	U	■												K.SRLEEIDR.V
498	3	511.2877	1020.5608	1020.5604	0.0005	0	57	3.1e-06	1	U	■												K.ANQSLVVYK.D
557	1	530.3041	1058.5936	1058.5972	-0.0035	1	47	1.8e-05	1	U	■												R.DVKLDASALK.A
559		353.8732	1058.5978	1058.5972	0.0006	1	38	0.00014	1	U	■												R.DVKLDASALK.A
571		538.7726	1075.5306	1075.5509	-0.0203	0	10	0.15	1	U						■							K.GETANTAATLK.D
572	1	538.8007	1075.5868	1075.5873	-0.0005	1	27	0.003	1	U	■												K.DKASGSLGTLK.L
573	1	539.2698	1076.5250	1077.4873	-0.9622	0	11	0.11	1	U									■				K.NDGSAQAIMR.E + Oxidation (M)
575		539.2798	1076.5450	1076.5462	-0.0011	0	31	0.0012	1	U						■							-.QSALSSSIER.L
628	1	551.2671	1100.5196	1100.5210	-0.0014	0	76	2.3e-07	1		■	■	■	■	■	■	■	■	■		■	■	K.DDAAGQAIANR.F
646	2	559.2905	1116.5664	1116.5663	0.0002	0	91	7.6e-10	1		■		■										K.ALDDAIASVDK.F
775	1	595.7635	1189.5124	1189.5139	-0.0014	0	67	1.2e-06	1	U	■												K.YYDAEVDTSK.G
777		397.5587	1189.6543	1190.6507	-0.9964	1	2	3.2	1	U						■							R.DTTKATVTIGGK.D
779	2	596.3017	1190.5888	1190.5891	-0.0002	0	67	9.9e-07	1		■	■	■	■									K.NQSALSSSIER.L
782		598.8018	1195.5890	1194.5517	1.0374	0	3	0.56	1	U				■									K.DAAQSSIDFGGK.K
789		600.8531	1199.6916	1199.6734	0.0182	1	18	0.017	1	U									■				K.LRSSLGAVQNR.F
791		601.7866	1201.5586	1201.5615	-0.0029	1	28	0.0014	1	U	■												K.DGKYYVNDTK.S
801	1	605.3088	1208.6030	1208.6037	-0.0007	0	73	5.4e-08	1	U	■												K.SGNDAYIIQTK.D
849	1	617.8240	1233.6334	1233.6354	-0.0019	1	63	1.8e-06	1	U	■												K.QVAVGAGDFKDK.A
850		412.2185	1233.6337	1233.6354	-0.0017	1	49	4.8e-05	1	U	■												K.QVAVGAGDFKDK.A
855		619.8574	1237.7002	1238.5561	-0.8558	0	7	0.22	1	U					■								K.NQSSMSTAIER.L + Oxidation (M)
882		627.8040	1253.5934	1254.6244	-1.0310	0	3	0.53	1	U				■									K.FNALDAATAFSK.L
900		422.2342	1263.6808	1263.6823	-0.0015	1	42	6.3e-05	1	U	■												K.ANQSLVVYKDK.S
901	1	632.8478	1263.6810	1263.6823	-0.0012	1	61	7.3e-07	1	U	■												K.ANQSLVVYKDK.S
942		645.2855	1288.5564	1288.5571	-0.0007	0	54	3.6e-06	1	U		■											K.SYSFAADGADSAK.T
965		651.8098	1301.6050	1301.6827	-0.0776	0	18	0.039	1	U											■		K.AATLSDLDLNAAK.K
986		656.8648	1311.7150	1311.7146	0.0004	0	20	0.01	1	U										■			K.AQIIQQAGNSVLA.-
1033		672.8773	1343.7400	1343.7408	-0.0008	0	62	6.3e-07	1	U					■								-.SLSLITQNNINK.N
1036	1	675.3282	1348.6418	1348.6470	-0.0052	0	61	8.5e-07	1	U		■											K.GSVANTAATSDDLK.L
1060	1	688.3214	1374.6282	1374.6303	-0.0021	1	69	1.2e-07	1	U	■												K.YYDAEVDTSKGK.I
1061		459.2168	1374.6286	1374.6303	-0.0017	1	50	9.9e-06	1	U	■												K.YYDAEVDTSKGK.I
1123	1	474.2526	1419.7360	1419.7358	0.0002	1	48	1.6e-05	1		■		■										K.ALDDAIASVDKFR.S
1124	1	710.8756	1419.7366	1419.7358	0.0009	1	98	1.6e-10	1		■		■										K.ALDDAIASVDKFR.S
1131		712.8860	1423.7574	1423.7671	-0.0096	1	6	0.24	1	U									■				K.VYTANITNKTATK.G
1148	5	720.9119	1439.8092	1439.8096	-0.0004	0	112	2.9e-11	1		■	■	■	■	■	■	■						K.AQIIQQAGNSVLAK.A
1164	1	726.8694	1451.7242	1451.7256	-0.0014	1	97	2.2e-10	1	U	■												K.DKSGNDAYIIQTK.D
1167	1	484.9157	1451.7253	1451.7256	-0.0003	1	30	0.001	1	U	■												K.DKSGNDAYIIQTK.D
1223		747.5334	1493.0522	1491.6729	1.3794	1	5	2.1	1	U	■												K.SSKYYDAEVDTSK.G
1224	1	747.9180	1493.8214	1493.8202	0.0013	0	71	4.5e-07	1		■	■	■	■			■	■					K.ANQVPQQVLSLLQG.-
1344	3	796.4261	1590.8376	1590.8366	0.0011	0	69	1.3e-07	1	U	■												R.VSSQTQFNGVNVLAK.D
1367		807.9127	1613.8108	1613.8121	-0.0013	1	111	7.8e-11	1		■	■	■	■	■	■	■	■	■		■	■	R.INSAKDDAAGQAIANR.F
1368		538.9447	1613.8123	1613.8121	0.0002	1	54	3.4e-05	1		■	■	■	■	■	■	■	■	■		■	■	R.INSAKDDAAGQAIANR.F
1404		822.8673	1643.7200	1643.8367	-0.1166	0	2	0.67	1	U							■						K.LSDTVTQVGDGSAAPVK.V
1406		549.2590	1644.7552	1643.8367	0.9185	0	0	0.92	1	U							■						K.LSDTVTQVGDGSAAPVK.V
1433	2	836.3809	1670.7472	1670.7457	0.0015	0	122	4.2e-12	1		■	■	■	■	■	■	■	■		■			R.IQDADYATEVSNMSK.A
1445		840.6140	1679.2134	1678.7832	0.4302	0	8	0.15	1	U					■								K.DMTITSAGGNAQVATDK.A
1457	7	843.9507	1685.8868	1685.8836	0.0033	0	105	3e-10	1			■	■		■					■			K.IQVGANDGETITIDLK.K
1457	6	843.9507	1685.8868	1684.8996	0.9873	0	58	1.4e-05	2					■		■							K.IQVGANDGQTITIDLK.K
1461	1	844.3781	1686.7416	1686.7407	0.0010	0	103	3.6e-10	1		■	■	■	■	■	■	■	■		■			R.IQDADYATEVSNMSK.A + Oxidation (M)
1467		847.4275	1692.8404	1692.8418	-0.0013	0	87	2.1e-09	1	U		■											K.LTTEATTASSSTADPLK.A
1471		565.7605	1694.2597	1692.8418	1.4179	0	3	0.49	1	U		■											K.LTTEATTASSSTADPLK.A
1579		597.9327	1790.7763	1790.8911	-0.1148	1	3	4.1	1		■	■	■	■	■	■	■	■					K.DDAAGQAIANRFTSNIK.G
1606		601.9709	1802.8909	1803.9438	-1.0530	1	0	4.3	1		■	■	■	■									K.NQSALSSSIERLSSGLR.I
1678		935.4445	1868.8744	1868.8752	-0.0008	0	109	1.3e-11	1	U		■											K.NGAITNSTGGTIYETADGK.L
1743	10	966.0002	1929.9858	1929.9830	0.0029	0	89	2.8e-09	1	U	■												K.MNIQVGANDGQTITIDLK.K
1768		975.9749	1949.9352	1949.9330	0.0022	0	121	7.7e-13	1	U		■											K.AAANADVYVEDGALSANATK.D
1774		653.8124	1958.4154	1959.0021	-0.5867	1	5	0.68	1	U						■							K.NNAGGDTQATLAKVATATGAK.A
1793		986.9601	1971.9056	1971.9022	0.0035	0	108	1.5e-11	1	U		■											K.GTTDTNGVTAYTNTISNDK.A
1849		687.0358	2058.0856	2058.0779	0.0076	1	42	6.2e-05	1	U	■												K.MNIQVGANDGQTITIDLKK.I
1870	3	1043.0700	2084.1254	2085.1066	-0.9811	0	78	1.1e-07	5	U							■						M.AQVINTNSLSLITQNNIDK.N
1871	3	1043.0700	2084.1254	2084.1225	0.0029	0	142	4.6e-14	1		■	■	■	■					■		■	■	M.AQVINTNSLSLiTQNNiNK.N
1872	1	695.7164	2084.1274	2084.1225	0.0048	0	71	4.9e-07	1		■	■	■	■					■		■	■	M.AQVINTNSLSLiTQNNiNK.N
1872	1	695.7164	2084.1274	2085.1066	-0.9792	0	69	8.7e-07	4	U							■						M.AQVINTNSLSLITQNNIDK.N
1872	1	695.7164	2084.1274	2085.0814	-0.9540	0	66	1.6e-06	5	U								■					M.AQVINTNSLSLNTQNNINK.N
1876	3	1043.5620	2085.1094	2085.0814	0.0280	0	91	5.8e-09	1	U								■					M.AQVINTNSLSLNTQNNINK.N
1921		724.0348	2169.0826	2169.0801	0.0025	0	5	0.35	1	U	■												K.IDSSTLNLSSFDATNLGTSVK.D
1923	1	1085.5490	2169.0834	2169.0801	0.0033	0	112	6.4e-12	1	U	■												K.IDSSTLNLSSFDATNLGTSVK.D
1924	1	724.3642	2170.0708	2170.0688	0.0019	0	18	0.018	1	U		■											K.VDTVNVTNAHVSAEGMANLTK.S
1925		1086.0430	2170.0714	2170.0688	0.0026	0	67	2e-07	1	U		■											K.VDTVNVTNAHVSAEGMANLTK.S

Query	Dupes	Observed	Mr(expt)	Mr(calc)	Delta	M	Score	Expect	Rank	U	1	2	3	4	5	6	7	8	9	10	11	12	Peptide
1926		1087.5720	2173.1294	2173.1226	0.0068	0	96	2.3e-10	1	U		■											K.TLSIINPNTGDSSQATVTIGGK.E
1954	3	1117.0570	2232.0994	2232.0982	0.0013	0	130	7.3e-13	1	U	■												R.LDSAIANLNNTTTNLSEAQSR.I
1956	1	745.0406	2232.1000	2232.0982	0.0018	0	90	7.4e-09	1	U	■												R.LDSAIANLNNTTTNLSEAQSR.I
1957	1	1117.5490	2233.0834	2234.0775	-0.9940	0	22	0.0089	2	U				■									R.LDSAVTNLNNTSTNLSEAQSR.I
1967		1125.0550	2248.0954	2248.0931	0.0023	0	113	2.8e-11	1			■	■		■		■	■					R.LDSAVTNLNNTTTNLSEAQSR.I
1991	1	1149.5960	2297.1774	2297.1751	0.0024	1	135	2.9e-14	1	U	■												K.KIDSSTLNLSSFDATNLGTSVK.D
1994		766.7339	2297.1799	2297.1751	0.0048	1	50	9.7e-06	1	U	■												K.KIDSSTLNLSSFDATNLGTSVK.D
2011		1174.1170	2346.2194	2346.2179	0.0015	1	48	1.6e-05	1	U	■												R.LEEIDRVSSQTQFNGVNVLAK.D
2012		783.0810	2346.2212	2346.2179	0.0032	1	68	1.4e-07	1	U	■												R.LEEIDRVSSQTQFNGVNVLAK.D
2019	2	1182.5580	2363.1014	2363.0952	0.0063	0	72	6.8e-08	1	U		■											K.SNFTIDMGGTGTVTYTVSNGDVK.A
2044	1	1234.6000	2467.1854	2467.1827	0.0028	0	133	5.2e-14	1	U	■												K.INFNSTNESGTTPTAATEVTTVGR.D
2045		823.4035	2467.1887	2467.1827	0.0060	0	43	4.5e-05	1	U	■												K.INFNSTNESGTTPTAATEVTTVGR.D
2054	1	1245.6460	2489.2774	2489.2762	0.0013	0	138	1.4e-14	1	U		■											K.ASDLLANITDGSVITGGGANAFGVAAK.N
2055		830.7665	2489.2777	2489.2762	0.0015	0	59	1.2e-06	1	U		■											K.ASDLLANITDGSVITGGGANAFGVAAK.N
2075	7	1283.6040	2565.1934	2565.1930	0.0005	0	117	5.8e-12	1			■		■		■							R.ELTVQASTGTNSDSDLDSIQDEIK.S
2076	1	856.0844	2565.2314	2565.2294	0.0020	0	29	0.0035	2	U			■										R.ELTVQATTGTNSTSDLDSIQDEIK.S
2076	1	856.0844	2565.2314	2565.1930	0.0384	0	24	0.011	3			■		■		■							R.ELTVQASTGTNSDSDLDSIQDEIK.S
2081	8	1283.6240	2565.2334	2565.2293	0.0041	0	157	5.9e-16	1	U	■												R.ELTVQATTGTNSESDLSSIQDEIK.S
2081	8	1283.6240	2565.2334	2565.2294	0.0041	0	76	7.7e-08	2	U			■										R.ELTVQATTGTNSTSDLDSIQDEIK.S
2082	1	856.0854	2565.2344	2565.2293	0.0050	0	44	0.00011	1	U	■												R.ELTVQATTGTNSESDLSSIQDEIK.S
2102	4	1315.1460	2628.2774	2628.2739	0.0035	0	144	1.7e-14	1		■	■	■	■	■	■			■	■			R.NANDGISVAQTTEGALSEINNNLQR
2103	2	877.1003	2628.2791	2628.2739	0.0052	0	83	2.2e-08	1		■	■	■	■	■	■			■	■			R.NANDGISVAQTTEGALSEINNNLQR
2112		885.1066	2652.2980	2652.2991	-0.0011	1	50	1e-05	1	U	■												K.GKINFNSTNESGTTPTAATEVTTVGR.D
2151		937.1285	2808.3637	2808.3625	0.0012	1	54	1.2e-05	1	U	■												R.ELTVQATTGTNSESDLSSIQDEIKSR.L
2151		937.1285	2808.3637	2808.3625	0.0012	1	30	0.0029	2	U			■										R.ELTVQATTGTNSTSDLDSIQDEIKSR.L
2151		937.1285	2808.3637	2808.3261	0.0376	1	7	0.58	3			■		■		■							R.ELTVQASTGTNSDSDLDSIQDEIKSR.L
2152		941.1411	2820.4015	2820.3989	0.0026	1	96	2.3e-10	1	U	■												R.VRELTVQATTGTNSESDLSSIQDEIK.S
2156		945.8010	2834.3812	2834.3781	0.0030	1	73	1.6e-07	1			■		■		■							R.IRELTVQASTGTNSDSDLDSIQDEIK.S
2156		945.8010	2834.3812	2834.4145	-0.0333	1	30	0.0034	2	U			■										R.IRELTVQATTGTNSTSDLDSIQDEIK.S
2170		988.8188	2963.4346	2963.4360	-0.0015	0	56	2.5e-06	1	U		■											K.DATLTVTSGTGQNTVYSTTGSGAQFTSLAK.V
2171		1482.7250	2963.4354	2963.4360	-0.0006	0	75	3.2e-08	1	U		■											K.DATLTVTSGTGQNTVYSTTGSGAQFTSLAK.V
2193	1	1049.2000	3144.5782	3144.5759	0.0023	1	90	9.2e-10	1	U	■												R.SSLGAVQNRLDSAIANLNNTTTNLSEAQSR.I
2207		1086.5750	3256.7032	3256.7011	0.0021	1	128	6.9e-13	1		■	■	■	■									M.AQVINTNSLSLITQNNINKNQSALSSSIER.L
2236		1125.9400	3374.7982	3374.7570	0.0412	1	0	0.95	1	U							■						K.IDSSALGLSGFSVAGGALKLSDTVTQVGDGSAAF
2276	1	1254.6350	3760.8832	3760.8755	0.0076	0	65	3.2e-07	1	U	■												K.DVTTNQSTFNAANISDAGVLSIGASTTAPSNLTA

57 subsets and intersections (149 subset proteins in total)

10 per page 1

Not what you expected? Try the select summary.

Mascot: http://www.matrixscience.com/
