# Supplementary material for: MS-H: A Novel Proteomic Approach to Isolate and Type the E. coli H Antigen Using Membrane Filtration and Liquid Chromatography-Tandem Mass Spectrometry (LC-MS/MS)
Source: PLoS One. 2013 Feb 21;8(2):e57339. doi: 10.1371/journal.pone.0057339 (PMC3578835; doi:10.1371/journal.pone.0057339)
Supplement: Representative Peptide Data S1 — Peptide data are represented as the Mascot search results from all 53 serotypes, obtained under the Orbitrap platform in Table 4 with related E. coli reference strains. “U” denotes a unique peptide specific for each of the proteins 1.1, 1.2, and beyond. The number 1.1 (shown as 1 in the peptide list and phylogenetic tree) represents the protein which obtained the highest score and confidence value after a Mascot search. This protein, known as the first hit, was used to designate the MS-H type of the unknown flagellin. Related peptides 1.2 (2), 1.3 (3), etc. represented the second, third, etc. hits for MS-H typing analysis. (DOCX) [file pone.0057339.s009.docx › H11-07-6285.pdf]

MASCOT Search Results

User :
E-mail :
Search title : Submitted from 20110915-0644 by Mascot Daemon on VARIABLE
MS data file : C:\Documents and Settings\keding\Desktop\Raw data\20110915-001-0031-00644\20110915-004-076285MS1-RP.RAW
Database : Flagellin_v2 (192 sequences; 89,845 residues)
Taxonomy : Bacteria (Eubacteria) (192 sequences)
Timestamp : 18 Sep 2011 at 14:35:11 GMT

Not what you expected? Try **the select summary**.

Search parameters
Score distribution
Legend

Protein Family Summary

Significance threshold p< 0.05 Max. number of families AUTO
Ions score or expect cut-off 0 Dendrograms cut at 0

Protein family 1 (out of 1)

10 per page 1

1

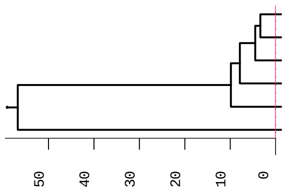

1	gi|257754552	2367	H11 0|O26:H11|11368 [gi|260855903|ref|YP_003229794| H11 0|O26:H11|11368] [...
5	gi|6941882	243	H14 0|O138:H14|O138:H14
4	gi|30059910	245	H39 0|H39|E3a
2	gi|154710451	607	H36 0|H36|0
3	gi|6009849	250	H49 0|H49|U11a-44
6	gi|121144335	199	H55 0|O75:H55|E2987-73

Threshold (**0**): 0

		Score	Mass	Matches	Sequences	emPAI
1.1	gi|257754552<br>H11 0|O26:H11|11368 [gi|260855903|ref|YP_003229794| H11 0|O26:H11|11368] [gi|323152744|ref|EFZ39016| Hxx 0|0|EPECa14]	2367	50921	62 (51)	36 (33)	19.26
1.2	gi|154710451<br>H36 0|H36|0	607	57784	20 (14)	15 (9)	1.17
1.3	gi|6009849<br>H49 0|H49|U11a-44	250	58493	9 (7)	7 (5)	0.47
1.4	gi|30059910<br>H39 0|H39|E3a	245	44239	9 (7)	7 (5)	0.65
1.5	gi|6941882<br>H14 0|O138:H14|O138:H14	243	56492	19 (8)	17 (6)	0.57
1.6	gi|121144335<br>H55 0|O75:H55|E2987-73	199	62285	7 (5)	6 (4)	0.29

86 peptide matches (77 non-duplicate, 9 duplicate)

Query	Dupes	Observed	Mr(expt)	Mr(calc)	Delta	M	Score	Expect	Rank	U	1	2	3	4	5	6	Peptide
24		316.6882	631.3618	631.3653	-0.0035	0	30	0.0092	1		■	■	■	■	■	■	R.LSSGLR.I
71	2	347.1977	692.3808	692.3857	-0.0049	0	28	0.0038	1	U	■						R.FTANIK.G
100		366.7018	731.3890	731.3926	-0.0035	0	3	1.9	1		■	■					K.GLTQASR.N
107		370.7016	739.3886	739.3938	-0.0052	0	27	0.0027	1	U	■						K.VMYLSK.S
117	1	378.6989	755.3832	755.3887	-0.0055	0	35	0.00047	1	U	■						K.VMYLSK.S + Oxidation (M)
135		387.7005	773.3864	773.3919	-0.0055	0	37	0.00043	1	U	■						R.LEEIDR.V
145		396.2393	790.4640	790.4185	0.0456	0	0	0.94	1	U		■					K.TGTNTAVK.S
217		423.2188	844.4230	844.4402	-0.0172	0	23	0.0051	1	U			■				K.AAAGAESIR.Y
222		424.7007	847.3868	846.4447	0.9422	0	2	0.73	3	U					■		K.AATTADGLK.A
274		438.7399	875.4652	876.4552	-0.9900	0	5	0.32	1	U					■		K.AATTADSLK.A
289		446.2389	890.4632	890.4709	-0.0077	0	46	7.9e-05	1	U	■						K.ATGSDLISK.F
290		447.7283	893.4420	892.4290	1.0130	0	16	0.027	1	U					■		K.VDQAAFDK.A
330		466.7398	931.4650	930.4883	0.9768	0	3	2.1	1					■	■	■	R.SSLGAVQNR.L
381		480.2447	958.4748	958.4832	-0.0084	0	68	2.9e-07	1	U	■						R.SDLGAVQNR.F
395		484.2055	966.3964	965.4818	0.9147	1	1	0.73	1	U		■			■		K.avYKDADGK.L
414		487.7445	973.4744	972.4988	0.9756	0	22	0.0091	1	U		■					R.SDLGAIQNR.F
436		493.7084	985.4022	985.5556	-0.1534	0	3	0.53	1	U						■	K.AAASNVLAAAK.N
493		508.7866	1015.5586	1016.5502	-0.9916	0	18	0.018	1	U				■			K.NVDLSAVATK.L
546		524.2562	1046.4978	1046.5066	-0.0088	0	34	0.00069	1	U	■						K.VLAENNEMK.I
562		528.2675	1054.5204	1054.5295	-0.0090	0	69	1.8e-07	1	U	■						K.NSAGLYTTTK.V
573		531.2677	1060.5208	1060.5513	-0.0304	0	0	0.92	1	U		■					K.GSVNNTVATAK.D
576		532.2544	1062.4942	1062.5015	-0.0073	0	31	0.0016	1	U	■						K.VLAENNEMK.I + Oxidation (M)

Query	Dupes	Observed	Mr(expt)	Mr(calc)	Delta	M	Score	Expect	Rank	U	1	2	3	4	5	6	Peptide
657		551.2629	1100.5112	1100.5210	-0.0098	0	67	2e-06	▶1		■	■	■	■	■	■	K.DDAAGQAIANR.F
716		568.2556	1134.4966	1133.5564	0.9402	1	1	1.4	▶1	U		■					K.DADGKITTDAK.T
718	▶1	568.2785	1134.5424	1134.5517	-0.0092	0	54	7e-06	▶1	U	■						K.TITETASGNNK.V
766		581.3165	1160.6184	1160.5925	0.0260	0	0	1.3	▶1	U					■		K.ALDEAISSIDK.F
770		582.7917	1163.5688	1163.5782	-0.0094	0	63	1.4e-06	▶1		■	■					K.SQSSLSSAIER.L
802		592.2910	1182.5674	1182.5769	-0.0094	0	64	4.1e-07	▶1	U	■						K.SVTFTVTDADK.K
813		397.8860	1190.6362	1190.5891	0.0471	0	7	1.2	▶1				■		■		K.NQSALSSSIER.L
819		598.7963	1195.5780	1194.5517	1.0264	0	6	0.28	▶1	U			■				K.DAAQSSIDFGGK.K
844		606.3245	1210.6344	1210.6445	-0.0101	0	81	7.4e-09	▶1	U	■						K.ASLITSETVYK.N
889	▶2	619.3011	1236.5876	1235.6146	0.9730	0	61	2.3e-06	▶1	U	■						R.VSEQTQFNGVK.V
1009	▶1	651.8432	1301.6718	1301.6827	-0.0108	0	82	1.4e-08	▶1	U	■						K.AATLSDLDLNAAK.K
1026		656.3381	1310.6616	1310.6718	-0.0102	1	80	1.2e-08	▶1	U	■						K.SVTFTVTDADKK.A
1027		437.8945	1310.6617	1310.6718	-0.0101	1	48	1.9e-05	▶1	U	■						K.SVTFTVTDADKK.A
1077		448.2449	1341.7129	1341.7252	-0.0123	0	9	0.13	▶1	U					■		K.ADLVAANATVVGNK.Y
1081		672.8722	1343.7298	1343.7408	-0.0110	0	70	1.1e-07	▶1	U						■	-.SLSLITQNNINK.N
1188		477.5958	1429.7656	1429.7776	-0.0120	1	18	0.024	▶1	U	■						K.AATLSDLDLNAAKK.T
1189		715.8906	1429.7666	1429.7776	-0.0110	1	51	1.4e-05	▶1	U	■						K.AATLSDLDLNAAKK.T
1214		724.8676	1447.7206	1447.7307	-0.0101	0	77	4.2e-08	▶1	U	■						K.TLGLDGFNIDGAQK.A
1262	▶1	743.8663	1485.7180	1485.7311	-0.0130	0	67	3.1e-07	▶1	U	■						K.SEGGSPILVNEDAAK.S
1324		773.8953	1545.7760	1545.7886	-0.0126	0	72	1.2e-07	▶1	U	■						K.SLQSTTNPLETIDK.A
1382		538.9395	1613.7967	1613.8121	-0.0154	1	27	0.018	▶1		■	■	■	■	■	■	R.INSAKDDAAGQAIANR.F
1383		807.9072	1613.7998	1613.8121	-0.0123	1	86	2.1e-08	▶1		■	■	■	■	■	■	R.INSAKDDAAGQAIANR.F
1448		567.5818	1699.7236	1699.7359	-0.0123	0	50	1.6e-05	▶1		■	■					R.IEDADYATEVSNMSR.A
1449		850.8693	1699.7240	1699.7359	-0.0119	0	122	9.5e-13	▶1		■	■					R.IEDADYATEVSNMSR.A
1456		853.8831	1705.7516	1705.8734	-0.1217	1	1	0.82	▶1	U					■		K.LTTDAETKAATTADGLK.A
1465		858.8655	1715.7164	1715.7308	-0.0144	0	108	3.3e-11	▶1		■	■					R.IEDADYATEVSNMSR.A + Oxidation (M)
1478		862.3900	1722.7654	1723.8741	-1.1086	1	5	0.32	▶1	U					■		K.ITASNGDKLYIDTTGR.L
1489		579.6204	1735.8394	1735.8840	-0.0446	1	14	0.042	▶1	U					■		K.LTTDAETKAATTADSLK.A
1572		457.4684	1825.8445	1826.9163	-1.0718	1	2	0.57	▶1	U		■					K.TYTGSAGLANAKAGDVFGK.M
1575		916.4657	1830.9168	1830.9323	-0.0154	0	87	2.2e-09	▶1	U	■						K.SDTNIAGTGIDATALAAAAK.N
1576		611.3140	1830.9202	1830.9323	-0.0121	0	13	0.052	▶1	U	■						K.SDTNIAGTGIDATALAAAAK.N
1596		927.9503	1853.8860	1853.9007	-0.0147	0	95	4.7e-10	▶1	U	■						K.TGSTLVVNGATYDVSADGK.T
1597		618.9694	1853.8864	1853.9007	-0.0143	0	59	2e-06	▶1	U	■						K.TGSTLVVNGATYDVSADGK.T
1648		638.3046	1911.8920	1911.9062	-0.0142	0	13	0.055	▶1	U	■						K.DTTGNDIFVSAADGSLTTK.S
1649	▶1	956.9548	1911.8950	1911.9062	-0.0111	0	62	6.1e-07	▶1	U	■						K.DTTGNDIFVSAADGSLTTK.S
1662		644.0156	1929.0250	1929.0418	-0.0169	1	12	0.15	▶1	U	■						K.SLQSTTNPLETIDKALAK.V
1673		648.6250	1942.8532	1942.8690	-0.0159	1	38	0.00024	▶1		■	■					R.SRIEDADYATEVSNMSR.A
1674		972.4342	1942.8538	1942.8690	-0.0152	1	51	1.5e-05	▶1		■	■					R.SRIEDADYATEVSNMSR.A
1700		661.6673	1981.9801	1981.9957	-0.0156	1	35	0.0007	▶1	U	■						K.KTGSTLVVNGATYDVSADGK.T
1701		991.9987	1981.9828	1981.9957	-0.0128	1	77	3.9e-08	▶1	U	■						K.KTGSTLVVNGATYDVSADGK.T
1707		664.6672	1990.9798	1990.9960	-0.0162	1	28	0.0028	▶1	U	■						R.LEEIDRVSEQTQFNGVK.V
1708		996.4993	1990.9840	1990.9960	-0.0119	1	53	9.2e-06	▶1	U	■						R.LEEIDRVSEQTQFNGVK.V
1710		665.3320	1992.9742	1992.9865	-0.0123	0	30	0.0028	▶1	U	■						R.FDSAITNLGNTVNNLSSAR.S
1711		997.4949	1992.9752	1992.9865	-0.0112	0	125	7.7e-13	▶1	U	■						R.FDSAITNLGNTVNNLSSAR.S
1724		674.3380	2019.9922	2019.0371	0.9550	1	4	0.38	▶1	U					■		K.AATTADSLKALDEAISSIDK.F
1745		688.0304	2061.0694	2061.0477	0.0217	1	7	0.24	▶1	U					■		K.AATTADELKALDEAISSIDK.F
1755		1043.0600	2084.1054	2084.1225	-0.0171	0	94	2.7e-09	▶1		■	■	■	■	■		M.AQVINTNSLSLiTQNNiNK.N
1756		695.7103	2084.1091	2084.1225	-0.0135	0	46	0.00017	▶1		■	■	■	■	■		M.AQVINTNSLSLiTQNNiNK.N
1875		1195.0340	2388.0534	2388.0718	-0.0183	0	110	1e-11	▶1	U	■						K.ATGTDNYDVGGDAYTVNVDSGAVK.D
1876		797.0257	2388.0553	2388.0718	-0.0165	0	99	1.5e-10	▶1	U	■						K.ATGTDNYDVGGDAYTVNVDSGAVK.D
1914		886.0964	2655.2674	2655.2848	-0.0174	0	71	1.9e-07	▶1	U	■						R.NANDGISVAQTTEGALNEINNNLQR.V
1915		1328.6420	2655.2694	2655.2848	-0.0154	0	122	1.7e-12	▶1	U	■						R.NANDGISVAQTTEGALNEINNNLQR.V
1934		927.1071	2778.2995	2777.3315	0.9680	0	4	0.43	▶1	U	■						R.ELTVQATNGTNSDSDLSSIQAEITQR.L
1937		936.4352	2806.2838	2806.3832	-0.0995	1	4	0.79	▶1	U				■			R.VRELTVQATTGTNSDSDLSSIQDEIK.S
1943		956.1899	2865.5479	2865.5672	-0.0193	0	36	0.00028	▶1		■	■					R.AQILQQAGTSVLAQANQTTQNVLSLLR.-

▶ **43 subsets and intersections (165 subset proteins in total)**

10 per page 1

Not what you expected? Try **the select summary**.

Mascot: http://www.matrixscience.com/
