# Supplementary material for: MS-H: A Novel Proteomic Approach to Isolate and Type the E. coli H Antigen Using Membrane Filtration and Liquid Chromatography-Tandem Mass Spectrometry (LC-MS/MS)
Source: PLoS One. 2013 Feb 21;8(2):e57339. doi: 10.1371/journal.pone.0057339 (PMC3578835; doi:10.1371/journal.pone.0057339)
Supplement: Representative Peptide Data S1 — Peptide data are represented as the Mascot search results from all 53 serotypes, obtained under the Orbitrap platform in Table 4 with related E. coli reference strains. “U” denotes a unique peptide specific for each of the proteins 1.1, 1.2, and beyond. The number 1.1 (shown as 1 in the peptide list and phylogenetic tree) represents the protein which obtained the highest score and confidence value after a Mascot search. This protein, known as the first hit, was used to designate the MS-H type of the unknown flagellin. Related peptides 1.2 (2), 1.3 (3), etc. represented the second, third, etc. hits for MS-H typing analysis. (DOCX) [file pone.0057339.s009.docx › H12-E241M.pdf]

MASCOT Search Results

User : keding
E-mail : chengkeding@yahoo.com
Search title : flagellin
MS data file : C:\Xcalibur\data\20110921-00646\E241M-MS1.RAW
Database : Flagellin_v2 (192 sequences; 89,845 residues)
Taxonomy : Bacteria (Eubacteria) (192 sequences)
Timestamp : 22 Sep 2011 at 17:31:26 GMT

Not what you expected? Try **the select summary**.

Search parameters

Score distribution

Legend

Protein Family Summary

Significance threshold p< 0.05 Max. number of families AUTO
Ions score or expect cut-off 0 Dendrograms cut at 0

Protein families 1–3 (out of 3)

10 per page 1

1

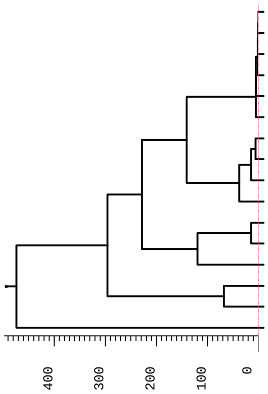

1	gi|6009839	11059	H12 0|0|Bi 316-42 [gi|30059866|ref|AAP13305| H12 0|H12|Bi 316-42]
16	gi|154710458	404	H47 0|H47|0
5	gi|16901498	3532	H34 0|H34|0
6	gi|30059920	3082	H46 0|H46|5306-56
9	gi|88683097	2280	H44 0|H44|0
14	gi|33590260	1077	H29 0|O157:H29|Y63
4	gi|6009849	3537	H49 0|H49|U11a-44
7	gi|6009841	2706	H23 0|0|K42 [gi|30059882|ref|AAP13313| H23 0|H23|HW23]
13	gi|30577587	1263	H17 0|O15:H17|P12b
11	gi|33590227	1763	H42 NM|O157:NM(H42)|25
3	gi|157081065	3539	Hxx(H28 99.7%) 0|0|E24377A [gi|157159035|ref|YP_001463228| Hxx 0|0|E24377A]
15	gi|33590243	773	H16 NM|O157:NM(H16)|PC02
10	gi|121144335	2258	H55 0|O75:H55|E2987-73
12	gi|6941886	1583	H43 0|O157:H43|O157:H43 [gi|30059916|ref|AAP13330| H43 0|H43|149-51]
8	gi|30059874	2663	H18 0|H18|K 12a [gi|293405409|ref|ZP_06649401| Hxx 0|0|FVEC1412] [gi|300899178|ref|ZP_0…
2	gi|5107847	8727	H12 0|O111:H12|DEC 6a

Threshold (**0**): 0

		Score	Mass	Matches	Sequences	emPAI
1.1	gi|6009839	11059	60984	212 (185)	59 (56)	59.31
	H12 0|0|Bi 316-42 [gi|30059866|ref|AAP13305| H12 0|H12|Bi 316-42]					
1.2	gi|5107847	8727	57823	180 (153)	51 (48)	42.08
	H12 0|O111:H12|DEC 6a					
1.3	gi|157081065	3539	59373	81 (65)	27 (25)	5.25
	Hxx(H28 99.7%) 0|0|E24377A [gi|157159035|ref|YP_001463228| Hxx 0|0|E24377A]					
	**3 samesets of gi|157081065**					
1.4	gi|6009849	3537	58493	80 (66)	27 (26)	5.43
	H49 0|H49|U11a-44					
1.5	gi|16901498	3532	56006	82 (65)	28 (25)	5.57
	H34 0|H34|0					
	**3 samesets of gi|16901498**					
1.6	gi|30059920	3082	57918	75 (58)	28 (23)	4.24
	H46 0|H46|5306-56					
	**1 sameset of gi|30059920**					
1.7	gi|6009841	2706	60422	70 (53)	27 (22)	3.41
	H23 0|0|K42 [gi|30059882|ref|AAP13313| H23 0|H23|HW23]					
1.8	gi|30059874	2663	57210	68 (53)	25 (21)	3.79
	H18 0|H18|K 12a [gi|293405409|ref|ZP_06649401| Hxx 0|0|FVEC1412] [gi|300899178|ref|ZP_07117455| Hxx 0|0|MS 198-1] [gi|218705418|ref|YP_002412937| Hxx 0|0|UMN026] [gi|218432515|ref|…					
1.9	gi|88683097	2280	55289	62 (46)	20 (17)	2.78
	H44 0|H44|0					
1.10	gi|121144335	2258	62285	59 (45)	20 (17)	2.27
	H55 0|O75:H55|E2987-73					
1.11	gi|33590227	1763	44094	44 (32)	18 (14)	2.95
	H42 NM|O157:NM(H42)|25					
1.12	gi|6941886	1583	51071	46 (33)	19 (14)	2.49
	H43 0|O157:H43|O157:H43 [gi|30059916|ref|AAP13330| H43 0|H43|149-51]					
1.13	gi|30577587	1263	36285	40 (25)	16 (11)	3.42
	H17 0|O15:H17|P12b					
1.14	gi|33590260	1077	45720	45 (27)	18 (13)	2.51
	H29 0|O157:H29|Y63					
1.15	gi|33590243	773	55093	27 (15)	14 (7)	0.79

		Score	Mass	Matches	Sequences	emPAI
	H16 NM|O157:NM(H16)|PC02					
	2 samesets of gi|33590243					
1.16	gi|154710458	404	39177	22 (12)	9 (7)	1.07
	H47 0|H47|0					

316 peptide matches (159 non-duplicate, 157 duplicate)

Query	Dupes	Observed	Mr(expt)	Mr(calc)	Delta	M	Score	Expect	Rank	U	1	2	3	4	5	6	7	8	9	10	11	12	13	14	15	16	Peptide
21	2	316.6894	631.3642	631.3653	-0.0011	0	31	0.0069	1		■	■	■	■	■	■	■	■	■	■	■	■	■	■	■	■	R.LSSGLR.I
26		318.6892	635.3638	635.2949	0.0690	0	7	0.19	1	U					■												K.ADGMVK.D + Oxidation (M)
56	1	344.2055	686.3964	686.3963	0.0002	0	42	6.9e-05	1		■	■															K.ALDQLK.D
74	3	352.2032	702.3918	702.3912	0.0007	0	23	0.0068	1		■	■															K.ETVLNK.V
74	1	352.2032	702.3918	703.3752	-0.9834	0	22	0.0091	2	U											■						K.DDTLIK.V
74	1	352.2032	702.3918	702.3912	0.0007	0	6	0.33	3	U														■			K.AIASVDK.F
77		353.2179	704.4212	704.3705	0.0508	0	5	0.31	1	U									■								K.DGTLTAK.D
79	1	355.1976	708.3806	708.3806	0.0000	0	22	0.038	1		■	■	■	■	■	■	■	■	■	■	■	■	■	■		■	R.FTSNIK.G
83	1	358.7064	715.3982	715.3977	0.0006	0	35	0.0023	1		■	■	■	■	■	■	■	■	■	■	■	■	■	■			K.GLTQAAR.N
86	2	358.7186	715.4226	715.4228	-0.0002	0	30	0.007	1		■	■															K.ITIGGQK.A
96		367.1988	732.3830	731.3813	1.0017	0	6	0.8	1	U															■		R.LSEIDR.V
113	1	380.2032	758.3918	758.4174	-0.0255	0	30	0.0066	1	U															■		K.LDEALAK.V
114	4	380.6942	759.3738	759.3763	-0.0024	0	37	0.001	1		■	■	■	■	■	■	■	■	■	■			■	■		■	R.LDEIDR.V
127		387.2021	772.3896	771.4490	0.9406	0	16	0.068	1	U													■				K.ALDAAIAK.V
140		395.2388	788.4630	789.3868	-0.9238	0	19	0.012	1	U							■										K.DADGAITK.T
159	1	402.1924	802.3702	801.4708	0.8994	1	2	0.7	2		■	■															K.VATAKQGK.A
194		415.2263	828.4380	828.4381	-0.0001	0	12	0.062	1	U																■	K.YYAVVSK.S
211		423.2213	844.4280	844.4402	-0.0122	0	18	0.015	1	U				■													K.AAAGAESIR.Y
219		424.7208	847.4270	847.4287	-0.0017	0	66	3.1e-07	1		■	■															K.DGDTVTIK.A
241		434.2552	866.4958	867.3610	-0.8652	0	3	0.54	1	U							■										K.FDTDADGK.I
267		440.7528	879.4910	880.4290	-0.9380	0	3	0.45	1	U						■											K.GVYTDAAGK.F
273		445.7260	889.4374	890.5073	-1.0698	1	6	0.96	1	U						■											K.KIDSSTLK.L
286		452.2417	902.4688	902.5073	-0.0384	0	5	0.32	1	U			■														K.AATTADLLK.A
308		458.7688	915.5230	916.4866	-0.9635	0	3	0.49	1	U						■											K.AATTTDPLK.A
344	2	473.2585	944.5024	944.5039	-0.0015	0	73	1.6e-07	1		■	■		■	■	■	■	■									R.SSLGAIQNR.L
395		488.7760	975.5374	974.5033	1.0342	0	1	0.77	1	U						■											K.AGDTANLSVK.I
403		490.2446	978.4746	978.4771	-0.0024	0	101	9.1e-11	1		■	■															K.AGDGQSIGFK.K
405	2	491.2338	980.4530	980.4563	-0.0033	0	39	0.00013	1		■	■															K.YSIDANNGK.V
420		493.7533	985.4920	985.5556	-0.0636	0	3	0.55	2	U										■							K.AAASNVLAAAK.N
423		494.7674	987.5202	988.5189	-0.9987	0	13	0.05	1	U												■					K.TNLVTAADGK.T
428		497.2467	992.4788	991.5008	0.9780	0	3	0.47	1	U															■		K.VLASDQTMK.I
436	1	501.2343	1000.4540	1001.5142	-1.0601	0	20	0.011	1	U			■														K.AGDTANLNVK.I
444	1	502.2605	1002.5064	1002.5094	-0.0030	1	35	0.002	1		■	■	■	■	■	■	■	■	■	■			■	■			K.SRLDEIDR.V
445		335.1772	1002.5098	1002.5094	0.0004	1	32	0.0037	1		■	■	■	■	■	■	■	■	■	■			■	■			K.SRLDEIDR.V
453		503.8627	1005.7108	1005.4727	0.2381	0	2	0.6	1	U								■									K.TNAGTDTQAK.L
454		504.3569	1006.6992	1005.5607	1.1385	1	11	0.1	1	U														■			K.AIASVDKFR.S
473		340.8126	1019.4160	1018.5295	0.8865	1	4	0.57	1	U							■										K.DADGAITKTK.E
477	3	511.2605	1020.5064	1020.5088	-0.0023	0	71	1.2e-07	1		■	■															K.VTVDSGTGTGK.Y
543	1	531.2940	1060.5734	1060.5764	-0.0030	0	75	2.9e-08	1		■	■															K.AATISDLTAAK.M
560		537.7830	1073.5514	1073.5717	-0.0202	0	14	0.037	1	U						■											R.ISADALQSAAK.G
564		539.2687	1076.5228	1077.4873	-0.9644	0	19	0.018	1	U															■		K.NDGSAQAIMR.E + Oxidation (
617	1	551.2661	1100.5176	1100.5210	-0.0034	0	64	3.5e-06	1		■	■	■	■	■	■	■	■	■	■	■	■	■	■	■	■	K.DDAAGQAIANR.F
623		554.2916	1106.5686	1106.5720	-0.0034	1	57	1.8e-06	1		■	■															K.AGDGQSIGFKK.T
624		554.2921	1106.5696	1106.5720	-0.0024	1	46	2.7e-05	1		■	■															K.KAGDGQSIGFK.K
625		369.8646	1106.5720	1106.5720	-0.0000	1	60	1e-06	1		■	■															K.AGDGQSIGFKK.T
626		369.8647	1106.5723	1106.5720	0.0003	1	22	0.0064	1		■	■															K.KAGDGQSIGFK.K
718		387.5471	1159.6195	1160.5925	-0.9730	0	6	0.38	2		■	■	■														K.ALDEAISSIDK.F
722	1	581.3027	1160.5908	1160.5925	-0.0016	0	81	1.1e-08	1		■	■	■														K.ALDEAISSIDK.F
738		585.8391	1169.6636	1169.6518	0.0118	0	14	0.04	1	U				■													R.YIYLLQMVK.L
742	1	587.3078	1172.6010	1172.6765	-0.0754	1	3	0.49	2		■	■															K.ETVLNKVATAK.Q
766		397.2031	1188.5875	1187.6034	0.9841	0	1	0.73	1							■				■							K.ALDDAISQIDK.F
770	1	596.2996	1190.5846	1190.5891	-0.0044	0	63	3.1e-06	1		■	■	■	■	■	■	■	■				■					K.NQSALSSSIER.L
774		598.4261	1194.8376	1195.5907	-0.7530	1	6	0.24	1	U					■												K.ADGMVKDGYIK.G
784		600.8526	1199.6906	1199.6734	0.0172	1	11	0.076	1	U															■		K.LRSSLGAVQNR.F
794		606.8625	1211.7104	1211.5856	0.1248	1	4	0.44	1	U					■												K.ADGMVKDGYIK.G + Oxidation
807	1	609.7840	1217.5534	1217.5888	-0.0354	0	18	0.014	1	U														■			R.VTIDGDGTNQAK.I
813		407.5500	1219.6282	1220.6150	-0.9868	0	1	0.72	2	U															■		R.VSNQTQFNGVK.V
858		624.3705	1246.7264	1245.6565	1.0700	0	9	0.31	1	U												■					K.TALAAAGADTSGLK.L
869	1	628.8445	1255.6744	1254.6244	1.0500	0	6	0.23	1	U				■													K.FNALDAATAFSK.L
904		427.5612	1279.6618	1278.6568	1.0050	0	1	1.2	1	U							■										R.DALAASLHAEPGK.T
958		656.8636	1311.7126	1311.7146	-0.0020	0	66	2.7e-07	1	U		■															K.AQIIQQAGNSVLA.-
962		438.5371	1312.5895	1311.7146	0.8748	0	2	0.64	1	U		■															K.AQIIQQAGNSVLA.-
975		660.3423	1318.6700	1317.6929	0.9772	0	1	0.85	1	U															■		K.TLGLDNFSVAPGK.V
1016	1	672.8761	1343.7376	1343.7408	-0.0032	0	73	4.6e-08	1	U										■							-.SLSLITQNNINK.N
1069	1	463.9107	1388.7103	1387.7195	0.9908	0	13	0.048	1	U																■	K.VTVDLADAAGDLTK.T
1075		697.3253	1392.6360	1391.6205	1.0156	0	18	0.016	1	U										■							K.ASDSYSFSATAASK.D
1085		703.3437	1404.6728	1403.6391	1.0337	0	1	8	1	U													■				K.GFTVSGMADFSAAK.L + Oxidati
1088		703.8254	1405.6362	1405.7677	-0.1315	1	2	5.9	1	U															■		R.FTANIKGLTQASR.N
1135	3	720.9103	1439.8060	1439.8096	-0.0036	0	100	4.6e-10	1		■		■	■	■	■	■	■	■	■	■						K.AQIIQQAGNSVLAK.A
1155		729.3936	1456.7726	1455.8045	0.9681	0	2	1.1	1													■	■	■			K.AQIIQQAGNSVLSK.A
1162		732.8860	1463.7574	1463.7620	-0.0045	1	66	3.2e-07	1		■	■	■														K.ALDEAISSIDKFR.S
1163		488.9266	1463.7580	1463.7620	-0.0040	1	48	2.1e-05	1		■	■	■														K.ALDEAISSIDKFR.S
1179	2	739.3521	1476.6896	1476.6919	-0.0022	0	100	9.1e-11	1		■	■															K.TASVTMGGTTYNFK.T
1185		739.8825	1477.7504	1478.7002	-0.9497	0	0	0.9	1	U							■										K.TVNGSWTTNDGTVK.F
1186		493.5914	1477.7524	1478.7002	-0.9478	0	1	0.71	1	U							■										K.TVNGSWTTNDGTVK.F

Query	Dupes	Observed	Mr(expt)	Mr(calc)	Delta	M	Score	Expect	Rank	U	1	2	3	4	5	6	7	8	9	10	11	12	13	14	15	16	Peptide
1188		494.2582	1479.7528	1479.7569	-0.0042	1	24	0.0043	1		■	■															K.VTVDSGTGTGKYAPK.V
1189		740.8848	1479.7550	1479.7569	-0.0019	1	35	0.0003	1		■	■															K.VTVDSGTGTGKYAPK.V
1193	14	742.3653	1482.7160	1482.7202	-0.0042	0	114	3.6e-12	1	U	■																K.AVAADGDTSATITYK.S
1213	11	747.3457	1492.6768	1492.6794	-0.0026	0	107	1.3e-10	1		■	■															K.AASGEVNFDVDANGK.I
1221		747.3483	1492.6820	1492.6868	-0.0048	0	105	1.9e-10	1		■	■															K.TASVTMGGTTYNFK.T + Oxidati
1224	3	747.9162	1493.8178	1493.8202	-0.0023	0	60	5.5e-06	1	U	■		■	■	■	■	■	■	■		■	■	■	■			K.ANQVPQQVLSLxQG.-
1224		747.9162	1493.8178	1493.7474	0.0704	1	8	0.91	6	U															■		K.QNSTGYEKVQVGGK.D
1255		757.4182	1512.8218	1513.6896	-0.8678	1	3	0.51	1	U					■												K.DADGVYSTENKTSK.T
1262		758.9121	1515.8096	1515.8144	-0.0048	1	71	7.8e-08	1		■	■															K.ALDQLKDGDTVTIK.A
1264	1	506.2778	1515.8116	1515.8144	-0.0029	1	45	2.8e-05	1		■	■															K.ALDQLKDGDTVTIK.A
1265		759.3969	1516.7792	1515.8144	0.9648	1	4	0.37	1	U																■	K.KVTVDLADAAGDLTK.T
1270		506.9316	1517.7730	1517.7950	-0.0221	0	11	0.073	1	U									■								K.ANQVPQQVLSLHQG.-
1272		760.5572	1519.0998	1517.7950	1.3048	0	1	0.81	1	U									■								K.ANQVPQQVLSLHQG.-
1290		513.6015	1537.7827	1536.8372	0.9454	1	13	0.071	1	U									■								K.ANQVPQQVLSLRQG.-
1313	3	781.4177	1560.8208	1560.8260	-0.0052	0	74	1.7e-07	1		■	■	■	■	■	■	■		■	■	■	■		■			R.VSGQTQFNGVNVLAK
1315		521.2814	1560.8224	1560.8260	-0.0036	0	6	1.2	1		■	■	■	■	■	■	■		■	■	■	■		■			R.VSGQTQFNGVNVLAK
1353		803.3989	1604.7832	1604.7868	-0.0036	1	119	1.2e-12	1		■	■															K.KTASVTMGGTTYNFK.T
1354		535.9351	1604.7835	1604.7868	-0.0034	1	32	0.00069	1		■	■															K.KTASVTMGGTTYNFK.T
1360		807.9111	1613.8076	1613.8121	-0.0045	1	90	8.1e-09	1		■	■	■	■	■	■	■	■	■	■	■	■	■	■	■	■	R.INSAKDDAAGQAIANR.F
1361		538.9434	1613.8084	1613.8121	-0.0037	1	53	4.6e-05	1		■	■	■	■	■	■	■	■	■	■	■	■	■	■	■	■	R.INSAKDDAAGQAIANR.F
1369		541.2668	1620.7786	1620.7818	-0.0032	1	36	0.00022	1		■	■															K.KTASVTMGGTTYNFK.T + Oxidat
1370		811.3968	1620.7790	1620.7818	-0.0027	1	79	1.2e-08	1		■	■															K.KTASVTMGGTTYNFK.T + Oxidat
1391	1	823.9047	1645.7948	1644.9046	0.8902	1	14	0.043	1	U					■												K.TTANTAAKGSDILAALK.T
1414	1	836.3784	1670.7422	1670.7457	-0.0035	0	130	5.7e-13	1		■	■	■	■	■	■	■	■	■	■	■	■	■	■			R.IQDADYATEVSNMSK.A
1415		557.9216	1670.7430	1670.7457	-0.0028	0	38	0.00096	1		■	■	■	■	■	■	■	■	■	■	■	■	■	■			R.IQDADYATEVSNMSK.A
1439	8	843.4570	1684.8994	1684.8996	-0.0001	0	112	2.7e-11	1		■	■	■	■	■	■	■		■								K.IQVGANDGQTITIDLK
1441	8	843.4573	1684.9000	1685.8836	-0.9835	0	67	7.5e-07	2									■		■							K.IQVGANDGETITIDLK.K
1443		844.3745	1686.7344	1686.7407	-0.0062	0	102	4.6e-10	1		■	■	■	■	■	■	■	■	■	■	■	■	■	■			R.IQDADYATEVSNMSK.A + Oxidat
1538		596.9608	1787.8606	1787.8214	0.0392	0	6	0.25	1	U			■														K.AVYVSADGNFTTDAETK.A
1561		607.3015	1818.8827	1817.9595	0.9232	1	5	0.48	1												■		■	■			K.NQSALSTSIERLSSGLR.I
1585		618.9745	1853.9017	1854.9000	-0.9983	0	9	0.23	1	U								■									K.FDGVDISVDASTFANAVK.N
1641	2	964.9699	1927.9252	1927.9276	-0.0023	0	125	5.7e-13	1		■	■															K.SGVQTYQAVFAAGDGTASAK.Y
1642	1	643.6493	1927.9261	1927.9276	-0.0015	0	62	1.1e-06	1		■	■															K.SGVQTYQAVFAAGDGTASAK.Y
1649	2	969.9744	1937.9342	1937.9364	-0.0022	0	143	5.4e-15	1		■	■															K.MDAATNTITTTNNALTASK.A
1650		646.9856	1937.9350	1937.9364	-0.0014	0	80	1.1e-08	1		■	■															K.MDAATNTITTTNNALTASK.A
1658		977.9712	1953.9278	1953.9313	-0.0035	0	165	3.5e-17	1		■	■															K.MDAATNTITTTNNALTASK.A + O
1672	1	987.0193	1972.0240	1972.0266	-0.0025	0	94	4.2e-10	1	U	■																K.AGDVAASLLPPAGQTASGVYK.A
1673		658.3489	1972.0249	1972.0266	-0.0017	0	79	1.3e-08	1	U	■																K.AGDVAASLLPPAGQTASGVYK.A
1677		659.6340	1975.8802	1975.9997	-0.1195	1	18	0.017	1	U								■									K.DMVGLKLDNTGVTTAGVNR.Y + O
1712	7	1021.4690	2040.9234	2040.9236	-0.0002	0	112	5.6e-12	1		■	■															K.TGADADAATANAGVSFTDTASK.E
1723	1	1043.0670	2084.1194	2084.1225	-0.0031	0	126	1.6e-12	1		■		■	■	■	■	■	■	■			■		■	■	■	M.AQVINTNSLSLiTQNNiNK.N
1723	1	1043.0670	2084.1194	2085.1066	-0.9871	0	114	2.4e-11	4	U											■						M.AQVINTNSLSLITQNNIDK.N
1723	1	1043.0670	2084.1194	2085.0814	-0.9620	0	111	4.9e-11	5	U													■				M.AQVINTNSLSLNTQNNINK.N
1725	1	695.7142	2084.1208	2085.1066	-0.9858	0	60	6.3e-06	1	U											■						M.AQVINTNSLSLITQNNIDK.N
1725	1	695.7142	2084.1208	2085.0814	-0.9606	0	55	2.3e-05	2	U													■				M.AQVINTNSLSLNTQNNINK.N
1725	1	695.7142	2084.1208	2084.1225	-0.0018	0	52	4.2e-05	3		■		■	■	■	■	■	■	■			■		■	■	■	M.AQVINTNSLSLiTQNNiNK.N
1764		1095.5360	2189.0574	2190.0917	-1.0342	1	3	0.58	1		■	■															K.AASGEVNFDVDANGKITIGGQK.A
1765		731.0412	2190.1018	2190.0917	0.0101	1	11	0.097	1		■	■															K.AASGEVNFDVDANGKITIGGQK.A
1774	1	1104.0480	2206.0814	2206.0866	-0.0051	0	121	1.8e-12	1		■	■															K.IDSDTLGLNGFNVNGSGTIANK.A
1800	2	1125.0510	2248.0874	2248.0931	-0.0057	0	136	1.4e-13	1		■	■	■	■	■	■	■	■		■	■	■	■				R.LDSAVTNLNNTTTNLSEAQSR.I
1801		750.3698	2248.0876	2248.0931	-0.0055	0	113	3.1e-11	1		■	■	■	■	■	■	■	■		■	■	■	■				R.LDSAVTNLNNTTTNLSEAQSR.I
1803		753.7324	2258.1754	2258.2019	-0.0265	1	1	0.8	1	U											■						R.LAEIDRVSGQTQFNGVNVLAK.N
1807		761.7309	2282.1709	2282.0550	0.1159	0	12	0.069	1	U								■									K.DGSLTAADDGAALYLDDTGNLSK.T
1811		763.0187	2286.0343	2286.0434	-0.0091	1	2	0.57	1	U										■							K.DMTITSAGGNAQVATDKAYNDK.Y
1821		768.4033	2302.1881	2302.1917	-0.0037	1	70	4.7e-07	1		■	■	■	■	■	■	■		■	■				■			R.LDEIDRVSGQTQFNGVNVLAK.D
1834		773.3906	2317.1500	2318.1866	-1.0367	1	7	0.4	2									■					■				R.LDEIDRVSGQTQFNGVNVLSK
1834		773.3906	2317.1500	2316.2074	0.9426	1	2	1.4	3	U												■					R.LEEIDRVSGQTQFNGVNVLAK.D
1841		1168.0950	2334.1754	2334.1815	-0.0061	1	171	7.8e-18	1		■	■															K.KIDSDTLGLNGFNVNGSGTIANK.A
1843	3	779.0666	2334.1780	2334.1815	-0.0036	1	70	9.3e-08	1		■	■															K.KIDSDTLGLNGFNVNGSGTIANK.A
1873		820.3927	2458.1563	2459.2867	-1.1305	1	0	0.91	2	U												■					K.TALAAAGADTSGLKLVQLSNTDSAG
1874		1236.1100	2470.2054	2470.2075	-0.0021	0	122	6.6e-13	1		■	■															K.VGAEVYVSANGTLTTDATSEGTVTK
1888	1	856.0710	2565.1912	2565.2293	-0.0382	0	29	0.0036	3	U												■					R.ELTVQATTGTNSESDLSSIQDEIK.
1889	3	1283.6030	2565.1914	2565.1930	-0.0015	0	130	3e-13	1		■	■	■	■	■												R.ELTVQASTGTNSDSDLDSIQDEIK.
1889	2	1283.6030	2565.1914	2565.2293	-0.0379	0	19	0.042	3	U												■					R.ELTVQATTGTNSESDLSSIQDEIK.
1890	1	856.0714	2565.1924	2565.1930	-0.0006	0	75	8.6e-08	1		■	■	■	■	■												R.ELTVQASTGTNSDSDLDSIQDEIK.
1901	1	877.0976	2628.2710	2628.2739	-0.0029	0	78	8.1e-08	1		■	■	■	■	■	■		■	■	■					■		R.NANDGISVAQTTEGALSEINNNLQR
1902	3	1315.1430	2628.2714	2628.2739	-0.0025	0	130	5.1e-13	1		■	■	■	■	■	■		■	■	■					■		R.NANDGISVAQTTEGALSEINNNLQR
1915	5	886.7668	2657.2786	2657.2821	-0.0035	0	115	3e-12	1		■	■															K.AYLTSDGNLTTNDAGGATAATLDGL
1918	2	1329.6480	2657.2814	2657.2821	-0.0006	0	144	3.6e-15	1		■	■															K.AYLTSDGNLTTNDAGGATAATLDGL
1931	3	895.3991	2683.1755	2683.1807	-0.0052	0	80	9.7e-09	1		■	■															K.ADVSNATATYTDADGEMTTIGSYTT
1937	5	1342.5980	2683.1814	2683.1807	0.0008	0	122	6.7e-13	1		■	■															K.ADVSNATATYTDADGEMTTIGSYTT
1944		909.4399	2725.2979	2725.3042	-0.0064	1	104	4.3e-11	1		■	■															K.TGADADAATANAGVSFTDTASKETV
1951	1	929.4655	2785.3747	2785.3770	-0.0023	1	80	1.8e-08	1		■	■															K.AYLTSDGNLTTNDAGGATAATLDGL
1954		945.7986	2834.3740	2834.3781	-0.0042	1	86	8e-09	1		■	■	■	■	■												R.IRELTVQASTGTNSDSDLDSIQDEI
1964		966.8613	2897.5621	2897.4591	0.1030	1	0	5	1								■				■	■		■			R.NANDGISLAQTTEGALSEINNNLQR
1967		972.8801	2915.6185	2916.5781	-0.9596	1	48	7.3e-05	1	U									■								K.AQIIQQAGNSVLAKANQVPQQVLSL
1967		972.8801	2915.6185	2915.6192	-0.0008	1	43	0.00023	2		■		■	■	■	■	■	■			■						K.AQIIQQAGNSVLAKANQVPQQVLSL
1971	3	975.4972	2923.4698	2923.4662	0.0035	1	53	8.2e-06	1		■	■															K.VGAEVYVSANGTLTTDATSEGTVTK
1987		1031.8560	3092.5462	3092.5448	0.0014	1	95	1.4e-09	1		■		■	■	■	■	■	■	■	■	■						R.IQDADYATEVSNMSKAQIIQQAGNS
1997	4	1054.4740	3160.4002	3160.4030	-0.0029	1	101	2.5e-10	1		■	■															K.YADKADVSNATATYTDADGEMTTIG
1999	2	1058.1500	3171.4282	3171.4381	-0.0099	1	107	1.8e-11	1	U	■																K.ADAAQTATVYTYNASAGNFSFSNVS
2002		1059.2030	3174.5872	3174.5865	0.0007	1	107	5e-11	1		■	■		■	■	■	■	■									R.SSLGAIQNRLDSAVTNLNNTTTNLS
2006		1086.5740	3256.7002	3256.7011	-0.0009	1	114	1.6e-11	1		■		■	■	■	■	■	■				■					M.AQVINTNSLSLITQNNINKNQSALS

Query	Dupes	Observed	Mr(expt)	Mr(calc)	Delta	M	Score	Expect	Rank	U	1	2	3	4	5	6	7	8	9	10	11	12	13	14	15	16	Peptide
2040		1125.9390	3374.7952	3374.7570	0.0382	1	11	0.089	1	U											■						K.IDSSALGLSGFSVAGGALKLSDTVT

60 subsets and intersections (146 subset proteins in total)

2	gi|46093564	20	Hxx(H54 100.0%) 0|0|E223-69 [gi|283982455|ref|ADB56974| H54 0|O161:H54|O161:H54]
3	gi|112820172	18	H21 0|EHEC serogroup: O113:H21|0

10 per page 1

Not what you expected? Try the select summary.

Mascot: http://www.matrixscience.com/
