# Supplementary material for: MS-H: A Novel Proteomic Approach to Isolate and Type the E. coli H Antigen Using Membrane Filtration and Liquid Chromatography-Tandem Mass Spectrometry (LC-MS/MS)
Source: PLoS One. 2013 Feb 21;8(2):e57339. doi: 10.1371/journal.pone.0057339 (PMC3578835; doi:10.1371/journal.pone.0057339)
Supplement: Representative Peptide Data S1 — Peptide data are represented as the Mascot search results from all 53 serotypes, obtained under the Orbitrap platform in Table 4 with related E. coli reference strains. “U” denotes a unique peptide specific for each of the proteins 1.1, 1.2, and beyond. The number 1.1 (shown as 1 in the peptide list and phylogenetic tree) represents the protein which obtained the highest score and confidence value after a Mascot search. This protein, known as the first hit, was used to designate the MS-H type of the unknown flagellin. Related peptides 1.2 (2), 1.3 (3), etc. represented the second, third, etc. hits for MS-H typing analysis. (DOCX) [file pone.0057339.s009.docx › H14-E182.pdf]

MATRIX SCIENCE MASCOT Search Results

User :
E-mail :
Search title : Submitted from 20110728-h11-21 by Mascot Daemon on VARIABLE
MS data file : C:\Documents and Settings\keding\Desktop\Raw data\20110727-h11-21\20110728-022-E182MS1.RAW
Database : Flagellin_v2 (192 sequences; 89,845 residues)
Taxonomy : Bacteria (Eubacteria) (192 sequences)
Timestamp : 29 Jul 2011 at 14:09:36 GMT

Not what you expected? Try **the select summary**.

Search parameters

Score distribution

Legend

Protein Family Summary

Significance threshold p< 0.05 Max. number of families AUTO
Ions score or expect cut-off 0 Dendrograms cut at 0

Protein family 1 (out of 1)

10 per page 1

1

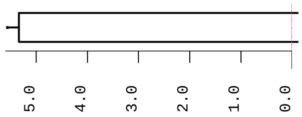

1 gi|6941882 998 H14 0|O138:H14|O138:H14
2 gi|33590243 229 H16 NM|O157:NM(H16)|PC02

Threshold (**0**): 0

		Score	Mass	Matches	Sequences	emPAI
**1.1**	**gi|6941882**	998	56492	40 (21)	28 (18)	2.10
	H14 0|O138:H14|O138:H14					
	**1 sameset of gi|6941882**					
**1.2**	**gi|33590243**	229	55093	16 (6)	11 (5)	0.34
	H16 NM|O157:NM(H16)|PC02					
	**2 samesets of gi|33590243**					

48 peptide matches (39 non-duplicate, 9 duplicate)

Query	Dupes	Observed	Mr(expt)	Mr(calc)	Delta	M	Score	Expect	Rank	U	1	2	Peptide
**27**	1	**316.6911**	**631.3676**	**631.3653**	**0.0023**	**0**	**24**	**0.037**	1		■	■	**R.LSSGLR.I**
93		355.1986	708.3826	708.3806	0.0020	0	6	0.47	1	U	■		R.FTSNIK.G
**97**		**358.7073**	**715.4000**	**715.3977**	**0.0024**	**0**	**32**	**0.0041**	1	**U**	■		**K.GLTQAAR.N**
108		366.2271	730.4396	731.3813	-0.9417	0	0	3.5	2	U		■	R.LSEIDR.V
**130**		**380.2047**	**758.3948**	**758.4174**	**-0.0225**	**0**	**25**	**0.017**	1	**U**		■	**K.LDEALAK.V**
**135**	3	**380.6965**	**759.3784**	**759.3763**	**0.0022**	**0**	**27**	**0.013**	1	**U**	■		**R.LDEIDR.V**
335	1	439.3085	876.6024	876.4552	0.1472	0	13	0.053	1	U	■		K.AATTADSLK.A
337		439.7281	877.4416	877.4393	0.0024	0	10	0.1	1	U	■		K.LTTDAETK.A
352		447.7201	893.4256	892.4290	0.9966	0	2	0.61	1	U	■		K.VDQAAFDK.A
372		452.3161	902.6176	903.4661	-0.8485	0	2	0.73	1	U	■		K.AATTADNLK.A
374		452.7356	903.4566	904.4502	-0.9935	0	6	0.37	1	U	■		K.AATTADDLK.A
**429**	3	**466.7567**	**931.4988**	**930.4883**	**1.0106**	**0**	**74**	**1.8e-07**	1		■	■	**R.SSLGAVQNR**
**444**		**469.7515**	**937.4884**	**937.4869**	**0.0016**	**0**	**36**	**0.00028**	1	**U**	■		**K.LYIDTTGR.L**
515		483.8502	965.6858	965.4818	0.2041	1	7	0.2	2	U	■		K.AVYKDADGK.L
610		502.2408	1002.4670	1002.5094	-0.0424	1	5	0.44	1	U	■		K.SRLDEIDR.V
648		508.7748	1015.5350	1014.5709	0.9641	0	1	0.74	1	U		■	K.ALATTNPLSK.L
757	1	539.2704	1076.5262	1077.4873	-0.9610	0	9	0.16	1	U		■	K.NDGSAQAIMR.E + Oxidation (M)
**818**		**551.2685**	**1100.5224**	**1100.5210**	**0.0014**	**0**	**69**	**1.1e-06**	1		■	■	**K.DDAAGQAIANR.F**
821		551.7571	1101.4996	1100.6077	0.8919	1	1	7.7	1	U		■	K.LDEALAKVDK.L
**858**		**561.2964**	**1120.5782**	**1120.5764**	**0.0018**	**0**	**55**	**3.4e-06**	1	**U**	■		**K.YLTPGVGDTAK.G**
**918**		**573.2781**	**1144.5416**	**1144.5400**	**0.0016**	**0**	**44**	**0.00034**	1	**U**	■		**K.YTVSAGYDAAK.A**
**966**		**581.3041**	**1160.5936**	**1160.5925**	**0.0012**	**0**	**82**	**8.2e-09**	1	**U**	■		**K.ALDEAISSIDK.F**
**1015**		**596.3030**	**1190.5914**	**1190.5891**	**0.0024**	**0**	**36**	**0.0015**	1	**U**	■		**K.NQSALSSSIER.L**
1039		600.8537	1199.6928	1199.6734	0.0194	1	3	0.53	1	U		■	K.LRSSLGAVQNR.F
1297		448.2495	1341.7267	1341.7252	0.0015	0	0	0.97	1	U	■		K.ADLVAANATVVGNK.Y
**1298**		**671.8708**	**1341.7270**	**1341.7252**	**0.0018**	**0**	**73**	**5e-08**	1	**U**	■		**K.ADLVAANATVVGNK.Y**
**1424**		**720.9129**	**1439.8112**	**1439.8096**	**0.0016**	**0**	**101**	**3.2e-10**	1	**U**	■		**K.AQIIQQAGNSVLAK.A**
**1453**		**488.9282**	**1463.7628**	**1463.7620**	**0.0008**	**1**	**32**	**0.00095**	1	**U**	■		**K.ALDEAISSIDKFR.S**

Query	Dupes	Observed	Mr(expt)	Mr(calc)	Delta	M	Score	Expect	Rank	U	1	2	Peptide
1488		747.9187	1493.8228	1493.8202	0.0027	0	19	0.078	1	U	■		K.ANQVPQQVLSLLQG.-
1575		781.4215	1560.8284	1560.8260	0.0024	0	71	4e-07	1	U	■		R.VSGQTQFNGVNVLAK.D
1693		836.3813	1670.7480	1670.7457	0.0023	0	123	2.7e-12	1	U	■		R.IQDADYATEVSNMSK.A
1713		844.3790	1686.7434	1686.7407	0.0028	0	48	0.00011	1	U	■		R.IQDADYATEVSNMSK.A + Oxidation (M)
1721		846.3931	1690.7716	1690.7686	0.0030	0	130	9.4e-14	1	U	■		K.SYSFDTTTASAADVQK.Y
1722		564.6042	1690.7908	1690.7686	0.0222	0	1	0.85	1	U	■		K.SYSFDTTTASAADVQK.Y
1734		854.1137	1706.2128	1705.8734	0.3394	1	4	0.45	1	U	■		K.LTTDAETKAATTADGLK.A
1749		860.3588	1718.7030	1718.7974	-0.0943	0	1	0.73	1	U		■	K.ALAYNDAPMSVYFGGK.N + Oxidation (M)
1949		1043.0700	2084.1254	2084.1225	0.0029	0	69	8.8e-07	1	U	■	■	M.AQVINTNSLSLiTQNNiNK.N
1987		750.3724	2248.0954	2248.0931	0.0023	0	59	8.2e-06	1	U	■		R.LDSAVTNLNNTTTNLSEAQSR.I
1988		1125.0550	2248.0954	2248.0931	0.0023	0	111	5e-11	1	U	■		R.LDSAVTNLNNTTTNLSEAQSR.I

46 subsets and intersections (166 subset proteins in total)

10 per page 1

Not what you expected? Try **the select summary**.

Mascot: http://www.matrixscience.com/
