# Supplementary material for: MS-H: A Novel Proteomic Approach to Isolate and Type the E. coli H Antigen Using Membrane Filtration and Liquid Chromatography-Tandem Mass Spectrometry (LC-MS/MS)
Source: PLoS One. 2013 Feb 21;8(2):e57339. doi: 10.1371/journal.pone.0057339 (PMC3578835; doi:10.1371/journal.pone.0057339)
Supplement: Representative Peptide Data S1 — Peptide data are represented as the Mascot search results from all 53 serotypes, obtained under the Orbitrap platform in Table 4 with related E. coli reference strains. “U” denotes a unique peptide specific for each of the proteins 1.1, 1.2, and beyond. The number 1.1 (shown as 1 in the peptide list and phylogenetic tree) represents the protein which obtained the highest score and confidence value after a Mascot search. This protein, known as the first hit, was used to designate the MS-H type of the unknown flagellin. Related peptides 1.2 (2), 1.3 (3), etc. represented the second, third, etc. hits for MS-H typing analysis. (DOCX) [file pone.0057339.s009.docx › H15-E183.pdf]

MATRIX SCIENCE MASCOT Search Results

User :
E-mail :
Search title : Submitted from 20110728-h11-21 by Mascot Daemon on VARIABLE
MS data file : C:\Documents and Settings\keding\Desktop\Raw data\20110727-h11-21\20110728-023-E183MS1.RAW
Database : Flagellin_v2 (192 sequences; 89,845 residues)
Taxonomy : Bacteria (Eubacteria) (192 sequences)
Timestamp : 29 Jul 2011 at 14:11:22 GMT

Not what you expected? Try **the select summary**.

▶**Search parameters**
▶**Score distribution**
▶**Legend**

Protein Family Summary

Significance threshold p< 0.05 Max. number of families AUTO
Ions score or expect cut-off 0 Dendrograms cut at 0

Protein family 1 (out of 1)

10 per page 1

▼1

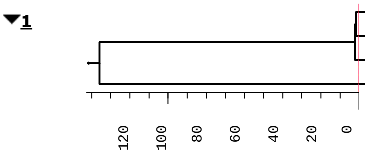

1	gi|30059870	1783	H15 0|H15|E 39a
2	gi|30059928	789	H52 0|H52|C2187-69
4	gi|33590252	194	Hxx(H8 89.5%) 0|0|PC41
3	gi|121144335	751	H55 0|O75:H55|E2987-73

Threshold (**0**): 0

		Score	Mass	Matches	Sequences	emPAI
☑ 1.1	gi|30059870<br>H15 0|H15|E 39a	1783	57416	50 (36)	31 (29)	5.30
☑ 1.2	gi|30059928<br>H52 0|H52|C2187-69	789	46003	29 (19)	16 (14)	2.49
☑ 1.3	gi|121144335<br>H55 0|O75:H55|E2987-73	751	62285	27 (16)	15 (12)	1.16
☑ 1.4	gi|33590252<br>Hxx(H8 89.5%) 0|0|PC41	194	52373	29 (6)	8 (5)	0.44

▼76 peptide matches (47 non-duplicate, 29 duplicate)

Query	Dupes	Observed	Mr(expt)	Mr(calc)	Delta	M	Score	Expect	Rank	U	1	2	3	4	Peptide
18	▶1	316.6910	631.3674	631.3653	0.0021	0	25	0.031	▶1		■	■	■	■	R.LSSGLR.I
46		332.7061	663.3976	663.3955	0.0021	0	15	0.029	▶1	U	■				K.LYLQK.D
73		351.7061	701.3976	700.4119	0.9857	1	2	0.9	▶2	U				■	K.DPTKLK.A
84		355.1987	708.3828	708.3806	0.0022	0	11	0.48	▶1		■	■	■		R.FTSNIK.G
86		358.7072	715.3998	715.3977	0.0022	0	34	0.0032	▶1		■	■	■		K.GLTQAAR.N
97	▶1	366.2270	730.4394	731.3813	-0.9419	0	5	1	▶2	U				■	R.LSEIDR.V
118	▶1	380.6963	759.3780	759.3763	0.0018	0	37	0.0011	▶1		■	■	■		R.LDEIDR.V
142		395.2092	788.4038	788.4028	0.0010	0	20	0.01	▶1	U	■				K.AGDTANLK.V
259	▶17	421.7593	841.5040	841.4658	0.0383	0	15	0.032	▶1	U				■	K.AVTQPQAK.D
388		459.2512	916.4878	916.4866	0.0013	0	46	2.4e-05	▶1	U	■				K.AATTTDPLK.A
403		466.7440	931.4734	930.4883	0.9852	0	3	2.4	▶1			■	■		R.SSLGAVQNR.L
436	▶1	473.2599	944.5052	944.5039	0.0013	0	73	1.4e-07	▶1	U	■				R.SSLGAIQNR.L
564	▶1	502.2628	1002.5110	1002.5094	0.0016	1	29	0.0077	▶1		■	■	■		K.SRLDEIDR.V
565		335.1783	1002.5131	1002.5094	0.0037	1	14	0.24	▶1		■	■	■		K.SRLDEIDR.V
723	▶1	551.2682	1100.5218	1100.5210	0.0008	0	53	4.8e-05	▶1		■	■	■	■	K.DDAAGQAIANR.F
759		560.7963	1119.5780	1119.5771	0.0009	0	86	2.3e-09	▶1	U	■				R.ISADSLQSATK.S
825	▶1	576.7733	1151.5320	1151.5306	0.0014	0	12	0.06	▶1	U	■				K.DGSLTTDNTTK.L
889		397.2044	1188.5914	1187.6034	0.9880	0	3	0.54	▶1	U			■		K.ALDDAISQIDK.F
892		397.5594	1189.6564	1190.5891	-0.9327	0	1	4.1	▶2		■	■			K.NQSALSSSIER.L
893		596.3024	1190.5902	1190.5891	0.0012	0	43	0.00027	▶1		■	■			K.NQSALSSSIER.L
916		601.8177	1201.6208	1201.6190	0.0018	0	90	1.1e-09	▶1	U	■				K.ALDEAISQIDK.F
922		602.8256	1203.6366	1203.6347	0.0020	0	81	1.6e-08	▶1	U	■				K.ESTAADVIASIK.D
1139		672.8782	1343.7418	1343.7408	0.0010	0	62	6.6e-07	▶1	U			■		-.SLSLITQNNINK.N
1213		707.3668	1412.7190	1412.7147	0.0043	0	97	1.9e-10	▶1	U	■				K.VTIGGTTAQSYTSK.D
1253		480.6095	1438.8067	1439.8096	-1.0029	0	1	4.1	▶1		■		■		K.AQIIQQAGNSVLAK.A
1254		720.9130	1439.8114	1439.8096	0.0018	0	120	4e-12	▶1		■		■		K.AQIIQQAGNSVLAK.A
1278		728.5283	1455.0420	1453.7161	1.3259	1	2	0.91	▶1	U				■	K.HDNVKVELGGSDGK.T
1315		747.9185	1493.8224	1493.8202	0.0023	0	51	5.4e-05	▶1		■	■			K.ANQVPQQVLSLLQG.-

Query	Dupes	Observed	Mr(expt)	Mr(calc)	Delta	M	Score	Expect	Rank	U	1	2	3	4	Peptide
1332		502.6043	1504.7911	1504.7885	0.0025	1	42	5.8e-05	▶1	U	■				K.ALDEAISQIDKFR.S
1372		766.8845	1531.7544	1530.7777	0.9767	0	15	0.035	▶1	U		■			K.IQLTDELDVDGSVK.T
1402	▶2	781.4208	1560.8270	1560.8260	0.0010	0	72	2.9e-07	▶1		■	■	■		R.VSGQTQFNGVNVLAK.D
1461		538.9453	1613.8141	1613.8121	0.0020	1	28	0.015	▶1		■	■	■	■	R.INSAKDDAAGQAIANR.F
1505	▶1	824.4450	1646.8754	1646.8727	0.0027	0	99	1.2e-10	▶1	U	■				K.VTVGTTSVDVVLASDGK.I
1526		557.9227	1670.7463	1670.7457	0.0005	0	5	0.92	▶1		■	■	■		R.IQDADYATEVSNMSK.A
1527		836.3809	1670.7472	1670.7457	0.0015	0	114	2.4e-11	▶1		■	■	■		R.IQDADYATEVSNMSK.A
1551		844.3793	1686.7440	1686.7407	0.0034	0	83	3.5e-08	▶1		■	■	■		R.IQDADYATEVSNMSK.A + Oxidation (M)
1699	▶1	913.9611	1825.9076	1825.9058	0.0018	0	95	3e-10	▶1	U	■				K.STGFTVGTGATGLTVGTDGK.V
1820		1043.0700	2084.1254	2084.1225	0.0029	0	102	4.5e-10	▶1		■	■		■	M.AQVINTNSLSLiTQNNiNK.N
1821		695.7159	2084.1259	2084.1225	0.0033	0	73	3.3e-07	▶1		■	■		■	M.AQVINTNSLSLiTQNNiNK.N
1892		750.3726	2248.0960	2248.0931	0.0029	0	76	1.7e-07	▶1		■	■	■		R.LDSAVTNLNNTTTNLSEAQSR.I
1893		1125.0560	2248.0974	2248.0931	0.0043	0	141	5.1e-14	▶1		■	■	■		R.LDSAVTNLNNTTTNLSEAQSR.I
1933		1192.5830	2383.1514	2383.1503	0.0011	0	109	1.3e-11	▶1	U	■				K.DGSALYIDSTGNLTQNSAGLTSAK.L
1956		1297.1300	2592.2454	2592.2402	0.0052	0	64	3.8e-07	▶1	U	■				R.ELTVQATTGTNSQSDLDSIQDEIK.S
1959		877.0999	2628.2779	2628.2739	0.0040	0	29	0.0053	▶1		■	■	■		R.NANDGISVAQTTEGALSEINNNLQR
1960		1315.1470	2628.2794	2628.2739	0.0055	0	117	8.6e-12	▶1		■	■	■		R.NANDGISVAQTTEGALSEINNNLQR
1971	▶1	889.7922	2666.3548	2666.3511	0.0036	1	93	4.8e-10	▶1	U	■				K.KIDSSTLNLTGFNVNGSGSVANTAATK.A
2013		1141.5250	3421.5532	3421.5434	0.0098	0	58	1.6e-06	▶1	U	■				K.DGSAPTSAITATINNGFGDSSALTSNDYTYDPAK.G

▶51 subsets and intersections (167 subset proteins in total)

10 per page 1

Not what you expected? Try the select summary.

Mascot: http://www.matrixscience.com/
