# Supplementary material for: MS-H: A Novel Proteomic Approach to Isolate and Type the E. coli H Antigen Using Membrane Filtration and Liquid Chromatography-Tandem Mass Spectrometry (LC-MS/MS)
Source: PLoS One. 2013 Feb 21;8(2):e57339. doi: 10.1371/journal.pone.0057339 (PMC3578835; doi:10.1371/journal.pone.0057339)
Supplement: Representative Peptide Data S1 — Peptide data are represented as the Mascot search results from all 53 serotypes, obtained under the Orbitrap platform in Table 4 with related E. coli reference strains. “U” denotes a unique peptide specific for each of the proteins 1.1, 1.2, and beyond. The number 1.1 (shown as 1 in the peptide list and phylogenetic tree) represents the protein which obtained the highest score and confidence value after a Mascot search. This protein, known as the first hit, was used to designate the MS-H type of the unknown flagellin. Related peptides 1.2 (2), 1.3 (3), etc. represented the second, third, etc. hits for MS-H typing analysis. (DOCX) [file pone.0057339.s009.docx › H16-E184.pdf]

MASCOT Search Results

User :
E-mail :
Search title : Submitted from 20110728-h11-21 by Mascot Daemon on VARIABLE
MS data file : C:\Documents and Settings\keding\Desktop\Raw data\20110727-h11-21\20110728-024-E184MS1.RAW
Database : Flagellin_v2 (192 sequences; 89,845 residues)
Taxonomy : Bacteria (Eubacteria) (192 sequences)
Timestamp : 29 Jul 2011 at 14:12:59 GMT

Not what you expected? Try **the select summary**.

- **Search parameters**
- **Score distribution**
- **Legend**

Protein Family Summary

Significance threshold p< 0.05 Max. number of families AUTO
Ions score or expect cut-off 0 Dendrograms cut at 0

Protein family 1 (out of 1)

10 per page 1

1

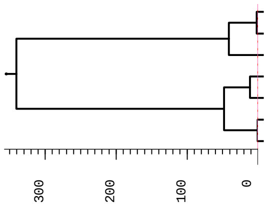

1	gi|33590243	1453	H16 NM|O157:NM(H16)|PC02
2	gi|112820171	583	H21 0|EHEC serogroup: O113:H21|0 [gi|291323135|ref|EFE62563| Hxx 0|0|B088] [gi|29344630...
4	gi|46093552	361	H53 0|0|E480-68
6	gi|323174561	258	Hxx(H25 99.7%) 0|0|LT-68
7	gi|290918477	167	Hxx(H52 80.8%) 0|0|B2N
3	gi|30059870	507	H15 0|H15|E 39a
5	gi|6009849	285	H49 0|H49|U11a-44

Threshold (**0**): 0

		Score	Mass	Matches	Sequences	emPAI
1.1	gi|33590243	1453	55093	51 (37)	34 (29)	7.09
	H16 NM|O157:NM(H16)|PC02					
	**2 samesets of gi|33590243**					
1.2	gi|112820171	583	51472	16 (11)	10 (8)	0.98
	H21 0|EHEC serogroup: O113:H21|0 [gi|291323135|ref|EFE62563| Hxx 0|0|B088] [gi|293446305|ref|ZP_06662727| Hxx 0|0|B088]					
	**7 samesets of gi|112820171**					
1.3	gi|30059870	507	57416	20 (13)	16 (13)	1.06
	H15 0|H15|E 39a					
1.4	gi|46093552	361	44861	14 (7)	11 (7)	0.64
	H53 0|0|E480-68					
1.5	gi|6009849	285	58493	19 (8)	15 (8)	0.55
	H49 0|H49|U11a-44					
1.6	gi|323174561	258	46392	17 (6)	13 (6)	0.51
	Hxx(H25 99.7%) 0|0|LT-68					
	**1 sameset of gi|323174561**					
1.7	gi|290918477	167	42592	12 (6)	8 (6)	0.57
	Hxx(H52 80.8%) 0|0|B2N					

87 peptide matches (73 non-duplicate, 14 duplicate)

Query	Dupes	Observed	Mr(expt)	Mr(calc)	Delta	M	Score	Expect	Rank	U	1	2	3	4	5	6	7	Peptide
18		316.6909	631.3672	631.3653	0.0019	0	21	0.07	1		■	■	■	■	■	■	■	R.LSSGLR.I
21		317.6880	633.3614	632.3129	1.0485	0	3	0.54	1	U				■				R.ANVDSK.T
48		337.2170	672.4194	673.3759	-0.9564	0	1	0.72	1	U						■		K.NGATALK.L
60		347.2014	692.3882	692.3857	0.0025	0	14	0.09	1		■	■						R.FTANIK.G
89	1	366.6991	731.3836	731.3813	0.0023	0	24	0.013	1	U	■							R.LSEIDR.V
90	2	366.7048	731.3950	731.3926	0.0025	0	24	0.014	1		■	■		■				K.GLTQASR.N
108	2	380.2168	758.4190	758.4174	0.0017	0	31	0.005	1	U	■							K.LDEALAK.V
113	1	380.6964	759.3782	759.3763	0.0020	0	30	0.0051	1				■		■	■	■	R.LDEIDR.V
129		393.6934	785.3722	785.3708	0.0015	0	22	0.006	1	U	■							K.AFGSNYK.N
130		393.7276	785.4406	786.4599	-1.0193	0	4	0.44	1	U						■		K.QTGQLIK.V
157		401.7268	801.4390	801.4385	0.0006	0	40	9.4e-05	1	U	■							K.AVEWAVK.N
240		423.2227	844.4308	844.4402	-0.0094	0	13	0.046	1	U					■			K.AAAGAESIR.Y
344	2	452.2202	902.4258	902.5073	-0.0814	0	21	0.0076	1	U							■	K.IDSSALGLK.G
383		466.2406	930.4666	930.4658	0.0008	0	27	0.0082	1	U	■							K.SEAAPDLTK.V
384	1	466.2517	930.4888	930.4883	0.0006	0	59	6.1e-06	1		■					■	■	R.SSLGAVQNR
414		473.2547	944.4948	944.5039	-0.0091	0	57	6.5e-06	1				■	■	■			R.SSLGAIQNR
524		495.7593	989.5040	989.5029	0.0011	0	63	6.8e-07	1	U	■							K.GAELSASDLK.A
529		496.7585	991.5024	991.5008	0.0016	0	38	0.00018	1	U	■							K.VLASDQTMK.I
552		501.7865	1001.5584	1001.5579	0.0005	0	42	0.00036	1	U	■							K.VPMSSAVALK.S
564		504.3582	1006.7018	1005.5607	1.1411	1	4	0.5	1							■	■	K.AIASVDKFR.S
566		504.7555	1007.4964	1007.4957	0.0007	0	44	3.7e-05	1	U	■							K.VLASDQTMK.I + Oxidation (M)

Query	Dupes	Observed	Mr(expt)	Mr(calc)	Delta	M	Score	Expect	Rank	U	1	2	3	4	5	6	7	Peptide
584		508.2933	1014.5720	1014.5709	0.0011	0	42	5.8e-05	1	U	■							K.ALATTNPLSK.L
604		512.2779	1022.5412	1022.5396	0.0016	0	56	2.3e-06	1	U	■							K.VYTANITNK.T
661		531.7545	1061.4944	1061.4924	0.0021	0	81	2.1e-08	1	U	■							K.NDGSAQAIMR.E
670		538.2728	1074.5310	1074.5306	0.0005	0	34	0.00059	1	U	■							K.VNATDGSVGGAK.A
673		538.7776	1075.5406	1075.5411	-0.0004	0	39	0.00018	1	U	■							K.DVQLANFGGR.V
677	3	539.7515	1077.4884	1077.4873	0.0012	0	48	1.7e-05	1	U	■							K.NDGSAQAIMR.E + Oxidation (M)
729		551.2685	1100.5224	1100.5210	0.0014	0	70	8.8e-07	1		■	■	■	■	■	■	■	K.DDAAGQAIANR.F
758		560.7962	1119.5778	1119.5771	0.0007	0	59	1.3e-06	1	U			■					R.ISADSLQSATK.S
802		569.7664	1137.5182	1137.5150	0.0033	0	33	0.00048	1	U	■							K.DTTDATGTAGTK.V
804		570.2835	1138.5524	1137.5666	0.9858	0	15	0.031	1	U						■		K.ATADYVVQSGK.D
811		381.1620	1140.4642	1141.5655	-1.1014	0	2	0.69	2	U				■				K.VTEFQEVYK.K
834		576.7732	1151.5318	1151.5306	0.0012	0	16	0.024	1	U			■					K.DGSLTTDNTTK.L
858		582.7974	1163.5802	1163.5782	0.0020	0	67	6.8e-07	1		■	■		■				K.SQSSLSSAIER.L
888		587.8271	1173.6396	1173.5778	0.0618	1	14	0.037	1	U				■				K.DKDGAYHAAVK.N
912		596.3019	1190.5892	1190.5891	0.0002	0	23	0.027	1				■		■		■	K.NQSALSSSIER.L
922		598.8018	1195.5890	1194.5517	1.0374	0	7	0.22	1	U					■			K.DAAQSSIDFGGK.K
940		600.8531	1199.6916	1199.6734	0.0182	1	3	0.47	1	U	■							K.LRSSLGAVQNR.F
944		601.8178	1201.6210	1201.6190	0.0020	0	64	3.6e-07	1	U			■					K.ALDEAISQIDK.F
948		603.3250	1204.6354	1203.6347	1.0008	0	22	0.013	1	U			■					K.ESTAADVIASIK.D
965		611.3161	1220.6176	1220.6150	0.0027	0	49	1.3e-05	1	U	■							R.VSNQTQFNGVK.V
1012		624.3721	1246.7296	1247.6734	-0.9438	1	3	1.2	1				■		■			K.FRSSLGAIQNR.L
1117		651.8616	1301.7086	1301.6827	0.0260	0	18	0.037	1	U		■						K.AATLSDLDLNAAK.K
1151		659.8544	1317.6942	1317.6929	0.0014	0	74	3.8e-08	1	U	■							K.TLGLDNFSVAPGK.V
1155		441.5261	1321.5565	1322.6466	-1.0902	1	1	0.87	1	U					■			K.DAAQSSIDFGGKK.Y
1267		707.3660	1412.7174	1412.7147	0.0027	0	59	1.2e-06	1	U			■					K.VTIGGTTAQSYTSK.D
1283		475.5941	1423.7605	1423.7671	-0.0066	1	8	0.17	1	U	■							K.VYTANITNKTATK.G
1311		720.9124	1439.8102	1439.8096	0.0006	0	45	0.00013	1				■		■			K.AQIIQQAGNSVLAK.A
1312		480.9444	1439.8114	1439.8096	0.0018	0	2	2.7	1				■		■			K.AQIIQQAGNSVLAK.A
1331		728.5278	1455.0410	1455.8045	-0.7635	0	2	0.93	1	U						■		K.AQIIQQAGNSVLSK.A
1364	1	747.9186	1493.8226	1493.8202	0.0025	0	4	1.1	1				■		■	■		K.ANQVPQQVLSLLQG.-
1365		499.2549	1494.7429	1493.7474	0.9955	1	3	2.5	1	U	■							K.QNSTGYEKVQVGGK.D
1388		504.2517	1509.7333	1509.7311	0.0022	1	23	0.0058	1	U	■							R.EDDKVYTANITNK.T
1389		755.8746	1509.7346	1509.7311	0.0036	1	26	0.0032	1	U	■							R.EDDKVYTANITNK.T
1476		793.5782	1585.1418	1584.7532	0.3886	1	3	0.54	1	U					■			K.KYEFAGGNSTNGGGVK.F
1543		548.9657	1643.8753	1643.8744	0.0009	1	33	0.00054	1	U	■							K.VQVGGKDVQLANFGGR.V
1549		824.4449	1646.8752	1646.8727	0.0025	0	83	4.5e-09	1	U			■					K.VTVGTTSVDVVLASDGK.I
1567		836.3804	1670.7462	1670.7457	0.0005	0	58	1e-05	1				■		■	■	■	R.IQDADYATEVSNMSK.A
1604		850.8765	1699.7384	1699.7359	0.0025	0	117	3.4e-12	1		■	■						R.IEDADYATEVSNMSR.A
1607		852.4095	1702.8044	1702.8025	0.0020	0	77	2.1e-08	1	U	■							K.ALAYNDAPMSVYFGGK.N
1623		858.8715	1715.7284	1715.7308	-0.0024	0	94	8.3e-10	1		■	■						R.IEDADYATEVSNMSR.A + Oxidation (M)
1628	1	860.4084	1718.8022	1718.7974	0.0049	0	56	2.2e-06	1	U	■							K.ALAYNDAPMSVYFGGK.N + Oxidation (M)
1637		867.6140	1733.2134	1731.7257	1.4877	0	3	0.49	1	U				■				R.IEDSDYATEVSNMSR.A + Oxidation (M)
1746		929.9278	1857.8410	1857.8381	0.0029	0	117	2e-12	1	U	■							K.NADVETYFGTGNVQDTK.D
1793		648.6309	1942.8709	1942.8690	0.0018	1	39	0.00023	1		■	■						R.SRIEDADYATEVSNMSR.A
1794		972.4438	1942.8730	1942.8690	0.0040	1	51	1.2e-05	1		■	■						R.SRIEDADYATEVSNMSR.A
1798		653.9626	1958.8660	1958.8640	0.0020	1	38	0.00036	1		■	■						R.SRIEDADYATEVSNMSR.A + Oxidation (M)
1813		997.5022	1992.9898	1992.9865	0.0034	0	114	1e-11	1		■	■		■				R.FDSAITNLGNTVNNLSSAR.S
1829		1043.0700	2084.1254	2084.1225	0.0029	0	122	4.4e-12	1		■	■	■	■	■	■		M.AQVINTNSLSLiTQNNiNK.N
1830		695.7158	2084.1256	2084.1225	0.0030	0	9	0.82	2		■	■	■	■	■	■		M.AQVINTNSLSLiTQNNiNK.N
1875		566.5231	2262.0633	2262.1087	-0.0455	0	4	0.39	1	U						■		R.LDSAITNLNNTTTNLSEAQSR.I
1894		793.7282	2378.1628	2378.1615	0.0013	0	60	1.1e-06	1	U	■							K.NLDVHQVQDTQGNPVPNSFAAK.T
1924		729.3831	2913.5033	2914.6349	-1.1316	1	1	0.72	1	U					■			R.YIYLLQMVKLTLMLMQAVISLLAAK.R + 3 Oxidation (M)

45 subsets and intersections (156 subset proteins in total)

10 per page 1

Not what you expected? Try **the select summary**.

Mascot: http://www.matrixscience.com/
