# Supplementary material for: MS-H: A Novel Proteomic Approach to Isolate and Type the E. coli H Antigen Using Membrane Filtration and Liquid Chromatography-Tandem Mass Spectrometry (LC-MS/MS)
Source: PLoS One. 2013 Feb 21;8(2):e57339. doi: 10.1371/journal.pone.0057339 (PMC3578835; doi:10.1371/journal.pone.0057339)
Supplement: Representative Peptide Data S1 — Peptide data are represented as the Mascot search results from all 53 serotypes, obtained under the Orbitrap platform in Table 4 with related E. coli reference strains. “U” denotes a unique peptide specific for each of the proteins 1.1, 1.2, and beyond. The number 1.1 (shown as 1 in the peptide list and phylogenetic tree) represents the protein which obtained the highest score and confidence value after a Mascot search. This protein, known as the first hit, was used to designate the MS-H type of the unknown flagellin. Related peptides 1.2 (2), 1.3 (3), etc. represented the second, third, etc. hits for MS-H typing analysis. (DOCX) [file pone.0057339.s009.docx › H17-E185.pdf]

MATRIX SCIENCE MASCOT Search Results

User :
E-mail :
Search title : Submitted from 20110728-h11-21 by Mascot Daemon on VARIABLE
MS data file : C:\Documents and Settings\keding\Desktop\Raw data\20110727-h11-21\20110728-025-EC185MS1.RAW
Database : Flagellin_v2 (192 sequences; 89,845 residues)
Taxonomy : Bacteria (Eubacteria) (192 sequences)
Timestamp : 29 Jul 2011 at 14:14:48 GMT

Not what you expected? Try **the select summary**.

▶ **Search parameters**

▶ **Score distribution**

▶ **Legend**

Protein Family Summary

Significance threshold p< 0.05 Max. number of families AUTO
Ions score or expect cut-off 0 Dendrograms cut at 0

Protein family 1 (out of 1)

10 per page 1

▼**1**

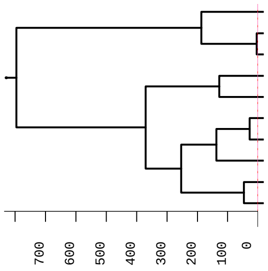

1	gi|224579297	3010	H17 0|O15:K-:H17|P12b
9	gi|33590243	652	H16 NM|O157:NM(H16)|PC02
10	gi|33590252	402	Hxx(H8 89.5%) 0|0|PC41
2	gi|30577587	1522	H17 0|O15:H17|P12b
6	gi|33590227	1092	H42 NM|O157:NM(H42)|25
3	gi|30059870	1268	H15 0|H15|E 39a
8	gi|5107847	803	H12 0|O111:H12|DEC 6a
5	gi|121144335	1126	H55 0|O75:H55|E2987-73
4	gi|30059862	1218	H10 0|H10|Bi 623-42
7	gi|6941884	986	H42 0|O157:H42|O157:H42

Threshold (**0**): 0

		Score	Mass	Matches	Sequences	emPAI
**1.1**	**gi|224579297**<br>H17 0|O15:K-:H17|P12b	3010	49533	63 (56)	27 (25)	11.36
**1.2**	**gi|30577587**<br>H17 0|O15:H17|P12b	1522	36285	36 (29)	21 (18)	5.83
**1.3**	**gi|30059870**<br>H15 0|H15|E 39a	1268	57416	36 (27)	18 (13)	1.73
**1.4**	**gi|30059862**<br>H10 0|H10|Bi 623-42<br>▶ **2 samesets of gi|30059862**	1218	44249	35 (27)	18 (13)	2.65
**1.5**	**gi|121144335**<br>H55 0|O75:H55|E2987-73	1126	62285	35 (25)	17 (12)	1.40
**1.6**	**gi|33590227**<br>H42 NM|O157:NM(H42)|25	1092	44094	25 (22)	12 (10)	1.75
**1.7**	**gi|6941884**<br>H42 0|O157:H42|O157:H42	986	44049	25 (22)	12 (10)	1.75
**1.8**	**gi|5107847**<br>H12 0|O111:H12|DEC 6a	803	57823	24 (16)	14 (9)	1.05
**1.9**	**gi|33590243**<br>H16 NM|O157:NM(H16)|PC02<br>▶ **2 samesets of gi|33590243**	652	55093	32 (16)	17 (10)	1.13
**1.10**	**gi|33590252**<br>Hxx(H8 89.5%) 0|0|PC41	402	52373	27 (13)	9 (7)	0.73

▼**152 peptide matches (94 non-duplicate, 58 duplicate)**

Query	Dupes	Observed	Mr(expt)	Mr(calc)	Delta	M	Score	Expect	Rank	U	1	2	3	4	5	6	7	8	9	10	Peptide
19	▶1	316.6906	631.3666	631.3653	0.0013	0	29	0.012	▶1		■	■	■	■	■	■	■	■	■	■	R.LSSGLR.I
71		355.1985	708.3824	708.3806	0.0018	0	18	0.095	▶1		■	■	■	■	■	■	■	■			R.FTSNIK.G
73		358.7069	715.3992	715.3977	0.0016	0	35	0.0022	▶1		■	■	■	■	■	■	■	■			K.GLTQAAR.N
87	▶1	366.6990	731.3834	731.3813	0.0021	0	39	0.00043	▶1		■								■	■	R.LSEIDR.V
102	▶1	380.2039	758.3932	758.4174	-0.0241	0	32	0.0039	▶1	U									■		K.LDEALAK.V
107	▶2	380.6959	759.3772	759.3763	0.0010	0	29	0.0072	▶1			■	■		■			■			R.LDEIDR.V
121		386.7324	771.4502	771.4490	0.0012	0	53	4.9e-06	▶1	U		■									K.ALDAAIAK.V
133		395.2189	788.4232	787.4440	0.9793	1	2	0.66	▶1	U				■							K.LVEKDGK.Y
136	▶1	395.7978	789.5810	788.4028	1.1782	0	6	0.27	▶1	U			■								K.AGDTANLK.V
212	▶1	415.2090	828.4034	828.4018	0.0017	0	24	0.0043	▶1	U	■										K.VSFSFDK.A
236	▶11	421.7592	841.5038	841.4658	0.0381	0	16	0.024	▶1	U										■	K.AVTQPQAK.D
261		425.1959	848.3772	847.4287	0.9485	0	5	0.33	▶1	U								■			K.DGDTVTIK.A
277		431.2282	860.4418	860.4240	0.0179	0	14	0.036	▶1	U										■	K.VELGGSDGK.T

Query	Dupes	Observed	Mr(expt)	Mr(calc)	Delta	M	Score	Expect	Rank	U	1	2	3	4	5	6	7	8	9	10	Peptide
335		452.2119	902.4092	901.4181	0.9911	0	13	0.051	1	U				■							K.YYVNDTK.S
369	1	466.2517	930.4888	930.4883	0.0006	0	59	5.5e-06	1			■		■	■				■		R.SSLGAVQNR
498		493.7545	985.4944	985.5556	-0.0612	0	3	0.5	1	U					■						K.AAASNVLAAAK.N
513		496.7579	991.5012	991.5008	0.0004	0	15	0.028	1	U									■		K.VLASDQTMK.I
538	1	502.2622	1002.5098	1002.5094	0.0004	1	16	0.14	1			■	■		■			■			K.SRLDEIDR.V
551	1	505.3163	1008.6180	1007.4957	1.1223	0	2	0.58	1	U									■		K.VLASDQTMK.I + Oxidation (M)
553		505.7618	1009.5090	1009.5080	0.0010	0	33	0.00052	1	U		■									K.YAVVDSATGK.Y
651	1	539.2702	1076.5258	1077.4873	-0.9614	0	13	0.076	1	U									■		K.NDGSAQAIMR.E + Oxidation (M)
701		551.2680	1100.5214	1100.5210	0.0004	0	72	5.5e-07	1		■	■	■	■	■	■	■	■	■	■	K.DDAAGQAIANR.F
732		560.7960	1119.5774	1119.5771	0.0003	0	52	5.9e-06	1	U			■								R.ISADSLQSATK.S
795		382.2095	1143.6067	1144.6564	-1.0497	1	6	2.1	1		■	■	■	■	■	■	■	■	■	■	R.LSSGLRINSAK.D
810		576.7725	1151.5304	1151.5306	-0.0002	0	12	0.058	1	U			■								K.DGSLTTDNTTK.L
893		600.8536	1199.6926	1199.6734	0.0192	1	3	0.47	1	U									■		K.LRSSLGAVQNR.F
900		603.3101	1204.6056	1204.6048	0.0009	0	47	4.1e-05	1			■				■	■				K.NQSALSTSIER.L
901		603.3258	1204.6370	1203.6347	1.0024	0	22	0.011	1	U			■								K.ESTAADVIASIK.D
918	2	612.2886	1222.5626	1222.5612	0.0015	0	46	2.5e-05	1		■				■						K.NQSSMSTAIER.L
947	1	620.2858	1238.5570	1238.5561	0.0010	0	64	3.8e-07	1		■				■						K.NQSSMSTAIER.L + Oxidation (M)
986	1	630.8437	1259.6728	1259.6721	0.0007	0	99	1.1e-10	1	U	■										K.TELVTLGSGANAK.T
1091		656.8652	1311.7158	1311.7146	0.0012	0	41	8.6e-05	1	U								■			K.AQIIQQAGNSVLA.-
1107		439.9118	1316.7136	1317.6929	-0.9793	0	1	0.81	1	U									■		K.TLGLDNFSVAPGK.V
1108		659.8546	1317.6946	1317.6929	0.0018	0	47	2.2e-05	1	U									■		K.TLGLDNFSVAPGK.V
1147	1	672.8782	1343.7418	1343.7408	0.0010	0	59	1.1e-06	1	U					■						-.SLSLITQNNINK.N
1202		694.3763	1386.7380	1386.7354	0.0026	0	100	1.3e-10	1	U		■									K.LTAADGTAIAAADVK.D
1246		475.2274	1422.6604	1423.7671	-1.1067	1	1	0.89	1	U									■		K.VYTANITNKTATK.G
1269	2	720.9133	1439.8120	1439.8096	0.0024	0	105	1.5e-10	1				■	■	■	■	■				K.AQIIQQAGNSVLAK.A
1286		728.2500	1454.4854	1454.6889	-0.2034	0	2	0.95	1	U								■			K.AAADGDTSATITYK.S
1291		728.9096	1455.8046	1455.8045	0.0001	0	108	2.5e-11	1	U		■									K.AQIIQQAGNSVLSK.A
1326	1	747.9178	1493.8210	1493.8202	0.0009	0	84	2.5e-08	1			■	■	■		■	■				K.ANQVPQQVLSLLQG.-
1326		747.9178	1493.8210	1493.7474	0.0736	1	5	1.9	5	U									■		K.QNSTGYEKVQVGGK.D
1395		514.2353	1539.6841	1539.6835	0.0006	1	66	2.6e-07	1	U	■										K.SSDTANMKDGTTNAK.V
1396		770.8497	1539.6848	1539.6835	0.0014	1	48	1.7e-05	1	U	■										K.SSDTANMKDGTTNAK.V
1404		774.5757	1547.1368	1546.7991	0.3377	0	2	1.1	1	U										■	K.GEGGIVADAAGQLYVK.V
1421		522.2789	1563.8149	1563.8145	0.0004	0	21	0.0072	1	U	■										K.IDSATLGLTGFDVQK.K
1423		782.9158	1563.8170	1563.8145	0.0026	0	124	3.6e-13	1	U	■										K.IDSATLGLTGFDVQK.K
1441		789.4190	1576.8234	1576.8209	0.0025	0	88	2.2e-09	1	U		■									R.VSGQTQFNGVNVLSK.N
1459	2	796.4268	1590.8390	1590.8366	0.0025	0	74	3.7e-08	1		■			■							R.VSSQTQFNGVNVLAK.D
1481		538.9451	1613.8135	1613.8121	0.0014	1	26	0.02	1		■	■	■	■	■	■	■	■	■	■	R.INSAKDDAAGQAIANR.F
1482		807.9141	1613.8136	1613.8121	0.0015	1	71	6.5e-07	1		■	■	■	■	■	■	■	■	■	■	R.INSAKDDAAGQAIANR.F
1519		823.5882	1645.1618	1643.8744	1.2875	1	1	0.81	1	U									■		K.VQVGGKDVQLANFGGR.V
1542	1	836.3810	1670.7474	1670.7457	0.0017	0	112	3.4e-11	1		■	■	■	■	■	■	■	■			R.IQDADYATEVSNMSK.A
1560	1	844.3789	1686.7432	1686.7407	0.0026	0	107	1.6e-10	1		■	■	■	■	■	■	■	■			R.IQDADYATEVSNMSK.A + Oxidation (M)
1565		564.2756	1689.8050	1689.8686	-0.0636	1	1	0.72	1	U				■							K.DGATINKQVAVGAGDFK.D
1569		564.9774	1691.9104	1691.9094	0.0010	1	35	0.00031	1	U	■										K.IDSATLGLTGFDVQKK.S
1570		846.9631	1691.9116	1691.9094	0.0022	1	54	3.8e-06	1	U	■										K.IDSATLGLTGFDVQKK.S
1604	1	860.3589	1718.7032	1718.7974	-0.0941	0	2	0.67	1	U									■		K.ALAYNDAPMSVYFGGK.N + Oxidation (M)
1627		871.4350	1740.8554	1740.8530	0.0025	0	77	2e-08	1	U		■									K.QVNLLSYTDTASNSTK.Y
1706	1	911.9289	1821.8432	1821.8421	0.0011	0	142	6.4e-15	1	U	■										K.IYATDYDGTNATAFTAK.T
1707		608.2888	1821.8446	1821.8421	0.0025	0	26	0.0027	1	U	■										K.IYATDYDGTNATAFTAK.T
1792	2	979.4792	1956.9438	1956.9429	0.0010	0	115	2.9e-12	1	U	■										K.ATNSYFAIVADGSADNTLK.N
1793		653.3228	1956.9466	1956.9429	0.0037	0	65	3.4e-07	1	U	■										K.ATNSYFAIVADGSADNTLK.N
1858	3	1043.0700	2084.1254	2084.1225	0.0029	0	120	6.4e-12	1				■	■			■		■	■	M.AQVINTNSLSLiTQNNiNK.N
1859		695.7167	2084.1283	2084.1225	0.0057	0	77	1.3e-07	1				■	■			■		■	■	M.AQVINTNSLSLiTQNNiNK.N
1859		695.7167	2084.1283	2085.1066	-0.9783	0	75	2.1e-07	4	U						■					M.AQVINTNSLSLITQNNIDK.N
1859		695.7167	2084.1283	2085.0814	-0.9531	0	66	1.7e-06	5	U		■									M.AQVINTNSLSLNTQNNINK.N
1860	3	1043.5500	2085.0854	2085.0814	0.0040	0	96	1.6e-09	1	U		■									M.AQVINTNSLSLNTQNNINK.N
1862	3	1043.5630	2085.1114	2085.1066	0.0049	0	103	3.1e-10	1	U						■					M.AQVINTNSLSLITQNNIDK.N
1901	2	1092.5070	2182.9994	2182.9978	0.0016	0	130	1.6e-13	1	U	■										K.LTSTDSAGNATDAGYGLQAADGK.I
1902		728.6747	2183.0023	2182.9978	0.0044	0	107	3.6e-11	1	U	■										K.LTSTDSAGNATDAGYGLQAADGK.I
1938		750.3730	2248.0972	2248.0931	0.0041	0	66	1.6e-06	1			■	■		■	■		■			R.LDSAVTNLNNTTTNLSEAQSR.I
1939		1125.0560	2248.0974	2248.0931	0.0043	0	124	2.3e-12	1			■	■		■	■		■			R.LDSAVTNLNNTTTNLSEAQSR.I
1950		763.3605	2287.0597	2286.0434	1.0163	1	4	0.42	1	U					■						K.DMTITSAGGNAQVATDKAYNDK.Y + Oxidation (M
1957		767.0641	2298.1705	2297.1751	0.9954	1	1	0.71	2	U				■							K.KIDSSTLNLSSFDATNLGTSVK.D
1961		769.0776	2304.2110	2304.2074	0.0036	1	49	1.1e-05	1	U	■										R.LSEIDRVSSQTQFNGVNVLAK.D
1962		770.4013	2308.1821	2308.1798	0.0023	0	66	2.6e-07	1	U	■										K.AAVEITTTTTPYTEGNNTIALK.S
1965	3	1155.0990	2308.1834	2308.1798	0.0036	0	80	1e-08	1	U	■										K.AAVEITTTTTPYTEGNNTIALK.S
1977	1	1166.0520	2330.0894	2330.0874	0.0020	0	130	1.1e-13	1	U	■										K.TATYTDASGNTQTSAITLGGTDGK.T
1978		777.7043	2330.0911	2330.0874	0.0037	0	44	4.2e-05	1	U	■										K.TATYTDASGNTQTSAITLGGTDGK.T
2007	1	1247.6120	2493.2094	2493.2082	0.0012	0	131	7.9e-14	1	U	■										R.ELTVQASTGTNSASDLSSIQDEIK.S
2008		832.0776	2493.2110	2493.2082	0.0028	0	83	4.9e-09	1	U	■										R.ELTVQASTGTNSASDLSSIQDEIK.S
2025		1307.1500	2612.2854	2612.2790	0.0065	0	107	2.1e-11	1	U		■									R.NANDGISLAQTAEGALSEINNNLQR.I
2027		1315.1450	2628.2754	2628.2739	0.0015	0	131	3.6e-13	1		■		■	■	■			■	■		R.NANDGISVAQTTEGALSEINNNLQR
2028		877.1002	2628.2788	2628.2739	0.0049	0	75	1.5e-07	1		■		■	■	■			■	■		R.NANDGISVAQTTEGALSEINNNLQR
2045		917.1347	2748.3823	2748.3777	0.0045	1	39	0.00013	1	U	■										R.VRELTVQASTGTNSASDLSSIQDEIK.S
2063		1049.5270	3145.5592	3144.5759	0.9833	1	21	0.0081	2	U				■							R.SSLGAVQNRLDSAIANLNNTTTNLSEAQSR.I
2063		1049.5270	3145.5592	3144.5395	1.0196	0	18	0.016	3	U							■				R.SSLGAVQNRPDSAVTNLNNTTTNLSEAQSR.I
2064		1573.7890	3145.5634	3145.5599	0.0035	0	100	1.1e-10	1	U	■										R.SSLGAVQNQLNSAITNLNNTTTNLSEAQSR.I
2064		1573.7890	3145.5634	3144.5395	1.0239	0	68	1.5e-07	2	U							■				R.SSLGAVQNRPDSAVTNLNNTTTNLSEAQSR.I
2064		1573.7890	3145.5634	3144.5759	0.9876	1	53	4.6e-06	3	U				■							R.SSLGAVQNRLDSAIANLNNTTTNLSEAQSR.I
2065	1	1049.8570	3146.5492	3145.5599	0.9893	0	60	9.5e-07	1	U	■										R.SSLGAVQNQLNSAITNLNNTTTNLSEAQSR.I
2082		1166.2910	3495.8512	3495.8421	0.0091	0	19	0.013	1	U	■										K.NQVAGTPVTTSTPVAVTAAANTALSNAGVTGSVELVK.
2085		1191.5920	3571.7542	3571.7490	0.0052	1	106	2.3e-11	1	U	■										K.TATYTDASGNTQTSAITLGGTDGKTELVTLGSGANAK.

▶ 49 subsets and intersections (157 subset proteins in total)

10 per page 1

Not what you expected? Try **the select summary**.

Mascot: http://www.matrixscience.com/
