# Supplementary material for: MS-H: A Novel Proteomic Approach to Isolate and Type the E. coli H Antigen Using Membrane Filtration and Liquid Chromatography-Tandem Mass Spectrometry (LC-MS/MS)
Source: PLoS One. 2013 Feb 21;8(2):e57339. doi: 10.1371/journal.pone.0057339 (PMC3578835; doi:10.1371/journal.pone.0057339)
Supplement: Representative Peptide Data S1 — Peptide data are represented as the Mascot search results from all 53 serotypes, obtained under the Orbitrap platform in Table 4 with related E. coli reference strains. “U” denotes a unique peptide specific for each of the proteins 1.1, 1.2, and beyond. The number 1.1 (shown as 1 in the peptide list and phylogenetic tree) represents the protein which obtained the highest score and confidence value after a Mascot search. This protein, known as the first hit, was used to designate the MS-H type of the unknown flagellin. Related peptides 1.2 (2), 1.3 (3), etc. represented the second, third, etc. hits for MS-H typing analysis. (DOCX) [file pone.0057339.s009.docx › H18-E186.pdf]

{MATRIX SCIENCE} MASCOT Search Results

User :
E-mail :
Search title : Submitted from 20110728-h11-21 by Mascot Daemon on VARIABLE
MS data file : C:\Documents and Settings\keding\Desktop\Raw data\20110727-h11-21\20110728-026-EC186MS1.RAW
Database : Flagellin_v2 (192 sequences; 89,845 residues)
Taxonomy : Bacteria (Eubacteria) (192 sequences)
Timestamp : 29 Jul 2011 at 14:16:43 GMT

Not what you expected? Try **the select summary**.

▸ **Search parameters**
▸ **Score distribution**
▸ **Legend**

Protein Family Summary

Significance threshold p< 0.05 Max. number of families AUTO
Ions score or expect cut-off 0 Dendrograms cut at 0

Protein families 1–2 (out of 2)

10 per page 1

▾**1**

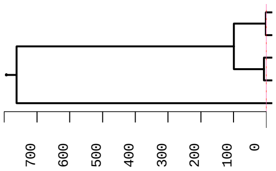

1	gi|30059874	1436	H18 0|H18|K 12a [gi|293405409|ref|ZP_06649401| Hxx 0|0|FVEC1412] [gi|300899178|re...
4	gi|40994711	701	H4 0|O154:H4|0
3	gi|6009849	784	H49 0|H49|U11a-44
5	gi|33590243	321	H16 NM|O157:NM(H16)|PC02
2	gi|224579297	1221	H17 0|O15:K-:H17|P12b

Threshold (**0**): 0

		Score	Mass	Matches	Sequences	emPAI
1.1	gi|30059874	1436	57210	38 (27)	24 (21)	3.53
	H18 0|H18|K 12a [gi|293405409|ref|ZP_06649401| Hxx 0|0|FVEC1412] [gi|300899178|ref|ZP_07117455| Hxx 0|0|MS 198-1] [gi|218705418|ref|YP_002412937| Hxx 0|0|UMN026] [gi|218432...					
1.2	gi|224579297	1221	49533	31 (23)	20 (18)	3.13
	H17 0|O15:K-:H17|P12b					
1.3	gi|6009849	784	58493	29 (16)	16 (13)	1.40
	H49 0|H49|U11a-44					
1.4	gi|40994711	701	36224	26 (14)	15 (11)	2.41
	H4 0|O154:H4|0					
	▸ 1 sameset of gi|40994711					
1.5	gi|33590243	321	55093	19 (6)	12 (5)	0.42
	H16 NM|O157:NM(H16)|PC02					
	▸ 2 samesets of gi|33590243					

▾**74 peptide matches (59 non-duplicate, 15 duplicate)**

Query	Dupes	Observed	Mr(expt)	Mr(calc)	Delta	M	Score	Expect	Rank	U	1	2	3	4	5	Peptide
5		305.1667	608.3188	609.2428	-0.9240	0	6	0.28	▸1	U				■		K.NDSMK.I + Oxidation (M)
30	▸3	316.6909	631.3672	631.3653	0.0019	0	24	0.04	▸1		■	■	■	■	■	R.LSSGLR.I
56		331.6815	661.3484	661.3469	0.0016	0	23	0.0055	▸1	U	■					K.DMVGLK.L
74		339.6788	677.3430	677.3418	0.0012	0	25	0.0029	▸1	U	■					K.DMVGLK.L + Oxidation (M)
97		355.1986	708.3826	708.3806	0.0020	0	8	1	▸1		■	■	■	■		R.FTSNIK.G
101	▸1	358.7072	715.3998	715.3977	0.0022	0	29	0.0099	▸1		■	■	■	■		K.GLTQAAR.N
116	▸1	367.1967	732.3788	731.3813	0.9975	0	4	1.3	▸1			■			■	R.LSEIDR.V
131		380.2042	758.3938	758.4174	-0.0235	0	28	0.01	▸1	U					■	K.LDEALAK.V
136	▸1	380.6960	759.3774	759.3763	0.0012	0	37	0.0011	▸1		■		■	■		R.LDEIDR.V
148	▸1	386.7325	771.4504	771.4490	0.0014	0	14	0.036	▸1	U				■		K.ALDAAIAK.V
233		415.2088	828.4030	828.4018	0.0013	0	14	0.04	▸1	U		■				K.VSFSFDK.A
273	▸2	423.7197	845.4248	844.4402	0.9846	0	15	0.035	▸1	U			■			K.AAAGAESIR.Y
411		466.7436	931.4726	930.4883	0.9844	0	5	1.5	▸1					■	■	R.SSLGAVQNR
433		470.7403	939.4660	939.4662	-0.0001	0	35	0.00035	▸1	U	■					K.GTFTANTTK.F
444	▸1	473.2596	944.5046	944.5039	0.0007	0	46	6.8e-05	▸1		■		■			R.SSLGAIQNR.L
580	▸2	502.2624	1002.5102	1002.5094	0.0008	1	16	0.14	▸1		■		■	■		K.SRLDEIDR.V
589		503.3683	1004.7220	1005.4727	-0.7507	0	2	0.58	▸1	U	■					K.TNAGTDTQAK.L
607	▸1	508.2628	1014.5110	1014.5709	-0.0599	0	2	0.66	▸1	U					■	K.ALATTNPLSK.L
689	▸1	539.2699	1076.5252	1077.4873	-0.9620	0	12	0.092	▸1	U					■	K.NDGSAQAIMR.E + Oxidation (M)
751		551.2681	1100.5216	1100.5210	0.0006	0	70	9.4e-07	▸1		■	■	■	■	■	K.DDAAGQAIANR.F
909		596.3022	1190.5898	1190.5891	0.0008	0	62	3.4e-06	▸1		■		■			K.NQSALSSSIER.L
914		598.8019	1195.5892	1194.5517	1.0376	0	3	0.54	▸1	U			■			K.DAAQSSIDFGGK.K
922		600.8537	1199.6928	1199.6734	0.0194	1	3	0.46	▸1	U					■	K.LRSSLGAVQNR.F
955		612.2885	1222.5624	1222.5612	0.0013	0	42	5.9e-05	▸1	U		■				K.NQSSMSTAIER.L
980		620.2864	1238.5582	1238.5561	0.0022	0	12	0.065	▸1	U		■				K.NQSSMSTAIER.L + Oxidation (M)
1021		630.8431	1259.6716	1259.6721	-0.0005	0	86	2.4e-09	▸1	U		■				K.TELVTLGSGANAK.T

Query	Dupes	Observed	Mr(expt)	Mr(calc)	Delta	M	Score	Expect	Rank	U	1	2	3	4	5	Peptide
1023		631.3148	1260.6150	1260.6132	0.0019	0	49	1.2e-05	▶1	U	■					K.LSDLMANNANAK.T
1050		639.3121	1276.6096	1276.6081	0.0015	0	83	5.1e-09	▶1	U	■					K.LSDLMANNANAK.T + Oxidation (M)
1127		659.3425	1316.6704	1316.6685	0.0020	0	70	1.1e-07	▶1	U	■					K.LDNTGVTTAGVNR.Y
1278		720.9120	1439.8094	1439.8096	-0.0002	0	84	1.8e-08	▶1		■		■	■		K.AQIIQQAGNSVLAK.A
1335		747.9177	1493.8208	1493.8202	0.0007	0	26	0.017	▶1		■		■	■		K.ANQVPQQVLSLLQG.-
1443		789.4184	1576.8222	1576.8209	0.0013	0	94	4.9e-10	▶1		■			■		R.VSGQTQFNGVNVLSK
1461		796.4266	1590.8386	1590.8366	0.0021	0	58	1.5e-06	▶1	U		■				R.VSSQTQFNGVNVLAK.D
1545		836.3809	1670.7472	1670.7457	0.0015	0	110	6.8e-11	▶1		■	■	■	■		R.IQDADYATEVSNMSK.A
1567		844.3782	1686.7418	1686.7407	0.0012	0	96	1.9e-09	▶1		■	■	■	■		R.IQDADYATEVSNMSK.A + Oxidation (M)
1578		564.9775	1691.9107	1691.9094	0.0013	1	17	0.02	▶1	U		■				K.KIDSATLGLTGFDVQK.K
1609		860.3575	1718.7004	1718.7974	-0.0969	0	1	0.76	▶1	U					■	K.ALAYNDAPMSVYFGGK.N + Oxidation (M)
1672		887.9641	1773.9136	1773.9109	0.0028	0	131	2.2e-13	▶1	U	■					K.AVASSTDILNAVAGVDGSK.V
1720		911.9299	1821.8452	1821.8421	0.0031	0	147	2.1e-15	▶1	U		■				K.IYATDYDGTNATAFTAK.T
1749		931.3964	1860.7782	1860.9152	-0.1370	1	2	0.7	▶1	U					■	K.AVEWAVKNDGSAQAIMR.E + Oxidation (M)
1814	▶1	979.4799	1956.9452	1956.9429	0.0024	0	127	1.8e-13	▶1	U		■				K.ATNSYFAIVADGSADNTLK.N
1815		653.3226	1956.9460	1956.9429	0.0031	0	77	1.8e-08	▶1	U		■				K.ATNSYFAIVADGSADNTLK.N
1873		695.7155	2084.1247	2084.1225	0.0021	0	72	4.1e-07	▶1		■		■	■	■	M.AQVINTNSLSLiTQNNiNK.N
1874		1043.0700	2084.1254	2084.1225	0.0029	0	120	5.8e-12	▶1		■		■	■	■	M.AQVINTNSLSLiTQNNiNK.N
1917		1092.5070	2182.9994	2182.9978	0.0016	0	138	2.7e-14	▶1	U		■				K.LTSTDSAGNATDAGYGLQAADGK.I
1952		1125.0540	2248.0934	2248.0931	0.0003	0	124	2.4e-12	▶1		■		■	■		R.LDSAVTNLNNTTTNLSEAQSR.I
1953		750.3727	2248.0963	2248.0931	0.0032	0	55	2e-05	▶1		■		■	■		R.LDSAVTNLNNTTTNLSEAQSR.I
1976		1155.0980	2308.1814	2308.1798	0.0016	0	62	7e-07	▶1	U		■				K.AAVEITTTTTPYTEGNNTIALK.S
1977		770.4022	2308.1848	2308.1798	0.0050	0	17	0.022	▶1	U		■				K.AAVEITTTTTPYTEGNNTIALK.S
1983		1165.5440	2329.0734	2329.0710	0.0025	0	112	6e-12	▶1	U	■					K.TPAEYFAQADGTITSGENAATSK.A
1984		777.3652	2329.0738	2329.0710	0.0028	0	54	3.9e-06	▶1	U	■					K.TPAEYFAQADGTITSGENAATSK.A
1985		777.7032	2330.0878	2330.0874	0.0004	0	43	4.6e-05	▶1	U		■				K.TATYTDASGNTQTSAITLGGTDGK.T
1986		1166.0530	2330.0914	2330.0874	0.0040	0	108	1.8e-11	▶1	U		■				K.TATYTDASGNTQTSAITLGGTDGK.T
2007		823.0651	2466.1735	2466.1703	0.0031	0	79	1.3e-08	▶1	U	■					K.VSTEADVGFGAAAPGTPVEYTYHK.D
2011		832.0778	2493.2116	2493.2082	0.0034	0	34	0.00044	▶1	U		■				R.ELTVQASTGTNSASDLSSIQDEIK.S
2028		1315.1450	2628.2754	2628.2739	0.0015	0	111	3.7e-11	▶1		■	■	■		■	R.NANDGISVAQTTEGALSEINNNLQR
2042		955.4830	2863.4272	2863.4199	0.0073	1	63	5.4e-07	▶1	U	■					K.KIDSDTLNLAGFNVNGEGETANTAATLK.D
2062		1166.2890	3495.8452	3495.8421	0.0031	0	21	0.0081	▶1	U		■				K.NQVAGTPVTTSTPVAVTAAANTALSNAGVTGSVELVK.L
2064		1191.5920	3571.7542	3571.7490	0.0052	1	80	1.1e-08	▶1	U		■				K.TATYTDASGNTQTSAITLGGTDGKTELVTLGSGANAK.T

▶**47 subsets and intersections (163 subset proteins in total)**

▶**2** **gi|112820172** 19 H21 0|EHEC serogroup: O113:H21|0

10 per page 1

Not what you expected? Try **the select summary**.

Mascot: http://www.matrixscience.com/
