# Supplementary material for: MS-H: A Novel Proteomic Approach to Isolate and Type the E. coli H Antigen Using Membrane Filtration and Liquid Chromatography-Tandem Mass Spectrometry (LC-MS/MS)
Source: PLoS One. 2013 Feb 21;8(2):e57339. doi: 10.1371/journal.pone.0057339 (PMC3578835; doi:10.1371/journal.pone.0057339)
Supplement: Representative Peptide Data S1 — Peptide data are represented as the Mascot search results from all 53 serotypes, obtained under the Orbitrap platform in Table 4 with related E. coli reference strains. “U” denotes a unique peptide specific for each of the proteins 1.1, 1.2, and beyond. The number 1.1 (shown as 1 in the peptide list and phylogenetic tree) represents the protein which obtained the highest score and confidence value after a Mascot search. This protein, known as the first hit, was used to designate the MS-H type of the unknown flagellin. Related peptides 1.2 (2), 1.3 (3), etc. represented the second, third, etc. hits for MS-H typing analysis. (DOCX) [file pone.0057339.s009.docx › H19-09-0523.pdf]

MATRIX SCIENCE MASCOT Search Results

User :
E-mail :
Search title : Submitted from 20110728-h11-21 by Mascot Daemon on VARIABLE
MS data file : C:\Documents and Settings\keding\Desktop\Raw data\20110727-h11-21\20110728-027-090523MS1.RAW
Database : Flagellin_v2 (192 sequences; 89,845 residues)
Taxonomy : Bacteria (Eubacteria) (192 sequences)
Timestamp : 29 Jul 2011 at 14:18:21 GMT

Not what you expected? Try **the select summary**.

Search parameters
Score distribution
Legend

Protein Family Summary

Significance threshold p< 0.05 Max. number of families AUTO
Ions score or expect cut-off 0 Dendrograms cut at 0

Protein family 1 (out of 1)

10 per page 1

1

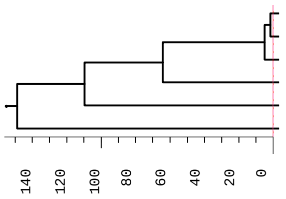

1	**gi|30059876**	3037	H19 0|H19|A 18d
6	**gi|46093552**	251	H53 0|0|E480-68
5	**gi|33590243**	349	H16 NM|O157:NM(H16)|PC02
2	**gi|30059874**	1155	H18 0|H18|K 12a [gi|293405409|ref|ZP_06649401| Hxx 0|0|FVEC1412] [gi|3008991...
3	**gi|121144335**	876	H55 0|O75:H55|E2987-73
4	**gi|224579297**	591	H17 0|O15:K-:H17|P12b

Threshold (**0**): 0

		Score	Mass	Matches	Sequences	emPAI
**1.1**	**gi|30059876**<br>H19 0|H19|A 18d<br>**1 sameset of gi|30059876**	3037	62485	82 (66)	37 (34)	12.66
**1.2**	**gi|30059874**<br>H18 0|H18|K 12a [gi|293405409|ref|ZP_06649401| Hxx 0|0|FVEC1412] [gi|300899178|ref|ZP_07117455| Hxx 0|0|MS 198-1] [gi|218705418|ref|YP_002412937| Hxx 0|0|UMN026] [gi|21...	1155	57210	42 (30)	21 (16)	2.83
**1.3**	**gi|121144335**<br>H55 0|O75:H55|E2987-73	876	62285	33 (21)	18 (13)	1.66
**1.4**	**gi|224579297**<br>H17 0|O15:K-:H17|P12b	591	49533	22 (14)	12 (8)	1.31
**1.5**	**gi|33590243**<br>H16 NM|O157:NM(H16)|PC02<br>**2 samesets of gi|33590243**	349	55093	21 (10)	14 (7)	0.79
**1.6**	**gi|46093552**<br>H53 0|0|E480-68	251	44861	20 (7)	15 (5)	0.64

112 peptide matches (84 non-duplicate, 28 duplicate)

Query	Dupes	Observed	Mr(expt)	Mr(calc)	Delta	M	Score	Expect	Rank	U	1	2	3	4	5	6	Peptide
19	3	**316.6907**	**631.3668**	**631.3653**	**0.0015**	**0**	**31**	**0.0069**	1		■	■	■	■	■	■	**R.LSSGLR.I**
23		317.6880	633.3614	632.3129	1.0485	0	5	0.33	1	U						■	R.ANVDSK.T
70		355.1985	708.3824	708.3806	0.0018	0	17	0.12	1		■	■	■	■		■	R.FTSNIK.G
72		**358.7070**	**715.3994**	**715.3977**	**0.0018**	**0**	**30**	**0.0068**	1		■	■	■	■			**K.GLTQAAR.N**
73		358.7140	715.4134	715.4116	0.0019	0	8	0.86	1	U						■	K.IDIDLK.K
86	1	366.6990	731.3834	731.3813	0.0021	0	2	2	1					■	■		R.LSEIDR.V
101		**380.2043**	**758.3940**	**758.4174**	**-0.0233**	**0**	**25**	**0.019**	1	**U**					■		**K.LDEALAK.V**
105	2	**380.6961**	**759.3776**	**759.3763**	**0.0014**	**0**	**29**	**0.007**	1		■	■	■				**R.LDEIDR.V**
252	1	**426.2298**	**850.4450**	**850.4436**	**0.0014**	**0**	**54**	**3.6e-06**	1	**U**	■						**K.ALYLDEK.G**
315		**445.7330**	**889.4514**	**889.4505**	**0.0009**	**0**	**24**	**0.016**	1	**U**	■						**K.SQEVNVSK.D**
370		**466.2518**	**930.4890**	**930.4883**	**0.0008**	**0**	**64**	**1.7e-06**	1		■		■		■		**R.SSLGAVQNR**
373		466.7436	931.4726	930.4559	1.0167	0	3	2.4	1	U						■	K.DGAYHAAVK.N
388		**470.7405**	**939.4664**	**939.4662**	**0.0003**	**0**	**26**	**0.0024**	1	**U**		■					**K.GTFTANTTK.F**
404		**473.2488**	**944.4830**	**944.4815**	**0.0016**	**0**	**62**	**1.8e-06**	1	**U**	■						**K.AETTADPLK.A**
405		473.2614	944.5082	944.5039	0.0043	0	4	1.3	1			■				■	R.SSLGAIQNR
483		490.7309	979.4472	979.4458	0.0014	0	13	0.072	1	U	■						K.DGTITSSDGK.A
534		**502.2620**	**1002.5094**	**1002.5094**	**0.0000**	**1**	**39**	**0.00082**	1		■	■	■				**K.SRLDEIDR.V**
535		335.1779	1002.5119	1002.5094	0.0025	1	15	0.17	1		■	■	■				K.SRLDEIDR.V
544		503.3684	1004.7222	1005.4727	-0.7505	0	0	0.98	1	U		■					K.TNAGTDTQAK.L
557	1	**506.7511**	**1011.4876**	**1011.4873**	**0.0004**	**0**	**39**	**0.00014**	1	**U**	■						**K.AADGTVTYSK.N**
653		539.2700	1076.5254	1077.4873	-0.9618	0	6	0.34	1	U					■		K.NDGSAQAIMR.E + Oxidation (M)

Query	Dupes	Observed	Mr(expt)	Mr(calc)	Delta	M	Score	Expect	Rank	U	1	2	3	4	5	6	Peptide
694		551.2678	1100.5210	1100.5210	0.0000	0	53	4.2e-05	▶1		■	■	■	■	■	■	K.DDAAGQAIANR.F
790		572.8200	1143.6254	1144.6564	-1.0309	1	3	4.9	▶1		■	■	■	■	■	■	R.LSSGLRINSAK.D
828		581.3037	1160.5928	1160.5925	0.0004	0	88	1.9e-09	▶1	U	■						K.ALDEAISSIDK.F
836	▶1	582.7971	1163.5796	1163.5782	0.0014	0	66	8.5e-07	▶1	U	■						K.GNLTQTGSGTTK.A
872	▶2	596.3027	1190.5908	1190.5891	0.0018	0	55	1.7e-05	▶1		■	■					K.NQSALSSSIER.L
892		600.8531	1199.6916	1199.6734	0.0182	1	3	0.46	▶1	U					■		K.LRSSLGAVQNR.F
938		620.2844	1238.5542	1238.5561	-0.0018	0	9	0.12	▶1				■	■			K.NQSSMSTAIER.L + Oxidation (M)
976		630.8258	1259.6370	1259.6721	-0.0351	0	15	0.033	▶1	U						■	K.TVANGGDIVLSSK.T
977		630.8429	1259.6712	1259.6721	-0.0009	0	71	7.3e-08	▶1	U				■			K.TELVTLGSGANAK.T
1096		659.3426	1316.6706	1316.6685	0.0022	0	64	3.9e-07	▶1	U		■					K.LDNTGVTTAGVNR.Y
1133		672.8777	1343.7408	1343.7408	0.0000	0	76	2.7e-08	▶1	U			■				-.SLSLITQNNINK.N
1154		683.3239	1364.6332	1364.6783	-0.0451	0	3	0.45	▶1	U			■				K.GSVSNTAATTDTLK.L
1198		706.5145	1411.0144	1409.6787	1.3358	0	0	0.93	▶1	U						■	K.AEIQIDSHSADPK.A
1236	▶2	718.3207	1434.6268	1434.6263	0.0006	0	106	2.5e-11	▶1	U	■						K.DSNSYSFSATAASK.D
1238		480.9441	1439.8105	1439.8096	0.0009	0	50	4.7e-05	▶1		■	■	■				K.AQIIQQAGNSVLAK.A
1239	▶2	720.9135	1439.8124	1439.8096	0.0028	0	110	5e-11	▶1		■	■	■				K.AQIIQQAGNSVLAK.A
1247	▶1	724.3826	1446.7506	1446.7501	0.0006	0	97	5e-10	▶1	U	■						K.GMTITSAGGNAQVLK.D
1269		732.3803	1462.7460	1462.7450	0.0011	0	84	7.3e-09	▶1	U	■						K.GMTITSAGGNAQVLK.D + Oxidation (M)
1271		732.8884	1463.7622	1463.7620	0.0003	1	78	2.3e-08	▶1	U	■						K.ALDEAISSIDKFR.S
1272	▶1	488.9282	1463.7628	1463.7620	0.0008	1	48	2.3e-05	▶1	U	■						K.ALDEAISSIDKFR.S
1310		498.5832	1492.7278	1493.7474	-1.0196	1	5	1.8	▶1	U					■		K.QNSTGYEKVQVGGK.D
1311		747.9178	1493.8210	1493.8202	0.0009	0	34	0.0024	▶1		■	■					K.ANQVPQQVLSLLQG.-
1384		780.9052	1559.7958	1560.8260	-1.0302	0	4	2.1	▶1	U			■				R.VSGQTQFNGVNVLAK.D
1405		526.6147	1576.8223	1576.8209	0.0013	0	33	0.00066	▶1		■	■					R.VSGQTQFNGVNVLSK.D
1406	▶2	789.4185	1576.8224	1576.8209	0.0015	0	94	4.5e-10	▶1		■	■					R.VSGQTQFNGVNVLSK.D
1430		534.7189	1601.1349	1601.8988	-0.7640	1	9	0.14	▶1	U						■	K.TVANGGDIVLSSKTIK.A
1436	▶1	803.8822	1605.7498	1605.7482	0.0016	0	112	5.9e-12	▶1	U	■						K.AANIDDSGAITDQTSK.V
1441		538.9449	1613.8129	1613.8121	0.0008	1	46	0.00021	▶1		■	■	■	■	■	■	R.INSAKDDAAGQAIANR.F
1442		807.9142	1613.8138	1613.8121	0.0017	1	68	1.4e-06	▶1		■	■	■	■	■	■	R.INSAKDDAAGQAIANR.F
1486		549.6334	1645.8784	1645.8787	-0.0004	0	48	1.8e-05	▶1	U	■						K.NVQFAAATASNVLAAAK.D
1487		823.9477	1645.8808	1645.8787	0.0021	0	120	1.1e-12	▶1	U	■						K.NVQFAAATASNVLAAAK.D
1502		836.3804	1670.7462	1670.7457	0.0005	0	132	4.2e-13	▶1		■	■	■	■			R.IQDADYATEVSNMSK.A
1503		557.9231	1670.7475	1670.7457	0.0017	0	47	0.00011	▶1		■	■	■	■			R.IQDADYATEVSNMSK.A
1514		559.9532	1676.8378	1676.8370	0.0008	0	56	2.6e-06	▶1	U	■						K.IDSDTLNLAGFNVNGK.G
1515	▶2	839.4265	1676.8384	1676.8370	0.0015	0	64	4.4e-07	▶1	U	■						K.IDSDTLNLAGFNVNGK.G
1523		844.3787	1686.7428	1686.7407	0.0022	0	109	8.5e-11	▶1		■	■	■	■			R.IQDADYATEVSNMSK.A + Oxidation (M)
1548		569.9570	1706.8492	1706.8475	0.0017	0	57	1.9e-06	▶1	U	■						K.DAYGNSAAAAVGTVIEAK.G
1549	▶2	854.4319	1706.8492	1706.8475	0.0017	0	98	1.7e-10	▶1	U	■						K.DAYGNSAAAAVGTVIEAK.G
1555		855.3944	1708.7742	1708.7726	0.0016	0	105	3.4e-11	▶1	U	■						K.AATWDNLMANTDTTGK.D
1559		571.2645	1710.7717	1711.8781	-1.1064	1	2	0.6	▶1	U						■	K.AADGLYALKDGTGYAVK.D
1565		572.6022	1714.7848	1715.7308	-0.9460	0	5	0.68	▶1	U						■	R.IEDSDYATEVSNMSR.A
1570		860.3580	1718.7014	1718.7974	-0.0959	0	4	0.42	▶1	U					■		K.ALAYNDAPMSVYFGGK.N + Oxidation (M)
1578		863.3915	1724.7684	1724.7676	0.0009	0	110	9.3e-12	▶1	U	■						K.AATWDNLMANTDTTGK.D + Oxidation (M)
1658	▶1	602.6514	1804.9324	1804.9319	0.0005	1	57	9.6e-06	▶1	U	■						K.KIDSDTLNLAGFNVNGK.G
1659	▶1	903.4738	1804.9330	1804.9319	0.0011	1	115	1.5e-11	▶1	U	■						K.KIDSDTLNLAGFNVNGK.G
1660		602.9793	1805.9161	1806.9476	-1.0315	1	15	0.038	▶2	U			■				K.KIDSSTLNLTGFNVNGK.G
1670		911.4660	1820.9174	1820.9156	0.0018	0	75	3.2e-08	▶1	U	■						K.DSLLSTLAPNAGDTFTAK.V
1671		607.9799	1820.9179	1820.9156	0.0023	0	13	0.053	▶1	U	■						K.DSLLSTLAPNAGDTFTAK.V
1686		615.2999	1842.8779	1841.8908	0.9871	1	11	0.073	▶1	U					■		K.VNATDGSVGGAKAFGSNYK.N
1692		617.9700	1850.8882	1850.8858	0.0024	1	55	3.1e-06	▶1	U	■						K.SQEVNVSKDGTITSSDGK.A
1745		653.3220	1956.9442	1956.9429	0.0013	0	23	0.0047	▶1	U				■			K.ATNSYFAIVADGSADNTLK.N
1746	▶1	979.4799	1956.9452	1956.9429	0.0024	0	104	4.5e-11	▶1	U				■			K.ATNSYFAIVADGSADNTLK.N
1757		666.3447	1996.0123	1996.0113	0.0010	1	32	0.00058	▶1	U	■						K.ALYLDEKGNLTQTGSGTTK.A
1758		999.0140	1996.0134	1996.0113	0.0022	1	48	1.7e-05	▶1	U	■						K.ALYLDEKGNLTQTGSGTTK.A
1765		695.7155	2084.1247	2084.1225	0.0021	0	63	3.1e-06	▶1		■	■			■	■	M.AQVINTNSLSLiTQNNiNK.N
1766		1043.0700	2084.1254	2084.1225	0.0029	0	97	1.3e-09	▶1		■	■			■	■	M.AQVINTNSLSLiTQNNiNK.N
1785		1091.5530	2181.0914	2181.0914	0.0001	0	91	8.5e-10	▶1	U	■						K.GSVANTAATTDNLTLAGFTAGTK.A
1786		728.0378	2181.0916	2181.0914	0.0002	0	44	4.1e-05	▶1	U	■						K.GSVANTAATTDNLTLAGFTAGTK.A
1806	▶1	1125.0540	2248.0934	2248.0931	0.0003	0	124	2.3e-12	▶1		■	■	■				R.LDSAVTNLNNTTTNLSEAQSR.I
1807		750.3719	2248.0939	2248.0931	0.0008	0	91	5.2e-09	▶1		■	■	■				R.LDSAVTNLNNTTTNLSEAQSR.I
1811		761.7325	2282.1757	2282.0550	0.1207	0	3	0.55	▶1	U		■					K.DGSLTAADDGAALYLDDTGNLSK.T
1823		877.0995	2628.2767	2628.2739	0.0028	0	29	0.0053	▶1		■	■	■	■	■		R.NANDGISVAQTTEGALSEINNNLQR
1824		1315.1470	2628.2794	2628.2739	0.0055	0	58	8.2e-06	▶1		■	■	■	■	■		R.NANDGISVAQTTEGALSEINNNLQR

▶55 subsets and intersections (162 subset proteins in total)

10 per page 1

Not what you expected? Try **the select summary**.

Mascot: http://www.matrixscience.com/
