# Supplementary material for: MS-H: A Novel Proteomic Approach to Isolate and Type the E. coli H Antigen Using Membrane Filtration and Liquid Chromatography-Tandem Mass Spectrometry (LC-MS/MS)
Source: PLoS One. 2013 Feb 21;8(2):e57339. doi: 10.1371/journal.pone.0057339 (PMC3578835; doi:10.1371/journal.pone.0057339)
Supplement: Representative Peptide Data S1 — Peptide data are represented as the Mascot search results from all 53 serotypes, obtained under the Orbitrap platform in Table 4 with related E. coli reference strains. “U” denotes a unique peptide specific for each of the proteins 1.1, 1.2, and beyond. The number 1.1 (shown as 1 in the peptide list and phylogenetic tree) represents the protein which obtained the highest score and confidence value after a Mascot search. This protein, known as the first hit, was used to designate the MS-H type of the unknown flagellin. Related peptides 1.2 (2), 1.3 (3), etc. represented the second, third, etc. hits for MS-H typing analysis. (DOCX) [file pone.0057339.s009.docx › H20-E188.pdf]

# MATRIX SCIENCE MASCOT Search Results

**User** :
**E-mail** :
**Search title** : Submitted from 20110728-h11-21 by Mascot Daemon on VARIABLE
**MS data file** : C:\Documents and Settings\keding\Desktop\Raw data\20110727-h11-21\20110728-028-EC188MS1.RAW
**Database** : Flagellin_v2 (192 sequences; 89,845 residues)
**Taxonomy** : Bacteria (Eubacteria) (192 sequences)
**Timestamp** : 29 Jul 2011 at 14:20:17 GMT

Not what you expected? Try **the select summary**.

▶ **Search parameters**

▶ **Score distribution**

▶ **Legend**

## Protein Family Summary

Significance threshold p< 0.05 Max. number of families AUTO
Ions score or expect cut-off 0 Dendrograms cut at 0

## Protein families 1–2 (out of 2)

10 per page 1

▼**1**

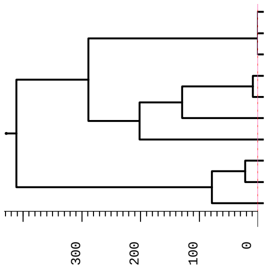


| | | | |
|---|---|---|---|
| 1 | gi\|30059878 | 4523 | H20 0\|H20\|H3306 |
| 10 | gi\|99645039 | 536 | H27 0\|H27\|0 |
| 2 | gi\|6009849 | 2410 | H49 0\|H49\|U11a-44 |
| 9 | gi\|33590243 | 626 | H16 NM\|O157:NM(H16)\|PC02 |
| 3 | gi\|218361276 | 2012 | Hxx(H120 100.0%) 0\|0\|IAI1 [gi\|323948196\|ref\|EGB44184\| H120 0\|H120\|H120] [gi\|218554508\|... |
| 8 | gi\|224579297 | 871 | H17 0\|O15:K-:H17\|P12b |
| 6 | gi\|30577587 | 1058 | H17 0\|O15:H17\|P12b |
| 4 | gi\|5107845 | 1712 | H7 0\|O55:H7\|DEC 5d [gi\|5107857\|ref\|AAD28529\| Hxx 0\|0\|ECOR 37] |
| 7 | gi\|5107837 | 975 | H7 0\|O128:H7\|DEC 13a |
| 5 | gi\|121144335 | 1652 | H55 0\|O75:H55\|E2987-73 |

Threshold (**0**): 0

| | | Score | Mass | Matches | Sequences | emPAI |
|---|---|---|---|---|---|---|
| ☑ **1.1** | gi\|30059878<br>H20 0\|H20\|H3306 | 4523 | 58613 | 117 (95) | 41 (40) | 22.75 |
| ☑ **1.2** | gi\|6009849<br>H49 0\|H49\|U11a-44 | 2410 | 58493 | 71 (49) | 25 (20) | 4.45 |
| ☑ **1.3** | gi\|218361276<br>Hxx(H120 100.0%) 0\|0\|IAI1 [gi\|323948196\|ref\|EGB44184\| H120 0\|H120\|H120] [gi\|218554508\|ref\|YP_002387421\| Hxx 0\|0\|IAI1]<br>▶ **2 samesets of gi\|218361276** | 2012 | 62286 | 61 (41) | 21 (18) | 3.22 |
| ☑ **1.4** | gi\|5107845<br>H7 0\|O55:H7\|DEC 5d [gi\|5107857\|ref\|AAD28529\| Hxx 0\|0\|ECOR 37] | 1712 | 56638 | 59 (40) | 19 (17) | 3.35 |
| ☑ **1.5** | gi\|121144335<br>H55 0\|O75:H55\|E2987-73 | 1652 | 62285 | 54 (36) | 18 (15) | 2.44 |
| ☑ **1.6** | gi\|30577587<br>H17 0\|O15:H17\|P12b | 1058 | 36285 | 37 (25) | 13 (10) | 3.42 |
| ☑ **1.7** | gi\|5107837<br>H7 0\|O128:H7\|DEC 13a | 975 | 56230 | 45 (27) | 15 (13) | 1.94 |
| ☑ **1.8** | gi\|224579297<br>H17 0\|O15:K-:H17\|P12b | 871 | 49533 | 24 (15) | 8 (7) | 1.17 |
| ☑ **1.9** | gi\|33590243<br>H16 NM\|O157:NM(H16)\|PC02<br>▶ **2 samesets of gi\|33590243** | 626 | 55093 | 24 (11) | 10 (6) | 0.69 |
| ☑ **1.10** | gi\|99645039<br>H27 0\|H27\|0 | 536 | 50847 | 25 (11) | 12 (7) | 0.87 |

▼**163 peptide matches (93 non-duplicate, 70 duplicate)**

| Query | Dupes | Observed | Mr(expt) | Mr(calc) | Delta | M | Score | Expect | Rank | U | 1 | 2 | 3 | 4 | 5 | 6 | 7 | 8 | 9 | 10 | Peptide |
|---|---|---|---|---|---|---|---|---|---|---|---|---|---|---|---|---|---|---|---|---|---|
| 4 | | 302.1612 | 602.3078 | 602.3388 | -0.0309 | 0 | 8 | 0.24 | ▶2 | U | | ■ | | | | | | | | | K.GTIANK.A |
| 4 | | 302.1612 | 602.3078 | 603.3592 | -1.0513 | 0 | 4 | 0.61 | ▶4 | U | | | ■ | | | | | | | | K.VTIGSK.S |
| 4 | | 302.1612 | 602.3078 | 602.3024 | 0.0055 | 0 | 2 | 0.96 | ▶5 | | | | | ■ | | | ■ | | | | K.LGNGDK.V |
| 21 | ▶3 | 316.6909 | 631.3672 | 631.3653 | 0.0019 | 0 | 32 | 0.0066 | ▶1 | | ■ | ■ | ■ | ■ | ■ | ■ | ■ | ■ | ■ | ■ | R.LSSGLR.I |
| 42 | ▶1 | 332.7059 | 663.3972 | 663.3955 | 0.0017 | 0 | 23 | 0.0052 | ▶1 | U | ■ | | | | | | | | | | K.LYLQK.D |
| 56 | ▶2 | 338.2067 | 674.3988 | 674.3963 | 0.0025 | 0 | 13 | 0.06 | ▶1 | U | | | | | | | | | | ■ | K.TVTGLGK.T |
| 86 | ▶4 | 352.2039 | 702.3932 | 701.4072 | 0.9861 | 0 | 34 | 0.00058 | ▶1 | U | ■ | | | | | | | | | | K.NGLTAVK.S |
| 92 | ▶1 | 355.1988 | 708.3830 | 708.3806 | 0.0024 | 0 | 17 | 0.13 | ▶1 | | ■ | ■ | ■ | ■ | ■ | ■ | ■ | ■ | | | R.FTSNIK.G |
| 95 | ▶1 | 358.7070 | 715.3994 | 715.3977 | 0.0018 | 0 | 29 | 0.0089 | ▶1 | | ■ | ■ | ■ | ■ | ■ | ■ | ■ | ■ | | | K.GLTQAAR.N |
| 105 | | 366.2074 | 730.4002 | 731.3926 | -0.9923 | 0 | 1 | 2.6 | ▶1 | | | | | | | | | | ■ | ■ | K.GLTQASR.N |
| 124 | | 380.2043 | 758.3940 | 758.4174 | -0.0233 | 0 | 29 | 0.0079 | ▶1 | U | | | | | | | | | ■ | | K.LDEALAK.V |
| 128 | ▶4 | 380.6961 | 759.3776 | 759.3763 | 0.0014 | 0 | 38 | 0.00094 | ▶1 | | ■ | ■ | ■ | ■ | ■ | ■ | ■ | | | | R.LDEIDR.V |
| 139 | ▶1 | 382.2144 | 762.4142 | 762.4123 | 0.0019 | 0 | 33 | 0.00049 | ▶1 | U | ■ | | | | | | | | | | K.IDSSTLK.L |

| Query | Dupes | Observed | Mr(expt) | Mr(calc) | Delta | M | Score | Expect | Rank | U | 1 | 2 | 3 | 4 | 5 | 6 | 7 | 8 | 9 | 10 | Peptide |
|---|---|---|---|---|---|---|---|---|---|---|---|---|---|---|---|---|---|---|---|---|---|
| 266 | | 423.2230 | 844.4314 | 844.4402 | -0.0088 | 0 | 13 | 0.046 | 1 | U | | ■ | | | | | | | | | K.AAAGAESIR.Y |
| 343 | | 446.2614 | 890.5082 | 890.5073 | 0.0010 | 1 | 29 | 0.0038 | 1 | U | ■ | | | | | | | | | | K.KIDSSTLK.L |
| 426 | 4 | 473.2595 | 944.5044 | 944.5039 | 0.0005 | 0 | 73 | 1.5e-07 | 1 | | ■ | ■ | | ■ | | | ■ | | | | R.SSLGAIQNR.L |
| 459 | | 480.2317 | 958.4488 | 958.4832 | -0.0344 | 0 | 3 | 0.89 | 1 | U | | | | | | | | | | ■ | R.SDLGAVQNR.F |
| 513 | 1 | 490.7260 | 979.4374 | 979.4458 | -0.0084 | 0 | 3 | 0.75 | 1 | U | | | ■ | | | | | | | | K.DGTITSSDGK.A |
| 521 | | 493.2287 | 984.4428 | 985.5444 | -1.1016 | 0 | 0 | 1 | 1 | U | | | | | | | | | | ■ | K.SEGGSPILVK.E |
| 527 | | 493.7546 | 985.4946 | 985.5556 | -0.0610 | 0 | 7 | 0.18 | 1 | U | | | | | ■ | | | | | | K.AAASNVLAAAK.N |
| 566 | 1 | 502.2625 | 1002.5104 | 1002.5094 | 0.0010 | 1 | 47 | 0.00012 | 1 | | ■ | ■ | ■ | ■ | ■ | ■ | ■ | | | | K.SRLDEIDR.V |
| 567 | | 335.1780 | 1002.5122 | 1002.5094 | 0.0028 | 1 | 26 | 0.013 | 1 | | ■ | ■ | ■ | ■ | ■ | ■ | ■ | | | | K.SRLDEIDR.V |
| 675 | 2 | 539.2703 | 1076.5260 | 1077.4873 | -0.9612 | 0 | 14 | 0.062 | 1 | U | | | | | | | | | ■ | | K.NDGSAQAIMR.E + Oxidation (M) |
| 677 | | 539.2804 | 1076.5462 | 1076.5462 | 0.0001 | 0 | 41 | 9.9e-05 | 1 | U | | | | ■ | | | | | | | -.QSALSSSIER.L |
| 719 | 2 | 551.2682 | 1100.5218 | 1100.5210 | 0.0008 | 0 | 78 | 1.4e-07 | 1 | | ■ | ■ | ■ | ■ | ■ | ■ | | ■ | ■ | ■ | K.DDAAGQAIANR.F |
| 739 | | 557.8187 | 1113.6228 | 1113.6393 | -0.0165 | 1 | 0 | 0.94 | 1 | U | | | | | | ■ | | | | | K.ALDAAIAKVDK.F |
| 850 | | 390.1811 | 1167.5215 | 1166.5819 | 0.9395 | 0 | 1 | 0.84 | 1 | U | | | | | | | | | | ■ | K.DVTFTIDATGK.D |
| 877 | | 396.8673 | 1187.5801 | 1187.6034 | -0.0233 | 0 | 0 | 0.98 | 1 | U | | | | | ■ | | | | | | K.ALDDAISQIDK.F |
| 883 | | 596.3022 | 1190.5898 | 1190.5891 | 0.0008 | 0 | 81 | 4.6e-08 | 1 | | ■ | ■ | ■ | | | | | | | ■ | K.NQSALSSSIER.L |
| 884 | | 397.8896 | 1190.6470 | 1190.5891 | 0.0579 | 0 | 0 | 5 | 1 | | ■ | ■ | ■ | | | | | | | ■ | K.NQSALSSSIER.L |
| 889 | | 598.8018 | 1195.5890 | 1194.5517 | 1.0374 | 0 | 7 | 0.2 | 1 | U | | ■ | | | | | | | | | K.DAAQSSIDFGGK.K |
| 897 | | 600.8536 | 1199.6926 | 1199.6734 | 0.0192 | 1 | 10 | 0.1 | 1 | U | | | | | | | | | ■ | | K.LRSSLGAVQNR.F |
| 973 | | 627.8044 | 1253.5942 | 1254.6244 | -1.0302 | 0 | 2 | 0.62 | 1 | U | | ■ | | | | | | | | | K.FNALDAATAFSK.L |
| 1006 | 2 | 636.3050 | 1270.5954 | 1270.5942 | 0.0013 | 0 | 68 | 1.5e-07 | 1 | U | ■ | | | | | | | | | | K.NGFAAGATSNAYK.L |
| 1066 | 1 | 651.8627 | 1301.7108 | 1302.6415 | -0.9307 | 0 | 7 | 0.48 | 1 | U | | | | | | | | | | ■ | K.AATASDLDLNNAK.K |
| 1086 | | 656.8657 | 1311.7168 | 1311.7146 | 0.0022 | 0 | 35 | 0.00031 | 1 | U | | | | | | | ■ | | | | K.AQIIQQAGNSVLA.- |
| 1143 | | 672.8789 | 1343.7432 | 1343.7408 | 0.0024 | 0 | 72 | 6.4e-08 | 1 | U | | | | | ■ | | | | | | -.SLSLITQNNINK.N |
| 1263 | | 480.9438 | 1439.8096 | 1439.8096 | -0.0000 | 0 | 28 | 0.0069 | 1 | | ■ | ■ | ■ | ■ | ■ | | | | | | K.AQIIQQAGNSVLAK.A |
| 1265 | 3 | 720.9125 | 1439.8104 | 1439.8096 | 0.0008 | 0 | 117 | 9.3e-12 | 1 | | ■ | ■ | ■ | ■ | ■ | | | | | | K.AQIIQQAGNSVLAK.A |
| 1331 | 1 | 747.9188 | 1493.8230 | 1493.8202 | 0.0029 | 0 | 66 | 1.6e-06 | 1 | | ■ | ■ | ■ | | | ■ | | | | | K.ANQVPQQVLSLLQG.- |
| 1410 | 2 | 781.4216 | 1560.8286 | 1560.8260 | 0.0026 | 0 | 62 | 2.9e-06 | 1 | | ■ | ■ | | ■ | ■ | | ■ | | | | R.VSGQTQFNGVNVLAK.D |
| 1465 | | 807.9142 | 1613.8138 | 1613.8121 | 0.0017 | 1 | 89 | 1.2e-08 | 1 | | ■ | ■ | ■ | ■ | ■ | ■ | | ■ | ■ | ■ | R.INSAKDDAAGQAIANR.F |
| 1466 | | 538.9453 | 1613.8141 | 1613.8121 | 0.0020 | 1 | 31 | 0.0078 | 1 | | ■ | ■ | ■ | ■ | ■ | ■ | | ■ | ■ | ■ | R.INSAKDDAAGQAIANR.F |
| 1480 | | 542.9459 | 1625.8159 | 1625.8161 | -0.0003 | 1 | 40 | 9.1e-05 | 1 | U | ■ | | | | | | | | | | K.NGFAAGATSNAYKLNK.D |
| 1481 | | 813.9157 | 1625.8168 | 1625.8161 | 0.0007 | 1 | 95 | 3.1e-10 | 1 | U | ■ | | | | | | | | | | K.NGFAAGATSNAYKLNK.D |
| 1502 | | 823.9493 | 1645.8840 | 1645.8787 | 0.0053 | 0 | 40 | 0.0001 | 1 | U | | | ■ | | | | | | | | K.NVQFAAATASNVLAAAK.D |
| 1527 | 1 | 836.3806 | 1670.7466 | 1670.7457 | 0.0009 | 0 | 128 | 1e-12 | 1 | | ■ | ■ | ■ | ■ | ■ | ■ | ■ | ■ | | | R.IQDADYATEVSNMSK.A |
| 1529 | | 557.9233 | 1670.7481 | 1670.7457 | 0.0023 | 0 | 57 | 1.3e-05 | 1 | | ■ | ■ | ■ | ■ | ■ | ■ | ■ | ■ | | | R.IQDADYATEVSNMSK.A |
| 1547 | 5 | 843.4579 | 1684.9012 | 1684.8996 | 0.0017 | 0 | 113 | 2.1e-11 | 1 | | ■ | ■ | | | | | | | | | K.IQVGANDGQTITIDLK.K |
| 1547 | 4 | 843.4579 | 1684.9012 | 1685.8836 | -0.9823 | 0 | 34 | 0.0014 | 2 | | | | ■ | ■ | ■ | | ■ | | | | K.IQVGANDGETITIDLK.K |
| 1552 | | 844.3779 | 1686.7412 | 1686.7407 | 0.0006 | 0 | 111 | 5.9e-11 | 1 | | ■ | ■ | ■ | ■ | ■ | ■ | ■ | ■ | | | R.IQDADYATEVSNMSK.A + Oxidation (M) |
| 1553 | | 563.2545 | 1686.7417 | 1686.7407 | 0.0010 | 0 | 35 | 0.0023 | 1 | | ■ | ■ | ■ | ■ | ■ | ■ | ■ | ■ | | | R.IQDADYATEVSNMSK.A + Oxidation (M) |
| 1578 | 1 | 854.4325 | 1706.8504 | 1706.8475 | 0.0029 | 0 | 104 | 3.8e-11 | 1 | U | | | ■ | | | | | | | | K.DAYGNSAAAAVGTVIEAK.G |
| 1591 | | 572.2979 | 1713.8719 | 1715.0089 | -1.1370 | 0 | 7 | 0.19 | 1 | U | | ■ | | | | | | | | | K.LTLMLMQAVISLLAAK.R |
| 1597 | | 860.3581 | 1718.7016 | 1718.7974 | -0.0957 | 0 | 8 | 0.17 | 1 | U | | | | | | | | | ■ | | K.ALAYNDAPMSVYFGGK.N + Oxidation (M) |
| 1620 | | 581.6327 | 1741.8763 | 1740.8530 | 1.0233 | 0 | 4 | 0.36 | 1 | U | | | | | | ■ | | | | | K.QVNLLSYTDTASNSTK.Y |
| 1647 | 3 | 887.4141 | 1772.8136 | 1772.8105 | 0.0032 | 0 | 75 | 4.6e-08 | 1 | U | ■ | | | | | | | | | | K.AVYVEADGDFTTDAATK.A |
| 1681 | 1 | 902.4167 | 1802.8188 | 1802.8170 | 0.0018 | 0 | 82 | 2.7e-08 | 1 | U | ■ | | | | | | | | | | K.ASYTNTDGTLTTDNTTK.L |
| 1688 | 2 | 904.9601 | 1807.9056 | 1807.9064 | -0.0008 | 0 | 123 | 6.8e-13 | 1 | U | ■ | | | | | | | | | | K.LTGFNVNGSGSVANTAATK.A |
| 1690 | 1 | 603.6437 | 1807.9093 | 1807.9064 | 0.0028 | 0 | 57 | 2.6e-06 | 1 | U | ■ | | | | | | | | | | K.LTGFNVNGSGSVANTAATK.A |
| 1696 | | 605.3396 | 1812.9970 | 1812.9945 | 0.0024 | 1 | 32 | 0.0026 | 1 | | ■ | ■ | | | | | | | | | K.IQVGANDGQTITIDLKK.I |
| 1696 | | 605.3396 | 1812.9970 | 1813.9785 | -0.9816 | 1 | 29 | 0.0052 | 2 | | | | ■ | ■ | ■ | | ■ | | | | K.IQVGANDGETITIDLKK.I |
| 1783 | | 979.4800 | 1956.9454 | 1956.9429 | 0.0026 | 0 | 99 | 1.2e-10 | 1 | U | | | | | | | | ■ | | | K.ATNSYFAIVADGSADNTLK.N |
| 1823 | | 1015.5210 | 2029.0274 | 2029.0215 | 0.0059 | 0 | 114 | 3.8e-12 | 1 | U | ■ | | | | | | | | | | K.AATTTDPLAALDDAISQIDK.F |
| 1846 | | 695.7159 | 2084.1259 | 2084.1225 | 0.0033 | 0 | 77 | 1.3e-07 | 1 | | ■ | ■ | ■ | | | | | | ■ | ■ | M.AQVINTNSLSLiTQNNiNK.N |
| 1846 | | 695.7159 | 2084.1259 | 2085.0814 | -0.9555 | 0 | 58 | 1e-05 | 5 | U | | | | | | ■ | | | | | M.AQVINTNSLSLNTQNNINK.N |
| 1847 | 1 | 1043.3160 | 2084.6174 | 2084.1225 | 0.4949 | 0 | 141 | 4.8e-14 | 1 | | ■ | ■ | ■ | | | | | | ■ | ■ | M.AQVINTNSLSLiTQNNiNK.N |
| 1847 | 1 | 1043.3160 | 2084.6174 | 2085.0814 | -0.4640 | 0 | 79 | 7.5e-08 | 5 | U | | | | | | ■ | | | | | M.AQVINTNSLSLNTQNNINK.N |
| 1849 | | 698.3722 | 2092.0948 | 2092.0900 | 0.0048 | 0 | 101 | 8.8e-11 | 1 | U | ■ | | | | | | | | | | K.TTAADVLSSLADGTTITATGVK.N |
| 1850 | | 1047.0550 | 2092.0954 | 2092.0900 | 0.0055 | 0 | 123 | 5.4e-13 | 1 | U | ■ | | | | | | | | | | K.TTAADVLSSLADGTTITATGVK.N |
| 1919 | 2 | 1125.0560 | 2248.0974 | 2248.0931 | 0.0043 | 0 | 137 | 1.1e-13 | 1 | | ■ | ■ | ■ | ■ | ■ | ■ | | | | | R.LDSAVTNLNNTTTNLSEAQSR.I |
| 1921 | | 750.3732 | 2248.0978 | 2248.0931 | 0.0047 | 0 | 98 | 1.1e-09 | 1 | | ■ | ■ | ■ | ■ | ■ | ■ | | | | | R.LDSAVTNLNNTTTNLSEAQSR.I |
| 1946 | | 768.4056 | 2302.1950 | 2302.1917 | 0.0032 | 1 | 61 | 3.4e-06 | 1 | | ■ | ■ | | ■ | ■ | | ■ | | | | R.LDEIDRVSGQTQFNGVNVLAK.D |
| 1947 | | 1152.1050 | 2302.1954 | 2302.1917 | 0.0037 | 1 | 52 | 3e-05 | 1 | | ■ | ■ | | ■ | ■ | | ■ | | | | R.LDEIDRVSGQTQFNGVNVLAK.D |
| 1961 | | 778.4062 | 2332.1968 | 2332.1910 | 0.0057 | 1 | 48 | 1.7e-05 | 1 | U | ■ | | | | | | | | | | K.AATTTDPLAALDDAISQIDKFR.S |
| 1982 | 1 | 1228.0970 | 2454.1794 | 2454.1762 | 0.0032 | 0 | 60 | 9.4e-07 | 1 | U | ■ | | | | | | | | | | K.AGDTATFSVEIGGTTQDVVLSSDGK.L |
| 1996 | 1 | 865.0880 | 2592.2422 | 2592.2402 | 0.0019 | 0 | 83 | 4.6e-09 | 1 | U | ■ | | | | | | | | | | R.ELTVQATTGTNSQSDLDSIQDEIK.S |
| 1998 | 1 | 1297.1290 | 2592.2434 | 2592.2402 | 0.0032 | 0 | 131 | 7.5e-14 | 1 | U | ■ | | | | | | | | | | R.ELTVQATTGTNSQSDLDSIQDEIK.S |
| 2000 | 1 | 1298.1110 | 2594.2074 | 2594.2024 | 0.0050 | 0 | 99 | 1.2e-10 | 1 | U | ■ | | | | | | | | | | K.DNNTFTYDTTATTAELQSYLTPK.A |
| 2001 | | 865.7435 | 2594.2087 | 2594.2024 | 0.0063 | 0 | 56 | 2.8e-06 | 1 | U | ■ | | | | | | | | | | K.DNNTFTYDTTATTAELQSYLTPK.A |
| 2011 | 2 | 1315.1470 | 2628.2794 | 2628.2739 | 0.0055 | 0 | 135 | 1.5e-13 | 1 | | ■ | ■ | ■ | ■ | ■ | | ■ | ■ | ■ | | R.NANDGISVAQTTEGALSEINNNLQR |
| 2012 | 1 | 877.1007 | 2628.2803 | 2628.2739 | 0.0064 | 0 | 91 | 3.5e-09 | 1 | | ■ | ■ | ■ | ■ | ■ | | ■ | ■ | ■ | | R.NANDGISVAQTTEGALSEINNNLQR |
| 2039 | 3 | 902.4728 | 2704.3966 | 2704.3919 | 0.0046 | 0 | 49 | 1.2e-05 | 1 | U | ■ | | | | | | | | | | K.ADLAAAAIGTPGAADSTGAIAYTVSAGLTK.T |
| 2043 | 1 | 1353.2080 | 2704.4014 | 2704.3919 | 0.0095 | 0 | 82 | 6.3e-09 | 1 | U | ■ | | | | | | | | | | K.ADLAAAAIGTPGAADSTGAIAYTVSAGLTK.T |
| 2059 | | 946.1336 | 2835.3790 | 2835.3734 | 0.0056 | 1 | 59 | 4.1e-06 | 1 | U | ■ | | | | | | | | | | R.ELTVQATTGTNSQSDLDSIQDEIKSR.L |
| 2060 | | 950.1456 | 2847.4150 | 2847.4098 | 0.0052 | 1 | 89 | 2.3e-09 | 1 | U | ■ | | | | | | | | | | R.VRELTVQATTGTNSQSDLDSIQDEIK.S |
| 2060 | | 950.1456 | 2847.4150 | 2848.3938 | -0.9788 | 1 | 15 | 0.047 | 2 | | | | | ■ | | | ■ | | | | R.IRELTVQATTGTNSDSDLDSIQDEIK.S |
| 2071 | | 984.1502 | 2949.4288 | 2949.4243 | 0.0044 | 1 | 25 | 0.0031 | 1 | U | ■ | | | | | | | | | | K.LNKDNNTFTYDTTATTAELQSYLTPK.A |
| 2091 | | 1086.5760 | 3256.7062 | 3256.7011 | 0.0051 | 1 | 126 | 9.6e-13 | 1 | | ■ | ■ | ■ | | | | | | | ■ | M.AQVINTNSLSLITQNNINKNQSALSSSIER.L |
| 2092 | | 815.1840 | 3256.7069 | 3256.7011 | 0.0058 | 1 | 36 | 0.00094 | 1 | | ■ | ■ | ■ | | | | | | | ■ | M.AQVINTNSLSLITQNNINKNQSALSSSIER.L |
| 2096 | | 1645.8510 | 3289.6874 | 3289.6790 | 0.0084 | 0 | 51 | 7.4e-06 | 1 | U | ■ | | | | | | | | | | K.LYIDTTGNLTQNGGNNGVGTLAEATLSGLALNK.N |
| 2097 | | 1097.5700 | 3289.6882 | 3289.6790 | 0.0092 | 0 | 65 | 2.9e-07 | 1 | U | ■ | | | | | | | | | | K.LYIDTTGNLTQNGGNNGVGTLAEATLSGLALNK.N |
| 2112 | | 1116.2300 | 3345.6682 | 3344.6736 | 0.9946 | 1 | 19 | 0.011 | 1 | U | ■ | | | | | | | | | | K.TTAADVLSSLADGTTITATGVKNGFAAGATSNAYK.L |

62 subsets and intersections (157 subset proteins in total)

| | | | |
|---|---|---|---|
| **2** | **gi\|46093564** | 15 | Hxx(H54 100.0%) 0\|0\|E223-69 [gi\|283982455\|ref\|ADB56974\| H54 0\|O161:H54\|O161:H54] |

10 per page 1

---

Not what you expected? Try **the select summary**.

**Mascot: http://www.matrixscience.com/**
