# Supplementary material for: MS-H: A Novel Proteomic Approach to Isolate and Type the E. coli H Antigen Using Membrane Filtration and Liquid Chromatography-Tandem Mass Spectrometry (LC-MS/MS)
Source: PLoS One. 2013 Feb 21;8(2):e57339. doi: 10.1371/journal.pone.0057339 (PMC3578835; doi:10.1371/journal.pone.0057339)
Supplement: Representative Peptide Data S1 — Peptide data are represented as the Mascot search results from all 53 serotypes, obtained under the Orbitrap platform in Table 4 with related E. coli reference strains. “U” denotes a unique peptide specific for each of the proteins 1.1, 1.2, and beyond. The number 1.1 (shown as 1 in the peptide list and phylogenetic tree) represents the protein which obtained the highest score and confidence value after a Mascot search. This protein, known as the first hit, was used to designate the MS-H type of the unknown flagellin. Related peptides 1.2 (2), 1.3 (3), etc. represented the second, third, etc. hits for MS-H typing analysis. (DOCX) [file pone.0057339.s009.docx › H21-E189.pdf]

# MASCOT Search Results

**User** :
**E-mail** :
**Search title** : Submitted from 20110728-h11-21 by Mascot Daemon on VARIABLE
**MS data file** : C:\Documents and Settings\keding\Desktop\Raw data\20110727-h11-21\20110728-029-EC189MS1.RAW
**Database** : Flagellin_v2 (192 sequences; 89,845 residues)
**Taxonomy** : Bacteria (Eubacteria) (192 sequences)
**Timestamp** : 29 Jul 2011 at 14:01:20 GMT

Not what you expected? Try **the select summary**.

**Search parameters**
**Score distribution**
**Legend**

## Protein Family Summary

Significance threshold p< 0.05 Max. number of families AUTO
Ions score or expect cut-off 0 Dendrograms cut at 0

## Protein families 1–2 (out of 2)

10 per page 1

**1**

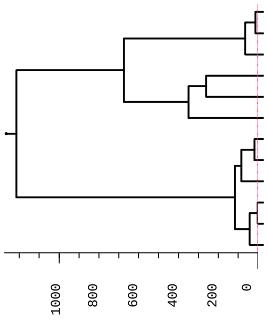

| | | | |
|---|---|---|---|
| 1 | gi\|112820171 | 6862 | H21 0\|EHEC serogroup: O113:H21\|0 [gi\|291323135\|ref\|EFE62563\| Hxx 0\|0\|B088] [gi\|29344630... |
| 2 | gi\|33590237 | 4813 | H11 NM\|O157:NM(H11)\|F199 |
| 4 | gi\|62532942 | 2815 | H11 0\|O26:H11\|0 |
| 3 | gi\|40353143 | 3118 | H2 0\|O111:H2\|5721/96 |
| 6 | gi\|30059872 | 1602 | H16 0\|H16\|F 8316-41 |
| 7 | gi\|46093552 | 1384 | H53 0\|0\|E480-68 |
| 11 | gi\|30577587 | 640 | H17 0\|O15:H17\|P12b |
| 10 | gi\|33590227 | 723 | H42 NM\|O157:NM(H42)\|25 |
| 12 | gi\|121144335 | 583 | H55 0\|O75:H55\|E2987-73 |
| 5 | gi\|30059878 | 1702 | H20 0\|H20\|H3306 |
| 8 | gi\|16901498 | 1015 | H34 0\|H34\|0 |
| 9 | gi\|290439 | 827 | Hxx(H7 98.6%) 0\|0\|U5-41 (Orskov) |

Threshold (**0**): 0

| | | Score | Mass | Matches | Sequences | emPAI |
|---|---|---|---|---|---|---|
| 1.1 | **gi\|112820171** | 6862 | 51472 | 188 (164) | 50 (49) | 110.42 |
| | H21 0\|EHEC serogroup: O113:H21\|0 [gi\|291323135\|ref\|EFE62563\| Hxx 0\|0\|B088] [gi\|293446305\|ref\|ZP_06662727\| Hxx 0\|0\|B088] | | | | | |
| 1.2 | **gi\|33590237** | 4813 | 50951 | 135 (115) | 39 (38) | 41.98 |
| | H11 NM\|O157:NM(H11)\|F199 | | | | | |
| 1.3 | **gi\|40353143** | 3118 | 47290 | 125 (98) | 37 (33) | 22.80 |
| | H2 0\|O111:H2\|5721/96 | | | | | |
| 1.4 | **gi\|62532942** | 2815 | 37909 | 84 (72) | 25 (25) | 19.29 |
| | H11 0\|O26:H11\|0 | | | | | |
| 1.5 | **gi\|30059878** | 1702 | 58613 | 45 (36) | 26 (22) | 3.14 |
| | H20 0\|H20\|H3306 | | | | | |
| 1.6 | **gi\|30059872** | 1602 | 52639 | 52 (35) | 19 (11) | 2.37 |
| | H16 0\|H16\|F 8316-41 | | | | | |
| 1.7 | **gi\|46093552** | 1384 | 44861 | 41 (29) | 16 (11) | 1.90 |
| | H53 0\|0\|E480-68 | | | | | |
| 1.8 | **gi\|16901498** | 1015 | 56006 | 32 (24) | 17 (13) | 1.35 |
| | H34 0\|H34\|0 | | | | | |
| | **3 samesets of gi\|16901498** | | | | | |
| 1.9 | **gi\|290439** | 827 | 59752 | 34 (21) | 20 (11) | 1.01 |
| | Hxx(H7 98.6%) 0\|0\|U5-41 (Orskov) | | | | | |
| 1.10 | **gi\|33590227** | 723 | 44094 | 30 (20) | 14 (10) | 1.38 |
| | H42 NM\|O157:NM(H42)\|25 | | | | | |
| 1.11 | **gi\|30577587** | 640 | 36285 | 22 (16) | 10 (7) | 1.20 |
| | H17 0\|O15:H17\|P12b | | | | | |
| 1.12 | **gi\|121144335** | 583 | 62285 | 26 (16) | 15 (9) | 0.67 |
| | H55 0\|O75:H55\|E2987-73 | | | | | |

**282 peptide matches (145 non-duplicate, 137 duplicate)**

| Query | Dupes | Observed | Mr(expt) | Mr(calc) | Delta | M | Score | Expect | Rank | U | 1 | 2 | 3 | 4 | 5 | 6 | 7 | 8 | 9 | 10 | 11 | 12 | Peptide |
|---|---|---|---|---|---|---|---|---|---|---|---|---|---|---|---|---|---|---|---|---|---|---|---|
| 12 | 4 | 308.6752 | 615.3358 | 615.3340 | 0.0018 | 0 | 18 | 0.031 | 1 | | ■ | ■ | ■ | ■ | | | | | | | | | K.VDNLR.S |
| 21 | 4 | 316.6906 | 631.3666 | 631.3653 | 0.0013 | 0 | 30 | 0.009 | 1 | | ■ | ■ | ■ | | ■ | ■ | ■ | ■ | ■ | ■ | ■ | ■ | R.LSSGLR.I |
| 26 | | 318.1652 | 634.3158 | 635.2949 | -0.9790 | 0 | 9 | 0.11 | 1 | U | | | | | | | | ■ | | | | | K.ADGMVK.D + Oxidation (M) |
| 65 | 1 | 347.2009 | 692.3872 | 692.3857 | 0.0015 | 0 | 26 | 0.0054 | 1 | | ■ | ■ | ■ | | | ■ | | | | | | | R.FTANIK.G |
| 86 | | 358.7138 | 715.4130 | 715.4116 | 0.0015 | 0 | 13 | 0.38 | 1 | U | | | | | | | ■ | | | | | | K.IDIDLK.K |
| 98 | 4 | 366.7046 | 731.3946 | 731.3926 | 0.0021 | 0 | 32 | 0.0021 | 1 | | ■ | ■ | ■ | | | ■ | ■ | | | | | | K.GLTQASR.N |
| 103 | | 370.7048 | 739.3950 | 739.3938 | 0.0012 | 0 | 28 | 0.002 | 1 | | ■ | ■ | ■ | ■ | | | | | | | | | K.VMYLSK.S |
| 114 | 1 | 378.7022 | 755.3898 | 755.3887 | 0.0011 | 0 | 43 | 6.4e-05 | 1 | | ■ | ■ | ■ | ■ | | | | | | | | | K.VMYLSK.S + Oxidation (M) |

| Query | Dupes | Observed | Mr(expt) | Mr(calc) | Delta | M | Score | Expect | Rank | U | 1 | 2 | 3 | 4 | 5 | 6 | 7 | 8 | 9 | 10 | 11 | 12 | Peptide |
|---|---|---|---|---|---|---|---|---|---|---|---|---|---|---|---|---|---|---|---|---|---|---|---|
| 133 | 3 | 387.7033 | 773.3920 | 773.3919 | 0.0001 | 0 | 36 | 0.00058 | 1 | | ■ | ■ | ■ | ■ | | | | | | | | | R.LEEIDR.V |
| 163 | | 401.7272 | 801.4398 | 801.4385 | 0.0014 | 0 | 4 | 0.44 | 1 | U | | | | | | ■ | | | | | | | K.AVEWAVK.N |
| 188 | 1 | 409.2244 | 816.4342 | 816.4341 | 0.0001 | 0 | 42 | 6.5e-05 | 1 | U | ■ | | | | | | | | | | | | K.LTANVDGK.A |
| 315 | | 446.2427 | 890.4708 | 890.4709 | -0.0001 | 0 | 45 | 9.5e-05 | 1 | | ■ | ■ | ■ | ■ | | | | | | | | | K.ATGSDLISK.F |
| 340 | | 453.7381 | 905.4616 | 904.5052 | 0.9565 | 1 | 0 | 0.9 | 4 | U | | | | | | | | | ■ | | | | K.ADMKALLK.A + Oxidation (M) |
| 366 | | 466.7442 | 931.4738 | 930.4883 | 0.9856 | 0 | 3 | 2.3 | 1 | | | | | | | ■ | | | ■ | | ■ | ■ | R.SSlGAvQNR |
| 388 | | 473.2594 | 944.5042 | 944.5039 | 0.0003 | 0 | 59 | 4.1e-06 | 1 | | | | | | ■ | | ■ | ■ | | | | | R.SSLGAIQNR |
| 418 | 6 | 480.2490 | 958.4834 | 958.4832 | 0.0002 | 0 | 67 | 3.7e-07 | 1 | | ■ | ■ | ■ | ■ | | | | | | | | | R.SDLGAVQNR.F |
| 418 | 4 | 480.2490 | 958.4834 | 958.5196 | -0.0361 | 0 | 32 | 0.0011 | 2 | U | | | | | | | | | | ■ | | | R.SSLGVVQNR.L |
| 467 | | 493.7542 | 985.4938 | 985.5556 | -0.0618 | 0 | 2 | 0.57 | 1 | U | | | | | | | | | | | | ■ | K.AAASNVLAAAK.N |
| 475 | 3 | 495.7649 | 989.5152 | 989.5142 | 0.0011 | 0 | 63 | 6.9e-07 | 1 | U | ■ | | | | | | | | | | | | K.NSAGQLTATK.V |
| 529 | | 508.7074 | 1015.4002 | 1014.5709 | 0.8293 | 0 | 6 | 0.27 | 1 | U | | | | | | ■ | | | | | | | K.ALATTNPLSK.L |
| 569 | 2 | 524.2607 | 1046.5068 | 1046.5066 | 0.0002 | 0 | 32 | 0.0011 | 1 | | ■ | ■ | ■ | ■ | | | | | | | | | K.VLAENNEMK.I |
| 585 | | 529.3038 | 1056.5930 | 1056.5927 | 0.0003 | 0 | 13 | 0.049 | 1 | U | | | ■ | | | | | | | | | | -.LLTQNNLNK.S |
| 592 | | 532.2584 | 1062.5022 | 1062.5015 | 0.0007 | 0 | 37 | 0.00038 | 1 | | ■ | ■ | ■ | ■ | | | | | | | | | K.VLAENNEMK.I + Oxidation (M) |
| 608 | 2 | 539.2700 | 1076.5254 | 1077.4873 | -0.9618 | 0 | 12 | 0.095 | 1 | U | | | | | | ■ | | | | | | | K.NDGSAQAIMR.E + Oxidation (M) |
| 610 | | 539.2751 | 1076.5356 | 1075.5411 | 0.9946 | 0 | 1 | 1 | 1 | U | | | | | | ■ | | | | | | | K.DVQLANFGGR.V |
| 659 | 4 | 551.2673 | 1100.5200 | 1100.5210 | -0.0010 | 0 | 76 | 2.2e-07 | 1 | | ■ | ■ | ■ | | ■ | ■ | ■ | ■ | ■ | ■ | ■ | ■ | K.DDAAGQAIANR.F |
| 702 | | 562.2932 | 1122.5718 | 1122.5921 | -0.0202 | 1 | 2 | 0.69 | 1 | U | | | ■ | | | | | | | | | | K.LADKGSIEYK.G |
| 724 | 3 | 568.2838 | 1134.5530 | 1134.5517 | 0.0014 | 0 | 55 | 5.7e-06 | 1 | | ■ | ■ | | ■ | | | | | | | | | K.TITETASGNNK.V |
| 741 | | 572.8188 | 1143.6230 | 1144.6564 | -1.0333 | 1 | 1 | 7.6 | 1 | | ■ | ■ | ■ | | ■ | ■ | ■ | ■ | ■ | ■ | ■ | ■ | R.LSSGLRINSAK.D |
| 784 | 2 | 582.7964 | 1163.5782 | 1163.5782 | 0.0000 | 0 | 64 | 1.2e-06 | 1 | | ■ | ■ | ■ | | | ■ | ■ | | | | | | K.SQSSLSSAIER.L |
| 792 | | 583.8246 | 1165.6346 | 1165.6343 | 0.0004 | 1 | 34 | 0.00085 | 1 | | ■ | ■ | ■ | ■ | | | | | | | | | K.ATGSDLISKFK.A |
| 803 | | 587.3150 | 1172.6154 | 1172.6149 | 0.0005 | 1 | 38 | 0.00015 | 1 | U | ■ | | | | | | | | | | | | K.GNGKLTANVDGK.A |
| 812 | | 394.5319 | 1180.5739 | 1179.6499 | 0.9239 | 0 | 8 | 0.17 | 1 | U | | | | | | | | | ■ | | | | K.LNTTTGLYGLK.T |
| 823 | | 596.3020 | 1190.5894 | 1190.5891 | 0.0004 | 0 | 59 | 6.5e-06 | 1 | | | | | | ■ | | | ■ | ■ | | | | K.NQSALSSSIER.L |
| 842 | | 600.8535 | 1199.6924 | 1199.6734 | 0.0190 | 1 | 10 | 0.091 | 1 | U | | | | | | ■ | | | | | | | K.LRSSLGAVQNR.F |
| 889 | | 617.8194 | 1233.6242 | 1234.6558 | -1.0315 | 0 | 0 | 4 | 1 | U | | | | | | | | | ■ | | | | K.VTVGGVDIYNAK.S |
| 896 | 1 | 618.8148 | 1235.6150 | 1235.6146 | 0.0004 | 0 | 60 | 2.1e-06 | 1 | | ■ | ■ | ■ | ■ | | | | | | | | | R.VSEQTQFNGVK.V |
| 956 | | 636.3052 | 1270.5958 | 1270.5942 | 0.0017 | 0 | 53 | 5.2e-06 | 1 | U | | | | | ■ | | | | | | | | K.NGFAAGATSNAYK.L |
| 1011 | 3 | 651.8492 | 1301.6838 | 1301.6827 | 0.0012 | 0 | 77 | 4.4e-08 | 1 | | ■ | ■ | ■ | ■ | | | | | | | | | K.AATLSDLDLNAAK.K |
| 1085 | | 672.8768 | 1343.7390 | 1343.7408 | -0.0018 | 0 | 74 | 3.7e-08 | 1 | U | | | | | | | | | | | | ■ | -.SLSLITQNNINK.N |
| 1111 | | 683.3242 | 1364.6338 | 1364.6783 | -0.0445 | 0 | 0 | 0.9 | 1 | U | | | | | | | | | | | | ■ | K.GSVSNTAATTDTLK.L |
| 1112 | | 683.3364 | 1364.6582 | 1364.6572 | 0.0010 | 0 | 76 | 2.3e-08 | 1 | U | | | | ■ | | | | | | | | | R.FDSAITNLGNTVN.- |
| 1138 | | 695.8451 | 1389.6756 | 1390.7303 | -1.0547 | 1 | 2 | 0.64 | 1 | U | | | | | | ■ | | | | | | | K.TATKGAELSASDLK.A |
| 1155 | | 703.8915 | 1405.7684 | 1405.7677 | 0.0007 | 1 | 45 | 0.00031 | 1 | | ■ | ■ | ■ | | | ■ | | | | | | | R.FTANIKGLTQASR.N |
| 1156 | | 469.5969 | 1405.7689 | 1405.7677 | 0.0011 | 1 | 32 | 0.0052 | 1 | | ■ | ■ | ■ | | | ■ | | | | | | | R.FTANIKGLTQASR.N |
| 1156 | | 469.5969 | 1405.7689 | 1405.7677 | 0.0011 | 1 | 2 | 6.3 | 2 | | | | | | ■ | | | ■ | ■ | ■ | ■ | ■ | R.FTSNIKGLTQAAR.N |
| 1188 | | 477.6000 | 1429.7782 | 1429.7776 | 0.0006 | 1 | 26 | 0.0037 | 1 | | ■ | ■ | ■ | ■ | | | | | | | | | K.AATLSDLDLNAAKK.T |
| 1189 | | 715.8964 | 1429.7782 | 1429.7776 | 0.0006 | 1 | 95 | 5.1e-10 | 1 | | ■ | ■ | ■ | ■ | | | | | | | | | K.AATLSDLDLNAAKK.T |
| 1199 | | 720.9119 | 1439.8092 | 1439.8096 | -0.0004 | 0 | 75 | 1.6e-07 | 1 | | | | | | ■ | | | ■ | | ■ | | ■ | K.AQIIQQAGNSVLAK.A |
| 1204 | 3 | 724.8737 | 1447.7328 | 1447.7307 | 0.0021 | 0 | 89 | 2.4e-09 | 1 | | ■ | ■ | ■ | ■ | | | | | | | | | K.TLGLDGFNIDGAQK.A |
| 1221 | | 487.5949 | 1459.7629 | 1459.7630 | -0.0002 | 1 | 35 | 0.00034 | 1 | U | ■ | | | | | | | | | | | | K.NSAGQLTATKVENK.A |
| 1222 | | 730.8892 | 1459.7638 | 1459.7630 | 0.0008 | 1 | 51 | 8.5e-06 | 1 | U | ■ | | | | | | | | | | | | K.NSAGQLTATKVENK.A |
| 1250 | 2 | 743.8722 | 1485.7298 | 1485.7311 | -0.0012 | 0 | 73 | 7.8e-08 | 1 | | ■ | ■ | ■ | ■ | | | | | | | | | K.SEGGSPILVNEDAAK.S |
| 1260 | | 746.5443 | 1491.0740 | 1491.7681 | -0.6941 | 0 | 1 | 0.81 | 1 | U | | | | | | | ■ | | | | | | K.VAANTSGLAANQTFK.S |
| 1265 | | 747.9182 | 1493.8218 | 1493.8202 | 0.0017 | 0 | 44 | 0.00026 | 1 | | | | | | ■ | | | ■ | ■ | ■ | ■ | | K.ANQVPQQVLSLLQG.- |
| 1333 | 1 | 773.9016 | 1545.7886 | 1545.7886 | 0.0000 | 0 | 72 | 1.2e-07 | 1 | | ■ | ■ | ■ | ■ | | | | | | | | | K.SLQSTTNPLETIDK.A |
| 1344 | | 778.9098 | 1555.8050 | 1555.8067 | -0.0016 | 1 | 36 | 0.00045 | 1 | | ■ | ■ | ■ | ■ | | | | | | | | | K.VDNLRSDLGAVQNR.F |
| 1345 | | 519.6091 | 1555.8055 | 1555.8067 | -0.0012 | 1 | 35 | 0.00054 | 1 | | ■ | ■ | ■ | ■ | | | | | | | | | K.VDNLRSDLGAVQNR.F |
| 1349 | | 781.0683 | 1560.1220 | 1559.6297 | 0.4923 | 0 | 5 | 1.4 | 1 | U | | | ■ | | | | | | | | | | R.IEDADYATEVSNMS.- + Oxidation (M) |
| 1351 | | 781.4208 | 1560.8270 | 1560.8260 | 0.0010 | 0 | 59 | 6.2e-06 | 1 | | | | | | ■ | | | ■ | ■ | ■ | | ■ | R.VSGQTQFNGVNVLAK |
| 1411 | | 538.9448 | 1613.8126 | 1613.8121 | 0.0005 | 1 | 41 | 0.00069 | 1 | | ■ | ■ | ■ | | ■ | ■ | ■ | ■ | ■ | ■ | ■ | ■ | R.INSAKDDAAGQAIANR.F |
| 1412 | | 807.9141 | 1613.8136 | 1613.8121 | 0.0015 | 1 | 106 | 2.1e-10 | 1 | | ■ | ■ | ■ | | ■ | ■ | ■ | ■ | ■ | ■ | ■ | ■ | R.INSAKDDAAGQAIANR.F |
| 1436 | | 546.2711 | 1635.7915 | 1636.8308 | -1.0394 | 0 | 14 | 0.038 | 1 | U | | | | | | | ■ | | | | | | K.IDTGTLGLANFSVDSK.F |
| 1442 | | 823.9059 | 1645.7972 | 1644.9046 | 0.8926 | 1 | 15 | 0.03 | 1 | U | | | | | | | | ■ | | | | | K.TTANTAAKGSDILAALK.T |
| 1451 | | 553.3052 | 1656.8938 | 1657.8523 | -0.9585 | 0 | 11 | 0.084 | 1 | U | | | | | | | | | | ■ | | | K.VDLDAAATDIGTALGQK.V |
| 1465 | | 836.3813 | 1670.7480 | 1670.7457 | 0.0023 | 0 | 73 | 3e-07 | 1 | | | | | | ■ | | | ■ | ■ | ■ | ■ | ■ | R.IQDADYATEVSNMSK.A |
| 1487 | 2 | 843.4580 | 1684.9014 | 1685.8836 | -0.9821 | 0 | 40 | 0.00039 | 1 | | | | | | | | | | ■ | | | ■ | K.IQVGANDGETITIDLK.K |
| 1488 | 3 | 843.4584 | 1684.9022 | 1684.8996 | 0.0027 | 0 | 78 | 6.6e-08 | 1 | | | | | | ■ | | | ■ | | | | | K.IQVGANDGQTITIDLK.K |
| 1500 | 1 | 850.8762 | 1699.7378 | 1699.7359 | 0.0019 | 0 | 136 | 4.1e-14 | 1 | | ■ | ■ | | | | ■ | | | | | | | R.IEDADYATEVSNMSR.A |
| 1501 | | 567.5870 | 1699.7392 | 1699.7359 | 0.0033 | 0 | 95 | 5e-10 | 1 | | ■ | ■ | | | | ■ | | | | | | | R.IEDADYATEVSNMSR.A |
| 1524 | | 572.9164 | 1715.7274 | 1715.7308 | -0.0034 | 0 | 47 | 3.7e-05 | 1 | | ■ | ■ | | | | ■ | | | | | | | R.IEDADYATEVSNMSR.A + Oxidation (M) |
| 1525 | | 858.8719 | 1715.7292 | 1715.7308 | -0.0016 | 0 | 130 | 1.9e-13 | 1 | | ■ | ■ | | | | ■ | | | | | | | R.IEDADYATEVSNMSR.A + Oxidation (M) |
| 1530 | | 860.3578 | 1718.7010 | 1719.8428 | -1.1417 | 0 | 1 | 0.81 | 1 | U | | | | | | | | | | ■ | | | K.VAANSDGEAVGFATVQGK.N |
| 1570 | 1 | 586.3199 | 1755.9379 | 1755.9367 | 0.0012 | 0 | 39 | 0.00028 | 1 | | ■ | ■ | ■ | ■ | | | | | | | | | K.IQVGANDGETITINLAK.I |
| 1576 | 10 | 878.9769 | 1755.9392 | 1755.9367 | 0.0026 | 0 | 82 | 1.4e-08 | 1 | | ■ | ■ | ■ | ■ | | | | | | | | | K.IQVGANDGETITINLAK.I |
| 1588 | | 887.4130 | 1772.8114 | 1772.8105 | 0.0010 | 0 | 37 | 0.0003 | 1 | U | | | | | ■ | | | | | | | | K.AVYVEADGDFTTDAATK.A |
| 1597 | | 596.3041 | 1785.8905 | 1786.7679 | -0.8775 | 1 | 1 | 0.87 | 1 | U | | | ■ | | | | | | | | | | R.SRIEDADYATEVSNMS.- |
| 1614 | | 902.4175 | 1802.8204 | 1802.8170 | 0.0034 | 0 | 56 | 1.1e-05 | 1 | U | | | | | ■ | | | | | | | | K.ASYTNTDGTLTTDNTTK.L |
| 1618 | | 601.9706 | 1802.8900 | 1803.9438 | -1.0539 | 1 | 1 | 3.6 | 1 | | | | | | ■ | | | ■ | ■ | | | | K.NQSALSSSIERLSSGLR.I |
| 1622 | | 904.9610 | 1807.9074 | 1807.9064 | 0.0010 | 0 | 102 | 8e-11 | 1 | U | | | | | ■ | | | | | | | | K.LTGFNVNGSGSVANTAATK.A |
| 1649 | 4 | 927.9585 | 1853.9024 | 1853.9007 | 0.0017 | 0 | 122 | 1e-12 | 1 | | ■ | ■ | | ■ | | | | | | | | | K.TGSTLVVNGATYDVSADGK.T |
| 1649 | 3 | 927.9585 | 1853.9024 | 1852.9167 | 0.9858 | 0 | 57 | 3.6e-06 | 2 | U | | | ■ | | | | | | | | | | K.TGSTLVVNGATYNVSADGK.T |
| 1651 | | 618.9752 | 1853.9038 | 1853.9007 | 0.0031 | 0 | 50 | 1.7e-05 | 1 | | ■ | ■ | | ■ | | | | | | | | | K.TGSTLVVNGATYDVSADGK.T |
| 1651 | | 618.9752 | 1853.9038 | 1852.9167 | 0.9871 | 0 | 40 | 0.00016 | 2 | U | | | ■ | | | | | | | | | | K.TGSTLVVNGATYNVSADGK.T |
| 1655 | | 619.6522 | 1855.9348 | 1855.9349 | -0.0002 | 1 | 30 | 0.0012 | 1 | | ■ | ■ | | ■ | | | | | | | | | K.TITETASGNNKVMYLSK.S |
| 1656 | | 928.9749 | 1855.9352 | 1855.9349 | 0.0003 | 1 | 113 | 5.8e-12 | 1 | | ■ | ■ | | ■ | | | | | | | | | K.TITETASGNNKVMYLSK.S |
| 1666 | | 624.9840 | 1871.9302 | 1871.9299 | 0.0003 | 1 | 19 | 0.013 | 1 | | ■ | ■ | | ■ | | | | | | | | | K.TITETASGNNKVMYLSK.S + Oxidation (M) |
| 1707 | | 644.0217 | 1929.0433 | 1929.0418 | 0.0014 | 1 | 32 | 0.0015 | 1 | | ■ | ■ | ■ | ■ | | | | | | | | | K.SLQSTTNPLETIDKALAK.V |
| 1708 | | 965.5296 | 1929.0446 | 1929.0418 | 0.0028 | 1 | 84 | 1e-08 | 1 | | ■ | ■ | ■ | ■ | | | | | | | | | K.SLQSTTNPLETIDKALAK.V |
| 1716 | | 972.4428 | 1942.8710 | 1942.8690 | 0.0020 | 1 | 75 | 5.3e-08 | 1 | | ■ | ■ | | | | ■ | | | | | | | R.SRIEDADYATEVSNMSR.A |
| 1718 | 1 | 648.6313 | 1942.8721 | 1942.8690 | 0.0030 | 1 | 56 | 3.7e-06 | 1 | | ■ | ■ | | | | ■ | | | | | | | R.SRIEDADYATEVSNMSR.A |

| Query | Dupes | Observed | Mr(expt) | Mr(calc) | Delta | M | Score | Expect | Rank | U | 1 | 2 | 3 | 4 | 5 | 6 | 7 | 8 | 9 | 10 | 11 | 12 | Peptide |
|---|---|---|---|---|---|---|---|---|---|---|---|---|---|---|---|---|---|---|---|---|---|---|---|
| 1722 | | 980.4399 | 1958.8652 | 1958.8640 | 0.0013 | 1 | 62 | 1.5e-06 | ▶1 | | ■ | ■ | | | | ■ | | | | | | | R.SRIEDADYATEVSNMSR.A + Oxidation (M) |
| 1722 | | 980.4399 | 1958.8652 | 1958.8640 | 0.0013 | 1 | 1 | 2 | ▶2 | U | | | | | | | ■ | | | | | | R.SRIEDSDYATEVSNMSR.A |
| 1723 | | 653.9625 | 1958.8657 | 1958.8640 | 0.0017 | 1 | 69 | 3.1e-07 | ▶1 | | ■ | ■ | | | | ■ | | | | | | | R.SRIEDADYATEVSNMSR.A + Oxidation (M) |
| 1747 | ▶1 | 992.0062 | 1981.9978 | 1981.9957 | 0.0022 | 1 | 142 | 1.4e-14 | ▶1 | | ■ | ■ | | ■ | | | | | | | | | K.KTGSTLVVNGATYDVSADGK.T |
| 1747 | | 992.0062 | 1981.9978 | 1981.0116 | 0.9862 | 1 | 24 | 0.0088 | ▶2 | U | | | ■ | | | | | | | | | | K.KTGSTLVVNGATYNVSADGK.T |
| 1749 | ▶3 | 661.6734 | 1981.9984 | 1981.9957 | 0.0027 | 1 | 55 | 6.1e-06 | ▶1 | | ■ | ■ | | ■ | | | | | | | | | K.KTGSTLVVNGATYDVSADGK.T |
| 1749 | ▶2 | 661.6734 | 1981.9984 | 1981.0116 | 0.9867 | 1 | 27 | 0.0038 | ▶2 | U | | | ■ | | | | | | | | | | K.KTGSTLVVNGATYNVSADGK.T |
| 1755 | | 664.6722 | 1990.9948 | 1990.9960 | -0.0012 | 1 | 50 | 1.5e-05 | ▶1 | | ■ | ■ | ■ | ■ | | | | | | | | | R.LEEIDRVSEQTQFNGVK.V |
| 1756 | | 996.5057 | 1990.9968 | 1990.9960 | 0.0009 | 1 | 85 | 5.9e-09 | ▶1 | | ■ | ■ | ■ | ■ | | | | | | | | | R.LEEIDRVSEQTQFNGVK.V |
| 1760 | ▶4 | 997.5020 | 1992.9894 | 1992.9865 | 0.0030 | 0 | 133 | 1.2e-13 | ▶1 | | ■ | ■ | ■ | | | ■ | ■ | | | | | | R.FDSAITNLGNTVNNLSSAR.S |
| 1761 | ▶1 | 665.3375 | 1992.9907 | 1992.9865 | 0.0042 | 0 | 74 | 1e-07 | ▶1 | | ■ | ■ | ■ | | | ■ | ■ | | | | | | R.FDSAITNLGNTVNNLSSAR.S |
| 1796 | ▶6 | 1021.0310 | 2040.0474 | 2040.0415 | 0.0059 | 0 | 84 | 4.4e-09 | ▶1 | U | ■ | | | | | | | | | | | | K.IVYEGIEFTNTGTVAIDAK.G |
| 1813 | ▶2 | 1043.0700 | 2084.1254 | 2084.1225 | 0.0029 | 0 | 127 | 1.3e-12 | ▶1 | | ■ | ■ | | | ■ | | ■ | ■ | ■ | | | | M.AQVINTNSLSLiTQNNiNK.N |
| 1813 | ▶2 | 1043.0700 | 2084.1254 | 2085.1066 | -0.9811 | 0 | 84 | 2.6e-08 | ▶4 | U | | | | | | | | | | ■ | | | M.AQVINTNSLSLITQNNIDK.N |
| 1814 | | 695.7164 | 2084.1274 | 2085.0814 | -0.9540 | 0 | 76 | 1.7e-07 | ▶1 | U | | | | | | | | | | | ■ | | M.AQVINTNSLSLNTQNNINK.N |
| 1814 | | 695.7164 | 2084.1274 | 2084.1225 | 0.0048 | 0 | 76 | 1.8e-07 | ▶2 | | ■ | ■ | | | ■ | | ■ | ■ | ■ | | | | M.AQVINTNSLSLiTQNNiNK.N |
| 1814 | | 695.7164 | 2084.1274 | 2085.1066 | -0.9792 | 0 | 70 | 6.7e-07 | ▶5 | U | | | | | | | | | | ■ | | | M.AQVINTNSLSLITQNNIDK.N |
| 1815 | ▶2 | 1043.0710 | 2084.1274 | 2085.0814 | -0.9540 | 0 | 82 | 4.6e-08 | ▶4 | U | | | | | | | | | | | ■ | | M.AQVINTNSLSLNTQNNINK.N |
| 1817 | | 1047.0540 | 2092.0934 | 2092.0900 | 0.0035 | 0 | 114 | 4e-12 | ▶1 | U | | | | | ■ | | | | | | | | K.TTAADVLSSLADGTTITATGVK.N |
| 1819 | | 1049.6050 | 2097.1954 | 2096.9763 | 0.2191 | 1 | 2 | 0.6 | ▶1 | U | | | | | | | ■ | | | | | | K.KDNGELGYVVENADGTFNR.A |
| 1834 | | 1062.0430 | 2122.0714 | 2123.0382 | -0.9668 | 1 | 1 | 0.86 | ▶1 | U | | | | | | | | | ■ | | | | K.TENTLLTTDAAFDKLGNGDK.V |
| 1842 | ▶6 | 1068.0120 | 2134.0094 | 2134.0066 | 0.0028 | 0 | 135 | 3.5e-14 | ▶1 | U | ■ | | | | | | | | | | | | K.QVYVSTADGSLTTSSDTQFK.I |
| 1843 | | 712.3444 | 2134.0114 | 2134.0066 | 0.0047 | 0 | 35 | 0.00035 | ▶1 | U | ■ | | | | | | | | | | | | K.QVYVSTADGSLTTSSDTQFK.I |
| 1889 | | 1125.0560 | 2248.0974 | 2248.0931 | 0.0043 | 0 | 75 | 2e-07 | ▶1 | | | | | | ■ | | | ■ | ■ | ■ | ■ | ■ | R.LDSAVTNLNNTTTNLSEAQSR.I |
| 1920 | | 774.4044 | 2320.1914 | 2320.1911 | 0.0003 | 1 | 40 | 0.00017 | ▶1 | | ■ | ■ | ■ | ■ | | | | | | | | | K.TLGLDGFNIDGAQKATGSDLISK.F |
| 1924 | | 785.0114 | 2352.0124 | 2353.1471 | -1.1348 | 0 | 1 | 0.89 | ▶1 | U | | | | | | | | | | | | ■ | K.DSLLSMLAPNAGDSFTASVSIGGK.A + Oxida |
| 1929 | | 1199.1440 | 2396.2734 | 2396.2659 | 0.0075 | 0 | 93 | 4.7e-10 | ▶1 | U | | | | | | ■ | | | | | | | R.AQILQQAGTSVLAQANQTTQNVL.- |
| 1940 | ▶1 | 1228.0950 | 2454.1754 | 2454.1762 | -0.0008 | 0 | 74 | 3.9e-08 | ▶1 | U | | | | | ■ | | | | | | | | K.AGDTATFSVEIGGTTQDVVLSSDGK.L |
| 1956 | | 1297.1280 | 2592.2414 | 2592.2402 | 0.0012 | 0 | 117 | 1.9e-12 | ▶1 | U | | | | | ■ | | | | | | | | R.ELTVQATTGTNSQSDLDSIQDEIK.S |
| 1957 | | 865.0884 | 2592.2434 | 2592.2402 | 0.0031 | 0 | 60 | 1e-06 | ▶1 | U | | | | | ■ | | | | | | | | R.ELTVQATTGTNSQSDLDSIQDEIK.S |
| 1958 | | 1298.1090 | 2594.2034 | 2594.2024 | 0.0010 | 0 | 78 | 1.6e-08 | ▶1 | U | | | | | ■ | | | | | | | | K.DNNTFTYDTTATTAELQSYLTPK.A |
| 1972 | ▶1 | 1328.6530 | 2655.2914 | 2655.2848 | 0.0066 | 0 | 105 | 8e-11 | ▶1 | | ■ | | ■ | | | | | | | | | | R.NANDGISVAQTTEGALNEINNNLQR.I |
| 1972 | ▶1 | 1328.6530 | 2655.2914 | 2656.2437 | -0.9522 | 0 | 94 | 9.5e-10 | ▶2 | U | | ■ | | | | | | | | | | | R.NANDGNSVAQTTEGALNEINNNLQR.V |
| 1973 | ▶1 | 886.1050 | 2655.2932 | 2655.2848 | 0.0084 | 0 | 78 | 3.9e-08 | ▶1 | | ■ | | ■ | | | | | | | | | | R.NANDGISVAQTTEGALNEINNNLQR.I |
| 1973 | ▶1 | 886.1050 | 2655.2932 | 2656.1225 | -0.9505 | 0 | 65 | 7.4e-07 | ▶2 | U | | ■ | | | | | | | | | | | R.NANDGNSVAQTTEGALNEINNNLQR.V |
| 1982 | ▶7 | 1338.6670 | 2675.3194 | 2675.3178 | 0.0017 | 0 | 63 | 5e-07 | ▶1 | U | ■ | | | | | | | | | | | | K.AVEFTISGSTDTSGTSATVAPTTALYK.N |
| 1986 | ▶1 | 892.7811 | 2675.3215 | 2675.3178 | 0.0037 | 0 | 30 | 0.00096 | ▶1 | U | ■ | | | | | | | | | | | | K.AVEFTISGSTDTSGTSATVAPTTALYK.N |
| 2003 | ▶1 | 902.4715 | 2704.3927 | 2704.3919 | 0.0008 | 0 | 40 | 9.5e-05 | ▶1 | U | | | | | ■ | | | | | | | | K.ADLAAAAIGTPGAADSTGAIAYTVSAGLTK.T |
| 2004 | | 1353.2050 | 2704.3954 | 2704.3919 | 0.0035 | 0 | 61 | 8.6e-07 | ▶1 | U | | | | | ■ | | | | | | | | K.ADLAAAAIGTPGAADSTGAIAYTVSAGLTK.T |
| 2015 | ▶4 | 1382.6640 | 2763.3134 | 2763.3159 | -0.0024 | 0 | 58 | 2.2e-06 | ▶1 | | ■ | | ■ | | | | | | | | | | R.ELSVQATNGTNSDSDLSSIQAEITQR.L |
| 2015 | | 1382.6640 | 2763.3134 | 2763.3662 | -0.0527 | 0 | 3 | 0.66 | ▶2 | U | | | | | | | | | ■ | | | | K.ITTETTSAGSATTNPLAALDDAISSIDK.F |
| 2018 | ▶6 | 922.1135 | 2763.3187 | 2763.3159 | 0.0028 | 0 | 38 | 0.0002 | ▶1 | | ■ | | ■ | | | | | | | | | | R.ELSVQATNGTNSDSDLSSIQAEITQR.L |
| 2032 | | 956.1967 | 2865.5683 | 2865.5672 | 0.0011 | 0 | 84 | 3.7e-09 | ▶1 | | ■ | | | | | | ■ | | | | | | R.AQILQQAGTSVLAQANQTTQNVLSLLR.- |
| 2033 | | 1433.7950 | 2865.5754 | 2865.5672 | 0.0082 | 0 | 130 | 9.2e-14 | ▶1 | | ■ | | | | | | ■ | | | | | | R.AQILQQAGTSVLAQANQTTQNVLSLLR.- |
| 2036 | ▶1 | 963.4462 | 2887.3168 | 2887.3108 | 0.0060 | 0 | 50 | 1e-05 | ▶1 | U | ■ | | | | | | | | | | | | K.ATGTDNYQINGTDNYTVNVDSGVVQDK.D |
| 2039 | | 963.5245 | 2887.5517 | 2888.6083 | -1.0567 | 1 | 18 | 0.014 | ▶1 | U | | | | | | | | | ■ | | | | K.AQIIQQAGSSVLAKANQVPQQVLSLLQG.- |
| 2048 | | 978.8281 | 2933.4625 | 2933.4591 | 0.0034 | 1 | 59 | 2.2e-06 | ▶1 | | ■ | ■ | ■ | | | | | | | | | | R.SDLGAVQNRFDSAITNLGNTVNNLSSAR.S |
| 2050 | | 991.1563 | 2970.4471 | 2970.4418 | 0.0053 | 1 | 27 | 0.002 | ▶1 | | ■ | ■ | | ■ | | | | | | | | | K.TGSTLVVNGATYDVSADGKTITETASGNNK.V |
| 2058 | | 1011.8440 | 3032.5102 | 3032.5010 | 0.0092 | 1 | 50 | 1.9e-05 | ▶1 | | ■ | | ■ | | | | | | | | | | R.IRELSVQATNGTNSDSDLSSIQAEITQR.L |
| 2058 | | 1011.8440 | 3032.5102 | 3032.5010 | 0.0091 | 1 | 48 | 2.6e-05 | ▶2 | | | ■ | | ■ | | | | | | | | | R.VRELTVQATNGTNSDSDLSSIQAEITQR.L |
| 2072 | ▶2 | 1063.4940 | 3187.4602 | 3187.4542 | 0.0060 | 1 | 125 | 3e-13 | ▶1 | U | ■ | | | | | | | | | | | | K.ATGTDNYQINGTDNYTVNVDSGVVQDKDGK.Q |
| 2077 | | 1077.5750 | 3229.7032 | 3229.6902 | 0.0130 | 1 | 97 | 2.7e-10 | ▶1 | | ■ | ■ | | | | | ■ | | | | | | M.AQVINTNSLSLLTQNNLNKSQSSLSSAIER.L |
| 2092 | | 1097.5710 | 3289.6912 | 3289.6790 | 0.0122 | 0 | 41 | 8e-05 | ▶1 | U | | | | | ■ | | | | | | | | K.LYIDTTGNLTQNGGNNGVGTLAEATLSGLALNK. |
| 2108 | | 837.4263 | 3345.6761 | 3344.6736 | 1.0025 | 1 | 2 | 0.57 | ▶1 | U | | | | | ■ | | | | | | | | K.TTAADVLSSLADGTTITATGVKNGFAAGATSNAY |

▶ 79 subsets and intersections (158 subset proteins in total)

| ▶2 | gi\|112820172 | 71 | H21 0\|EHEC serogroup: O113:H21\|0 |
|---|---|---|---|

10 per page 1

Not what you expected? Try the select summary.

Mascot: http://www.matrixscience.com/
