# Supplementary material for: MS-H: A Novel Proteomic Approach to Isolate and Type the E. coli H Antigen Using Membrane Filtration and Liquid Chromatography-Tandem Mass Spectrometry (LC-MS/MS)
Source: PLoS One. 2013 Feb 21;8(2):e57339. doi: 10.1371/journal.pone.0057339 (PMC3578835; doi:10.1371/journal.pone.0057339)
Supplement: Representative Peptide Data S1 — Peptide data are represented as the Mascot search results from all 53 serotypes, obtained under the Orbitrap platform in Table 4 with related E. coli reference strains. “U” denotes a unique peptide specific for each of the proteins 1.1, 1.2, and beyond. The number 1.1 (shown as 1 in the peptide list and phylogenetic tree) represents the protein which obtained the highest score and confidence value after a Mascot search. This protein, known as the first hit, was used to designate the MS-H type of the unknown flagellin. Related peptides 1.2 (2), 1.3 (3), etc. represented the second, third, etc. hits for MS-H typing analysis. (DOCX) [file pone.0057339.s009.docx › H23-E191.pdf]

MATRIX SCIENCE MASCOT Search Results

User :
E-mail :
Search title : Submitted from 20110810-0587 by Mascot Daemon on VARIABLE
MS data file : C:\Documents and Settings\keding\Desktop\Raw data\20110811-001-0031-00587\20110811-003-EC191MS1.RAW
Database : Flagellin_v2 (192 sequences; 89,845 residues)
Taxonomy : Bacteria (Eubacteria) (192 sequences)
Timestamp : 12 Aug 2011 at 15:51:32 GMT

Not what you expected? Try **the select summary**.

▶ **Search parameters**

▶ **Score distribution**

▶ **Legend**

Protein Family Summary

Significance threshold p< 0.05 Max. number of families AUTO
Ions score or expect cut-off 0 Dendrograms cut at 0

Protein families 1–2 (out of 2)

10 per page 1

▼**1**

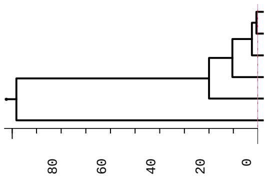

1	**gi|6009841**	1645	H23 0|0|K42 [gi|30059882|ref|AAP13313| H23 0|H23|HW23]
3	**gi|6009849**	634	H49 0|H49|U11a-44
4	**gi|6941882**	608	H14 0|O138:H14|O138:H14
6	**gi|33590243**	202	H16 NM|O157:NM(H16)|PC02
5	**gi|40994711**	541	H4 0|O154:H4|0
2	**gi|192955539**	657	H9 0|O111:H9|E110019 [gi|193071144|ref|ZP_03052068| H9 0|O111:H9|E110019]

Threshold (**0**): 0

		Score	Mass	Matches	Sequences	emPAI
☑ **1.1**	**gi|6009841**	1645	60422	40 (31)	33 (29)	4.18
	H23 0|0|K42 [gi|30059882|ref|AAP13313| H23 0|H23|HW23]					
☑ **1.2**	**gi|192955539**	657	68106	21 (10)	16 (9)	0.60
	H9 0|O111:H9|E110019 [gi|193071144|ref|ZP_03052068| H9 0|O111:H9|E110019]					
	▶ **2 samesets of gi|192955539**					
☑ **1.3**	**gi|6009849**	634	58493	26 (12)	18 (11)	0.93
	H49 0|H49|U11a-44					
☑ **1.4**	**gi|6941882**	608	56492	27 (11)	21 (10)	0.86
	H14 0|O138:H14|O138:H14					
☑ **1.5**	**gi|40994711**	541	36224	19 (10)	14 (8)	1.40
	H4 0|O154:H4|0					
☑ **1.6**	**gi|33590243**	202	55093	15 (4)	12 (3)	0.26
	H16 NM|O157:NM(H16)|PC02					
	▶ **2 samesets of gi|33590243**					

▼**72 peptide matches (63 non-duplicate, 9 duplicate)**

Query	Dupes	Observed	Mr(expt)	Mr(calc)	Delta	M	Score	Expect	Rank	U	1	2	3	4	5	6	Peptide
21		316.1742	630.3338	631.3653	-1.0315	0	2	4.8	▶1		■	■	■	■	■	■	R.LSSGLR.I
81		355.1978	708.3810	708.3806	0.0004	0	6	0.7	▶1		■	■	■	■	■		R.FTSNIK.G
95		366.6981	731.3816	731.3813	0.0003	0	4	1.3	▶1	U						■	R.LSEIDR.V
113	▶1	**380.2035**	**758.3924**	**758.4174**	**-0.0249**	**0**	**30**	**0.0054**	▶1	**U**						■	**K.LDEALAK.V**
115	▶3	**380.6952**	**759.3758**	**759.3763**	**-0.0004**	**0**	**26**	**0.014**	▶1		■	■	■	■	■		**R.LDEIDR.V**
128		387.2024	772.3902	771.4490	0.9412	0	1	1.9	▶1	U					■		K.ALDAAIAK.V
242	▶2	**423.2216**	**844.4286**	**844.4402**	**-0.0116**	**0**	**14**	**0.042**	▶1	**U**			■				**K.AAAGAESIR.Y**
247	▶1	**424.2553**	**846.4960**	**846.4447**	**0.0514**	**0**	**15**	**0.034**	▶1	**U**				■			**K.AATTADGLK.A**
267		**430.7397**	**859.4648**	**859.4651**	**-0.0003**	**0**	**40**	**0.00011**	▶1	**U**	■						**K.DVVLADTK.A**
277		**434.6879**	**867.3612**	**867.3610**	**0.0002**	**0**	**37**	**0.00021**	▶1	**U**	■						**K.FDTDADGK.I**
307		444.7376	887.4606	888.4916	-1.0310	0	1	1.4	▶1	U				■			K.AATTADVLK.A
356		**460.2456**	**918.4766**	**918.4771**	**-0.0004**	**0**	**59**	**1.3e-06**	▶1	**U**	■						**K.AATVSDVTR.A**
356		460.2456	918.4766	918.4658	0.0108	0	8	0.16	▶2	U				■			K.AATTADELK.A
359		460.3325	918.6504	918.4407	0.2098	0	1	0.81	▶1	U			■				K.ADSTANNVK.I
377		466.7432	931.4718	930.4883	0.9836	0	3	2.3	▶1			■		■	■	■	R.SSLGAVQNR
400	▶1	**473.2531**	**944.4916**	**944.5039**	**-0.0123**	**0**	**45**	**8.9e-05**	▶1		■		■				**R.SSLGAIQNR.L**
517		502.2401	1002.4656	1002.5094	-0.0438	1	11	0.42	▶1		■	■	■	■	■		K.SRLDEIDR.V
546		508.7745	1015.5344	1014.5709	0.9635	0	2	0.59	▶1	U						■	K.ALATTNPLSK.L
649	▶1	539.2702	1076.5258	1077.4873	-0.9614	0	10	0.13	▶1	U						■	K.NDGSAQAIMR.E + Oxidation (M)
720		**551.2681**	**1100.5216**	**1100.5210**	**0.0006**	**0**	**72**	**5.6e-07**	▶1		■	■	■	■	■	■	**K.DDAAGQAIANR.F**
860		**588.2826**	**1174.5506**	**1174.5506**	**0.0001**	**0**	**42**	**6.3e-05**	▶1	**U**	■						**K.EYTTSYAVNK.D**
882		397.5584	1189.6534	1190.5891	-0.9357	0	1	4	▶1		■	■	■	■			K.NQSALSSSIER.L
884		**596.3025**	**1190.5904**	**1190.5891**	**0.0014**	**0**	**67**	**1.1e-06**	▶1		■	■	■	■			**K.NQSALSSSIER.L**
898		600.8537	1199.6928	1199.6734	0.0194	1	11	0.071	▶1	U						■	K.LRSSLGAVQNR.F
936		407.5508	1219.6306	1220.6150	-0.9844	0	2	0.62	▶1	U						■	R.VSNQTQFNGVK.V

Query	Dupes	Observed	Mr(expt)	Mr(calc)	Delta	M	Score	Expect	Rank	U	1	2	3	4	5	6	Peptide
990		623.8013	1245.5880	1245.5878	0.0003	0	49	1.2e-05	1	U	■						K.GGSLTFGDTTYK.I
1028		634.3229	1266.6312	1266.6303	0.0009	0	63	5e-07	1	U	■						K.LTTDTTSAGTATK.D
1051		639.8387	1277.6628	1277.6616	0.0013	0	69	1.2e-07	1	U	■						R.AGGTLVNGAYDIK.T
1052		640.3351	1278.6556	1278.6568	-0.0011	0	60	1.1e-06	1	U	■						R.DALAASLHAEPGK.T
1053		427.2261	1278.6565	1278.6568	-0.0003	0	29	0.0013	1	U	■						R.DALAASLHAEPGK.T
1102		434.9186	1301.7340	1300.6987	1.0353	1	0	2.2	2	U			■				K.LKDGDSVAVAAQK.Y
1181		679.8491	1357.6836	1357.6838	-0.0001	0	92	6.2e-10	1	U	■						K.LNDGDVVTINNGK.D
1201		688.3230	1374.6314	1374.6304	0.0011	0	96	2.6e-10	1	U	■						K.VSFDAGTSTDTFK.D
1237		468.9054	1403.6944	1403.6932	0.0011	1	29	0.0012	1	U	■						K.TKEYTTSYAVNK.D
1284		720.9125	1439.8104	1439.8096	0.0008	0	106	1e-10	1		■	■	■	■	■		K.AQIIQQAGNSVLAK.A
1301		728.3703	1454.7260	1454.7253	0.0008	0	92	1e-09	1	U	■						K.TTNTALTTTDAFAK.L
1329		740.3578	1478.7010	1478.7002	0.0009	0	58	1.6e-06	1	U	■						K.TVNGSWTTNDGTVK.F
1344		747.9198	1493.8250	1493.8202	0.0049	0	56	1.4e-05	1		■	■	■	■	■		K.ANQVPQQVLSLLQG.-
1352		750.4046	1498.7946	1498.7879	0.0068	0	72	6e-08	1	U	■						K.DPLAALDAAISSIDK.F
1423		781.4203	1560.8260	1560.8260	0.0000	0	66	1.1e-06	1		■		■	■			R.VSGQTQFNGVNVLAK.D
1495		545.3018	1632.8836	1632.8835	0.0001	0	33	0.00045	1	U	■						K.YKPTIGATVNLNSAGK.L
1528		836.3810	1670.7474	1670.7457	0.0017	0	136	1.5e-13	1		■	■	■	■	■		R.IQDADYATEVSNMSK.A
1540		560.0858	1677.2356	1676.8370	0.3986	0	3	0.52	1	U		■					K.IDSDTLNLAGFNVNGK.G
1587		860.3572	1718.6998	1718.7974	-0.0975	0	2	0.67	1	U						■	K.ALAYNDAPMSVYFGGK.N + Oxidation (M)
1621		581.6324	1741.8754	1740.8530	1.0224	0	3	0.56	1	U					■		K.QVNLLSYTDTASNSTK.Y
1628		584.9463	1751.8171	1751.8611	-0.0440	1	5	0.34	1	U				■			K.LTTDAETKAATTADCLK.A
1681		359.9973	1794.9501	1795.8873	-0.9372	1	2	0.67	1	U				■			K.LTTDAETKAATTADMLK.A + Oxidation (M)
1689		902.4505	1802.8864	1803.9438	-1.0574	1	2	2.9	1		■	■	■	■			K.NQSALSSSIERLSSGLR.I
1760		630.4536	1888.3390	1887.1049	1.2341	1	5	0.34	1	U			■				K.LTLMLMQAVISLLAAKR.L + Oxidation (M)
1793		646.6531	1936.9375	1935.9538	0.9837	1	46	2.8e-05	1	U	■						K.LNDGDVVTINNGKDTAYK.Y
1851		1031.9830	2061.9514	2063.0456	-1.0941	1	5	0.29	1	U				■			K.AATTADMLKALDEAISSIDK.F
1860		1043.0690	2084.1234	2084.1225	0.0009	0	100	7.2e-10	1		■	■	■	■	■	■	M.AQVINTNSLSLiTQNNiNK.N
1861		695.7155	2084.1247	2084.1225	0.0021	0	63	3.4e-06	1		■	■	■	■	■	■	M.AQVINTNSLSLiTQNNiNK.N
1885		716.3427	2146.0063	2146.0066	-0.0004	1	57	2e-06	1	U	■						K.VSFDAGTSTDTFKDADGAITK.T
1935		750.3718	2248.0936	2248.0931	0.0005	0	95	1.9e-09	1		■	■	■	■	■		R.LDSAVTNLNNTTTNLSEAQSR.I
1975		787.7433	2360.2081	2359.0240	1.1841	1	1	0.74	1	U		■					K.NGYTYDAASKSYSFAADGADSAK.T
2002		1241.0860	2480.1574	2480.1555	0.0020	0	144	3.9e-15	1	U	■						K.IGQGTAGVDPDDASDDVLGTISYSK.S
2004		1245.6500	2489.2854	2489.2762	0.0093	0	111	7.4e-12	1	U		■					K.ASDLLANITDGSVITGGGANAFGVAAK.N
2034		1322.1570	2642.2994	2642.2896	0.0099	0	113	8.8e-12	1	U	■						R.NANDGISLAQTTEGALSEINNNLQR.V
2041		893.4689	2677.3849	2677.3043	0.0806	1	0	0.91	1	U				■			K.GTITIDGSAQDVQISSDGKITASNGDK.L
2063		933.5052	2797.4938	2797.4821	0.0116	0	33	0.00051	1	U					■		K.IQIGANDNQTISIGLQQIDSTTLNLK.G
2064		1399.7560	2797.4974	2797.4821	0.0153	0	24	0.0041	1	U					■		K.IQIGANDNQTISIGLQQIDSTTLNLK.G
2141		844.9301	3375.6913	3376.7256	-1.0343	1	3	0.88	1	U						■	-.MAQVINTNSLSLLTQNNLNKSQSSLSSAIER.L + Oxidation (

47 subsets and intersections (161 subset proteins in total)

---

2 **gi|112820172** 17 H21 0|EHEC serogroup: O113:H21|0

10 per page 1

Not what you expected? Try **the select summary**.

Mascot: http://www.matrixscience.com/
