# Supplementary material for: MS-H: A Novel Proteomic Approach to Isolate and Type the E. coli H Antigen Using Membrane Filtration and Liquid Chromatography-Tandem Mass Spectrometry (LC-MS/MS)
Source: PLoS One. 2013 Feb 21;8(2):e57339. doi: 10.1371/journal.pone.0057339 (PMC3578835; doi:10.1371/journal.pone.0057339)
Supplement: Representative Peptide Data S1 — Peptide data are represented as the Mascot search results from all 53 serotypes, obtained under the Orbitrap platform in Table 4 with related E. coli reference strains. “U” denotes a unique peptide specific for each of the proteins 1.1, 1.2, and beyond. The number 1.1 (shown as 1 in the peptide list and phylogenetic tree) represents the protein which obtained the highest score and confidence value after a Mascot search. This protein, known as the first hit, was used to designate the MS-H type of the unknown flagellin. Related peptides 1.2 (2), 1.3 (3), etc. represented the second, third, etc. hits for MS-H typing analysis. (DOCX) [file pone.0057339.s009.docx › H24-E192.pdf]

MATRIX SCIENCE MASCOT Search Results

User :
E-mail :
Search title : Submitted from 20110810-0587 by Mascot Daemon on VARIABLE
MS data file : C:\Documents and Settings\keding\Desktop\Raw data\20110811-001-0031-00587\20110811-004-EC192MS1.RAW
Database : Flagellin_v2 (192 sequences; 89,845 residues)
Taxonomy : Bacteria (Eubacteria) (192 sequences)
Timestamp : 12 Aug 2011 at 15:53:44 GMT

Not what you expected? Try **the select summary**.

Search parameters

Score distribution

Legend

Protein Family Summary

Significance threshold p< 0.05 Max. number of families AUTO
Ions score or expect cut-off 0 Dendrograms cut at 0

Protein family 1 (out of 1)

10 per page 1

1

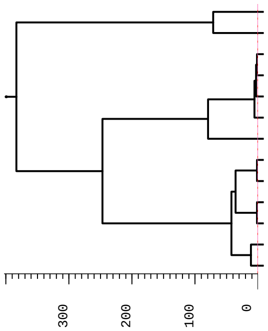

1	gi|30059884	1752	H24 0|H24|HW25
11	gi|224579297	303	H17 0|O15:K-:H17|P12b
2	gi|6009841	1052	H23 0|0|K42 [gi|30059882|ref|AAP13313| H23 0|H23|HW23]
5	gi|6009849	787	H49 0|H49|U11a-44
9	gi|4731618	512	Hxx(H7 98.7%) 0|0|DEC 3f
4	gi|12516024	791	H7 0|O157:H7|EDL933 [gi|15802358|ref|NP_288384| H7 0|O157:H7|EDL933]
8	gi|121144335	640	H55 0|O75:H55|E2987-73
3	gi|192955539	820	H9 0|O111:H9|E110019 [gi|193071144|ref|ZP_03052068| H9 0|O111:H9|E110019]
12	gi|154710444	299	H35 0|H35|0
10	gi|33590243	334	H16 NM|O157:NM(H16)|PC02
13	gi|33590252	287	Hxx(H8 89.5%) 0|0|PC41
6	gi|40994711	754	H4 0|O154:H4|0
7	gi|56383041	744	H4 0|O68:H4|P 7d

Threshold (**0**): 0

		Score	Mass	Matches	Sequences	emPAI
1.1	**gi|30059884**	1752	47256	45 (31)	27 (21)	5.61
	H24 0|H24|HW25					
1.2	**gi|6009841**	1052	60422	35 (22)	20 (17)	2.05
	H23 0|0|K42 [gi|30059882|ref|AAP13313| H23 0|H23|HW23]					
1.3	**gi|192955539**	820	68106	28 (16)	16 (13)	1.12
	H9 0|O111:H9|E110019 [gi|193071144|ref|ZP_03052068| H9 0|O111:H9|E110019]					
	**2 samesets of gi|192955539**					
1.4	**gi|12516024**	791	59916	36 (17)	21 (13)	1.35
	H7 0|O157:H7|EDL933 [gi|15802358|ref|NP_288384| H7 0|O157:H7|EDL933]					
1.5	**gi|6009849**	787	58493	31 (17)	17 (13)	1.40
	H49 0|H49|U11a-44					
1.6	**gi|40994711**	754	36224	25 (16)	14 (12)	3.06
	H4 0|O154:H4|0					
1.7	**gi|56383041**	744	36464	25 (16)	14 (12)	3.01
	H4 0|O68:H4|P 7d					
1.8	**gi|121144335**	640	62285	25 (13)	15 (10)	0.85
	H55 0|O75:H55|E2987-73					
1.9	**gi|4731618**	512	56848	20 (11)	11 (8)	0.76
	Hxx(H7 98.7%) 0|0|DEC 3f					
1.10	**gi|33590243**	334	55093	16 (8)	10 (7)	0.59
	H16 NM|O157:NM(H16)|PC02					
	**2 samesets of gi|33590243**					
1.11	**gi|224579297**	303	49533	11 (7)	7 (6)	0.57
	H17 0|O15:K-:H17|P12b					
1.12	**gi|154710444**	299	52714	14 (7)	9 (6)	0.53
	H35 0|H35|0					
1.13	**gi|33590252**	287	52373	32 (7)	8 (6)	0.53
	Hxx(H8 89.5%) 0|0|PC41					

119 peptide matches (85 non-duplicate, 34 duplicate)

Query	Dupes	Observed	Mr(expt)	Mr(calc)	Delta	M	Score	Expect	Rank	U	1	2	3	4	5	6	7	8	9	10	11	12	13	Peptide
15	2	**316.6898**	**631.3650**	**631.3653**	**-0.0003**	**0**	**25**	**0.028**	1		■	■	■	■	■	■	■	■	■	■	■	■	■	**R.LSSGLR**
29		330.2079	658.4012	659.3238	-0.9226	0	6	0.28	1	U	■													K.NNDAVK.T
48		351.7053	701.3960	700.4119	0.9841	1	0	1.4	1	U													■	K.DPTKLK.A
55		355.1976	708.3806	708.3806	0.0000	0	13	0.35	1		■	■	■	■	■	■	■	■	■		■	■		R.FTSNIK.G

Query	Dupes	Observed	Mr(expt)	Mr(calc)	Delta	M	Score	Expect	Rank	U	1	2	3	4	5	6	7	8	9	10	11	12	13	Peptide
69		366.6978	731.3810	731.3813	-0.0003	0	42	0.00023	▶1		■									■	■		■	R.LSEIDR.V
86		380.2036	758.3926	758.4174	-0.0247	0	32	0.0038	▶1	U										■				K.LDEALAK.V
87	▶2	380.6954	759.3762	759.3763	-0.0000	0	29	0.0078	▶1			■	■	■	■	■	■	■	■					R.LDEIDR.V
101		387.7224	773.4302	773.4283	0.0019	0	10	0.26	▶1	U	■													K.AVTDAAVK.L
110	▶2	394.7293	787.4440	787.4440	0.0001	0	55	3.2e-06	▶1	U	■													K.ATGEVALK.D
193	▶20	421.7585	841.5024	841.4658	0.0367	0	14	0.036	▶1	U													■	K.AVTQPQAK.D
210		423.2219	844.4292	844.4402	-0.0110	0	15	0.033	▶1	U					■									K.AAAGAESIR.Y
214		424.7129	847.4112	846.4633	0.9480	1	9	0.13	▶1	U				■										K.ADMKALAK.A
271		446.2288	890.4430	890.4531	-0.0101	1	24	0.014	▶1	U				■										K.ADMKALDK.A
278		448.7213	895.4280	894.4303	0.9978	1	2	0.57	▶1	U				■										K.ADMKALCK.A + Oxidation (M)
289		452.7350	903.4554	903.4848	-0.0293	1	0	1.4	▶1	U				■										K.ADMKALQK.A
293		453.7380	905.4614	904.5052	0.9563	1	2	0.67	▶2	U				■					■					K.ADMKALxK.A + Oxidation (M)
294	▶1	453.8398	905.6650	904.4688	1.1963	1	5	0.32	▶1	U				■										K.ADMKALEK.A
323	▶1	466.2511	930.4876	930.4883	-0.0006	0	54	1.8e-05	▶1		■		■			■	■	■		■		■		R.SSLGAVQNR
347	▶1	473.2595	944.5044	944.5039	0.0005	0	39	0.00035	▶1			■		■	■				■					R.SSLGAIQNR.L
472		508.7744	1015.5342	1014.5709	0.9633	0	1	0.73	▶1	U										■				K.ALATTNPLSK.L
495		514.7305	1027.4464	1027.4458	0.0006	0	51	8.6e-06	▶1	U	■													K.TASYTDADGK.A
538		527.2644	1052.5142	1052.5138	0.0004	0	51	8.1e-06	▶1	U	■													K.GNDTATYAIK.A
557		354.4787	1060.4143	1061.4924	-1.0781	0	3	0.55	▶1	U										■				K.NDGSAQAIMR.E
578		539.2699	1076.5252	1077.4873	-0.9620	0	11	0.12	▶1	U										■				K.NDGSAQAIMR.E + Oxidation (M)
621		546.4001	1090.7856	1089.5601	1.2256	1	4	0.38	▶1	U												■		R.VMAANDIKGR.T + Oxidation (M)
642		551.2681	1100.5216	1100.5210	0.0006	0	54	3.3e-05	▶1		■	■	■	■	■	■	■	■		■	■	■	■	K.DDAAGQAIANR.F
711		570.2411	1138.4676	1138.5479	-0.0803	0	16	0.023	▶1	U									■					K.DHSAGQAIANR.F
815		397.2037	1188.5893	1187.6034	0.9859	0	2	0.7	▶1	U								■						K.ALDDAISQIDK.F
820		596.3023	1190.5900	1190.5891	0.0010	0	34	0.0021	▶1			■	■	■	■									K.NQSALSSSIER.L
853		603.3095	1204.6044	1204.6048	-0.0003	0	39	0.00021	▶1		■					■	■							K.NQSALSTSIER.L
929		623.8008	1245.5870	1245.5878	-0.0007	0	21	0.0086	▶1	U		■												K.GGSLTFGDTTYK.I
939		627.8037	1253.5928	1254.6244	-1.0316	0	2	0.63	▶1	U					■									K.FNALDAATAFSK.L
969		634.3226	1266.6306	1266.6303	0.0003	0	43	5.5e-05	▶1	U		■												K.LTTDTTSAGTATK.D
993		427.2262	1278.6568	1278.6568	-0.0000	0	11	0.085	▶1	U		■												R.DALAASLHAEPGK.T
1110		672.8782	1343.7418	1343.7408	0.0010	0	57	1.8e-06	▶1	U								■						-.SLSLITQNNINK.N
1112		673.8765	1345.7384	1345.7089	0.0295	0	3	0.47	▶1	U					■									K.DTVSSDALLAQVK.A
1128		683.3237	1364.6328	1364.6783	-0.0455	0	1	0.81	▶1	U								■						K.GSVSNTAATTDTLK.L
1138		688.3235	1374.6324	1374.6304	0.0021	0	57	2.1e-06	▶1	U		■												K.VSFDAGTSTDTFK.D
1225		720.9133	1439.8120	1439.8096	0.0024	0	101	3.9e-10	▶1			■	■	■	■	■	■	■	■					K.AQIIQQAGNSVLAK.A
1245		728.9097	1455.8048	1455.8045	0.0003	0	116	4.1e-12	▶1	U	■													K.AQIIQQAGNSVLSK.-
1246		486.2756	1455.8050	1455.8045	0.0005	0	58	2.5e-06	▶1	U	■													K.AQIIQQAGNSVLSK.-
1290		498.9244	1493.7514	1493.8202	-0.0688	0	4	2.3	▶1			■	■	■	■	■	■							K.ANQVPQQVLSLLQG.-
1292		747.9196	1493.8246	1493.8202	0.0045	0	43	0.00031	▶1			■	■	■	■	■	■							K.ANQVPQQVLSLLQG.-
1292		747.9196	1493.8246	1492.7158	1.1089	0	2	3.8	▶5	U												■		K.SIDDALSQVDQFR.S
1358	▶2	781.4200	1560.8254	1560.8260	-0.0006	0	70	4.5e-07	▶1		■	■		■	■			■	■					R.VSGQTQFGVNVLAK
1408		807.9136	1613.8126	1613.8121	0.0005	1	64	3.6e-06	▶1		■	■	■	■	■	■	■	■		■	■	■	■	R.INSAKDDAAGQAIANR.F
1409		538.9449	1613.8129	1613.8121	0.0008	1	21	0.063	▶1		■	■	■	■	■	■	■	■		■	■	■	■	R.INSAKDDAAGQAIANR.F
1451		556.2804	1665.8194	1665.8210	-0.0016	1	39	0.00012	▶1	U	■													K.LQDEKGNDTATYAIK.A
1452		833.9179	1665.8212	1665.8210	0.0003	1	77	2e-08	▶1	U	■													K.LQDEKGNDTATYAIK.A
1457		557.9229	1670.7469	1670.7457	0.0011	0	57	1.2e-05	▶1		■	■	■	■	■	■	■	■	■		■			R.IQDADYATEVSNMSK.A
1458		836.3809	1670.7472	1670.7457	0.0015	0	94	2.5e-09	▶1		■	■	■	■	■	■	■	■	■		■			R.IQDADYATEVSNMSK.A
1486	▶2	852.4267	1702.8388	1702.8374	0.0015	0	109	1.3e-11	▶1	U	■													K.LGGDNGTTEIVVDAASGK.T
1496		855.4025	1708.7904	1708.7904	0.0001	0	134	4.3e-14	▶1	U	■													K.AQDGSLYAANVDEATGK.V
1497		570.6047	1708.7923	1708.7904	0.0019	0	44	3.7e-05	▶1	U	■													K.AQDGSLYAANVDEATGK.V
1557		879.9631	1757.9116	1757.9047	0.0070	0	110	1.1e-11	▶1	U	■													K.TTSPLAALDDAISQIDK.F
1577		595.2953	1782.8641	1782.8636	0.0005	1	8	0.17	▶1	U	■													K.TASYTDADGKAVTDAAVK.L
1595		359.9971	1794.9491	1795.8047	-0.8556	1	0	1	▶1	U													■	K.TMSYTDADGVKHDNVK.V + Oxidation (
1607		601.9706	1802.8900	1803.9438	-1.0539	1	0	4.6	▶1			■	■	■	■									K.NQSALSSSIERLSSGLR.I
1608	▶1	902.9432	1803.8718	1803.9438	-0.0720	1	2	3.3	▶2			■	■	■	■									K.NQSALSSSIERLSSGLR.I
1630		458.9463	1831.7561	1832.8976	-1.1415	1	9	0.15	▶1	U									■					K.STAGTGVNAAAQAADSASKR.D
1641		922.9389	1843.8632	1843.8622	0.0010	0	64	3.6e-07	▶1	U		■												K.ATGNTTTVDFNSGIMTSK.V
1654		621.3059	1860.8959	1860.9152	-0.0193	1	0	0.97	▶1	U										■				K.AVEWAVKNDGSAQAIMR.E + Oxidation
1717		979.4795	1956.9444	1956.9429	0.0016	0	84	3.7e-09	▶1	U											■			K.ATNSYFAIVADGSADNTLK.N
1777		688.0346	2061.0820	2061.0742	0.0078	1	49	1.5e-05	▶1	U	■													K.TTSPLAALDDAISQIDKFR.S
1781		1043.0700	2084.1254	2084.1225	0.0029	0	123	3.7e-12	▶1			■	■	■	■	■	■			■		■	■	M.AQVINTNSLSLiTQNNiNK.N
1782		695.7159	2084.1259	2084.1225	0.0033	0	84	2.6e-08	▶1			■	■	■	■	■	■			■		■	■	M.AQVINTNSLSLiTQNNiNK.N
1808		724.3636	2170.0690	2170.0688	0.0001	0	22	0.0059	▶1	U			■											K.VDTVNVTNAHVSAEGMANLTK.S
1839		750.3724	2248.0954	2248.0931	0.0023	0	86	1.3e-08	▶1		■	■	■	■	■	■	■	■	■					R.LDSAVTNLNNTTTNLSEAQSR.I
1840		1125.0550	2248.0954	2248.0931	0.0023	0	137	1.1e-13	▶1		■	■	■	■	■	■	■	■	■					R.LDSAVTNLNNTTTNLSEAQSR.I
1902		1245.6490	2489.2834	2489.2762	0.0073	0	104	3.8e-11	▶1	U			■											K.ASDLLANITDGSVITGGGANAFGVAAK.N
1911		1276.6130	2551.2114	2551.2137	-0.0023	0	110	1.4e-11	▶1	U	■													R.ELTVQATTGTNSDSDLSSIQDEIK.S
1913		855.3972	2563.1698	2564.2864	-1.1166	1	5	0.28	▶1	U								■						-.SLSLITQNNINKNQSSMSTAIER.L + Oxid
1924		1311.1580	2620.3014	2620.2980	0.0034	0	104	3.9e-11	▶1	U	■													K.TYDAGALQNVDLSSATNTVTAIPNGK.T
1925		874.4418	2620.3036	2620.2980	0.0055	0	44	4.2e-05	▶1	U	■													K.TYDAGALQNVDLSSATNTVTAIPNGK.T
1929		1322.1540	2642.2934	2642.2896	0.0039	0	111	1.2e-11	▶1		■	■												R.NANDGISLAQTTEGALSEINNNLQR.V
1930		881.7734	2642.2984	2642.2896	0.0088	0	55	5.4e-06	▶1		■	■												R.NANDGISLAQTTEGALSEINNNLQR.V
1931		882.4599	2644.3579	2644.2688	0.0890	0	15	0.035	▶1	U												■		R.NANDGISVAQTTEGSLSEINNNLQR.I
1938		533.6647	2663.2871	2664.2919	-1.0047	1	0	1.1	▶1	U				■										K.ALYKAASEGSDGASLTFGNTEYTIAK.A
1968		933.5046	2797.4920	2797.4821	0.0098	0	31	0.00076	▶1	U						■								K.IQIGANDNQTISIGLQQIDSTTLNLK.G
1968		933.5046	2797.4920	2798.4661	-0.9742	0	20	0.0099	▶2	U							■							K.IQIGANDNETISIGLQQIDSTTLNLK.G
1969		1399.7560	2797.4974	2798.4661	-0.9687	0	21	0.0071	▶1	U							■							K.IQIGANDNETISIGLQQIDSTTLNLK.G
1969		1399.7560	2797.4974	2797.4821	0.0153	0	20	0.01	▶2	U						■								K.IQIGANDNQTISIGLQQIDSTTLNLK.G
2009		1054.5060	3160.4962	3160.5708	-0.0747	1	13	0.16	▶2		■		■			■	■	■						R.SSLGAVQNRLDSAVTNLNNTTTNLSEAQSR.I
2075		909.7455	3634.9529	3635.8894	-0.9365	0	5	0.33	▶1	U	■													K.TSAAVNTLGGGAGSVAVDFATTSLTAITGLGS
2077		727.9995	3634.9611	3635.8894	-0.9283	0	1	0.87	▶1	U	■													K.TSAAVNTLGGGAGSVAVDFATTSLTAITGLGS

▶46 subsets and intersections (154 subset proteins in total)

10 per page 1

Not what you expected? Try **the select summary**.

Mascot: http://www.matrixscience.com/
