# Supplementary material for: MS-H: A Novel Proteomic Approach to Isolate and Type the E. coli H Antigen Using Membrane Filtration and Liquid Chromatography-Tandem Mass Spectrometry (LC-MS/MS)
Source: PLoS One. 2013 Feb 21;8(2):e57339. doi: 10.1371/journal.pone.0057339 (PMC3578835; doi:10.1371/journal.pone.0057339)
Supplement: Representative Peptide Data S1 — Peptide data are represented as the Mascot search results from all 53 serotypes, obtained under the Orbitrap platform in Table 4 with related E. coli reference strains. “U” denotes a unique peptide specific for each of the proteins 1.1, 1.2, and beyond. The number 1.1 (shown as 1 in the peptide list and phylogenetic tree) represents the protein which obtained the highest score and confidence value after a Mascot search. This protein, known as the first hit, was used to designate the MS-H type of the unknown flagellin. Related peptides 1.2 (2), 1.3 (3), etc. represented the second, third, etc. hits for MS-H typing analysis. (DOCX) [file pone.0057339.s009.docx › H25-E193.pdf]

(MATRIX SCIENCE) MASCOT Search Results

User :
E-mail :
Search title : Submitted from 20110810-0587 by Mascot Daemon on VARIABLE
MS data file : C:\Documents and Settings\keding\Desktop\Raw data\20110811-001-0031-00587\20110811-005-EC193MS1.RAW
Database : Flagellin_v2 (192 sequences; 89,845 residues)
Taxonomy : Bacteria (Eubacteria) (192 sequences)
Timestamp : 12 Aug 2011 at 15:55:43 GMT

Not what you expected? Try **the select summary**.

▶**Search parameters**

▶**Score distribution**

▶**Legend**

Protein Family Summary

Significance threshold p< 0.05 Max. number of families AUTO
Ions score or expect cut-off 0 Dendrograms cut at 0

Protein families 1–2 (out of 2)

10 per page 1

▼**1**

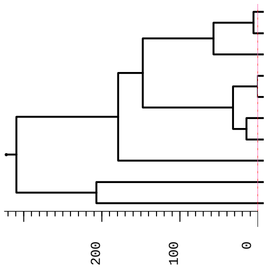

1	gi|323968340	1320	Hxx(H25 99.0%) 0|0|M863 [gi|327253054|ref|EGE64708| Hxx 0|0|STEC_7v]
8	gi|33590243	283	H16 NM|O157:NM(H16)|PC02
5	gi|125659361	470	H55 0|H55|781-55
3	gi|6009841	552	H23 0|0|K42 [gi|30059882|ref|AAP13313| H23 0|H23|HW23]
9	gi|46093552	273	H53 0|0|E480-68
7	gi|30059874	406	H18 0|H18|K 12a [gi|293405409|ref|ZP_06649401| Hxx 0|0|FVEC1412] [gi|300899178|ref|ZP_0...
10	gi|5107837	85	H7 0|O128:H7|DEC 13a
4	gi|192955539	516	H9 0|O111:H9|E110019 [gi|193071144|ref|ZP_03052068| H9 0|O111:H9|E110019]
2	gi|30059886	1301	H25 0|H25|HW26
6	gi|30059884	443	H24 0|H24|HW25

Threshold (**0**): 0

		Score	Mass	Matches	Sequences	emPAI
☑ 1.1	gi|323968340	1320	46353	33 (26)	24 (19)	4.57
	Hxx(H25 99.0%) 0|0|M863 [gi|327253054|ref|EGE64708| Hxx 0|0|STEC_7v]					
☑ 1.2	gi|30059886	1301	41679	31 (24)	22 (17)	4.37
	H25 0|H25|HW26					
☑ 1.3	gi|6009841	552	60422	20 (11)	17 (9)	0.79
	H23 0|0|K42 [gi|30059882|ref|AAP13313| H23 0|H23|HW23]					
☑ 1.4	gi|192955539	516	68106	16 (10)	14 (9)	0.60
	H9 0|O111:H9|E110019 [gi|193071144|ref|ZP_03052068| H9 0|O111:H9|E110019]					
	▶**2 samesets of gi|192955539**					
☑ 1.5	gi|125659361	470	36284	17 (11)	14 (9)	1.61
	H55 0|H55|781-55					
	▶**1 sameset of gi|125659361**					
☑ 1.6	gi|30059884	443	47256	12 (7)	11 (6)	0.60
	H24 0|H24|HW25					
☑ 1.7	gi|30059874	406	57210	17 (10)	15 (9)	0.75
	H18 0|H18|K 12a [gi|293405409|ref|ZP_06649401| Hxx 0|0|FVEC1412] [gi|300899178|ref|ZP_07117455| Hxx 0|0|MS 198-1] [gi|218705418|ref|YP_002412937| Hxx 0|0|UMN026] [gi|218432515|ref|...					
☑ 1.8	gi|33590243	283	55093	10 (6)	9 (5)	0.42
	H16 NM|O157:NM(H16)|PC02					
	▶**3 samesets of gi|33590243**					
☑ 1.9	gi|46093552	273	44861	10 (5)	9 (4)	0.43
	H53 0|0|E480-68					
☑ 1.10	gi|5107837	85	56230	8 (4)	6 (3)	0.26
	H7 0|O128:H7|DEC 13a					

▼**66 peptide matches (62 non-duplicate, 4 duplicate)**

Query	Dupes	Observed	Mr(expt)	Mr(calc)	Delta	M	Score	Expect	Rank	U	1	2	3	4	5	6	7	8	9	10	Peptide
11		316.1739	630.3332	631.3653	-1.0321	0	2	5.5	▶2		■	■	■	■	■	■	■	■	■	■	R.LSSGLR.I
31		330.6995	659.3844	659.3238	0.0606	0	6	0.24	▶1	U						■					K.NNDAVK.T
62		355.1989	708.3832	708.3806	0.0026	0	14	0.28	▶1		■	■	■	■	■	■	■		■	■	R.FTSNIK.G
**85**		**380.6959**	**759.3772**	**759.3763**	**0.0010**	**0**	**32**	**0.0037**	▶1		■	■	■	■	■		■			■	**R.LDEIDR.V**
**97**		**389.2300**	**776.4454**	**776.4280**	**0.0175**	**0**	**16**	**0.026**	▶1	**U**							■				**K.TVITTDK.G**
104		395.7278	789.4410	789.3868	0.0542	0	2	0.64	▶1	U			■								K.DADGAITK.T
196		434.6877	867.3608	867.3610	-0.0002	0	8	0.15	▶1	U			■								K.FDTDADGK.I
**268**		**466.2512**	**930.4878**	**930.4883**	**-0.0004**	**0**	**61**	**3.7e-06**	▶1		■	■		■	■	■		■			**R.SSLGAVQNR**
271		466.7430	931.4714	930.4559	1.0155	0	1	3.8	▶1	U									■		K.DGAYHAAVK.N
**285**		**473.2534**	**944.4922**	**944.5039**	**-0.0117**	**0**	**55**	**9.7e-06**	▶1				■				■		■	■	**R.SSLGAIQNR**
365	▶1	502.2630	1002.5114	1002.5094	0.0020	1	17	0.11	▶1		■	■	■	■	■		■			■	K.SRLDEIDR.V
369		503.3683	1004.7220	1005.4727	-0.7507	0	0	0.97	▶1	U							■				K.TNAGTDTQAK.L

Query	Dupes	Observed	Mr(expt)	Mr(calc)	Delta	M	Score	Expect	Rank	U	1	2	3	4	5	6	7	8	9	10	Peptide
371		336.1957	1005.5653	1005.5607	0.0046	1	4	0.38	1		■	■									K.AIASVDKFR.S
450		539.2692	1076.5238	1077.4873	-0.9634	0	20	0.014	1	U								■			K.NDGSAQAIMR.E + Oxidation (M)
507		551.2671	1100.5196	1100.5210	-0.0014	0	71	7.9e-07	1		■	■	■	■	■	■	■	■	■		K.DDAAGQAIANR.F
569		569.7894	1137.5642	1137.5666	-0.0024	0	54	3.6e-06	1		■	■									K.ATADYVVQSGK.D
653		596.3035	1190.5924	1190.5891	0.0034	0	55	1.9e-05	1				■	■			■				K.NQSALSSSIER.L
670		600.8533	1199.6920	1199.6734	0.0186	1	13	0.049	1	U								■			K.LRSSLGAVQNR.F
680		603.3118	1204.6090	1204.6048	0.0043	0	46	5e-05	1		■				■	■					K.NQSALSTSIER.L
731		619.3093	1236.6040	1236.5986	0.0054	0	73	1.4e-07	1	U		■									K.NYSAGDAADILK.N
792		426.5494	1276.6264	1277.6616	-1.0352	0	0	0.96	1	U			■								R.AGGTLVNGAYDIK.T
811		430.2589	1287.7549	1288.5571	-0.8023	0	0	0.97	2	U				■							K.SYSFAADGADSAK.T
818	1	646.3385	1290.6624	1290.6568	0.0056	0	72	5.9e-08	1		■	■									K.DGAAQGFVTLQGK.N
834		650.8127	1299.6108	1299.6095	0.0013	0	88	1.6e-09	1	U		■									K.DFYAASVNAASGK.V
834		650.8127	1299.6108	1300.5935	-0.9827	0	29	0.0012	2	U	■										K.DFYAASVDAASGK.V
872		658.8795	1315.7444	1316.6685	-0.9240	0	2	0.65	1	U							■				K.LDNTGVTTAGVNR.Y
941		688.3207	1374.6268	1374.6304	-0.0035	0	62	6.5e-07	1	U			■								K.VSFDAGTSTDTFK.D
961		695.8829	1389.7512	1390.7303	-0.9791	1	5	0.32	1	U								■			K.TATKGAELSASDLK.A
977		470.6087	1408.8043	1409.6787	-0.8744	0	0	0.89	1	U									■		K.AEIQIDSHSADPK.A
1017		720.9130	1439.8114	1439.8096	0.0018	0	33	0.0022	1				■	■	■		■				K.AQIIQQAGNSVLAK.A
1038		728.9096	1455.8046	1455.8045	0.0001	0	105	4.4e-11	1		■					■					K.AQIIQQAGNSVLSK
1075		747.9189	1493.8232	1493.8202	0.0031	0	60	5.7e-06	1		■		■	■	■		■				K.ANQVPQQVLSLLQG.-
1168		789.4179	1576.8212	1576.8209	0.0003	0	42	7.9e-05	1		■	■		■	■		■				R.VSGQTQFNGVNVLSK
1189	1	803.4023	1604.7900	1604.7894	0.0007	0	116	2.6e-12	1	U		■									K.LTDLNLSDVTDTNGK.V
1197		538.9427	1613.8063	1613.8121	-0.0058	1	2	5.9	1		■	■	■	■	■	■	■	■	■		R.INSAKDDAAGQAIANR.F
1230		551.6332	1651.8778	1651.8781	-0.0003	1	48	1.6e-05	1		■	■									K.KIDSSTLGLNGFSVSK.N
1231		826.9470	1651.8794	1651.8781	0.0014	1	62	7.1e-07	1		■	■									K.KIDSSTLGLNGFSVSK.N
1272		852.4268	1702.8390	1702.8374	0.0017	0	98	1.7e-10	1	U						■					K.LGGDNGTTEIVVDAASGK.T
1294		860.3566	1718.6986	1718.7974	-0.0987	0	0	0.94	1	U								■			K.ALAYNDAPMSVYFGGK.N + Oxidation (M)
1319		581.6324	1741.8754	1740.8530	1.0224	0	1	0.88	2	U					■						K.QVNLLSYTDTASNSTK.Y
1328		584.9460	1751.8162	1751.9053	-0.0892	1	13	0.049	1	U									■		K.TIKAEIQIDSHSADPK.A
1370		597.9789	1790.9149	1790.9163	-0.0014	1	69	9.6e-07	1		■	■									K.VSADKDGAAQGFVTLQGK.N
1371		896.4655	1790.9164	1790.9163	0.0002	1	60	8.8e-06	1		■	■									K.VSADKDGAAQGFVTLQGK.N
1387		600.3233	1797.9481	1797.9472	0.0008	0	25	0.0043	1		■	■									K.LGVSDTSSLSLHNILDK.D
1559		695.7151	2084.1235	2084.1225	0.0009	0	86	1.7e-08	1		■		■	■	■		■	■	■		M.AQVINTNSLSLiTQNNiNK.N
1560		1043.0700	2084.1254	2084.1225	0.0029	0	124	2.6e-12	1		■		■	■	■		■	■	■		M.AQVINTNSLSLiTQNNiNK.N
1566		525.5301	2098.0913	2098.0906	0.0007	1	45	2.9e-05	1		■	■									K.LGVSDTSSLSLHNILDKDGK.A
1567	1	700.3713	2098.0921	2098.0906	0.0015	1	55	3.3e-06	1		■	■									K.LGVSDTSSLSLHNILDKDGK.A
1645		1132.0610	2262.1074	2262.1087	-0.0013	0	138	1.7e-14	1		■	■									R.LDSAITNLNNTTTNLSEAQSR.I
1645		1132.0610	2262.1074	2261.0883	1.0191	0	28	0.0016	2	U										■	R.LDSAVTNLNNTNTNLSEAQSR.I
1646		755.0435	2262.1087	2262.1087	-0.0001	0	71	7.9e-08	1		■	■									R.LDSAITNLNNTTTNLSEAQSR.I
1646		755.0435	2262.1087	2261.0883	1.0203	0	17	0.019	2	U										■	R.LDSAVTNLNNTNTNLSEAQSR.I
1666		1151.5400	2301.0654	2301.0649	0.0006	0	117	8.5e-12	1	U		■									K.VTLNTIDVTYDDYANGVDDAK.Q
1666		1151.5400	2301.0654	2300.0808	0.9846	0	89	5.4e-09	2	U	■										K.VTLNTINVTYDDYANGVDDAK.Q
1699		1219.1040	2436.1934	2436.1908	0.0027	0	99	1.3e-10	1		■	■									K.VTTTATEQFEGASTEDPLALLDK.A
1705		1245.6480	2489.2814	2489.2762	0.0053	0	121	7.5e-13	1	U				■							K.ASDLLANITDGSVITGGGANAFGVAAK.N
1723		881.7720	2642.2942	2642.2896	0.0046	0	42	0.00011	1		■	■	■			■					R.NANDGISLAQTTEGALSEINNNLQR.V
1724		1322.1550	2642.2954	2642.2896	0.0059	0	118	2.9e-12	1		■	■	■			■					R.NANDGISLAQTTEGALSEINNNLQR.V
1747		679.3328	2713.3021	2714.3763	-1.0742	1	2	0.7	1	U			■								R.AGGTLVNGAYDIKTTNTALTTTDAFAK.L
1760		933.5030	2797.4872	2797.4821	0.0050	0	56	2.6e-06	1	U					■						K.IQIGANDNQTISIGLQQIDSTTLNLK.G
1761		1399.7520	2797.4894	2797.4821	0.0073	0	38	0.00016	1	U					■						K.IQIGANDNQTISIGLQQIDSTTLNLK.G
1854		728.1957	3635.9421	3635.8894	0.0527	0	0	0.94	1	U						■					K.TSAAVNTLGGGAGSVAVDFATTSLTAITGLGSGAISEI

37 subsets and intersections (155 subset proteins in total)

2	gi|112820172	17	H21 0|EHEC serogroup: O113:H21|0

10 per page 1

Not what you expected? Try **the select summary**.

Mascot: http://www.matrixscience.com/
