# Supplementary material for: MS-H: A Novel Proteomic Approach to Isolate and Type the E. coli H Antigen Using Membrane Filtration and Liquid Chromatography-Tandem Mass Spectrometry (LC-MS/MS)
Source: PLoS One. 2013 Feb 21;8(2):e57339. doi: 10.1371/journal.pone.0057339 (PMC3578835; doi:10.1371/journal.pone.0057339)
Supplement: Representative Peptide Data S1 — Peptide data are represented as the Mascot search results from all 53 serotypes, obtained under the Orbitrap platform in Table 4 with related E. coli reference strains. “U” denotes a unique peptide specific for each of the proteins 1.1, 1.2, and beyond. The number 1.1 (shown as 1 in the peptide list and phylogenetic tree) represents the protein which obtained the highest score and confidence value after a Mascot search. This protein, known as the first hit, was used to designate the MS-H type of the unknown flagellin. Related peptides 1.2 (2), 1.3 (3), etc. represented the second, third, etc. hits for MS-H typing analysis. (DOCX) [file pone.0057339.s009.docx › H26-E194.pdf]

MATRIX SCIENCE MASCOT Search Results

User :
E-mail :
Search title : Submitted from 20110810-0587 by Mascot Daemon on VARIABLE
MS data file : C:\Documents and Settings\keding\Desktop\Raw data\20110811-001-0031-00587\20110811-006-EC194MS1.RAW
Database : Flagellin_v2 (192 sequences; 89,845 residues)
Taxonomy : Bacteria (Eubacteria) (192 sequences)
Timestamp : 12 Aug 2011 at 15:58:11 GMT

Not what you expected? Try **the select summary**.

- **Search parameters**
- **Score distribution**
- **Legend**

Protein Family Summary

Significance threshold p< 0.05 Max. number of families AUTO
Ions score or expect cut-off 0 Dendrograms cut at 0

Protein family 1 (out of 1)

10 per page 1

1

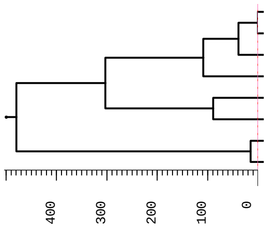

1	gi|30059888	8562	H26 0|H26|HW27
8	gi|59044701	202	H11 0|O26:H11|CL5
7	gi|33590227	1470	H42 NM|O157:NM(H42)|25
3	gi|121144335	2747	H55 0|O75:H55|E2987-73
2	gi|300316660	4278	Hxx(H9 99.7%) 0|0|MS 175-1 [gi|323940414|ref|EGB36605| Hxx 0|0|E482] [gi|300956560|ref|...
5	gi|30577587	1883	H17 0|O15:H17|P12b
4	gi|30059920	2426	H46 0|H46|5306-56
6	gi|16901500	1845	H49 0|H49|0

Threshold (**0**): 0

		Score	Mass	Matches	Sequences	emPAI
1.1	gi|30059888<br>H26 0|H26|HW27	8562	57263	165 (147)	59 (56)	96.61
1.2	gi|300316660<br>Hxx(H9 99.7%) 0|0|MS 175-1 [gi|323940414|ref|EGB36605| Hxx 0|0|E482] [gi|300956560|ref|ZP_07168842| Hxx 0|0|MS 175-1] [gi|300449175|ref|EFK12795| Hxx 0|0|MS 116-1] [gi|300951548|ref|...	4278	68134	79 (69)	28 (25)	4.44
1.3	gi|121144335<br>H55 0|O75:H55|E2987-73	2747	62285	59 (48)	22 (18)	3.01
1.4	gi|30059920<br>H46 0|H46|5306-56<br>**1 sameset of gi|30059920**	2426	57918	65 (48)	24 (21)	4.54
1.5	gi|30577587<br>H17 0|O15:H17|P12b	1883	36285	46 (37)	19 (17)	8.69
1.6	gi|16901500<br>H49 0|H49|0	1845	57940	58 (40)	22 (18)	3.20
1.7	gi|33590227<br>H42 NM|O157:NM(H42)|25	1470	44094	35 (27)	14 (11)	2.67
1.8	gi|59044701<br>H11 0|O26:H11|CL5	202	46459	13 (7)	6 (4)	0.51

206 peptide matches (109 non-duplicate, 97 duplicate)

Query	Dupes	Observed	Mr(expt)	Mr(calc)	Delta	M	Score	Expect	Rank	U	1	2	3	4	5	6	7	8	Peptide
16		313.1814	624.3482	624.3483	-0.0000	0	9	0.14	1	U	■								K.ITYTK.D
26	2	316.6900	631.3654	631.3653	0.0001	0	32	0.0059	1		■	■	■	■	■	■	■	■	R.LSSGLR.I
28		318.1970	634.3794	635.3643	-0.9848	0	1	0.85	1	U		■							K.LAGFTK.G
82		355.1974	708.3802	708.3806	-0.0004	0	19	0.075	1		■	■	■	■	■	■	■		R.FTSNIK.G
87	3	358.7059	715.3972	715.3977	-0.0004	0	26	0.019	1		■	■	■	■	■	■	■		K.GLTQAAR.N
113		373.7055	745.3964	745.3970	-0.0006	0	34	0.0004	1	U	■								K.ADAGTALK.G
127	1	380.6952	759.3758	759.3763	-0.0004	0	31	0.005	1		■	■	■	■	■	■			R.LDEIDR.V
130		381.7030	761.3914	761.3919	-0.0005	0	20	0.011	1	U	■								K.AADGSITK.D
139		386.7315	771.4484	771.4490	-0.0006	0	10	0.098	1	U					■				K.ALDAAIAK.V
252	1	423.2396	844.4646	844.4654	-0.0008	0	47	2e-05	1	U	■								K.ATAADVLGK.A
373	2	466.2506	930.4866	930.4883	-0.0016	0	82	2.6e-08	1		■	■	■		■				R.SSLGAVQNR.L
399		473.2534	944.4922	944.5039	-0.0117	0	32	0.0019	1					■		■			R.SSLGAIQNR.L
415		477.2491	952.4836	952.4866	-0.0029	1	31	0.00087	1	U	■								K.DTVTKFDK.A
416		318.5027	952.4863	952.4866	-0.0003	1	25	0.003	1	U	■								K.DTVTKFDK.A
429	2	481.2452	960.4758	960.4764	-0.0005	0	45	3.2e-05	1	U	■								K.SEATTDPLK.A
441		484.2451	966.4756	966.4771	-0.0014	0	47	2e-05	1	U	■								K.GYLGASNTGK.I
456	3	487.7487	973.4828	973.4828	-0.0000	0	71	1.1e-07	1	U	■								K.NTANLGAADK.A
512		502.2614	1002.5082	1002.5094	-0.0012	1	39	0.00073	1		■	■	■	■	■	■			K.SRLDEIDR.V
513		335.1770	1002.5092	1002.5094	-0.0002	1	31	0.0042	1		■	■	■	■	■	■			K.SRLDEIDR.V
691		551.2673	1100.5200	1100.5210	-0.0010	0	67	1.9e-06	1		■	■	■	■	■	■	■	■	K.DDAAGQAIANR.F

Query	Dupes	Observed	Mr(expt)	Mr(calc)	Delta	M	Score	Expect	Rank	U	1	2	3	4	5	6	7	8	Peptide
692		367.8475	1100.5207	1100.5210	-0.0004	0	45	0.00027	1		■	■	■	■	■	■	■	■	K.DDAAGQAIANR.F
764	1	571.2715	1140.5284	1140.5299	-0.0014	0	78	1.5e-08	1	U	■								K.VSDGEGSTVYK.A
800	1	580.2852	1158.5558	1158.5557	0.0001	0	63	5.3e-07	1	U	■								K.SYTFDATGVAK.A
823		390.5513	1168.6321	1168.6340	-0.0019	1	29	0.0014	1	U	■								K.ITYTKDTVTK.F
856	1	594.8092	1187.6038	1187.6034	0.0005	0	84	4.4e-09	1		■		■	■					K.ALDDAISQIDK.F
863	1	596.3019	1190.5892	1190.5891	0.0002	0	67	1.1e-06	1		■	■		■		■			K.NQSALSSSIER.L
868		598.8013	1195.5880	1194.5517	1.0364	0	3	0.46	1	U						■			K.DAAQSSIDFGGK.K
927	1	614.7964	1227.5782	1227.5772	0.0011	0	63	4.9e-07	1	U	■								K.FDQTAGTVDFK.G
948		618.3344	1234.6542	1234.6557	-0.0015	1	65	1.9e-06	1	U	■								K.FDKATAADVLGK.A
948		618.3344	1234.6542	1235.6146	-0.9604	0	3	3	3	U								■	R.VSEQTQFNGVK.V
949	2	412.5594	1234.6564	1234.6557	0.0006	1	23	0.033	1	U	■								K.FDKATAADVLGK.A
949	1	412.5594	1234.6564	1235.6146	-0.9583	0	6	1.6	2	U								■	R.VSEQTQFNGVK.V
956		620.8334	1239.6522	1238.5561	1.0962	0	2	0.69	1	U			■						K.NQSSMSTAIER.L + Oxidation (M)
1037	1	426.8891	1277.6455	1277.6463	-0.0008	1	31	0.00074	1	U	■								K.AADGSITKDATTK.S
1038		639.8303	1277.6460	1277.6463	-0.0003	1	102	5.7e-11	1	U	■								K.AADGSITKDATTK.S
1079		651.8624	1301.7102	1301.6827	0.0276	0	5	0.71	1	U								■	K.AATLSDLDLNAAK.K
1137		672.8781	1343.7416	1343.7408	0.0008	0	83	5.1e-09	1	U			■						-.SLSLITQNNINK.N
1246		480.9437	1439.8093	1439.8096	-0.0003	0	51	3.3e-05	1		■	■	■	■		■	■		K.AQIIQQAGNSVLAK.A
1247	2	720.9122	1439.8098	1439.8096	0.0002	0	112	3e-11	1		■	■	■	■		■	■		K.AQIIQQAGNSVLAK.A
1291		493.2508	1476.7306	1476.7308	-0.0002	1	21	0.0082	1	U	■								K.DATTKSEATTDPLK.A
1292		739.3726	1476.7306	1476.7308	-0.0001	1	72	7e-08	1	U	■								K.DATTKSEATTDPLK.A
1302		495.9352	1484.7838	1484.7834	0.0003	0	72	9.2e-08	1	U	■								K.INIGGTEQEVNIAK.D
1303	1	743.3995	1484.7844	1484.7834	0.0010	0	97	3.2e-10	1	U	■								K.INIGGTEQEVNIAK.D
1306		744.8853	1487.7560	1487.7580	-0.0019	1	108	1.7e-11	1	U	■								K.NTANLGAADKATVDK.L
1308		496.9260	1487.7562	1487.7580	-0.0018	1	54	4.3e-06	1	U	■								K.NTANLGAADKATVDK.L
1312		746.3934	1490.7722	1490.7729	-0.0006	1	89	1.2e-09	1		■		■	■					K.ALDDAISQIDKFR.S
1313		497.9321	1490.7745	1490.7729	0.0016	1	44	4.1e-05	1		■		■	■					K.ALDDAISQIDKFR.S
1319	1	747.9191	1493.8236	1493.8202	0.0035	0	62	3.5e-06	1		■	■		■	■	■	■		K.ANQVPQQVLSLLQG.-
1404		780.9058	1559.7970	1560.8260	-1.0290	0	7	0.95	1				■	■		■	■		R.VSGQTQFNGVNVLAK
1437	3	789.4180	1576.8214	1576.8209	0.0005	0	94	4.6e-10	1		■	■			■				R.VSGQTQFNGVNVLSK
1438		526.6146	1576.8220	1576.8209	0.0010	0	54	5.5e-06	1		■	■			■				R.VSGQTQFNGVNVLSK
1470		807.9126	1613.8106	1613.8121	-0.0015	1	94	3.6e-09	1		■	■	■	■	■	■	■	■	R.INSAKDDAAGQAIANR.F
1471	1	538.9442	1613.8108	1613.8121	-0.0013	1	44	0.00036	1		■	■	■	■	■	■	■	■	R.INSAKDDAAGQAIANR.F
1490	1	812.8760	1623.7374	1623.7376	-0.0002	0	129	1.4e-13	1	U	■								K.DLTDAGFTASAADANGK.I
1491		542.2538	1623.7396	1623.7376	0.0019	0	71	8.5e-08	1	U	■								K.DLTDAGFTASAADANGK.I
1545	1	836.3800	1670.7454	1670.7457	-0.0003	0	132	3.8e-13	1		■	■	■	■	■	■	■		R.IQDADYATEVSNMSK.A
1546		557.9230	1670.7472	1670.7457	0.0014	0	61	5.3e-06	1		■	■	■	■	■	■	■		R.IQDADYATEVSNMSK.A
1554		560.6609	1678.9609	1678.8526	0.1083	0	0	0.92	3	U			■						K.IDSSTLNLTGFNVNGK.G
1571	12	843.9506	1685.8866	1685.8836	0.0031	0	125	3.1e-12	1		■	■	■						K.IQVGANDGETITIDLK.K
1571	12	843.9506	1685.8866	1684.8996	0.9871	0	45	0.0003	2					■		■			K.IQVGANDGQTITIDLK.K
1574		844.3774	1686.7402	1686.7407	-0.0004	0	107	1.5e-10	1		■	■	■	■	■	■	■		R.IQDADYATEVSNMSK.A + Oxidation (M)
1585		565.6287	1693.8643	1693.8635	0.0008	1	31	0.0008	1	U	■								K.ADAGTALKGYLGASNTGK.I
1643	2	871.4409	1740.8672	1740.8683	-0.0010	0	141	8.3e-15	1	U	■								K.AYAVTAGAVQTGGADVYK.D
1644		581.2968	1740.8686	1740.8683	0.0003	0	18	0.015	1	U	■								K.AYAVTAGAVQTGGADVYK.D
1690		597.9709	1790.8909	1790.8911	-0.0002	1	46	0.00022	1		■	■	■	■	■	■	■		K.DDAAGQAIANRFTSNIK.G
1709		602.3170	1803.9292	1803.9438	-0.0147	1	0	5.4	1		■	■		■		■			K.NQSALSSSIERLSSGLR.I
1766		628.9775	1883.9107	1883.9113	-0.0006	1	46	2.4e-05	1	U	■								K.VSDGEGSTVYKAADGSITK.D
1807	9	972.9733	1943.9320	1943.9299	0.0022	0	131	1.2e-13	1	U	■								K.LFAGAQDATITFDSGMTAK.F
1815	1	648.9858	1943.9356	1943.9299	0.0057	0	26	0.0038	1	U	■								K.LFAGAQDATITFDSGMTAK.F
1826	1	980.9705	1959.9264	1959.9248	0.0016	0	64	8.3e-07	1	U	■								K.LFAGAQDATITFDSGMTAK.F + Oxidation (M)
1845		991.4756	1980.9366	1980.9389	-0.0022	1	132	1.2e-13	1	U	■								K.ATGKDLTDAGFTASAADANGK.I
1846	1	661.3198	1980.9376	1980.9389	-0.0013	1	56	4.2e-06	1	U	■								K.ATGKDLTDAGFTASAADANGK.I
1888	2	1043.0690	2084.1234	2084.1225	0.0009	0	120	6.2e-12	1		■	■		■		■			M.AQVINTNSLSLITQNNINK.N
1889		695.7160	2084.1262	2085.0814	-0.9552	0	84	2.5e-08	1	U					■				M.AQVINTNSLSLNTQNNINK.N
1889		695.7160	2084.1262	2084.1225	0.0036	0	75	2.2e-07	2		■	■		■		■			M.AQVINTNSLSLITQNNINK.N
1889		695.7160	2084.1262	2085.1066	-0.9804	0	74	2.8e-07	5	U							■		M.AQVINTNSLSLITQNNIDK.N
1890	2	1043.5610	2085.1074	2085.0814	0.0260	0	96	1.6e-09	1	U					■				M.AQVINTNSLSLNTQNNINK.N
1890	2	1043.5610	2085.1074	2085.1066	0.0009	0	88	1.1e-08	2	U							■		M.AQVINTNSLSLITQNNIDK.N
1912		711.0325	2130.0757	2130.0692	0.0065	1	50	1.1e-05	1	U	■								K.SEATTDPLKALDDAISQIDK.F
1959	1	750.3723	2248.0951	2248.0931	0.0020	0	114	2.3e-11	1		■	■	■	■	■		■		R.LDSAVTNLNNTTTNLSEAQSR.I
1960	1	1125.0550	2248.0954	2248.0931	0.0023	0	124	2.4e-12	1		■	■	■	■	■		■		R.LDSAVTNLNNTTTNLSEAQSR.I
1972	2	1136.0360	2270.0574	2270.0550	0.0024	0	140	1.1e-14	1	U	■								K.DGSITDTNGDALYLDSTGNLTK.N
1986	1	1146.5600	2291.1054	2291.1030	0.0025	0	127	2.1e-13	1	U	■								K.IDSDTLNLAGFNVNGAGSVDNAK.A
2002		773.7375	2318.1907	2318.1866	0.0040	1	71	1.8e-07	1		■	■			■				R.LDEIDRVSGQTQFNGVNVLSK
2013		786.3736	2356.0990	2356.0965	0.0025	0	65	3.4e-07	1	U	■								K.GASISADAMASTLNNGSYTANVGGK.A
2014	2	1179.0570	2356.0994	2356.0965	0.0030	0	123	4.8e-13	1	U	■								K.GASISADAMASTLNNGSYTANVGGK.A
2034		807.4064	2419.1974	2419.1979	-0.0006	1	88	1.5e-09	1	U	■								K.KIDSDTLNLAGFNVNGAGSVDNAK.A
2035		1210.6080	2419.2014	2419.1979	0.0035	1	124	4.2e-13	1	U	■								K.KIDSDTLNLAGFNVNGAGSVDNAK.A
2047		820.4096	2458.2070	2458.2050	0.0020	1	66	2.4e-07	1	U	■								K.ATVDKLFAGAQDATITFDSGMTAK.F
2052		1245.6480	2489.2814	2489.2762	0.0053	0	112	6.7e-12	1	U		■							K.ASDLLANITDGSVITGGGANAFGVAAK.N
2066	5	1290.6120	2579.2094	2579.2086	0.0008	0	120	1e-12	1		■	■							R.ELTVQATTGTNSDSDLDSIQDEIK.S
2067		860.7440	2579.2102	2579.2086	0.0016	0	50	9.5e-06	1		■	■							R.ELTVQATTGTNSDSDLDSIQDEIK.S
2079		1306.6330	2611.2514	2612.2790	-1.0275	0	22	0.0064	1	U					■				R.NANDGISLAQTAEGALSEINNNLQR.I
2085		877.0992	2628.2758	2628.2739	0.0019	0	80	4.2e-08	1		■	■	■	■		■			R.NANDGISVAQTTEGALSEINNNLQR
2087	1	1315.1470	2628.2794	2628.2739	0.0055	0	136	1e-13	1		■	■	■	■		■			R.NANDGISVAQTTEGALSEINNNLQR
2094		1321.5880	2641.1614	2641.1555	0.0059	0	94	6.9e-10	1	U	■								K.DTTGALTTEDDETVTATYYGFADGK.V
2116		677.0489	2704.1665	2703.2848	0.8817	0	13	0.045	1	U								■	R.NANDGISFAQTTEGALNEINNNLQR.V
2127		1399.7500	2797.4854	2797.4821	0.0033	0	35	0.00033	1	U					■				K.IQIGANDNQTISIGLQQIDSTTLNLK.G
2128		933.5029	2797.4869	2797.4821	0.0047	0	54	4.1e-06	1	U					■				K.IQIGANDNQTISIGLQQIDSTTLNLK.G
2131		941.7895	2822.3467	2822.3418	0.0049	1	82	6.4e-09	1		■	■							R.ELTVQATTGTNSDSDLDSIQDEIKSR.L
2133		950.4726	2848.3960	2848.3938	0.0022	1	119	1.9e-12	1		■	■							R.IRELTVQATTGTNSDSDLDSIQDEIK.S
2133		950.4726	2848.3960	2847.4098	0.9862	1	18	0.023	2	U				■					R.VRELTVQATTGTNSQSDLDSIQDEIK.S
2160	4	1027.8160	3080.4262	3080.4211	0.0051	0	93	4.6e-10	1	U	■								K.AAAGDSITYAGTDTGLGVAADASTYTYNAANK.S

Query	Dupes	Observed	Mr(expt)	Mr(calc)	Delta	M	Score	Expect	Rank	U	1	2	3	4	5	6	7	8	Peptide
2161	2	1541.2210	3080.4274	3080.4211	0.0064	0	104	4.1e-11	1	U	■								K.AAAGDSITYAGTDTGLGVAADASTYTYNAANK.S
2165		1031.8560	3092.5462	3092.5448	0.0014	1	45	0.00014	1		■	■	■	■		■	■		R.IQDADYATEVSNMSKAQIIQQAGNSVLAK.A
2178		1054.5320	3160.5742	3160.5708	0.0033	1	99	4e-10	1		■	■	■		■				R.SSLGAVQNRLDSAVTNLNNTTTNLSEAQSR.I
2178		1054.5320	3160.5742	3160.5708	0.0034	1	32	0.0018	3	U						■			R.SSLGAIQNRLDSAVTNLNNTSTNLSEAQSR.I
2186	1	1086.5750	3256.7032	3256.7011	0.0021	1	133	2.2e-13	1		■	■		■		■			M.AQVINTNSLSLITQNNINKNQSALSSSIER.L
2209		1125.9420	3374.8042	3374.7570	0.0472	1	0	0.97	1	U							■		K.IDSSALGLSGFSVAGGALKLSDTVTQVGDGSAAPVK.V

63 subsets and intersections (162 subset proteins in total)

10 per page 1

Not what you expected? Try **the select summary**.

Mascot: http://www.matrixscience.com/
