# Supplementary material for: MS-H: A Novel Proteomic Approach to Isolate and Type the E. coli H Antigen Using Membrane Filtration and Liquid Chromatography-Tandem Mass Spectrometry (LC-MS/MS)
Source: PLoS One. 2013 Feb 21;8(2):e57339. doi: 10.1371/journal.pone.0057339 (PMC3578835; doi:10.1371/journal.pone.0057339)
Supplement: Representative Peptide Data S1 — Peptide data are represented as the Mascot search results from all 53 serotypes, obtained under the Orbitrap platform in Table 4 with related E. coli reference strains. “U” denotes a unique peptide specific for each of the proteins 1.1, 1.2, and beyond. The number 1.1 (shown as 1 in the peptide list and phylogenetic tree) represents the protein which obtained the highest score and confidence value after a Mascot search. This protein, known as the first hit, was used to designate the MS-H type of the unknown flagellin. Related peptides 1.2 (2), 1.3 (3), etc. represented the second, third, etc. hits for MS-H typing analysis. (DOCX) [file pone.0057339.s009.docx › H27-E195.pdf]

MASCOT Search Results

User :
E-mail :
Search title : Submitted from 20110815-0595-02 by Mascot Daemon on VARIABLE
MS data file : C:\Documents and Settings\keding\Desktop\Raw data\20110815-001-0031-00595\20110815-005-EC195MS3rp.RAW
Database : Flagellin_v2 (192 sequences; 89,845 residues)
Taxonomy : Bacteria (Eubacteria) (192 sequences)
Timestamp : 18 Aug 2011 at 19:06:00 GMT

Not what you expected? Try **the select summary**.

Search parameters

Score distribution

Legend

Protein Family Summary

Significance threshold p< 0.05 Max. number of families AUTO
Ions score or expect cut-off 0 Dendrograms cut at 0

Protein families 1–2 (out of 2)

10 per page 1

1

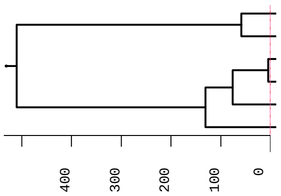

1	gi|218427480	2031	Hxx(H27 99.6%) 0|0|ED1a [gi|218689916|ref|YP_002398128| Hxx 0|0|ED1a]
4	gi|46093545	550	H3 0|0|Bi7327-41
2	gi|30059888	837	H26 0|H26|HW27
5	gi|30059928	522	H52 0|H52|C2187-69
3	gi|16901498	596	H34 0|H34|0
6	gi|33590227	428	H42 NM|O157:NM(H42)|25

Threshold (**0**): 0

		Score	Mass	Matches	Sequences	emPAI
1.1	**gi|218427480**<br>Hxx(H27 99.6%) 0|0|ED1a [gi|218689916|ref|YP_002398128| Hxx 0|0|ED1a]	2031	50836	56 (44)	35 (31)	12.99
1.2	**gi|30059888**<br>H26 0|H26|HW27<br>**1 sameset of gi|30059888**	837	57263	26 (17)	21 (14)	1.44
1.3	**gi|16901498**<br>H34 0|H34|0<br>**3 samesets of gi|16901498**	596	56006	19 (14)	16 (12)	1.22
1.4	**gi|46093545**<br>H3 0|0|Bi7327-41	550	55534	21 (12)	14 (8)	0.88
1.5	**gi|30059928**<br>H52 0|H52|C2187-69	522	46003	18 (14)	13 (11)	1.46
1.6	**gi|33590227**<br>H42 NM|O157:NM(H42)|25	428	44094	14 (11)	11 (9)	1.21

89 peptide matches (81 non-duplicate, 8 duplicate)

Query	Dupes	Observed	Mr(expt)	Mr(calc)	Delta	M	Score	Expect	Rank	U	1	2	3	4	5	6	Peptide
5		302.1705	602.3264	601.3911	0.9353	1	1	1.1	2	U				■			K.TVVRK.D
11		308.1854	614.3562	615.3591	-1.0029	0	2	1.3	1	U				■			K.NLEIK.Q
30	1	316.6896	631.3646	631.3653	-0.0007	0	24	0.039	1		■	■	■	■	■	■	R.LSSGLR.I
61		330.2076	658.4006	658.4014	-0.0007	0	13	0.05	1	U	■						K.AAVSLAK.D
76		338.2051	674.3956	674.3963	-0.0007	0	10	0.13	1	U	■						K.TVTGLGK.T
94		347.1997	692.3848	692.3857	-0.0009	0	19	0.028	1	U	■						R.FTANIK.G
114		358.7058	715.3970	715.3977	-0.0006	0	29	0.0089	1			■	■		■	■	K.GLTQAAR.N
124		366.7034	731.3922	731.3926	-0.0003	0	30	0.0032	1	U	■						K.GLTQASR.N
128		371.6935	741.3724	741.3731	-0.0006	0	18	0.015	1	U	■						K.TMYLSK.S
147		379.6906	757.3666	757.3680	-0.0014	0	30	0.0011	1	U	■						K.TMYLSK.S + Oxidation (M)
152		380.6950	759.3754	759.3763	-0.0008	0	27	0.011	1			■	■		■		R.LDEIDR.V
155		381.2000	760.3854	761.3919	-1.0065	0	3	2.5	1	U		■					K.AADGSITK.D
172		387.7030	773.3914	773.3919	-0.0005	0	32	0.0013	1	U	■						R.LEEIDR.V
216		405.2047	808.3948	808.3967	-0.0018	0	23	0.0051	1	U	■						K.TSDPVYK.N
366		446.2419	890.4692	890.4709	-0.0017	0	45	0.0001	1	U	■						K.ATGSDLISK.F
439	2	466.2506	930.4866	930.4883	-0.0016	0	49	6e-05	1			■		■	■		R.SSLGAVQNR
472		473.2584	944.5022	944.5039	-0.0017	0	54	1.2e-05	1	U			■				R.SSLGAIQNR.L
504		480.2481	958.4816	958.4832	-0.0016	0	75	5.2e-08	1	U	■						R.SDLGAVQNR.F
504		480.2481	958.4816	958.5196	-0.0379	0	36	0.00047	2	U						■	R.SSLGVVQNR.L
507		481.2499	960.4852	960.4764	0.0089	0	2	0.68	1	U		■					K.SEATTDPLK.A
537		487.7519	973.4892	973.4828	0.0064	0	4	0.62	1	U		■					K.NTANLGAADK.A

Query	Dupes	Observed	Mr(expt)	Mr(calc)	Delta	M	Score	Expect	Rank	U	1	2	3	4	5	6	Peptide
549		489.6762	977.3378	978.5134	-1.1756	0	18	0.017	▶1	U					■		K.GFSVSGNALK.V
646		510.7159	1019.4172	1020.5274	-1.1101	0	1	1.2	▶2	U				■			K.VLATNQTMK.I + Oxidation (M)
694		524.2599	1046.5052	1046.5066	-0.0014	0	22	0.011	▶1	U	■						K.VLAENNEMK.I
712		527.7612	1053.5078	1053.5091	-0.0012	0	57	3.5e-06	▶1	U	■						K.NSAGQFTTTK.V
731		532.2570	1062.4994	1062.5015	-0.0021	0	37	0.00044	▶1	U	■						K.VLAENNEMK.I + Oxidation (M)
746	▶1	536.2762	1070.5378	1070.5397	-0.0018	0	39	0.00013	▶1	U	■						K.YQGITFTNK.G
811		551.2671	1100.5196	1100.5210	-0.0014	0	72	5.6e-07	▶1		■	■	■	■	■	■	K.DDAAGQAIANR.F
897		381.1603	1140.4591	1140.5299	-0.0708	0	5	0.33	▶1	U		■					K.VSDGEGSTVYK.A
913		573.8086	1145.6026	1144.6564	0.9463	1	6	1.2	▶1		■	■	■	■	■	■	R.LSSGLRINSAK.D
947		580.3179	1158.6212	1158.6245	-0.0032	0	64	4.3e-07	▶1	U	■						K.VGGTGIDATGLAK.A
959		582.7958	1163.5770	1163.5782	-0.0012	0	61	2.2e-06	▶1		■			■			K.SQSSLSSAIER.L
962		584.2973	1166.5800	1166.5819	-0.0019	0	68	3.8e-07	▶1	U	■						K.DVTFTIDATGK.D
964	▶1	390.1799	1167.5179	1166.5819	0.9359	0	1	0.74	▶1	U	■						K.DVTFTIDATGK.D
997		596.3011	1190.5876	1190.5891	-0.0014	0	50	5.9e-05	▶1			■	■		■		K.NQSALSSSIER.L
1014		600.8522	1199.6898	1199.6734	0.0164	1	12	0.069	▶1	U				■			K.LRSSLGAVQNR.F
1033		606.8635	1211.7124	1211.5856	0.1268	1	0	0.89	▶1	U			■				K.ADGMVKDGYIK.G + Oxidation (M)
1080	▶1	618.8135	1235.6124	1235.6146	-0.0022	0	70	2.5e-07	▶1	U	■						R.VSEQTQFNGVK.V
1080		618.8135	1235.6124	1234.6306	0.9818	0	41	0.00019	▶2	U				■			R.VSQQTQFNGVK.V
1176		644.3062	1286.5978	1286.5990	-0.0012	0	52	6.5e-06	▶1	U			■				K.GLTSSDGSTAYTK.T
1208	▶1	652.3275	1302.6404	1302.6415	-0.0011	0	87	4.1e-09	▶1	U	■						K.AATASDLDLNNAK.K
1391		716.3738	1430.7330	1430.7365	-0.0034	1	63	8.8e-07	▶1	U	■						K.AATASDLDLNNAKK.V
1392		477.9185	1430.7337	1430.7365	-0.0028	1	14	0.062	▶1	U	■						K.AATASDLDLNNAKK.V
1408		720.9109	1439.8072	1439.8096	-0.0024	0	80	4.5e-08	▶1			■	■			■	K.AQIIQQAGNSVLAK.A
1418		724.8719	1447.7292	1447.7307	-0.0015	0	79	2.7e-08	▶1	U	■						K.TLGLDGFNIDGAQK.A
1473		747.9168	1493.8190	1493.8202	-0.0011	0	46	0.00014	▶1			■	■		■	■	K.ANQVPQQVLSLLQG.-
1480		500.9342	1499.7808	1499.7831	-0.0023	1	18	0.017	▶1	U	■						K.SEGGSPILVKEDAAK.S
1481		750.8979	1499.7812	1499.7831	-0.0019	1	69	1.3e-07	▶1	U	■						K.SEGGSPILVKEDAAK.S
1519		508.9259	1523.7559	1523.7580	-0.0021	1	8	0.3	▶1	U	■						K.NSAGQFTTTKVENK.A
1541		773.9009	1545.7872	1545.7886	-0.0014	0	80	1.7e-08	▶1	U	■						K.SLQSTTNPLETIDK.A
1632		538.9438	1613.8096	1613.8121	-0.0025	1	34	0.0037	▶1		■	■	■	■	■	■	R.INSAKDDAAGQAIANR.F
1633		807.9124	1613.8102	1613.8121	-0.0019	1	116	2.1e-11	▶1		■	■	■	■	■	■	R.INSAKDDAAGQAIANR.F
1641		812.8760	1623.7374	1623.7376	-0.0002	0	96	2.7e-10	▶1	U		■					K.DLTDAGFTASAADANGK.I
1670		823.9094	1645.8042	1644.9046	0.8996	1	0	1	▶1	U			■				K.TTANTAAKGSDILAALK.T
1679		553.3042	1656.8908	1657.8523	-0.9615	0	8	0.16	▶1	U						■	K.VDLDAAATDIGTALGQK.V
1696		836.3796	1670.7446	1670.7457	-0.0011	0	109	8e-11	▶1			■	■		■	■	R.IQDADYATEVSNMSK.A
1733		850.8738	1699.7330	1699.7359	-0.0029	0	125	5.3e-13	▶1	U	■						R.IEDADYATEVSNMSR.A
1758		858.8702	1715.7258	1715.7308	-0.0050	0	70	2.2e-07	▶1	U	■						R.IEDADYATEVSNMSR.A + Oxidation (M)
1787		871.4400	1740.8654	1740.8683	-0.0028	0	124	4.1e-13	▶1	U		■					K.AYAVTAGAVQTGGADVYK.D
1813		590.5894	1768.7464	1769.9159	-1.1696	0	0	1.1	▶1	U				■			K.IQVGANDGQTIEIGLDK.I
1861		902.4510	1802.8874	1803.9438	-1.0564	1	1	3.6	▶1			■	■		■		K.NQSALSSSIERLSSGLR.I
1912		622.9735	1865.8987	1865.9007	-0.0020	0	37	0.00022	▶1	U	■						K.VGSSLVVNGADYEVSADGK.T
1913		933.9570	1865.8994	1865.9007	-0.0012	0	115	3.1e-12	▶1	U	■						K.VGSSLVVNGADYEVSADGK.T
1955		955.9381	1909.8616	1909.8654	-0.0037	0	117	2.2e-12	▶1	U	■						K.GTDAFDGSGNGTLTANIDGK.D
1980		972.4401	1942.8656	1942.8690	-0.0034	1	51	1.4e-05	▶1	U	■						R.SRIEDADYATEVSNMSR.A
1981		648.6295	1942.8667	1942.8690	-0.0024	1	54	7.2e-06	▶1	U	■						R.SRIEDADYATEVSNMSR.A
1990	▶1	653.8138	1958.4196	1958.8640	-0.4444	1	3	1.1	▶1	U	■						R.SRIEDADYATEVSNMSR.A + Oxidation (M)
2018		664.6718	1990.9936	1990.9960	-0.0024	1	45	4.8e-05	▶1	U	■						R.LEEIDRVSEQTQFNGVK.V
2021		997.4990	1992.9834	1992.9865	-0.0030	0	109	3.5e-11	▶1		■			■			R.FDSAITNLGNTVNNLSSAR.S
2022		665.3354	1992.9844	1992.9865	-0.0021	0	48	4e-05	▶1		■			■			R.FDSAITNLGNTVNNLSSAR.S
2023		499.4685	1993.8449	1992.9865	0.8584	0	11	0.2	▶1		■			■			R.FDSAITNLGNTVNNLSSAR.S
2024		665.6716	1993.9930	1993.9957	-0.0027	1	72	1.7e-07	▶1	U	■						K.KVGSSLVVNGADYEVSADGK.T
2025		998.0039	1993.9932	1993.9957	-0.0024	1	95	8.7e-10	▶1	U	■						K.KVGSSLVVNGADYEVSADGK.T
2078		1043.0670	2084.1194	2084.1225	-0.0031	0	108	9.4e-11	▶1		■	■	■	■	■		M.AQVINTNSLSLiTQNNiNK.N
2078		1043.0670	2084.1194	2085.1066	-0.9871	0	49	8.7e-05	▶4	U						■	M.AQVINTNSLSLITQNNIDK.N
2079		695.7145	2084.1217	2084.1225	-0.0009	0	55	2.2e-05	▶1		■	■	■	■	■		M.AQVINTNSLSLiTQNNiNK.N
2079		695.7145	2084.1217	2085.1066	-0.9849	0	38	0.0011	▶5	U						■	M.AQVINTNSLSLITQNNIDK.N
2154		558.3077	2229.2017	2230.0753	-0.8737	1	6	0.25	▶1	U		■					K.DLTDAGFTASAADANGKITYTK.D
2176		1136.0330	2270.0514	2270.0550	-0.0036	0	78	1.6e-08	▶1	U		■					K.DGSITDTNGDALYLDSTGNLTK.N
2255		886.1019	2655.2839	2655.2848	-0.0009	0	86	6.2e-09	▶1	U	■						R.NANDGISVAQTTEGALNEINNNLQR.V
2256		1328.6500	2655.2854	2655.2848	0.0006	0	124	9.2e-13	▶1	U	■						R.NANDGISVAQTTEGALNEINNNLQR.V

▶60 subsets and intersections (163 subset proteins in total)

▶2 **gi|46093564** 22 Hxx(H54 100.0%) 0|0|E223-69 [gi|283982455|ref|ADB56974| H54 0|O161:H54|O161:...

10 per page 1

Not what you expected? Try **the select summary**.

Mascot: http://www.matrixscience.com/
