# Supplementary material for: MS-H: A Novel Proteomic Approach to Isolate and Type the E. coli H Antigen Using Membrane Filtration and Liquid Chromatography-Tandem Mass Spectrometry (LC-MS/MS)
Source: PLoS One. 2013 Feb 21;8(2):e57339. doi: 10.1371/journal.pone.0057339 (PMC3578835; doi:10.1371/journal.pone.0057339)
Supplement: Representative Peptide Data S1 — Peptide data are represented as the Mascot search results from all 53 serotypes, obtained under the Orbitrap platform in Table 4 with related E. coli reference strains. “U” denotes a unique peptide specific for each of the proteins 1.1, 1.2, and beyond. The number 1.1 (shown as 1 in the peptide list and phylogenetic tree) represents the protein which obtained the highest score and confidence value after a Mascot search. This protein, known as the first hit, was used to designate the MS-H type of the unknown flagellin. Related peptides 1.2 (2), 1.3 (3), etc. represented the second, third, etc. hits for MS-H typing analysis. (DOCX) [file pone.0057339.s009.docx › H28-E196.pdf]

MATRIX SCIENCE MASCOT Search Results

User :
E-mail :
Search title : Submitted from 20110810-0587 by Mascot Daemon on VARIABLE
MS data file : C:\Documents and Settings\keding\Desktop\Raw data\20110811-001-0031-00587\20110811-008-EC196MS1.RAW
Database : Flagellin_v2 (192 sequences; 89,845 residues)
Taxonomy : Bacteria (Eubacteria) (192 sequences)
Timestamp : 12 Aug 2011 at 16:02:10 GMT

Not what you expected? Try **the select summary**.

Search parameters

Score distribution

Legend

Protein Family Summary

Significance threshold p< 0.05 Max. number of families AUTO
Ions score or expect cut-off 0 Dendrograms cut at 0

Protein family 1 (out of 1)

10 per page 1

1

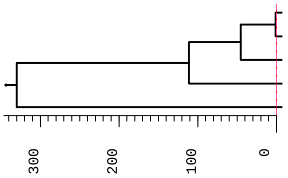

1	gi|30059892	3095	H28 0|H28|HW30
5	gi|33590252	200	Hxx(H8 89.5%) 0|0|PC41
4	gi|40994711	621	H4 0|O154:H4|0
2	gi|3377710	1481	H9 0|0|O78:K80; EC341 95 [gi|30059860|ref|AAP13302| H9 0|H9|Bi 7575-41]
3	gi|30059888	1400	H26 0|H26|HW27

Threshold (**0**): 0

		Score	Mass	Matches	Sequences	emPAI
1.1	gi|30059892<br>H28 0|H28|HW30	3095	59307	54 (50)	28 (27)	5.99
1.2	gi|3377710<br>H9 0|0|O78:K80; EC341 95 [gi|30059860|ref|AAP13302| H9 0|H9|Bi 7575-41]	1481	68093	25 (22)	15 (14)	1.57
1.3	gi|30059888<br>H26 0|H26|HW27<br>**1 sameset of gi|30059888**	1400	57263	23 (20)	14 (13)	1.73
1.4	gi|40994711<br>H4 0|O154:H4|0	621	36224	10 (9)	7 (7)	1.20
1.5	gi|33590252<br>Hxx(H8 89.5%) 0|0|PC41	200	52373	8 (3)	4 (2)	0.20

69 peptide matches (48 non-duplicate, 21 duplicate)

Query	Dupes	Observed	Mr(expt)	Mr(calc)	Delta	M	Score	Expect	Rank	U	1	2	3	4	5	Peptide
31	2	421.7590	841.5034	841.4658	0.0377	0	14	0.039	1	U					■	K.AVTQPQAK.D
66	1	466.2505	930.4864	930.4883	-0.0018	0	32	0.0031	1		■	■	■	■		R.SSLGAVQNR.L
213		576.7808	1151.5470	1151.5492	-0.0022	0	76	2.5e-08	1	U	■					R.MSAESLQSATK.S
224		581.3029	1160.5912	1160.5925	-0.0012	0	90	1.3e-09	1		■	■				K.ALDEAISSIDK.F
347		653.3165	1304.6184	1305.6776	-1.0592	0	3	0.55	1	U					■	K.LGTDTATASITGAK.L
436		720.9112	1439.8078	1439.8096	-0.0018	0	96	1.1e-09	1		■	■	■	■		K.AQIIQQAGNSVLAK.A
455	6	731.3940	1460.7734	1460.7722	0.0012	0	98	1.6e-10	1	U	■					K.IGSTSIDVVLASDGK.I
464	1	488.9281	1463.7625	1463.7620	0.0005	1	60	1.3e-06	1		■	■				K.ALDEAISSIDKFR.S
466		732.8896	1463.7646	1463.7620	0.0027	1	81	1.2e-08	1		■	■				K.ALDEAISSIDKFR.S
487		747.9193	1493.8240	1493.8202	0.0039	0	63	3e-06	1		■	■	■	■		K.ANQVPQQVLSLLQG.-
540	1	773.9065	1545.7984	1545.7998	-0.0014	0	106	4.6e-11	1	U	■					K.TSTAADVIASLANNAK.V
541		516.2741	1545.8005	1545.7998	0.0007	0	48	2.7e-05	1	U	■					K.TSTAADVIASLANNAK.V
559	1	781.4194	1560.8242	1560.8260	-0.0018	0	57	8.8e-06	1	U	■					R.VSGQTQFNGVNVLAK.D
560		521.2848	1560.8326	1560.8260	0.0066	0	32	0.0026	1	U	■					R.VSGQTQFNGVNVLAK.D
644	1	556.4179	1666.2319	1664.8621	1.3698	0	8	0.17	1	U					■	K.AAATETTSFGGTPITLK.N
647		836.3795	1670.7444	1670.7457	-0.0013	0	101	5.1e-10	1		■	■	■	■		R.IQDADYATEVSNMSK.A
663		843.9492	1685.8838	1684.8996	0.9843	0	80	1e-07	1	U	■					K.IQVGANDGQTITIDLK.K
663		843.9492	1685.8838	1685.8836	0.0003	0	76	2.5e-07	2			■	■			K.IQVGANDGETITIDLK.K
740		892.4423	1782.8700	1782.8636	0.0064	0	93	5.1e-10	1	U	■					K.STGFTVDVGATGTSAGDIK.V
744	1	894.9203	1787.8260	1787.8214	0.0047	0	76	3e-08	1	U	■					K.AVYVSADGNFTTDAETK.A
763	1	902.9424	1803.8702	1803.9438	-0.0736	1	5	1.7	2		■	■	■			K.NQSALSSSIERLSSGLR.I
780		919.9609	1837.9072	1837.9058	0.0015	0	111	7.7e-12	1	U	■					K.ADGSLTTDNTTNLFLQK.D
786		617.9662	1850.8768	1850.8799	-0.0031	0	17	0.018	1	U	■					K.GIVQQHTGTGFEDAYTK.A
846	1	972.9736	1943.9326	1943.9299	0.0028	0	96	3.9e-10	1	U			■			K.LFAGAQDATITFDSGMTAK.F

Query	Dupes	Observed	Mr(expt)	Mr(calc)	Delta	M	Score	Expect	Rank	U	1	2	3	4	5	Peptide
902		677.3619	2029.0639	2029.0579	0.0060	1	47	2e-05	▶1	U	■					K.AATTADPLKALDEAISSIDK.F
929		695.7155	2084.1247	2084.1225	0.0021	0	81	4.9e-08	▶1		■	■	■	■	■	M.AQVINTNSLSLiTQNNiNK.N
930		1043.0700	2084.1254	2084.1225	0.0029	0	142	4.6e-14	▶1		■	■	■	■	■	M.AQVINTNSLSLiTQNNiNK.N
999		1125.0580	2248.1014	2248.0931	0.0083	0	138	1e-13	▶1		■	■	■	■		R.LDSAVTNLNNTTTNLSEAQSR.I
1000		750.3757	2248.1053	2248.0931	0.0122	0	64	2.3e-06	▶1		■	■	■	■		R.LDSAVTNLNNTTTNLSEAQSR.I
1006		757.6906	2270.0500	2270.0550	-0.0051	0	53	5.5e-06	▶1	U			■			K.DGSITDTNGDALYLDSTGNLTK.N
1007		1136.0350	2270.0554	2270.0550	0.0004	0	132	6.9e-14	▶1	U			■			K.DGSITDTNGDALYLDSTGNLTK.N
1025		768.4056	2302.1950	2302.1917	0.0032	1	71	3.5e-07	▶1	U	■					R.LDEIDRVSGQTQFNGVNVLAK.D
1026	▶1	1154.0600	2306.1054	2306.1026	0.0028	0	115	3.5e-12	▶1	U	■					K.DGSELFIDVDGNLTQNNAGTVK.A
1042		786.3735	2356.0987	2356.0965	0.0022	0	63	5.3e-07	▶1	U			■			K.GASISADAMASTLNNGSYTANVGGK.A
1076		827.4142	2479.2208	2479.2191	0.0017	0	88	1.7e-09	▶1	U	■					K.IDSDTLGLSGFNVNGSGAVANTAATK.S
1079		1245.6480	2489.2814	2489.2762	0.0053	0	120	8.9e-13	▶1	U		■				K.ASDLLANITDGSVITGGGANAFGVAAK.N
1093		856.0712	2565.1918	2565.1930	-0.0012	0	32	0.002	▶1		■	■				R.ELTVQASTGTNSDSDLDSIQDEIK.S
1094		1283.6050	2565.1954	2565.1930	0.0025	0	120	3.3e-12	▶1		■	■				R.ELTVQASTGTNSDSDLDSIQDEIK.S
1103		1304.6660	2607.3174	2607.3140	0.0034	1	78	1.5e-08	▶1	U	■					K.KIDSDTLGLSGFNVNGSGAVANTAATK.S
1104	▶1	870.1136	2607.3190	2607.3140	0.0049	1	73	5e-08	▶1	U	■					K.KIDSDTLGLSGFNVNGSGAVANTAATK.S
1110		877.0982	2628.2728	2628.2739	-0.0011	0	35	0.0016	▶1		■	■	■			R.NANDGISVAQTTEGALSEINNNLQR.I
1111	▶1	1315.1440	2628.2734	2628.2739	-0.0005	0	124	2e-12	▶1		■	■	■			R.NANDGISVAQTTEGALSEINNNLQR.I
1143	▶2	916.1490	2745.4252	2745.4185	0.0067	0	75	3.4e-08	▶1	U	■					K.SDLAAAQLLAPGTADANGTVTYTVGAGLK.T
1150		933.5047	2797.4923	2797.4821	0.0101	0	59	1.4e-06	▶1	U				■		K.IQIGANDNQTISIGLQQIDSTTLNLK.G
1155		945.8007	2834.3803	2834.3781	0.0021	1	106	8.7e-11	▶1		■	■				R.IRELTVQASTGTNSDSDLDSIQDEIK.S
1172		1027.8180	3080.4322	3080.4211	0.0111	0	89	1.1e-09	▶1	U			■			K.AAAGDSITYAGTDTGLGVAADASTYTYNAANK.S
1188		1086.5750	3256.7032	3256.7011	0.0021	1	129	5.6e-13	▶1		■	■	■			M.AQVINTNSLSLITQNNINKNQSALSSSIER.L
1189		815.1835	3256.7049	3256.7011	0.0038	1	29	0.0056	▶1		■	■	■			M.AQVINTNSLSLITQNNINKNQSALSSSIER.L

▶**65 subsets and intersections (143 subset proteins in total)**

10 per page 1

Not what you expected? Try **the select summary**.

Mascot: http://www.matrixscience.com/
