# Supplementary material for: MS-H: A Novel Proteomic Approach to Isolate and Type the E. coli H Antigen Using Membrane Filtration and Liquid Chromatography-Tandem Mass Spectrometry (LC-MS/MS)
Source: PLoS One. 2013 Feb 21;8(2):e57339. doi: 10.1371/journal.pone.0057339 (PMC3578835; doi:10.1371/journal.pone.0057339)
Supplement: Representative Peptide Data S1 — Peptide data are represented as the Mascot search results from all 53 serotypes, obtained under the Orbitrap platform in Table 4 with related E. coli reference strains. “U” denotes a unique peptide specific for each of the proteins 1.1, 1.2, and beyond. The number 1.1 (shown as 1 in the peptide list and phylogenetic tree) represents the protein which obtained the highest score and confidence value after a Mascot search. This protein, known as the first hit, was used to designate the MS-H type of the unknown flagellin. Related peptides 1.2 (2), 1.3 (3), etc. represented the second, third, etc. hits for MS-H typing analysis. (DOCX) [file pone.0057339.s009.docx › H29-E197.pdf]

MASCOT Search Results

User :
E-mail :
Search title : Submitted from 20110810-0587 by Mascot Daemon on VARIABLE
MS data file : C:\Documents and Settings\keding\Desktop\Raw data\20110811-001-0031-00587\20110811-009-EC197MS1.RAW
Database : Flagellin_v2 (192 sequences; 89,845 residues)
Taxonomy : Bacteria (Eubacteria) (192 sequences)
Timestamp : 12 Aug 2011 at 16:03:48 GMT

Not what you expected? Try **the select summary**.

Search parameters

Score distribution

Legend

Protein Family Summary

Significance threshold p< 0.05 Max. number of families AUTO
Ions score or expect cut-off 0 Dendrograms cut at 0

Protein families 1–2 (out of 2)

10 per page 1

1

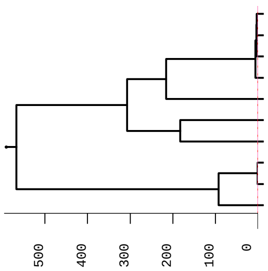

1	gi|30059896	11208	H29 0|H29|HW31
4	gi|6941886	3570	H43 0|O157:H43|O157:H43 [gi|30059916|ref|AAP13330| H43 0|H43|149-51]
10	gi|112820171	621	H21 0|EHEC serogroup: O113:H21|0 [gi|291323135|ref|EFE62563| Hxx 0|0|B088] [gi|29344630...
2	gi|33590260	8997	H29 0|O157:H29|Y63
9	gi|30059888	1195	H26 0|H26|HW27
8	gi|30577587	1336	H17 0|O15:H17|P12b
3	gi|33590227	3986	H42 NM|O157:NM(H42)|25
5	gi|30059892	1763	H28 0|H28|HW30
7	gi|323186803	1355	Hxx(H45 99.1%) 0|0|RN587/1
6	gi|145997	1714	Hxx(H12 99.7%) 0|0|Bi 316-42 (Orskov)

Threshold (**0**): 0

		Score	Mass	Matches	Sequences	emPAI
1.1	gi|30059896<br>H29 0|H29|HW31	11208	45760	199 (180)	26 (26)	19.02
1.2	gi|33590260<br>H29 0|O157:H29|Y63	8997	45720	162 (145)	23 (23)	10.46
1.3	gi|33590227<br>H42 NM|O157:NM(H42)|25	3986	44094	71 (64)	12 (12)	2.42
1.4	gi|6941886<br>H43 0|O157:H43|O157:H43 [gi|30059916|ref|AAP13330| H43 0|H43|149-51]	3570	51071	67 (59)	11 (11)	1.56
1.5	gi|30059892<br>H28 0|H28|HW30	1763	59307	39 (32)	14 (14)	1.50
1.6	gi|145997<br>Hxx(H12 99.7%) 0|0|Bi 316-42 (Orskov)	1714	61008	39 (33)	10 (9)	0.98
1.7	gi|323186803<br>Hxx(H45 99.1%) 0|0|RN587/1	1355	57844	32 (27)	10 (10)	1.05
1.8	gi|30577587<br>H17 0|O15:H17|P12b	1336	36285	37 (34)	11 (10)	1.85
1.9	gi|30059888<br>H26 0|H26|HW27<br>**1 sameset of gi|30059888**	1195	57263	23 (22)	9 (9)	0.75
1.10	gi|112820171<br>H21 0|EHEC serogroup: O113:H21|0 [gi|291323135|ref|EFE62563| Hxx 0|0|B088] [gi|293446305|ref|ZP_06662727| Hxx 0|0|B088]	621	51472	15 (14)	3 (3)	0.28

300 peptide matches (70 non-duplicate, 230 duplicate)

Query	Dupes	Observed	Mr(expt)	Mr(calc)	Delta	M	Score	Expect	Rank	U	1	2	3	4	5	6	7	8	9	10	Peptide
13		387.2846	772.5546	771.4490	1.1056	0	4	1.1	1	U								■			K.ALDAAIAK.V
61		466.2516	930.4886	930.4883	0.0004	0	54	2e-05	1		■	■		■	■			■	■		R.SSLGAVQNR.L
86		495.3526	988.6906	989.5142	-0.8235	0	17	0.023	1	U										■	K.NSAGQLTATK.V
219		603.3083	1204.6020	1204.6048	-0.0027	0	54	6.6e-06	1		■	■	■					■			K.NQSALSTSIER.L
231		609.7847	1217.5548	1217.5888	-0.0340	0	19	0.011	1	U		■									R.VTIDGDGTNQAK.I
254	21	613.3079	1224.6012	1224.5986	0.0026	0	80	9e-09	1		■	■									K.SYAASVDAGGTVK.L
420	17	723.3671	1444.7196	1444.7158	0.0039	0	109	1.3e-11	1	U	■										R.VTIDGDGSLGANQAK.I
430	3	728.9088	1455.8030	1455.8045	-0.0015	0	111	1.2e-11	1		■	■		■				■			K.AQIIQQAGNSVLSK.A
435	3	731.3953	1460.7760	1460.7722	0.0038	0	94	4.4e-10	1	U					■						K.IGSTSIDVVLASDGK.I
454		498.8630	1493.5672	1492.6868	0.8804	0	1	1.5	1	U						■					K.TASVTMGGTTYNFK.T + Oxidation (M)
455	1	747.9185	1493.8224	1493.8202	0.0023	0	75	1.8e-07	1		■	■	■	■	■	■	■	■	■		K.ANQVPQQVLSLLQG.-
479	2	508.9355	1523.7847	1523.7831	0.0015	0	54	6.9e-06	1		■	■									K.IDSSTLGLNGFSVSK.N
480	4	762.9017	1523.7888	1523.7831	0.0057	0	109	2e-11	1		■	■									K.IDSSTLGLNGFSVSK.N
500	11	772.8820	1543.7494	1543.7519	-0.0024	0	113	5.3e-12	1		■	■									K.GGVATSTYVVQYGDK.S

Query	Dupes	Observed	Mr(expt)	Mr(calc)	Delta	M	Score	Expect	Rank	U	1	2	3	4	5	6	7	8	9	10	Peptide
531	▶5	781.4200	1560.8254	1560.8260	-0.0006	0	88	7.4e-09	▶1		■	■	■	■	■	■	■				R.VSGQTQFNGVNVLAK
533		521.2830	1560.8272	1560.8260	0.0012	0	56	1.3e-05	▶1		■	■	■	■	■	■	■				R.VSGQTQFNGVNVLAK
583		538.9461	1613.8165	1613.8121	0.0044	1	42	0.00058	▶1		■	■	■	■	■	■	■	■	■	■	R.INSAKDDAAGQAIANR.F
629	▶1	836.3795	1670.7444	1670.7457	-0.0013	0	121	5.2e-12	▶1		■	■	■	■	■	■	■	■	■		R.IQDADYATEVSNMSK.A
700		597.9712	1790.8918	1790.8911	0.0007	1	58	1.3e-05	▶1		■	■	■	■	■	■	■	■	■		K.DDAAGQAIANRFTSNIK.G
708	▶15	900.4783	1798.9420	1798.9789	-0.0368	1	132	1.2e-13	▶1	U	■	■		■							K.IQVGANDGQTISIDLkK.I
713	▶9	600.6550	1798.9432	1798.9789	-0.0357	1	59	2.6e-06	▶1	U	■	■		■							K.IQVGANDGQTISIDLkK.I
748	▶1	919.9609	1837.9072	1837.9058	0.0015	0	117	2e-12	▶1	U					■						K.ADGSLTTDNTTNLFLQK.D
824	▶2	1010.4940	2018.9734	2018.9769	-0.0035	0	169	1.3e-17	▶1	U	■										K.NYVANDSLVNANGAAGAAATR.V
825	▶1	673.9991	2018.9755	2018.9769	-0.0015	0	86	2.8e-09	▶1	U	■										K.NYVANDSLVNANGAAGAAATR.V
844	▶3	1043.0700	2084.1254	2085.0814	-0.9560	0	78	1.1e-07	▶4	U								■			M.AQVINTNSLSLNTQNNINK.N
845	▶3	1043.0700	2084.1254	2084.1225	0.0029	0	134	2.5e-13	▶1		■	■		■	■	■	■		■	■	M.AQVINTNSLSLiTQNNiNK.N
845	▶3	1043.0700	2084.1254	2085.1066	-0.9811	0	79	9e-08	▶4	U			■								M.AQVINTNSLSLITQNNIDK.N
849	▶8	695.7162	2084.1268	2084.1225	0.0042	0	67	1.3e-06	▶1		■	■		■	■	■	■		■	■	M.AQVINTNSLSLiTQNNiNK.N
852	▶7	696.0428	2085.1066	2085.1066	0.0000	0	66	1.8e-06	▶1	U			■								M.AQVINTNSLSLITQNNIDK.N
852	▶8	696.0428	2085.1066	2085.0814	0.0252	0	64	2.4e-06	▶2	U								■			M.AQVINTNSLSLNTQNNINK.N
884		1111.0570	2220.0994	2220.0982	0.0013	0	130	4.4e-13	▶1		■	■									R.LSSAVTNLNNTTTNLSEAQSR.I
885	▶1	741.0409	2220.1009	2220.0982	0.0027	0	73	2.1e-07	▶1		■	■									R.LSSAVTNLNNTTTNLSEAQSR.I
901		751.0410	2250.1012	2249.1209	0.9802	1	9	0.14	▶1	U							■				K.DSSMKIQVGANDGQTITIDLK.K + Oxidation (M)
902		1126.0600	2250.1054	2249.1209	0.9845	1	14	0.041	▶1	U							■				K.DSSMKIQVGANDGQTITIDLK.K + Oxidation (M)
921		1152.1040	2302.1934	2302.1917	0.0017	1	97	8.4e-10	▶1		■	■			■	■	■				R.LDEIDRVSGQTQFNGVNVLAK.D
922		768.4052	2302.1938	2302.1917	0.0020	1	55	1.4e-05	▶1		■	■			■	■	■				R.LDEIDRVSGQTQFNGVNVLAK.D
931	▶3	774.7480	2321.2222	2321.2226	-0.0005	0	64	7.8e-07	▶1	U	■										K.NALETSEAITQLPNGANAPIAVK.M
932		1161.6190	2321.2234	2321.2226	0.0008	0	94	8.4e-10	▶1	U	■										K.NALETSEAITQLPNGANAPIAVK.M
934		581.3143	2321.2281	2321.2226	0.0055	0	44	7.1e-05	▶1	U	■										K.NALETSEAITQLPNGANAPIAVK.M
948		786.3769	2356.1089	2356.0965	0.0124	0	77	2.1e-08	▶1	U									■		K.GASISADAMASTLNNGSYTANVGGK.A
979	▶6	839.0951	2514.2635	2514.2636	-0.0001	0	75	3.1e-08	▶1		■	■									K.MDASVLTDLNITDASAVSLHNVTK.G
980		1258.1400	2514.2654	2514.2636	0.0019	0	95	3.5e-10	▶1		■	■									K.MDASVLTDLNITDASAVSLHNVTK.G
983		629.5741	2514.2673	2514.2636	0.0037	0	49	1.1e-05	▶1		■	■									K.MDASVLTDLNITDASAVSLHNVTK.G
986		844.4280	2530.2622	2530.2585	0.0037	0	73	4.8e-08	▶1		■	■									K.MDASVLTDLNITDASAVSLHNVTK.G + Oxidation
998	▶10	851.4120	2551.2142	2551.2137	0.0005	0	72	8.9e-08	▶1		■	■	■								R.ELTVQATTGTNSDSDLSSIQDEIK.S
1005	▶11	1276.6150	2551.2154	2551.2137	0.0017	0	165	4.7e-17	▶1		■	■	■								R.ELTVQATTGTNSDSDLSSIQDEIK.S
1023		856.0727	2565.1963	2565.1930	0.0033	0	66	8.1e-07	▶1						■	■	■				R.ELTVQASTGTNSDSDLDSIQDEIK.S
1023		856.0727	2565.1963	2565.2293	-0.0331	0	20	0.03	▶3	U				■							R.ELTVQATTGTNSESDLSSIQDEIK.S
1046		870.1120	2607.3142	2607.3140	0.0001	1	82	5.9e-09	▶1	U					■						K.KIDSDTLGLSGFNVNGSGAVANTAATK.S
1049		877.0995	2628.2767	2628.2739	0.0028	0	73	2.1e-07	▶1						■		■		■		R.NANDGISVAQTTEGALSEINNNLQR.I
1054	▶3	881.7717	2642.2933	2642.2896	0.0037	0	80	1.7e-08	▶1		■	■	■	■							R.NANDGISLAQTTEGALSEINNNLQR.V
1056	▶4	1322.1540	2642.2934	2642.2896	0.0039	0	125	5.9e-13	▶1		■	■	■	■							R.NANDGISLAQTTEGALSEINNNLQR.V
1056	▶4	1322.1540	2642.2934	2642.2896	0.0039	0	107	3.1e-11	▶3	U						■					R.NANDAISVAQTTEGALSEINNNLQR.I
1057	▶3	881.7726	2642.2960	2642.2896	0.0064	0	63	8e-07	▶3	U						■					R.NANDAISVAQTTEGALSEINNNLQR.I
1085		916.1476	2745.4210	2745.4185	0.0025	0	55	3.5e-06	▶1	U					■						K.SDLAAAQLLAPGTADANGTVTYTVGAGLK.T
1088		917.7895	2750.3467	2750.3399	0.0067	1	47	1.9e-05	▶1		■	■									K.GGVATSTYVVQYGDKSYAASVDAGGTVK.L
1093		932.4573	2794.3501	2794.3468	0.0032	1	103	6.4e-11	▶1		■	■	■								R.ELTVQATTGTNSDSDLSSIQDEIKSR.L
1095		562.2692	2806.3096	2806.3832	-0.0736	1	2	1.1	▶1		■	■	■								R.VRELTVQATTGTNSDSDLSSIQDEIK.S
1105	▶8	936.4706	2806.3900	2806.3832	0.0067	1	110	1.9e-11	▶1		■	■	■								R.VRELTVQATTGTNSDSDLSSIQDEIK.S
1105	▶8	936.4706	2806.3900	2806.3832	0.0068	1	79	2.2e-08	▶2	U								■			R.IRELTVQASTGTNSDSDLSSIQDEIK.S
1119		734.6210	2934.4549	2934.4458	0.0091	0	61	1.5e-06	▶1	U	■										K.IELSQNGATAATSEFAGASTNDPLTLLDK.A
1121		1468.2360	2934.4574	2934.4458	0.0116	0	101	1.3e-10	▶1	U	■										K.IELSQNGATAATSEFAGASTNDPLTLLDK.A
1122	▶6	979.1608	2934.4606	2934.4458	0.0148	0	110	1.5e-11	▶1	U	■										K.IELSQNGATAATSEFAGASTNDPLTLLDK.A
1143		1027.8150	3080.4232	3080.4211	0.0021	0	110	9.9e-12	▶1	U									■		K.AAAGDSITYAGTDTGLGVAADASTYTYNAANK.S
1158		1091.2470	3270.7192	3271.6756	-0.9564	1	119	1.7e-12	▶1	U								■			M.AQVINTNSLSLNTQNNINKNQSALSTSIER.L
1158		1091.2470	3270.7192	3271.7008	-0.9816	1	113	6.6e-12	▶2	U			■								M.AQVINTNSLSLITQNNIDKNQSALSTSIER.L
1158		1091.2470	3270.7192	3270.7167	0.0024	1	110	1.4e-11	▶3		■	■									M.AQVINTNSLSLITQNNINKNQSALSTSIER.L
1159		818.6874	3270.7205	3270.7167	0.0038	1	42	8.8e-05	▶1		■	■									M.AQVINTNSLSLITQNNINKNQSALSTSIER.L
1159		818.6874	3270.7205	3271.7008	-0.9803	1	39	0.00019	▶2	U			■								M.AQVINTNSLSLITQNNIDKNQSALSTSIER.L
1159		818.6874	3270.7205	3271.6756	-0.9551	1	35	0.00051	▶3	U								■			M.AQVINTNSLSLNTQNNINKNQSALSTSIER.L

▶52 subsets and intersections (160 subset proteins in total)

▶2 gi|307553085 147 Hxx(H54 27.9%) 0|0|ABU 83972

10 per page 1

Not what you expected? Try the select summary.

Mascot: http://www.matrixscience.com/
