# Supplementary material for: MS-H: A Novel Proteomic Approach to Isolate and Type the E. coli H Antigen Using Membrane Filtration and Liquid Chromatography-Tandem Mass Spectrometry (LC-MS/MS)
Source: PLoS One. 2013 Feb 21;8(2):e57339. doi: 10.1371/journal.pone.0057339 (PMC3578835; doi:10.1371/journal.pone.0057339)
Supplement: Representative Peptide Data S1 — Peptide data are represented as the Mascot search results from all 53 serotypes, obtained under the Orbitrap platform in Table 4 with related E. coli reference strains. “U” denotes a unique peptide specific for each of the proteins 1.1, 1.2, and beyond. The number 1.1 (shown as 1 in the peptide list and phylogenetic tree) represents the protein which obtained the highest score and confidence value after a Mascot search. This protein, known as the first hit, was used to designate the MS-H type of the unknown flagellin. Related peptides 1.2 (2), 1.3 (3), etc. represented the second, third, etc. hits for MS-H typing analysis. (DOCX) [file pone.0057339.s009.docx › H30-E198.pdf]

MATRIX SCIENCE MASCOT Search Results

User :
E-mail :
Search title : Submitted from 20110811-0587-02 by Mascot Daemon on VARIABLE
MS data file : C:\Documents and Settings\keding\Desktop\Raw data\20110811-001-0031-00587\20110811-010-EC198MS1rp.RAW
Database : Flagellin_v2 (192 sequences; 89,845 residues)
Taxonomy : Bacteria (Eubacteria) (192 sequences)
Timestamp : 15 Aug 2011 at 15:25:29 GMT

Not what you expected? Try **the select summary**.

- **Search parameters**
- **Score distribution**
- **Legend**

Protein Family Summary

Significance threshold p< 0.05 Max. number of families AUTO
Ions score or expect cut-off 0 Dendrograms cut at 0

Protein family 1 (out of 1)

10 per page 1

1

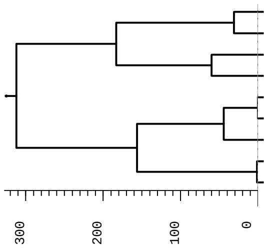

1	gi|30059894	2654	H30 0|H30|HW32 [gi|188494683|ref|ZP_03001953| Hxx 0|0|53638] [gi|188489882|ref|EDU649...
7	gi|30577587	835	H17 0|O15:H17|P12b
9	gi|30059896	623	H29 0|H29|HW31
5	gi|6941886	903	H43 0|O157:H43|O157:H43 [gi|30059916|ref|AAP13330| H43 0|H43|149-51]
2	gi|6941882	1178	H14 0|O138:H14|O138:H14
6	gi|88683097	882	H44 0|H44|0
3	gi|16901498	1137	H34 0|H34|0
4	gi|121144335	963	H55 0|O75:H55|E2987-73
8	gi|4731618	765	Hxx(H7 98.7%) 0|0|DEC 3f

Threshold (**0**): 0

		Score	Mass	Matches	Sequences	emPAI
1.1	gi|30059894	2654	58102	77 (62)	36 (31)	14.74
	H30 0|H30|HW32 [gi|188494683|ref|ZP_03001953| Hxx 0|0|53638] [gi|188489882|ref|EDU64985| Hxx 0|0|53638]					
1.2	gi|6941882	1178	56492	42 (28)	25 (18)	3.61
	H14 0|O138:H14|O138:H14					
1.3	gi|16901498	1137	56006	35 (27)	19 (17)	3.41
	H34 0|H34|0					
	1 sameset of gi|16901498					
1.4	gi|121144335	963	62285	29 (18)	18 (12)	1.52
	H55 0|O75:H55|E2987-73					
1.5	gi|6941886	903	51071	29 (22)	17 (12)	2.28
	H43 0|O157:H43|O157:H43 [gi|30059916|ref|AAP13330| H43 0|H43|149-51]					
1.6	gi|88683097	882	55289	31 (19)	20 (13)	2.00
	H44 0|H44|0					
1.7	gi|30577587	835	36285	27 (20)	16 (12)	4.26
	H17 0|O15:H17|P12b					
1.8	gi|4731618	765	56848	21 (14)	12 (9)	1.20
	Hxx(H7 98.7%) 0|0|DEC 3f					
1.9	gi|30059896	623	45760	25 (19)	17 (13)	2.51
	H29 0|H29|HW31					

115 peptide matches (90 non-duplicate, 25 duplicate)

Query	Dupes	Observed	Mr(expt)	Mr(calc)	Delta	M	Score	Expect	Rank	U	1	2	3	4	5	6	7	8	9	Peptide
7		316.6904	631.3662	631.3653	0.0009	0	19	0.086	1		■	■	■	■	■	■	■	■	■	R.LSSGLR
10		322.7035	643.3924	643.3905	0.0020	0	10	0.1	1	U	■									K.VADVLK.A
34		358.7143	715.4140	715.3977	0.0164	0	7	0.67	1		■	■	■	■	■	■	■	■	■	K.GLTQAAR.N
39		364.2274	726.4402	727.3613	-0.9210	0	17	0.02	1	U			■							K.NQAGNPK.K
56		380.6962	759.3778	759.3763	0.0016	0	35	0.0018	1		■	■	■	■		■	■	■	■	R.LDEIDR.V
58		382.2138	762.4130	762.4123	0.0007	0	19	0.014	1	U	■									K.IDSSTLK.L
162		439.7060	877.3974	876.4552	0.9422	0	12	0.057	1	U		■								K.AATTADSLK.A
163		439.7229	877.4312	877.4393	-0.0080	0	2	0.56	1	U		■								K.LTTDAETK.A
211	1	466.2520	930.4894	930.4883	0.0012	0	63	2.1e-06	1		■	■		■	■	■	■		■	R.SSLGAVQNR.L
218		468.7518	935.4890	936.4586	-0.9696	0	2	0.58	1	U		■								K.AATTADMLK.A + Oxidation (M)
264	2	490.2653	978.5160	978.5134	0.0026	0	43	6.1e-05	1	U	■									K.LTSFNVNGK.G
275		494.7699	987.5252	988.5189	-0.9937	0	10	0.11	1	U					■					K.TNLVTAADGK.T
276		495.2626	988.5106	988.5077	0.0030	0	31	0.00086	1	U	■									K.SEVTTDPLK.A
296		502.2625	1002.5104	1002.5094	0.0010	1	49	7.3e-05	1		■	■	■	■		■	■	■	■	K.SRLDEIDR.V
297		335.1779	1002.5119	1002.5094	0.0025	1	17	0.13	1		■	■	■	■		■	■	■	■	K.SRLDEIDR.V
301		503.7890	1005.5634	1005.5607	0.0027	1	7	0.21	1	U									■	K.AIASVDKFR.S
436		551.2687	1100.5228	1100.5210	0.0018	0	72	6.1e-07	1		■	■	■	■	■	■	■		■	K.DDAAGQAIANR.F

Query	Dupes	Observed	Mr(expt)	Mr(calc)	Delta	M	Score	Expect	Rank	U	1	2	3	4	5	6	7	8	9	Peptide
454		559.2917	1116.5688	1116.5663	0.0026	0	61	8.1e-07	1	U	■									K.ALDDAIASVDK.F
483		570.2424	1138.4702	1138.5479	-0.0777	0	14	0.037	1	U								■		K.DHSAGQAIANR.F
568	1	596.3027	1190.5908	1190.5891	0.0018	0	52	3.8e-05	1		■	■	■		■					K.NQSALSSSIER.L
570		397.8713	1190.5921	1190.5891	0.0030	0	8	0.86	1		■	■	■		■					K.NQSALSSSIER.L
591		602.7928	1203.5710	1203.6571	-0.0861	1	1	1.9	1	U								■		R.SSIERLSSGLR.L
594		603.3109	1204.6072	1204.6048	0.0025	0	17	0.037	1								■		■	K.NQSALSTSIER.L
620	1	611.3027	1220.5908	1220.5885	0.0024	0	72	6.2e-08	1	U	■									K.AADGSLTTEATGK.S
642		617.8269	1233.6392	1233.6578	-0.0186	1	5	1.2	1		■	■		■	■	■	■		■	K.FRSSLGAVQNR.L
831		672.8789	1343.7432	1343.7408	0.0024	0	55	3.3e-06	1	U				■						-.SLSLITQNNINK.N
856		683.3245	1364.6344	1364.6783	-0.0439	0	1	0.78	1	U				■						K.GSVSNTAATTDTLK.L
899		703.3475	1404.6804	1403.6391	1.0413	0	2	2	1	U							■			K.GFTVSGMADFSAAK.L + Oxidation (M)
918	1	710.8776	1419.7406	1419.7358	0.0049	1	52	6.1e-06	1	U	■									K.ALDDAIASVDKFR.S
919		474.2542	1419.7408	1419.7358	0.0050	1	39	0.00014	1	U	■									K.ALDDAIASVDKFR.S
944	2	720.9139	1439.8132	1439.8096	0.0036	0	120	4.1e-12	1		■	■	■	■		■		■		K.AQIIQQAGNSVLAK.A
945		480.9454	1439.8144	1439.8096	0.0048	0	68	6.9e-07	1		■	■	■	■		■		■		K.AQIIQQAGNSVLAK.A
954		723.3673	1444.7200	1444.7158	0.0043	0	21	0.0081	1	U									■	R.VTIDGDGSLGANQAK.I
1007		747.9195	1493.8244	1493.8202	0.0043	0	48	0.0001	1	U	■	■	■		■	■	■		■	K.ANQVPQQVLSLxQG.-
1008		498.9491	1493.8255	1494.7791	-0.9536	0	22	0.036	1	U						■				K.ANQVPQQVLSLNQG.-
1008		498.9491	1493.8255	1493.8202	0.0053	0	22	0.038	2		■	■	■		■		■		■	K.ANQVPQQVLSLLQG.-
1043		760.5603	1519.1060	1517.7950	1.3110	0	4	0.38	1	U						■				K.ANQVPQQVLSLHQG.-
1061		773.0520	1544.0894	1543.7995	0.2900	0	2	0.69	1	U						■				K.ANQVPQQVLSLYQG.-
1081		781.4227	1560.8308	1560.8260	0.0048	0	30	0.0045	1			■	■	■	■	■		■	■	R.VSGQTQFNGVNVLAK.D
1104	2	789.4200	1576.8254	1576.8209	0.0045	0	82	8.5e-09	1		■						■			R.VSGQTQFNGVNVLSK
1105		526.6160	1576.8262	1576.8209	0.0052	0	32	0.00085	1		■						■			R.VSGQTQFNGVNVLSK
1141		807.9139	1613.8132	1613.8121	0.0011	1	66	2.2e-06	1		■	■	■	■	■	■	■		■	R.INSAKDDAAGQAIANR.F
1142		538.9459	1613.8159	1613.8121	0.0038	1	41	0.0007	1		■	■	■	■	■	■	■		■	R.INSAKDDAAGQAIANR.F
1194		836.3822	1670.7498	1670.7457	0.0041	0	123	3.1e-12	1		■	■	■	■	■	■	■	■	■	R.IQDADYATEVSNMSK.A
1195		557.9244	1670.7514	1670.7457	0.0056	0	43	0.00033	1		■	■	■	■	■	■	■	■	■	R.IQDADYATEVSNMSK.A
1207	1	843.9518	1685.8890	1685.8836	0.0055	0	76	2.7e-07	1		■			■				■		K.IQVGANDGETITIDLK.K
1207	1	843.9518	1685.8890	1684.8996	0.9895	0	30	0.01	2			■	■			■				K.IQVGANDGQTITIDLK
1208		844.3797	1686.7448	1686.7407	0.0042	0	62	4.5e-06	1		■	■	■	■	■	■	■	■	■	R.IQDADYATEVSNMSK.A + Oxidation (M)
1210		845.4132	1688.8118	1688.8080	0.0038	0	7	0.2	1	U	■									K.ITFGSGMTVDFTQASK.K
1311		898.4828	1794.9510	1795.8873	-0.9363	1	2	0.7	1	U		■								K.LTTDAETKAATTADMLK.A + Oxidation (M)
1319	1	900.4835	1798.9524	1798.9789	-0.0264	1	46	4.4e-05	1	U					■				■	K.IQVGANDGQTISIDLkK.I
1363		927.6592	1853.3038	1851.9108	1.3930	1	5	0.57	1					■		■				K.NQSSMSTAIERLSSGLR.I + Oxidation (M)
1437	2	989.4578	1976.9010	1976.8963	0.0047	0	100	1e-10	1	U	■									K.SYSFDATALTNGDGTGATTK.V
1438		659.9752	1976.9038	1976.8963	0.0074	0	57	2e-06	1	U	■									K.SYSFDATALTNGDGTGATTK.V
1497		1043.0720	2084.1294	2084.1225	0.0069	0	101	4.8e-10	1		■	■	■		■	■			■	M.AQVINTNSLSLITQNNINK.N
1497		1043.0720	2084.1294	2085.0814	-0.9520	0	72	3.9e-07	4	U							■			M.AQVINTNSLSLNTQNNINK.N
1498		695.7179	2084.1319	2085.0814	-0.9495	0	73	3e-07	1	U							■			M.AQVINTNSLSLNTQNNINK.N
1498		695.7179	2084.1319	2084.1225	0.0093	0	72	4.5e-07	2		■	■	■		■	■			■	M.AQVINTNSLSLITQNNINK.N
1500		696.6981	2087.0725	2087.0634	0.0091	1	42	6.7e-05	1	U	■									K.SEVTTDPLKALDDAIASVDK.F
1501		1044.5440	2087.0734	2087.0634	0.0101	1	28	0.0018	1	U	■									K.SEVTTDPLKALDDAIASVDK.F
1535		1076.0220	2150.0294	2150.0240	0.0055	0	71	8.2e-08	1	U	■									K.AYAANGDNTAQISIGGSAQDVK.I
1536	1	717.6838	2150.0296	2150.0240	0.0056	0	30	0.0009	1	U	■									K.AYAANGDNTAQISIGGSAQDVK.I
1563		1110.7720	2219.5294	2219.1104	0.4191	1	0	4.6	1	U					■					K.DGTMKIQVGANDGQTISIDLK.K + Oxidation (M)
1587		750.3749	2248.1029	2248.0931	0.0098	0	81	4.5e-08	1		■	■	■	■	■		■	■		R.LDSAVTNLNNTTTNLSEAQSR.I
1588		1125.0590	2248.1034	2248.0931	0.0103	0	114	2.6e-11	1		■	■	■	■	■		■	■		R.LDSAVTNLNNTTTNLSEAQSR.I
1624		773.7389	2318.1949	2318.1866	0.0082	1	55	5.8e-06	1		■						■			R.LDEIDRVSGQTQFNGVNVLSK
1631		785.0120	2352.0142	2353.1471	-1.1330	0	4	0.44	1	U				■						K.DSLLSMLAPNAGDSFTASVSIGGK.A + Oxidation (M
1647		1235.1020	2468.1894	2468.1820	0.0075	0	61	8.8e-07	1	U	■									K.ATEADLTAAGFSQGAVVSGNSTWTK.S
1648		823.7372	2468.1898	2468.1820	0.0078	0	35	0.0003	1	U	■									K.ATEADLTAAGFSQGAVVSGNSTWTK.S
1653	2	837.4156	2509.2250	2509.2184	0.0066	0	46	2.3e-05	1	U	■									K.IASDGTLTDVNGDALYIGSDGNLTK.N
1658	2	1255.6220	2509.2294	2509.2184	0.0110	0	69	1.4e-07	1	U	■									K.IASDGTLTDVNGDALYIGSDGNLTK.N
1659		1258.1440	2514.2734	2514.2636	0.0099	0	19	0.012	1	U									■	K.MDASVLTDLNITDASAVSLHNVTK.G
1660		1265.1050	2528.1954	2528.1891	0.0063	0	85	3.5e-09	1	U	■									K.NQAGGPDAATLDGIFNGANGNAAVDAK.I
1661		843.7406	2528.2000	2528.1891	0.0108	0	113	5.3e-12	1	U	■									K.NQAGGPDAATLDGIFNGANGNAAVDAK.I
1672	1	1283.6240	2565.2334	2565.2294	0.0041	0	113	1.6e-11	1	U	■									R.ELTVQATTGTNSTSDLDSIQDEIK.S
1672	1	1283.6240	2565.2334	2565.2293	0.0041	0	67	6.7e-07	2	U					■					R.ELTVQATTGTNSESDLSSIQDEIK.S
1672	1	1283.6240	2565.2334	2565.1930	0.0405	0	39	0.00036	3			■	■							R.ELTVQASTGTNSDSDLDSIQDEIK.S
1673		856.0856	2565.2350	2565.2294	0.0056	0	39	0.00041	1	U	■									R.ELTVQATTGTNSTSDLDSIQDEIK.S
1673		856.0856	2565.2350	2565.1930	0.0420	0	29	0.004	2			■	■							R.ELTVQASTGTNSDSDLDSIQDEIK.S
1673		856.0856	2565.2350	2565.2293	0.0056	0	23	0.014	3	U					■					R.ELTVQATTGTNSESDLSSIQDEIK.S
1687		1315.1460	2628.2774	2628.2739	0.0035	0	131	3.7e-13	1		■	■	■	■		■		■		R.NANDGISVAQTTEGALSEINNNLQR.I
1688		877.1008	2628.2806	2628.2739	0.0067	0	70	4.1e-07	1		■	■	■	■		■		■		R.NANDGISVAQTTEGALSEINNNLQR.I
1698		1329.1260	2656.2374	2656.2293	0.0082	0	77	5.4e-08	1	U	■									K.TVTETYHEFANGNILDDDGAALYK.A
1699		665.0677	2656.2417	2656.2293	0.0124	0	35	0.0008	1	U	■									K.TVTETYHEFANGNILDDDGAALYK.A
1700	1	886.4213	2656.2421	2656.2293	0.0128	0	70	2.8e-07	1	U	■									K.TVTETYHEFANGNILDDDGAALYK.A
1709		893.4684	2677.3834	2677.3043	0.0791	1	15	0.034	1	U		■								K.GTITIDGSAQDVQISSDGKITASNGDK.L
1731		945.8143	2834.4211	2834.4145	0.0066	1	59	4.2e-06	1	U	■									R.IRELTVQATTGTNSTSDLDSIQDEIK.S
1731		945.8143	2834.4211	2834.3781	0.0429	1	20	0.032	2			■	■							R.IRELTVQASTGTNSDSDLDSIQDEIK.S
1741		994.2016	2979.5830	2978.4080	1.1750	0	7	0.22	1	U						■				K.GGVANAVATAADMTAAGFTLAGNTYTGSAGFK.A + Oxi
1754		1086.5780	3256.7122	3256.7011	0.0111	1	91	3.1e-09	1		■	■	■		■					M.AQVINTNSLSLITQNNINKNQSALSSSIER.L

60 subsets and intersections (161 subset proteins in total)

10 per page 1

Not what you expected? Try the select summary.

Mascot: http://www.matrixscience.com/
