# Supplementary material for: MS-H: A Novel Proteomic Approach to Isolate and Type the E. coli H Antigen Using Membrane Filtration and Liquid Chromatography-Tandem Mass Spectrometry (LC-MS/MS)
Source: PLoS One. 2013 Feb 21;8(2):e57339. doi: 10.1371/journal.pone.0057339 (PMC3578835; doi:10.1371/journal.pone.0057339)
Supplement: Representative Peptide Data S1 — Peptide data are represented as the Mascot search results from all 53 serotypes, obtained under the Orbitrap platform in Table 4 with related E. coli reference strains. “U” denotes a unique peptide specific for each of the proteins 1.1, 1.2, and beyond. The number 1.1 (shown as 1 in the peptide list and phylogenetic tree) represents the protein which obtained the highest score and confidence value after a Mascot search. This protein, known as the first hit, was used to designate the MS-H type of the unknown flagellin. Related peptides 1.2 (2), 1.3 (3), etc. represented the second, third, etc. hits for MS-H typing analysis. (DOCX) [file pone.0057339.s009.docx › H31-E199.pdf]

MASCOT Search Results

User :
E-mail :
Search title : Submitted from 20110811-0587-02 by Mascot Daemon on VARIABLE
MS data file : C:\Documents and Settings\keding\Desktop\Raw data\20110811-001-0031-00587\20110811-011-EC199MS1rp.RAW
Database : Flagellin_v2 (192 sequences; 89,845 residues)
Taxonomy : Bacteria (Eubacteria) (192 sequences)
Timestamp : 15 Aug 2011 at 15:21:58 GMT

Not what you expected? Try **the select summary**.

▶**Search parameters**

▶**Score distribution**

▶**Legend**

Protein Family Summary

Significance threshold p< 0.05 Max. number of families AUTO
Ions score or expect cut-off 0 Dendrograms cut at 0

Protein families 1–3 (out of 3)

10 per page 1

▼**1**

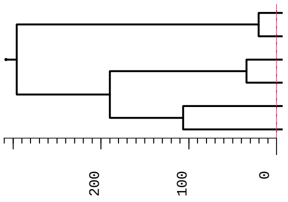

1	gi|30059898	1659	H31 0|H31|HW33 [gi|300818653|ref|ZP_07098861| Hxx 0|0|MS 107-1] [gi|30052...
3	gi|121144335	786	H55 0|O75:H55|E2987-73
2	gi|30059894	1234	H30 0|H30|HW32 [gi|188494683|ref|ZP_03001953| Hxx 0|0|53638] [gi|18848988...
6	gi|30577587	534	H17 0|O15:H17|P12b
5	gi|30059896	634	H29 0|H29|HW31
4	gi|6941886	759	H43 0|O157:H43|O157:H43 [gi|30059916|ref|AAP13330| H43 0|H43|149-51]

Threshold (**0**): 0

		Score	Mass	Matches	Sequences	emPAI
☑ 1.1	gi|30059898	1659	56551	49 (43)	34 (29)	9.19
	H31 0|H31|HW33 [gi|300818653|ref|ZP_07098861| Hxx 0|0|MS 107-1] [gi|300528825|ref|EFK49887| Hxx 0|0|MS 107-1]					
☑ 1.2	gi|30059894	1234	58102	34 (29)	23 (19)	3.95
	H30 0|H30|HW32 [gi|188494683|ref|ZP_03001953| Hxx 0|0|53638] [gi|188489882|ref|EDU64985| Hxx 0|0|53638]					
☑ 1.3	gi|121144335	786	62285	23 (19)	16 (12)	1.52
	H55 0|O75:H55|E2987-73					
☑ 1.4	gi|6941886	759	51071	21 (19)	14 (12)	2.08
	H43 0|O157:H43|O157:H43 [gi|30059916|ref|AAP13330| H43 0|H43|149-51]					
☑ 1.5	gi|30059896	634	45760	18 (17)	13 (12)	2.05
	H29 0|H29|HW31					
☑ 1.6	gi|30577587	534	36285	15 (13)	11 (9)	2.11
	H17 0|O15:H17|P12b					

▼**70 peptide matches (66 non-duplicate, 4 duplicate)**

Query	Dupes	Observed	Mr(expt)	Mr(calc)	Delta	M	Score	Expect	Rank	U	1	2	3	4	5	6	Peptide
37		358.7145	715.4144	715.3977	0.0168	0	0	1	2		■	■	■	■	■	■	K.GLTQAAR.N
67		380.6961	759.3776	759.3763	0.0014	0	29	0.0071	1		■	■	■		■	■	R.LDEIDR.V
110		409.7254	817.4362	818.4498	-1.0135	1	1	1.1	1	U	■						K.ITAKDGSK.L
191		448.2303	894.4460	894.4447	0.0014	0	14	0.042	1	U	■						K.AVYADATGK.L
238		466.2523	930.4900	930.4883	0.0018	0	56	1.1e-05	1		■	■	■	■	■	■	R.SSLGAVQNR.L
260	1	473.2494	944.4842	944.4815	0.0028	0	36	0.00074	1	U	■						K.AETTADPLK.A
312		494.7655	987.5164	988.5077	-0.9912	0	5	0.35	1	U		■					K.SEVTTDPLK.A
327		502.2625	1002.5104	1002.5094	0.0010	1	33	0.0029	1		■	■	■		■	■	K.SRLDEIDR.V
360		516.2733	1030.5320	1030.5295	0.0026	0	64	3.6e-07	1	U	■						K.QDVVLASDGK.I
465		551.2687	1100.5228	1100.5210	0.0018	0	52	6.5e-05	1		■	■	■	■	■	■	K.DDAAGQAIANR.F
551		581.3049	1160.5952	1160.5925	0.0028	0	67	2.5e-07	1	U	■						K.ALDEAISSIDK.F
590		596.3034	1190.5922	1190.5891	0.0032	0	45	0.00018	1		■	■		■			K.NQSALSSSIER.L
751		647.8104	1293.6062	1293.6049	0.0014	0	30	0.001	1	U	■						K.ADGTGLTTDNTTK.Y
834		672.8793	1343.7440	1343.7408	0.0032	0	34	0.00043	1	U			■				-.SLSLITQNNINK.N
938		720.9136	1439.8126	1439.8096	0.0030	0	94	1.6e-09	1		■	■	■				K.AQIIQQAGNSVLAK.A
939		480.9450	1439.8132	1439.8096	0.0036	0	35	0.0016	1		■	■	■				K.AQIIQQAGNSVLAK.A
941		482.2181	1443.6325	1443.7933	-0.1608	1	4	0.44	1	U	■						K.QDVVLASDGKITAK.D
971		488.9297	1463.7673	1463.7620	0.0053	1	44	5.1e-05	1	U	■						K.ALDEAISSIDKFR.S
972		732.8913	1463.7680	1463.7620	0.0061	1	70	1.5e-07	1	U	■						K.ALDEAISSIDKFR.S
1005		747.9200	1493.8254	1493.8202	0.0053	0	38	0.001	1		■	■		■	■	■	K.ANQVPQQVLSLLQG.-
1090	1	781.4222	1560.8298	1560.8260	0.0038	0	62	3.2e-06	1		■		■	■	■		R.VSGQTQFNGVNVLAK.D
1091		521.2844	1560.8314	1560.8260	0.0054	0	27	0.0095	1		■		■	■	■		R.VSGQTQFNGVNVLAK.D

Query	Dupes	Observed	Mr(expt)	Mr(calc)	Delta	M	Score	Expect	Rank	U	1	2	3	4	5	6	Peptide
1144	▶1	802.3657	1602.7168	1602.7162	0.0007	0	105	3.3e-11	▶1	U	■						K.YYLQDDGSVTNGSGK.A
1157		538.9458	1613.8156	1613.8121	0.0035	1	47	0.00016	▶1		■	■	■	■	■	■	R.INSAKDDAAGQAIANR.F
1158		807.9151	1613.8156	1613.8121	0.0035	1	58	1.4e-05	▶1		■	■	■	■	■	■	R.INSAKDDAAGQAIANR.F
1220		836.3818	1670.7490	1670.7457	0.0033	0	91	4.4e-09	▶1		■	■	■	■	■	■	R.IQDADYATEVSNMSK.A
1221		557.9239	1670.7499	1670.7457	0.0041	0	31	0.0054	▶1		■	■	■	■	■	■	R.IQDADYATEVSNMSK.A
1323		880.9404	1759.8662	1759.8629	0.0033	0	81	7.3e-09	▶1	U	■						K.ATGFTSGTYTVGTDGVVK.S
1365		900.4816	1798.9486	1798.9789	-0.0302	1	78	3.1e-08	▶1	U				■	■		K.IQVGANDGQTISIDLkK.I
1375		902.9615	1803.9084	1803.9102	-0.0017	1	76	1.4e-07	▶1	U	■						K.LTTDAETKAETTADPLK.A
1376		602.3107	1803.9103	1803.9102	0.0001	1	26	0.014	▶1	U	■						K.LTTDAETKAETTADPLK.A
1382		907.6427	1813.2708	1812.9945	0.2763	1	3	2.2	▶1	U	■						K.IQVGANDGQTITIDLKK.I
1382		907.6427	1813.2708	1813.9785	-0.7077	1	2	2.8	▶2			■	■				K.IQVGANDGETITIDLKK.I
1509		989.4576	1976.9006	1976.8963	0.0043	0	68	1.5e-07	▶1	U		■					K.SYSFDATALTNGDGTATTK.V
1546		1010.4980	2018.9814	2018.9769	0.0045	0	83	4.8e-09	▶1	U					■		K.NYVANDSLVNANGAAGAAATR.V
1548		1015.5640	2029.1134	2029.1055	0.0079	0	93	5.4e-10	▶1	U	■						K.TAGAINVTGAVISADALLSASK.A
1549		677.3785	2029.1137	2029.1055	0.0082	0	43	5.4e-05	▶1	U	■						K.TAGAINVTGAVISADALLSASK.A
1580		1043.0710	2084.1274	2084.1225	0.0049	0	107	1.4e-10	▶1		■	■		■	■		M.AQVINTNSLSLITQNNINK.N
1580		1043.0710	2084.1274	2085.0814	-0.9540	0	72	3.7e-07	▶4	U						■	M.AQVINTNSLSLNTQNNINK.N
1581		695.7169	2084.1289	2085.0814	-0.9525	0	67	1.4e-06	▶1	U						■	M.AQVINTNSLSLNTQNNINK.N
1581		695.7169	2084.1289	2084.1225	0.0063	0	61	5.6e-06	▶2		■	■		■	■		M.AQVINTNSLSLITQNNINK.N
1622		1076.0220	2150.0294	2150.0240	0.0055	0	15	0.032	▶1	U		■					K.AYAANGDNTAQISIGGSAQDVK.I
1664		743.6825	2228.0257	2228.0193	0.0064	1	38	0.00016	▶1	U	■						K.SGGNDVYNKADGTGLTTDNTTK.Y
1679		1125.0560	2248.0974	2248.0931	0.0043	0	118	8.5e-12	▶1		■	■	■	■		■	R.LDSAVTNLNNTTTNLSEAQSR.I
1680		750.3734	2248.0984	2248.0931	0.0053	0	87	1.1e-08	▶1		■	■	■	■		■	R.LDSAVTNLNNTTTNLSEAQSR.I
1700		768.4064	2302.1974	2302.1917	0.0056	1	33	0.0023	▶1		■		■		■		R.LDEIDRVSGQTQFNGVNVLAK.D
1742		1255.6190	2509.2234	2509.2184	0.0050	0	71	7.4e-08	▶1	U		■					K.IASDGTLTDVNGDALYIGSDGNLTK.N
1743	▶1	837.4159	2509.2259	2509.2184	0.0075	0	47	1.9e-05	▶1	U		■					K.IASDGTLTDVNGDALYIGSDGNLTK.N
1746		1265.1060	2528.1974	2528.1891	0.0083	0	24	0.004	▶1	U		■					K.NQAGGPDAATLDGIFNGANGNAAVDAK.I
1747		843.7401	2528.1985	2528.1891	0.0093	0	107	2.2e-11	▶1	U		■					K.NQAGGPDAATLDGIFNGANGNAAVDAK.I
1748		845.4050	2533.1932	2534.1197	-0.9265	0	0	0.93	▶1	U			■				K.YYAHTNGSVTNDSGSAIYATEADK.L
1758		1283.6250	2565.2354	2565.2294	0.0061	0	76	8.2e-08	▶1	U		■					R.ELTVQATTGTNSTSDLDSIQDEIK.S
1758		1283.6250	2565.2354	2565.2293	0.0061	0	37	0.00067	▶2	U				■			R.ELTVQATTGTNSESDLSSIQDEIK.S
1758		1283.6250	2565.2354	2565.1930	0.0425	0	24	0.012	▶3	U	■						R.ELTVQASTGTNSDSDLDSIQDEIK.S
1759		856.0858	2565.2356	2565.2294	0.0062	0	42	0.00017	▶1	U		■					R.ELTVQATTGTNSTSDLDSIQDEIK.S
1759		856.0858	2565.2356	2565.1930	0.0426	0	32	0.0018	▶2	U	■						R.ELTVQASTGTNSDSDLDSIQDEIK.S
1759		856.0858	2565.2356	2565.2293	0.0062	0	28	0.0051	▶3	U				■			R.ELTVQATTGTNSESDLSSIQDEIK.S
1761		860.1109	2577.3109	2577.3035	0.0074	1	57	1.9e-06	▶1	U	■						K.KIDSDTLGLSGFNVNGGGAVANTAATK.D
1772		1315.1460	2628.2774	2628.2739	0.0035	0	119	6.3e-12	▶1		■	■	■				R.NANDGISVAQTTEGALSEINNNLQR.I
1773		877.1000	2628.2782	2628.2739	0.0043	0	70	4.3e-07	▶1		■	■	■				R.NANDGISVAQTTEGALSEINNNLQR.I
1781		886.4193	2656.2361	2656.2293	0.0068	0	52	1.7e-05	▶1	U		■					K.TVTETYHEFANGNILDDDGAALYK.A
1782		1329.1260	2656.2374	2656.2293	0.0082	0	40	0.00025	▶1	U		■					K.TVTETYHEFANGNILDDDGAALYK.A
1813		1463.2040	2924.3934	2924.3828	0.0106	0	66	3.7e-07	▶1	U	■						K.FNQANNTFTYNTTSTAAELQSYLTPK.A
1814		975.8056	2924.3950	2924.3828	0.0122	0	27	0.0033	▶1	U	■						K.FNQANNTFTYNTTSTAAELQSYLTPK.A
1829		1054.5080	3160.5022	3160.5708	-0.0687	1	14	0.11	▶1		■	■	■	■		■	R.SSLGAVQNRLDSAVTNLNNTTTNLSEAQSR.I
1836		1086.5780	3256.7122	3256.7011	0.0111	1	29	0.0048	▶1		■	■		■			M.AQVINTNSLSLITQNNINKNQSALSSSIER.L

▶62 subsets and intersections (165 subset proteins in total)

▶2	gi|112820172	14	H21 0|EHEC serogroup: O113:H21|0
▶3	gi|9858183	13	H7 0|O157:H7|Y350-1

10 per page 1

Not what you expected? Try **the select summary**.

Mascot: http://www.matrixscience.com/
