# Supplementary material for: MS-H: A Novel Proteomic Approach to Isolate and Type the E. coli H Antigen Using Membrane Filtration and Liquid Chromatography-Tandem Mass Spectrometry (LC-MS/MS)
Source: PLoS One. 2013 Feb 21;8(2):e57339. doi: 10.1371/journal.pone.0057339 (PMC3578835; doi:10.1371/journal.pone.0057339)
Supplement: Representative Peptide Data S1 — Peptide data are represented as the Mascot search results from all 53 serotypes, obtained under the Orbitrap platform in Table 4 with related E. coli reference strains. “U” denotes a unique peptide specific for each of the proteins 1.1, 1.2, and beyond. The number 1.1 (shown as 1 in the peptide list and phylogenetic tree) represents the protein which obtained the highest score and confidence value after a Mascot search. This protein, known as the first hit, was used to designate the MS-H type of the unknown flagellin. Related peptides 1.2 (2), 1.3 (3), etc. represented the second, third, etc. hits for MS-H typing analysis. (DOCX) [file pone.0057339.s009.docx › H32-E200.pdf]

MATRIX SCIENCE MASCOT Search Results

User :
E-mail :
Search title : Submitted from 20110815-0595-02 by Mascot Daemon on VARIABLE
MS data file : C:\Documents and Settings\keding\Desktop\Raw data\20110815-001-0031-00595\20110815-010-EC200MS3.RAW
Database : Flagellin_v2 (192 sequences; 89,845 residues)
Taxonomy : Bacteria (Eubacteria) (192 sequences)
Timestamp : 18 Aug 2011 at 19:00:50 GMT

Not what you expected? Try **the select summary**.

Search parameters

Score distribution

Legend

Protein Family Summary

Significance threshold p< 0.05 Max. number of families AUTO
Ions score or expect cut-off 0 Dendrograms cut at 0

Protein family 1 (out of 1)

10 per page 1

1

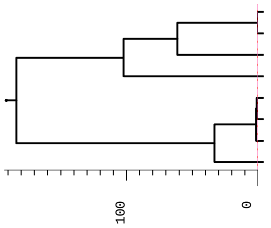

1	gi|12697206	2274	H32 0|serotype O114:H32|K10 [gi|30059900|ref|AAP13322| H32 0|H32|HW34]
7	gi|30059910	542	H39 0|H39|E3a
4	gi|6009849	1070	H49 0|H49|U11a-44
5	gi|30059928	1049	H52 0|H52|C2187-69
2	gi|30059898	1309	H31 0|H31|HW33 [gi|300818653|ref|ZP_07098861| Hxx 0|0|MS 107-1] [gi|300528825|ref|EFK4...
6	gi|125659369	1027	H44 0|H44|781-55
3	gi|6941882	1136	H14 0|O138:H14|O138:H14
8	gi|33590243	525	H16 NM|O157:NM(H16)|PC02

Threshold (**0**): 0

		Score	Mass	Matches	Sequences	emPAI
1.1	gi|12697206	2274	58154	54 (43)	32 (30)	6.66
	H32 0|serotype O114:H32|K10 [gi|30059900|ref|AAP13322| H32 0|H32|HW34]					
1.2	gi|30059898	1309	56551	35 (25)	22 (18)	2.68
	H31 0|H31|HW33 [gi|300818653|ref|ZP_07098861| Hxx 0|0|MS 107-1] [gi|300528825|ref|EFK49887| Hxx 0|0|MS 107-1]					
1.3	gi|6941882	1136	56492	40 (23)	26 (17)	2.28
	H14 0|O138:H14|O138:H14					
1.4	gi|6009849	1070	58493	32 (21)	21 (16)	1.99
	H49 0|H49|U11a-44					
1.5	gi|30059928	1049	46003	30 (20)	20 (15)	2.74
	H52 0|H52|C2187-69					
1.6	gi|125659369	1027	58912	31 (21)	19 (15)	1.81
	H44 0|H44|781-55					
1.7	gi|30059910	542	44239	25 (14)	17 (11)	1.55
	H39 0|H39|E3a					
1.8	gi|33590243	525	55093	24 (11)	15 (8)	0.79
	H16 NM|O157:NM(H16)|PC02					

2 samesets of gi|33590243

93 peptide matches (72 non-duplicate, 21 duplicate)

Query	Dupes	Observed	Mr(expt)	Mr(calc)	Delta	M	Score	Expect	Rank	U	1	2	3	4	5	6	7	8	Peptide
28	1	315.6945	629.3744	629.3860	-0.0116	1	7	0.22	1	U								■	K.VDKLR.S
34	1	316.6898	631.3650	631.3653	-0.0003	0	30	0.0089	1		■	■	■	■	■	■	■	■	R.LSSGLR.I
41	1	322.7024	643.3902	643.3905	-0.0002	0	34	0.00042	1	U	■								K.VADVLK.A
93		352.2027	702.3908	702.3912	-0.0003	0	2	0.99	1	U							■		K.AIASVDK.F
101		355.1975	708.3804	708.3806	-0.0002	0	19	0.077	1		■	■	■	■	■	■	■		R.FTSNIK.G
103		358.7060	715.3974	715.3977	-0.0002	0	31	0.0061	1		■	■	■	■	■	■	■		K.GLTQAAR.N
135	1	380.2029	758.3912	758.4174	-0.0261	0	33	0.0031	1	U								■	K.LDEALAK.V
139		380.6950	759.3754	759.3763	-0.0008	0	36	0.0014	1		■	■	■	■	■	■	■		R.LDEIDR.V
145		382.2133	762.4120	762.4123	-0.0003	0	32	0.00067	1	U	■								K.IDSSTLK.L
229		409.7238	817.4330	818.4498	-1.0167	1	6	0.39	2			■				■			K.ITAKDGSK.L
275		423.2213	844.4280	844.4402	-0.0122	0	23	0.0052	1	U				■					K.AAAGAESIR.Y
280	2	424.2551	846.4956	846.4447	0.0510	0	15	0.041	1	U			■						K.AATTADGLK.A
336		439.8229	877.6312	876.4552	1.1760	0	1	0.79	1	U			■						K.AATTADSLK.A
345		446.2603	890.5060	890.5073	-0.0012	1	27	0.006	1	U	■								K.KIDSSTLK.L
348		447.7188	893.4230	892.4290	0.9940	0	0	0.97	1	U			■						K.VDQAAFDK.A
403		464.8065	927.5984	926.4821	1.1163	0	4	0.42	1	U			■						K.AATTADHLK.A
409	2	466.2510	930.4874	930.4883	-0.0008	0	58	6.4e-06	1		■	■	■		■	■	■	■	R.SSLGAVQNR
445	1	473.2526	944.4906	944.5039	-0.0133	0	8	0.49	1	U				■					R.SSLGAIQNR.L
474	1	481.2447	960.4748	960.4764	-0.0015	0	42	6e-05	1	U	■								K.SEATTDPLK.A
515		489.6763	977.3380	978.5134	-1.1754	0	1	0.76	1	U					■				K.GFSVSGNALK.V

Query	Dupes	Observed	Mr(expt)	Mr(calc)	Delta	M	Score	Expect	Rank	U	1	2	3	4	5	6	7	8	Peptide
519	1	490.2634	978.5122	978.5134	-0.0012	0	38	0.00018	1	U	■								K.LTSFNVNGK.G
541		495.2662	988.5178	989.5029	-0.9851	0	33	0.00049	1	U								■	K.GAELSASDLK.A
576	2	502.2613	1002.5080	1002.5094	-0.0014	1	38	0.001	1		■	■	■	■	■	■	■		K.SRLDEIDR.V
577		335.1768	1002.5086	1002.5094	-0.0008	1	25	0.018	1		■	■	■	■	■	■	■		K.SRLDEIDR.V
600	1	508.2618	1014.5090	1014.5709	-0.0619	0	9	0.13	1	U								■	K.ALATTNPLSK.L
605		508.7899	1015.5652	1016.5502	-0.9850	0	13	0.048	1	U							■		K.NVDLSAVATK.L
625		513.7151	1025.4156	1024.5302	0.8855	0	14	0.039	1	U						■			K.AGDTAHLNVK.I
749	1	551.2673	1100.5200	1100.5210	-0.0010	0	76	2.2e-07	1		■	■	■	■	■	■	■	■	K.DDAAGQAIANR.F
788	1	559.2897	1116.5648	1116.5663	-0.0014	0	80	9e-09	1	U	■								K.ALDDAIASVDK.F
933		596.3012	1190.5878	1190.5891	-0.0012	0	58	8.3e-06	1		■	■	■	■	■				K.NQSALSSSIER.L
938		598.8008	1195.5870	1194.5517	1.0354	0	3	0.48	1	U				■					K.DAAQSSIDFGGK.K
947		600.8525	1199.6904	1199.6734	0.0170	1	11	0.081	1	U								■	K.LRSSLGAVQNR.F
990		407.5507	1219.6303	1220.6150	-0.9847	0	7	0.22	1	U								■	R.VSNQTQFNGVK.V
993		611.3015	1220.5884	1220.5885	-0.0000	0	64	4.2e-07	1	U	■								K.AADGSLTTEATGK.S
1017		413.5892	1237.7458	1237.5761	0.1697	0	2	0.7	1	U					■				K.FGANDTAAAAMAK.T
1316	1	710.8744	1419.7342	1419.7358	-0.0015	1	88	1.4e-09	1	U	■								K.ALDDAIASVDKFR.S
1317		474.2524	1419.7354	1419.7358	-0.0004	1	62	7e-07	1	U	■								K.ALDDAIASVDKFR.S
1342		720.9116	1439.8086	1439.8096	-0.0010	0	96	1.1e-09	1		■	■	■	■		■			K.AQIIQQAGNSVLAK.A
1343		480.9435	1439.8087	1439.8096	-0.0009	0	1	3.9	1		■	■	■	■		■			K.AQIIQQAGNSVLAK.A
1360		728.9080	1455.8014	1455.8045	-0.0031	0	77	3.1e-08	1	U					■				K.AQIIQQAGNSVLSK.A
1406		747.9219	1493.8292	1493.8202	0.0091	0	67	1.2e-06	1		■	■	■	■	■	■	■		K.ANQVPQQVLSLLQG.-
1500		781.1614	1560.3082	1560.8260	-0.5178	0	4	1.7	1			■	■	■	■	■	■		R.VSGQTQFNGVNVLAK
1579		538.9440	1613.8102	1613.8121	-0.0019	1	34	0.0038	1		■	■	■	■	■	■	■	■	R.INSAKDDAAGQAIANR.F
1653		836.3792	1670.7438	1670.7457	-0.0019	0	128	1e-12	1		■	■	■	■	■	■	■		R.IQDADYATEVSNMSK.A
1679	1	843.9489	1685.8832	1685.8836	-0.0003	0	115	3e-11	1	U	■								K.IQVGANDGETITIDLK.K
1679	1	843.9489	1685.8832	1684.8996	0.9837	0	64	4.3e-06	2			■	■	■		■			K.IQVGANDGQTITIDLK.K
1721		860.3571	1718.6996	1718.7974	-0.0977	0	5	0.32	1	U								■	K.ALAYNDAPMSVYFGGK.N + Oxidation (M)
1755		584.9456	1751.8150	1751.8611	-0.0461	1	5	0.3	2	U			■						K.LTTDAETKAATTADCLK.A
1764		880.9377	1759.8608	1759.8629	-0.0021	0	54	4e-06	1	U		■							K.ATGFTSGTYTVGTDGVVK.S
1806		359.9966	1794.9466	1795.8873	-0.9407	1	2	0.67	2	U			■						K.LTTDAETKAATTADMLK.A + Oxidation (M)
1943		647.6498	1939.9276	1938.9898	0.9377	0	1	0.77	1	U							■		K.IDSSTLGLSGFSVSQNSLK.L
1962		982.4459	1962.8772	1962.8807	-0.0034	0	113	5.1e-12	1	U	■								K.SYSFDATALTNGDGTAGSTK.V
2006		677.3755	2029.1047	2029.1055	-0.0008	0	56	2.6e-06	1	U		■							K.TAGAINVTGAVISADALLSASK.A
2007		1015.5610	2029.1074	2029.1055	0.0019	0	104	3.7e-11	1	U		■							K.TAGAINVTGAVISADALLSASK.A
2037		1043.0680	2084.1214	2084.1225	-0.0011	0	134	2.5e-13	1		■	■	■	■	■	■	■	■	M.AQVINTNSLSLiTQNNiNK.N
2038		695.7147	2084.1223	2084.1225	-0.0003	0	78	1e-07	1		■	■	■	■	■	■	■	■	M.AQVINTNSLSLiTQNNiNK.N
2083		1083.0260	2164.0374	2164.0396	-0.0022	0	95	3.5e-10	1	U	■								K.AYAANGDNTAQISIGGSAQEVK.I
2101		739.7235	2216.1487	2215.1630	0.9856	0	0	6	1		■	■	■	■	■	■	■	■	-.MAQVINTNSLSLiTQNNiNK.N
2129		1125.0530	2248.0914	2248.0931	-0.0017	0	137	1.1e-13	1		■	■	■	■	■	■			R.LDSAVTNLNNTTTNLSEAQSR.I
2130		750.3715	2248.0927	2248.0931	-0.0004	0	85	2e-08	1		■	■	■	■	■	■			R.LDSAVTNLNNTTTNLSEAQSR.I
2142		1137.0580	2272.1014	2272.1046	-0.0031	0	91	8.6e-10	1	U	■								K.ITFGSGMTVDFTQVSNNVDIK.G
2147		761.7320	2282.1742	2283.1053	-0.9311	1	0	0.95	1	U								■	R.VTAFVEDNGSATSVDLAAGKMGK.A + Oxidation (M)
2207		1250.1020	2498.1894	2498.1925	-0.0031	0	137	2e-14	1	U	■								K.ATEADLTAAGFSQSAVVSGNSTWTK.S
2208		833.7377	2498.1913	2498.1925	-0.0012	0	114	4.3e-12	1	U	■								K.ATEADLTAAGFSQSAVVSGNSTWTK.S
2215	1	851.4086	2551.2040	2551.2051	-0.0012	0	65	4.7e-07	1	U	■								K.NQAGGPAAATLDGIFNGANGHDAVDAK.I
2223		877.0978	2628.2716	2628.2739	-0.0023	0	86	1.1e-08	1		■	■	■	■	■	■		■	R.NANDGISVAQTTEGALSEINNNLQR.I
2224		1315.1440	2628.2734	2628.2739	-0.0005	0	106	1.2e-10	1		■	■	■	■	■	■		■	R.NANDGISVAQTTEGALSEINNNLQR.I
2228		1329.1200	2656.2254	2656.2293	-0.0038	0	89	3.7e-09	1	U	■								K.TVTETYHEFANGNILDDDGAALYK.A
2229	1	886.4164	2656.2274	2656.2293	-0.0019	0	60	2.6e-06	1	U	■								K.TVTETYHEFANGNILDDDGAALYK.A
2237		893.4645	2677.3717	2677.3043	0.0674	1	9	0.11	1	U			■						K.GTITIDGSAQDVQISSDGKITASNGDK.L
2285		1086.5740	3256.7002	3256.7011	-0.0009	1	89	4.9e-09	1		■	■	■	■	■				M.AQVINTNSLSLITQNNINKNQSALSSSIER.L
2289		1103.9170	3308.7292	3309.6661	-0.9369	1	0	0.92	1	U							■		K.GLTQAARNANDGISLAQTAEGALSEINNNLQR.V

55 subsets and intersections (161 subset proteins in total)

10 per page 1

Not what you expected? Try **the select summary**.

Mascot: http://www.matrixscience.com/
