# Supplementary material for: MS-H: A Novel Proteomic Approach to Isolate and Type the E. coli H Antigen Using Membrane Filtration and Liquid Chromatography-Tandem Mass Spectrometry (LC-MS/MS)
Source: PLoS One. 2013 Feb 21;8(2):e57339. doi: 10.1371/journal.pone.0057339 (PMC3578835; doi:10.1371/journal.pone.0057339)
Supplement: Representative Peptide Data S1 — Peptide data are represented as the Mascot search results from all 53 serotypes, obtained under the Orbitrap platform in Table 4 with related E. coli reference strains. “U” denotes a unique peptide specific for each of the proteins 1.1, 1.2, and beyond. The number 1.1 (shown as 1 in the peptide list and phylogenetic tree) represents the protein which obtained the highest score and confidence value after a Mascot search. This protein, known as the first hit, was used to designate the MS-H type of the unknown flagellin. Related peptides 1.2 (2), 1.3 (3), etc. represented the second, third, etc. hits for MS-H typing analysis. (DOCX) [file pone.0057339.s009.docx › H33-E201.pdf]

# MASCOT Search Results

**User** :
**E-mail** :
**Search title** : Submitted from 20110815-0595-02 by Mascot Daemon on VARIABLE
**MS data file** : C:\Documents and Settings\keding\Desktop\Raw data\20110815-001-0031-00595\20110815-011-EC201MS3.RAW
**Database** : Flagellin_v2 (192 sequences; 89,845 residues)
**Taxonomy** : Bacteria (Eubacteria) (192 sequences)
**Timestamp** : 18 Aug 2011 at 19:03:21 GMT

Not what you expected? Try **the select summary**.

▶ **Search parameters**

▶ **Score distribution**

▶ **Legend**

## Protein Family Summary

Significance threshold p< 0.05 Max. number of families AUTO
Ions score or expect cut-off 0 Dendrograms cut at 0

## Protein families 1–2 (out of 2)

10 per page 1

▼**1**

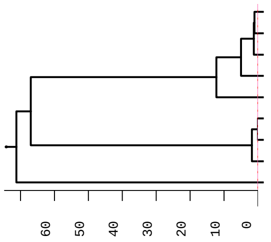

| | | | |
|---|---|---|---|
| 1 | **gi\|30059902** | 2072 | H33 0\|H33\|HW35 |
| 4 | **gi\|157081065** | 595 | Hxx(H28 99.7%) 0\|0\|E24377A [gi\|157159035\|ref\|YP_001463228\| Hxx 0\|0\|E24377A] |
| 2 | **gi\|30059910** | 1156 | H39 0\|H39\|E3a |
| 3 | **gi\|88683097** | 763 | H44 0\|H44\|0 |
| 5 | **gi\|6009849** | 515 | H49 0\|H49\|U11a-44 |
| 7 | **gi\|33590243** | 378 | H16 NM\|O157:NM(H16)\|PC02 |
| 9 | **gi\|16901492** | 275 | H8 0\|H8\|0 |
| 8 | **gi\|154710444** | 368 | H35 0\|H35\|0 |
| 6 | **gi\|154710451** | 392 | H36 0\|H36\|0 |

Threshold (**0**): 0

| | | Score | Mass | Matches | Sequences | emPAI |
|---|---|---|---|---|---|---|
| **1.1** | **gi\|30059902**<br>H33 0\|H33\|HW35 | 2072 | 43893 | 37 (32) | 26 (25) | 8.50 |
| **1.2** | **gi\|30059910**<br>H39 0\|H39\|E3a | 1156 | 44239 | 28 (23) | 19 (18) | 3.87 |
| **1.3** | **gi\|88683097**<br>H44 0\|H44\|0 | 763 | 55289 | 25 (17) | 17 (13) | 1.52 |
| **1.4** | **gi\|157081065**<br>Hxx(H28 99.7%) 0\|0\|E24377A [gi\|157159035\|ref\|YP_001463228\| Hxx 0\|0\|E24377A]<br>▶ **3 samesets of gi\|157081065** | 595 | 59373 | 22 (15) | 15 (12) | 1.13 |
| **1.5** | **gi\|6009849**<br>H49 0\|H49\|U11a-44 | 515 | 58493 | 21 (13) | 16 (11) | 1.04 |
| **1.6** | **gi\|154710451**<br>H36 0\|H36\|0 | 392 | 57784 | 17 (8) | 14 (6) | 0.56 |
| **1.7** | **gi\|33590243**<br>H16 NM\|O157:NM(H16)\|PC02<br>▶ **2 samesets of gi\|33590243** | 378 | 55093 | 20 (9) | 15 (7) | 0.59 |
| **1.8** | **gi\|154710444**<br>H35 0\|H35\|0 | 368 | 52714 | 16 (9) | 11 (7) | 0.62 |
| **1.9** | **gi\|16901492**<br>H8 0\|H8\|0<br>▶ **3 samesets of gi\|16901492** | 275 | 52025 | 17 (7) | 14 (6) | 0.54 |

▼**80 peptide matches (73 non-duplicate, 7 duplicate)**

| Query | Dupes | Observed | Mr(expt) | Mr(calc) | Delta | M | Score | Expect | Rank | U | 1 | 2 | 3 | 4 | 5 | 6 | 7 | 8 | 9 | Peptide |
|---|---|---|---|---|---|---|---|---|---|---|---|---|---|---|---|---|---|---|---|---|
| 36 | | 315.6997 | 629.3848 | 629.3860 | -0.0012 | 1 | 3 | 0.47 | ▶1 | U | | | | | | | ■ | | | K.VDKLR.S |
| 44 | ▶1 | **316.6897** | **631.3648** | **631.3653** | **-0.0005** | **0** | **28** | **0.017** | ▶1 | | ■ | ■ | ■ | ■ | ■ | ■ | ■ | ■ | ■ | **R.LSSGLR.I** |
| 78 | | 331.6804 | 661.3462 | 660.3806 | 0.9656 | 0 | 1 | 0.82 | ▶1 | U | | | | | | | | | ■ | K.TGAVSVK.T |
| 114 | | 347.6845 | 693.3544 | 692.3493 | 1.0051 | 0 | 12 | 0.14 | ▶1 | U | | | | | | ■ | | | | K.AGDVFGK.M |
| 128 | | **352.2025** | **702.3904** | **702.3912** | **-0.0007** | **0** | **35** | **0.00045** | ▶1 | | ■ | ■ | | | | | | | | **K.AIASVDK.F** |
| 137 | | 355.1969 | 708.3792 | 708.3806 | -0.0014 | 0 | 9 | 0.35 | ▶1 | | ■ | ■ | ■ | ■ | ■ | ■ | | ■ | | R.FTSNIK.G |
| 143 | ▶1 | **358.7057** | **715.3968** | **715.3977** | **-0.0008** | **0** | **33** | **0.0039** | ▶1 | | ■ | ■ | ■ | ■ | ■ | | | | | **K.GLTQAAR.N** |
| 181 | | **380.2029** | **758.3912** | **758.4174** | **-0.0261** | **0** | **31** | **0.0046** | ▶1 | **U** | | | | | | | ■ | | | **K.LDEALAK.V** |
| 181 | | 380.2029 | 758.3912 | 757.4698 | 0.9215 | 1 | 5 | 1.7 | ▶2 | U | | | | | | ■ | | | | K.LDKALAK.V |
| 183 | | **380.6949** | **759.3752** | **759.3763** | **-0.0010** | **0** | **26** | **0.015** | ▶1 | | ■ | ■ | ■ | ■ | ■ | | | | | **R.LDEIDR.V** |
| 259 | ▶1 | 409.7236 | 817.4326 | 816.4090 | 1.0237 | 0 | 2 | 0.84 | ▶2 | U | | | | | | | | | ■ | K.GTTTPGQR.D |
| 305 | | **423.2217** | **844.4288** | **844.4402** | **-0.0114** | **0** | **25** | **0.003** | ▶1 | **U** | | | | | ■ | | | | | **K.AAAGAESIR.Y** |
| 332 | | 430.7084 | 859.4022 | 860.4967 | -1.0945 | 1 | 1 | 0.86 | ▶1 | U | | | | | | | | | ■ | K.EVTTVKGK.G |
| 334 | | 430.7215 | 859.4284 | 860.4240 | -0.9955 | 0 | 3 | 0.55 | ▶1 | U | | | | | | | | | ■ | K.VELGGSDGK.T |
| 388 | | **452.2605** | **902.5064** | **902.5073** | **-0.0008** | **0** | **14** | **0.042** | ▶1 | **U** | | | | ■ | | | | | | **K.AATLDALTK.N** |

| Query | Dupes | Observed | Mr(expt) | Mr(calc) | Delta | M | Score | Expect | Rank | U | 1 | 2 | 3 | 4 | 5 | 6 | 7 | 8 | 9 | Peptide |
|---|---|---|---|---|---|---|---|---|---|---|---|---|---|---|---|---|---|---|---|---|
| 439 | 2 | 466.2507 | 930.4868 | 930.4883 | -0.0014 | 0 | 68 | 7.5e-07 | 1 | | ■ | ■ | ■ | ■ | | | ■ | ■ | | R.SSLGAVQNR |
| 459 | | 469.3183 | 936.6220 | 937.4505 | -0.8285 | 0 | 3 | 0.51 | 1 | U | | | | | | ■ | | | | K.GFISTDNGK.T |
| 475 | | 473.2529 | 944.4912 | 944.4927 | -0.0014 | 0 | 83 | 1.5e-08 | 1 | U | ■ | | | | | | | | | K.ADAGALVNSK.N |
| 477 | | 473.2788 | 944.5430 | 944.5039 | 0.0391 | 0 | 2 | 1.8 | 1 | U | | | | | ■ | | | | | R.SSLGAIQNR.L |
| 544 | | 487.7605 | 973.5064 | 973.5444 | -0.0379 | 1 | 16 | 0.036 | 1 | U | | | | | | ■ | | | | K.ISAEDLKAK.A |
| 579 | | 330.1733 | 987.4981 | 987.5349 | -0.0368 | 0 | 0 | 0.99 | 1 | U | | | | | | | | | ■ | K.ALSQVDSLR.S |
| 616 | 1 | 502.2611 | 1002.5076 | 1002.5094 | -0.0018 | 1 | 36 | 0.0015 | 1 | | ■ | ■ | ■ | ■ | ■ | | | | | K.SRLDEIDR.V |
| 617 | | 335.1766 | 1002.5080 | 1002.5094 | -0.0014 | 1 | 32 | 0.004 | 1 | | ■ | ■ | ■ | ■ | ■ | | | | | K.SRLDEIDR.V |
| 624 | | 503.7867 | 1005.5588 | 1005.5607 | -0.0019 | 1 | 62 | 6.7e-07 | 1 | | ■ | ■ | | | | | | | | K.AIASVDKFR.S |
| 625 | | 336.1939 | 1005.5599 | 1005.5607 | -0.0008 | 1 | 20 | 0.0095 | 1 | | ■ | ■ | | | | | | | | K.AIASVDKFR.S |
| 643 | | 508.2614 | 1014.5082 | 1014.5709 | -0.0627 | 0 | 1 | 0.86 | 2 | U | | | | | | | ■ | | | K.ALATTNPLSK.L |
| 650 | | 508.7896 | 1015.5646 | 1016.5502 | -0.9856 | 0 | 14 | 0.037 | 1 | U | | ■ | | | | | | | | K.NVDLSAVATK.L |
| 688 | | 346.1815 | 1035.5227 | 1035.4985 | 0.0242 | 0 | 1 | 0.88 | 2 | U | | | | | | | | | ■ | K.QYAANVDQK.T |
| 746 | | 358.2025 | 1071.5857 | 1071.5448 | 0.0409 | 0 | 0 | 0.92 | 1 | U | | | | | | | | | ■ | K.TEDPLAAIDK.A |
| 747 | 1 | 358.5182 | 1072.5328 | 1073.5651 | -1.0324 | 1 | 15 | 0.033 | 1 | U | | | | | | | | ■ | | R.VMAANDIKGR.T |
| 800 | | 549.2806 | 1096.5466 | 1096.5401 | 0.0066 | 0 | 13 | 0.05 | 1 | U | | | | | | | | | ■ | K.TYSVSDLQGK.S |
| 813 | | 551.2671 | 1100.5196 | 1100.5210 | -0.0014 | 0 | 69 | 1.1e-06 | 1 | | ■ | ■ | ■ | ■ | ■ | ■ | ■ | ■ | ■ | K.DDAAGQAIANR.F |
| 861 | | 376.8752 | 1127.6038 | 1128.6503 | -1.0465 | 1 | 5 | 0.31 | 1 | U | | | | | | | | ■ | | K.TLGLDKLDVR.N |
| 868 | | 378.5181 | 1132.5325 | 1133.5564 | -1.0240 | 1 | 6 | 0.23 | 1 | U | | | | | | ■ | | | | K.DADGKITTDAK.T |
| 911 | | 385.2108 | 1152.6106 | 1151.5822 | 1.0283 | 0 | 0 | 0.99 | 2 | U | | | | | | ■ | | | | K.ATLNGSEAYVK.G |
| 976 | | 397.8665 | 1190.5777 | 1190.5891 | -0.0114 | 0 | 2 | 3.1 | 2 | | | | | ■ | ■ | | | | | K.NQSALSSSIER.L |
| 983 | | 598.8005 | 1195.5864 | 1194.5517 | 1.0348 | 0 | 3 | 0.48 | 1 | U | | | | | ■ | | | | | K.DAAQSSIDFGGK.K |
| 990 | | 600.8530 | 1199.6914 | 1199.6734 | 0.0180 | 1 | 11 | 0.074 | 1 | U | | | | | | | ■ | | | K.LRSSLGAVQNR.F |
| 999 | | 603.3088 | 1204.6030 | 1204.6048 | -0.0017 | 0 | 58 | 3e-06 | 1 | | ■ | ■ | | | | | | | | K.NQSALSTSIER.L |
| 1017 | | 406.2026 | 1215.5860 | 1216.6663 | -1.0803 | 1 | 7 | 0.18 | 1 | U | | | | | | | | ■ | | K.EINSKTLGLDK.L |
| 1024 | | 407.5511 | 1219.6315 | 1220.6150 | -0.9835 | 0 | 2 | 0.7 | 1 | | | | | | | | ■ | ■ | | R.VSNQTQFNGVK.V |
| 1210 | | 442.2686 | 1323.7840 | 1322.6466 | 1.1373 | 1 | 5 | 0.3 | 1 | U | | | | | ■ | | | | | K.DAAQSSIDFGGKK.Y |
| 1386 | | 728.9080 | 1455.8014 | 1455.8045 | -0.0031 | 0 | 121 | 1.3e-12 | 1 | U | ■ | | | | | | | | | K.AQIIQQAGNSVLSK.A |
| 1400 | | 490.2570 | 1467.7492 | 1467.7682 | -0.0190 | 0 | 2 | 0.65 | 1 | U | | | ■ | | | | | | | K.ANQVPQQVLSLSQG.- |
| 1435 | | 747.9178 | 1493.8210 | 1493.8202 | 0.0009 | 0 | 57 | 1.2e-05 | 1 | U | ■ | ■ | ■ | ■ | ■ | | | | | K.ANQVPQQVLSLxQG.- |
| 1481 | | 506.9332 | 1517.7778 | 1517.7950 | -0.0173 | 0 | 18 | 0.016 | 1 | U | | | ■ | | | | | | | K.ANQVPQQVLSLHQG.- |
| 1497 | | 510.6994 | 1529.0764 | 1527.7715 | 1.3048 | 0 | 7 | 0.22 | 1 | U | | | ■ | | | | | | | K.ANQVPQQVLSLMQG.- + Oxidation (M) |
| 1547 | | 781.6654 | 1561.3162 | 1560.8260 | 0.4902 | 0 | 70 | 4.9e-07 | 1 | | ■ | ■ | ■ | ■ | ■ | | | | | R.VSGQTQFNGVNVLAK |
| 1631 | | 538.9441 | 1613.8105 | 1613.8121 | -0.0016 | 1 | 42 | 0.00058 | 1 | | ■ | ■ | ■ | ■ | ■ | ■ | ■ | ■ | ■ | R.INSAKDDAAGQAIANR.F |
| 1717 | | 836.3788 | 1670.7430 | 1670.7457 | -0.0027 | 0 | 122 | 4.2e-12 | 1 | | ■ | ■ | ■ | ■ | ■ | | | | | R.IQDADYATEVSNMSK.A |
| 1795 | | 573.4125 | 1717.2157 | 1715.7308 | 1.4849 | 0 | 1 | 1.6 | 1 | | | | | | | ■ | ■ | | ■ | R.IEDADYATEVSNMSR.A + Oxidation (M) |
| 1797 | | 860.3568 | 1718.6990 | 1718.7974 | -0.0983 | 0 | 1 | 0.75 | 1 | U | | | | | | | ■ | | | K.ALAYNDAPMSVYFGGK.N + Oxidation (M) |
| 1798 | | 574.0725 | 1719.1957 | 1718.7974 | 0.3983 | 0 | 1 | 0.75 | 2 | U | | | | | | | ■ | | | K.ALAYNDAPMSVYFGGK.N + Oxidation (M) |
| 1813 | | 865.9364 | 1729.8582 | 1729.8595 | -0.0012 | 0 | 156 | 2.5e-16 | 1 | U | ■ | | | | | | | | | K.NAAGSQNVTSAIGDIANK.A |
| 1835 | | 874.4559 | 1746.8972 | 1746.9000 | -0.0027 | 0 | 142 | 6.7e-15 | 1 | U | ■ | | | | | | | | | K.LSDSITQVGASGSLADVK.L |
| 1901 | | 359.9964 | 1794.9456 | 1795.8589 | -0.9132 | 0 | 6 | 0.25 | 1 | U | | | | ■ | | | | | | K.STGFTVDVGATGNSAGDIK.V |
| 1977 | | 618.0994 | 1851.2764 | 1851.9108 | -0.6345 | 1 | 1 | 0.77 | 1 | U | | | ■ | | | | | | | K.NQSSMSTAIERLSSGLR.I + Oxidation (M) |
| 2010 | | 940.4912 | 1878.9678 | 1878.9687 | -0.0009 | 0 | 108 | 1.8e-11 | 1 | U | ■ | | | | | | | | | K.IDSSTLGLGGFSVSNNALK.L |
| 2116 | | 499.4687 | 1993.8457 | 1992.9865 | 0.8592 | 0 | 23 | 0.012 | 1 | | | | | | | | ■ | ■ | ■ | R.FDSAITNLGNTVNNLSSAR.S |
| 2144 | | 1018.0200 | 2034.0254 | 2034.0269 | -0.0015 | 0 | 99 | 1.2e-10 | 1 | U | ■ | | | | | | | | | K.ANANIYTGTSSADPLALLDK.A |
| 2161 | | 1033.0390 | 2064.0634 | 2064.0640 | -0.0006 | 0 | 109 | 1.3e-11 | 1 | U | ■ | | | | | | | | | K.VGADALGAAVGYVTVQGQNFK.A |
| 2162 | | 689.0285 | 2064.0637 | 2064.0640 | -0.0004 | 0 | 88 | 1.5e-09 | 1 | U | ■ | | | | | | | | | K.VGADALGAAVGYVTVQGQNFK.A |
| 2172 | | 1043.0680 | 2084.1214 | 2084.1225 | -0.0011 | 0 | 142 | 4.6e-14 | 1 | | ■ | ■ | ■ | ■ | ■ | ■ | ■ | ■ | ■ | M.AQVINTNSLSLiTQNNiNK.N |
| 2173 | | 695.7148 | 2084.1226 | 2084.1225 | 0.0000 | 0 | 76 | 1.5e-07 | 1 | | ■ | ■ | ■ | ■ | ■ | ■ | ■ | ■ | ■ | M.AQVINTNSLSLiTQNNiNK.N |
| 2234 | | 732.3937 | 2194.1593 | 2194.1052 | 0.0540 | 1 | 1 | 0.8 | 1 | U | | | | | | | ■ | | | R.VSNQTQFNGVKVLASDQTMK.I |
| 2236 | | 1101.5870 | 2201.1594 | 2201.0601 | 0.0994 | 1 | 2 | 0.7 | 1 | U | | | | | | ■ | | | | K.AKADTAGFTTSTGFTVAAGGDQK.A |
| 2248 | | 741.0397 | 2220.0973 | 2220.0982 | -0.0009 | 0 | 73 | 2.4e-07 | 1 | | ■ | ■ | ■ | | | | | | | R.LSSAVTNLNNTTTNLSEAQSR.I |
| 2249 | | 1111.0560 | 2220.0974 | 2220.0982 | -0.0007 | 0 | 130 | 4.5e-13 | 1 | | ■ | ■ | ■ | | | | | | | R.LSSAVTNLNNTTTNLSEAQSR.I |
| 2280 | | 762.0590 | 2283.1552 | 2283.1053 | 0.0499 | 1 | 4 | 0.45 | 1 | U | | | | | | | ■ | | | R.VTAFVEDNGSATSVDLAAGKMGK.A + Oxidation (M) |
| 2344 | | 1276.6120 | 2551.2094 | 2551.2137 | -0.0043 | 0 | 165 | 4.7e-17 | 1 | | ■ | ■ | | | | | | | | R.ELTVQATTGTNSDSDLSSIQDEIK.S |
| 2355 | | 1322.1520 | 2642.2894 | 2642.2896 | -0.0001 | 0 | 84 | 6.3e-09 | 1 | U | ■ | | | | | ■ | | | | R.NANDGISiAQTTEGALSEINNNLQR.V |
| 2356 | | 881.7707 | 2642.2903 | 2642.2896 | 0.0007 | 0 | 68 | 2.7e-07 | 1 | U | ■ | | | | | ■ | | | | R.NANDGISiAQTTEGALSEINNNLQR.V |
| 2413 | | 1091.2460 | 3270.7162 | 3270.7167 | -0.0006 | 1 | 141 | 1e-14 | 1 | | ■ | ■ | | | | | | | | M.AQVINTNSLSLITQNNINKNQSALSTSIER.L |

**44 subsets and intersections (155 subset proteins in total)**

**2** **gi|46093564** 16 Hxx(H54 100.0%) 0|0|E223-69 [gi|283982455|ref|ADB56974| H54 0|O161:H54|O161:H54]

10 per page 1

Not what you expected? Try **the select summary**.

**Mascot: http://www.matrixscience.com/**
