# Supplementary material for: MS-H: A Novel Proteomic Approach to Isolate and Type the E. coli H Antigen Using Membrane Filtration and Liquid Chromatography-Tandem Mass Spectrometry (LC-MS/MS)
Source: PLoS One. 2013 Feb 21;8(2):e57339. doi: 10.1371/journal.pone.0057339 (PMC3578835; doi:10.1371/journal.pone.0057339)
Supplement: Representative Peptide Data S1 — Peptide data are represented as the Mascot search results from all 53 serotypes, obtained under the Orbitrap platform in Table 4 with related E. coli reference strains. “U” denotes a unique peptide specific for each of the proteins 1.1, 1.2, and beyond. The number 1.1 (shown as 1 in the peptide list and phylogenetic tree) represents the protein which obtained the highest score and confidence value after a Mascot search. This protein, known as the first hit, was used to designate the MS-H type of the unknown flagellin. Related peptides 1.2 (2), 1.3 (3), etc. represented the second, third, etc. hits for MS-H typing analysis. (DOCX) [file pone.0057339.s009.docx › H34-E589.pdf]

MASCOT Search Results

User :
E-mail :
Search title : Submitted from 20110815-0595-02 by Mascot Daemon on VARIABLE
MS data file : C:\Documents and Settings\keding\Desktop\Raw data\20110815-001-0031-00595\20110815-012-EC589MS3.RAW
Database : Flagellin_v2 (192 sequences; 89,845 residues)
Taxonomy : Bacteria (Eubacteria) (192 sequences)
Timestamp : 18 Aug 2011 at 18:55:38 GMT

Not what you expected? Try **the select summary**.

Search parameters

Score distribution

Legend

Protein Family Summary

Significance threshold p< 0.05 Max. number of families AUTO
Ions score or expect cut-off 0 Dendrograms cut at 0

Protein family 1 (out of 1)

10 per page 1

1

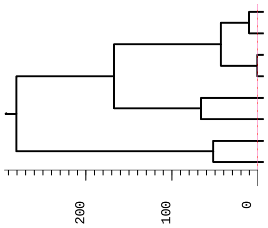

1	**gi|30059904**	3835	H34 0|H34|BP 12665
2	**gi|6009849**	2178	H49 0|H49|U11a-44
3	**gi|30059928**	1705	H52 0|H52|C2187-69
8	**gi|33590243**	625	H16 NM|O157:NM(H16)|PC02
6	**gi|30059896**	967	H29 0|H29|HW31
5	**gi|16901496**	974	H33 0|H33|0
7	**gi|5107837**	883	H7 0|O128:H7|DEC 13a
4	**gi|121144335**	1436	H55 0|O75:H55|E2987-73

Threshold (**0**): 0

		Score	Mass	Matches	Sequences	emPAI
**1.1**	**gi|30059904**<br>H34 0|H34|BP 12665	3835	55949	78 (67)	39 (34)	12.10
**1.2**	**gi|6009849**<br>H49 0|H49|U11a-44	2178	58493	52 (40)	29 (23)	3.89
**1.3**	**gi|30059928**<br>H52 0|H52|C2187-69	1705	46003	38 (32)	22 (20)	5.07
**1.4**	**gi|121144335**<br>H55 0|O75:H55|E2987-73	1436	62285	41 (32)	21 (17)	2.10
**1.5**	**gi|16901496**<br>H33 0|H33|0<br>**1 sameset of gi|16901496**	974	43915	29 (21)	17 (13)	2.43
**1.6**	**gi|30059896**<br>H29 0|H29|HW31	967	45760	29 (21)	17 (13)	2.27
**1.7**	**gi|5107837**<br>H7 0|O128:H7|DEC 13a	883	56230	29 (21)	16 (13)	1.48
**1.8**	**gi|33590243**<br>H16 NM|O157:NM(H16)|PC02<br>**3 samesets of gi|33590243**	625	55093	26 (13)	17 (8)	0.89

114 peptide matches (79 non-duplicate, 35 duplicate)

Query	Dupes	Observed	Mr(expt)	Mr(calc)	Delta	M	Score	Expect	Rank	U	1	2	3	4	5	6	7	8	Peptide
26	2	315.6944	629.3742	629.3860	-0.0118	1	4	0.41	1	U								■	K.VDKLR.S
30	2	**316.6893**	**631.3640**	**631.3653**	**-0.0013**	**0**	**27**	**0.019**	1		■	■	■	■	■	■	■	■	**R.LSSGLR.I**
73		347.1873	692.3600	692.3857	-0.0257	0	10	0.23	1	U								■	R.FTANIK.G
84		352.2023	702.3900	702.3912	-0.0011	0	3	0.8	1							■			K.AIASVDK.F
93		355.1971	708.3796	708.3806	-0.0010	0	20	0.067	1		■	■	■	■	■	■	■		R.FTSNIK.G
95		**358.7060**	**715.3974**	**715.3977**	**-0.0002**	**0**	**34**	**0.003**	1		■	■	■	■	■	■	■		**K.GLTQAAR.N**
102		364.2081	726.4016	727.3613	-0.9596	0	10	0.11	1	U	■								K.NQAGNPK.K
125		**380.6951**	**759.3756**	**759.3763**	**-0.0006**	**0**	**38**	**0.00088**	1		■	■	■	■	■	■	■		**R.LDEIDR.V**
246	1	**423.2214**	**844.4282**	**844.4402**	**-0.0120**	**0**	**24**	**0.0043**	1	**U**		■							**K.AAAGAESIR.Y**
316	1	**444.2632**	**886.5118**	**886.5124**	**-0.0005**	**0**	**83**	**5e-09**	1	**U**	■								**K.GSDILAALK.T**
345		453.7368	905.4590	904.5052	0.9539	1	3	0.52	2	U							■		K.ADMKALLK.A + Oxidation (M)
376		463.2361	924.4576	925.4141	-0.9565	0	5	0.29	1	U								■	K.QNSTGYEK.V
387	1	**466.2509**	**930.4872**	**930.4883**	**-0.0010**	**0**	**61**	**3.9e-06**	1				■	■	■	■		■	**R.SSLGAVQNR**
393		311.5060	931.4962	930.4658	1.0304	0	4	1.9	1	U								■	K.SEAAPDLTK.V
420	1	**473.2586**	**944.5026**	**944.5039**	**-0.0013**	**0**	**58**	**4.3e-06**	1		■	■					■		**R.SSLGAIQNR.L**
422		473.3201	944.6256	944.4927	0.1330	0	8	0.45	1	U					■				K.ADAGALVNSK.N
485		**489.6765**	**977.3384**	**978.5134**	**-1.1750**	**0**	**14**	**0.036**	1	**U**			■						**K.GFSVSGNALK.V**
544		**335.1765**	**1002.5077**	**1002.5094**	**-0.0017**	**1**	**32**	**0.0036**	1		■	■	■	■	■	■	■		**K.SRLDEIDR.V**
545	1	**502.2616**	**1002.5086**	**1002.5094**	**-0.0008**	**1**	**39**	**0.00074**	1		■	■	■	■	■	■	■		**K.SRLDEIDR.V**

Query	Dupes	Observed	Mr(expt)	Mr(calc)	Delta	M	Score	Expect	Rank	U	1	2	3	4	5	6	7	8	Peptide
573		508.2230	1014.4314	1014.5709	-0.1395	0	0	0.97	▶1	U								■	K.ALATTNPLSK.L
679		539.2689	1076.5232	1077.4873	-0.9640	0	15	0.046	▶1	U								■	K.NDGSAQAIMR.E + Oxidation (M)
734		551.2672	1100.5198	1100.5210	-0.0012	0	72	5.5e-07	▶1		■	■	■	■	■	■		■	K.DDAAGQAIANR.F
774	▶3	560.7864	1119.5582	1119.5594	-0.0012	0	89	1.3e-09	▶1	U	■								K.ASVAATADGMVK.D
808		568.7834	1135.5522	1135.5543	-0.0021	0	63	7.7e-07	▶1	U	■								K.ASVAATADGMVK.D + Oxidation (M)
825		382.5590	1144.6552	1144.6564	-0.0012	1	1	7.4	▶1		■	■	■	■	■	■	■	■	R.LSSGLRINSAK.D
913		596.3017	1190.5888	1190.5891	-0.0002	0	78	8.7e-08	▶1		■	■	■						K.NQSALSSSIER.L
918		598.8007	1195.5868	1194.5517	1.0352	0	4	0.38	▶1	U		■							K.DAAQSSIDFGGK.K
926		599.7641	1197.5136	1197.5150	-0.0013	0	70	1.1e-07	▶1	U	■								K.DADGVYSTENK.T
935		301.1774	1200.6805	1199.6734	1.0070	1	6	0.22	▶1	U								■	K.LRSSLGAVQNR.F
1027	▶1	623.8350	1245.6554	1245.6565	-0.0010	0	81	8.6e-09	▶1	U	■								K.ATVSGLLGNTDAK.G
1033		627.8032	1253.5918	1254.6244	-1.0326	0	0	0.96	▶1	U		■							K.FNALDAATAFSK.L
1098	▶1	644.3065	1286.5984	1286.5990	-0.0006	0	79	1.3e-08	▶1	U	■								K.GLTSSDGSTAYTK.T
1146		656.8643	1311.7140	1311.7146	-0.0006	0	24	0.0041	▶1	U							■		K.AQIIQQAGNSVLA.-
1204		672.8773	1343.7400	1343.7408	-0.0008	0	65	3.1e-07	▶1	U				■					-.SLSLITQNNINK.N
1207		673.8741	1345.7336	1345.7089	0.0247	0	6	0.27	▶1	U		■							K.DTVSSDALLAQVK.A
1226		683.3231	1364.6316	1364.6783	-0.0467	0	0	1	▶1	U				■					K.GSVSNTAATTDTLK.L
1236		458.9235	1373.7487	1373.7514	-0.0028	1	43	4.8e-05	▶1	U	■								K.KATVSGLLGNTDAK.G
1237		687.8820	1373.7494	1373.7514	-0.0020	1	109	1.4e-11	▶1	U	■								K.KATVSGLLGNTDAK.G
1328	▶2	720.9106	1439.8066	1439.8096	-0.0030	0	112	2.9e-11	▶1		■	■		■					K.AQIIQQAGNSVLAK.A
1398		747.9182	1493.8218	1493.8202	0.0017	0	69	8.6e-07	▶1		■	■	■		■	■			K.ANQVPQQVLSLLQG.-
1491	▶2	781.4191	1560.8236	1560.8260	-0.0024	0	69	5.5e-07	▶1		■	■	■	■	■	■	■		R.VSGQTQFNGVNVLAK
1559		807.9125	1613.8104	1613.8121	-0.0017	1	90	9.3e-09	▶1		■	■	■	■	■	■		■	R.INSAKDDAAGQAIANR.F
1560	▶1	538.9445	1613.8117	1613.8121	-0.0004	1	34	0.0036	▶1		■	■	■	■	■	■		■	R.INSAKDDAAGQAIANR.F
1597	▶6	817.9146	1633.8146	1633.8159	-0.0013	0	107	1.8e-11	▶1	U	■								K.TEAGVTVTAEGNTGTVK.I
1613		823.9097	1645.8048	1644.9046	0.9002	1	5	0.33	▶1	U	■								K.TTANTAAKGSDILAALK.T
1646	▶1	836.3793	1670.7440	1670.7457	-0.0017	0	121	5.2e-12	▶1		■	■	■	■	■	■	■		R.IQDADYATEVSNMSK.A
1671	▶3	843.4553	1684.8960	1684.8996	-0.0035	0	130	4.4e-13	▶1		■	■							K.IQVGANDGQTITIDLK.K
1672	▶3	843.4554	1684.8962	1685.8836	-0.9873	0	58	6.2e-06	▶2					■			■		K.IQVGANDGETITIDLK.K
1675		844.3775	1686.7404	1686.7407	-0.0002	0	90	7.6e-09	▶1		■	■	■	■	■	■	■		R.IQDADYATEVSNMSK.A + Oxidation (M)
1708		428.6962	1710.7557	1711.8451	-1.0894	1	6	0.23	▶1	U	■								K.ASVAATADGMVKDGYIK.G + Oxidation (M)
1723		860.3562	1718.6978	1718.7974	-0.0995	0	4	0.37	▶1	U								■	K.ALAYNDAPMSVYFGGK.N + Oxidation (M)
1738		866.4064	1730.7982	1730.7999	-0.0016	1	82	7.9e-09	▶1	U	■								K.EIYKDADGVYSTENK.T
1739		577.9405	1730.7997	1730.7999	-0.0002	1	36	0.00028	▶1	U	■								K.EIYKDADGVYSTENK.T
1740		866.4380	1730.8614	1730.8574	0.0040	0	118	1.7e-12	▶1	U	■								K.TSDPLAALDDAISSIDK.F
1742		577.9814	1730.9224	1731.0038	-0.0814	0	2	0.8	▶1	U		■							K.LTLMLMQAVISLLAAK.R + Oxidation (M)
1760		583.4153	1747.2241	1746.9987	0.2254	0	1	0.89	▶1	U		■							K.LTLMLMQAVISLLAAK.R + 2 Oxidation (M)
1843		605.3383	1812.9931	1813.9785	-0.9855	1	40	0.00038	▶1					■			■		K.IQVGANDGETITIDLKK.I
1843		605.3383	1812.9931	1812.9945	-0.0014	1	34	0.0017	▶2		■	■							K.IQVGANDGQTITIDLKK.I
1943		635.9772	1904.9098	1905.9466	-1.0368	1	4	0.44	▶1	U				■					K.VKDMTITSAGGNAQVATDK.A
1947		955.4606	1908.9066	1908.9065	0.0001	0	101	7.8e-11	▶1	U	■								K.IDSDTLGLSGFNVNGSADK.A
2036		1010.4950	2018.9754	2018.9769	-0.0015	0	79	1.1e-08	▶1	U						■			K.NYVANDSLVNANGAAGAAATR.V
2040		1018.0220	2034.0294	2034.0269	0.0025	1	85	3.1e-09	▶1	U	■								K.TSDPLAALDDAISSIDKFR.S
2041		679.0178	2034.0316	2034.0269	0.0046	1	62	6.3e-07	▶1	U	■								K.TSDPLAALDDAISSIDKFR.S
2044	▶1	680.0076	2037.0010	2037.0015	-0.0005	1	56	2.4e-06	▶1	U	■								K.KIDSDTLGLSGFNVNGSADK.A
2045		1019.5080	2037.0014	2037.0015	-0.0000	1	47	1.8e-05	▶1	U	■								K.KIDSDTLGLSGFNVNGSADK.A
2058		1033.0390	2064.0634	2064.0640	-0.0006	0	87	2e-09	▶1	U					■				K.VGADALGAAVGYVTVQGQNFK.A
2074		1043.0680	2084.1214	2084.1225	-0.0011	0	141	4.7e-14	▶1		■	■	■		■	■		■	M.AQVINTNSLSLiTQNNiNK.N
2075		695.7147	2084.1223	2084.1225	-0.0003	0	86	1.7e-08	▶1		■	■	■		■	■		■	M.AQVINTNSLSLiTQNNiNK.N
2164	▶1	750.3715	2248.0927	2248.0931	-0.0004	0	95	1.7e-09	▶1		■	■	■	■					R.LDSAVTNLNNTTTNLSEAQSR.I
2166	▶1	1125.0540	2248.0934	2248.0931	0.0003	0	128	1.1e-12	▶1		■	■	■	■					R.LDSAVTNLNNTTTNLSEAQSR.I
2182		761.7330	2282.1772	2283.1053	-0.9281	1	1	0.88	▶1	U								■	R.VTAFVEDNGSATSVDLAAGKMGK.A + Oxidation (M)
2201		768.4040	2302.1902	2302.1917	-0.0016	1	76	1.2e-07	▶1		■	■	■	■	■	■	■		R.LDEIDRVSGQTQFNGVNVLAK
2259		1283.6000	2565.1854	2565.1930	-0.0075	0	151	2.4e-15	▶1		■	■	■						R.ELTVQASTGTNSDSDLDSIQDEIK.S
2268		1315.1460	2628.2774	2628.2739	0.0035	0	131	3.7e-13	▶1		■	■	■	■			■	■	R.NANDGISVAQTTEGALSEINNNLQR.I
2269		877.0998	2628.2776	2628.2739	0.0037	0	91	4.1e-09	▶1		■	■	■	■			■	■	R.NANDGISVAQTTEGALSEINNNLQR.I
2308		945.7999	2834.3779	2834.3781	-0.0003	1	94	1.3e-09	▶1		■	■	■						R.IRELTVQASTGTNSDSDLDSIQDEIK.S
2315		966.8290	2897.4652	2897.4591	0.0061	1	75	1.8e-07	▶1		■	■	■	■			■	■	R.NANDGISVAQTTEGALSEINNNLQRIR.E
2315		966.8290	2897.4652	2897.4591	0.0061	1	5	1.5	▶2						■	■			R.NANDGISLAQTTEGALSEINNNLQRVR.E
2342		1086.5740	3256.7002	3256.7011	-0.0009	1	114	1.5e-11	▶1		■	■	■						M.AQVINTNSLSLITQNNINKNQSALSSSIER.L

▶ 62 subsets and intersections (159 subset proteins in total)

10 per page 1

Not what you expected? Try **the select summary**.

Mascot: http://www.matrixscience.com/
