# Supplementary material for: MS-H: A Novel Proteomic Approach to Isolate and Type the E. coli H Antigen Using Membrane Filtration and Liquid Chromatography-Tandem Mass Spectrometry (LC-MS/MS)
Source: PLoS One. 2013 Feb 21;8(2):e57339. doi: 10.1371/journal.pone.0057339 (PMC3578835; doi:10.1371/journal.pone.0057339)
Supplement: Representative Peptide Data S1 — Peptide data are represented as the Mascot search results from all 53 serotypes, obtained under the Orbitrap platform in Table 4 with related E. coli reference strains. “U” denotes a unique peptide specific for each of the proteins 1.1, 1.2, and beyond. The number 1.1 (shown as 1 in the peptide list and phylogenetic tree) represents the protein which obtained the highest score and confidence value after a Mascot search. This protein, known as the first hit, was used to designate the MS-H type of the unknown flagellin. Related peptides 1.2 (2), 1.3 (3), etc. represented the second, third, etc. hits for MS-H typing analysis. (DOCX) [file pone.0057339.s009.docx › H35-E203.pdf]

MATRIX SCIENCE MASCOT Search Results

User :
E-mail :
Search title : Submitted from 20110819-606 by Mascot Daemon on VARIABLE
MS data file : C:\Documents and Settings\keding\Desktop\Raw data\20110818-001-0031-00606\20110818-006-EC203MS2.RAW
Database : Flagellin_v2 (192 sequences; 89,845 residues)
Taxonomy : Bacteria (Eubacteria) (192 sequences)
Timestamp : 19 Aug 2011 at 17:53:21 GMT

Not what you expected? Try **the select summary**.

Search parameters

Score distribution

Legend

Protein Family Summary

Significance threshold p< 0.05 Max. number of families AUTO
Ions score or expect cut-off 0 Dendrograms cut at 0

Protein families 1–2 (out of 2)

10 per page 1

1

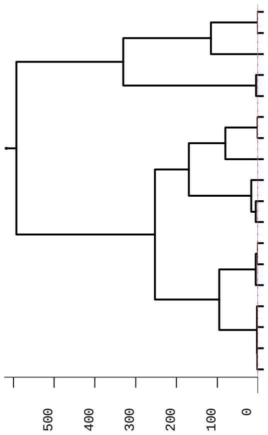

1	**gi|154710444**	1022	H35 0|H35|0
9	**gi|46093552**	442	H53 0|0|E480-68
6	**gi|33590243**	525	H16 NM|O157:NM(H16)|PC02
3	**gi|124516981**	650	H40 0|O79:H40|E49
16	**gi|46093563**	262	H54 0|0|E223-69
2	**gi|125659364**	716	H44 0|H44|E2987-73
7	**gi|30059910**	497	H39 0|H39|E3a
14	**gi|30059920**	278	H46 0|H46|5306-56
4	**gi|323174561**	615	Hxx(H25 99.7%) 0|0|LT-68
5	**gi|30577587**	541	H17 0|O15:H17|P12b
11	**gi|33590227**	310	H42 NM|O157:NM(H42)|25
12	**gi|157081065**	308	Hxx(H28 99.7%) 0|0|E24377A [gi|157159035|ref|YP_001463228| Hxx 0|0|E24377A]
18	**gi|37651050**	189	H28 0|O145:H28|0917/99 [gi|37651052|ref|CAD97427| Hxx 0|O145:H-|2032/99]
10	**gi|125659369**	398	H44 0|H44|781-55
15	**gi|170520207**	263	Hxx(H34 99.6%) 0|0|SMS-3-5 [gi|170682489|ref|YP_001743319| Hxx 0|0|SMS-3-5]
8	**gi|88683097**	465	H44 0|H44|0
17	**gi|6009849**	261	H49 0|H49|U11a-44
13	**gi|30059868**	304	H14 0|H14|F 10018-41

Threshold (**0**): 0

		Score	Mass	Matches	Sequences	emPAI
**1.1**	gi|154710444	1022	52714	35 (23)	29 (21)	2.79
	H35 0|H35|0					
**1.2**	gi|125659364	716	46286	24 (13)	19 (12)	1.45
	H44 0|H44|E2987-73					
	**2 samesets of gi|125659364**					
**1.3**	gi|124516981	650	51573	23 (13)	20 (13)	1.24
	H40 0|O79:H40|E49					
**1.4**	gi|323174561	615	46392	22 (10)	18 (10)	0.99
	Hxx(H25 99.7%) 0|0|LT-68					
	**1 sameset of gi|323174561**					
**1.5**	gi|30577587	541	36285	19 (9)	15 (9)	1.20
	H17 0|O15:H17|P12b					
**1.6**	gi|33590243	525	55093	27 (11)	19 (10)	0.89
	H16 NM|O157:NM(H16)|PC02					
	**2 samesets of gi|33590243**					
**1.7**	gi|30059910	497	44239	21 (10)	16 (9)	1.05
	H39 0|H39|E3a					
**1.8**	gi|88683097	465	55289	22 (10)	17 (9)	0.78
	H44 0|H44|0					
**1.9**	gi|46093552	442	44861	19 (7)	15 (7)	0.64
	H53 0|0|E480-68					
**1.10**	gi|125659369	398	58912	18 (7)	14 (7)	0.46
	H44 0|H44|781-55					
**1.11**	gi|33590227	310	44094	13 (5)	12 (5)	0.44
	H42 NM|O157:NM(H42)|25					
**1.12**	gi|157081065	308	59373	20 (7)	16 (7)	0.46
	Hxx(H28 99.7%) 0|0|E24377A [gi|157159035|ref|YP_001463228| Hxx 0|0|E24377A]					
	**3 samesets of gi|157081065**					
**1.13**	gi|30059868	304	56561	18 (7)	13 (7)	0.49
	H14 0|H14|F 10018-41					
	**1 sameset of gi|30059868**					

		Score	Mass	Matches	Sequences	emPAI
☑ 1.14	gi|30059920	278	57918	16 (5)	12 (5)	0.32
	H46 0|H46|5306-56					
	▶1 sameset of gi|30059920					
☑ 1.15	gi|170520207	263	55891	19 (6)	14 (6)	0.41
	Hxx(H34 99.6%) 0|0|SMS-3-5 [gi|170682489|ref|YP_001743319| Hxx 0|0|SMS-3-5]					
	▶2 samesets of gi|170520207					
☑ 1.16	gi|46093563	262	54419	19 (6)	16 (6)	0.42
	H54 0|0|E223-69					
☑ 1.17	gi|6009849	261	58493	21 (6)	17 (6)	0.39
	H49 0|H49|U11a-44					
☑ 1.18	gi|37651050	189	55672	15 (5)	11 (5)	0.33
	H28 0|O145:H28|0917/99 [gi|37651052|ref|CAD97427| Hxx 0|O145:H-|2032/99]					

▼123 peptide matches (110 non-duplicate, 13 duplicate)

Query	Dupes	Observed	Mr(expt)	Mr(calc)	Delta	M	Score	Expect	Rank	U	1	2	3	4	5	6	7	8	9	10	11	12	13	14	15	16	17	18	Peptide
9		301.1995	600.3844	600.3595	0.0250	0	5	0.35	▶3	U																■			K.LNVQK.A
22		308.6864	615.3582	616.3180	-0.9598	0	5	0.66	▶1	U																■			K.IGGADGK.T
24	▶1	310.1758	618.3370	618.2973	0.0398	1	22	0.0057	▶1	U			■																K.DKDNK.D
32	▶1	315.7001	629.3856	629.3860	-0.0004	1	4	0.43	▶1	U						■													K.VDKLR.S
35		316.6895	631.3644	631.3653	-0.0009	0	29	0.011	▶1		■	■	■	■	■	■	■	■	■	■	■	■	■	■	■	■	■	■	R.LSSGLR.I
48		323.6919	645.3692	645.3697	-0.0005	0	19	0.012	▶1	U	■																		K.TLGLDK.L
73		337.2155	672.4164	673.3759	-0.9594	0	1	0.76	▶1	U				■															K.NGATALK.L
104		352.1938	702.3730	703.3752	-1.0022	0	0	1.4	▶2	U											■								K.DDTLIK.V
114		355.1976	708.3806	708.3806	0.0000	0	12	0.46	▶1		■	■		■	■		■	■	■	■	■	■	■	■	■		■	■	R.FTSNIK.G
118		358.7128	715.4110	715.4116	-0.0005	0	10	0.36	▶1	U									■										K.IDIDLK.K
118		358.7128	715.4110	715.3977	0.0134	0	5	1.1	▶2			■		■	■		■	■		■	■	■	■	■	■		■	■	K.GLTQAAR.N
131		366.2259	730.4372	731.3813	-0.9441	0	1	2.8	▶2	U						■													R.LSEIDR.V
149	▶1	380.2030	758.3914	758.4174	-0.0259	0	33	0.003	▶1	U						■													K.LDEALAK.V
154		380.6951	759.3756	759.3763	-0.0006	0	10	0.56	▶1			■		■	■		■	■		■		■	■	■	■		■	■	R.LDEIDR.V
154	▶1	380.6951	759.3756	758.3922	0.9834	0	9	0.67	▶2	U																■			R.LNEIDR.V
169	▶1	387.2110	772.4074	772.4079	-0.0004	0	38	0.00038	▶1	U	■																		R.LQEIDR.V
169		387.2110	772.4074	773.3919	-0.9845	0	23	0.012	▶2	U			■																R.LEEIDR.V
198		398.7672	795.5198	795.3988	0.1211	0	4	0.4	▶1	U	■																		K.DVHVGGGR.V
268		418.2314	834.4482	834.4487	-0.0005	0	23	0.0049	▶1	U	■																		K.EVLFEAK.V
269		418.2369	834.4592	834.4600	-0.0007	0	38	0.00018	▶1	U		■																	K.AFVSVQGK.S
272		419.7185	837.4224	837.4232	-0.0008	0	37	0.00018	▶1	U	■																		K.DGSGYVIK.G
289		423.2212	844.4278	844.4402	-0.0124	0	13	0.05	▶1	U																	■		K.AAAGAESIR.Y
321		431.2284	860.4422	860.4426	-0.0003	0	34	0.00042	▶1	U	■																		R.VMAANDIK.G
386		452.2420	902.4694	902.5073	-0.0378	0	2	0.62	▶2	U												■							K.AATTADLLK.A
388		452.7030	903.3914	902.5073	0.8842	0	2	0.98	▶1													■						■	K.AATLDALTK.N
432	▶1	465.7659	929.5172	930.4559	-0.9387	0	1	3.8	▶1	U									■										K.DGAYHAAVK.N
434	▶2	466.2511	930.4876	930.4883	-0.0006	0	61	3.4e-06	▶1		■	■		■	■	■	■	■		■		■	■					■	R.SSLGAVQNR
464	▶2	473.2791	944.5436	944.5039	0.0397	0	4	1.1	▶1										■					■	■		■		R.SSLGAIQNR
469		474.7258	947.4370	946.4971	0.9399	0	7	0.19	▶1	U			■																K.SIDATELAK.L
501		480.2668	958.5190	958.5196	-0.0005	0	3	0.9	▶1	U											■								R.SSLGVVQNR.L
523		483.7657	965.5168	965.5182	-0.0013	1	37	0.00021	▶1	U	■																		K.KDGSGYVIK.G
548		489.2240	976.4334	976.5077	-0.0742	0	3	0.5	▶1	U																■			K.TETVTIGEK.T
608	▶1	502.2744	1002.5342	1002.5094	0.0248	1	2	3.6	▶1			■		■	■		■	■		■		■	■	■	■		■	■	K.SRLDEIDR.V
612		503.2729	1004.5312	1004.5325	-0.0012	0	48	1.6e-05	▶1	U	■																		K.VLASQQTMK.I
642		508.7900	1015.5654	1016.5502	-0.9848	0	14	0.037	▶1	U							■												K.NVDLSAVATK.L
715	▶1	529.2786	1056.5426	1056.5451	-0.0025	0	44	4.1e-05	▶1	U	■																		K.GDQLTADPLK.S
728		531.8008	1061.5870	1061.4924	0.0947	0	2	1.7	▶1	U						■													K.NDGSAQAIMR.E
750		358.5184	1072.5334	1073.5651	-1.0318	1	8	0.17	▶1	U	■																		R.VMAANDIKGR.T
755		539.2696	1076.5246	1077.4873	-0.9626	0	13	0.065	▶1	U						■													K.NDGSAQAIMR.E + Oxidat
801	▶1	551.2674	1100.5202	1100.5210	-0.0008	0	73	5.1e-07	▶1		■	■	■	■	■	■	■	■	■	■	■	■	■	■	■	■	■	■	K.DDAAGQAIANR.F
888		382.5592	1144.6558	1144.6564	-0.0006	1	11	0.66	▶1		■	■	■	■	■	■	■	■	■	■	■	■	■	■	■	■	■	■	R.LSSGLRINSAK.D
924		581.2986	1160.5826	1159.5179	1.0647	1	1	0.98	▶1	U																■			K.MTYTDSNGKK.V + Oxidat
930		582.7960	1163.5774	1163.5782	-0.0008	0	65	1.1e-06	▶1		■		■			■			■							■			K.SQSSLSSAIER.L
949		590.2794	1178.5442	1178.5455	-0.0013	0	32	0.00062	▶1	U	■																		K.LYVDNTDPDK.L
991		600.8531	1199.6916	1199.6734	0.0182	1	6	0.23	▶1	U						■													K.LRSSLGAVQNR.F
1021		406.2030	1215.5872	1216.6663	-1.0791	1	4	0.44	▶1	U	■															■			K.EINSkTLGLDK.L
1030		407.5505	1219.6297	1220.6150	-0.9853	0	14	0.041	▶1		■					■													R.VSNQTQFNGVK.V
1032		611.3140	1220.6134	1220.6150	-0.0015	0	51	8.7e-06	▶1		■					■													R.VSNQTQFNGVK.V
1097		627.8036	1253.5926	1254.6244	-1.0318	0	1	0.74	▶1	U																	■		K.FNALDAATAFSK.L
1151		643.8388	1285.6630	1286.6466	-0.9836	0	18	0.017	▶1	U																■			K.DGSAALVAGQSSPK.S
1163		431.8635	1292.5687	1292.6209	-0.0522	0	1	0.73	▶2	U																	■		K.DTGAVSVTGGSGTGK.Y
1181		651.8616	1301.7086	1301.6827	0.0260	0	5	0.72	▶2	U			■																K.AATLSDLDLNAAK.K
1222		331.9123	1323.6201	1322.6466	0.9735	1	1	0.87	▶1	U																	■		K.DAAQSSIDFGGKK.Y
1248		671.4752	1340.9358	1341.7252	-0.7894	0	15	0.034	▶1	U													■						K.ADLVAANATVVGNK.Y
1252		336.6785	1342.6849	1341.7252	0.9597	0	1	0.75	▶2	U													■						K.ADLVAANATVVGNK.Y
1354		475.2518	1422.7336	1423.6943	-0.9607	1	3	0.46	▶1	U	■																		K.DGSGYVIKGTADNK.E
1356		712.8853	1423.7560	1423.7671	-0.0110	1	1	0.79	▶1	U						■													K.VYTANITNKTATK.G
1384		481.3485	1441.0237	1441.7889	-0.7652	0	7	0.83	▶1	U							■												K.AQIVQQAGNSVLSK.A
1410		728.9094	1455.8042	1455.8045	-0.0003	0	101	1.3e-10	▶1			■		■	■														K.AQIIQQAGNSVLSK.A
1418		730.9010	1459.7874	1459.7882	-0.0008	0	93	4.5e-10	▶1	U														■					K.IGTTSVNVVLASDGK.I
1418		730.9010	1459.7874	1460.7723	-0.9848	0	19	0.013	▶2	U																		■	K.IGTTSVDVVLASDGK.I
1418		730.9010	1459.7874	1460.7722	-0.9848	0	19	0.014	▶3	U												■							K.IGSTSIDVVLASDGK.I
1452		742.3742	1482.7338	1482.7355	-0.0016	0	90	9.9e-10	▶1	U	■																		K.NLTFTDGATAPTFK.L
1456		743.8719	1485.7292	1485.7311	-0.0018	0	78	2.2e-08	▶1	U			■																K.SEGGSPILVNEDAAK.S
1463		746.9460	1491.8774	1492.7158	-0.8383	0	9	0.16	▶1	U	■																		K.SIDDALSQVDQFR.S
1466		747.9174	1493.8202	1493.8202	0.0001	0	46	0.00015	▶1	U		■		■	■		■	■		■	■	■	■	■	■		■		K.ANQVPQQVLSLxQG.-

Query	Dupes	Observed	Mr(expt)	Mr(calc)	Delta	M	Score	Expect	Rank	U	1	2	3	4	5	6	7	8	9	10	11	12	13	14	15	16	17	18	Peptide
1516		762.9081	1523.8016	1523.7580	0.0437	1	1	1.4	2	U			■																K.NSAGQFTTTKVENK.A
1543		515.7154	1544.1244	1543.7995	0.3249	0	3	0.5	1	U								■											K.ANQVPQQVLSLYQG.-
1608		534.7180	1601.1322	1601.8988	-0.7667	1	10	0.1	1	U									■										K.TVANGGDIVLSSKTIK.A
1658		548.7341	1643.1805	1643.8744	-0.6939	1	1	0.78	1	U						■													K.VQVGGKDVQLANFGGR.V
1664		823.9088	1645.8030	1644.9046	0.8984	1	10	0.1	1	U															■				K.TTANTAAKGSDILAALK.T
1693		836.3801	1670.7456	1670.7457	-0.0001	0	114	2.6e-11	1			■		■	■		■	■		■	■	■	■	■	■		■		R.IQDADYATEVSNMSK.A
1694		836.4487	1670.8828	1670.8839	-0.0011	0	105	1.8e-10	1	U				■															K.IQVGANDGQTISIDLK.K
1734		849.9781	1697.9416	1697.9424	-0.0008	0	108	1.5e-11	1	U										■									K.LAGATVAGQSGAIVVTGAR.I
1747		428.6962	1710.7557	1711.8451	-1.0894	1	3	0.46	1	U															■				K.ASVAATADGMVKDGYIK.G +
1749		571.7518	1712.2336	1711.8451	0.3885	1	16	0.026	1	U															■				K.ASVAATADGMVKDGYIK.G +
1758		858.8716	1715.7286	1715.7308	-0.0022	0	129	2.7e-13	1		■								■										R.IEDSDYATEVSNMSR.A
1758		858.8716	1715.7286	1715.7308	-0.0022	0	5	0.7	2				■			■										■			R.IEDADYATEVSNMSR.A + (
1769		574.9565	1721.8477	1721.8836	-0.0359	1	2	0.65	3	U										■									K.FTTDAATKAATTADPLK.A
1772		863.9692	1725.9238	1725.9261	-0.0022	0	80	9.4e-09	1	U			■																K.IQVGANDGEAITINLAK.I
1809		584.9459	1751.8159	1751.9053	-0.0895	1	14	0.044	1	U									■										K.TIKAEIQIDSHSADPK.A
1872		359.9967	1794.9471	1795.8589	-0.9117	0	5	0.33	1	U												■							K.STGFTVDVGATGNSAGDIK.V
1889		603.6538	1807.9396	1807.9390	0.0006	1	23	0.006	1	U			■																K.TVTDTTPGAPKVMYLSK.S
1967		624.4621	1870.3645	1869.9585	0.4060	1	8	0.14	1	U									■										K.VAANTSGLAANQTFKSYK.K
2013		638.6898	1913.0476	1913.0469	0.0007	1	40	0.00011	1	U			■																K.SIDATELAKLAINLADQK.S
2025		643.6621	1927.9645	1929.0418	-1.0774	1	9	0.21	1	U			■																K.SLQSTTNPLETIDKALAK.V
2045		648.6299	1942.8679	1942.8690	-0.0012	1	54	6.9e-06	1				■			■										■			R.SRIEDADYATEVSNMSR.A
2049		649.6650	1945.9732	1945.9745	-0.0013	0	55	3.5e-06	1	U	■																		R.TDDNVAAKPQLFLDQSGK.Y
2059		653.8130	1958.4172	1958.8640	-0.4468	1	2	1.3	1				■			■										■			R.SRIEDADYATEVSNMSR.A +
2092		997.5007	1992.9868	1992.9865	0.0004	0	126	5.6e-13	1		■		■			■			■										R.FDSAITNLGNTVNNLSSAR.S
2112		1005.5050	2008.9954	2008.9953	0.0001	0	105	2.9e-11	1	U	■																		R.NTFSTSDLAATATTELAPAK.
2177		1060.4930	2118.9714	2118.9706	0.0008	0	107	1.9e-11	1	U	■																		K.FNSTGTGTTGTAGTDFIDATG
2219		739.7247	2216.1523	2216.1219	0.0304	0	3	2.9	1	U					■														-.MAQVINTNSLSLNTQNNINK.
2219		739.7247	2216.1523	2216.1470	0.0052	0	3	2.9	2	U	■	■	■	■		■	■	■	■	■	■	■	■	■	■	■	■		-.MAQVINTNSLSLiTQNNidK.
2220		1111.0560	2220.0974	2220.0982	-0.0007	0	101	3.3e-10	1			■					■	■											R.LSSAVTNLNNTTTNLSEAQSR
2221		741.0402	2220.0988	2220.0982	0.0006	0	24	0.017	1			■					■	■											R.LSSAVTNLNNTTTNLSEAQSR
2295		1195.0940	2388.1734	2388.1697	0.0038	0	113	5.7e-12	1	U			■																K.FTINSTAATGADATITTDIDV
2305		808.4310	2422.2712	2421.1660	1.1052	1	5	0.35	1	U																■			K.NGDYEITVDKDGSAALVAGQS
2367		966.8271	2897.4595	2897.4591	0.0004	1	84	2.3e-08	1							■		■		■		■	■		■		■	■	R.NANDGISVAQTTEGALSEINN
2367		966.8271	2897.4595	2897.4591	0.0004	1	8	0.76	2			■		■							■								R.NANDGISLAQTTEGALSEINN
2372		978.2137	2931.6193	2931.6142	0.0051	1	46	2.9e-05	1			■		■	■														K.AQIIQQAGNSVLSKANQVPQQ
2372		978.2137	2931.6193	2930.5938	1.0255	1	14	0.048	2	U								■											K.AQIIQQAGNSVLAKANQVPQQ
2380		994.2006	2979.5800	2978.4080	1.1720	0	1	0.78	1	U								■											K.GGVANAVATAADMTAAGFTLA
2387		1037.1880	3108.5422	3108.5397	0.0025	1	96	1.6e-09	1			■		■	■														R.IQDADYATEVSNMSKAQIIQQ
2394		1045.2000	3132.5782	3132.5759	0.0023	1	88	1.7e-09	1			■					■	■											R.SSLGAVQNRLSSAVTNLNNTT
2395		1052.8530	3155.5372	3154.5129	1.0243	1	6	0.24	1	U																	■		R.LSWVGGALTTNDPTGSTPATN
2398		1077.5710	3229.6912	3229.6902	0.0010	1	110	1.5e-11	1		■		■			■			■							■			M.AQVINTNSLSLLTQNNLNKSQ
2403		1091.2480	3270.7222	3271.7008	-0.9786	1	127	2.9e-13	1	U											■								M.AQVINTNSLSLITQNNIDKNQ
2403		1091.2480	3270.7222	3271.6756	-0.9534	1	122	1e-12	2	U					■														M.AQVINTNSLSLNTQNNINKNQ
2403		1091.2480	3270.7222	3270.7167	0.0054	1	110	1.4e-11	3			■		■			■												M.AQVINTNSLSLITQNNINKNQ

47 subsets and intersections (143 subset proteins in total)

2 gi|76364202 13 Hxx(H6 97.8%) 0|Escherichia coli O111:H-|0

10 per page 1

Not what you expected? Try the select summary.

Mascot: http://www.matrixscience.com/
