# Supplementary material for: MS-H: A Novel Proteomic Approach to Isolate and Type the E. coli H Antigen Using Membrane Filtration and Liquid Chromatography-Tandem Mass Spectrometry (LC-MS/MS)
Source: PLoS One. 2013 Feb 21;8(2):e57339. doi: 10.1371/journal.pone.0057339 (PMC3578835; doi:10.1371/journal.pone.0057339)
Supplement: Representative Peptide Data S1 — Peptide data are represented as the Mascot search results from all 53 serotypes, obtained under the Orbitrap platform in Table 4 with related E. coli reference strains. “U” denotes a unique peptide specific for each of the proteins 1.1, 1.2, and beyond. The number 1.1 (shown as 1 in the peptide list and phylogenetic tree) represents the protein which obtained the highest score and confidence value after a Mascot search. This protein, known as the first hit, was used to designate the MS-H type of the unknown flagellin. Related peptides 1.2 (2), 1.3 (3), etc. represented the second, third, etc. hits for MS-H typing analysis. (DOCX) [file pone.0057339.s009.docx › H36-E204.pdf]

MATRIX SCIENCE MASCOT Search Results

User :
E-mail :
Search title : Submitted from 20110819-606 by Mascot Daemon on VARIABLE
MS data file : C:\Documents and Settings\keding\Desktop\Raw data\20110818-001-0031-00606\20110818-007-EC204MS2.RAW
Database : Flagellin_v2 (192 sequences; 89,845 residues)
Taxonomy : Bacteria (Eubacteria) (192 sequences)
Timestamp : 19 Aug 2011 at 17:57:40 GMT

Not what you expected? Try **the select summary**.

▸ **Search parameters**
▸ **Score distribution**
▸ **Legend**

Protein Family Summary

Significance threshold p< 0.05 Max. number of families AUTO
Ions score or expect cut-off 0 Dendrograms cut at 0

Protein families 1–2 (out of 2)

10 per page 1

▾**1**

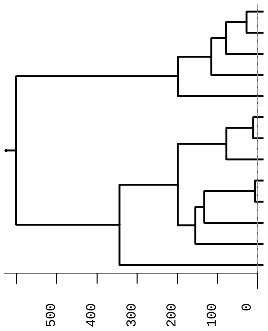

1	gi|154710451	6467	H36 0|H36|0
2	gi|33590252	1243	Hxx(H8 89.5%) 0|0|PC41
9	gi|30059872	771	H16 0|H16|F 8316-41
13	gi|40353143	417	H2 0|O111:H2|5721/96
4	gi|154710444	945	H35 0|H35|0
8	gi|125659369	794	H44 0|H44|781-55
10	gi|30059868	726	H14 0|H14|F 10018-41
12	gi|121144335	613	H55 0|O75:H55|E2987-73
3	gi|30059908	1236	H38 0|H38|P9b
11	gi|290918477	630	Hxx(H52 80.8%) 0|0|B2N
6	gi|33590227	869	H42 NM|O157:NM(H42)|25
5	gi|6009851	873	H51 0|H51|C218-70 [gi|30059926|ref|AAP13335| H51 0|H51|C218-70]
7	gi|30577587	858	H17 0|O15:H17|P12b

Threshold (**0**): 0

		Score	Mass	Matches	Sequences	emPAI
**1.1**	gi|154710451<br>H36 0|H36|0	6467	57784	144 (128)	52 (49)	42.08
**1.2**	gi|33590252<br>Hxx(H8 89.5%) 0|0|PC41	1243	52373	41 (26)	18 (12)	1.65
**1.3**	gi|30059908<br>H38 0|H38|P9b<br>▸ **1 sameset of gi|30059908**	1236	46317	31 (23)	20 (16)	2.70
**1.4**	gi|154710444<br>H35 0|H35|0	945	52714	28 (18)	15 (10)	1.34
**1.5**	gi|6009851<br>H51 0|H51|C218-70 [gi|30059926|ref|AAP13335| H51 0|H51|C218-70]	873	61407	30 (18)	18 (11)	1.08
**1.6**	gi|33590227<br>H42 NM|O157:NM(H42)|25	869	44094	25 (19)	15 (12)	1.96
**1.7**	gi|30577587<br>H17 0|O15:H17|P12b	858	36285	23 (15)	14 (10)	1.85
**1.8**	gi|125659369<br>H44 0|H44|781-55	794	58912	23 (16)	14 (11)	1.03
**1.9**	gi|30059872<br>H16 0|H16|F 8316-41	771	52639	34 (16)	18 (9)	1.07
**1.10**	gi|30059868<br>H14 0|H14|F 10018-41<br>▸ **1 sameset of gi|30059868**	726	56561	27 (16)	15 (11)	1.08
**1.11**	gi|290918477<br>Hxx(H52 80.8%) 0|0|B2N	630	42592	21 (13)	13 (9)	1.27
**1.12**	gi|121144335<br>H55 0|O75:H55|E2987-73	613	62285	21 (12)	14 (9)	0.67
**1.13**	gi|40353143<br>H2 0|O111:H2|5721/96<br>▸ **1 sameset of gi|40353143**	417	47290	18 (12)	13 (9)	0.96

▾**230 peptide matches (138 non-duplicate, 92 duplicate)**

Query	Dupes	Observed	Mr(expt)	Mr(calc)	Delta	M	Score	Expect	Rank	U	1	2	3	4	5	6	7	8	9	10	11	12	13	Peptide
5		303.1735	604.3324	603.3228	1.0096	0	1	0.88	▸1	U		■												K.VVGSDK.T
16	▸2	316.6891	631.3636	631.3653	-0.0017	0	32	0.0059	▸1		■	■	■	■	■	■	■	■	■	■	■	■	■	R.LSSGLR.I
53	▸1	347.1818	692.3490	692.3493	-0.0003	0	51	2.1e-05	▸1	U	■													K.AGDVFGK.M

Query	Dupes	Observed	Mr(expt)	Mr(calc)	Delta	M	Score	Expect	Rank	U	1	2	3	4	5	6	7	8	9	10	11	12	13	Peptide
60	1	355.1974	708.3802	708.3806	-0.0004	0	19	0.09	1		■		■	■	■	■	■	■		■	■	■		R.FTSNIK.G
76		366.7036	731.3926	731.3926	0.0001	0	31	0.003	1		■	■		■					■				■	K.GLTQASR.N
77	1	366.7156	731.4166	731.3813	0.0353	0	17	0.072	2			■			■				■					R.LSEIDR.V
78	2	366.7158	731.4170	731.4177	-0.0007	0	24	0.014	1	U	■													K.ISATNVK.I
86		378.6938	755.3730	755.3887	-0.0157	0	2	0.91	1	U													■	K.VMYLSK.S + Oxidation (M)
89		380.6865	759.3584	758.4174	0.9411	0	1	4.7	1	U									■					K.LDEALAK.V
93	1	382.1860	762.3574	762.3582	-0.0007	0	15	0.032	1	U	■													K.TTDPMAK.L
100		387.2020	772.3894	771.4490	0.9404	0	3	1.2	2	U							■							K.ALDAAIAK.V
101	2	387.2101	772.4056	772.4079	-0.0022	0	30	0.0029	1	U	■													R.LEEINR.V
101		387.2101	772.4056	772.4079	-0.0022	0	2	1.5	2	U				■										R.LQEIDR.V
103	1	388.2131	774.4116	774.4123	-0.0007	0	29	0.0028	1	U	■													K.ISAEDLK.A
112		396.2158	790.4170	790.4185	-0.0014	0	18	0.015	1	U	■													K.TGTNTAVK.S
168		418.2366	834.4586	834.4600	-0.0013	0	23	0.0055	1	U			■											K.AFVSVQGK.S
183	4	422.2860	842.5574	841.4658	1.0917	0	14	0.039	1	U		■												K.AVTQPQAK.D
205		430.7089	859.4032	860.4240	-1.0207	0	0	0.98	2	U		■												K.VELGGSDGK.T
268		452.7344	903.4542	902.5073	0.9470	0	6	0.34	1	U											■			K.IDSSALGLK.G
302	1	466.2511	930.4876	930.4883	-0.0006	0	81	3.3e-08	1				■	■			■	■	■	■	■	■		R.SSLGAVQNR
321	2	469.7324	937.4502	937.4505	-0.0003	0	47	1.9e-05	1	U	■													K.GFISTDNGK.T
330	1	473.2792	944.5438	944.5039	0.0399	0	12	0.2	1	U					■									R.SSLGAIQNR.L
369		483.7481	965.4816	965.4818	-0.0001	1	41	8.6e-05	1	U	■													K.VAYKDADGK.I
369		483.7481	965.4816	965.4818	-0.0001	1	24	0.0037	2	U										■				K.AVYKDADGK.L
370		322.8345	965.4817	965.4818	-0.0001	1	16	0.025	1	U	■													K.VAYKDADGK.I
370	1	322.8345	965.4817	965.4818	-0.0001	1	7	0.19	2	U										■				K.AVYKDADGK.L
386	1	487.2558	972.4970	972.4988	-0.0018	0	91	8.6e-10	1	U	■													R.SDLGAIQNR.F
388	2	487.2564	972.4982	971.5148	0.9834	0	73	5.8e-08	1	U	■	■												R.SdLGAIQNR.F
392		487.7608	973.5070	973.5444	-0.0373	1	9	0.16	1	U	■													K.ISAEDLKAK.A
401		489.6763	977.3380	978.5134	-1.1754	0	20	0.0095	1	U											■			K.GFSVSGNALK.V
419		493.7534	985.4922	985.5556	-0.0634	0	5	0.33	1	U												■		K.AAASNVLAAAK.N
463	2	505.7530	1009.4914	1009.4928	-0.0013	0	77	2.2e-08	1	U	■													K.SSITTESGTK.I
557		354.4786	1060.4140	1061.4924	-1.0784	0	2	0.69	1	U									■					K.NDGSAQAIMR.E
560	4	531.2817	1060.5488	1060.5513	-0.0024	0	62	6.6e-07	1	U	■													K.GSVNNTVATAK.D
562		531.7277	1061.4408	1061.4924	-0.0515	0	4	1	1	U									■					K.NDGSAQAIMR.E
586		358.5186	1072.5340	1073.5651	-1.0312	1	21	0.0087	1	U				■										R.VMAANDIKGR.T
590	1	539.2698	1076.5250	1077.4873	-0.9622	0	12	0.084	1	U									■					K.NDGSAQAIMR.E + Oxidation (M)
637	1	551.2675	1100.5204	1100.5210	-0.0006	0	74	3.4e-07	1		■	■	■	■	■	■	■	■	■	■	■	■	■	K.DDAAGQAIANR.F
666		560.2891	1118.5636	1118.5641	-0.0005	1	25	0.0031	1	U	■													K.TTDPMAKLDK.A
686		567.7843	1133.5540	1133.5564	-0.0024	1	52	1.1e-05	1	U	■													K.DADGKITTDAK.T
689		568.2600	1134.5054	1134.5591	-0.0536	1	1	1.2	1	U	■													K.TTDPMAKLDK.A + Oxidation (M)
694		569.7719	1137.5292	1137.5302	-0.0010	0	56	2.3e-06	1	U	■													K.GDGFTIDNTAK.Y
715		382.5591	1144.6555	1144.6564	-0.0009	1	0	8.6	1		■	■	■	■	■	■	■	■	■	■	■	■	■	R.LSSGLRINSAK.D
736	2	577.2907	1152.5668	1151.5822	0.9846	0	45	3.3e-05	1	U	■													K.ATLNGSEAYVK.G
737	1	577.2958	1152.5770	1152.5775	-0.0004	0	68	1.7e-07	1	U	■													K.TYTGSAGLANAK.A
747		579.3287	1156.6428	1156.6451	-0.0023	0	56	2.3e-06	1	U	■													K.AATLENLALNK.T
760	1	582.7959	1163.5772	1163.5782	-0.0010	0	50	2.8e-05	1		■	■		■					■				■	K.SQSSLSSAIER.L
762	3	582.8035	1163.5924	1163.5935	-0.0011	0	60	2.7e-06	1		■	■												R.VSGQTQFNGVK.V
786		393.5469	1177.6189	1177.6190	-0.0001	1	16	0.024	1	U	■													K.ITTDAKTETAK.T
787		589.8170	1177.6194	1177.6190	0.0004	1	59	1.4e-06	1	U	■													K.ITTDAKTETAK.T
820		397.2037	1188.5893	1187.6034	0.9859	0	3	0.47	1	U												■		K.ALDDAISQIDK.F
853	1	401.5205	1201.5397	1202.5125	-0.9729	0	6	0.26	1	U		■												K.TMSYTDADGVK.H + Oxidation (M)
892		406.8577	1217.5513	1216.6663	0.8850	1	2	0.67	1	U				■										K.EINSKTLGLDK.L
896		407.5508	1219.6306	1220.6150	-0.9844	0	2	0.63	2					■					■					R.VSNQTQFNGVK.V
899		611.7241	1221.4336	1220.6150	0.8187	0	2	0.61	1					■					■					R.VSNQTQFNGVK.V
1013		651.8621	1301.7096	1301.6827	0.0270	0	4	0.82	2	U													■	K.AATLSDLDLNAAK.K
1021		653.8360	1305.6574	1305.6776	-0.0202	0	4	0.39	1	U		■												K.LGTDTATASITGAK.L
1068	1	671.4755	1340.9364	1341.7252	-0.7888	0	4	0.4	1	U										■				K.ADLVAANATVVGNK.Y
1073	1	672.8779	1343.7412	1343.7408	0.0004	0	91	7.7e-10	1	U												■		-.SLSLITQNNINK.N
1090		456.8586	1367.5540	1367.7409	-0.1869	0	0	0.98	1	U			■											K.LTDASGLSLHNLK.D
1145		475.5921	1423.7545	1423.7671	-0.0126	1	4	0.39	1	U									■					K.VYTANITNKTATK.G
1146		712.8850	1423.7554	1423.7671	-0.0116	1	0	0.99	1	U									■					K.VYTANITNKTATK.G
1171		720.9120	1439.8094	1439.8096	-0.0002	0	53	2.4e-05	1						■	■		■		■		■		K.AQIIQQAGNSVLAK.A
1189		728.9099	1455.8052	1455.8045	0.0007	0	120	1.4e-12	1				■				■							K.AQIIQQAGNSVLSK.A
1219		743.8722	1485.7298	1485.7311	-0.0012	0	69	2.1e-07	1	U													■	K.SEGGSPILVNEDAAK.S
1232		747.9190	1493.8234	1493.8202	0.0033	0	40	0.00061	1				■		■	■	■	■		■				K.ANQVPQQVLSLLQG.-
1232		747.9190	1493.8234	1493.7474	0.0760	1	2	3.9	5	U									■					K.QNSTGYEKVQVGGK.D
1259		504.2499	1509.7279	1509.7311	-0.0032	1	30	0.0013	1	U	■													K.DLTDKGFISTDNGK.T
1260		755.8719	1509.7292	1509.7311	-0.0019	1	76	3e-08	1	U	■													K.DLTDKGFISTDNGK.T
1282		509.9453	1526.8141	1526.8165	-0.0024	1	47	2e-05	1	U	■													K.VDALRSDLGAIQNR.F
1307		772.8246	1543.6346	1543.6348	-0.0001	0	22	0.0068	1	U													■	R.IEDADYATEVSNMS.-
1330		781.4201	1560.8256	1560.8260	-0.0004	0	69	5.4e-07	1				■			■		■		■	■	■		R.VSGQTQFNGVNVLAK
1380	6	801.3754	1600.7362	1600.7369	-0.0007	0	109	1.3e-11	1	U	■													K.YYVQEDGAITNGSGK.V
1399		807.9131	1613.8116	1613.8121	-0.0005	1	99	1e-09	1		■	■	■	■	■	■	■	■	■	■	■	■	■	R.INSAKDDAAGQAIANR.F
1400		538.9449	1613.8129	1613.8121	0.0008	1	41	0.00065	1		■	■	■	■	■	■	■	■	■	■	■	■	■	R.INSAKDDAAGQAIANR.F
1447		548.7345	1643.1817	1643.8744	-0.6927	1	2	0.59	1	U									■					K.VQVGGKDVQLANFGGR.V
1473		836.3802	1670.7458	1670.7457	0.0001	0	111	5.2e-11	1				■		■	■	■	■		■	■	■		R.IQDADYATEVSNMSK.A
1474		836.4497	1670.8848	1670.8839	0.0009	0	102	3.5e-10	1	U					■									K.IQVGANDGQTISIDLK.K
1509		849.9777	1697.9408	1697.9424	-0.0016	0	93	5.5e-10	1	U								■						K.LAGATVAGQSGAIVVTGAR.I
1514	1	850.8735	1699.7324	1699.7359	-0.0035	0	146	4.4e-15	1		■	■							■					R.IEDADYATEVSNMSR.A
1543		858.8713	1715.7280	1715.7308	-0.0028	0	108	3.2e-11	1		■	■							■					R.IEDADYATEVSNMSR.A + Oxidation (M)
1567		579.7484	1736.2234	1736.8251	-0.6017	0	3	0.55	1	U					■									K.GTADGMTSGTTPVVATGAK.A + Oxidatio
1603	2	882.4622	1762.9098	1762.9101	-0.0003	0	111	8.4e-12	1	U	■													K.EITAETLGLTGFNVNGK.G
1608	1	588.9731	1763.8975	1762.9101	0.9873	0	43	5.5e-05	1	U	■													K.EITAETLGLTGFNVNGK.G
1634		894.3892	1786.7638	1786.7679	-0.0041	1	63	5.4e-07	1	U													■	R.SRIEDADYATEVSNMS.-
1659		601.2810	1800.8212	1801.9574	-1.1362	1	4	0.43	1	U					■									K.DPLAALDAAISSIDKFR.S

Query	Dupes	Observed	Mr(expt)	Mr(calc)	Delta	M	Score	Expect	Rank	U	1	2	3	4	5	6	7	8	9	10	11	12	13	Peptide
1661		451.2217	1800.8577	1799.8425	1.0152	0	0	0.91	1	U													■	K.DVFVSAADGSLTTSSDTK.V
1671		603.6538	1807.9396	1807.9390	0.0006	1	16	0.033	1	U													■	K.TVTDTTPGAPKVMYLSK.S
1671		603.6538	1807.9396	1808.8905	-0.9509	0	4	0.46	2	U											■			K.VTTNSNGEAVGYVSVQGK.D
1787		644.0261	1929.0565	1929.0531	0.0034	1	42	0.00016	1	U	■													K.AATLENLALNKTGTNTAVK.S
1795	13	646.9998	1937.9776	1937.9807	-0.0031	0	52	5.8e-06	1	U	■													K.ASVEINGSSQAVIIDHNGK.M
1797	1	969.9971	1937.9796	1937.9807	-0.0010	0	101	8.9e-11	1	U	■													K.ASVEINGSSQAVIIDHNGK.M
1813		972.4413	1942.8680	1942.8690	-0.0010	1	65	5.5e-07	1		■	■							■					R.SRIEDADYATEVSNMSR.A
1815	1	648.6307	1942.8703	1942.8690	0.0012	1	49	2.2e-05	1		■	■							■					R.SRIEDADYATEVSNMSR.A
1868	10	1001.9710	2001.9274	2001.9280	-0.0006	0	112	6.4e-12	1	U	■													K.ADTAGFTTSTGFTVAAGGDQK.A
1902	1	1019.5120	2037.0094	2037.0127	-0.0032	0	142	6.9e-15	1	U	■													R.FDSTITNLGNTVNNLTSAR.S
1935	2	1043.0680	2084.1214	2084.1225	-0.0011	0	106	1.7e-10	1		■	■	■	■	■			■		■				M.AQVINTNSLSLiTQNNiNK.N
1936		695.7155	2084.1247	2084.1225	0.0021	0	66	1.6e-06	1		■	■	■	■	■			■		■				M.AQVINTNSLSLiTQNNiNK.N
1936		695.7155	2084.1247	2085.0814	-0.9567	0	65	2.2e-06	4	U							■							M.AQVINTNSLSLNTQNNINK.N
1936		695.7155	2084.1247	2085.1066	-0.9819	0	59	8.5e-06	5	U						■								M.AQVINTNSLSLITQNNIDK.N
1937	2	1043.5610	2085.1074	2085.0814	0.0260	0	98	9.6e-10	1	U							■							M.AQVINTNSLSLNTQNNINK.N
1937	2	1043.5610	2085.1074	2085.1066	0.0009	0	68	1e-06	2	U						■								M.AQVINTNSLSLITQNNIDK.N
1963	1	1070.4970	2138.9794	2138.9790	0.0005	0	154	4e-16	1	U	■													K.MTAADDNAELFIDNSGNLTK.N
1964		714.0009	2138.9809	2138.9790	0.0019	0	76	2.8e-08	1	U	■													K.MTAADDNAELFIDNSGNLTK.N
1971		1078.4940	2154.9734	2154.9739	-0.0005	0	137	2e-14	1	U	■													K.MTAADDNAELFIDNSGNLTK.N + Oxidati
1995		734.6937	2201.0593	2201.0601	-0.0008	1	77	1.9e-08	1	U	■													K.AKADTAGFTTSTGFTVAAGGDQK.A
2004		1111.0560	2220.0974	2220.0982	-0.0007	0	93	2.2e-09	1	U			■											R.LSSAVTNLNNTTTNLSEAQSR.I
2034		758.0407	2271.1003	2271.1019	-0.0016	1	42	5.8e-05	1	U	■													K.ATLNGSEAYVKGDGFTIDNTAK.Y
2067		799.7651	2396.2735	2396.2659	0.0075	0	29	0.0011	1	U									■					R.AQILQQAGTSVLAQANQTTQNVL.-
2090	2	1261.5890	2521.1634	2521.1643	-0.0008	0	145	3.3e-15	1	U	■													K.MVDTGTVTTTIDNGFGTAQSNTYK.Y
2091		841.3954	2521.1644	2521.1643	0.0001	0	82	6.1e-09	1	U	■													K.MVDTGTVTTTIDNGFGTAQSNTYK.Y
2096		1269.5860	2537.1574	2537.1592	-0.0018	0	108	1.4e-11	1	U	■													K.MVDTGTVTTTIDNGFGTAQSNTYK.Y + Oxi
2097		846.7271	2537.1595	2537.1592	0.0003	0	87	2e-09	1	U	■													K.MVDTGTVTTTIDNGFGTAQSNTYK.Y + Oxi
2119		881.7717	2642.2933	2642.2896	0.0037	0	69	2e-07	1	U	■		■		■	■					■			R.NANDGISiAQTTEGALSEINNNLQR.V
2120	1	1322.1540	2642.2934	2642.2896	0.0039	0	113	9.4e-12	1	U	■		■		■	■					■			R.NANDGISiAQTTEGALSEINNNLQR.V
2155		919.7933	2756.3581	2756.3577	0.0004	0	48	1.7e-05	1	U			■											K.NNTGDATATQPGTSGTTVVAASIHLSTGK.N
2164	1	1409.1780	2816.3414	2816.3424	-0.0010	0	122	5.8e-13	1	U	■													R.ELSVQATNGTNSPSDLDSIQNEITQR.L
2165	1	939.7885	2816.3437	2816.3424	0.0013	0	108	1.7e-11	1	U	■													R.ELSVQATNGTNSPSDLDSIQNEITQR.L
2177		956.2006	2865.5800	2865.5672	0.0128	0	79	1.2e-08	1		■			■										R.AQILQQAGTSVLAQANQTTQNVLSLLR.-
2178		1433.7980	2865.5814	2865.5672	0.0142	0	141	7.1e-15	1		■			■										R.AQILQQAGTSVLAQANQTTQNVLSLLR.-
2179		717.4028	2865.5821	2865.5672	0.0149	0	51	7.8e-06	1		■			■										R.AQILQQAGTSVLAQANQTTQNVLSLLR.-
2182		966.8287	2897.4643	2897.4591	0.0052	1	54	2e-05	1	U	■		■		■	■					■			R.NANDGISiAQTTEGALSEINNNLQRVR.E
2182		966.8287	2897.4643	2897.4591	0.0052	1	11	0.4	3									■	■	■		■		R.NANDGISVAQTTEGALSEINNNLQRIR.E
2190		976.7897	2927.3473	2927.3495	-0.0022	1	102	6.3e-11	1	U	■													K.MVDTGTVTTTIDNGFGTAQSNTYKYDK.A
2191		978.2153	2931.6241	2931.6142	0.0099	1	13	0.066	1				■				■							K.AQIIQQAGNSVLSKANQVPQQVLSLLQG.-
2192		982.1234	2943.3484	2943.3444	0.0039	1	67	2.2e-07	1	U	■													K.MVDTGTVTTTIDNGFGTAQSNTYKYDK.A +
2197		998.1760	2991.5062	2991.5010	0.0052	1	71	8.1e-08	1	U	■													R.SDLGAIQNRFDSTITNLGNTVNNLTSAR.S
2202		1024.8440	3071.5102	3071.5119	-0.0018	1	99	1.3e-10	1	U	■													R.VRELSVQATNGTNSPSDLDSIQNEITQR.L
2207		777.1224	3104.4605	3103.4802	0.9803	1	4	0.44	1	U					■									K.SGVMIGSATFTNGKGTADGMTSGTTPVVATG
2208		1037.1880	3108.5422	3108.5397	0.0025	1	69	6.8e-07	1				■				■							R.IQDADYATEVSNMSKAQIIQQAGNSVLSK.A
2208		1037.1880	3108.5422	3108.5397	0.0025	1	30	0.006	2						■	■		■		■		■		R.IQDADYATEVSNMSKAQIIQQAGNSVLAK.A
2223		1068.1670	3201.4792	3201.4738	0.0053	0	34	0.00041	1	U	■													K.ASNSFSFDIDDAAGTTAPQVATYLNPTANDK.
2226		1077.5700	3229.6882	3229.6902	-0.0020	1	138	2.5e-14	1		■	■		■										M.AQVINTNSLSLLTQNNLNKSQSSLSSAIER.I
2240		1091.2460	3270.7162	3270.7167	-0.0006	1	118	2.3e-12	1	U			■											M.AQVINTNSLSLITQNNINKNQSALSTSIER.I
2240		1091.2460	3270.7162	3271.7008	-0.9846	1	115	4.1e-12	2	U						■								M.AQVINTNSLSLITQNNIDKNQSALSTSIER.I
2240		1091.2460	3270.7162	3271.6756	-0.9594	1	112	8.2e-12	3	U							■							M.AQVINTNSLSLNTQNNINKNQSALSTSIER.I
2273		1125.9410	3374.8012	3374.7570	0.0442	1	2	0.71	1	U						■								K.IDSSALGLSGFSVAGGALKLSDTVTQVGDGS/
2284		1144.5480	3430.6222	3430.6165	0.0057	1	152	5.9e-16	1	U	■													K.ASNSFSFDIDDAAGTTAPQVATYLNPTANDKT

62 subsets and intersections (157 subset proteins in total)

2 gi|112820172 38 H21 0|EHEC serogroup: O113:H21|0

10 per page 1

Not what you expected? Try **the select summary**.

Mascot: http://www.matrixscience.com/
