# Supplementary material for: MS-H: A Novel Proteomic Approach to Isolate and Type the E. coli H Antigen Using Membrane Filtration and Liquid Chromatography-Tandem Mass Spectrometry (LC-MS/MS)
Source: PLoS One. 2013 Feb 21;8(2):e57339. doi: 10.1371/journal.pone.0057339 (PMC3578835; doi:10.1371/journal.pone.0057339)
Supplement: Representative Peptide Data S1 — Peptide data are represented as the Mascot search results from all 53 serotypes, obtained under the Orbitrap platform in Table 4 with related E. coli reference strains. “U” denotes a unique peptide specific for each of the proteins 1.1, 1.2, and beyond. The number 1.1 (shown as 1 in the peptide list and phylogenetic tree) represents the protein which obtained the highest score and confidence value after a Mascot search. This protein, known as the first hit, was used to designate the MS-H type of the unknown flagellin. Related peptides 1.2 (2), 1.3 (3), etc. represented the second, third, etc. hits for MS-H typing analysis. (DOCX) [file pone.0057339.s009.docx › H37-E205.pdf]

MASCOT Search Results

User :
E-mail :
Search title : Submitted from 20110819-606 by Mascot Daemon on VARIABLE
MS data file : C:\Documents and Settings\keding\Desktop\Raw data\20110818-001-0031-00606\20110818-008-EC205MS2.RAW
Database : Flagellin_v2 (192 sequences; 89,845 residues)
Taxonomy : Bacteria (Eubacteria) (192 sequences)
Timestamp : 19 Aug 2011 at 18:00:42 GMT

Not what you expected? Try **the select summary**.

▶ **Search parameters**

▶ **Score distribution**

▶ **Legend**

Protein Family Summary

Significance threshold p< 0.05 Max. number of families AUTO
Ions score or expect cut-off 0 Dendrograms cut at 0

Protein family 1 (out of 1)

10 per page 1

▼1

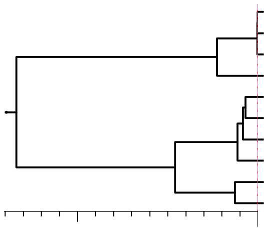

1	gi|30059906	4269	H37 0|H37|P11a [gi|301076183|ref|EFK90989| Hxx 0|0|MS 146-1] [gi|301645557|ref|ZP_07245...
5	gi|88683097	1284	H44 0|H44|0
3	gi|6009849	1809	H49 0|H49|U11a-44
4	gi|121144335	1312	H55 0|O75:H55|E2987-73
2	gi|154710451	2072	H36 0|H36|0
9	gi|112820171	735	H21 0|EHEC serogroup: O113:H21|0 [gi|291323135|ref|EFE62563| Hxx 0|0|B088] [gi|29344630...
10	gi|23477612	575	H2 0|O128:H2|0
8	gi|33590243	835	H16 NM|O157:NM(H16)|PC02
6	gi|125659364	1087	H44 0|H44|E2987-73
7	gi|323174561	1078	Hxx(H25 99.7%) 0|0|LT-68

Threshold (**0**): 0

		Score	Mass	Matches	Sequences	emPAI
1.1	gi|30059906	4269	58209	83 (71)	45 (40)	18.52
	H37 0|H37|P11a [gi|301076183|ref|EFK90989| Hxx 0|0|MS 146-1] [gi|301645557|ref|ZP_07245488| Hxx 0|0|MS 146-1]					
1.2	gi|154710451	2072	57784	47 (38)	32 (26)	4.56
	H36 0|H36|0					
1.3	gi|6009849	1809	58493	47 (33)	29 (21)	3.63
	H49 0|H49|U11a-44					
1.4	gi|121144335	1312	62285	37 (26)	22 (15)	1.95
	H55 0|O75:H55|E2987-73					
1.5	gi|88683097	1284	55289	37 (26)	23 (16)	2.57
	H44 0|H44|0					
1.6	gi|125659364	1087	46286	32 (23)	21 (16)	2.96
	H44 0|H44|E2987-73					
	▶ 2 samesets of gi|125659364					
1.7	gi|323174561	1078	46392	29 (20)	20 (15)	2.69
	Hxx(H25 99.7%) 0|0|LT-68					
	▶ 1 sameset of gi|323174561					
1.8	gi|33590243	835	55093	31 (13)	23 (10)	1.13
	H16 NM|O157:NM(H16)|PC02					
	▶ 2 samesets of gi|33590243					
1.9	gi|112820171	735	51472	18 (11)	14 (9)	0.98
	H21 0|EHEC serogroup: O113:H21|0 [gi|291323135|ref|EFE62563| Hxx 0|0|B088] [gi|293446305|ref|ZP_06662727| Hxx 0|0|B088]					
	▶ 7 samesets of gi|112820171					
1.10	gi|23477612	575	51966	16 (10)	13 (8)	0.85
	H2 0|O128:H2|0					

▼ 161 peptide matches (126 non-duplicate, 35 duplicate)

Query	Dupes	Observed	Mr(expt)	Mr(calc)	Delta	M	Score	Expect	Rank	U	1	2	3	4	5	6	7	8	9	10	Peptide
32	▶1	315.7000	629.3854	629.3860	-0.0006	1	2	0.58	▶1	U								■			K.VDKLR.S
36	▶1	316.6897	631.3648	631.3653	-0.0005	0	25	0.028	▶1		■	■	■	■	■	■	■	■	■	■	R.LSSGLR.I
96		347.1816	692.3486	692.3493	-0.0007	0	49	2.8e-05	▶1	U		■									K.AGDVFGK.M
119		355.1976	708.3806	708.3806	0.0000	0	18	0.093	▶1		■	■	■	■	■	■	■				R.FTSNIK.G
126		358.7061	715.3976	715.3977	-0.0000	0	32	0.0043	▶1		■		■	■	■	■	■				K.GLTQAAR.N
142		366.7160	731.4174	731.4177	-0.0003	0	18	0.059	▶1	U		■									K.ISATNVK.I
142		366.7160	731.4174	731.3813	0.0361	0	11	0.29	▶2									■		■	R.LSEIDR.V
152		375.1840	748.3534	748.3603	-0.0069	0	2	0.58	▶1	U	■										K.ITDTDGK.A
162		380.2029	758.3912	758.4174	-0.0261	0	29	0.0072	▶1	U								■			K.LDEALAK.V
162		380.2029	758.3912	757.4698	0.9215	1	9	0.71	▶2	U		■									K.LDKALAK.V
165	▶2	380.6950	759.3754	759.3763	-0.0008	0	38	0.00093	▶1		■		■	■	■	■	■				R.LDEIDR.V

Query	Dupes	Observed	Mr(expt)	Mr(calc)	Delta	M	Score	Expect	Rank	U	1	2	3	4	5	6	7	8	9	10	Peptide
173		382.2130	762.4114	762.4123	-0.0009	0	26	0.0022	1	U	■										K.IDSSTLK.L
184		387.2106	772.4066	772.4079	-0.0012	0	26	0.0065	1	U		■									R.LEEINR.V
189		388.2133	774.4120	774.4123	-0.0003	0	28	0.0039	1	U		■									K.ISAEDLK.A
279		418.2370	834.4594	834.4600	-0.0005	0	44	3.6e-05	1	U						■					K.AFVSVQGK.S
293		423.2216	844.4286	844.4402	-0.0116	0	16	0.024	1	U			■								K.AAAGAESIR.Y
323		430.7085	859.4024	860.4240	-1.0215	0	8	0.15	1	U										■	K.VELGGSDGK.T
374		446.2601	890.5056	890.5073	-0.0016	1	33	0.0017	1	U	■										K.KIDSSTLK.L
423		460.3311	918.6476	918.4407	0.2070	0	1	0.88	1	U			■								K.ADSTANNVK.I
447		466.7427	931.4708	930.4883	0.9826	0	3	2.3	1					■	■	■	■	■			R.SSLGAVQNR
473	2	473.2589	944.5032	944.5039	-0.0007	0	62	2.1e-06	1		■		■								R.SSLGAIQNR.L
480	1	475.2575	948.5004	948.5029	-0.0024	0	54	4.2e-06	1	U	■										K.LTGFNVNGK.A
490		476.7399	951.4652	951.4662	-0.0009	0	28	0.0016	1	U	■										K.NVYVDASGK.L
541		487.2563	972.4980	971.5148	0.9832	0	24	0.0041	1	U										■	R.SNLGAIQNR.F
546	1	487.7517	973.4888	972.4988	0.9900	0	38	0.0002	1	U		■									R.SDLGAIQNR.F
587		495.7129	989.4112	989.5029	-0.0917	0	0	1.3	1	U								■			K.GAELSASDLK.A
616	1	502.2613	1002.5080	1002.5094	-0.0014	1	47	0.00012	1		■		■	■	■	■	■				K.SRLDEIDR.V
617		335.1769	1002.5089	1002.5094	-0.0005	1	36	0.0014	1		■		■	■	■	■	■				K.SRLDEIDR.V
621		503.7470	1005.4794	1005.5607	-0.0813	1	10	0.095	1							■	■				K.AIASVDKFR.S
630		505.7534	1009.4922	1009.4928	-0.0005	0	37	0.00021	1	U		■									K.SSITTESGTK.I
643		508.2617	1014.5088	1014.5709	-0.0621	0	3	0.5	1	U								■			K.ALATTNPLSK.L
722		531.2820	1060.5494	1060.5513	-0.0018	0	60	1e-06	1	U		■									K.GSVNNTVATAK.D
725		532.8860	1063.7574	1062.5015	1.2559	0	8	0.31	1	U									■		K.VLAENNEMK.I + Oxidation (M)
793		551.2673	1100.5200	1100.5210	-0.0010	0	78	1.3e-07	1		■	■	■	■	■	■	■	■	■	■	K.DDAAGQAIANR.F
856		569.7711	1137.5276	1137.5302	-0.0026	0	33	0.00053	1	U		■									K.GDGFTIDNTAK.Y
876		382.5593	1144.6561	1144.6564	-0.0003	1	4	3.4	1		■	■	■	■	■	■	■	■	■	■	R.LSSGLRINSAK.D
896		576.7981	1151.5816	1151.5822	-0.0006	0	51	8.3e-06	1	U		■									K.ATLNGSEAYVK.G
899		577.2956	1152.5766	1152.5775	-0.0008	0	63	5.6e-07	1	U		■									K.TYTGSAGLANAK.A
917		388.5352	1162.5838	1163.5935	-1.0097	0	2	1.8	1			■								■	R.VSGQTQFNGVK.V
918	1	582.7964	1163.5782	1163.5782	0.0000	0	61	2.3e-06	1			■						■	■		K.SQSSLSSAIER.L
967		596.3016	1190.5886	1190.5891	-0.0004	0	52	3.6e-05	1		■		■								K.NQSALSSSIER.L
973		598.8010	1195.5874	1194.5517	1.0358	0	4	0.4	1	U			■								K.DAAQSSIDFGGK.K
983		400.5628	1198.6666	1199.6734	-1.0069	1	0	0.94	1	U								■			K.LRSSLGAVQNR.F
987		600.8532	1199.6918	1199.6734	0.0184	1	12	0.07	1	U								■			K.LRSSLGAVQNR.F
1025		407.5510	1219.6312	1220.6150	-0.9838	0	2	0.63	1	U								■			R.VSNQTQFNGVK.V
1148	1	648.3462	1294.6778	1294.6769	0.0010	0	64	4e-07	1	U	■										K.ALYIDSTGNLTK.N
1162		651.8620	1301.7094	1301.6827	0.0268	0	5	0.74	2	U									■		K.AATLSDLDLNAAK.K
1222		672.8773	1343.7400	1343.7408	-0.0008	0	75	3.5e-08	1	U				■							-.SLSLITQNNINK.N
1248		683.3227	1364.6308	1364.6783	-0.0475	0	0	0.99	1	U				■							K.GSVSNTAATTDTLK.L
1325		712.8854	1423.7562	1423.7671	-0.0108	1	1	0.78	1	U								■			K.VYTANITNKTATK.G
1329	2	714.3401	1426.6656	1426.6650	0.0006	0	107	1.9e-11	1	U	■										R.IDFDSGMSVTLDK.V
1353	1	720.9119	1439.8092	1439.8096	-0.0004	0	101	3.4e-10	1		■		■	■	■						K.AQIIQQAGNSVLAK.A
1381		730.1288	1458.2430	1457.7726	0.4705	0	107	1.8e-11	1	U	■										K.ITIDGSAQEVNIAK.D
1392		490.2581	1467.7525	1467.7682	-0.0157	0	2	0.68	1	U					■						K.ANQVPQQVLSLSQG.-
1412		743.8715	1485.7284	1485.7311	-0.0026	0	84	6.4e-09	1	U									■		K.SEGGSPILVNEDAAK.S
1423		747.9174	1493.8202	1493.8202	0.0001	0	49	7.6e-05	1	U	■		■		■	■	■				K.ANQVPQQVLSLxQG.-
1463		506.9331	1517.7775	1517.7950	-0.0176	0	15	0.031	1	U					■						K.ANQVPQQVLSLHQG.-
1519	2	781.4200	1560.8254	1560.8260	-0.0006	0	72	2.8e-07	1		■		■	■	■	■					R.VSGQTQFNGVNVLAK.D
1581	1	801.3751	1600.7336	1600.7369	-0.0013	0	105	2.8e-11	1	U		■									K.YYVQEDGAITNGSGK.V
1596		807.9121	1613.8096	1613.8121	-0.0025	1	93	4.9e-09	1		■	■	■	■	■	■	■	■	■	■	R.INSAKDDAAGQAIANR.F
1597		538.9440	1613.8102	1613.8121	-0.0019	1	36	0.0024	1		■	■	■	■	■	■	■	■	■	■	R.INSAKDDAAGQAIANR.F
1644		548.7344	1643.1814	1643.8744	-0.6930	1	6	0.26	1	U								■			K.VQVGGKDVQLANFGGR.V
1676	1	836.3802	1670.7458	1670.7457	0.0001	0	126	1.6e-12	1		■		■	■	■	■	■				R.IQDADYATEVSNMSK.A
1678		836.4495	1670.8844	1670.8839	0.0005	0	90	5.6e-09	1	U							■				K.IQVGANDGQTISIDLK.K
1698		843.4573	1684.9000	1684.8996	0.0005	0	103	2.1e-10	1		■		■		■						K.IQVGANDGQTITIDLK
1698		843.4573	1684.9000	1685.8836	-0.9835	0	27	0.0085	2	U				■							K.IQVGANDGETITIDLK.K
1699		844.3771	1686.7396	1686.7407	-0.0010	0	95	2.6e-09	1		■		■	■	■	■	■				R.IQDADYATEVSNMSK.A + Oxidation (M)
1713		850.8749	1699.7352	1699.7359	-0.0007	0	140	1.5e-14	1			■						■	■	■	R.IEDADYATEVSNMSR.A
1745	1	860.3559	1718.6972	1718.7974	-0.1001	0	1	0.76	1	U								■			K.ALAYNDAPMSVYFGGK.N + Oxidation (M)
1764		577.6423	1729.9051	1731.0038	-1.0987	0	1	0.78	1	U			■								K.LTLMLMQAVISLLAAK.R + Oxidation (M)
1800		586.9786	1757.9140	1757.9159	-0.0020	1	43	5.9e-05	1	U	■										K.ITIDGSAQEVNIAKDGK.I
1804		882.4628	1762.9110	1762.9101	0.0009	0	106	2.3e-11	1	U		■									K.EITAETLGLTGFNVNGK.G
1866		605.3004	1812.8794	1813.9785	-1.0992	1	0	3.9	1	U				■							K.IQVGANDGETITIDLKK.I
1867		907.4481	1812.8816	1812.8741	0.0075	0	129	5.3e-13	1	U	■										K.GAAVYAAADGSLTTETTSK.S
1945		947.5002	1892.9858	1892.9844	0.0015	0	88	1.5e-09	1	U	■										K.STTTNFDAATAVNVLAAVK.D
1946		632.0028	1892.9866	1892.9844	0.0022	0	18	0.016	1	U	■										K.STTTNFDAATAVNVLAAVK.D
1990	5	646.9996	1937.9770	1937.9807	-0.0037	0	61	7.6e-07	1	U		■									K.ASVEINGSSQAVIIDHNGK.M
1992		969.9975	1937.9804	1937.9807	-0.0002	0	79	1.3e-08	1	U		■									K.ASVEINGSSQAVIIDHNGK.M
2002		648.6302	1942.8688	1942.8690	-0.0003	1	43	7.4e-05	1			■						■	■	■	R.SRIEDADYATEVSNMSR.A
2060	1	1001.9720	2001.9294	2001.9280	0.0014	0	105	3.5e-11	1	U		■									K.ADTAGFTTSTGFTVAAGGDQK.A
2077		675.6804	2024.0194	2025.0266	-1.0072	1	0	0.99	1	U	■										K.ITDTDGKALYIDSTGNLTK.N
2092		1022.0210	2042.0274	2042.0167	0.0107	0	99	1.2e-10	1	U	■										K.SEATANPLAALDDAISQIDK.F
2112	1	1036.5180	2071.0214	2071.0222	-0.0007	0	118	1.5e-12	1	U	■										K.AYTVVNGAESYAVATNNTVK.T
2113	1	691.3478	2071.0216	2071.0222	-0.0006	0	41	8.5e-05	1	U	■										K.AYTVVNGAESYAVATNNTVK.T
2126		1043.0690	2084.1234	2084.1225	0.0009	0	142	4.6e-14	1		■	■	■		■	■	■	■	■	■	M.AQVINTNSLSLiTQNNiNK.N
2127		695.7159	2084.1259	2084.1225	0.0033	0	75	1.9e-07	1		■	■	■		■	■	■	■	■	■	M.AQVINTNSLSLiTQNNiNK.N
2170		1070.4970	2138.9794	2138.9790	0.0005	0	140	1.1e-14	1	U		■									K.MTAADDNAELFIDNSGNLTK.N
2171		714.0014	2138.9824	2138.9790	0.0034	0	41	8.3e-05	1	U		■									K.MTAADDNAELFIDNSGNLTK.N
2199	1	1094.4940	2186.9734	2186.9756	-0.0022	0	91	7.9e-10	1	U	■										K.ATVTETYHEFANGNIYDDK.G
2200	1	729.9989	2186.9749	2186.9756	-0.0008	0	63	4.7e-07	1	U	■										K.ATVTETYHEFANGNIYDDK.G
2216		739.7242	2216.1508	2215.1630	0.9877	0	0	6.3	2		■	■	■		■	■	■	■	■	■	-.MAQVINTNSLSLiTQNNiNK.N
2236		749.4071	2245.1995	2246.1390	-0.9395	1	1	0.76	1	U			■								K.DTVSSDALLAQVKADSTANNVK.I
2242	1	1125.0530	2248.0914	2248.0931	-0.0017	0	124	2.4e-12	1		■		■	■							R.LDSAVTNLNNTTTNLSEAQSR.I
2243		750.3715	2248.0927	2248.0931	-0.0004	0	88	1e-08	1		■		■	■							R.LDSAVTNLNNTTTNLSEAQSR.I

Query	Dupes	Observed	Mr(expt)	Mr(calc)	Delta	M	Score	Expect	Rank	U	1	2	3	4	5	6	7	8	9	10	Peptide
2279		768.4047	2302.1923	2302.1917	0.0005	1	62	2.9e-06	1		■		■	■	■	■					R.LDEIDRVSGQTQFNGVNVLAK.D
2294		1176.0920	2350.1694	2350.1686	0.0009	0	166	2.6e-17	1	U	■										K.VNSTVDITGASISAAAMTNELTGK.A
2295		784.3976	2350.1710	2350.1686	0.0024	0	84	4.5e-09	1	U	■										K.VNSTVDITGASISAAAMTNELTGK.A
2328		1261.5890	2521.1634	2521.1643	-0.0008	0	126	2.6e-13	1	U		■									K.MVDTGTVTTTIDNGFGTAQSNTYK.Y
2329		841.3954	2521.1644	2521.1643	0.0001	0	94	3.8e-10	1	U		■									K.MVDTGTVTTTIDNGFGTAQSNTYK.Y
2335		855.3985	2563.1737	2564.2864	-1.1127	1	0	0.97	1	U				■							-.SLSLITQNNINKNQSSMSTAIER.L + Oxidation (
2337	1	1289.6150	2577.2154	2577.2155	-0.0000	0	158	1.8e-16	1	U	■										K.NGSDTLTQATLNDVLTGANSVDDTR.I
2338	1	860.0800	2577.2182	2577.2155	0.0027	0	109	1.1e-11	1	U	■										K.NGSDTLTQATLNDVLTGANSVDDTR.I
2341		1297.1260	2592.2374	2592.2402	-0.0028	0	127	2.1e-13	1	U	■										R.ELTVQATTGTNSQSDLDSIQDEIK.S
2342		865.0876	2592.2410	2592.2402	0.0007	0	85	3.3e-09	1	U	■										R.ELTVQATTGTNSQSDLDSIQDEIK.S
2351		1315.1450	2628.2754	2628.2739	0.0015	0	124	2e-12	1		■		■	■	■			■			R.NANDGISVAQTTEGALSEINNNLQR
2352		877.0994	2628.2764	2628.2739	0.0025	0	75	1.6e-07	1		■		■	■	■			■			R.NANDGISVAQTTEGALSEINNNLQR
2355		1322.1530	2642.2914	2642.2896	0.0019	0	53	7.9e-06	1	U		■				■	■				R.NANDGISiAQTTEGALSEINNNLQR.V
2358		886.0870	2655.2392	2655.2413	-0.0021	0	91	1.9e-09	1	U	■										K.ATDANLTTAGFTQGVVDSNGNSTWTK.S
2359		1328.6270	2655.2394	2655.2413	-0.0018	0	129	3.4e-13	1	U	■										K.ATDANLTTAGFTQGVVDSNGNSTWTK.S
2360		888.1347	2661.3823	2662.2974	-0.9151	1	0	0.89	1	U									■		K.QVYVSTADGSLTTSSDTQFKIDATK.L
2386		716.0830	2860.3029	2860.3039	-0.0010	1	20	0.01	1	U	■										K.LTTDDKATVTETYHEFANGNIYDDK.G
2390		962.1561	2883.4465	2883.4434	0.0030	1	71	7.4e-08	1	U	■										R.NANDGISVAQTTEGALSEINNNLQRVR.E
2391		966.8276	2897.4610	2897.4591	0.0019	1	79	7.1e-08	1	U		■				■	■				R.NANDGISiAQTTEGALSEINNNLQRVR.E
2391		966.8276	2897.4610	2897.4591	0.0019	1	17	0.1	3				■	■	■			■			R.NANDGISVAQTTEGALSEINNNLQRIR.E
2397		978.2140	2931.6202	2931.6142	0.0060	1	39	0.00017	1							■	■				K.AQIIQQAGNSVLSKANQVPQQVLSLLQG.-
2397		978.2140	2931.6202	2931.5778	0.0424	1	6	0.3	2	U					■						K.AQIIQQAGNSVLAKANQVPQQVLSLEQG.-
2401	3	979.4569	2935.3489	2935.3472	0.0017	0	96	2.4e-10	1	U	■										K.DGSTINYTGTGNGLGIAATSAYTYHDSTK.S
2409		1031.8560	3092.5462	3092.5448	0.0014	1	99	6.1e-10	1		■		■	■	■						R.IQDADYATEVSNMSKAQIIQQAGNSVLAK.A
2410		774.1443	3092.5481	3092.5448	0.0033	1	37	0.00091	1		■		■	■	■						R.IQDADYATEVSNMSKAQIIQQAGNSVLAK.A
2421		1059.2040	3174.5902	3174.5865	0.0037	1	115	7.4e-12	1		■		■								R.SSLGAIQNRLDSAVTNLNNTTTNLSEAQSR.I
2421		1059.2040	3174.5902	3174.5865	0.0037	1	99	3.2e-10	2	U							■				R.SSLGAVQNRLDSAITNLNNTTTNLSEAQSR.I
2425		1068.1660	3201.4762	3201.4738	0.0023	0	88	1.5e-09	1	U		■									K.ASNSFSFDIDDAAGTTAPQVATYLNPTANDK.T
2427		1077.5720	3229.6942	3229.6902	0.0040	1	144	5.8e-15	1			■						■	■		M.AQVINTNSLSLLTQNNLNKSQSSLSSAIER.L
2430		1086.5750	3256.7032	3256.7011	0.0021	1	120	4.4e-12	1		■		■								M.AQVINTNSLSLITQNNINKNQSALSSSIER.L
2430		1086.5750	3256.7032	3256.7011	0.0021	1	91	2.9e-09	3	U										■	M.AQVINTNSLSLLTQNNLNKSQSSLNSAIER.L
2433		1091.2470	3270.7192	3270.7167	0.0024	1	104	6.4e-11	1							■	■				M.AQVINTNSLSLITQNNINKNQSALSTSIER.L

70 subsets and intersections (151 subset proteins in total)

10 per page 1

Not what you expected? Try **the select summary**.

Mascot: http://www.matrixscience.com/
