# Supplementary material for: MS-H: A Novel Proteomic Approach to Isolate and Type the E. coli H Antigen Using Membrane Filtration and Liquid Chromatography-Tandem Mass Spectrometry (LC-MS/MS)
Source: PLoS One. 2013 Feb 21;8(2):e57339. doi: 10.1371/journal.pone.0057339 (PMC3578835; doi:10.1371/journal.pone.0057339)
Supplement: Representative Peptide Data S1 — Peptide data are represented as the Mascot search results from all 53 serotypes, obtained under the Orbitrap platform in Table 4 with related E. coli reference strains. “U” denotes a unique peptide specific for each of the proteins 1.1, 1.2, and beyond. The number 1.1 (shown as 1 in the peptide list and phylogenetic tree) represents the protein which obtained the highest score and confidence value after a Mascot search. This protein, known as the first hit, was used to designate the MS-H type of the unknown flagellin. Related peptides 1.2 (2), 1.3 (3), etc. represented the second, third, etc. hits for MS-H typing analysis. (DOCX) [file pone.0057339.s009.docx › H38-E206.pdf]

MASCOT Search Results

User :
E-mail :
Search title : Submitted from 20110819-606 by Mascot Daemon on VARIABLE
MS data file : C:\Documents and Settings\keding\Desktop\Raw data\20110818-001-0031-00606\20110818-009-EC206MS2.RAW
Database : Flagellin_v2 (192 sequences; 89,845 residues)
Taxonomy : Bacteria (Eubacteria) (192 sequences)
Timestamp : 19 Aug 2011 at 18:05:49 GMT

Not what you expected? Try **the select summary**.

Search parameters

Score distribution

Legend

Protein Family Summary

Significance threshold p< 0.05 Max. number of families AUTO
Ions score or expect cut-off 0 Dendrograms cut at 0

Protein family 1 (out of 1)

10 per page 1

1

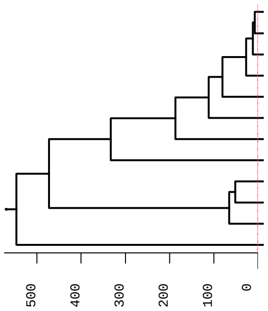

1	gi|30059908	4118	H38 0|H38|P9b
5	gi|88683097	1302	H44 0|H44|0
9	gi|6009849	973	H49 0|H49|U11a-44
7	gi|30059912	1084	H41 0|H41|RVC 1787
6	gi|145997	1102	Hxx(H12 99.7%) 0|0|Bi 316-42 (Orskov)
10	gi|121144335	826	H55 0|O75:H55|E2987-73
4	gi|330911731	1376	Hxx(H4 99.4%) 0|0|AA86
3	gi|33590227	1424	H42 NM|O157:NM(H42)|25
12	gi|112820171	639	H21 0|EHEC serogroup: O113:H21|0 [gi|291323135|ref|EFE62563| Hxx 0|0|B088] [gi|29344630...
8	gi|154710451	1013	H36 0|H36|0
11	gi|33590243	689	H16 NM|O157:NM(H16)|PC02
2	gi|291470750	1432	Hxx(H37 99.8%) 0|0|B354 [gi|293410282|ref|ZP_06653858| Hxx 0|0|B354]

Threshold (**0**): 0

		Score	Mass	Matches	Sequences	emPAI
1.1	gi|30059908<br>H38 0|H38|P9b	4118	46317	87 (73)	41 (38)	40.23
1.2	gi|291470750<br>Hxx(H37 99.8%) 0|0|B354 [gi|293410282|ref|ZP_06653858| Hxx 0|0|B354]<br>**1 sameset of gi|291470750**	1432	58221	37 (26)	23 (17)	2.17
1.3	gi|33590227<br>H42 NM|O157:NM(H42)|25	1424	44094	33 (25)	20 (15)	3.56
1.4	gi|330911731<br>Hxx(H4 99.4%) 0|0|AA86<br>**2 samesets of gi|330911731**	1376	36283	38 (28)	20 (18)	6.46
1.5	gi|88683097<br>H44 0|H44|0	1302	55289	39 (27)	22 (16)	2.37
1.6	gi|145997<br>Hxx(H12 99.7%) 0|0|Bi 316-42 (Orskov)	1102	61008	32 (22)	18 (13)	1.57
1.7	gi|30059912<br>H41 0|H41|RVC 1787<br>**1 sameset of gi|30059912**	1084	57346	34 (24)	18 (14)	1.73
1.8	gi|154710451<br>H36 0|H36|0	1013	57784	35 (20)	22 (14)	1.71
1.9	gi|6009849<br>H49 0|H49|U11a-44	973	58493	35 (21)	21 (13)	1.54
1.10	gi|121144335<br>H55 0|O75:H55|E2987-73	826	62285	31 (19)	18 (12)	1.16
1.11	gi|33590243<br>H16 NM|O157:NM(H16)|PC02<br>**2 samesets of gi|33590243**	689	55093	29 (14)	18 (9)	1.01
1.12	gi|112820171<br>H21 0|EHEC serogroup: O113:H21|0 [gi|291323135|ref|EFE62563| Hxx 0|0|B088] [gi|293446305|ref|ZP_06662727| Hxx 0|0|B088]<br>**7 samesets of gi|112820171**	639	51472	20 (12)	14 (8)	0.98

163 peptide matches (125 non-duplicate, 38 duplicate)

Query	Dupes	Observed	Mr(expt)	Mr(calc)	Delta	M	Score	Expect	Rank	U	1	2	3	4	5	6	7	8	9	10	11	12	Peptide
29	1	315.7000	629.3854	629.3860	-0.0006	1	9	0.12	1	U											■		K.VDKLR.S
32	1	316.6895	631.3644	631.3653	-0.0009	0	29	0.012	1		■	■	■	■	■	■	■	■	■	■	■	■	R.LSSGLR.I
70		344.2054	686.3962	686.3963	-0.0000	0	7	0.2	1	U						■							K.ALDQLK.D
92		352.1937	702.3728	703.3752	-1.0024	0	1	1.2	2	U			■										K.DDTLIK.V

Query	Dupes	Observed	Mr(expt)	Mr(calc)	Delta	M	Score	Expect	Rank	U	1	2	3	4	5	6	7	8	9	10	11	12	Peptide
93	▶1	352.2025	702.3904	702.3912	-0.0007	0	47	3.1e-05	▶1	U	■												K.AIASVDK.F
101		355.1971	708.3796	708.3806	-0.0010	0	19	0.077	▶1		■	■	■	■	■	■	■	■	■	■			R.FTSNIK.G
102		355.6708	709.3270	709.3283	-0.0012	0	18	0.095	▶1	U	■												K.FDDSVK.T
106		358.7062	715.3978	715.3977	0.0002	0	32	0.0045	▶1		■	■	■	■	■	■	■		■	■			K.GLTQAAR.N
119		366.6973	731.3800	731.3926	-0.0125	0	0	3.1	▶1									■			■	■	K.GLTQASR.N
136	▶1	380.2030	758.3914	758.4174	-0.0259	0	31	0.0043	▶1	U											■		K.LDEALAK.V
136		380.2030	758.3914	757.4698	0.9217	1	1	4.6	▶2	U								■					K.LDKALAK.V
139	▶2	380.6950	759.3754	759.3763	-0.0008	0	38	0.00087	▶1		■	■		■	■	■	■		■	■			R.LDEIDR.V
153		386.7315	771.4484	771.4490	-0.0006	0	15	0.034	▶1	U				■									K.ALDAAIAK.V
241	▶1	418.2365	834.4584	834.4600	-0.0015	0	41	8.6e-05	▶1	U	■												K.AFVSVQGK.S
262	▶1	423.2215	844.4284	844.4402	-0.0118	0	25	0.0035	▶1	U									■				K.AAAGAESIR.Y
403	▶2	466.2506	930.4866	930.4883	-0.0016	0	73	2.4e-07	▶1		■			■	■		■			■	■		R.SSLGAVQNR
444		475.2527	948.4908	948.5029	-0.0120	0	7	0.21	▶1	U		■											K.LTGFNVNGK.A
504	▶1	487.7122	973.4098	972.4988	0.9110	0	12	0.086	▶1	U								■					R.SDLGAIQNR.F
546		495.3521	988.6896	989.5142	-0.8245	0	3	0.5	▶1	U												■	K.NSAGQLTATK.V
574	▶1	502.2616	1002.5086	1002.5094	-0.0008	1	38	0.001	▶1		■	■		■	■	■	■		■	■			K.SRLDEIDR.V
575		335.1771	1002.5095	1002.5094	0.0001	1	34	0.0021	▶1		■	■		■	■	■	■		■	■			K.SRLDEIDR.V
580		503.7867	1005.5588	1005.5607	-0.0019	1	58	1.4e-06	▶1	U	■												K.AIASVDKFR.S
581		336.1939	1005.5599	1005.5607	-0.0008	1	31	0.00075	▶1	U	■												K.AIASVDKFR.S
642		518.7690	1035.5234	1035.5237	-0.0002	0	38	0.00016	▶1	U	■												K.SFGIDDAALK.N
726		539.7794	1077.5442	1077.4873	0.0570	0	2	0.64	▶1	U											■		K.NDGSAQAIMR.E + Oxidation (M)
729		360.5066	1078.4980	1077.4873	1.0107	0	1	0.81	▶1	U											■		K.NDGSAQAIMR.E + Oxidation (M)
770		551.2675	1100.5204	1100.5210	-0.0006	0	69	1.2e-06	▶1		■	■	■	■	■	■	■	■	■	■	■	■	K.DDAAGQAIANR.F
800		559.8270	1117.6394	1118.5641	-0.9247	1	6	0.26	▶1	U								■					K.TTDPMAKLDK.A
829		568.2596	1134.5046	1133.5564	0.9482	1	0	1.7	▶1	U								■					K.DADGKITTDAK.T
830	▶1	379.1755	1134.5047	1133.5564	0.9482	1	2	1.2	▶1	U								■					K.DADGKITTDAK.T
855		573.3353	1144.6560	1144.6564	-0.0003	1	41	0.00071	▶1		■	■	■	■	■	■	■	■	■	■	■	■	R.LSSGLRINSAK.D
856		382.5593	1144.6561	1144.6564	-0.0003	1	3	4.8	▶1		■	■	■	■	■	■	■	■	■	■	■	■	R.LSSGLRINSAK.D
892		582.2989	1162.5832	1163.5935	-1.0103	0	5	0.89	▶1	U								■					R.VSGQTQFNGVK.V
893		388.5352	1162.5838	1163.5935	-1.0097	0	5	0.94	▶1	U								■					R.VSGQTQFNGVK.V
897		389.8676	1166.5810	1166.5819	-0.0010	1	15	0.077	▶1	U	■												K.FDDSVKTDLK.L
898		584.2978	1166.5810	1166.5819	-0.0009	1	46	5.8e-05	▶1	U	■												K.FDDSVKTDLK.L
931		397.1862	1188.5368	1187.6034	0.9334	0	2	0.7	▶1	U										■			K.ALDDAISQIDK.F
941		598.8011	1195.5876	1194.5517	1.0360	0	6	0.25	▶1	U									■				K.DAAQSSIDFGGK.K
952		600.8531	1199.6916	1199.6734	0.0182	1	12	0.07	▶1	U											■		K.LRSSLGAVQNR.F
962	▶1	603.3093	1204.6040	1204.6048	-0.0007	0	61	1.6e-06	▶1		■		■	■									K.NQSALSTSIER.L
995		407.5506	1219.6300	1220.6150	-0.9850	0	1	0.86	▶1	U											■		R.VSNQTQFNGVK.V
1047		627.8036	1253.5926	1254.6244	-1.0318	0	2	0.68	▶1	U									■				K.FNALDAATAFSK.L
1110		648.4721	1294.9296	1294.6769	0.2528	0	2	0.58	▶1	U		■											K.ALYIDSTGNLTK.N
1124		434.9183	1301.7331	1300.6987	1.0344	1	1	1.9	▶2	U									■				K.LKDGDSVAVAAQK.Y
1190		672.8782	1343.7418	1343.7408	0.0010	0	71	7.8e-08	▶1	U										■			-.SLSLITQNNINK.N
1213		684.8773	1367.7400	1367.7409	-0.0008	0	95	2.9e-10	▶1	U	■												K.LTDASGLSLHNLK.D
1214		456.9207	1367.7403	1367.7409	-0.0006	0	48	1.5e-05	▶1	U	■												K.LTDASGLSLHNLK.D
1291		712.8848	1423.7550	1423.7671	-0.0120	1	1	0.91	▶1	U											■		K.VYTANITNKTATK.G
1339	▶2	728.9089	1455.8032	1455.8045	-0.0013	0	108	2.2e-11	▶1		■			■									K.AQIIQQAGNSVLSK.A
1340		486.2751	1455.8035	1455.8045	-0.0010	0	2	0.96	▶1		■			■									K.AQIIQQAGNSVLSK.A
1345	▶1	729.8932	1457.7718	1457.7726	-0.0007	0	126	2.8e-13	▶1	U		■											K.ITIDGSAQEVNIAK.D
1345	▶1	729.8932	1457.7718	1457.7726	-0.0007	0	40	9.3e-05	▶2	U							■						K.ISIGGTEQEVNIAK.D
1348		730.3632	1458.7118	1459.7630	-1.0512	1	4	0.42	▶3	U												■	K.NSAGQLTATKVENK.A
1354		490.2579	1467.7519	1467.7682	-0.0163	0	5	0.3	▶1	U					■								K.ANQVPQQVLSLSQG.-
1371		743.8723	1485.7300	1485.7311	-0.0010	0	65	4.3e-07	▶1	U												■	K.SEGGSPILVNEDAAK.S
1384		747.9181	1493.8216	1493.8202	0.0015	0	54	2.2e-05	▶1	U	■	■	■	■	■	■	■		■				K.ANQVPQQVLSLxQG.-
1426		506.9341	1517.7805	1517.7950	-0.0146	0	18	0.017	▶1	U					■								K.ANQVPQQVLSLHQG.-
1430	▶2	762.8965	1523.7784	1523.7831	-0.0047	0	114	6.8e-12	▶1	U	■												K.IDSSTLGLNGFSVSK.N
1495	▶2	781.9135	1561.8124	1560.8260	0.9864	0	63	2.3e-06	▶1		■	■	■		■	■	■		■	■			R.VSGQTQFNGVNVLAK
1538	▶2	797.4153	1592.8160	1592.8158	0.0002	0	124	3.6e-13	▶1	U	■												K.SYAATVAANGNVTLNK.A
1562		807.9129	1613.8112	1613.8121	-0.0009	1	92	5.8e-09	▶1		■	■	■	■	■	■	■	■	■	■	■	■	R.INSAKDDAAGQAIANR.F
1563		538.9445	1613.8117	1613.8121	-0.0004	1	44	0.00033	▶1		■	■	■	■	■	■	■	■	■	■	■	■	R.INSAKDDAAGQAIANR.F
1639	▶1	836.3798	1670.7450	1670.7457	-0.0007	0	126	1.6e-12	▶1		■	■	■	■	■	■	■		■	■			R.IQDADYATEVSNMSK.A
1662		844.3770	1686.7394	1686.7407	-0.0012	0	117	1.4e-11	▶1		■	■	■	■	■	■	■		■	■			R.IQDADYATEVSNMSK.A + Oxidation (M)
1675		565.9092	1694.7058	1694.7781	-0.0724	0	1	0.79	▶1	U										■			K.DMTITSAGGNAQVATDK.A + Oxidation (M)
1747		874.7718	1747.5290	1747.8840	-0.3549	0	16	0.026	▶1	U					■								K.ITTDAQTATTATTDPLK.A
1776		592.7603	1775.2591	1774.8962	0.3629	1	0	2.5	▶1												■	■	K.DDAAGQAIANRFTANIK.G
1810	▶2	900.4782	1798.9418	1798.9425	-0.0006	0	127	4.1e-13	▶1	U	■												K.IQVGANDGQTISIDLQK.I
1867	▶1	925.4358	1848.8570	1848.8602	-0.0032	0	111	7.6e-12	▶1	U	■												K.DENGNLTNQYVVQNGGK.S
1868		617.2933	1848.8581	1848.8602	-0.0021	0	46	2.5e-05	▶1	U	■												K.DENGNLTNQYVVQNGGK.S
1925		947.5003	1892.9860	1892.9844	0.0017	0	99	1.1e-10	▶1	U		■											K.STTTNFDAATAVNVLAAVK.D
1975	▶1	647.0003	1937.9791	1937.9807	-0.0016	0	52	5.7e-06	▶1	U								■					K.ASVEINGSSQAVIIDHNGK.M
1982		648.6298	1942.8676	1942.8690	-0.0015	1	21	0.014	▶1									■			■	■	R.SRIEDADYATEVSNMSR.A
2039	▶1	1001.7120	2001.4094	2001.9280	-0.5186	0	14	0.038	▶1	U								■					K.ADTAGFTTSTGFTVAAGGDQK.A
2061		675.6805	2024.0197	2025.0266	-1.0069	1	6	0.28	▶1	U		■											K.ITDTDGKALYIDSTGNLTK.N
2072		681.0019	2039.9839	2040.9236	-0.9397	0	0	0.92	▶1	U						■							K.TGADADAATANAGVSFTDTASK.E
2083		1036.5160	2071.0174	2071.0222	-0.0047	0	97	1.8e-10	▶1	U		■											K.AYTVVNGAESYAVATNNTVK.T
2095	▶1	1043.0680	2084.1214	2084.1225	-0.0011	0	128	1.2e-12	▶1		■	■		■	■	■	■	■	■		■	■	M.AQVINTNSLSLiTQNNiNK.N
2095	▶1	1043.0680	2084.1214	2085.1066	-0.9851	0	82	4.3e-08	▶4	U			■										M.AQVINTNSLSLITQNNIDK.N
2096		695.7150	2084.1232	2084.1225	0.0006	0	80	5.9e-08	▶1		■	■		■	■	■	■	■	■		■	■	M.AQVINTNSLSLiTQNNiNK.N
2096		695.7150	2084.1232	2085.1066	-0.9834	0	69	9.1e-07	▶4	U			■										M.AQVINTNSLSLITQNNIDK.N
2132		1070.4960	2138.9774	2138.9790	-0.0015	0	140	1.1e-14	▶1	U								■					K.MTAADDNAELFIDNSGNLTK.N
2151		729.9994	2186.9764	2186.9756	0.0007	0	60	9.1e-07	▶1	U		■											K.ATVTETYHEFANGNIYDDK.G
2169		739.7250	2216.1532	2215.1630	0.9901	0	1	5	▶1		■	■		■	■	■	■	■	■		■	■	-.MAQVINTNSLSLiTQNNiNK.N
2169		739.7250	2216.1532	2216.1470	0.0061	0	0	6.1	▶5	U			■										-.MAQVINTNSLSLITQNNIDK.N
2171	▶1	1111.0560	2220.0974	2220.0982	-0.0007	0	117	9.2e-12	▶1		■				■								R.LSSAVTNLNNTTTNLSEAQSR.I
2172		741.0399	2220.0979	2220.0982	-0.0003	0	70	4.6e-07	▶1		■				■								R.LSSAVTNLNNTTTNLSEAQSR.I

Query	Dupes	Observed	Mr(expt)	Mr(calc)	Delta	M	Score	Expect	Rank	U	1	2	3	4	5	6	7	8	9	10	11	12	Peptide
2186		745.3708	2233.0906	2233.1074	-0.0168	1	3	0.72	▶1	U					■								K.DANGKITTDAQTATTATTDPLK.A
2232		768.4047	2302.1923	2302.1917	0.0005	1	57	1e-05	▶1		■	■			■	■	■		■	■			R.LDEIDRVSGQTQFNGVNVLAK.D
2245		1176.0910	2350.1674	2350.1686	-0.0011	0	132	6.5e-14	▶1	U		■											K.VNSTVDITGASISAAAMTNELTGK.A
2246		785.0099	2352.0079	2353.1471	-1.1393	0	0	0.9	▶1	U										■			K.DSLLSMLAPNAGDSFTASVSIGGK.A + Oxida
2263		1219.6410	2437.2674	2437.2700	-0.0026	0	108	1.5e-11	▶1	U	■												K.NAVSVGDAITQLPGETAADAPVTIK.F
2264		610.3251	2437.2713	2437.2700	0.0013	0	50	1e-05	▶1	U	■												K.NAVSVGDAITQLPGETAADAPVTIK.F
2265		813.4315	2437.2727	2437.2700	0.0026	0	66	2.3e-07	▶1	U	■												K.NAVSVGDAITQLPGETAADAPVTIK.F
2285		1276.6130	2551.2114	2551.2137	-0.0023	0	145	5e-15	▶1		■		■										R.ELTVQATTGTNSDSDLSSIQDEIK.S
2286		851.4122	2551.2148	2551.2137	0.0011	0	74	6e-08	▶1		■		■										R.ELTVQATTGTNSDSDLSSIQDEIK.S
2299		881.7716	2642.2930	2642.2896	0.0034	0	75	5.4e-08	▶1	U						■							R.NANDAISVAQTTEGALSEINNNLQR.I
2299		881.7716	2642.2930	2642.2896	0.0034	0	74	6.1e-08	▶2	U	■		■					■					R.NANDGISiAQTTEGALSEINNNLQR.V
2300		1322.1540	2642.2934	2642.2896	0.0039	0	112	1e-11	▶1	U	■		■					■					R.NANDGISiAQTTEGALSEINNNLQR.V
2300		1322.1540	2642.2934	2642.2896	0.0039	0	96	4.7e-10	▶3	U						■							R.NANDAISVAQTTEGALSEINNNLQR.I
2301		888.1348	2661.3826	2662.2974	-0.9148	1	0	0.93	▶1	U												■	K.QVYVSTADGSLTTSSDTQFKIDATK.L
2321	▶1	919.7927	2756.3563	2756.3577	-0.0014	0	99	1.3e-10	▶1	U	■												K.NNTGDATATQPGTSGTTVVAASIHLSTGK.N
2329		928.1140	2781.3202	2781.3192	0.0009	0	76	2.3e-08	▶1	U	■												K.NSVDADVTASTEFTGASTNDPLTLLDK.A
2330		699.5835	2794.3049	2794.3960	-0.0911	1	2	0.94	▶1	U									■				K.RLSWVGGALTTNDPTGSTPATMSSLFK.A
2336		936.4680	2806.3822	2806.3832	-0.0011	1	81	1.5e-08	▶1		■		■										R.VRELTVQATTGTNSDSDLSSIQDEIK.S
2336		936.4680	2806.3822	2806.3832	-0.0010	1	62	1.1e-06	▶2	U				■									R.IRELTVQASTGTNSDSDLSSIQDEIK.S
2348		966.8273	2897.4601	2897.4591	0.0010	1	66	1.4e-06	▶1	U	■		■					■					R.NANDGISiAQTTEGALSEINNNLQRVR.E
2348		966.8273	2897.4601	2897.4591	0.0010	1	16	0.14	▶3						■		■		■	■	■		R.NANDGISVAQTTEGALSEINNNLQRIR.E
2352		978.2133	2931.6181	2931.6142	0.0039	1	31	0.0011	▶1		■			■									K.AQIIQQAGNSVLSKANQVPQQVLSLLQG.-
2363		998.1756	2991.5050	2991.5010	0.0040	1	34	0.0004	▶1	U								■					R.SDLGAIQNRFDSTITNLGNTVNNLTSAR.S
2374		1037.1880	3108.5422	3108.5397	0.0025	1	72	3.3e-07	▶1		■			■									R.IQDADYATEVSNMSKAQIIQQAGNSVLSK.A
2374		1037.1880	3108.5422	3108.5397	0.0025	1	5	1.9	▶2			■	■		■	■	■		■	■			R.IQDADYATEVSNMSKAQIIQQAGNSVLAK.A +
2386	▶1	1067.2040	3198.5902	3198.5905	-0.0003	1	92	6e-10	▶1	U	■												K.LTDASGLSLHNLKDENGNLTNQYVVQNGGK.S
2387	▶1	800.6550	3198.5909	3198.5905	0.0004	1	51	8.9e-06	▶1	U	■												K.LTDASGLSLHNLKDENGNLTNQYVVQNGGK.S
2390		1068.1640	3201.4702	3201.4738	-0.0037	0	33	0.00056	▶1	U								■					K.ASNSFSFDIDDAAGTTAPQVATYLNPTANDK.T
2393		1077.5710	3229.6912	3229.6902	0.0010	1	131	1.2e-13	▶1									■			■	■	M.AQVINTNSLSLLTQNNLNKSQSSLSSAIER.L
2394		808.4301	3229.6913	3229.6902	0.0011	1	46	3.6e-05	▶1									■			■	■	M.AQVINTNSLSLLTQNNLNKSQSSLSSAIER.L
2400		1633.2960	3264.5774	3264.5746	0.0028	0	107	2.1e-11	▶1	U	■												K.ANVTYSDVANGIDTATQSGQLVQVGADSTGTPK.
2401	▶1	1089.2000	3264.5782	3264.5746	0.0035	0	94	3.7e-10	▶1	U	■												K.ANVTYSDVANGIDTATQSGQLVQVGADSTGTPK.
2402		817.1535	3264.5849	3264.5746	0.0103	0	46	2.5e-05	▶1	U	■												K.ANVTYSDVANGIDTATQSGQLVQVGADSTGTPK.
2406		818.6866	3270.7173	3271.7008	-0.9835	1	28	0.0025	▶1	U			■										M.AQVINTNSLSLITQNNIDKNQSALSTSIER.L
2406		818.6866	3270.7173	3270.7167	0.0006	1	28	0.0025	▶2		■			■									M.AQVINTNSLSLITQNNINKNQSALSTSIER.L
2407		1091.2470	3270.7192	3271.7008	-0.9816	1	130	1.6e-13	▶1	U			■										M.AQVINTNSLSLITQNNIDKNQSALSTSIER.L
2407		1091.2470	3270.7192	3270.7167	0.0024	1	116	3.4e-12	▶2		■			■									M.AQVINTNSLSLITQNNINKNQSALSTSIER.L
2435		844.7079	3374.8025	3374.7570	0.0455	1	0	0.96	▶1	U			■										K.IDSSALGLSGFSVAGGALKLSDTVTQVGDGSAAF

▶54 subsets and intersections (148 subset proteins in total)

10 per page 1

Not what you expected? Try **the select summary**.

Mascot: http://www.matrixscience.com/
