# Supplementary material for: MS-H: A Novel Proteomic Approach to Isolate and Type the E. coli H Antigen Using Membrane Filtration and Liquid Chromatography-Tandem Mass Spectrometry (LC-MS/MS)
Source: PLoS One. 2013 Feb 21;8(2):e57339. doi: 10.1371/journal.pone.0057339 (PMC3578835; doi:10.1371/journal.pone.0057339)
Supplement: Representative Peptide Data S1 — Peptide data are represented as the Mascot search results from all 53 serotypes, obtained under the Orbitrap platform in Table 4 with related E. coli reference strains. “U” denotes a unique peptide specific for each of the proteins 1.1, 1.2, and beyond. The number 1.1 (shown as 1 in the peptide list and phylogenetic tree) represents the protein which obtained the highest score and confidence value after a Mascot search. This protein, known as the first hit, was used to designate the MS-H type of the unknown flagellin. Related peptides 1.2 (2), 1.3 (3), etc. represented the second, third, etc. hits for MS-H typing analysis. (DOCX) [file pone.0057339.s009.docx › H39-E207.pdf]

MATRIX SCIENCE MASCOT Search Results

User :
E-mail :
Search title : Submitted from 20110819-606 by Mascot Daemon on VARIABLE
MS data file : C:\Documents and Settings\keding\Desktop\Raw data\20110818-001-0031-00606\20110818-010-EC207MS2.RAW
Database : Flagellin_v2 (192 sequences; 89,845 residues)
Taxonomy : Bacteria (Eubacteria) (192 sequences)
Timestamp : 19 Aug 2011 at 18:08:37 GMT

Not what you expected? Try **the select summary**.

▸ **Search parameters**
▸ **Score distribution**
▸ **Legend**

Protein Family Summary

Significance threshold p< 0.05 Max. number of families AUTO
Ions score or expect cut-off 0 Dendrograms cut at 0

Protein families 1–2 (out of 2)

10 per page 1

▾ **1**

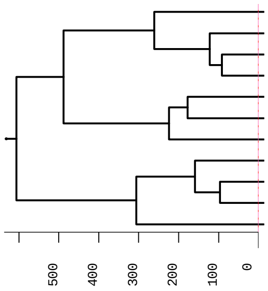

1	gi|30059910	2553	H39 0|H39|E3a
10	gi|33590243	637	H16 NM|O157:NM(H16)|PC02
9	gi|154710451	645	H36 0|H36|0
11	gi|124516981	563	H40 0|O79:H40|E49
2	gi|30059908	2243	H38 0|H38|P9b
7	gi|6009851	1143	H51 0|H51|C218-70 [gi|30059926|ref|AAP13335| H51 0|H51|C218-70]
6	gi|33590227	1239	H42 NM|O157:NM(H42)|25
3	gi|6009831	1363	Hxx(H1 100.0%) 0|0|Su 1242 [gi|312946515|ref|ADR27342| H1 0|O83:H1|NRG 857C] [gi|3075...
5	gi|88683097	1254	H44 0|H44|0
8	gi|121144335	885	H55 0|O75:H55|E2987-73
4	gi|291470750	1292	Hxx(H37 99.8%) 0|0|B354 [gi|293410282|ref|ZP_06653858| Hxx 0|0|B354]

Threshold (**0**): 0

		Score	Mass	Matches	Sequences	emPAI
☑ 1.1	gi|30059910<br>H39 0|H39|E3a	2553	44239	59 (47)	29 (26)	12.32
☑ 1.2	gi|30059908<br>H38 0|H38|P9b<br>▸ 1 sameset of gi|30059908	2243	46317	54 (44)	29 (25)	9.40
☑ 1.3	gi|6009831<br>Hxx(H1 100.0%) 0|0|Su 1242 [gi|312946515|ref|ADR27342| H1 0|O83:H1|NRG 857C] [gi|307553937|ref|ADN46712| Hxx 0|0|ABU 83972] [gi|301050721|ref|ZP_07197581| Hxx 0|0|MS 185-1] [gi|30...	1363	60901	42 (31)	29 (24)	3.36
☑ 1.4	gi|291470750<br>Hxx(H37 99.8%) 0|0|B354 [gi|293410282|ref|ZP_06653858| Hxx 0|0|B354]<br>▸ 1 sameset of gi|291470750	1292	58221	40 (28)	26 (20)	2.95
☑ 1.5	gi|88683097<br>H44 0|H44|0	1254	55289	37 (27)	21 (16)	2.37
☑ 1.6	gi|33590227<br>H42 NM|O157:NM(H42)|25	1239	44094	32 (23)	21 (15)	3.24
☑ 1.7	gi|6009851<br>H51 0|H51|C218-70 [gi|30059926|ref|AAP13335| H51 0|H51|C218-70]	1143	61407	31 (21)	21 (14)	1.56
☑ 1.8	gi|121144335<br>H55 0|O75:H55|E2987-73	885	62285	31 (21)	19 (14)	1.40
☑ 1.9	gi|154710451<br>H36 0|H36|0	645	57784	24 (12)	18 (8)	0.74
☑ 1.10	gi|33590243<br>H16 NM|O157:NM(H16)|PC02<br>▸ 2 samesets of gi|33590243	637	55093	24 (12)	17 (8)	0.79
☑ 1.11	gi|124516981<br>H40 0|O79:H40|E49	563	51573	18 (11)	13 (8)	0.86

▾ **142 peptide matches (113 non-duplicate, 29 duplicate)**

Query	Dupes	Observed	Mr(expt)	Mr(calc)	Delta	M	Score	Expect	Rank	U	1	2	3	4	5	6	7	8	9	10	11	Peptide
31		315.7000	629.3854	629.3860	-0.0006	1	2	0.63	▸1	U										■		K.VDKLR.S
36	▸2	**316.6897**	**631.3648**	**631.3653**	**-0.0005**	**0**	**33**	**0.0045**	▸1		■	■	■	■	■	■	■	■	■	■	■	**R.LSSGLR.I**
86		344.2049	686.3952	686.3963	-0.0010	0	8	0.14	▸1	U			■									K.ALDQLK.D
109	▸1	**352.2029**	**702.3912**	**702.3912**	**0.0001**	**0**	**46**	**3.5e-05**	▸1		■	■										**K.AIASVDK.F**
112		355.1975	708.3804	708.3806	-0.0002	0	18	0.1	▸1		■	■	■	■	■	■	■	■	■			R.FTSNIK.G
117		**358.7061**	**715.3976**	**715.3977**	**-0.0000**	**0**	**29**	**0.0086**	▸1		■	■	■	■	■	■	■	■				**K.GLTQAAR.N**
138		**380.2029**	**758.3912**	**758.4174**	**-0.0261**	**0**	**29**	**0.0076**	▸1	**U**										■		**K.LDEALAK.V**
138		380.2029	758.3912	757.4698	0.9215	1	11	0.44	▸2	U									■			K.LDKALAK.V
141		**380.6948**	**759.3750**	**759.3763**	**-0.0012**	**0**	**36**	**0.0013**	▸1		■	■	■	■	■			■				**R.LDEIDR.V**

Query	Dupes	Observed	Mr(expt)	Mr(calc)	Delta	M	Score	Expect	Rank	U	1	2	3	4	5	6	7	8	9	10	11	Peptide
170		396.6992	791.3838	790.4185	0.9654	0	0	0.98	▶1	U									■			K.TGTNTAVK.S
249		418.2368	834.4590	834.4600	-0.0009	0	38	0.00016	▶1	U		■										K.AFVSVQGK.S
277		424.7213	847.4280	847.4287	-0.0007	0	68	1.6e-07	▶1	U			■									K.DGDTVTIK.A
410	▶2	466.2506	930.4866	930.4883	-0.0016	0	79	6.2e-08	▶1		■	■			■			■		■		R.SSLGAVQNR
448	▶1	473.2588	944.5030	944.5039	-0.0009	0	46	8.4e-05	▶1				■	■			■					R.SSLGAIQNR.L
482		480.2667	958.5188	958.5196	-0.0007	0	3	0.92	▶1	U						■						R.SSLGVVQNR.L
539		491.2348	980.4550	980.4563	-0.0013	0	33	0.00047	▶1	U			■									K.YSIDANNGK.V
543		492.7347	983.4548	984.5604	-1.1055	0	1	0.83	▶1	U											■	K.LAINLADQK.S
590	▶2	502.2611	1002.5076	1002.5094	-0.0018	1	35	0.0017	▶1		■	■	■	■	■			■				K.SRLDEIDR.V
591		335.1767	1002.5083	1002.5094	-0.0011	1	27	0.013	▶1		■	■	■	■	■			■				K.SRLDEIDR.V
597		503.7868	1005.5590	1005.5607	-0.0017	1	59	1.2e-06	▶1		■	■										K.AIASVDKFR.S
598		336.1940	1005.5602	1005.5607	-0.0005	1	18	0.017	▶1		■	■										K.AIASVDKFR.S
616		508.2619	1014.5092	1014.5709	-0.0617	0	1	0.79	▶1	U										■		K.ALATTNPLSK.L
623	▶2	509.2811	1016.5476	1016.5502	-0.0026	0	67	2e-07	▶1	U	■											K.NVDLSAVATK.L
629		511.2612	1020.5078	1020.5088	-0.0009	0	71	1.1e-07	▶1	U			■									K.VTVDSGTGTGK.Y
664		520.3945	1038.7744	1037.4778	1.2967	0	5	0.33	▶1	U							■					K.AVDNGNGTYK.V
712		531.2951	1060.5756	1060.5764	-0.0008	0	26	0.0026	▶1	U			■									K.AATISDLTAAK.M
736		359.8728	1076.5966	1077.4873	-0.8907	0	2	0.87	▶1	U										■		K.NDGSAQAIMR.E + Oxidation (M)
769	▶1	547.2670	1092.5194	1092.5200	-0.0005	0	71	8.8e-08	▶1	U			■									K.AGDGQSIGFNK.T
784	▶1	551.2671	1100.5196	1100.5210	-0.0014	0	76	2.2e-07	▶1		■	■	■	■	■	■	■	■	■	■	■	K.DDAAGQAIANR.F
814		559.3037	1116.5928	1117.5979	-1.0050	0	1	0.86	▶1	U							■					K.AATISDLTAQK.A
817		559.8259	1117.6372	1118.5641	-0.9269	1	6	0.26	▶1	U									■			K.TTDPMAKLDK.A
881		382.5590	1144.6552	1144.6564	-0.0012	1	2	5.2	▶1		■	■	■	■	■	■	■	■	■	■	■	R.LSSGLRINSAK.D
923		581.3029	1160.5912	1160.5925	-0.0012	0	77	2.8e-08	▶1	U			■									K.ALDEAISSIDK.F
979		596.3013	1190.5880	1190.5891	-0.0010	0	44	0.0002	▶1				■	■			■					K.NQSALSSSIER.L
1000		600.8525	1199.6904	1199.6734	0.0170	1	8	0.17	▶1	U										■		K.LRSSLGAVQNR.F
1010		603.3092	1204.6038	1204.6048	-0.0009	0	58	2.9e-06	▶1		■	■				■						K.NQSALSTSIER.L
1119		632.2707	1262.5268	1261.6514	0.8754	0	1	0.71	▶1	U									■			K.IAIAGSTDGTTAGK.I
1167		648.4717	1294.9288	1294.6769	0.2520	0	5	0.33	▶1	U				■								K.ALYIDSTGNLTK.N
1181		651.8612	1301.7078	1301.6827	0.0252	0	6	0.64	▶1	U											■	K.AATLSDLDLNAAK.K
1195		437.5504	1309.6294	1308.6231	1.0062	1	8	0.16	▶1	U									■			K.TETAKTTDPMAK.L + Oxidation (M)
1234		671.3724	1340.7302	1340.7300	0.0003	0	70	1e-07	▶1	U	■											K.GTATQNPLALLDK.A
1241		672.8777	1343.7408	1343.7408	0.0000	0	64	4.1e-07	▶1	U								■				-.SLSLITQNNINK.N
1345		712.8853	1423.7560	1423.7671	-0.0110	1	1	0.84	▶1	U										■		K.VYTANITNKTATK.G
1365		720.9119	1439.8092	1439.8096	-0.0004	0	94	2e-09	▶1				■	■	■	■	■	■				K.AQIIQQAGNSVLAK.A
1368		481.6031	1441.7875	1441.7889	-0.0014	0	8	0.17	▶1	U	■											K.AQIVQQAGNSVLSK.A
1369	▶1	721.9011	1441.7876	1441.7889	-0.0012	0	98	1.6e-10	▶1	U	■											K.AQIVQQAGNSVLSK.A
1392		728.9085	1455.8024	1455.8045	-0.0021	0	94	6.7e-10	▶1	U		■										K.AQIIQQAGNSVLSK.A
1413		490.2572	1467.7498	1467.7682	-0.0184	0	1	0.75	▶1	U					■							K.ANQVPQQVLSLSQG.-
1436		743.8719	1485.7292	1485.7311	-0.0018	0	62	9.4e-07	▶1	U											■	K.SEGGSPILVNEDAAK.S
1441		496.6838	1487.0296	1487.8195	-0.7899	1	1	1.4	▶1	U									■			K.ISATNVKISAEDLK.A
1445		747.3499	1492.6852	1492.6868	-0.0016	0	85	2e-08	▶1	U			■									K.TASVTMGGTTYNFK.T + Oxidation (M)
1447		747.9175	1493.8204	1493.8202	0.0003	0	72	3.8e-07	▶1	U	■	■	■	■	■	■	■					K.ANQVPQQVLSLxQG.-
1452		500.2523	1497.7351	1498.7151	-0.9801	0	0	0.92	▶1	U						■						K.TDITYSGGDITGATK.D
1456		500.6959	1499.0659	1498.7879	0.2780	0	2	0.63	▶1	U							■					K.DPLAALDAAISSIDK.F
1488		506.9330	1517.7772	1517.7950	-0.0179	0	15	0.031	▶1	U					■							K.ANQVPQQVLSLHQG.-
1547	▶2	781.4199	1560.8252	1560.8260	-0.0008	0	70	4.6e-07	▶1		■	■	■	■	■	■		■				R.VSGQTQFNGVNVLAK
1590		790.4112	1578.8078	1578.8101	-0.0022	0	115	3.1e-12	▶1	U	■											K.LSDSITTIGNTTAASK.N
1609	▶2	797.4142	1592.8138	1592.8158	-0.0020	0	98	1.7e-10	▶1	U		■										K.SYAATVAANGNVTLNK.A
1641		807.9123	1613.8100	1613.8121	-0.0021	1	93	4.6e-09	▶1		■	■	■	■	■	■	■	■	■	■	■	R.INSAKDDAAGQAIANR.F
1642		538.9440	1613.8102	1613.8121	-0.0019	1	32	0.0059	▶1		■	■	■	■	■	■	■	■	■	■	■	R.INSAKDDAAGQAIANR.F
1654		540.3945	1618.1617	1617.8475	0.3142	1	5	0.31	▶1	U				■								K.LTGFNVNGKAAVDNAK.A
1671		545.0657	1632.1753	1632.8319	-0.6566	1	2	0.63	▶1	U									■			K.GSVNNTVATAKDLTDK.G
1686		548.7341	1643.1805	1643.8744	-0.6939	1	0	0.96	▶1	U										■		K.VQVGGKDVQLANFGGR.Y
1731		836.3798	1670.7450	1670.7457	-0.0007	0	116	1.6e-11	▶1		■	■	■	■	■	■	■	■				R.IQDADYATEVSNMSK.A
1753		844.3773	1686.7400	1686.7407	-0.0006	0	120	7.3e-12	▶1		■	■	■	■	■	■	■	■				R.IQDADYATEVSNMSK.A + Oxidation (M)
1803		573.4133	1717.2181	1717.8734	-0.6553	0	0	1.9	▶2	U								■				K.LTTEASTAAETTANPLK.A
1849	▶3	875.9521	1749.8896	1749.8897	-0.0001	0	121	7.9e-13	▶1	U	■											K.VTSDANGAAVGYVTIQGK.N
1849	▶1	875.9521	1749.8896	1748.9309	0.9588	0	8	0.16	▶2	U						■						K.IDSSALGLSGFSVAGGALK.L
1850		584.3053	1749.8941	1749.8897	0.0043	0	8	0.18	▶1	U	■											K.VTSDANGAAVGYVTIQGK.N
1867		588.7477	1763.2213	1763.7387	-0.5174	0	0	0.99	▶1	U							■					K.YDADAGNFSYNNTANK.T
1915	▶2	900.4781	1798.9416	1798.9425	-0.0008	0	143	1e-14	▶1	U		■										K.IQVGANDGQTISIDLQK.I
1915	▶2	900.4781	1798.9416	1798.9789	-0.0372	1	139	2.6e-14	▶2	U							■					K.IQVGANDGQTISIDLKK.I
1920		600.9818	1799.9236	1798.9789	0.9447	1	25	0.0064	▶1	U		■					■					K.IQVGANDGQTISIDLkK.I
2028	▶1	632.0019	1892.9839	1892.9844	-0.0005	0	15	0.033	▶1	U				■								K.STTTNFDAATAVNVLAAVK.D
2029		947.5002	1892.9858	1892.9844	0.0015	0	88	1.6e-09	▶1	U				■								K.STTTNFDAATAVNVLAAVK.D
2043		953.4627	1904.9108	1905.9466	-1.0357	1	3	0.51	▶1	U								■				K.VKDMTITSAGGNAQVATDK.A
2052		638.6902	1913.0488	1913.0469	0.0019	1	26	0.0027	▶1	U											■	K.SIDATELAKLAINLADQK.S
2062		643.6621	1927.9645	1929.0418	-1.0774	1	3	0.98	▶1	U											■	K.SLQSTTNPLETIDKALAK.V
2068	▶1	647.0004	1937.9794	1937.9807	-0.0013	0	59	1.3e-06	▶1	U									■			K.ASVEINGSSQAVIIDHNGK.M
2070		970.5021	1938.9896	1938.9898	-0.0002	0	99	1.1e-10	▶1	U	■											K.IDSSTLGLSGFSVSQNSLK.L
2071		647.3378	1938.9916	1938.9898	0.0017	0	73	5.1e-08	▶1	U	■											K.IDSSTLGLSGFSVSQNSLK.L
2105		987.0207	1972.0268	1972.0266	0.0003	0	62	5.7e-07	▶1	U			■									K.AGDVAASLLPPAGQTASGVYK.A
2154		675.6800	2024.0182	2025.0266	-1.0084	1	8	0.17	▶1	U				■								K.ITDTDGKALYIDSTGNLTK.N
2178		1036.5200	2071.0254	2071.0222	0.0033	0	113	5e-12	▶1	U				■								K.AYTVVNGAESYAVATNNTVK.T
2186		1043.0690	2084.1234	2084.1225	0.0009	0	127	1.2e-12	▶1		■	■	■	■	■		■		■	■	■	M.AQVINTNSLSLiTQNNiNK.N
2186		1043.0690	2084.1234	2085.1066	-0.9831	0	79	7.4e-08	▶4	U						■						M.AQVINTNSLSLITQNNIDK.N
2187		695.7152	2084.1238	2084.1225	0.0012	0	62	3.9e-06	▶1		■	■	■	■	■		■		■	■	■	M.AQVINTNSLSLiTQNNiNK.N
2187		695.7152	2084.1238	2085.1066	-0.9828	0	61	4.8e-06	▶4	U						■						M.AQVINTNSLSLITQNNIDK.N
2233		729.9987	2186.9743	2186.9756	-0.0014	0	69	1.2e-07	▶1	U				■								K.ATVTETYHEFANGNIYDDK.G
2247		739.7239	2216.1499	2215.1630	0.9868	0	3	3.4	▶1		■	■	■	■	■		■		■	■	■	-.MAQVINTNSLSLiTQNNiNK.N
2247		739.7239	2216.1499	2216.1470	0.0028	0	0	6.2	▶6	U						■						-.MAQVINTNSLSLITQNNIDK.N
2249	▶2	1111.0560	2220.0974	2220.0982	-0.0007	0	117	9.3e-12	▶1		■	■			■							R.LSSAVTNLNNTTTNLSEAQSR.I

Query	Dupes	Observed	Mr(expt)	Mr(calc)	Delta	M	Score	Expect	Rank	U	1	2	3	4	5	6	7	8	9	10	11	Peptide
2250		741.0402	2220.0988	2220.0982	0.0006	0	69	4.9e-07	▶1		■	■			■							R.LSSAVTNLNNTTTNLSEAQSR.I
2275		1125.0540	2248.0934	2248.0931	0.0003	0	66	1.5e-06	▶1				■	■		■	■	■				R.LDSAVTNLNNTTTNLSEAQSR.I
2307		768.4044	2302.1914	2302.1917	-0.0004	1	35	0.0015	▶1		■	■	■	■	■			■				R.LDEIDRVSGQTQFNGVNVLAK
2321		584.5411	2334.1353	2333.1533	0.9820	1	1	0.8	▶1	U		■										K.DGSMKIQVGANDGQTISIDLQK.I + Oxidation
2323		1176.0900	2350.1654	2350.1686	-0.0031	0	87	1.8e-09	▶1	U				■								K.VNSTVDITGASISAAAMTNELTGK.A
2327		797.0659	2388.1759	2388.1697	0.0062	0	43	5.2e-05	▶1	U											■	K.FTINSTAATGADATITTDIDVYK.N
2359		1276.6130	2551.2114	2551.2137	-0.0023	0	165	4.9e-17	▶1		■	■				■						R.ELTVQATTGTNSDSDLSSIQDEIK.S
2360		851.4120	2551.2142	2551.2137	0.0005	0	68	2.3e-07	▶1		■	■				■						R.ELTVQATTGTNSDSDLSSIQDEIK.S
2366		1307.1460	2612.2774	2612.2790	-0.0015	0	143	5e-15	▶1	U	■											R.NANDGISLAQTAEGALSEINNNLQR.V
2367		871.7672	2612.2798	2612.2790	0.0008	0	81	7.5e-09	▶1	U	■											R.NANDGISLAQTAEGALSEINNNLQR.V
2369		1315.1420	2628.2694	2628.2739	-0.0045	0	73	2.6e-07	▶1				■	■	■			■		■		R.NANDGISVAQTTEGALSEINNNLQR
2372		1322.1510	2642.2874	2642.2896	-0.0021	0	62	1.1e-06	▶1	U		■				■	■		■			R.NANDGISiAQTTEGALSEINNNLQR.V
2389		919.7929	2756.3569	2756.3577	-0.0008	0	83	4.8e-09	▶1	U		■										K.NNTGDATATQPGTSGTTVVAASIHLSTGK.N
2398		966.8275	2897.4607	2897.4591	0.0016	1	74	2.3e-07	▶1	U		■				■	■		■			R.NANDGISiAQTTEGALSEINNNLQRVR.E
2398		966.8275	2897.4607	2897.4591	0.0016	1	12	0.3	▶3				■		■			■		■		R.NANDGISVAQTTEGALSEINNNLQRIR.E
2410		1032.5150	3094.5232	3094.5241	-0.0009	1	56	2.6e-06	▶1	U	■											R.IQDADYATEVSNMSKAQIVQQAGNSVLSK.A
2416		1077.5710	3229.6912	3229.6902	0.0010	1	128	2.2e-13	▶1										■	■	■	M.AQVINTNSLSLLTQNNLNKSQSSLSSAIER.L
2420		818.6865	3270.7169	3271.7008	-0.9839	1	29	0.0016	▶1	U						■						M.AQVINTNSLSLITQNNIDKNQSALSTSIER.L
2420		818.6865	3270.7169	3270.7167	0.0002	1	25	0.0051	▶2		■	■										M.AQVINTNSLSLITQNNINKNQSALSTSIER.L
2421		1091.2470	3270.7192	3270.7167	0.0024	1	126	4e-13	▶1		■	■										M.AQVINTNSLSLITQNNINKNQSALSTSIER.L
2421		1091.2470	3270.7192	3271.7008	-0.9816	1	114	6e-12	▶3	U						■						M.AQVINTNSLSLITQNNIDKNQSALSTSIER.L

▶ 66 subsets and intersections (158 subset proteins in total)

▶ **2** **gi|112820172** 18 H21 0|EHEC serogroup: O113:H21|0

10 per page 1

Not what you expected? Try **the select summary**.

Mascot: http://www.matrixscience.com/
