# Supplementary material for: MS-H: A Novel Proteomic Approach to Isolate and Type the E. coli H Antigen Using Membrane Filtration and Liquid Chromatography-Tandem Mass Spectrometry (LC-MS/MS)
Source: PLoS One. 2013 Feb 21;8(2):e57339. doi: 10.1371/journal.pone.0057339 (PMC3578835; doi:10.1371/journal.pone.0057339)
Supplement: Representative Peptide Data S1 — Peptide data are represented as the Mascot search results from all 53 serotypes, obtained under the Orbitrap platform in Table 4 with related E. coli reference strains. “U” denotes a unique peptide specific for each of the proteins 1.1, 1.2, and beyond. The number 1.1 (shown as 1 in the peptide list and phylogenetic tree) represents the protein which obtained the highest score and confidence value after a Mascot search. This protein, known as the first hit, was used to designate the MS-H type of the unknown flagellin. Related peptides 1.2 (2), 1.3 (3), etc. represented the second, third, etc. hits for MS-H typing analysis. (DOCX) [file pone.0057339.s009.docx › H40-E208.pdf]

MATRIX SCIENCE MASCOT Search Results

User :
E-mail :
Search title : Submitted from 20110819-606 by Mascot Daemon on VARIABLE
MS data file : C:\Documents and Settings\keding\Desktop\Raw data\20110818-001-0031-00606\20110818-011-EC208MS2.RAW
Database : Flagellin_v2 (192 sequences; 89,845 residues)
Taxonomy : Bacteria (Eubacteria) (192 sequences)
Timestamp : 19 Aug 2011 at 18:13:54 GMT

Not what you expected? Try **the select summary**.

▸**Search parameters**

▸**Score distribution**

▸**Legend**

Protein Family Summary

Significance threshold p< 0.05 Max. number of families AUTO
Ions score or expect cut-off 0 Dendrograms cut at 0

Protein families 1–3 (out of 3)

10 per page 1

▾**1**

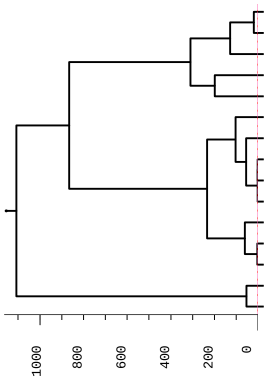

1	gi|124516981	7251	H40 0|O79:H40|E49
3	gi|192925955	2267	H2 0|O103:H2|E22 [gi|193066370|ref|ZP_03047418| H2 0|O103:H2|E22] [gi|260844327|ref|YP...
7	gi|30059872	1545	H16 0|H16|F 8316-41
5	gi|154710451	1725	H36 0|H36|0
6	gi|46093545	1552	H3 0|0|Bi7327-41
15	gi|121144335	459	H55 0|O75:H55|E2987-73
11	gi|33590227	900	H42 NM|O157:NM(H42)|25
8	gi|30059908	1469	H38 0|H38|P9b
13	gi|16901498	704	H34 0|H34|0
12	gi|6009849	704	H49 0|H49|U11a-44
10	gi|88683097	935	H44 0|H44|0
9	gi|30059910	1178	H39 0|H39|E3a
14	gi|192955539	697	H9 0|O111:H9|E110019 [gi|193071144|ref|ZP_03052068| H9 0|O111:H9|E110019]
2	gi|33590237	4240	H11 NM|O157:NM(H11)|F199
4	gi|62532942	2255	H11 0|O26:H11|0

Threshold (**0**): 0

		Score	Mass	Matches	Sequences	emPAI
☑ **1.1**	gi|124516981	7251	51573	146 (131)	48 (46)	70.53
	H40 0|O79:H40|E49					
☑ **1.2**	gi|33590237	4240	50951	97 (79)	35 (33)	23.45
	H11 NM|O157:NM(H11)|F199					
☑ **1.3**	gi|192925955	2267	51996	47 (38)	18 (14)	3.65
	H2 0|O103:H2|E22 [gi|193066370|ref|ZP_03047418| H2 0|O103:H2|E22] [gi|260844327|ref|YP_003222105| H2 0|O103:H2|12009] [gi|257759474|ref|BAI30971| H2 0|O103:H2|12009] [gi|3231585...					
☑ **1.4**	gi|62532942	2255	37909	56 (45)	22 (21)	10.30
	H11 0|O26:H11|0					
☑ **1.5**	gi|154710451	1725	57784	40 (32)	19 (16)	2.99
	H36 0|H36|0					
☑ **1.6**	gi|46093545	1552	55534	36 (27)	14 (12)	1.66
	H3 0|0|Bi7327-41					
☑ **1.7**	gi|30059872	1545	52639	44 (28)	20 (11)	2.17
	H16 0|H16|F 8316-41					
☑ **1.8**	gi|30059908	1469	46317	38 (26)	22 (18)	3.25
	H38 0|H38|P9b					
	▸**1 sameset of gi|30059908**					
☑ **1.9**	gi|30059910	1178	44239	28 (19)	14 (12)	1.94
	H39 0|H39|E3a					
☑ **1.10**	gi|88683097	935	55289	27 (18)	14 (11)	1.25
	H44 0|H44|0					
☑ **1.11**	gi|33590227	900	44094	22 (18)	13 (12)	1.75
	H42 NM|O157:NM(H42)|25					
☑ **1.12**	gi|6009849	704	58493	28 (14)	16 (8)	0.73
	H49 0|H49|U11a-44					
☑ **1.13**	gi|16901498	704	56006	25 (14)	14 (8)	0.77
	H34 0|H34|0					
	▸**3 samesets of gi|16901498**					
☑ **1.14**	gi|192955539	697	68106	30 (15)	17 (9)	0.68
	H9 0|O111:H9|E110019 [gi|193071144|ref|ZP_03052068| H9 0|O111:H9|E110019]					
	▸**2 samesets of gi|192955539**					
☑ **1.15**	gi|121144335	459	62285	23 (10)	14 (7)	0.51
	H55 0|O75:H55|E2987-73					

Score Mass Matches Sequences emPAI

▼248 peptide matches (150 non-duplicate, 98 duplicate)

Query	Dupes	Observed	Mr(expt)	Mr(calc)	Delta	M	Score	Expect	Rank	U	1	2	3	4	5	6	7	8	9	10	11	12	13	14	15	Peptide
10		313.1631	624.3116	624.3119	-0.0002	0	9	0.11	▶1	U	■															K.SIDYK.G
17	▶3	316.6896	631.3646	631.3653	-0.0007	0	25	0.029	▶1		■	■	■		■	■	■	■	■	■	■	■	■	■	■	R.LSSGLR.I
22		318.1911	634.3676	635.3643	-0.9966	0	2	0.61	▶1	U														■		K.LAGFTK.G
56	▶1	347.2000	692.3854	692.3857	-0.0003	0	26	0.0065	▶1		■	■	■				■									R.FTANIK.G
80		366.7035	731.3924	731.3926	-0.0001	0	34	0.0015	▶1		■	■	■		■		■									K.GLTQASR.N
82		370.7035	739.3924	739.3938	-0.0014	0	26	0.003	▶1		■	■		■												K.VMYLSK.S
88		378.7010	755.3874	755.3887	-0.0013	0	38	0.00021	▶1		■	■		■												K.VMYLSK.S + Oxidation (M)
106		387.7025	773.3904	773.3919	-0.0015	0	36	0.00058	▶1		■	■		■												R.LEEIDR.V
109		390.7158	779.4170	779.4178	-0.0007	0	25	0.0034	▶1	U	■															K.GITFTNK.S
184		418.2371	834.4596	834.4600	-0.0003	0	48	1.5e-05	▶1	U								■								K.AFVSVQGK.S
196	▶1	423.2215	844.4284	844.4402	-0.0118	0	15	0.032	▶1	U												■				K.AAAGAESIR.Y
225		430.7084	859.4022	860.4240	-1.0217	0	7	0.21	▶1	U			■													K.VELGGSDGK.T
226		431.2632	860.5118	860.4967	0.0151	1	1	0.72	▶1	U			■													K.EVTTVKGK.G
272		446.2422	890.4698	890.4709	-0.0011	0	53	1.7e-05	▶1		■	■		■												K.ATGSDLISK.F
330	▶2	466.2509	930.4872	930.4883	-0.0010	0	58	7.6e-06	▶1							■	■	■	■	■				■	■	R.SSLGAVQNR
363	▶1	473.2532	944.4918	944.5039	-0.0121	0	8	0.51	▶1													■	■			R.SSLGAIQNR.L
366		474.2556	946.4966	946.4971	-0.0005	0	50	9.8e-06	▶1	U	■															K.SIDATELAK.L
397		480.2484	958.4822	958.4832	-0.0010	0	69	2.3e-07	▶1		■	■		■												R.SDLGAVQNR.F
397		480.2484	958.4822	958.5196	-0.0373	0	32	0.0011	▶2	U											■					R.SSLGVVQNR.L
416	▶3	484.2266	966.4386	966.4407	-0.0020	0	64	4.3e-07	▶1	U	■															K.SGTAFDANGK.G
431		487.2563	972.4980	972.4988	-0.0008	0	32	0.0007	▶1	U					■											R.SDLGAIQNR.F
431		487.2563	972.4980	971.5148	0.9832	0	32	0.00072	▶2	U			■													R.SNLGAIQNR.F
461	▶1	493.2869	984.5592	984.5604	-0.0011	0	73	5.5e-08	▶1	U	■															K.LAINLADQK.S
466		493.7539	985.4932	985.5556	-0.0624	0	6	0.27	▶1	U															■	K.AAASNVLAAAK.N
504	▶1	502.2394	1002.4642	1002.5094	-0.0452	1	10	0.51	▶1									■	■	■		■	■	■	■	K.SRLDEIDR.V
523		505.7531	1009.4916	1009.4928	-0.0011	0	38	0.00017	▶1	U					■											K.SSITTESGTK.I
599	▶1	524.2597	1046.5048	1046.5066	-0.0018	0	39	0.00023	▶1		■	■		■												K.VLAENNEMK.I
617	▶2	528.2535	1054.4924	1053.5091	0.9834	0	48	2.3e-05	▶1	U	■															K.NSAGQFTTTK.V
617	▶2	528.2535	1054.4924	1054.5295	-0.0370	0	38	0.00022	▶2			■		■												K.NSAGLYTTTK.V
629		531.2820	1060.5494	1060.5513	-0.0018	0	56	2.7e-06	▶1	U					■											K.GSVNNTVATAK.D
630		532.2570	1062.4994	1062.5015	-0.0021	0	37	0.00043	▶1		■	■		■												K.VLAENNEMK.I + Oxidation (M)
658		540.3488	1078.6830	1077.4873	1.1958	0	9	0.13	▶1	U							■									K.NDGSAQAIMR.E + Oxidation (M)
677	▶1	544.2849	1086.5552	1086.5557	-0.0005	0	65	3e-07	▶1	U	■															K.TVTDTTPGAPK.V
705	▶2	551.2666	1100.5186	1100.5210	-0.0024	0	74	3.5e-07	▶1		■	■	■		■	■	■	■	■	■	■	■	■	■	■	K.DDAAGQAIANR.F
770		568.2590	1134.5034	1134.5517	-0.0482	0	4	0.63	▶1			■		■												K.TITETASGNNK.V
773		380.2114	1137.6124	1137.5150	0.0974	0	8	0.15	▶1	U							■									K.DTTDATGTAGTK.V
797		382.5580	1144.6522	1144.6564	-0.0042	1	3	4.9	▶1		■	■	■		■	■	■	■	■	■	■	■	■	■	■	R.LSSGLRINSAK.D
819		576.7980	1151.5814	1151.5822	-0.0008	0	34	0.00043	▶1	U					■											K.ATLNGSEAYVK.G
840		582.2988	1162.5830	1163.5935	-1.0105	0	4	1.3	▶1				■		■											R.VSGQTQFNGVK.V
843	▶4	582.7957	1163.5768	1163.5782	-0.0014	0	62	2.1e-06	▶1		■	■	■		■	■	■									K.SQSSLSSAIER.L
888		598.8013	1195.5880	1194.5517	1.0364	0	2	0.56	▶1	U												■				K.DAAQSSIDFGGK.K
915		606.8638	1211.7130	1211.5856	0.1274	1	1	0.82	▶1	U													■			K.ADGMVKDGYIK.G + Oxidation (M
938		407.5501	1219.6285	1220.6150	-0.9865	0	4	0.37	▶1	U							■									R.VSNQTQFNGVK.V
957	▶3	618.8142	1235.6138	1235.6146	-0.0008	0	77	4.5e-08	▶1		■	■		■												R.VSEQTQFNGVK.V
957	▶1	618.8142	1235.6138	1234.6306	0.9832	0	41	0.00019	▶2	U						■										R.VSQQTQFNGVK.V
978		415.8510	1244.5312	1244.6361	-0.1049	1	0	0.91	▶1	U														■		K.EQKVNISQDGK.I
979		623.3303	1244.6460	1244.6361	0.0100	1	15	0.029	▶1	U														■		K.EQKVNISQDGK.I
1065	▶2	651.8485	1301.6824	1301.6827	-0.0002	0	104	9e-11	▶1		■	■		■												K.AATLSDLDLNAAK.K
1111	▶1	665.3537	1328.6928	1328.6936	-0.0008	0	68	1.4e-07	▶1	U	■															K.GVLTANIDGQDVK.F
1130		672.8765	1343.7384	1343.7408	-0.0024	0	77	2.1e-08	▶1	U															■	-.SLSLITQNNINK.N
1149		683.3363	1364.6580	1364.6572	0.0008	0	67	2.2e-07	▶1	U				■												R.FDSAITNLGNTVN.-
1215		712.8856	1423.7566	1423.7671	-0.0104	1	1	0.86	▶1	U							■									K.VYTANITNKTATK.G
1227		715.8940	1429.7734	1429.7776	-0.0042	1	109	2e-11	▶1		■	■		■												K.AATLSDLDLNAAKK.T
1228		477.5990	1429.7752	1429.7776	-0.0024	1	14	0.067	▶1		■	■		■												K.AATLSDLDLNAAKK.T
1251	▶2	724.8726	1447.7306	1447.7307	-0.0001	0	87	4e-09	▶1		■	■		■												K.TLGLDGFNIDGAQK.A
1262		728.9086	1455.8026	1455.8045	-0.0019	0	107	3.1e-11	▶1	U								■								K.AQIIQQAGNSVLSK.A
1263		729.4049	1456.7952	1456.6583	0.1370	0	2	1	▶1	U												■				K.YEFAGGNSTNGGGVK.F
1302	▶3	743.8721	1485.7296	1485.7311	-0.0014	0	74	6.3e-08	▶1		■	■		■												K.SEGGSPILVNEDAAK.S
1312		747.9177	1493.8208	1493.8202	0.0007	0	74	2.2e-07	▶1	U								■	■	■	■	■	■	■		K.ANQVPQQVLSLxQG.-
1358		508.8995	1523.6767	1522.7515	0.9252	0	1	1.5	▶1	U														■		K.ITAADDNATLYLDK.Q
1360		508.9261	1523.7565	1523.7580	-0.0015	1	17	0.031	▶1	U	■															K.NSAGQFTTTKVENK.A
1361		762.8857	1523.7568	1523.7580	-0.0011	1	82	1.1e-08	▶1	U	■															K.NSAGQFTTTKVENK.A
1390	▶1	773.9014	1545.7882	1545.7886	-0.0004	0	87	3.5e-09	▶1		■	■		■												K.SLQSTTNPLETIDK.A
1399		778.9089	1555.8032	1555.8067	-0.0034	1	41	0.00013	▶1		■	■		■												K.VDNLRSDLGAVQNR.F
1400		519.6089	1555.8049	1555.8067	-0.0018	1	40	0.00016	▶1		■	■		■												K.VDNLRSDLGAVQNR.F
1408		781.4205	1560.8264	1560.8260	0.0004	0	66	1.2e-06	▶1									■	■	■	■	■	■		■	R.VSGQTQFNGVNVLAK
1478		807.9125	1613.8104	1613.8121	-0.0017	1	91	7.7e-09	▶1		■	■	■		■	■	■	■	■	■	■	■	■	■	■	R.INSAKDDAAGQAIANR.F
1479		538.9443	1613.8111	1613.8121	-0.0010	1	41	0.00068	▶1		■	■	■		■	■	■	■	■	■	■	■	■	■	■	R.INSAKDDAAGQAIANR.F
1524		823.9089	1645.8032	1644.9046	0.8986	1	14	0.038	▶1	U													■			K.TTANTAAKGSDILAALK.T
1555		836.3806	1670.7466	1670.7457	0.0009	0	111	5.1e-11	▶1									■	■	■	■	■	■	■	■	R.IQDADYATEVSNMSK.A
1560		838.8945	1675.7744	1676.8370	-1.0625	0	1	0.73	▶1	U														■		K.IDSDTLNLAGFNVNGK.G
1567		561.0881	1680.2425	1678.8526	1.3899	0	2	0.68	▶1	U															■	K.IDSSTLNLTGFNVNGK.G
1583		847.1066	1692.1986	1692.8418	-0.6431	0	0	0.95	▶1	U														■		K.LTTEATTASSSTADPLK.A
1588	▶1	850.8741	1699.7336	1699.7359	-0.0023	0	146	4.4e-15	▶1		■	■	■		■		■									R.IEDADYATEVSNMSR.A
1590		567.5862	1699.7368	1699.7359	0.0009	0	73	8.7e-08	▶1		■	■	■		■		■									R.IEDADYATEVSNMSR.A
1610		858.8716	1715.7286	1715.7308	-0.0022	0	120	2e-12	▶1		■	■	■		■		■									R.IEDADYATEVSNMSR.A + Oxidatic
1612		573.4127	1717.2163	1717.8734	-0.6571	0	1	1.6	▶1	U															■	K.LTTEASTAAETTANPLK.A
1615		860.3558	1718.6970	1718.7974	-0.1003	0	1	0.79	▶1	U							■									K.ALAYNDAPMSVYFGGK.N + Oxidati
1631	▶11	863.9701	1725.9256	1725.9261	-0.0004	0	115	3.4e-12	▶1	U	■															K.IQVGANDGEAITINLAK.I

Query	Dupes	Observed	Mr(expt)	Mr(calc)	Delta	M	Score	Expect	Rank	U	1	2	3	4	5	6	7	8	9	10	11	12	13	14	15	Peptide
1634	2	576.3160	1725.9262	1725.9261	0.0001	0	43	5.3e-05	1	U	■															K.IQVGANDGEAITINLAK.I
1675	1	875.9514	1749.8882	1749.8897	-0.0015	0	92	6.2e-10	1	U									■							K.VTSDANGAAVGYVTIQGK.N
1739	7	900.9279	1799.8412	1799.8425	-0.0013	0	132	1.1e-13	1	U	■															K.DVFVSAADGSLTTSSDTK.V
1740		600.9547	1799.8423	1799.8425	-0.0003	0	16	0.047	1	U	■															K.DVFVSAADGSLTTSSDTK.V
1745		600.9557	1799.8453	1798.9425	0.9028	0	0	1.8	1	U								■								K.IQVGANDGQTISIDLQK.I
1747	2	900.9304	1799.8462	1798.9425	0.9038	0	56	4.4e-06	2	U								■								K.IQVGANDGQTISIDLQK.I
1759		603.6529	1807.9369	1807.9390	-0.0021	1	37	0.00026	1	U	■															K.TVTDTTPGAPKVMYLSK.S
1764		605.1151	1812.3235	1812.9945	-0.6711	1	6	0.66	1													■	■			K.IQVGANDGQTITIDLKK.I
1807		618.4436	1852.3090	1851.9731	0.3359	1	1	1.1	1	U								■								K.AFVSVQGKSFGIDDAALK.N
1808	2	927.4659	1852.9172	1852.9167	0.0006	0	105	5.4e-11	1	U	■															K.TGSTLVVNGATYNVSADGK.T
1808	2	927.4659	1852.9172	1853.9007	-0.9835	0	78	3.2e-08	2			■		■												K.TGSTLVVNGATYDVSADGK.T
1809	1	618.6470	1852.9192	1852.9167	0.0025	0	68	3.2e-07	1	U	■															K.TGSTLVVNGATYNVSADGK.T
1809	1	618.6470	1852.9192	1853.9007	-0.9815	0	36	0.0005	2			■		■												K.TGSTLVVNGATYDVSADGK.T
1872		953.4640	1904.9134	1905.9466	-1.0331	1	2	0.64	1	U															■	K.VKDMTITSAGGNAQVATDK.A
1878		638.6898	1913.0476	1913.0469	0.0007	1	47	2.3e-05	1	U	■															K.SIDATELAKLAINLADQK.S
1881		639.1381	1914.3925	1914.0132	0.3793	1	0	0.97	1	U							■									K.VPMSSAVALKSEAAPDLTK.V
1894		965.5285	1929.0424	1929.0418	0.0006	1	105	7.6e-11	1		■	■		■												K.SLQSTTNPLETIDKALAK.V
1895	1	644.0217	1929.0433	1929.0418	0.0014	1	32	0.0015	1		■	■		■												K.SLQSTTNPLETIDKALAK.V
1915		972.4410	1942.8674	1942.8690	-0.0016	1	79	2.3e-08	1		■	■	■		■		■									R.SRIEDADYATEVSNMSR.A
1916		648.6302	1942.8688	1942.8690	-0.0003	1	65	5.2e-07	1		■	■	■		■		■									R.SRIEDADYATEVSNMSR.A
1928		980.4390	1958.8634	1958.8640	-0.0005	1	60	2.4e-06	1		■	■	■		■		■									R.SRIEDADYATEVSNMSR.A + Oxidat
1928		980.4390	1958.8634	1958.8640	-0.0005	1	11	0.18	2	U						■										R.SRIEDSDYATEVSNMSR.A
1929		653.9619	1958.8639	1958.8640	-0.0001	1	62	1.4e-06	1		■	■	■		■		■									R.SRIEDADYATEVSNMSR.A + Oxidat
1940		492.7139	1966.8265	1966.0007	0.8258	1	14	0.044	1	U														■		K.GSVANTAATSDDLKLAGFTK.G
1956	2	991.5118	1981.0090	1981.0116	-0.0026	1	115	5.1e-12	1	U	■															K.KTGSTLVVNGATYNVSADGK.T
1956	2	991.5118	1981.0090	1981.9957	-0.9866	1	96	3.8e-10	2			■		■												K.KTGSTLVVNGATYDVSADGK.T
1962	2	661.6736	1981.9990	1981.0116	0.9873	1	58	3.3e-06	1	U	■															K.KTGSTLVVNGATYNVSADGK.T
1962	2	661.6736	1981.9990	1981.9957	0.0033	1	45	6e-05	2			■		■												K.KTGSTLVVNGATYDVSADGK.T
1970		664.6715	1990.9927	1990.9960	-0.0033	1	47	3.4e-05	1		■	■		■												R.LEEIDRVSEQTQFNGVK.V
1971		996.5043	1990.9940	1990.9960	-0.0019	1	78	2.5e-08	1		■	■		■												R.LEEIDRVSEQTQFNGVK.V
1976	3	997.5012	1992.9878	1992.9865	0.0014	0	151	1.9e-15	1		■	■	■			■	■									R.FDSAITNLGNTVNNLSSAR.S
1977		665.3377	1992.9913	1992.9865	0.0048	0	79	3.4e-08	1		■	■	■			■	■									R.FDSAITNLGNTVNNLSSAR.S
1981		666.7966	1997.3680	1996.0815	1.2865	1	5	0.29	1	U												■				K.AAAGAESIRYIYLLQMVK.L
2023		679.1506	2034.4300	2034.9543	-0.5243	1	4	0.41	1	U							■									K.MGKALAYNDAPMSVYFGGK.N + Oxi
2058	1	1043.0680	2084.1214	2084.1225	-0.0011	0	119	8.8e-12	1		■	■	■		■	■		■	■	■		■	■	■		M.AQVINTNSLSLiTQNNiNK.N
2058	1	1043.0680	2084.1214	2085.1066	-0.9851	0	85	2e-08	4	U											■					M.AQVINTNSLSLITQNNIDK.N
2060	1	695.7155	2084.1247	2084.1225	0.0021	0	78	9.4e-08	1		■	■	■		■	■		■	■	■		■	■	■		M.AQVINTNSLSLiTQNNiNK.N
2060	1	695.7155	2084.1247	2085.1066	-0.9819	0	63	3e-06	4	U											■					M.AQVINTNSLSLITQNNIDK.N
2128		741.0401	2220.0985	2220.0982	0.0003	0	52	2.5e-05	1									■	■	■						R.LSSAVTNLNNTTTNLSEAQSR.I
2129		1111.0570	2220.0994	2220.0982	0.0013	0	118	6.3e-12	1									■	■	■						R.LSSAVTNLNNTTTNLSEAQSR.I
2202		592.5775	2366.2809	2365.0744	1.2065	0	2	0.64	2	U														■		K.SNFTIDMGGTGSVTYTVSNGDVK.A +
2205	3	1195.0430	2388.0714	2388.0718	-0.0003	0	130	1.2e-13	1		■	■		■												K.ATGTDNYDVGGDAYTVNVDSGAVK.D
2207		797.0323	2388.0751	2388.0718	0.0033	0	91	9.6e-10	1		■	■		■												K.ATGTDNYDVGGDAYTVNVDSGAVK.D
2208		1199.1400	2396.2654	2396.2659	-0.0005	0	103	5.5e-11	1	U							■									R.AQILQQAGTSVLAQANQTTQNVL.-
2209		799.7632	2396.2678	2396.2659	0.0018	0	40	0.0001	1	U							■									R.AQILQQAGTSVLAQANQTTQNVL.-
2243		1276.6140	2551.2134	2551.2137	-0.0003	0	144	5.7e-15	1									■	■		■					R.ELTVQATTGTNSDSDLSSIQDEIK.S
2254		1322.1520	2642.2894	2642.2896	-0.0001	0	43	7.7e-05	1	U					■	■		■			■					R.NANDGISiAQTTEGALSEINNNLQR.V
2255	2	1328.6500	2655.2854	2655.2848	0.0006	0	143	1.2e-14	1		■		■													R.NANDGISVAQTTEGALNEINNNLQR.I
2255	2	1328.6500	2655.2854	2656.2437	-0.9582	0	143	1.2e-14	2	U		■														R.NANDGNSVAQTTEGALNEINNNLQR.V
2256		886.1030	2655.2872	2655.2848	0.0024	0	86	6.1e-09	1		■		■													R.NANDGISVAQTTEGALNEINNNLQR.I
2256		886.1030	2655.2872	2656.2437	-0.9565	0	64	1e-06	2	U		■														R.NANDGNSVAQTTEGALNEINNNLQR.V
2264		888.7524	2663.2354	2663.2351	0.0002	1	66	2.9e-07	1		■	■		■												K.FKATGTDNYDVGGDAYTVNVDSGAVK.
2284		919.7928	2756.3566	2756.3577	-0.0011	0	78	1.7e-08	1	U								■								K.NNTGDATATQPGTSGTTVVAASIHLST
2285	2	1382.6630	2763.3114	2763.3159	-0.0044	0	146	3e-15	1	U	■															R.ELSVQATNGTNSDSDLSSIQAEITQR.
2286	3	922.1122	2763.3148	2763.3159	-0.0011	0	87	2.8e-09	1	U	■															R.ELSVQATNGTNSDSDLSSIQAEITQR.
2293		928.1129	2781.3169	2781.3192	-0.0024	0	12	0.06	1	U								■								K.NSVDADVTASTEFTGASTNDPLTLLDK
2303		940.0819	2817.2239	2816.3424	0.8815	0	5	0.3	1	U					■											R.ELSVQATNGTNSPSDLDSIQNEITQR.
2304		1433.7960	2865.5774	2865.5672	0.0102	0	134	4e-14	1		■		■		■	■										R.AQILQQAGTSVLAQANQTTQNVLSLLR
2305		956.2014	2865.5824	2865.5672	0.0152	0	112	6.8e-12	1		■		■		■	■										R.AQILQQAGTSVLAQANQTTQNVLSLLR
2310		966.8276	2897.4610	2897.4591	0.0019	1	51	3.8e-05	1	U					■	■		■			■					R.NANDGISiAQTTEGALSEINNNLQRVR
2310		966.8276	2897.4610	2897.4591	0.0019	1	11	0.38	3								■			■		■	■	■	■	R.NANDGISVAQTTEGALSEINNNLQRIR
2315		978.2135	2931.6187	2931.6142	0.0045	1	49	1.4e-05	1	U								■								K.AQIIQQAGNSVLSKANQVPQQVLSLLQ
2315		978.2135	2931.6187	2931.5778	0.0409	1	15	0.04	2	U										■						K.AQIIQQAGNSVLAKANQVPQQVLSLEQ
2317		978.8273	2933.4601	2933.4591	0.0010	1	76	4e-08	1		■	■														R.SDLGAVQNRFDSAITNLGNTVNNLSSA
2330		1011.8410	3032.5012	3032.5010	0.0001	1	82	1.1e-08	1	U	■															R.IRELSVQATNGTNSDSDLSSIQAEITQ
2330		1011.8410	3032.5012	3032.5010	0.0001	1	82	1.2e-08	2			■		■												R.VRELTVQATNGTNSDSDLSSIQAEITQ
2350		1077.5710	3229.6912	3229.6902	0.0010	1	114	5.5e-12	1		■	■	■		■	■										M.AQVINTNSLSLLTQNNLNKSQSSLSA
2351		808.4304	3229.6925	3229.6902	0.0023	1	25	0.0044	1		■	■	■		■	■										M.AQVINTNSLSLLTQNNLNKSQSSLSA
2358		1091.2460	3270.7162	3270.7167	-0.0006	1	110	1.5e-11	1									■	■							M.AQVINTNSLSLITQNNINKNQSALSTS
2358		1091.2460	3270.7162	3271.7008	-0.9846	1	106	3.4e-11	2	U											■					M.AQVINTNSLSLITQNNIDKNQSALSTS
2359		1097.2320	3288.6742	3288.6732	0.0010	1	72	5.9e-08	1	U										■						M.AQVINTNSLSLITQNNINKNQSSMSTA

70 subsets and intersections (152 subset proteins in total)

2	gi|112820172	222	H21 0|EHEC serogroup: O113:H21|0
3	gi|307553085	57	Hxx(H54 27.9%) 0|0|ABU 83972

10 per page 1

Not what you expected? Try the select summary.

Mascot: http://www.matrixscience.com/
