# Supplementary material for: MS-H: A Novel Proteomic Approach to Isolate and Type the E. coli H Antigen Using Membrane Filtration and Liquid Chromatography-Tandem Mass Spectrometry (LC-MS/MS)
Source: PLoS One. 2013 Feb 21;8(2):e57339. doi: 10.1371/journal.pone.0057339 (PMC3578835; doi:10.1371/journal.pone.0057339)
Supplement: Representative Peptide Data S1 — Peptide data are represented as the Mascot search results from all 53 serotypes, obtained under the Orbitrap platform in Table 4 with related E. coli reference strains. “U” denotes a unique peptide specific for each of the proteins 1.1, 1.2, and beyond. The number 1.1 (shown as 1 in the peptide list and phylogenetic tree) represents the protein which obtained the highest score and confidence value after a Mascot search. This protein, known as the first hit, was used to designate the MS-H type of the unknown flagellin. Related peptides 1.2 (2), 1.3 (3), etc. represented the second, third, etc. hits for MS-H typing analysis. (DOCX) [file pone.0057339.s009.docx › H41-E209.pdf]

MASCOT Search Results

User :
E-mail :
Search title : Submitted from 20110822-0611 by Mascot Daemon on VARIABLE
MS data file : C:\Documents and Settings\keding\Desktop\Raw data\20110823-001-0031-00611\20110823-003-EC209MS1-RP.RAW
Database : Flagellin_v2 (192 sequences; 89,845 residues)
Taxonomy : Bacteria (Eubacteria) (192 sequences)
Timestamp : 24 Aug 2011 at 18:01:50 GMT

Not what you expected? Try **the select summary**.

▸ **Search parameters**
▸ **Score distribution**
▸ **Legend**

Protein Family Summary

Significance threshold p< 0.05 Max. number of families AUTO
Ions score or expect cut-off 0 Dendrograms cut at 0

Protein families 1–2 (out of 2)

10 per page 1

▾**1**

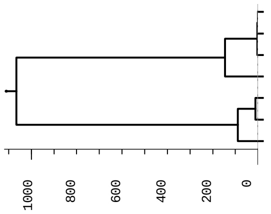

1	gi|30059912	2027	H41 0|H41|RVC 1787
6	gi|323174561	479	Hxx(H25 99.7%) 0|0|LT-68
2	gi|157081065	1306	Hxx(H28 99.7%) 0|0|E24377A [gi|157159035|ref|YP_001463228| Hxx 0|0|E24377A]
4	gi|33590227	714	H42 NM|O157:NM(H42)|25
7	gi|154710451	408	H36 0|H36|0
3	gi|124516981	1297	H40 0|O79:H40|E49
5	gi|46093545	597	H3 0|0|Bi7327-41

Threshold (**0**): 0

		Score	Mass	Matches	Sequences	emPAI
1.1	gi|30059912<br>H41 0|H41|RVC 1787<br>▸1 sameset of gi|30059912	2027	57346	46 (34)	35 (30)	5.66
1.2	gi|157081065<br>Hxx(H28 99.7%) 0|0|E24377A [gi|157159035|ref|YP_001463228| Hxx 0|0|E24377A]<br>▸3 samesets of gi|157081065	1306	59373	35 (23)	26 (19)	2.46
1.3	gi|124516981<br>H40 0|O79:H40|E49	1297	51573	36 (24)	28 (23)	3.42
1.4	gi|33590227<br>H42 NM|O157:NM(H42)|25	714	44094	21 (14)	17 (12)	1.75
1.5	gi|46093545<br>H3 0|0|Bi7327-41	597	55534	28 (10)	21 (9)	0.78
1.6	gi|323174561<br>Hxx(H25 99.7%) 0|0|LT-68<br>▸1 sameset of gi|323174561	479	46392	22 (11)	16 (10)	1.13
1.7	gi|154710451<br>H36 0|H36|0	408	57784	19 (8)	14 (7)	0.56

▾**106 peptide matches (93 non-duplicate, 13 duplicate)**

Query	Dupes	Observed	Mr(expt)	Mr(calc)	Delta	M	Score	Expect	Rank	U	1	2	3	4	5	6	7	Peptide
20	▸1	308.2083	614.4020	615.3591	-0.9571	0	23	0.0092	▸1	U					■			K.NLEIK.Q
32	▸1	315.7011	629.3876	629.3860	0.0016	1	12	0.056	▸1	U					■			K.VDKLR.S
38	▸1	316.6910	631.3674	631.3653	0.0021	0	23	0.052	▸1		■	■	■	■	■	■	■	R.LSSGLR.I
80		337.2166	672.4186	673.3759	-0.9572	0	4	0.41	▸1	U						■		K.NGATALK.L
102	▸1	347.2011	692.3876	692.3857	0.0019	0	13	0.11	▸1	U			■					R.FTANIK.G
113		352.2038	702.3930	702.3912	0.0019	0	1	1.3	▸1	U						■		K.AIASVDK.F
121		355.1986	708.3826	708.3806	0.0020	0	16	0.18	▸1		■	■		■	■	■	■	R.FTSNIK.G
123		358.7074	715.4002	715.3977	0.0026	0	32	0.0045	▸1		■	■		■		■		K.GLTQAAR.N
136		366.2192	730.4238	730.4225	0.0014	0	3	1.7	▸1	U					■			K.LDTALAK.V
137	▸1	366.7047	731.3948	731.3926	0.0023	0	21	0.03	▸1				■				■	K.GLTQASR.N
137		366.7047	731.3948	731.3813	0.0135	0	2	2.2	▸2	U					■			R.LSEIDR.V
165	▸1	380.6960	759.3774	759.3763	0.0012	0	38	0.0009	▸1		■	■				■		R.LDEIDR.V
184		388.2201	774.4256	773.3919	1.0337	0	13	0.12	▸1	U			■					R.LEEIDR.V
383	▸1	452.2613	902.5080	902.5073	0.0008	0	16	0.028	▸1	U		■						K.AATLDALTK.N
432		466.2407	930.4668	930.4658	0.0010	0	51	3.5e-05	▸1	U	■							K.SEATADPLK.A
433	▸1	466.2519	930.4892	930.4883	0.0010	0	75	1.4e-07	▸1		■	■			■	■		R.SSLGAVQNR
456		468.8455	935.6764	934.4356	1.2408	0	1	0.84	▸1	U		■						K.DGTVTNGSGK.A
472		473.7458	945.4770	944.5179	0.9592	0	0	2.8	▸2	U					■			K.IDADTLGLK.D
474		474.2559	946.4972	946.4971	0.0001	0	14	0.04	▸1	U			■					K.SIDATELAK.L
500		480.2382	958.4618	958.5196	-0.0577	1	19	0.022	▸1	U				■				K.SRLAEIDR.V
501	▸1	480.2490	958.4834	958.4832	0.0002	0	65	6.3e-07	▸1	U			■					R.SDLGAVQNR.F

Query	Dupes	Observed	Mr(expt)	Mr(calc)	Delta	M	Score	Expect	Rank	U	1	2	3	4	5	6	7	Peptide
501		480.2490	958.4834	958.5196	-0.0361	0	20	0.019	▶2	U				■				R.SSLGVVQNR.L
523		484.2276	966.4406	966.4407	-0.0000	0	57	2.2e-06	▶1	U			■					K.SGTAFDANGK.G
562		493.2876	984.5606	984.5604	0.0003	0	60	9.2e-07	▶1	U			■					K.LAINLADQK.S
598		502.2623	1002.5100	1002.5094	0.0006	1	35	0.0018	▶1		■	■				■		K.SRLDEIDR.V
643		511.3660	1020.7174	1019.5400	1.1775	0	4	0.57	▶1	U				■				K.AIAQVDTFR.S
693		524.2608	1046.5070	1046.5066	0.0004	0	23	0.0096	▶1	U			■					K.VLAENNEMK.I
695		525.2596	1048.5046	1048.5036	0.0010	0	89	1.3e-09	▶1	U	■							K.AADGSLTSEAK.G
703		526.7725	1051.5304	1051.5298	0.0006	0	60	1.1e-06	▶1	U	■							K.GYLGTGANTAK.I
708		527.7618	1053.5090	1053.5091	-0.0000	0	49	1.9e-05	▶1	U			■					K.NSAGQFTTTK.V
787		544.2853	1086.5560	1086.5557	0.0003	0	58	1.5e-06	▶1	U			■					K.TVTDTTPGAPK.V
810	▶1	551.2682	1100.5218	1100.5210	0.0008	0	78	1.4e-07	▶1		■	■	■	■	■	■	■	K.DDAAGQAIANR.F
834		558.7992	1115.5838	1115.5822	0.0016	1	43	5e-05	▶1	U	■							K.GKSEATADPLK.A
889		382.5601	1144.6585	1144.6564	0.0021	1	11	0.68	▶1		■	■	■	■	■	■	■	R.LSSGLRINSAK.D
908		384.5424	1150.6054	1151.5492	-0.9438	0	2	0.65	▶1	U		■						R.MSAESLQSATK.S
927		581.3041	1160.5936	1160.5925	0.0012	0	104	5.2e-11	▶1		■	■						K.ALDEAISSIDK.F
931		582.2415	1162.4684	1163.5935	-1.1251	0	1	2.5	▶2	U							■	R.VSGQTQFNGVK.V
936	▶1	582.7968	1163.5790	1163.5782	0.0008	0	59	4.2e-06	▶1				■		■		■	K.SQSSLSSAIER.L
982		596.3024	1190.5902	1190.5891	0.0012	0	77	1.1e-07	▶1		■	■						K.NQSALSSSIER.L
998		600.8538	1199.6930	1199.6734	0.0196	1	12	0.067	▶1	U					■			K.LRSSLGAVQNR.F
1071		618.8150	1235.6154	1235.6146	0.0008	0	70	2.5e-07	▶1	U			■					R.VSEQTQFNGVK.V
1071		618.8150	1235.6154	1234.6306	0.9848	0	45	7.6e-05	▶2	U					■			R.VSQQTQFNGVK.V
1165		646.3432	1290.6718	1290.6568	0.0150	0	14	0.042	▶1	U						■		K.DGAAQGFVTLQGK.N
1166		431.2319	1290.6739	1290.6568	0.0170	0	4	0.38	▶1	U						■		K.DGAAQGFVTLQGK.N
1185	▶1	651.8499	1301.6852	1301.6827	0.0026	0	57	4.8e-06	▶1	U			■					K.AATLSDLDLNAAK.K
1232		665.3546	1328.6946	1328.6936	0.0010	0	53	5.3e-06	▶1	U			■					K.GVLTANIDGQDVK.F
1392		720.9132	1439.8118	1439.8096	0.0022	0	108	6.7e-11	▶1		■	■		■				K.AQIIQQAGNSVLAK.A
1408		724.8736	1447.7326	1447.7307	0.0019	0	68	3.7e-07	▶1	U			■					K.TLGLDGFNIDGAQK.A
1423		729.8942	1457.7738	1457.7726	0.0013	0	115	3.3e-12	▶1	U	■							K.ISIGGTEQEVNIAK.D
1431		488.9290	1463.7652	1463.7620	0.0032	1	41	9.9e-05	▶1		■	■						K.ALDEAISSIDKFR.S
1462		743.8732	1485.7318	1485.7311	0.0008	0	79	2e-08	▶1	U			■					K.SEGGSPILVNEDAAK.S
1474		747.9191	1493.8236	1493.8202	0.0035	0	61	4.8e-06	▶1		■	■		■		■		K.ANQVPQQVLSLLQG.-
1547		773.9026	1545.7906	1545.7886	0.0020	0	78	3.3e-08	▶1	U			■					K.SLQSTTNPLETIDK.A
1569		781.4222	1560.8298	1560.8260	0.0038	0	79	6.6e-08	▶1		■	■		■				R.VSGQTQFNGVNVLAK
1636		538.9449	1613.8129	1613.8121	0.0008	1	22	0.062	▶1		■	■	■	■	■	■	■	R.INSAKDDAAGQAIANR.F
1664		818.9147	1635.8148	1635.8104	0.0044	0	72	6.8e-08	▶1	U	■							K.IDSDTLGLSGFNVNGK.G
1695		553.3054	1656.8944	1657.8523	-0.9579	0	0	0.98	▶1	U				■				K.VDLDAAATDIGTALGQK.V
1718		836.3810	1670.7474	1670.7457	0.0017	0	122	4.2e-12	▶1		■	■		■		■		R.IQDADYATEVSNMSK.A
1792		573.4144	1717.2214	1715.7308	1.4906	0	2	1.3	▶1				■				■	R.IEDADYATEVSNMSR.A + Oxidation (M)
1812		863.9733	1725.9320	1725.9261	0.0060	0	71	7.3e-08	▶1	U			■					K.IQVGANDGEAITINLAK.I
1817		578.0859	1731.2359	1731.7257	-0.4899	0	2	0.75	▶2	U					■			R.IEDSDYATEVSNMSR.A + Oxidation (M)
1842		583.2934	1746.8584	1746.9516	-0.0932	1	1	0.72	▶1	U					■			K.DFSVASAKVPTSGAVALK.S
1860		880.9564	1759.8982	1759.8087	0.0895	0	2	0.57	▶1	U					■			K.ALSFNDSQMSVYVDGK.N
1863	▶1	588.9779	1763.9119	1763.9054	0.0065	1	43	5e-05	▶1	U	■							K.KIDSDTLGLSGFNVNGK.G
1863		588.9779	1763.9119	1762.9101	1.0017	0	9	0.12	▶2	U							■	K.EITAETLGLTGFNVNGK.G
1913		900.9306	1799.8466	1799.8425	0.0041	0	114	6.8e-12	▶1	U			■					K.DVFVSAADGSLTTSSDTK.V
1920		602.3173	1803.9301	1803.9438	-0.0138	1	0	5.4	▶1		■	■						K.NQSALSSSIERLSSGLR.I
1929		605.1157	1812.3253	1812.9945	-0.6693	1	1	2.3	▶1		■	■						K.IQVGANDGQTITIDLKK.I
2028		634.3192	1899.9358	1899.9326	0.0031	0	11	0.083	▶1	U	■							K.SYSFNANGLTGANTATALK.G
2029		950.9769	1899.9392	1899.9326	0.0066	0	75	2.9e-08	▶1	U	■							K.SYSFNANGLTGANTATALK.G
2066		647.0020	1937.9842	1937.9807	0.0035	0	25	0.0034	▶1	U							■	K.ASVEINGSSQAVIIDHNGK.M
2086		653.6696	1957.9870	1957.9844	0.0026	0	9	0.26	▶1	U	■							K.STTTNYSAATTADLLSTIK.D
2087		980.0010	1957.9874	1957.9844	0.0030	0	93	9.4e-10	▶1	U	■							K.STTTNYSAATTADLLSTIK.D
2102		659.3043	1974.8911	1975.0586	-0.1675	1	5	0.31	▶1	U							■	K.IAIAGSTDGTTAGKISATNVK.I
2123		997.5029	1992.9912	1992.9865	0.0048	0	127	4.7e-13	▶1				■		■			R.FDSAITNLGNTVNNLSSAR.S
2191		695.7154	2084.1244	2084.1225	0.0018	0	66	1.5e-06	▶1		■	■	■		■	■	■	M.AQVINTNSLSLiTQNNiNK.N
2191		695.7154	2084.1244	2085.1066	-0.9822	0	58	1.1e-05	▶5	U				■				M.AQVINTNSLSLITQNNIDK.N
2192		1043.0700	2084.1254	2084.1225	0.0029	0	134	2.5e-13	▶1		■	■	■		■	■	■	M.AQVINTNSLSLiTQNNiNK.N
2192		1043.0700	2084.1254	2085.1066	-0.9811	0	78	9.5e-08	▶4	U				■				M.AQVINTNSLSLITQNNIDK.N
2199		700.6556	2098.9450	2098.0906	0.8544	1	0	0.96	▶1	U						■		K.LGVSDTSSLSLHNILDKDGK.A
2277		1125.0560	2248.0974	2248.0931	0.0043	0	124	2.2e-12	▶1		■	■		■				R.LDSAVTNLNNTTTNLSEAQSR.I
2278		750.3732	2248.0978	2248.0931	0.0047	0	80	5.5e-08	▶1		■	■		■				R.LDSAVTNLNNTTTNLSEAQSR.I
2322		1195.0450	2388.0754	2388.0718	0.0037	0	105	3.2e-11	▶1	U			■					K.ATGTDNYDVGGDAYTVNVDSGAVK.D
2352		641.8026	2563.1813	2562.3211	0.8602	1	1	0.82	▶1	U					■			K.VPTSGAVALKSEMSPTLTSVNATTGK.N + Oxidation (M)
2354		867.0703	2598.1891	2598.1834	0.0056	0	92	6.4e-10	▶1	U	■							K.ATEADLTGAGFSQGAVDTNGNSTWTK.S
2362		1315.1470	2628.2794	2628.2739	0.0055	0	111	3.5e-11	▶1		■	■						R.NANDGISVAQTTEGALSEINNNLQR.I
2363		877.1012	2628.2818	2628.2739	0.0079	0	66	1.1e-06	▶1		■	■						R.NANDGISVAQTTEGALSEINNNLQR.I
2385		930.7697	2789.2873	2789.2780	0.0092	0	71	8.3e-08	▶1	U	■							K.LTTSASNTVTQTYHEFANGNIYDDK.G
2405		1031.8580	3092.5522	3092.5448	0.0074	1	86	1e-08	▶1		■	■		■				R.IQDADYATEVSNMSKAQIIQQAGNSVLAK.A
2412		1077.5730	3229.6972	3229.6902	0.0070	1	132	9.1e-14	▶1				■		■		■	M.AQVINTNSLSLLTQNNLNKSQSSLSSAIER.L
2413		815.1839	3256.7065	3256.7011	0.0054	1	49	4.6e-05	▶1		■	■						M.AQVINTNSLSLITQNNINKNQSALSSSIER.L
2414		1086.5770	3256.7092	3256.7011	0.0081	1	120	3.8e-12	▶1		■	■						M.AQVINTNSLSLITQNNINKNQSALSSSIER.L
2431		1159.8850	3476.6332	3476.6220	0.0112	0	86	2.2e-09	▶1	U	■							K.AYTVANGAESYDVAAVTGAVTTTAGNSPVYADADGK.L

▶78 subsets and intersections (161 subset proteins in total)

▶2 gi|307553085 13 Hxx(H54 27.9%) 0|0|ABU 83972

10 per page 1

Not what you expected? Try the select summary.

Mascot: http://www.matrixscience.com/
