# Supplementary material for: MS-H: A Novel Proteomic Approach to Isolate and Type the E. coli H Antigen Using Membrane Filtration and Liquid Chromatography-Tandem Mass Spectrometry (LC-MS/MS)
Source: PLoS One. 2013 Feb 21;8(2):e57339. doi: 10.1371/journal.pone.0057339 (PMC3578835; doi:10.1371/journal.pone.0057339)
Supplement: Representative Peptide Data S1 — Peptide data are represented as the Mascot search results from all 53 serotypes, obtained under the Orbitrap platform in Table 4 with related E. coli reference strains. “U” denotes a unique peptide specific for each of the proteins 1.1, 1.2, and beyond. The number 1.1 (shown as 1 in the peptide list and phylogenetic tree) represents the protein which obtained the highest score and confidence value after a Mascot search. This protein, known as the first hit, was used to designate the MS-H type of the unknown flagellin. Related peptides 1.2 (2), 1.3 (3), etc. represented the second, third, etc. hits for MS-H typing analysis. (DOCX) [file pone.0057339.s009.docx › H42-E210M.pdf]

MASCOT Search Results

User : keding
E-mail : chengkeding@yahoo.com
Search title : flagellin
MS data file : C:\Xcalibur\data\20110921-00646\E210M1-MS1.RAW
Database : Flagellin_v2 (192 sequences; 89,845 residues)
Taxonomy : Bacteria (Eubacteria) (192 sequences)
Timestamp : 23 Sep 2011 at 16:18:53 GMT

Not what you expected? Try **the select summary**.

Search parameters

Score distribution

Legend

Protein Family Summary

Significance threshold p< 0.05 Max. number of families AUTO
Ions score or expect cut-off 0 Dendrograms cut at 0

Protein families 1–5 (out of 5)

10 per page 1

1

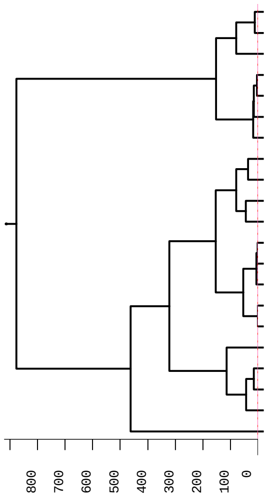

**1**	**gi|33590227**	2679	H42 NM|O157:NM(H42)|25
**21**	**gi|40353143**	196	H2 0|O111:H2|5721/96
**15**	**gi|30577587**	829	H17 0|O15:H17|P12b
**6**	**gi|33590260**	1260	H29 0|O157:H29|Y63
**18**	**gi|154710451**	522	H36 0|H36|0
**16**	**gi|46093545**	629	H3 0|0|Bi7327-41
**11**	**gi|323174561**	1195	Hxx(H25 99.7%) 0|0|LT-68
**2**	**gi|145997**	1935	Hxx(H12 99.7%) 0|0|Bi 316-42 (Orskov)
**20**	**gi|23477612**	418	H2 0|O128:H2|0
**10**	**gi|6941886**	1210	H43 0|O157:H43|O157:H43 [gi|30059916|ref|AAP13330| H43 0|H43|149-51]
**12**	**gi|30059928**	1070	H52 0|H52|C2187-69
**3**	**gi|6009831**	1720	Hxx(H1 100.0%) 0|0|Su 1242 [gi|312946515|ref|ADR27342| H1 0|O83:H1|NRG 857C] [gi|3075...
**8**	**gi|6009849**	1240	H49 0|H49|U11a-44
**7**	**gi|16901498**	1245	H34 0|H34|0
**5**	**gi|6941882**	1283	H14 0|O138:H14|O138:H14
**14**	**gi|30059862**	908	H10 0|H10|Bi 623-42
**13**	**gi|121144335**	981	H55 0|O75:H55|E2987-73
**19**	**gi|33590243**	476	H16 NM|O157:NM(H16)|PC02
**9**	**gi|125659369**	1233	H44 0|H44|781-55
**17**	**gi|154710444**	523	H35 0|H35|0
**4**	**gi|5107847**	1402	H12 0|O111:H12|DEC 6a

Threshold (**0**): 0

		Score	Mass	Matches	Sequences	emPAI
**1.1**	**gi|33590227**	2679	44094	52 (42)	30 (24)	11.55
	H42 NM|O157:NM(H42)|25					
**1.2**	**gi|145997**	1935	61008	56 (34)	35 (27)	4.09
	Hxx(H12 99.7%) 0|0|Bi 316-42 (Orskov)					
**1.3**	**gi|6009831**	1720	60901	51 (33)	31 (27)	3.84
	Hxx(H1 100.0%) 0|0|Su 1242 [gi|312946515|ref|ADR27342| H1 0|O83:H1|NRG 857C] [gi|307553937|ref|ADN46712| Hxx 0|0|ABU 83972] [gi|301050721|ref|ZP_07197581| Hxx 0|0|MS 185-1] [gi|30...					
**1.4**	**gi|5107847**	1402	57823	50 (28)	33 (24)	3.22
	H12 0|O111:H12|DEC 6a					
**1.5**	**gi|6941882**	1283	56492	64 (25)	40 (18)	2.28
	H14 0|O138:H14|O138:H14					
**1.6**	**gi|33590260**	1260	45720	40 (23)	22 (14)	2.51
	H29 0|O157:H29|Y63					
**1.7**	**gi|16901498**	1245	56006	41 (24)	23 (18)	2.31
	H34 0|H34|0					
	**3 samesets of gi|16901498**					
**1.8**	**gi|6009849**	1240	58493	41 (24)	24 (18)	2.16
	H49 0|H49|U11a-44					
**1.9**	**gi|125659369**	1233	58912	40 (24)	23 (17)	1.96
	H44 0|H44|781-55					
**1.10**	**gi|6941886**	1210	51071	33 (22)	20 (14)	2.08
	H43 0|O157:H43|O157:H43 [gi|30059916|ref|AAP13330| H43 0|H43|149-51]					
**1.11**	**gi|323174561**	1195	46392	45 (21)	25 (13)	2.22
	Hxx(H25 99.7%) 0|0|LT-68					
**1.12**	**gi|30059928**	1070	46003	36 (22)	19 (15)	2.49
	H52 0|H52|C2187-69					
**1.13**	**gi|121144335**	981	62285	40 (19)	24 (14)	1.28
	H55 0|O75:H55|E2987-73					
**1.14**	**gi|30059862**	908	44249	32 (18)	18 (13)	1.94

		Score	Mass	Matches	Sequences	emPAI
	H10 0|H10|Bi 623-42					
	▶2 samesets of gi|30059862					
☑ 1.15	gi|30577587	829	36285	29 (18)	16 (11)	2.40
	H17 0|O15:H17|P12b					
☑ 1.16	gi|46093545	629	55534	31 (12)	20 (7)	0.78
	H3 0|0|Bi7327-41					
☑ 1.17	gi|154710444	523	52714	31 (13)	20 (9)	0.95
	H35 0|H35|0					
☑ 1.18	gi|154710451	522	57784	26 (10)	18 (6)	0.65
	H36 0|H36|0					
☑ 1.19	gi|33590243	476	55093	23 (12)	16 (8)	0.79
	H16 NM|O157:NM(H16)|PC02					
	▶2 samesets of gi|33590243					
☑ 1.20	gi|23477612	418	51966	25 (9)	17 (6)	0.64
	H2 0|O128:H2|0					
☑ 1.21	gi|40353143	196	47290	19 (7)	13 (5)	0.50
	H2 0|O111:H2|5721/96					

▼272 peptide matches (217 non-duplicate, 55 duplicate)

Query	Dupes	Observed	Mr(expt)	Mr(calc)	Delta	M	Score	Expect	Rank	U	1	2	3	4	5	6	7	8	9	10	11	12	13	14	15	16	17	18	19	20	21	Peptide
4	▶1	301.6693	601.3240	601.3183	0.0057	0	12	0.12	▶1	U				■																		-.NNINK.N
5		302.1709	602.3272	601.3911	0.9361	1	8	0.21	▶2	U																■						K.TVVRK.D
14		308.1948	614.3750	615.3591	-0.9841	0	6	0.43	▶2	U																■						K.NLEIK.Q
23		315.6947	629.3748	629.3860	-0.0112	1	1	0.87	▶1																	■			■			K.VDKLR.S
26	▶1	316.6894	631.3642	631.3653	-0.0011	0	28	0.014	▶1		■	■	■	■	■	■	■	■	■	■	■	■	■	■	■	■	■	■	■	■	■	R.LSSGLR.I
31		318.6893	635.3640	635.2949	0.0692	0	2	0.6	▶1	U							■															K.ADGMVK.D + Oxi
34		323.1895	644.3644	645.3697	-1.0053	0	2	0.67	▶2	U																	■					K.TLGLDK.L
35		324.1658	646.3170	646.3438	-0.0268	0	17	0.018	▶1	U																	■					K.YHISK.D
35		324.1658	646.3170	647.3490	-1.0320	0	5	0.34	▶2	U																■						K.VTVDSK.A
55	▶3	337.2160	672.4174	673.3759	-0.9584	0	31	0.00084	▶1	U											■											K.NGATALK.L
72	▶1	344.2044	686.3942	686.3963	-0.0020	0	18	0.017	▶1			■	■	■																		K.ALDQLK.D
88		351.7052	701.3958	700.4119	0.9839	1	4	0.61	▶1	U																				■		K.DPTKLK.A
92	▶1	352.2020	702.3894	703.3501	-0.9606	0	2	0.85	▶1	U																■						K.AVNTGDK.T
94		352.2030	702.3914	702.3912	0.0003	0	6	0.39	▶1							■					■											K.AIASVDK.F
100		355.1977	708.3808	708.3806	0.0002	0	19	0.079	▶1		■	■	■	■	■	■	■	■	■	■	■	■	■	■	■	■	■	■				R.FTSNIK.G
103		358.7004	715.3862	715.3864	-0.0002	0	36	0.0019	▶1	U	■																					R.LAEIDR.V
104		358.7054	715.3962	715.3977	-0.0014	0	29	0.009	▶1		■	■	■	■	■	■	■	■	■	■	■	■	■	■	■							K.GLTQAAR.N
105	▶1	358.7132	715.4118	716.4068	-0.9950	0	12	0.44	▶2	U														■								K.LDASALK.A
106	▶1	358.7185	715.4224	715.4228	-0.0004	0	17	0.13	▶1			■		■																		K.ITIGGQK.A
107	▶1	358.7185	715.4224	715.3864	0.0360	0	13	0.38	▶1	U																				■		K.LVDANGK.D
134		378.7158	755.4170	755.3887	0.0283	0	1	0.96	▶1	U																					■	K.VMYLSK.S + Oxi
136	▶1	379.7122	757.4098	757.4698	-0.0599	1	6	0.23	▶1	U																		■				K.LDKALAK.V
137		380.2028	758.3910	758.4174	-0.0263	0	27	0.011	▶1	U																			■			K.LDEALAK.V
140	▶2	380.6949	759.3752	759.3763	-0.0010	0	34	0.0023	▶1			■	■	■	■	■	■	■	■		■	■	■		■							R.LDEIDR.V
149		387.2015	772.3884	771.4490	0.9394	0	10	0.25	▶1	U															■							K.ALDAAIAK.V
150		387.6994	773.3842	772.4079	0.9764	0	4	0.92	▶1	U																		■				R.LEEINR.V
150		387.6994	773.3842	772.4079	0.9764	0	1	1.8	▶2	U																	■					R.LQEIDR.V
150		387.6994	773.3842	773.3919	-0.0077	0	0	2.2	▶3											■				■							■	R.LEEIDR.V
161		393.2125	784.4104	785.3708	-0.9603	0	9	0.13	▶1	U																			■			K.AFGSNYK.N
164		393.7571	785.4996	786.4599	-0.9603	0	12	0.067	▶1	U											■											K.QTGQLIK.V
177		398.6735	795.3324	795.3988	-0.0663	0	2	0.59	▶1	U																	■					K.DVHVGGGR.V
187		402.1917	802.3688	801.4708	0.8980	1	2	0.61	▶1			■		■																		K.VATAKQGK.A
190		403.1996	804.3846	804.3977	-0.0131	0	1	0.79	▶1	U					■																	K.ITASNGDK.L
208	▶1	409.2130	816.4114	816.4090	0.0025	0	4	0.42	▶1	U																				■		K.GTTTPGQR.D
255		423.2211	844.4276	844.4402	-0.0126	0	14	0.038	▶1	U								■														K.AAAGAESIR.Y
278	▶1	430.2648	858.5150	859.4399	-0.9249	0	7	0.21	▶1	U													■									K.AQDVNVSK.D
280		431.2335	860.4524	860.4240	0.0285	0	15	0.032	▶1	U																				■		K.VELGGSDGK.T
281		431.2625	860.5104	860.4603	0.0501	0	9	0.12	▶1	U					■																	K.AATTADALK.A
281		431.2625	860.5104	860.4967	0.0137	1	5	0.32	▶2	U																				■		K.EVTTVKGK.G
294	▶1	436.2649	870.5152	870.5538	-0.0386	1	7	0.18	▶1	U																					■	K.LAIKLADK.G
313		439.2780	876.5414	876.4552	0.0862	0	4	0.41	▶1	U					■																	K.AATTADSLK.A
324		445.7570	889.4994	888.4916	1.0078	0	4	1.5	▶1	U					■																	K.AATTADVLK.A
326		447.7059	893.3972	892.4290	0.9682	0	2	0.59	▶1	U					■																	K.VDQAAFDK.A
337		452.2412	902.4678	901.4181	1.0497	0	13	0.047	▶1	U														■								K.YYVNDTK.S
337	▶1	452.2412	902.4678	902.5073	-0.0394	0	4	0.46	▶4	U					■																	K.AATTADILK.A
340		452.7332	903.4518	904.4502	-0.9983	0	5	0.45	▶3	U					■																	K.AATTADDLK.A
369		461.7472	921.4798	920.4637	1.0161	0	1	0.79	▶4	U					■																	K.AATTADMLK.A
380	▶2	466.2505	930.4864	930.4883	-0.0018	0	67	8.2e-07	▶1						■	■			■	■	■	■	■	■	■	■	■		■			R.SSLGAVQNR
391		468.7493	935.4840	936.4586	-0.9746	0	5	0.34	▶1	U					■																	K.AATTADMLK.A +
409	▶1	473.2578	944.5010	944.5039	-0.0029	0	74	1.3e-07	▶1				■	■			■	■														R.SSLGAIQNR.L
409		473.2578	944.5010	945.5243	-1.0233	1	4	1.1	▶2	U					■																	K.AATTADRLK.A
423		476.7465	951.4784	952.4865	-1.0081	0	5	0.35	▶1	U					■																	K.AATTADYLK.A
434		480.2654	958.5162	958.5196	-0.0033	0	5	0.62	▶1	U	■																					R.SSLGVVQNR.L
467		487.7594	973.5042	973.5444	-0.0401	1	14	0.057	▶1	U																		■				K.ISAEDLKAK.A
478		490.2442	978.4738	978.4771	-0.0032	0	60	1.2e-06	▶1			■		■																		K.AGDGQSIGFK.K
480		491.2330	980.4514	980.4563	-0.0049	0	22	0.0056	▶1			■	■	■																		K.YSIDANNGK.V
495		494.7670	987.5194	988.5189	-0.9995	0	16	0.026	▶1	U										■												K.TNLVTAADGK.T
523	▶1	502.2387	1002.4628	1002.5094	-0.0466	1	2	0.82	▶1			■	■	■	■	■	■	■	■		■	■	■		■							K.SRLDEIDR.V
555		510.7699	1019.5252	1020.5274	-1.0021	0	8	0.23	▶1	U																■						K.VLATNQTMK.I +
555		510.7699	1019.5252	1019.5400	-0.0147	0	7	0.31	▶2	U	■																					K.AIAQVDTFR.S
555		510.7699	1019.5252	1020.5274	-1.0021	0	1	1.2	▶3	U																	■					K.VLASQQTMK.I +

Query	Dupes	Observed	Mr(expt)	Mr(calc)	Delta	M	Score	Expect	Rank	U	1	2	3	4	5	6	7	8	9	10	11	12	13	14	15	16	17	18	19	20	21	Peptide
558	▶1	511.2609	1020.5072	1020.5088	-0.0015	0	55	5.2e-06	▶1			■	■	■																			K.VTVDSGTGTK.Y
595		519.7564	1037.4982	1037.5029	-0.0047	0	30	0.00094	▶1	U	■																					K.NYEITDGVK.N
620		528.2573	1054.5000	1053.5091	0.9910	0	15	0.048	▶1	U																					■	K.NSAGQFTTTK.V
624		528.8036	1055.5926	1056.5451	-0.9525	0	7	0.18	▶1	U																	■					K.GDQLTADPLK.S
625	▶2	529.3017	1056.5888	1056.5927	-0.0039	0	24	0.0037	▶1	U																					■	-.LLTQNNLNK.S
627	▶2	529.7855	1057.5564	1058.5972	-1.0407	1	12	0.06	▶1	U														■								R.DVKLDASALK.A
634		531.2932	1060.5718	1060.5764	-0.0046	0	59	1.2e-06	▶1			■	■	■																			K.AATISDLTAAK.M
634	▶1	531.2932	1060.5718	1060.5513	0.0206	0	17	0.022	▶2	U																		■				K.GSVNNTVATAK.D
654	▶1	537.7828	1073.5510	1073.5651	-0.0141	1	5	0.31	▶1	U																	■					R.VMAANDIKGR.T
654		537.7828	1073.5510	1074.5306	-0.9795	0	1	0.84	▶2	U																			■			K.VNATDGSVGGAK.A
659		539.2681	1076.5216	1077.4873	-0.9656	0	22	0.008	▶1	U																			■			K.NDGSAQAIMR.E +
691		546.8040	1091.5934	1092.5200	-0.9265	0	16	0.024	▶1	U			■																				K.AGDGQSIGFNK.T
712	▶1	551.2653	1100.5160	1100.5210	-0.0050	0	64	3.3e-06	▶1		■	■	■	■	■	■	■	■	■	■	■	■	■	■	■	■	■	■	■	■	■	K.DDAAGQAIANR.F
715		552.2897	1102.5648	1103.6339	-1.0690	1	5	0.33	▶1	U																				■		K.LFKLVDANGK.D
719	▶2	554.2734	1106.5322	1106.5356	-0.0034	0	28	0.0014	▶1	U																■						K.AGAFAAQTADGK.S
735		557.8162	1113.6178	1113.6393	-0.0215	1	5	0.34	▶1	U															■							K.ALDAAIAKVDK.F
749		562.2910	1122.5674	1122.5921	-0.0246	1	7	0.2	▶2	U																					■	K.LADKGSIEYK.G
760		376.8752	1127.6038	1128.6503	-1.0465	1	2	0.65	▶1	U																	■					K.TLGLDKLDVR.N
768		567.2730	1132.5314	1133.5564	-1.0250	1	12	0.058	▶1	U																		■				K.DADGKITTDAK.T
772		379.1755	1134.5047	1134.5591	-0.0544	1	10	0.15	▶1	U																		■				K.TTDPMAKLDK.A +
782		570.2813	1138.5480	1137.5666	0.9814	0	1	0.74	▶1	U											■												K.ATADYVVQSGK.D
826		579.7852	1157.5558	1156.6451	0.9107	0	11	0.071	▶1	U																		■				K.AATLENLALNK.T
837		387.8958	1160.6656	1160.5925	0.0731	0	1	0.92	▶1			■	■	■	■																		K.ALDEAISSIDK.F
879		595.3268	1188.6390	1187.6034	1.0357	0	2	0.56	▶2	U													■									K.ALDDAISQIDK.F
883	▶1	596.2999	1190.5852	1190.5891	-0.0038	0	35	0.0017	▶1			■	■	■	■		■	■		■		■		■								K.NQSALSSSIER.L
896		600.8518	1199.6890	1199.6734	0.0156	1	12	0.069	▶1																		■			■			K.LRSSLGAVQNR.F
905		603.3072	1204.5998	1204.6048	-0.0049	0	54	7.8e-06	▶1		■					■					■				■							K.NQSALSTSIER.L
913		606.8632	1211.7118	1211.5856	0.1262	1	4	0.43	▶1	U							■															K.ADGMVKDGYIK.G
922	▶1	406.2027	1215.5863	1216.6663	-1.0800	1	9	0.14	▶1	U																	■					K.EINSKTLGLDK.L
927	▶1	609.7834	1217.5522	1217.5888	-0.0366	0	14	0.044	▶1	U						■																R.VTIDGDGTNQAK.I
941		409.2123	1224.6151	1224.5986	0.0164	0	1	0.81	▶1	U						■																K.SYAASVDAGGTVK.
943		613.4324	1224.8502	1224.5986	0.2516	0	3	0.49	▶1	U						■																K.SYAASVDAGGTVK.
957		617.8176	1233.6206	1233.6354	-0.0147	1	7	0.73	▶1	U														■								K.QVAVGAGDFKDK.A
959		620.3156	1238.6166	1237.5761	1.0406	0	17	0.021	▶1	U												■										K.FGANDTAAAAMAK.
960		620.3619	1238.7092	1238.5561	0.1532	0	1	0.71	▶1										■				■									K.NQSSMSTAIER.L
982		624.3707	1246.7268	1245.6565	1.0704	0	8	0.36	▶1	U										■												K.TALAAAGADTSGLK
985		627.8018	1253.5890	1253.5710	0.0180	0	9	0.14	▶1	U												■										K.FGANDTAAAAMAK.
985		627.8018	1253.5890	1254.6244	-1.0354	0	2	0.64	▶2	U								■														K.FNALDAATAFSK.L
990		419.2128	1254.6166	1253.5710	1.0456	0	6	0.28	▶1	U												■										K.FGANDTAAAAMAK.
1035		641.3073	1280.6000	1279.7136	0.8865	1	0	1.7	▶1	U					■																	K.LYIDTTGRLTK.N
1039		427.8745	1280.6017	1279.7136	0.8881	1	0	1.5	▶2	U					■																	K.LYIDTTGRLTK.N
1055		648.2828	1294.5510	1294.6041	-0.0531	0	3	0.46	▶1	U																				■		K.DTGSFALIGDDGK.
1057		432.8784	1295.6134	1294.6041	1.0092	0	8	0.17	▶1	U																				■		K.DTGSFALIGDDGK.
1072		651.8608	1301.7070	1301.6827	0.0244	0	5	0.67	▶2	U																					■	K.AATLSDLDLNAAK.
1086		656.8622	1311.7098	1311.7146	-0.0048	0	44	3.6e-05	▶1	U				■																		K.AQIIQQAGNSVLA.
1108		440.8717	1319.5933	1318.6616	0.9317	0	4	0.36	▶1	U																					■	K.VSGESIDATELAK.
1118		332.1554	1324.5925	1325.6615	-1.0690	0	1	0.85	▶2	U											■												K.DFYAASVNAALGK.
1119		663.4515	1324.8884	1325.6615	-0.7731	0	6	0.25	▶1	U											■												K.DFYAASVNAALGK.
1144	▶1	448.2471	1341.7195	1341.7252	-0.0057	0	4	0.43	▶1	U					■																	K.ADLVAANATVVGNK
1151	▶1	672.8757	1343.7368	1343.7408	-0.0040	0	68	1.5e-07	▶1	U													■									-.SLSLITQNNINK.N
1211		697.3250	1392.6354	1391.6205	1.0150	0	10	0.1	▶1	U													■									K.ASDSYSFSATAASK
1276		720.9100	1439.8054	1439.8096	-0.0042	0	107	8.4e-11	▶1		■	■	■		■		■	■	■				■	■								K.AQIIQQAGNSVLAK
1278		480.9425	1439.8057	1439.8096	-0.0039	0	5	1.4	▶1		■	■	■		■		■	■	■				■	■								K.AQIIQQAGNSVLAK
1289		725.8469	1449.6792	1449.7463	-0.0671	1	1	0.86	▶1	U									■														K.ALDDAISSIDKFR.
1300		728.3494	1454.6842	1454.6889	-0.0046	0	55	4.5e-06	▶1	U				■																		K.AAAADGDTSATITY
1303		729.4025	1456.7904	1456.6583	0.1322	0	0	1.6	▶1	U								■														K.YEFAGGNSTNGGGV
1307		731.4036	1460.7926	1461.8038	-1.0112	1	5	0.35	▶2	U																				■		K.SLKTDSIAAISTQK
1336		741.9094	1481.8042	1482.7355	-0.9312	0	14	0.044	▶1	U																	■					K.NLTFTDGATAPTFK
1347	▶1	747.3467	1492.6788	1492.6794	-0.0006	0	114	2.4e-11	▶1			■	■	■																			K.AASGEVNFDVDANG
1349	▶2	747.9161	1493.8176	1493.8202	-0.0025	0	70	5.8e-07	▶1		■	■	■		■	■	■	■	■	■	■	■		■	■							K.ANQVPQQVLSLLQG
1350		747.9164	1493.8182	1493.7474	0.0708	1	3	3.1	▶5	U																			■			K.QNSTGYEKVQVGGK
1354	▶1	750.3616	1498.7086	1498.7151	-0.0065	0	100	1e-10	▶1	U	■																					K.TDITYSGGDITGAT
1390		762.9201	1523.8256	1523.8307	-0.0051	0	77	3.5e-08	▶1	U	■																					K.VNASSLTLHNILDK
1390		762.9201	1523.8256	1523.7580	0.0677	1	1	1.5	▶2	U																					■	K.NSAGQFTTTKVENK
1391		508.9492	1523.8258	1523.8307	-0.0050	0	41	0.00015	▶1	U	■																					K.VNASSLTLHNILDK
1400		766.8825	1531.7504	1530.7777	0.9727	0	12	0.069	▶1	U												■										K.IQLTDELDVDGSVK
1433	▶1	781.4171	1560.8196	1560.8260	-0.0064	0	84	1.8e-08	▶1		■	■	■	■	■	■	■	■	■	■		■	■										R.VSGQTQFNGVNVLA
1482		803.5942	1605.1738	1604.7868	0.3870	1	3	0.49	▶1			■		■																			K.KTASVTMGGTTYNF
1488		538.9429	1613.8069	1613.8121	-0.0052	1	40	0.00094	▶1		■	■	■	■	■	■	■	■	■	■	■	■	■	■	■	■	■	■	■	■	■	R.INSAKDDAAGQAIA
1489		807.9109	1613.8072	1613.8121	-0.0049	1	89	1.2e-08	▶1		■	■	■	■	■	■	■	■	■	■	■	■	■	■	■	■	■	■	■	■	■	R.INSAKDDAAGQAIA
1510		820.3982	1638.7818	1637.8308	0.9511	1	7	0.21	▶1	U																	■					K.DVHVGGGRVMAAND
1514		822.9223	1643.8300	1643.8367	-0.0066	0	145	3e-15	▶1	U	■																					K.LSDTVTQVGDGSAA
1516	▶1	823.9035	1645.7924	1644.9046	0.8878	1	19	0.012	▶1	U							■															K.TTANTAAKGSDILA
1523		827.4237	1652.8328	1651.8781	0.9547	1	1	0.74	▶1	U											■												K.KIDSSTLGLNGFSV
1526		553.6234	1657.8484	1657.8523	-0.0039	0	65	3.1e-07	▶1	U	■																					K.VDLDAAATDIGTAL
1527	▶1	829.9329	1657.8512	1657.8523	-0.0010	0	101	7.9e-11	▶1	U	■																					K.VDLDAAATDIGTAL
1542		836.3776	1670.7406	1670.7457	-0.0051	0	127	1.3e-12	▶1		■	■	■	■	■	■	■	■	■	■	■	■	■	■	■							R.IQDADYATEVSNMS
1560	▶2	843.4567	1684.8988	1685.8836	-0.9847	0	97	8.1e-10	▶1														■				■					K.IQVGANDGETITID
1560	▶2	843.4567	1684.8988	1684.8996	-0.0007	0	82	2.3e-08	▶2			■	■	■	■		■	■	■													K.IQVGANDGQTITID
1562		843.9736	1685.9326	1686.8828	-0.9502	1	7	1.8	▶2	U																				■		K.LKAATAETTYFGST
1567	▶1	846.3984	1690.7822	1690.7686	0.0136	0	2	0.69	▶2	U					■																	K.SYSFDTTTASAADV
1570		564.6022	1690.7848	1690.7686	0.0162	0	9	0.12	▶1	U					■																	K.SYSFDTTTASAADV
1573		847.6201	1693.2256	1691.8478	1.3778	1	1	0.81	▶1	U						■																K.LNKADVTYNDAANG

Query	Dupes	Observed	Mr(expt)	Mr(calc)	Delta	M	Score	Expect	Rank	U	1	2	3	4	5	6	7	8	9	10	11	12	13	14	15	16	17	18	19	20	21	Peptide
1576		848.6102	1695.2058	1694.7748	0.4311	1	2	0.66	1	U								■														K.AADDKDAAQSSIDF
1598		858.3698	1714.7250	1715.7308	-1.0058	0	2	1.3	1																			■	■	■		R.IEDADYATEVSNMS
1605		860.1302	1718.2458	1718.7974	-0.5515	0	1	0.85	1	U																			■			K.ALAYNDAPMSVYFG
1606		574.2863	1719.8371	1719.8428	-0.0057	0	49	1.2e-05	1	U	■																					K.VAANSDGEAVGFAT
1607	1	860.9264	1719.8382	1719.8428	-0.0045	0	134	3.7e-14	1	U	■																					K.VAANSDGEAVGFAT
1611		862.3939	1722.7732	1723.8741	-1.1008	1	4	0.37	1	U					■																	K.ITASNGDKLYIDTT
1617		577.6248	1729.8526	1730.9051	-1.0525	0	4	0.44	1	U										■												K.VQTATTTPGTAVDV
1624		579.3070	1734.8992	1735.9105	-1.0113	0	2	0.62	1	U												■										K.VTTNSAGAAVGYVT
1629		579.6232	1735.8478	1735.8840	-0.0362	1	9	0.12	1	U					■																	K.LTTDAETKAATTAD
1639		582.9897	1745.9473	1746.9987	-1.0514	0	3	0.46	1	U								■														K.LTLMLMQAVISLLA
1642		583.9830	1748.9272	1748.9309	-0.0037	0	54	4.1e-06	1	U	■																					K.IDSSALGLSGFSVA
1643		875.4709	1748.9272	1748.9309	-0.0036	0	130	1.1e-13	1	U	■																					K.IDSSALGLSGFSVA
1646		876.9225	1751.8304	1751.8611	-0.0307	1	2	0.65	1	U					■																	K.LTTDAETKAATTAD
1685		889.6435	1777.2724	1775.8036	1.4688	0	9	0.31	1	U																■						K.ALSFNDSQMSVYVD
1697		893.9484	1785.8822	1785.9108	-0.0286	1	3	0.64	1	U					■																	K.LTTDAETKAATTAD
1707		599.9363	1796.7871	1795.8873	0.8997	1	8	0.17	1	U					■																	K.LTTDAETKAATTAD
1715		902.9341	1803.8536	1802.9812	0.8725	1	1	4.4	1	U																■						K.GMTTAKPLEKLDTA
1723		605.1135	1812.3187	1811.9153	0.4034	1	0	2.6	2	U					■																	K.LTTDAETKAATTAD
1730		607.3004	1818.8794	1817.9595	0.9199	1	5	0.53	1		■					■					■				■							K.NQSALSTSIERLSS
1755		616.9962	1847.9668	1848.9065	-0.9398	1	3	0.54	1	U													■									K.AQDVNVSKDGTITT
1811		633.8049	1898.3929	1897.9422	0.4507	0	3	0.45	1	U													■									K.LAGFTAGATPAADG
1818	1	953.4623	1904.9100	1905.9466	-1.0365	1	7	0.2	1	U													■									K.VKDMTITSAGGNAQ
1819		636.9846	1907.9320	1908.9065	-0.9745	0	6	0.27	1	U							■															K.IDSDTLGLSGFNVN
1833		964.9683	1927.9220	1927.9276	-0.0055	0	129	2.4e-13	1			■	■	■																		K.SGVQTYQAVFAAGD
1837		969.9731	1937.9316	1937.9364	-0.0048	0	127	1.8e-13	1			■	■	■																		K.MDAATNTITTTNNA
1851		980.4525	1958.8904	1960.0490	-1.1586	1	0	2.1	2	U	■																					K.AIAQVDTFRSSLGV
1858		987.0182	1972.0218	1972.0266	-0.0047	0	55	3.4e-06	1			■	■																			K.AGDVAASLLPPAGQ
1871		668.4872	2002.4398	2001.9280	0.5118	0	1	0.85	1	U																		■				K.ADTAGFTTSTGFTV
1880		504.9618	2015.8181	2015.0422	0.7759	1	6	0.24	1	U									■													K.AATTADPLKALDDA
1891		1021.4690	2040.9234	2040.9236	-0.0002	0	119	1.2e-12	1			■		■																		K.TGADADAATANAGV
1912	1	1043.0660	2084.1174	2084.1225	-0.0051	0	119	7.8e-12	1			■	■		■	■	■	■	■	■	■	■		■		■	■	■	■	■		M.AQVINTNSLSLiTQ
1912	1	1043.0660	2084.1174	2085.1066	-0.9891	0	101	4.9e-10	4	U	■																					M.AQVINTNSLSLITQ
1912	1	1043.0660	2084.1174	2085.0814	-0.9640	0	85	2.3e-08	5	U															■							M.AQVINTNSLSLNTQ
1913		695.7137	2084.1193	2084.1225	-0.0033	0	75	2e-07	1			■	■		■	■	■	■	■	■	■	■		■		■	■	■	■	■		M.AQVINTNSLSLiTQ
1913		695.7137	2084.1193	2085.1066	-0.9873	0	73	3e-07	4	U	■																					M.AQVINTNSLSLITQ
1913		695.7137	2084.1193	2085.0814	-0.9621	0	61	5.7e-06	5	U															■							M.AQVINTNSLSLNTQ
1921		1050.4760	2098.9374	2098.0906	0.8468	1	9	0.14	1	U											■											K.LGVSDTSSLSLHNI
1921		1050.4760	2098.9374	2098.0430	0.8944	1	5	0.3	2	U																				■		K.TEVVTATDGKTYSV
1922		700.6536	2098.9390	2098.0906	0.8484	1	6	0.25	1	U											■											K.LGVSDTSSLSLHNI
1928		705.0798	2112.2176	2111.0317	1.1858	1	4	0.49	1	U											■											R.VSGQTQFNGVNVLS
1966		1093.0440	2184.0734	2184.0798	-0.0063	1	114	7.7e-12	1	U	■																					K.TDITYSGGDITGAT
1967		729.0318	2184.0736	2184.0798	-0.0062	1	50	1.7e-05	1	U	■																					K.TDITYSGGDITGAT
1971		731.0394	2190.0964	2190.0917	0.0047	1	5	0.37	1			■		■																		K.AASGEVNFDVDANG
1976		552.4839	2205.9065	2205.0947	0.8118	1	3	1.1	1	U											■											K.DGSMKIQVGANDGQ
1983		741.0453	2220.1141	2220.0982	0.0159	0	4	1.8	1	U						■																R.LSSAVTNLNNTTTN
1993		746.3715	2236.0927	2236.1196	-0.0269	1	4	0.93	1																	■	■		■	■	■	R.FDSAITNLGNTVNN
1997		1120.0680	2238.1214	2237.1474	0.9740	1	2	0.65	1	U																■						R.VSQQTQFNGVKVLA
2004		750.3680	2248.0822	2248.0931	-0.0109	0	105	2.1e-10	1		■	■	■	■	■		■	■	■	■		■	■		■							R.LDSAVTNLNNTTTN
2005		1125.0500	2248.0854	2248.0931	-0.0077	0	117	1.1e-11	1		■	■	■	■	■		■	■	■	■		■	■		■							R.LDSAVTNLNNTTTN
2012		761.2093	2280.6061	2280.1458	0.4602	1	3	0.92	1	U																		■				R.FDSTITNLGNTVNN
2017		763.0179	2286.0319	2286.0434	-0.0115	1	3	0.46	1	U													■									K.DMTITSAGGNAQVA
2057		810.6354	2428.8844	2430.2577	-1.3733	1	2	0.69	1	U										■												K.SVADNAKPLAALDD
2077		851.4119	2551.2139	2551.2137	0.0002	0	41	0.00013	1		■					■					■											R.ELTVQATTGTNSDS
2078	1	1276.6150	2551.2154	2551.2137	0.0017	0	163	7.9e-17	1		■					■					■											R.ELTVQATTGTNSDS
2079		858.7786	2573.3140	2574.2847	-0.9707	0	6	0.27	1	U																		■				K.VLASDNSMTIQVGA
2094		1322.1500	2642.2854	2642.2896	-0.0041	0	117	3.1e-12	1	U	■					■				■	■					■		■				R.NANDGISiAQTTEG
2094		1322.1500	2642.2854	2642.2896	-0.0041	0	104	6.8e-11	3	U		■																				R.NANDAISVAQTTEG
2095		881.7703	2642.2891	2642.2896	-0.0005	0	94	6.2e-10	1	U	■					■				■	■					■		■				R.NANDGISiAQTTEG
2095		881.7703	2642.2891	2642.2896	-0.0005	0	74	6.1e-08	3	U		■																				R.NANDAISVAQTTEG
2095		881.7703	2642.2891	2641.3559	0.9332	0	17	0.031	4	U									■													K.ADLTAAQLTTTAAG
2096		882.4576	2644.3510	2644.2688	0.0821	0	20	0.0091	1	U																	■					R.NANDGISVAQTTEG
2103		893.4643	2677.3711	2677.3043	0.0668	1	18	0.016	1	U					■																	K.GTITIDGSAQDVQI
2118		966.8264	2897.4574	2897.4591	-0.0017	1	47	0.0001	1				■	■	■		■	■	■			■	■						■			R.NANDGISVAQTTEG
2118		966.8264	2897.4574	2897.4591	-0.0017	1	5	1.5	2	U	■					■				■	■					■		■				R.NANDGISiAQTTEG
2122		975.4962	2923.4668	2923.4662	0.0005	1	30	0.0016	1			■	■	■																		K.VGAEVYVSANGTLT
2131		1031.8550	3092.5432	3092.5448	-0.0016	1	78	7.4e-08	1		■	■	■		■		■	■	■				■	■								R.IQDADYATEVSNMS
2136		1084.1560	3249.4462	3248.6525	0.7937	1	2	0.62	1			■	■	■																		K.IDSDTLGLNGFNVN
2137		1086.5720	3256.6942	3256.7011	-0.0069	1	132	2.8e-13	1			■	■		■		■	■		■		■		■								M.AQVINTNSLSLITQ
2137		1086.5720	3256.6942	3256.7011	-0.0069	1	98	6.9e-10	3	U																				■		M.AQVINTNSLSLLTQ
2143		1095.5660	3283.6762	3283.6784	-0.0022	1	85	2.8e-09	1	U	■																					K.LSDTVTQVGDGSAA
2144		821.9265	3283.6769	3283.6784	-0.0015	1	36	0.00028	1	U	■																					K.LSDTVTQVGDGSAA
2145		1097.2290	3288.6652	3288.6732	-0.0080	1	93	4.5e-10	1	U									■													M.AQVINTNSLSLITQ
2151		1103.9150	3308.7232	3309.6365	-0.9133	1	6	0.26	1	U									■													K.DTNDFTFNATIAAG

56 subsets and intersections (143 subset proteins in total)

2	gi|307553085	98	Hxx(H54 27.9%) 0|0|ABU 83972
3	gi|112820172	20	H21 0|EHEC serogroup: O113:H21|0
4	gi|46093564	18	Hxx(H54 100.0%) 0|0|E223-69 [gi|283982455|ref|ADB56974| H54 0|O161:H54|O161:H54]
5	gi|257752125	14	H11 0|O26:H11|11368 [gi|260853476|ref|YP_003227367| H11 0|O26:H11|11368] [gi|29837897...

10 per page 1

Mascot: http://www.matrixscience.com/
