# Supplementary material for: MS-H: A Novel Proteomic Approach to Isolate and Type the E. coli H Antigen Using Membrane Filtration and Liquid Chromatography-Tandem Mass Spectrometry (LC-MS/MS)
Source: PLoS One. 2013 Feb 21;8(2):e57339. doi: 10.1371/journal.pone.0057339 (PMC3578835; doi:10.1371/journal.pone.0057339)
Supplement: Representative Peptide Data S1 — Peptide data are represented as the Mascot search results from all 53 serotypes, obtained under the Orbitrap platform in Table 4 with related E. coli reference strains. “U” denotes a unique peptide specific for each of the proteins 1.1, 1.2, and beyond. The number 1.1 (shown as 1 in the peptide list and phylogenetic tree) represents the protein which obtained the highest score and confidence value after a Mascot search. This protein, known as the first hit, was used to designate the MS-H type of the unknown flagellin. Related peptides 1.2 (2), 1.3 (3), etc. represented the second, third, etc. hits for MS-H typing analysis. (DOCX) [file pone.0057339.s009.docx › H43-E211.pdf]

MATRIX SCIENCE MASCOT Search Results

User :
E-mail :
Search title : Submitted from 20110822-0608 by Mascot Daemon on VARIABLE
MS data file : C:\Documents and Settings\keding\Desktop\Raw data\20110822-002-0031-00608\20110822-002-EC211MS3.RAW
Database : Flagellin_v2 (192 sequences; 89,845 residues)
Taxonomy : Bacteria (Eubacteria) (192 sequences)
Timestamp : 24 Aug 2011 at 17:03:52 GMT

Not what you expected? Try **the select summary**.

▶ **Search parameters**
▶ **Score distribution**
▶ **Legend**

Protein Family Summary

Significance threshold p< 0.05 Max. number of families AUTO
Ions score or expect cut-off 0 Dendrograms cut at 0

Protein family 1 (out of 1)

10 per page 1

▼1

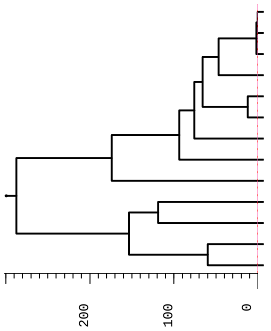

1	gi|6941886	10427	H43 0|O157:H43|O157:H43 [gi|30059916|ref|AAP13330| H43 0|H43|149-51]
4	gi|169754667	1919	Hxx(H3306 97.9%) 0|0|ATCC 8739 [gi|170019739|ref|YP_001724693| Hxx 0|0|ATCC 8739]
3	gi|30059920	1921	H46 0|H46|5306-56
7	gi|88683097	1478	H44 0|H44|0
12	gi|59044663	255	H11 0|O78:H11|ATCC 35401 [gi|59044673|ref|AAW84030| H11 0|O26:H11|88-353] [gi|590446...
13	gi|40353143	221	H2 0|O111:H2|5721/96
11	gi|46093552	754	H53 0|0|E480-68
2	gi|6009851	3238	H51 0|H51|C218-70 [gi|30059926|ref|AAP13335| H51 0|H51|C218-70]
6	gi|33590227	1844	H42 NM|O157:NM(H42)|25
9	gi|37651050	1087	H28 0|O145:H28|0917/99 [gi|37651052|ref|CAD97427| Hxx 0|O145:H-|2032/99]
5	gi|30577587	1873	H17 0|O15:H17|P12b
10	gi|5107855	1017	Hxx(H6 99.6%) 0|0|E2348/69
8	gi|121144335	1404	H55 0|O75:H55|E2987-73

Threshold (**0**): 0

		Score	Mass	Matches	Sequences	emPAI
**1.1**	gi|6941886	10427	51071	177 (165)	41 (40)	53.76
	H43 0|O157:H43|O157:H43 [gi|30059916|ref|AAP13330| H43 0|H43|149-51]					
**1.2**	gi|6009851	3238	61407	55 (53)	17 (15)	2.15
	H51 0|H51|C218-70 [gi|30059926|ref|AAP13335| H51 0|H51|C218-70]					
**1.3**	gi|30059920	1921	57918	46 (40)	14 (13)	2.02
	H46 0|H46|5306-56					
	▶ **1 sameset of gi|30059920**					
**1.4**	gi|169754667	1919	58285	46 (40)	14 (13)	2.00
	Hxx(H3306 97.9%) 0|0|ATCC 8739 [gi|170019739|ref|YP_001724693| Hxx 0|0|ATCC 8739]					
	▶ **1 sameset of gi|169754667**					
**1.5**	gi|30577587	1873	36285	43 (37)	12 (12)	4.26
	H17 0|O15:H17|P12b					
**1.6**	gi|33590227	1844	44094	48 (40)	14 (11)	2.95
	H42 NM|O157:NM(H42)|25					
**1.7**	gi|88683097	1478	55289	38 (32)	11 (11)	1.67
	H44 0|H44|0					
**1.8**	gi|121144335	1404	62285	40 (29)	15 (11)	1.28
	H55 0|O75:H55|E2987-73					
**1.9**	gi|37651050	1087	55672	42 (35)	16 (12)	1.37
	H28 0|O145:H28|0917/99 [gi|37651052|ref|CAD97427| Hxx 0|O145:H-|2032/99]					
**1.10**	gi|5107855	1017	52951	44 (30)	15 (10)	1.33
	Hxx(H6 99.6%) 0|0|E2348/69					
**1.11**	gi|46093552	754	44861	20 (15)	12 (7)	0.90
	H53 0|0|E480-68					
**1.12**	gi|59044663	255	46411	11 (8)	7 (5)	0.51
	H11 0|O78:H11|ATCC 35401 [gi|59044673|ref|AAW84030| H11 0|O26:H11|88-353] [gi|59044679|ref|AAW84033| H11 0|O26:H11|DEC9E] [gi|59044695|ref|AAW84041| H11 0|O111:H11|88-4110] [g...					
	▶ **4 samesets of gi|59044663**					
**1.13**	gi|40353143	221	47290	12 (8)	8 (5)	0.50
	H2 0|O111:H2|5721/96					

▼**249 peptide matches (108 non-duplicate, 141 duplicate)**

Query	Dupes	Observed	Mr(expt)	Mr(calc)	Delta	M	Score	Expect	Rank	U	1 2 3 4 5 6 7 8 9 10 11 12 13	Peptide
21		316.6902	631.3658	631.3653	0.0005	0	25	0.03	▶1		■■■■■■■■■■■■■	R.LSSGLR.I
66		355.1978	708.3810	708.3806	0.0004	0	25	0.023	▶1		■■■■■■■■■■■	R.FTSNIK.G
70		358.7072	715.3998	715.3977	0.0022	0	32	0.0042	▶1		■■■■■■■■■■	K.GLTQAAR.N

Query	Dupes	Observed	Mr(expt)	Mr(calc)	Delta	M	Score	Expect	Rank	U	1	2	3	4	5	6	7	8	9	10	11	12	13	Peptide
71		358.7136	715.4126	715.4116	0.0011	0	12	0.15	1	U											■			K.IDIDLK.K
74		359.2111	716.4076	715.3864	1.0212	0	1	1.5	1							■					■			R.LAEIDR.V
106	1	387.7042	773.3938	773.3919	0.0019	0	36	0.00059	1		■											■	■	R.LEEIDR.V
203		424.7051	847.3956	846.4447	0.9510	0	10	0.11	1	U										■				K.AATIQTDK.G
282		452.3163	902.6180	902.5073	0.1108	0	11	0.082	1	U									■					K.AATLDALTK.N
327	2	466.2519	930.4892	930.4883	0.0010	0	65	1.4e-06	1		■				■		■	■	■	■				R.SSLGAVQNR.L
407		488.7775	975.5404	974.5145	1.0260	1	2	0.71	1	U		■												K.SRLSEIDR.V
433		493.7543	985.4940	985.5556	-0.0616	0	1	0.77	1	U								■						K.AAASNVLAAAK.N
439	7	495.2668	988.5190	988.5189	0.0001	0	76	2.9e-08	1	U	■													K.TNLVTAADGK.T
496		509.2698	1016.5250	1016.5250	0.0000	1	37	0.00018	1	U	■													K.SRLEEIDR.V
497		339.8495	1016.5267	1016.5250	0.0016	1	29	0.0013	1	U	■													K.SRLEEIDR.V
538		520.2650	1038.5154	1037.5029	1.0125	0	1	0.71	1	U						■								K.NYEITDGVK.N
539		347.2059	1038.5959	1037.4778	1.1181	0	14	0.037	1	U		■												K.AVDNGNGTYK.V
576		529.3037	1056.5928	1056.5927	0.0001	0	25	0.003	1	U													■	-.LLTQNNLNK.S
583		532.3525	1062.6904	1062.5015	0.1889	0	5	0.6	1													■	■	K.VLAENNEMK.I + Oxidation (M)
601		537.6998	1073.3850	1073.5717	-0.1866	0	15	0.032	1	U			■											R.ISADALQSAAK.G
607		539.2807	1076.5468	1076.5462	0.0007	0	60	1.5e-06	1	U										■				-.QSALSSSIER.L
657	1	551.2683	1100.5220	1100.5210	0.0010	0	73	5e-07	1		■	■	■	■	■	■	■	■	■	■	■	■	■	K.DDAAGQAIANR.F
688		562.7599	1123.5052	1123.5033	0.0019	0	19	0.014	1	U									■					R.IQDADYATEV.-
747		384.5423	1150.6051	1151.5492	-0.9441	0	2	0.67	1	U									■					R.MSAESLQSATK.S
794	1	397.5589	1189.6549	1190.6507	-0.9958	1	7	1.2	1	U										■				R.DTTKATVTIGGK.D
795	2	596.3021	1190.5896	1190.5891	0.0006	0	72	3.4e-07	1		■	■	■	■					■					K.NQSALSSSIER.L
870	1	615.3279	1228.6412	1228.6412	0.0001	0	79	1.3e-08	1	U	■													K.QAGAAPGTALTSGK.V
874		410.8831	1229.6275	1229.6252	0.0023	0	3	0.49	1	U											■			K.LSIQVGANDGEK.I
874		410.8831	1229.6275	1228.6412	0.9863	0	2	0.69	2	U	■													K.QAGAAPGTALTSGK.V
879	4	616.8284	1231.6422	1231.6408	0.0014	0	89	1.2e-09	1	U	■													K.LVQLSNTDSAGK.V
911	4	623.8370	1245.6594	1245.6565	0.0030	0	90	9.2e-10	1	U	■													K.TALAAAGADTSGLK.L
912		416.2274	1245.6604	1245.6565	0.0039	0	4	0.39	1	U	■													K.TALAAAGADTSGLK.L
1029		652.3965	1302.7784	1301.6827	1.0958	0	4	0.74	1													■	■	K.AATLSDLDLNAAK.K
1080		336.6796	1342.6893	1343.7408	-1.0515	0	2	0.62	1	U								■						-.SLSLITQNNINK.N
1083		672.8779	1343.7412	1343.7408	0.0004	0	100	1.1e-10	1	U								■						-.SLSLITQNNINK.N
1103		683.3244	1364.6342	1364.6783	-0.0441	0	0	0.98	1	U								■						K.GSVSNTAATTDTLK.L
1222		486.2762	1455.8068	1455.8045	0.0023	0	76	3.9e-08	1		■				■									K.AQIIQQAGNSVLSK.A
1224	4	728.9116	1455.8086	1455.8045	0.0041	0	116	4.2e-12	1		■				■									K.AQIIQQAGNSVLSK.A
1232		486.8956	1457.6650	1457.7838	-0.1188	0	0	0.94	1	U												■		-.NSLSLLTQNNLNK.S
1235		729.8999	1457.7852	1457.7838	0.0015	0	59	1.3e-06	1	U												■		-.NSLSLLTQNNLNK.S
1280		498.9483	1493.8231	1493.8202	0.0029	0	20	0.056	1	U	■	■	■	■	■	■	■							K.ANQVPQQVLSLxQG.-
1281	1	747.9194	1493.8242	1493.8202	0.0041	0	57	1.3e-05	1	U	■	■	■	■	■	■	■							K.ANQVPQQVLSLxQG.-
1316		506.9344	1517.7814	1517.7950	-0.0137	0	14	0.042	1	U							■							K.ANQVPQQVLSLHQG.-
1374	4	781.4215	1560.8284	1560.8260	0.0024	0	66	1.2e-06	1		■		■	■		■	■	■	■	■				R.VSGQTQFNGVNVLAK
1382	1	521.6106	1561.8100	1560.8260	0.9840	0	53	2.5e-05	1		■		■	■		■	■	■	■	■				R.VSGQTQFNGVNVLAK
1441		807.9135	1613.8124	1613.8121	0.0003	1	100	9.6e-10	1		■	■	■	■	■	■	■	■	■	■	■	■	■	R.INSAKDDAAGQAIANR.F
1442		538.9456	1613.8150	1613.8121	0.0029	1	33	0.0045	1		■	■	■	■	■	■	■	■	■	■	■	■	■	R.INSAKDDAAGQAIANR.F
1453		810.8947	1619.7748	1619.8631	-0.0882	1	0	0.98	1	U											■			K.KVAANTSGLAANQTFK.S
1473		546.9290	1637.7652	1636.8308	0.9343	0	10	0.1	1	U											■			K.IDTGTLGLANFSVDSK.F
1479		547.6184	1639.8334	1639.8305	0.0029	0	46	2.4e-05	1	U	■													K.IDSSTLGLTGFDVSTK.A
1480	2	820.9240	1639.8334	1639.8305	0.0029	0	105	3.2e-11	1	U	■													K.IDSSTLGLTGFDVSTK.A
1526		555.6047	1663.7923	1662.8213	0.9710	0	0	1.4	1	U										■				K.IDSDTLGLNGFNVNGK.G
1532		557.9236	1670.7490	1670.7457	0.0032	0	73	3.3e-07	1		■	■	■	■	■	■	■	■		■				R.IQDADYATEVSNMSK.A
1533	7	836.3818	1670.7490	1670.7457	0.0033	0	128	1e-12	1		■	■	■	■	■	■	■	■		■				R.IQDADYATEVSNMSK.A
1536	13	836.4495	1670.8844	1670.8839	0.0005	0	123	2.8e-12	1		■	■												K.IQVGANDGQTISIDLK.K
1575		844.3781	1686.7416	1686.7407	0.0010	0	109	1e-10	1		■	■	■	■	■	■	■	■		■				R.IQDADYATEVSNMSK.A + Oxidation (M)
1585		846.4023	1690.7900	1689.9009	0.8891	1	3	0.48	1	U										■				-.QSALSSSIERLSSGLR.I
1613		570.2715	1707.7927	1707.7912	0.0015	0	73	4.6e-08	1	U	■													K.DASGNSTTAAVTLGGSDGK.T
1621	18	854.9052	1707.7958	1707.7912	0.0047	0	141	7.7e-15	1	U	■													K.DASGNSTTAAVTLGGSDGK.T
1664	1	866.4584	1730.9022	1730.9051	-0.0028	0	102	7.5e-11	1	U	■													K.VQTATTTPGTAVDVTAAK.T
1666		577.9764	1730.9074	1730.9051	0.0023	0	71	1e-07	1	U	■													K.VQTATTTPGTAVDVTAAK.T
1730	1	590.3182	1767.9328	1767.9255	0.0073	1	57	2.2e-06	1	U	■													K.KIDSSTLGLTGFDVSTK.A
1783		600.6688	1798.9846	1798.9789	0.0057	1	32	0.0013	1		■	■												K.IQVGANDGQTISIDLKK.I
1790		902.4532	1802.8918	1803.9438	-1.0520	1	1	3.6	1		■	■	■	■					■					K.NQSALSSSIERLSSGLR.I
1797		452.4763	1805.8761	1806.9476	-1.0715	1	10	0.11	1	U								■						K.KIDSSTLNLTGFNVNGK.G
1924		964.9677	1927.9208	1927.0262	0.8946	1	15	0.058	1	U											■			K.LSIQVGANDGEKIDIDLK.K
2003	2	674.6661	2020.9765	2020.9702	0.0063	0	79	1.3e-08	1	U	■													K.TYGATALNGADLSDPNNTVK.S
2004	3	1011.4960	2020.9774	2020.9702	0.0073	0	90	1e-09	1	U	■													K.TYGATALNGADLSDPNNTVK.S
2054	4	1043.0730	2084.1314	2085.1066	-0.9751	0	76	1.8e-07	4	U						■								M.AQVINTNSLSLITQNNIDK.N
2055	4	1043.0730	2084.1314	2084.1225	0.0089	0	134	2.5e-13	1		■	■	■	■			■				■			M.AQVINTNSLSLiTQNNiNK.N
2056	1	695.7178	2084.1316	2084.1225	0.0090	0	75	2e-07	1		■	■	■	■			■				■			M.AQVINTNSLSLiTQNNiNK.N
2056	1	695.7178	2084.1316	2085.0814	-0.9498	0	70	6.1e-07	4	U					■									M.AQVINTNSLSLNTQNNINK.N
2056	1	695.7178	2084.1316	2085.1066	-0.9750	0	59	8.5e-06	5	U						■								M.AQVINTNSLSLITQNNIDK.N
2059	4	1043.5640	2085.1134	2085.0814	0.0320	0	94	2.7e-09	4	U					■									M.AQVINTNSLSLNTQNNINK.N
2064		698.0079	2091.0019	2092.0900	-1.0881	0	14	0.044	1	U				■										K.TTAADVLSSLADGTTITATGVK.N
2085	1	1064.5530	2127.0914	2127.0881	0.0033	0	110	9.5e-12	1	U	■													K.SVADNAKPLAALDDAIAMVDK.F
2090	2	710.3671	2128.0795	2127.0881	0.9913	0	57	2e-06	1	U	■													K.SVADNAKPLAALDDAIAMVDK.F
2097		1072.5510	2143.0874	2143.0831	0.0044	0	84	4e-09	1	U	■													K.SVADNAKPLAALDDAIAMVDK.F + Oxidat
2098		715.3699	2143.0879	2143.0831	0.0048	0	64	4.4e-07	1	U	■													K.SVADNAKPLAALDDAIAMVDK.F + Oxidat
2159	2	1125.0570	2248.0994	2248.0931	0.0063	0	124	2.3e-12	1		■	■	■	■	■	■		■	■					R.LDSAVTNLNNTTTNLSEAQSR.I
2160	2	750.3739	2248.0999	2248.0931	0.0068	0	76	1.6e-07	1		■	■	■	■	■	■		■	■					R.LDSAVTNLNNTTTNLSEAQSR.I
2184		763.3491	2287.0255	2286.0434	0.9821	1	0	0.98	1	U								■						K.DMTITSAGGNAQVATDKAYNDK.Y + Oxida
2199		773.0778	2316.2116	2316.2074	0.0042	1	62	6.3e-07	1	U	■													R.LEEIDRVSGQTQFNGVNVLAK.D
2200		1159.1140	2316.2134	2316.2074	0.0061	1	102	5.7e-11	1	U	■													R.LEEIDRVSGQTQFNGVNVLAK.D
2221		811.0980	2430.2722	2430.2577	0.0145	1	82	6.1e-09	1	U	■													K.SVADNAKPLAALDDAIAMVDKFR.S
2222		608.5756	2430.2733	2430.2577	0.0156	1	39	0.00011	1	U	■													K.SVADNAKPLAALDDAIAMVDKFR.S
2224	1	612.5715	2446.2569	2445.1660	1.0909	1	2	0.58	2	U													■	K.ATGTDNYDVGGKTYTVNVESGAVK.N

Query	Dupes	Observed	Mr(expt)	Mr(calc)	Delta	M	Score	Expect	Rank	U	1	2	3	4	5	6	7	8	9	10	11	12	13	Peptide
2225	1	612.5720	2446.2589	2446.2526	0.0063	1	50	1e-05	1	U	■													K.SVADNAKPLAALDDAIAMVDKFR.S + Oxid
2226		816.4272	2446.2598	2446.2526	0.0072	1	68	1.5e-07	1	U	■													K.SVADNAKPLAALDDAIAMVDKFR.S + Oxid
2230		820.7711	2459.2915	2459.2867	0.0047	1	94	4.1e-10	1	U	■													K.TALAAAGADTSGLKLVQLSNTDSAGK.V
2249	1	856.0856	2565.2350	2565.2293	0.0056	0	42	0.0002	1	U	■													R.ELTVQATTGTNSESDLSSIQDEIK.S
2249		856.0856	2565.2350	2565.1930	0.0420	0	10	0.3	3										■	■				R.ELTVQASTGTNSDSDLDSIQDEIK.S
2250	8	1283.6250	2565.2354	2565.2293	0.0061	0	165	1e-16	1	U	■													R.ELTVQATTGTNSESDLSSIQDEIK.S
2256	8	1283.6260	2565.2374	2565.1930	0.0445	0	31	0.0023	3										■	■				R.ELTVQASTGTNSDSDLDSIQDEIK.S
2280	1	881.7720	2642.2942	2642.2896	0.0046	0	77	3.3e-08	1		■	■				■								R.NANDGISLAQTTEGALSEINNNLQR.V
2281	2	1322.1550	2642.2954	2642.2896	0.0059	0	130	1.6e-13	1		■	■				■								R.NANDGISLAQTTEGALSEINNNLQR.V
2289		893.7756	2678.3050	2678.2995	0.0055	1	45	2.9e-05	1	U	■													K.DASGNSTTAAVTLGGSDGKTNLVTAADGK.T
2313		937.1291	2808.3655	2808.3625	0.0030	1	88	5.1e-09	1	U	■													R.ELTVQATTGTNSESDLSSIQDEIKSR.L
2313		937.1291	2808.3655	2808.3261	0.0394	1	17	0.064	3										■	■				R.ELTVQASTGTNSDSDLDSIQDEIKSR.L
2314		941.1427	2820.4063	2820.3989	0.0074	1	109	1.3e-11	1	U	■													R.VRELTVQATTGTNSESDLSSIQDEIK.S
2337		998.1689	2991.4849	2991.4785	0.0064	1	100	9e-11	1	U	■													K.TNLVTAADGKTYGATALNGADLSPDNNTVK.S
2367		1054.5340	3160.5802	3160.5708	0.0093	1	99	3.5e-10	1		■				■			■	■					R.SSLGAVQNRLDSAVTNLNNTTTNLSEAQSR.I
2371		1077.5730	3229.6972	3229.6902	0.0070	1	90	1.4e-09	1	U											■			M.AQVINTNSLSLLTQNNLNKSQSSLSSAIER.I
2374		1086.5760	3256.7062	3256.7011	0.0051	1	130	3.6e-13	1		■	■	■	■										M.AQVINTNSLSLITQNNINKNQSALSSSIER.I
2400		1125.9430	3374.8072	3374.7570	0.0502	1	3	0.45	1	U						■								K.IDSSALGLSGFSVAGGALKLSDTVTQVGDGS
2429	1	908.6783	3630.6841	3630.6750	0.0091	0	53	4.9e-06	1	U	■													K.YYAQITSAANPGLDGAYEIHVNDADGSFTVA
2432	6	1211.2370	3630.6892	3630.6750	0.0141	0	129	1.2e-13	1	U	■													K.YYAQITSAANPGLDGAYEIHVNDADGSFTVA
2449	4	1252.9340	3755.7802	3755.7691	0.0111	0	75	2.8e-08	1	U	■													K.VTNVGYGLQNDSGTIFATDYDGTTVTTPGAET
2452		939.9536	3755.7853	3755.7691	0.0162	0	29	0.0013	1	U	■													K.VTNVGYGLQNDSGTIFATDYDGTTVTTPGAET

50 subsets and intersections (152 subset proteins in total)

10 per page 1

Not what you expected? Try **the select summary**.

Mascot: http://www.matrixscience.com/
